# Supplementary material for: Additive-controlled asymmetric iodocyclization enables enantioselective access to both α- and β-nucleosides
Source: Nat Commun. 2023 Jan 10;14:138. doi: 10.1038/s41467-022-35610-w (PMC9831021; doi:10.1038/s41467-022-35610-w)
Supplement: Supplementary file 1 — Supplementary Information [file 41467_2022_35610_MOESM1_ESM.pdf]

Supplementary Information

Additive-controlled asymmetric iodocyclization enables enantioselective access to both α- and β-nucleosides

Qi Wang[1], Jiayi Mu[1], Jie Zeng[2], Linxi Wan[1], Yangyang Zhong[1], Qiuhong Li[1], Yitong Li[1], Huijing Wang[1]*, and Fener Chen[1,3,4]*

[1] Sichuan Research Center for Drug Precision Industrial Technology, West China School of Pharmacy, Sichuan University, Chengdu 610041, China.

[2] Pharmaceutical Research Institute, Wuhan Institute of Technology, 430205 Wuhan, China.

[3] Engineering Center of Catalysis and synthesis for Chiral Molecules, Department of chemistry, Fudan University, Shanghai, 200433, China.

[4] Shanghai Engineering Center of Industrial Asymmetric Catalysis for Chiral Drugs, Shanghai 200433, China.

*Correspondence to: rfchen@fudan.edu.cn; wanghuijing@scu.edu.cn.

Table of contents

1 Supplementary Methods 2
1.1 General Information 2
1.2 Experimental Procedures 3
1.3 Crystallographic Data 54
2 Supplementary Discussion 59
2.1 Optimization of the reaction conditions 59
2.2 Mechanistic Studies. 64
3 Supplementary Figs 9-299 70
4 Supplementary Tables 15-21 216
5 Supplementary Figs 300-322 219
6 Supplementary References 242

1 Supplementary Methods

1.1 General Information

All commercially available reagents were used without further purification. Tetrahydrofuran (THF) and toluene (PhMe) were distilled from sodium/benzophenone ketyl. Chloroform and dichloromethane were distilled from CaH_2. Chromatography was conducted by using 300−400 mesh silica gel. All new compounds gave satisfactory spectroscopic analyses (^{1}H NMR, ^{13}C NMR, IR, HRMS, melting point). NMR spectra were recorded on a 400 MHz NMR or 600 MHz NMR spectrometer. Reference values for residual solvents were taken as δ = 7.26 ($CDCl_3$) ppm, δ = 3.31 (CD_3OD) ppm, δ = 2.50 ($(CD_3)_2SO$) ppm, δ = 4.70 (D_2O) ppm for ^{1}H NMR and δ = 77.16 ($CDCl_3$) ppm, δ = 49.00 (CD_3OD) ppm, δ = 39.52 ($(CD_3)_2SO$) ppm for ^{13}C NMR. Coupling constants (*J*) were given in Hz and were uncorrected and multiplicities for coupled signals were denoted as: s = singlet, d = doublet, t = triplet, q = quartet, m = multiplet, br = broad and dd = double doublet etc. Infrared (IR) spectra was recorded on a Perkin Elmer Spectrum Two FT-IR spectrometer. High-resolution mass spectra (HRMS) were recorded on a Bruker micro TOF Q III by the ESI method. Melting point (MP, for solid) was recorded on an SRS-optic melting point apparatus. Optical rotation was obtained from Rudolph Research Analytical Autopol VI automatic polarimeter. X-ray diffraction analysis was carried out by Dr. Meng Yang (Sichuan University). HPLC was performed using CHIRALCEL OD-H (4.6 x 250 mm) and Agilent 5HC C18 (250 mm x 4.6 μm) analytical column. Unless otherwise noted, all products are isolated yields.

Dimethyl sulfoxide (DMSO)	*N*, *N*-dimethylformamide (DMF)
Tetrahydrofuran (THF)	Toluene (PhMe)
Azodiisobutyronitrile (AIBN)	1,8-Diazabicyclo[5.4.0]undec-7-ene (DBU)
Diethyl azodicarboxylate (DEAD)	4-Dimethylaminopyridine (DMAP)
N, N'-dimethylethylenediamine (DMEDA)	Dess-Martin Periodinane (DMP)
Hexamethyldisilazane (HMDS)	Hexafluoroisopropanol (HFIP)
N-methylmorpholine *N*-oxide (NMO)	*N*-iodosuccinimide (NIS)
N-iodophthalimide (NIP)	*N*-bromosuccinimide (NBS)
1,3-Diiodo-5,5-dimethylhydantoin (DIDMH)	*N*-bromophthalimide (NBP)
1,3-Dibromo-5,5-dimethylhydantoin (DBDMH)	Tetrabutylammonium fluoride (TBAF)
2,4,4,6-Tetrabromocyclohexa-2,5-dienone (TBCO)	Tetrapropylammonium perruthenate (TPAP)

1.2 Experimental Procedures

Synthesis of vinyl iodine S3a.

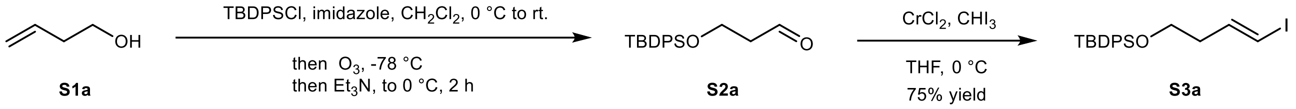

General procedure I:

Under an atmosphere of argon, a mixture of **S1a** (10.0 g, 138.7 mmol) and imidazole (11.3 g, 166.6 mmol) was added in anhydrous CH_2Cl_2 (400 mL) and stirred at 0 °C for 10 min. After adding TBDPSCl (41.9 g, 152.2 mmol), the reaction was warmed up to room temperature and stirred for 12 h. The reaction mixture was filtered with CH_2Cl_2 and cooled to –78 °C, after that O_3 was bubbled into the solution until blue color was present. Replace the stream of O_3 by O_2 until the blue color was dissipate, then Et_3N (28.1 g, 277.4 mmol) was added and stirred at 0 °C for 2 h. The solution was diluted with CH_2Cl_2 (100 mL) and saturated NH_4Cl aq. (100 mL). The layers were separated, and the aqueous layer was extracted with CH_2Cl_2 (50 mL x 4). The combined organic layer was washed with brine (50 mL x 4), dried over Na_2SO_4 and filtered, concentrated in vacuo. The crude material was purified via silica gel column chromatography to obtain **S2a**. According to this procedure, compounds **S7a**, **S7b** and **S7c** were synthesized.

General procedure II:

Under an atmosphere of argon, the $CrCl_2$ (7.7 g, 62.4 mmol) was dissolved in anhydrous THF (80 mL) at 0 °C. After stirring 30 min, CHI_3 (9.8 g, 25.0 mmol) and **S2a** (3.9 g, 12.5 mmol) was dissolved in anhydrous THF (10 mL) respectively and added to the reaction mixture. Then, the reaction mixture was warmed up to room temperature and stirred for 2 h. The solution was diluted with EtOAc (30 mL) and saturated NH_4Cl aq. (30 mL), then filtered. The layers were separated, and the aqueous layer was extracted with EtOAc (30 mL x 4). The combined organic layer was washed with brine (30 mL x 4), dried over Na_2SO_4 and filtered, concentrated in vacuo. The crude material was purified via silica gel column chromatography to obtain **S3a**. According to this procedure, compound **S3b** were synthesized.

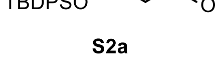

S2a

3-((tert-butyldiphenylsilyl)oxy)propanal (S2a). R_f = 0.52 (silica gel, PE:EtOAc = 7:1, UV); elution solvent is PE:EtOAc = 20:1, colourless oil (39.0 g, 90% yield). ^{1}H NMR (400 MHz, $CDCl_3$) δ 9.84 (s, 1H), 7.69 (d, *J* = 6.4 Hz, 4H), 7.50–7.36 (m, 6H), 4.05 (t, *J* = 6.0 Hz, 2H), 2.62 (td, *J* = 6.0, 2.2 Hz, 2H), 1.07 (s, 9H). ^{13}C NMR (100 MHz, $CDCl_3$) δ 202.0, 135.7 (2), 133.4 (2), 130.0 (4), 127.9 (4),

58.4, 46.5, 26.9 (3), 19.3. HRMS (ESI) m/z calcd. for $C_{19}H_{24}NaO_2Si$ $[M+Na]^+$: 335.1438, found: 335.1438. IR: 2930, 2857, 1727, 1472, 1427, 1111, 823, 739, 701 cm^{-1}.

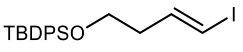

S3a

***(E)*-tert-butyl((4-iodobut-3-en-1-yl)oxy)diphenylsilane (S3a).** R_f = 0.80 (silica gel, PE, UV); elution solvent is PE, colourless oil (4.1 g, 75% yield). ^1H NMR (400 MHz, $CDCl_3$) δ 7.67 (d, J = 6.2 Hz, 4H), 7.46–7.38 (m, 6H), 6.55 (dt, J = 14.4, 7.4 Hz, 1H), 6.08 (d, J = 14.4 Hz, 1H), 3.70 (t, J = 6.4 Hz, 2H), 2.30 (dt, J = 7.4, 6.4 Hz, 2H), 1.07 (s, 9H). ^{13}C NMR (100 MHz, $CDCl_3$) δ 143.5, 135.8 (2), 133.8 (2), 129.9 (4), 127.9 (4), 76.7, 62.5, 39.3, 27.0 (3), 19.4. HRMS (ESI) m/z calcd. for $C_{20}H_{25}INaOSi$ $[M+Na]^+$: 459.0611, found: 459.0608. IR: 3063, 2928, 1644, 1427, 1259, 1110, 747, 700 cm^{-1}.

Synthesis of vinyl iodine S3b.

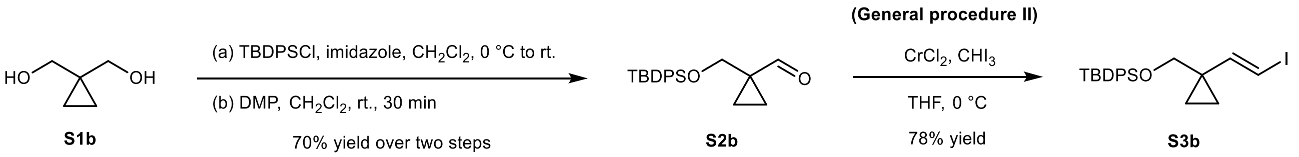

Under an atmosphere of argon, a mixture of **S1b** (2.0 g, 19.8 mmol) and imidazole (1.5 g, 21.7 mmol) was added in anhydrous CH_2Cl_2 (20 mL) and stirred at 0 °C for 10 min. After adding TBDPSCl (5.4 g, 19.8 mmol), the reaction was warmed up to room temperature and stirred for 12 h. The reaction mixture was diluted with CH_2Cl_2 (20 mL) and saturated NH_4Cl aq. (20 mL), then filtered. The layers were separated and the aqueous layer was extracted with CH_2Cl_2 (20 mL x 4). The combined organic layer was washed with brine (20 mL x 4), dried over Na_2SO_4 and filtered, concentrated in vacuo. The crude material was purified via silica gel column chromatography to obtain the protected intermediate. To a solution of the intermediate (5.5 g, 16.1 mmol) dissolved in CH_2Cl_2 (200 mL) was added dess-martin periodinane (DMP, 13.7 g, 32.3 mmol) at room temperature and stirred 20 min. The solution was quenched by saturated $NaHCO_3$ and $Na_2S_2O_3$ aq. (50 mL) and diluted with CH_2Cl_2 (50 mL), then filtered. The layers were separated, and the aqueous layer was extracted with CH_2Cl_2 (50 mL x 4). The combined organic layer was washed with saturated $NaHCO_3$ and $Na_2S_2O_3$ aq. (50 mL x 4), dried over Na_2SO_4 and filtered, concentrated in vacuo. The crude material was purified via silica gel column chromatography to obtain **S2b**.

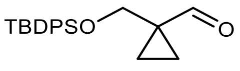

S2b

4-(((tert-butyldiphenylsilyl)oxy)methyl)cyclopropane-1-carbaldehyde (S2b). R_f = 0.54 (silica gel, PE:EtOAc = 7:1, UV); elution solvent is PE:EtOAc = 20:1, colourless oil (4.6 g, 70% yield over two steps). ^{1}H NMR (400 MHz, $CDCl_3$) δ 9.12 (s, 1H), 7.67 (d, *J* = 6.4 Hz, 4H), 7.46–7.38 (m, 6H), 3.97 (s, 2H), 1.18–1.09 (m, 4H), 1.07 (s, 9H). ^{13}C NMR (100 MHz, $CDCl_3$) δ 201.7, 135.7 (2), 133.4 (2), 129.9 (4), 127.9 (4), 62.7, 34.03, 26.9 (3), 19.4, 12.0 (2). HRMS (ESI) m/z calcd. for $C_{21}H_{26}NaO_2Si$ $[M+Na]^+$: 361.1600, found: 361.1601. IR: 2940, 2850, 1730, 1450, 1275, 1260, 1112, 740 cm^{-1}.

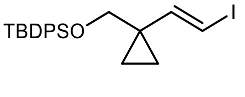

S3b

***(E)*-tert-butyl((1-(2-iodovinyl)cyclopropyl)methoxy)diphenylsilane (S3b).** R_f = 0.82 (silica gel, PE, UV); elution solvent is PE, colourless oil (4.9 g, 78% yield). ^{1}H NMR (400 MHz, $CDCl_3$) δ 7.66 (d, *J* = 6.2 Hz, 4H), 7.47–7.38 (m, 6H), 6.37 (d, *J* = 14.6 Hz, 1H), 6.04 (d, *J* = 14.6 Hz, 1H), 3.64 (s, 2H), 1.07 (s, 9H), 0.69–0.61 (m, 4H). ^{13}C NMR (100 MHz, $CDCl_3$) δ 149.4, 135.8 (2), 133.7 (2), 129.9 (4), 127.8 (4), 72.8, 67.3, 27.8, 27.0 (3), 19.5, 11.4 (2). HRMS (ESI) m/z calcd. for $C_{22}H_{27}INaOSi$ $[M+Na]^+$: 485.0774, found: 485.0774. IR: 3015, 2929, 2867, 1471, 1427, 1275, 1260, 1111, 905, 764 cm^{-1}.

Cu-catalyzed coupling reaction of C-N bonds.

General procedure: synthesis of compounds 1a-i.

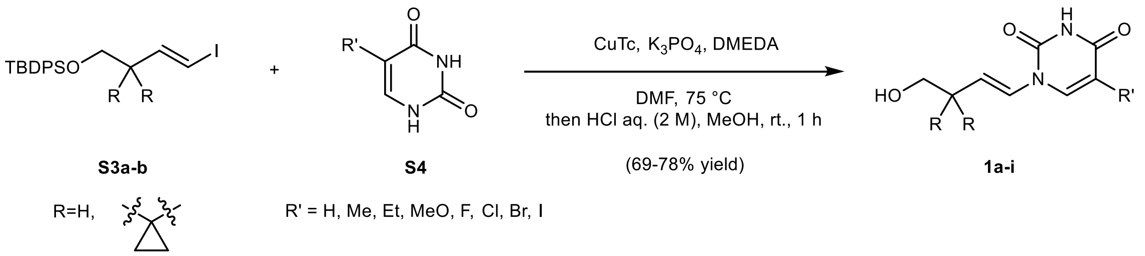

Under an atmosphere of argon, the reaction mixture of **S3** (2.0 mmol), nucleobase (**S4**, 4.0 mmol), CuTc (38 mg, 0.2 mmol) and K_3PO_4 (1.1 g, 5.0 mmol) in anhydrous *N, N*-dimethylformamide (DMF) (20 mL, 0.1 M) was degassed by bubbling argon for 10 min. After adding distilled *N, N'*-dimethylethylenediamine (DMEDA, 35 mg, 0.4 mmol), the reaction mixture was warmed up to 75 °C and stirred for 12 h. The solution was diluted with EtOAc (20 mL) and saturated NH_4Cl aq. (20 mL), then filtered. The layers were separated, and the aqueous layer was extracted with EtOAc (20 mL x 4). The combined organic layer was washed with brine (20 mL x 4), dried over Na_2SO_4 and filtered, concentrated in vacuo. The residue was dissolved in MeOH (5 mL) with addition HCl aq. (2 M, 1 mL) The mixture was stirred at room temperature for 1 h, then quenched with saturated $NaHCO_3$ aq. (2 mL). The MeOH was removed under vacuo and the aqueous layer was extracted with EtOAc (5 mL x 3). The combined organic layer was dried over Na_2SO_4 and filtered, concentrated in vacuo. The crude

material was purified via silica gel column chromatography and recrystallized from PE-MeOH mixed solution to obtain **1a-i.**

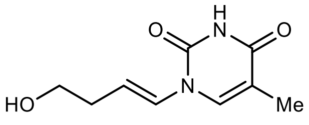

1a

***(E)*-5-methyl-1-(4-hydroxybut-1-en-1-yl) pyrimidine-2,4(1H,3H)-dione (1a).** R_f = 0.35 (silica gel, PE:acetone = 1:1, UV); elution solvent is CH_2Cl_2:MeOH = 15:1, white solid (298 mg, 76% yield). MP: 153.2–153.6 °C. ^{1}H NMR (400 MHz, CD_3OD) δ 7.68 (s, 1H), 6.92 (d, J = 14.4 Hz, 1H), 5.83 (dt, J = 14.4, 7.4 Hz, 1H), 3.65 (t, J = 6.4 Hz, 2H), 2.37 (q, J = 6.4 Hz, 2H), 1.91 (s, 3H). ^{13}C NMR (100 MHz, CD_3OD) δ 166.3, 151.4, 138.6, 126.5, 118.0, 112.2, 62.4, 34.3, 12.3. HRMS (ESI) m/z calcd. for $C_9H_{12}N_2NaO_3$ $[M+Na]^+$: 219.0740, found: 219.0741. IR: 3430, 3013, 2144, 1686, 1388, 1283, 1050, 950 cm^{-1}.

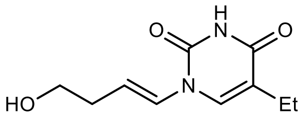

1b

***(E)*-5-ethyl-1-(4-hydroxybut-1-en-1-yl) pyrimidine-2,4(1H,3H)-dione (1b)**. R_f = 0.40 (silica gel, PE:acetone = 1:1, UV); elution solvent is CH_2Cl_2:MeOH = 15:1, white solid (315 mg, 75% yield). MP: 151.7–152.5 °C. ^{1}H NMR (400 MHz, CD_3OD) δ 7.61 (s, 1H), 6.92 (d, J = 14.4 Hz, 1H), 5.83 (dt, J = 14.4, 7.4 Hz, 1H), 3.65 (t, J = 6.4 Hz, 2H), 2.42–2.32 (m, 4H), 1.14 (t, J = 7.6 Hz, 3H). ^{13}C NMR (100 MHz, CD_3OD) δ 165.8, 151.3, 138.1, 126.6, 118.2, 118.0, 62.4, 34.3, 21.1, 13.6. HRMS (ESI) m/z calcd. for $C_{10}H_{14}N_2NaO_3$ $[M+Na]^+$: 233.0902, found: 233.0902. IR: 3450, 3050, 2944, 1665, 1445, 1285, 1052, 750 cm^{-1}.

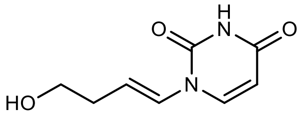

1c

***(E)*-1-(4-hydroxybut-1-en-1-yl) pyrimidine-2,4(1H,3H)-dione (1c).** R_f = 0.20 (silica gel, PE:acetone = 1:2, UV); elution solvent is CH_2Cl_2:MeOH = 10:1, white solid (255 mg, 70% yield). MP: 124.9–125.8 °C. ^{1}H NMR (400 MHz, CD_3OD) δ 7.81 (d, J = 8.0 Hz, 1H), 6.92 (d, J = 14.4 Hz, 1H), 5.84 (dt, J = 14.4, 7.4 Hz, 1H), 5.73 (d, J = 8.0 Hz, 1H), 3.65 (t, J = 6.4 Hz, 2H), 2.38 (q, J = 6.4 Hz, 2H). ^{13}C NMR (100 MHz, CD_3OD) δ 166.1, 151.3, 143.0, 126.7, 119.0, 103.2, 62.3, 34.2. HRMS (ESI) m/z calcd. for $C_8H_{10}N_2NaO_3$ $[M+Na]^+$: 205.0589, found: 205.0593. IR: 3250, 2826, 1996, 1952, 1996, 1683, 1275, 1038 cm^{-1}.

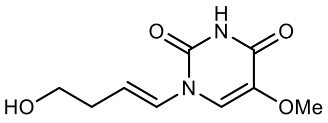

1d

***(E)*-5-methoxy-1-(4-hydroxybut-1-en-1-yl) pyrimidine-2,4(1H,3H)-dione (1d)**. R_f = 0.30 (silica gel, PE:acetone = 1:1, UV); elution solvent is CH_2Cl_2:MeOH = 15:1, white solid (314 mg, 74% yield). MP: 188.2–188.8 ° C. ^{1}H NMR (400 MHz, CD_3OD) δ 7.35 (s, 1H), 6.95 (d, J = 14.4 Hz, 1H), 5.83 (dt, J = 14.4, 7.4 Hz, 1H), 3.77 (s, 3H), 3.66 (t, J = 6.4 Hz, 2H), 2.38 (q, J = 6.4 Hz, 2H). ^{13}C NMR (100 MHz, CD_3OD) δ 161.8, 149.8, 138.4, 126.6, 121.4, 117.3, 62.5, 58.0, 34.3. HRMS (ESI) m/z calcd. for $C_9H_{12}N_2NaO_4$ $[M+Na]^+$: 235.0695, found: 235.0695. IR: 3442, 3018, 2835, 2882, 1685, 1634, 1477, 1141, 1050 cm^{-1}.

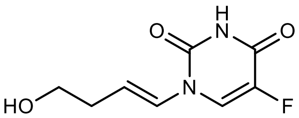

1e

***(E)*-5-fluoro-1-(4-hydroxybut-1-en-1-yl) pyrimidine-2,4(1H,3H)-dione (1e)**. R_f = 0.42 (silica gel, PE:acetone = 1:1, UV); elution solvent is CH_2Cl_2:MeOH = 15:1, white solid (292 mg, 73% yield). MP: 164.5–165.3 °C. ^{1}H NMR (400 MHz, CD_3OD) δ 8.10 (d, J = 6.6 Hz, 1H), 6.91 (d, J = 15.2 Hz, 1H), 5.82 (dt, J = 14.4, 7.4 Hz, 1H), 3.65 (t, J = 6.4 Hz, 2H), 2.37 (q, J = 6.4 Hz, 2H). ^{13}C NMR (100 MHz, CD_3OD) δ 159.4 (d, J = 27 Hz), 149.9, 142.3 (d, J = 233 Hz), 127.0 (d, J = 35 Hz),126.2, 118.5, 62.3, 34.1. HRMS (ESI) m/z calcd. for $C_8H_9FN_2NaO_3$ $[M+Na]^+$: 223.0495, found: 233.0490. IR: 3372, 2826, 1734, 1689, 1377, 1266, 1042, 780 cm^{-1}.

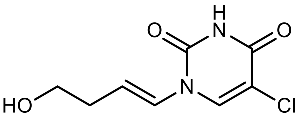

1f

***(E)*-5-chloro-1-(4-hydroxybut-1-en-1-yl) pyrimidine-2,4(1H,3H)-dione (1f)**. R_f = 0.40 (silica gel, PE:acetone = 1:1, UV); elution solvent is CH_2Cl_2:MeOH = 15:1, white solid (299 mg, 69% yield). MP: 177.2–177.8 °C. ^{1}H NMR (400 MHz, CD_3OD) δ 8.14 (s, 1H), 6.88 (d, J = 14.4 Hz, 1H), 5.89 (dt, J = 14.4, 7.4 Hz, 1H), 3.65 (t, J = 6.4 Hz, 2H), 2.38 (q, J = 6.4 Hz, 2H). ^{13}C NMR (100 MHz, CD_3OD) δ 161.5, 150.5, 140.1, 126.4, 119.7, 110.2, 62.3, 34.2. HRMS (ESI) m/z calcd. for $C_8H_9ClN_2NaO_3$ $[M+Na]^+$: 239.0199, found: 239.0199. IR: 3412, 3210, 2926, 1718, 1666, 1284, 1053, 655 cm^{-1}.

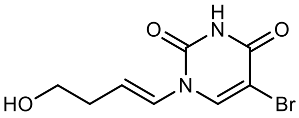

1g

***(E)*-5-bromo-1-(4-hydroxybut-1-en-1-yl)pyrimidine-2,4(1H,3H)-dione (1g)**. R_f = 0.42 (silica gel, PE:acetone = 1:1, UV); elution solvent is CH_2Cl_2:MeOH = 15:1, white solid (365 mg, 70% yield). MP: 162.0–162.6 °C. ^{1}H NMR (400 MHz, CD_3OD) δ 8.22 (s, 1H), 6.86 (d, *J* = 14.4 Hz, 1H), 5.89 (dt, *J* = 14.4, 7.4 Hz, 1H), 3.65 (t, *J* = 6.4 Hz, 2H), 2.37 (q, *J* = 6.4 Hz, 2H). ^{13}C NMR (100 MHz, CD_3OD) δ 161.5, 150.7, 142.6, 126.3, 120.0, 98.0, 62.2, 34.2. HRMS (ESI) m/z calcd. for $C_8H_9BrN_2NaO_3$ $[M+Na]^+$: 282.9694, found: 282.9691, 284.9674. IR: 3381, 3112, 2999, 1716, 1675, 1438, 1049, 858 cm^{-1}.

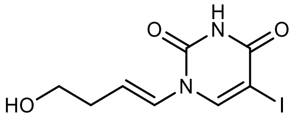

1h

***(E)*-5-iodo-1-(4-hydroxybut-1-en-1-yl) pyrimidine-2,4(1H,3H)-dione (1h)**. R_f = 0.38 (silica gel, PE:acetone = 1:1, UV); elution solvent is CH_2Cl_2:MeOH = 15:1, white solid (425 mg, 69% yield). MP: 164.9–165.7 °C. ^{1}H NMR (400 MHz, CD_3OD) δ 8.24 (s, 1H), 6.83 (d, *J* = 14.4 Hz, 1H), 5.88 (dt, *J* = 14.4, 7.4 Hz, 1H), 3.66 (t, *J* = 6.4 Hz, 2H), 2.38 (q, *J* = 6.4 Hz, 2H). ^{13}C NMR (100 MHz, CD_3OD) δ 162.8, 151.1, 147.6, 126.3, 120.1, 69.6 62.2, 34.2. HRMS (ESI) m/z calcd. for $C_8H_9IN_2NaO_3$ $[M+Na]^+$: 330.9556, found: 330.9551. IR: 3411, 3221, 2998, 1662, 1607, 1417, 1283, 1050, 947 cm^{-1}.

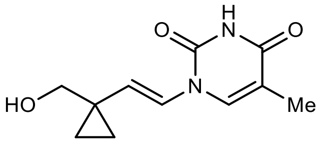

1i

***(E)*-5-methyl-1-(2-(1-(hydroxymethyl)cyclopropyl)vinyl) pyrimidine-2,4(1H,3H)-dione (1i)**. R_f = 0.44 (silica gel, PE:acetone = 1:1, UV); elution solvent is CH_2Cl_2:MeOH = 15:1, white solid (347 mg, 78% yield). MP: 162.7–163.2 °C. ^{1}H NMR (400 MHz, CD_3OD) δ 7.65 (s, 1H), 6.93 (d, *J* = 14.6 Hz, 1H), 5.77 (d, *J* = 14.6 Hz, 1H), 3.55 (s, 2H), 1.91 (s, 3H), 0.81–0.71 (m, 4H). ^{13}C NMR (100 MHz, CD_3OD) δ 166.3, 151.4, 138.8, 125.1, 124.4, 112.1, 68.4, 23.7, 12.5, 12.3 (2). HRMS (ESI) m/z calcd. for $C_{11}H_{14}N_2NaO_3$ $[M+Na]^+$: 245.0902, found: 245.0902. IR: 3442, 3004, 3010, 2886, 1720, 1607, 1444, 1345, 1066 cm^{-1}.

General synthetic route of chiral alcohol 4.

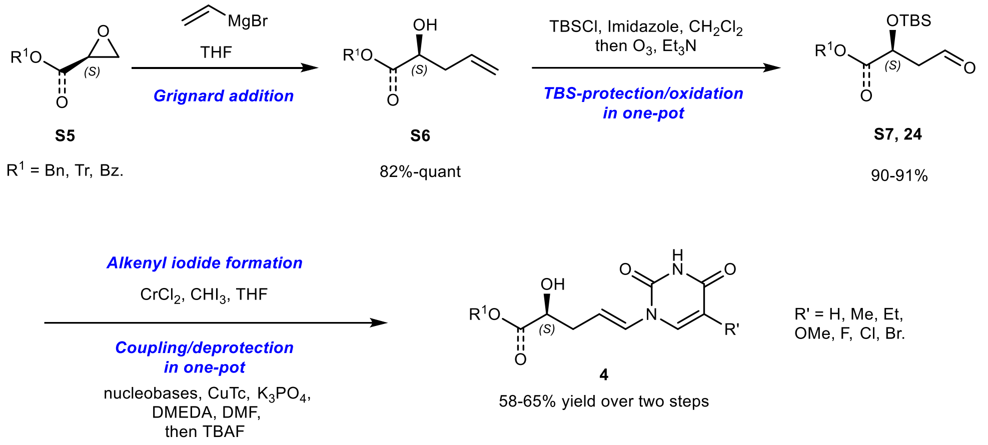

Synthesis of compounds S7a and S7c.

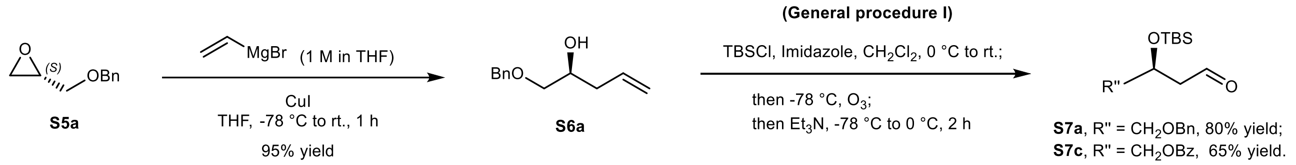

Under an atmosphere of argon, the mixture of CuI (1.1 g, 5.8 mmol) and **S5a** (10.0 g, 60.9 mmol) was dissolved in anhydrous THF (80 mL) at –78 °C. After stirring 15 min, to the reaction mixture vinylmagnesium bromide (1 M in THF, 133.9 mmol) was added dropwise via syringe, then the reaction mixture was warmed up to room temperature and stirred for 1 h. The solution was diluted with EtOAc (50 mL) and saturated NH_4Cl aq. (50 mL), then filtered. The layers were separated, and the aqueous layer was extracted with EtOAc (50 mL x 4). The combined organic layer was washed with brine (20 mL x 4), dried over Na_2SO_4 and filtered, concentrated in vacuo. The crude material was purified via silica gel column chromatography to obtain **S6a**.

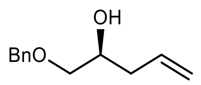

S6a

***(S)*-1-(benzyloxy)pent-4-en-2-ol (S6a).** R_f = 0.40 (silica gel, PE:EtOAc = 7:1, UV); elution solvent is PE:EtOAc = 15:1, colourless oil (11.1 g, 95% yield). $[\alpha]_D^{20}$ = +6.24 (*c* = 0.60, $CHCl_3$). ^{1}H NMR (400 MHz, $CDCl_3$) δ 7.40–7.27 (m, 5H), 5.90–5.78 (m, 1H), 5.18–5.05 (m, 2H), 4.56 (s, 2H), 3.93–3.84 (m, 1H), 3.52 (dd, *J* = 9.6, 3.4 Hz, 1H), 3.39 (dd, *J* = 9.6, 7.4 Hz, 1H), 2.54 (br, 1H), 2.27 (t, *J* = 6.8 Hz, 2H). ^{13}C NMR (100 MHz, $CDCl_3$) δ 138.0, 134.3, 128.5 (2), 127.8, 127.8 (2), 117.7, 74.0, 73.4, 69.8, 38.0. HRMS (ESI) m/z calcd. for $C_{12}H_{16}NaO_2$ $[M+Na]^+$: 215.1043, found: 215.1048. IR: 3430, 3001, 2909, 1642, 1453, 1275, 1260, 1092, 997, 764, 749 cm^{-1}.

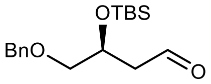

S7a

***(S)*-4-(benzyloxy)-3-((tert-butyldimethylsilyl)oxy)butanal (S7a).** R_f = 0.58 (silica gel, PE:EtOAc = 7:1, UV); elution solvent is PE:EtOAc = 20:1, colourless oil (14.3 g, 80% yield). $[\alpha]_D^{20}$ = +13.14 (*c* = 0.60, $CHCl_3$). ^1H NMR (400 MHz, $CDCl_3$) δ 9.80 (t, *J* = 2.4 Hz, 1H), 7.41–7.25 (m, 5H), 4.53 (s, 2H), 4.39–4.34 (m, 1H), 3.51 (dd, *J* = 9.6, 5.2 Hz, 1H), 3.40 (dd, *J* = 9.6, 6.2 Hz, 1H), 2.72–2.53 (m, 2H), 0.87 (s, 9H), 0.07 (s, 6H). ^{13}C NMR (100 MHz, $CDCl_3$) δ 201.4, 138.1, 128.5 (2), 127.8, 127.7 (2), 74.1, 73.5, 67.5, 49.0, 25.8 (3), 18.1, –4.4, –4.9. HRMS (ESI) m/z calcd. for $C_{17}H_{28}NaO_3Si$ $[M+Na]^+$: 331.1700, found: 331.1705. IR: 2954, 2928, 2856, 1726, 1472, 1361, 1258, 1101, 1027, 835, 745 cm^{-1}.

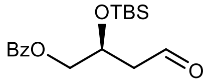

S7c

***(S)*-4-(benzoate)-3-((tert-butyldimethylsilyl)oxy)butanal (S7c).** R_f = 0.50 (silica gel, PE:EtOAc = 7:1, UV); elution solvent is PE:EtOAc = 20:1, colourless oil (12.1 g, 55% yield). $[\alpha]_D^{20}$ = +0.7 (*c* = 0.60, $CHCl_3$). ^1H NMR (400 MHz, $CDCl_3$) δ 9.85 (t, *J* = 2.2 Hz, 1H), 8.08–7.98 (m, 2H), 7.57 (t, *J* = 7.4 Hz, 1H), 7.45 (t, *J* = 7.8 Hz, 2H), 4.59–4.53 (m, 1H), 4.37 (dd, *J* = 11.2, 5.4 Hz, 1H), 4.25 (dd, *J* = 11.2, 5.4 Hz, 1H), 2.75–2.66 (m, 2H), 0.87 (s, 9H), 0.11 (s, 3H), 0.10 (s, 3H). ^{13}C NMR (150 MHz, $CDCl_3$) δ 200.7, 166.4, 133.3, 129.9, 129.8 (2), 128.6 (2), 68.0, 66.2, 48.8, 25.8 (3), 18.1, –4.4, –4.8. HRMS (ESI) m/z calcd. for $C_{17}H_{26}NaO_4Si$ $[M+Na]^+$: 345.1498, found: 345.1494. IR: 2954, 2856, 1724, 1452, 1274, 1097, 1070, 837, 760 cm^{-1}.

Synthesis of compounds 27 and 28.

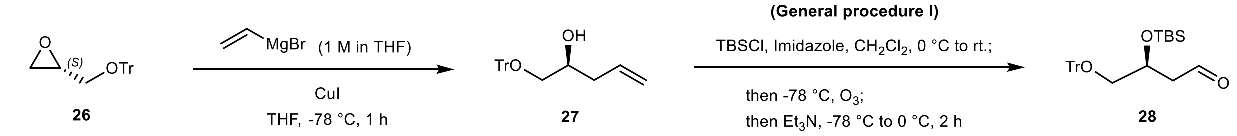

26 commercial available; 27; 28

Under an atmosphere of argon, the mixture of CuI (1.2 g, 6.3 mmol) and **26** (20.0 g, 63.2 mmol) was dissolved in anhydrous THF (150 mL) at –78 °C. After stirring 15 min, to the reaction mixture vinylmagnesium bromide (1 M in THF, 94.8 mmol) was added dropwise via syringe, then the reaction mixture was stirred for 1 h. The solution was diluted with EtOAc (100 mL) and saturated NH_4Cl aq. (100 mL), then filtered. The layers were separated, and the aqueous layer was extracted with EtOAc (50 mL x 4). The combined organic layer was washed with brine (50 mL x 4), dried over Na_2SO_4 and filtered, concentrated in vacuo. The crude product does not need purification and was directly used in

the next step.

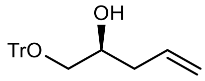

27

***(S)*-1-(trityloxy)pent-4-en-2-ol (27).** R_f = 0.50 (silica gel, PE:EtOAc = 8:1, UV); elution solvent is PE:EtOAc = 15:1, colourless oil (21.5 g, 99% yield). $[\alpha]_D^{20}$ = –8.95 (*c* = 0.60, $CHCl_3$). ^{1}H NMR (400 MHz, $CDCl_3$) δ 7.49–7.47 (m, 6H), 7.39–7.26 (m, 9H), 5.79 (td, *J* = 17.2, 7.0 Hz, 1H), 5.12–5.06 (m, 2H), 3.87 (s, 1H), 3.22 (dd, *J* = 9.4, 4.0 Hz, 1H), 3.13 (dd, *J* = 9.4, 7.0 Hz, 1H), 2.32–2.26 (m, 3H). ^{13}C NMR (100 MHz, $CDCl_3$) δ 144.0 (3), 134.4, 128.8 (6), 128.0 (6), 127.2 (3), 117.7, 86.8, 70.4, 67.2, 38.3. HRMS (ESI) m/z calcd. for $C_{24}H_{24}NaO_2$ $[M+Na]^+$: 367.1674, found: 367.1669. IR: 3495, 3057, 2874, 1597, 1490, 1448, 1070, 764, 705 cm^{-1}.

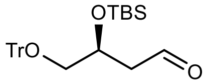

28

***(S)*-4-(trityloxy)-3-((tert-butyldimethylsilyl)oxy)butanal (28).** R_f = 0.55 (silica gel, PE:EtOAc = 10:1, UV); elution solvent is PE:EtOAc = 20:1, colourless oil (18.2 g, 91% yield). $[\alpha]_D^{20}$ = –9.04 (*c* = 0.60, $CHCl_3$). ^{1}H NMR (400 MHz, $CDCl_3$) δ 9.75 (s, 1H), 7.40–7.38 (m, 6H), 7.28–7.18 (m, 9H), 4.24–4.18 (m, 1H), 3.14 (dd, *J* = 9.2, 4.6 Hz, 1H), 3.05 (dd, *J* = 9.2, 7.0 Hz, 1H), 2.72–2.52 (m, 2H), 0.78 (s, 9H), -0.04 (s, 3H), -0.10 (s, 3H). ^{13}C NMR (100 MHz, $CDCl_3$) δ 202.0, 144.0 (3), 128.8 (6), 128.0 (6), 127.2 (3), 87.1, 67.7, 67.4, 49.3, 25.8, 18.1 (3), -4.4, -4.9. HRMS (ESI) m/z calcd. for $C_{29}H_{36}NaO_3Si$ $[M+Na]^+$: 483.2331, found: 483.2333. IR: 2929, 1727, 1448, 1253, 1219, 1077, 836, 774, 705 cm^{-1}.

Synthesis of compound S7b.

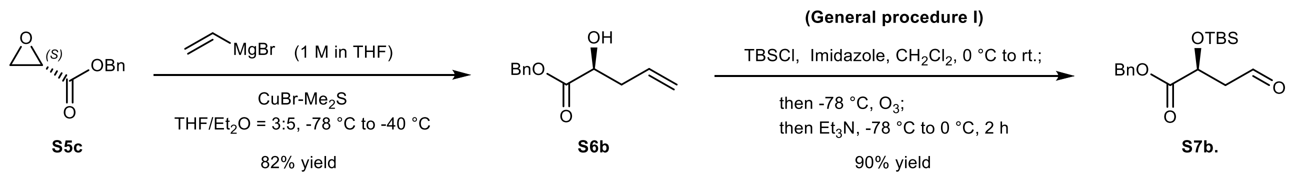

Under an atmosphere of argon, the reaction mixture of CuBr-Me_2S (12.7 g, 61.7 mmol) in anhydrous THF (120 mL) and Et_2O (24 mL) was cooled to –78 °C. After adding vinylmagnesium bromide (1 M in THF, 123.4 mmol) slowly via syringe, the reaction mixture was warmed up to –40 °C and stirred for 1.5 h. After that, **S5c** (10.0 g, 56.1 mmol) was added in the reaction solution slowly and stirred for 30 min. The solution was diluted with EtOAc (50 mL) and saturated NH_4Cl aq. (50 mL), then filtered. The layers were separated, and the aqueous layer was extracted with EtOAc (50 mL x 4).

The combined organic layer was washed with brine (50 mL x 4), dried over Na_2SO_4 and filtered, concentrated in vacuo. The crude material was purified via silica gel column chromatography to obtain **S6b**.

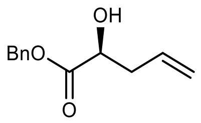

S6b

Benzyl *(S)*-2-hydroxypent-4-enoate (S6b). R_f = 0.38 (silica gel, PE:EtOAc = 5:1, UV); elution solvent is PE:EtOAc = 10:1, colourless oil (9.6 g, 82% yield). $[\alpha]_D^{20}$ = –29.67 (*c* = 0.60, $CHCl_3$). ^1H NMR (400 MHz, $CDCl_3$) δ 7.44–7.32 (m, 5H), 5.83–5.73 (m, 1H), 5.25–5.18 (m, 2H), 5.11 (d, *J* = 12.6 Hz, 2H), 4.39–4.26 (m, 1H), 2.83 (br, 1H), 2.63–2.42 (m, 2H). ^{13}C NMR (100 MHz, $CDCl_3$) δ 174.4, 135.2, 132.4, 128.8 (2), 128.7, 128.6 (2), 119.0, 70.1, 67.5, 38.8. HRMS (ESI) m/z calcd. for $C_{12}H_{14}NaO_3$ $[M+Na]^+$: 229.0835, found: 229.0831. IR: 3478, 3010, 2953, 1735, 1275, 1261, 1133, 1081, 994, 764, 750 cm^{-1}.

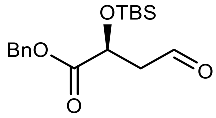

S7b

Benzyl *(S)*-2-((tert-butyldimethylsilyl)oxy)-4-oxobutanoate (S7b). R_f = 0.55 (silica gel, PE:EtOAc = 5:1, UV); elution solvent is PE:EtOAc = 15:1, colourless oil (13.5 g, 90% yield). $[\alpha]_D^{20}$ = –35.81 (*c* = 0.60, $CHCl_3$). ^1H NMR (400 MHz, $CDCl_3$) δ 9.77 (t, *J* = 1.8 Hz, 1H), 7.40–7.31 (m, 5H), 5.23–5.13 (m, 2H), 4.73 (t, *J* = 6.0 Hz, 1H), 2.83 (dd, *J* = 6.0, 1.6 Hz, 2H), 0.86 (s, 9H), 0.07 (s, 3H), 0.06 (s, 3H). ^{13}C NMR (100 MHz, $CDCl_3$) δ 199.3, 172.3, 135.3, 128.7 (2), 128.6, 128.6 (2), 67.8, 67.2, 48.4, 25.7 (3), 18.3, –4.8, –5.4. HRMS (ESI) m/z calcd. for $C_{17}H_{26}NaO_4Si$ $[M+Na]^+$: 345.1493, found: 345.1494. IR: 2953, 2929, 2857, 1731, 1463, 1361, 1276, 1259, 1160, 838, 750 cm^{-1}.

General procedure: synthesis of compounds 4a, 4c-i, 4l-m.

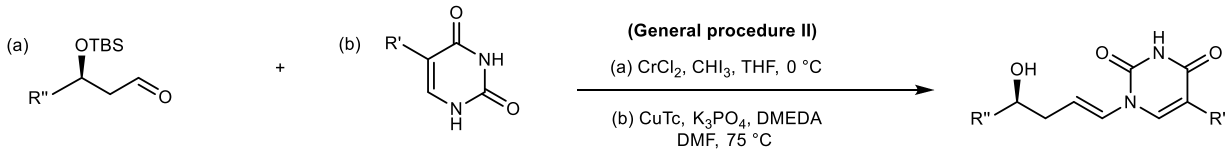

S7a-b, 24
R'' = CH_2OBn, CH_2OTr, CO_2Bn

S4
R' = H, Me, Et, MeO, F, Cl, Br

4a, 4c-i, 4l-m

(b) Under an atmosphere of argon, the reaction mixture of vinyl iodine (2.0 mmol), nucleobase (**S4**, 4.0 mmol), CuTc (38 mg, 0.2 mmol) and K_3PO_4 (1.1 g, 5.0 mmol) in anhydrous DMF (20 mL, 0.1 M) was degassed by bubbling argon for 10 min. After adding distilled DMEDA (35 mg, 0.4 mmol),

the reaction mixture was warmed up to 75 °C and stirred for 12 h and cooled to room temperature. Then tetrabutylammonium fluoride (TBAF, 1 M in THF, 3.0 mmol) was directly added to the reaction mixture. After stirring for 2 h, the solution was diluted with EtOAc (20 mL) and saturated NH_4Cl aq. (20 mL), then filtered. The layers were separated, and the aqueous layer was extracted with EtOAc (20 mL x 6). The combined organic layer was washed with brine (20 mL x 4), dried over Na_2SO_4 and filtered, concentrated in vacuo. The crude material was purified via silica gel column chromatography and recrystallized from PE-$CHCl_3$ mixed solution to obtain **4a, 4c-i, 4l-m**.

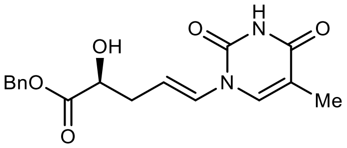

4a

Benzyl *(S,E)*-4-hydroxy-1-(5'-methyl-2',4'-dioxo-3',4'-dihydropyrimidin-1'(2H)-yl)pent-2-enoate (4a). R_f = 0.40 (silica gel, PE:acetone = 1:1, UV); elution solvent is PE:EtOAc = 1:1, white solid (489 mg, 59% yield). MP: 122.2–122.8 °C. $[\alpha]_D^{20}$ = –44.44 (*c* = 0.40, acetone). ^{1}H NMR (400 MHz, $CDCl_3$) δ 8.55 (br, 1H), 7.43–7.28 (m, 5H), 7.09 (s, 1H), 6.88 (d, *J* = 14.4 Hz, 1H), 5.50 (dt, *J* = 14.4, 7.6 Hz, 1H), 5.33–5.13 (m, 2H), 4.36 (t, *J* = 4.8 Hz,1H), 3.03 (br, 1H), 2.72–2.49 (m, 2H), 1.94 (s, 3H). ^{13}C NMR (100 MHz, $CDCl_3$) δ 174.2, 163.8, 149.4, 135.9, 135.1, 128.9 (2), 128.9, 128.8 (2), 126.7, 112.8, 111.8, 70.2, 67.9, 34.8, 12.5. HRMS (ESI) m/z calcd. for $C_{17}H_{18}N_2NaO_5$ $[M+Na]^+$: 353.1108, found: 353.1113. IR: 3452, 3016, 2970, 1738, 1435, 1365, 1435, 1365, 1217, 1107, 750 cm^{-1}.

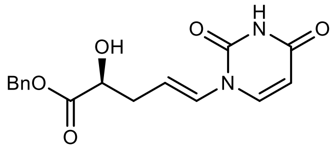

4c

Benzyl *(S,E)*-1-(2',4'-dioxo-3',4'-dihydropyrimidin-1'(2H)-yl)-4-hydroxypent-2-enoate (4c). R_f = 0.30 (silica gel, PE:acetone = 1:2, UV); elution solvent is PE:EtOAc = 1:2, white solid (455 mg, 58% yield). MP: 105.2–106.6 °C. $[\alpha]_D^{20}$ = –50.18 (*c* = 0.40, acetone). ^{1}H NMR (400 MHz, $CDCl_3$) δ 9.74 (br, 1H), 7.46–7.28 (m, 5H), 7.22 (d, *J* = 8.2 Hz, 1H), 6.85 (d, *J* = 14.4 Hz, 1H), 5.72 (d, *J* = 8.2 Hz, 1H), 5.52 (dt, *J* = 14.4, 7.6 Hz, 1H), 5.31–5.12 (m, 2H), 4.36 (q, *J* = 5.0 Hz, 1H), 3.46 (d, *J* = 4.8 Hz, 1H), 2.70–2.50 (m, 2H). ^{13}C NMR (100 MHz, $CDCl_3$) δ 174.1, 163.5, 149.4, 140.3, 135.1, 129.0 (2), 128.9, 128.8 (2), 126.8, 114.1, 103.2, 70.0, 67.8, 34.6. HRMS (ESI) m/z calcd. for $C_{16}H_{16}N_2NaO_5$ $[M+Na]^+$: 339.0951, found: 339.0955. IR: 3448, 3016, 2907, 1738, 1436, 1365, 1228, 895, 755 cm^{-1}.

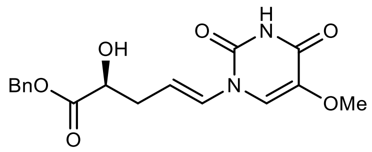

4d

Benzyl *(S,E)*-4-hydroxy-1-(5’-methoxy-2’,4’-dioxo-3’,4’-dihydropyrimidin-1’(2H)-yl)pent-2-enoate (4d). R_f = 0.30 (silica gel, PE:acetone = 1:1, UV); elution solvent is PE:EtOAc = 1:1, foam (519 mg, 60% yield). $[\alpha]_D^{20}$ = –10.12 (*c* = 0.20, acetone). ^{1}H NMR (400 MHz, CD_3OD) δ 7.40–7.24 (m, 5H), 7.11 (s, 1H), 6.81 (d, *J* = 14.4 Hz, 1H), 5.69 (dt, *J* = 14.4, 7.6 Hz, 1H), 5.25–5.10 (m, 2H), 4.35 (t, *J* = 5.6 Hz, 1H), 3.72 (s, 3H), 2.64–2.50 (m, 2H). ^{13}C NMR (100 MHz, CD_3OD) δ 174.8, 161.7, 149.6, 138.3, 137.3, 129.8 (2), 129.5 (2), 129.4, 127.6, 120.9, 114.1, 71.5, 67.8, 57.9, 36.1. HRMS (ESI) m/z calcd. for $C_{17}H_{18}N_2NaO_6$ $[M+Na]^+$: 369.1063, found: 369.1066. IR: 3496, 3016, 2970, 1738, 1436, 1365, 1228, 1217, 1030 cm^{-1}.

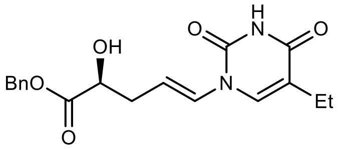

4e

Benzyl *(S,E)*-4-hydroxy-1-(5’-ethyl-2’,4’-dioxo-3’,4’-dihydropyrimidin-1’(2H)-yl)pent-2-enoate (4e). R_f = 0.42 (silica gel, PE:acetone = 1:1, UV); elution solvent is PE:EtOAc = 1:1, white solid (523 mg, 61% yield). MP: 97.4–99.2 °C. $[\alpha]_D^{20}$ = –41.02 (*c* = 0.40, acetone). ^{1}H NMR (400 MHz, $CDCl_3$) δ 9.18 (br, 1H), 7.42–7.27 (m, 5H), 7.07 (s, 1H), 6.91 (d, *J* = 14.4 Hz, 1H), 5.53 (dt, *J* = 14.4, 7.6 Hz, 1H), 5.32–5.14 (m, 2H), 4.36 (q, *J* = 5.2 Hz, 1H), 3.25 (d, *J* = 5.4 Hz, 1H), 2.72–2.50 (m, 2H), 2.36 (q, *J* = 7.4 Hz, 2H), 1.13 (t, *J* = 7.4 Hz, 3H). ^{13}C NMR (100 MHz, $CDCl_3$) δ 174.1, 163.6, 149.4, 135.2, 135.0, 128.8 (2), 128.7, 128.7 (2), 126.8, 117.5, 112.8, 70.2, 67.7, 34.8, 20.2, 13.0. HRMS (ESI) m/z calcd. for $C_{18}H_{20}N_2NaO_5$ $[M+Na]^+$: 367.1264, found: 367.1265. IR: 3450, 3016, 2970, 1738, 1438, 1366, 1228, 1217 cm^{-1}.

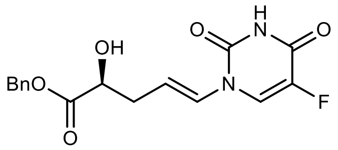

4f

Benzyl *(S,E)*-4-fluoro-1-(5’-ethyl-2’,4’-dioxo-3’,4’-dihydropyrimidin-1’(2H)-yl)pent-2-enoate (4f). R_f = 0.42 (silica gel, PE:acetone = 1:1, UV); elution solvent is PE:EtOAc = 1:1, white solid (488 mg, 58% yield). MP: 117.0–117.8 °C. $[\alpha]_D^{20}$ = –41.69 (*c* = 0.40, acetone). ^{1}H NMR (400 MHz, $CDCl_3$) δ 8.93 (br, 1H), 7.42–7.29 (m, 5H), 7.24 (s, 1H), 6.83 (d, *J* = 14.4 Hz, 1H), 5.39 (dt, *J* = 14.4, 7.6 Hz,

1H), 5.32–5.12 (m, 2H), 4.35 (q, J = 4.8 Hz, 1H), 3.03 (d, J = 4.2 Hz, 1H), 2.68–2.49 (m, 2H). ^{13}C NMR (100 MHz, $CDCl_3$) δ 174.0, 156.9 (d, J = 27 Hz), 147.9, 141.1 (d, J = 239 Hz), 135.0, 129.1 (2), 129.0, 128.9 (2), 126.4, 124.6 (d, J = 34Hz), 113.8, 70.0, 67.9, 34.5. HRMS (ESI) m/z calcd. for $C_{16}H_{15}FN_2NaO_5$ $[M+Na]^+$: 357.0857, found: 357.0862. IR: 3447, 3010, 2970, 1723, 1482, 1378,1282, 1261, 1103 cm^{-1}.

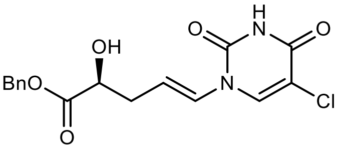

4g

Benzyl *(S,E)*-4-chloro-1-(5'-ethyl-2',4'-dioxo-3',4'-dihydropyrimidin-1'(2H)-yl)pent-2-enoate (4g). R_f = 0.37 (silica gel, PE:acetone = 1:1, UV); elution solvent is PE:EtOAc = 1:1, white solid (511 mg, 58% yield). MP: 135.0–136.3 °C. $[\alpha]_D^{20}$ = –31.65 (c = 0.40, acetone). ^{1}H NMR (400 MHz, $(CD_3)_2SO$) δ 11.97 (br, 1H), 8.17 (s, 1H), 7.38–7.30 (m, 5H), 6.75 (d, J = 14.4 Hz, 1H), 5.87 (dt, J = 14.4, 7.4 Hz, 1H), 5.62 (d, J = 6.2 Hz, 1H), 5.18–5.07 (m, 2H), 4.21 (q, J = 6.0 Hz, 1H), 2.47–2.39 (m, 2H). ^{13}C NMR (100 MHz, $(CD_3)_2SO$) δ 173.1, 158.9, 148.5, 138.1, 136.0, 128.4 (2), 128.2 (2), 128.1, 125.3, 115.0, 108.3, 69.8, 65.7, 34.7. HRMS (ESI) m/z calcd. for $C_{16}H_{15}ClN_2NaO_5$ $[M+Na]^+$: 373.0561, found: 373.0560, 375.0516. IR: 3498, 3222, 3080, 1703, 1627, 1429, 1365, 1183, 1107 cm^{-1}.

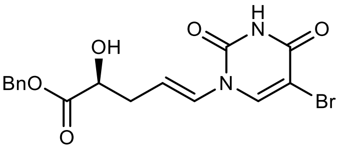

4h

Benzyl *(S,E)*-4-bromo-1-(5'-ethyl-2',4'-dioxo-3',4'-dihydropyrimidin-1'(2H)-yl)pent-2-enoate (4h). R_f = 0.39 (silica gel, PE:acetone = 1:1, UV); elution solvent is PE:EtOAc = 1:1, white solid (607 mg, 62% yield). MP: 161.6–162.5 °C. $[\alpha]_D^{20}$ = –170.96 (c = 0.20, acetone). ^{1}H NMR (400 MHz, $(CD_3)_2SO$) δ 11.93 (br, 1H), 8.22 (s, 1H), 7.41–7.27 (m, 5H), 6.73 (d, J = 14.4 Hz, 1H), 5.88 (dt, J = 14.4, 7.6 Hz, 1H), 5.62 (d, J = 6.2 Hz, 1H), 5.17–5.09 (m, 2H), 4.21 (q, J = 6.2 Hz, 1H), 2.48–2.23 (m, 2H). ^{13}C NMR (100 MHz, $(CD_3)_2SO$) δ 173.1, 159.1, 148.8, 140.4, 136.0, 128.4 (2), 128.2 (2), 128.1, 125.3, 115.1, 97.0, 69.8, 65.7, 34.7. HRMS (ESI) m/z calcd. for $C_{16}H_{15}BrN_2NaO_5$ $[M+Na]^+$: 417.0057, found: 417.0053, 419.0031. IR: 3489, 3016, 2970, 1738, 1438, 1365, 1228, 1217, 903 cm^{-1}.

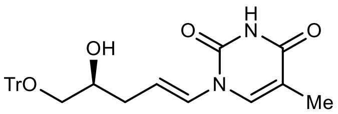

4i

***(S,E)*-1-(4-hydroxy-5-(trityloxy)pent-1-en-1-yl)-5'-methylpyrimidine-2',4'(1H,3H)-dione (4i)**. R_f

= 0.42 (silica gel, PE:acetone = 3:2, UV); elution solvent is PE:EtOAc = 2:1, white solid (608 mg, 65% yield). MP: 85.8–88.2 °C. $[\alpha]_D^{20}$ = –2.64 (*c* = 0.40, acetone). ^{1}H NMR (400 MHz, $CDCl_3$) δ 8.51 (br, 1H), 7.46–7.23 (m, 15H), 7.16 (s, 1H), 6.88 (d, *J* = 14.4 Hz, 1H), 5.56 (dt, *J* = 14.4, 7.4 Hz, 1H), 3.92–3.82 (m, 1H), 3.22 (dd, *J* = 9.4, 4.0 Hz, 1H), 3.11 (dd, *J* = 9.4, 6.6 Hz, 1H), 2.37–2.31 (m, 3H), 1.95 (s, 3H). ^{13}C NMR (100 MHz, $CDCl_3$) δ 163.9, 149.5, 143.8 (3), 136.2, 128.7 (6), 128.0 (6), 127.3 (3), 125.7, 115.3, 111.6, 86.9, 70.5, 66.8, 34.2, 12.5. HRMS (ESI) m/z calcd. for $C_{29}H_{28}N_2NaO_4$ $[M+Na]^+$: 491.1941, found: 491.1936. IR: 3446, 3016, 2970, 1738, 1448, 1365, 1228, 1217, 1091 cm^{-1}.

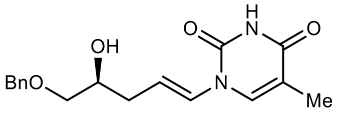

4l

***(S,E)*-1-(5-(benzyloxy)-4-hydroxypent-1-en-1-yl)-5'-methylpyrimidine-2',4'(1H,3H)-dione (4l)**. R_f = 0.40 (silica gel, PE:acetone = 3:2, UV); elution solvent is PE:EtOAc = 2:1, foam (455 mg, 58% yield). $[\alpha]_D^{20}$ = –14.31 (*c* = 0.40, acetone). ^{1}H NMR (400 MHz, $CDCl_3$) δ 9.38 (br, 1H), 7.38–7.27 (m, 5H), 6.95 (d, *J* = 14.4 Hz, 1H), 5.67 (dt, *J* = 14.4, 7.4 Hz, 1H), 4.57 (s, 2H), 3.93–3.88 (m, 1H), 3.52 (dd, *J* = 9.6, 3.6 Hz, 1H), 3.40 (dd, *J* = 9.6, 7.2 Hz, 1H), 2.81 (br, 1H), 2.38–2.28 (m, 2H), 1.94 (s, 3H). ^{13}C NMR (100 MHz, $CDCl_3$) δ 163.7, 149.4, 137.8, 136.2, 128.7 (2), 128.1, 128.0 (2), 125.7, 115.3, 111.8, 73.8, 73.6, 70.0, 34.0, 12.6. HRMS (ESI) m/z calcd. for $C_{17}H_{20}N_2NaO_4$ $[M+Na]^+$: 339.1315, found: 339.1314. IR: 3450, 3062, 2924, 1686, 1454, 1376, 1286, 1102, 950, 750 cm^{-1}.

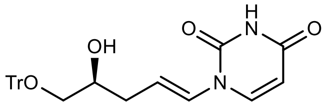

4m

***(S,E)*-1-(4-hydroxy-5-(trityloxy)pent-1-en-1-yl)-2',4'(1H,3H)-dione (4m)**. R_f = 0.25 (silica gel, PE:acetone = 3:2, UV); elution solvent is PE:EtOAc = 1:1, white solid (581 mg, 64% yield). MP: 83.2–84.0 °C. $[\alpha]_D^{20}$ = –5.39 (*c* = 0.20, acetone). ^{1}H NMR (400 MHz, $CDCl_3$) δ 9.64 (br, 1H), 7.43–7.20 (m, 15H), 7.20 (d, *J* = 8.0 Hz, 1H), 6.85 (d, *J* = 14.4 Hz, 1H), 5.69 (d, *J* = 8.0 Hz, 1H), 5.55 (dt, *J* = 14.4, 7.4 Hz, 1H), 3.87–3.82 (m, 1H), 3.18 (dd, *J* = 9.4, 4.0 Hz, 1H), 3.09 (dd, *J* = 9.4, 6.4 Hz, 1H), 2.69 (br, 1H), 2.33–2.29 (m, 2H). ^{13}C NMR (100 MHz, $CDCl_3$) δ 163.4, 149.5, 143.8 (3), 140.5, 128.7 (6), 128.0 (6), 127.3 (3), 125.8, 116.7, 103.0, 86.9, 70.4, 66.8, 34.1. HRMS (ESI) m/z calcd. for $C_{28}H_{26}N_2NaO_4$ $[M+Na]^+$: 477.1790, found: 477.1789. IR: 3440, 3010, 2965, 1690, 1441, 1216, 771, 668 cm^{-1}.

Synthesis of compound 4k.

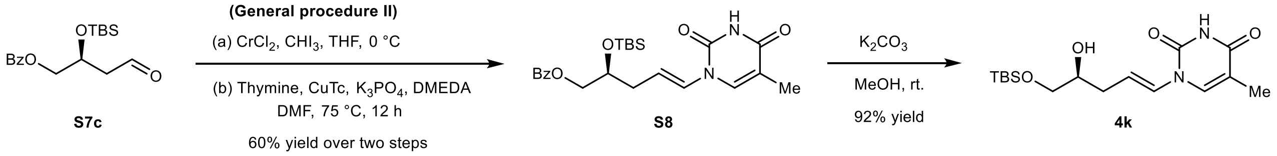

(b) Under an atmosphere of argon, the reaction mixture of vinyl iodine (10.0 g, 22.4 mmol), thymine (8.5 g, 67.2 mmol), CuTc (1.3 g, 6.7 mmol) and K_3PO_4 (12.2 g, 56.0 mmol) in anhydrous DMF (200 mL) was degassed by bubbling argon for 10 min. After adding distilled DMEDA (0.6 mg, 6.7 mmol), the reaction mixture was warmed up to 75 °C and stirred for 12 h. The solution was diluted with EtOAc (50 mL) and saturated NH_4Cl aq. (50 mL), then filtered. The layers were separated, and the aqueous layer was extracted with EtOAc (50 mL x 4). The combined organic layer was washed with brine (50 mL x 4), dried over Na_2SO_4, and filtered, concentrated in vacuo. The crude material was purified via silica gel column chromatography to obtain **S8**.

Under an atmosphere of argon, the reaction mixture of **S8** (7.5 g, 16.9 mmol) and K_2CO_3 (4.7 g, 33.8 mmol) was dissolved in anhydrous MeOH (100 mL) and stirred for 2 h at room temperature. The reaction was quenched with saturated NH_4Cl aq. (50 mL) and MeOH was removed under vacuo and the aqueous layer was extracted with $CHCl_3$ (50 mL x 3). The combined organic layer was washed with brine (50 mL x 4), dried over Na_2SO_4 and filtered, concentrated in vacuo. The crude material was purified via silica gel column chromatography and recrystallized from PE-MeOH mixed solution to obtain **4k**.

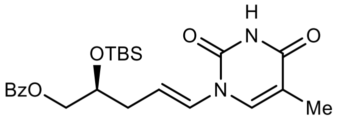

S8

***(S,E)*-4-((tert-butyldimethylsilyl)oxy)-1-(5'-methyl-2',4'-dioxo-3',4'-dihydropyrimidin-1'(2H)-yl)pent-2-en-1-yl benzoate (S8)**. R_f = 0.40 (silica gel, PE: EtOAc = 1:1, UV); elution solvent is PE:EtOAc = 6:1, white solid (10.3 g, 60% yield). MP: 124.5–126.2 °C. $[\alpha]_D^{20}$ = –1.12 (c = 0.60, $CHCl_3$). ^1H NMR (400 MHz, $CDCl_3$) δ 8.76 (br, 1H), 8.10–7.98 (m, 2H), 7.57 (t, J = 7.4 Hz, 1H), 7.45 (t, J = 7.8 Hz, 2H), 7.25 (s, 1H), 6.98 (d, J = 14.4 Hz, 1H), 5.66 (dt, J = 14.4, 7.6 Hz, 1H), 4.34–4.20 (m, 2H), 4.14–4.08 (m, 1H), 2.53–2.39 (m, 2H), 1.95 (s, 3H), 0.90 (s, 9H), 0.09 (s, 6H). ^{13}C NMR (100 MHz, $CDCl_3$) δ 166.5, 163.7, 149.4, 135.9, 133.3, 130.0, 129.8 (2), 128.6 (2), 126.1, 114.5, 111.9, 69.8, 67.9, 35.5, 25.9 (3), 18.2, 12.6, –4.4, –4.5. HRMS (ESI) m/z calcd. for $C_{23}H_{32}N_2NaO_5Si$ $[M+Na]^+$: 467.1973, found: 467.1969. IR: 3001, 2927, 1718, 1375, 1275, 1260, 1110, 764, 750 cm^{-1}.

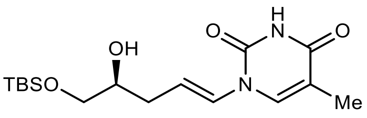

4k

***(S,E)*-1-(5-((tert-butyldimethylsilyl)oxy)-4-hydroxypent-1-en-1-yl)-5’-methylpyrimidine-2’,4’-(1H,3H)-dione (4k)**. R_f = 0.40 (silica gel, PE:acetone = 3:2, UV); elution solvent is PE:EtOAc = 2:1, white solid (7.3 g, 92% yield). MP: 119.0–120.1 °C. $[\alpha]_D^{20}$ = –8.54 (*c* = 0.20, acetone). ^{1}H NMR (400 MHz, $CDCl_3$) δ 9.84 (br, 1H), 7.33 (s, 1H), 6.95 (d, *J* = 14.4 Hz, 1H), 5.70 (dt, *J* = 14.4, 7.4 Hz, 1H), 3.74–3.70 (m, 1H), 3.62 (dd, *J* = 10.0, 4.0 Hz, 1H), 3.47 (dd, *J* = 10.0, 6.6 Hz, 1H), 2.80 (br, 1H), 2.39–2.23 (m, 2H), 1.92 (s, 3H), 0.87 (s, 9H), 0.05 (s, 6H). ^{13}C NMR (100 MHz, $CDCl_3$) δ 164.1, 149.7, 136.2, 125.5, 115.5, 111.7, 71.4, 66.7, 33.7, 25.9 (3), 18.3, 12.5, –5.3, –5.3. HRMS (ESI) m/z calcd. for $C_{16}H_{28}N_2NaO_4Si$ $[M+Na]^+$: 363.1711, found: 363.1715. IR: 3445, 3016, 2970, 1736, 1439, 1365, 1217, 1092, 837 cm^{-1}.

Synthesis of compound 4j.

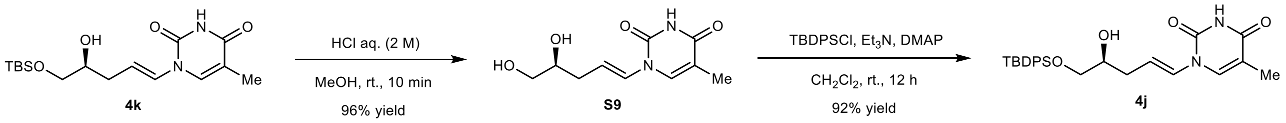

To the solution of **4k** (2.0 g, 2.9 mmol) dissolved in MeOH (15 mL) was added HCl aq. (2 M, 3 mL). The mixture was stirred at 25 °C for 1 h, then quenched with saturated $NaHCO_3$ aq. (4 mL). The MeOH was removed under vacuo and the aqueous layer was extracted with $CHCl_3$ (20 mL x 3). The combined organic layer was washed with brine (10 mL x 4), dried over Na_2SO_4 and filtered, concentrated in vacuo. The crude material was purified via silica gel column chromatography to obtain **S9**.

Mixture of **S9** (226 mg, 1.0 mmol), 4-dimethylaminopyridine (DMAP, 12 mg, 0.1 mmol) and Et_3N (121 mg, 1.2 mmol) was dissolved in anhydrous CH_2Cl_2 (5 mL) and stirred at 0 °C for 10 min. After adding TBDPSCl (330 mg, 1.2 mmol), the reaction mixture was warmed up to room temperature and stirred for 12 h. The solution was diluted with $CHCl_3$ (5 mL) and saturated NH_4Cl aq. (5 mL), then filtered. The layers were separated, and the aqueous layer was extracted with $CHCl_3$ (5 mL x 4). The combined organic layer was washed with brine (5 mL x 4), dried over Na_2SO_4 and filtered, concentrated in vacuo. The crude material was purified via silica gel column chromatography and recrystallized from PE-MeOH mixed solution to obtain **4j**.

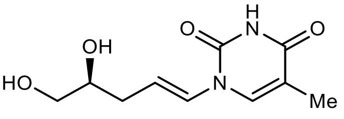

S9

***(S,E)*-1-(4,5-dihydroxypent-1-en-1-yl)-5'-methylpyrimidine-2',4'(1H,3H)-dione (S9)**. R_f = 0.20 (silica gel, CH_2Cl_2:MeOH = 10:1, UV); elution solvent is CH_2Cl_2:MeOH = 10:1, white solid (1.3 g, 96% yield). MP: 136.6–137.2 °C. $[\alpha]_D^{20}$ = –7.27 (*c* = 0.40, MeOH). ^{1}H NMR (400 MHz, CD_3OD) δ 7.68 (s, 1H), 6.91 (d, *J* = 14.4 Hz, 1H), 5.85 (dt, *J* = 14.4, 7.4 Hz, 1H), 3.72–3.66 (m, 1H), 3.59–3.44 (m, 2H), 2.44–2.22 (m, 2H), 1.91 (s, 3H). ^{13}C NMR (100 MHz, CD_3OD) δ 166.3, 151.4, 138.6, 126.8, 117.7, 112.2, 72.8, 66.6, 35.1, 12.3. HRMS (ESI) m/z calcd. for $C_{10}H_{14}N_2NaO_4$ $[M+Na]^+$: 249.0846, found: 249.0846. IR: 3369, 2921, 1666, 1387, 1444, 1284, 1032, 844 cm^{-1}.

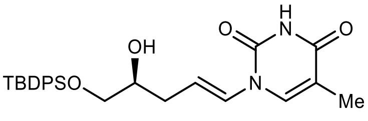

4j

***(S,E)*-1-(5-((tert-butyldiphenylsilyl)oxy-4-hydroxypent-1-en-1-yl)-5'-methylpyrimidine-2',4'-(1H,3H)-dione (4j)**. R_f = 0.43 (silica gel, PE:acetone = 3:2, UV); elution solvent is PE:EtOAc = 2:1, white solid (427 mg, 92% yield). MP: 75.0–75.8 °C. $[\alpha]_D^{20}$ = –6.56 (*c* = 0.40, acetone). ^{1}H NMR (400 MHz, $CDCl_3$) δ 9.51 (br, 1H), 7.67–7.65 (m, 4H), 7.46–7.37 (m, 6H), 7.24 (s, 1H), 6.93 (d, *J* = 14.6 Hz, 1H), 5.62 (dt, *J* = 14.6, 7.4 Hz, 1H), 3.85–3.78 (m, 1H), 3.68 (dd, *J* = 10.2, 4.0 Hz, 1H), 3.57 (dd, *J* = 10.2, 6.6 Hz, 1H), 2.72 (br, 1H), 2.32 (t, *J* = 6.6 Hz, 2H), 1.95 (s, 3H), 1.08 (s, 9H). ^{13}C NMR (100 MHz, $CDCl_3$) δ 164.0, 149.5, 136.2, 135.6 (4), 133.0 (2), 130.0 (2), 127.9 (4), 125.7, 115.4, 111.7, 71.6, 67.3, 33.6, 27.0 (3), 19.3, 12.5. HRMS (ESI) m/z calcd. for $C_{26}H_{32}N_2NaO_4Si$ $[M+Na]^+$: 487.2024, found: 487.2023. IR: 3440, 3016, 2970, 1738, 1432, 1365, 1228, 1217, 1092, 750 cm^{-1}.

Synthesis of compound 4b.

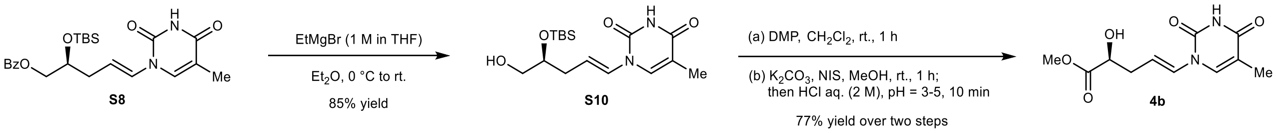

Under an atmosphere of argon, **S8** (2.0 g, 4.5 mmol) was dissolved in anhydrous Et_2O (20 mL) at 0 °C and stirred for 10 min. To the reaction mixture vinylmagnesium bromide (1 M in THF, 9.9 mmol) was added dropwise via syringe, then the reaction mixture was warmed up to room temperature and stirred for 1.5 h. The solution was diluted with EtOAc (10 mL) and saturated NH_4Cl aq. (10 mL), then filtered. The layers were separated and the aqueous layer was extracted with EtOAc (10 mL x 4). The combined organic layer was washed with brine (10 mL x 4), dried over Na_2SO_4 and filtered, concentrated in vacuo. The crude material was purified via silica gel column chromatography to obtain **S10**.

To a solution of **S10** (500 mg, 1.5 mmol) dissolved in CH_2Cl_2 (10 mL) was added DMP (1.3 g,

2.9 mmol) at room temperature and stirred for 10 min. The solution was quenched by saturated $NaHCO_3$ and $Na_2S_2O_3$ aq. (5 mL) and diluted with CH_2Cl_2 (5 mL), then filtered. The layers were separated and the aqueous layer was extracted with CH_2Cl_2 (5 mL x 4). The combined organic layer was washed with saturated $NaHCO_3$ and $Na_2S_2O_3$ aq. (5 mL x 4), dried over Na_2SO_4 and filtered, concentrated in vacuo. The crude material was purified via silica gel column chromatography to obtain aldehyde group intermediate.

Under an atmosphere of argon, K_2CO_3 (406 mg, 2.9 mmol) and NIS (585 mg, 2.6 mmol) were dissolved in anhydrous MeOH (15 mL) at room temperature and stirred for 20 min. After that, to the reaction mixture of the aldehyde group intermediate (452 mg, 1.3 mmol) was added slowly and stirred for 2 h. HCl aq. (2 M) was added dropwise in the reaction solution to adjust pH to 3–5. After that, the reaction mixture was stirred for another 2 h, then quenched with saturated $NaHCO_3$ aq. (5 mL). The MeOH was removed under vacuo and the aqueous layer was extracted with $CHCl_3$ (10 mL x 3). The combined organic layer was dried over Na_2SO_4 and filtered, concentrated in vacuo. The crude material was purified via silica gel column chromatography and recrystallized from PE-$CHCl_3$ mixed solution to obtain **4b**.

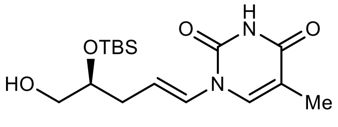

S10

***(S,E)*-1-(4-((tert-butyldimethylsilyl)oxy)-5-hydroxypent-1-en-1-yl)-5'-methylpyrimidine-2',4'-(1H,3H)-dione (S10)**. R_f = 0.40 (silica gel, PE: EtOAc = 1:1, UV); elution solvent is PE:EtOAc = 6:1, white solid (1.3 g, 85% yield). MP: 108.1–109.2 °C. $[\alpha]_D^{20}$ = +17.94 (*c* = 0.40, $CHCl_3$). ^1H NMR (400 MHz, $CDCl_3$) δ 8.65 (s, 1H), 6.93 (d, *J* = 14.4 Hz, 1H), 5.61 (dt, *J* = 14.4, 7.6 Hz, 1H), 3.86–3.79 (m, 1H), 3.59 (dd, *J* = 11.2, 4.2 Hz, 1H), 3.51 (dd, *J* = 11.2, 5.0 Hz, 1H), 2.45–2.33 (m, 2H), 1.97 (s, 3H), 1.78 (br, 1H), 0.90 (s, 9H), 0.10 (s, 3H), 0.09 (s, 3H). ^{13}C NMR (100 MHz, $CDCl_3$) δ 163.8, 149.5, 136.1, 125.9, 115.4, 111.9, 72.4, 65.8, 34.6, 25.9 (3), 18.2, 12.6, –4.4, –4.5. HRMS (ESI) m/z calcd. for $C_{16}H_{28}N_2NaO_4Si$ $[M+Na]^+$: 363.1711, found: 363.1715. IR: 3420, 3050, 2955, 1737, 1450, 1365, 1247, 750 cm^{-1}.

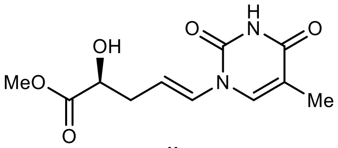

4b

Methyl *(S,E)*-4-hydroxy-1-(5'-methyl-2',4'-dioxo-3',4'-dihydropyrimidin-1'(2H)-yl)pent-2-enoate (4b). R_f = 0.40 (silica gel, PE:acetone = 1:1, UV); elution solvent is PE:EtOAc = 1:1, white solid

(745 mg, 77% yield over two steps). MP: 132.0–132.7 °C. $[\alpha]_D^{20}$ = –3.08 (*c* = 0.40, acetone). ^{1}H NMR (400 MHz, $CDCl_3$) δ 9.21 (br, 1H), 7.29 (s, 1H), 7.00 (d, *J* = 14.4 Hz, 1H), 5.64 (dt, *J* = 14.4, 7.6 Hz, 1H), 4.35−4.31 (m, 1H), 3.82 (s, 3H), 3.21 (d, *J* = 4.4 Hz, 1H), 2.73–2.49 (m, 2H), 1.95 (s, 3H). ^{13}C NMR (100 MHz, $CDCl_3$) δ 174.7, 163.8, 149.5, 135.9, 126.7, 113.1, 112.0, 70.3, 53.0, 34.8, 12.9. HRMS (ESI) m/z calcd. for $C_{11}H_{14}N_2NaO_5$ $[M+Na]^+$: 277.0795, found: 277.0798. IR: 3440, 3016, 2970, 1736, 1438, 1365, 1228, 1217, 1109, 908 cm^{-1}.

Synthesis of compound 4n.

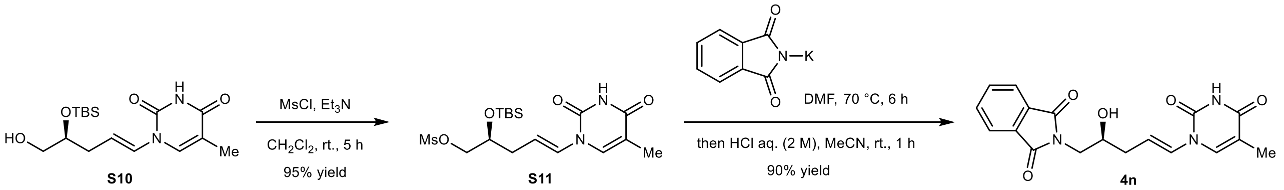

Under an atmosphere of argon, a mixture of **S10** (500 mg, 1.5 mmol) and Et_3N (182 mg, 1.8 mmol) was added in anhydrous CH_2Cl_2 (8 mL) and stirred at 0 °C for 10 min. After adding MsCl (206 mg, 1.8 mmol) slowly, the reaction mixture was warmed up to room temperature and stirred for 12 h. The solution was diluted with $CHCl_3$ (5 mL) and saturated NH_4Cl aq. (5 mL), then filtered. The layers were separated and the aqueous layer was extracted with $CHCl_3$ (5 mL x 4). The combined organic layer was washed with brine (5 mL x 4), dried over Na_2SO_4 and filtered, concentrated in vacuo. The crude material was purified via silica gel column chromatography to obtain **S11**.

Under an atmosphere of argon, a mixture of **S11** (584 mg, 1.7 mmol) and phthalimide potassium salt (635 mg, 3.4 mmol) was added in anhydrous DMF (5 mL) and stirred at 70 °C for 6 h. The solution was diluted with EtOAc (5 mL) and saturated NH_4Cl aq. (5 mL), then filtered. The layers were separated and the aqueous layer was extracted with EtOAc (5 mL x 4). The combined organic layer was washed with brine (5 mL x 4), dried over Na_2SO_4 and filtered, concentrated in vacuo. The residue was dissolved in MeCN (5 mL) with addition HCl aq. (2 M, 1 mL) The mixture was stirred at room temperature for 1 h, then quenched with saturated $NaHCO_3$ aq. (2 mL). The MeCN was removed under vacuo and the aqueous layer was extracted with EtOAc (5 mL x 3). The combined organic layer was dried over Na_2SO_4 and filtered, concentrated in vacuo. The crude material was purified via silica gel column chromatography and recrystallized from PE-MeOH mixed solution to obtain **4n**.

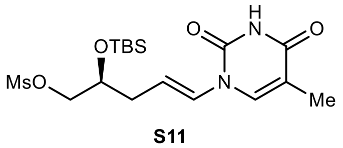

S11

***(S,E)*-4-((tert-butyldimethylsilyl)oxy)-1-(5'-methyl-2',4'-dioxo-3',4'-dihydropyrimidin-1'(2H)yl)**

pent-2-en-1-yl methanesulfonate (S11). R_f = 0.41 (silica gel, PE: EtOAc = 1:1, UV); elution solvent is PE:EtOAc = 6:1, white solid (496 mg, 95% yield). MP: 119.5–120.3 °C. $[\alpha]_D^{20}$ = +11.50 (*c* = 0.20, acetone). ^{1}H NMR (400 MHz, $CDCl_3$) δ 9.92 (br, 1H), 7.31 (s, 1H), 7.00 (d, *J* = 14.4 Hz, 1H), 5.63 (dt, *J* = 14.4, 7.6 Hz, 1H), 4.16–4.09 (m, 2H), 4.08–4.03 (m, 1H), 3.07 (s, 3H), 2.50–2.37 (m, 2H), 1.98 (s, 3H), 0.92 (s, 9H), 0.13 (s, 3H), 0.11 (s, 3H). ^{13}C NMR (100 MHz, $CDCl_3$) δ 163.5, 149.3, 135.8, 126.6, 113.7, 112.1, 71.7, 69.7, 37.6, 34.8, 25.8 (3), 18.2, 12.7, –4.5, –4.56. HRMS (ESI) m/z calcd. for $C_{17}H_{30}N_2NaO_6SSi$ $[M+Na]^+$: 441.1492, found: 441.1492. IR: 3282, 3080, 2927, 2842, 1691, 1463, 1362, 1259, 1174, 1121, 957, 837, 750 cm^{-1}.

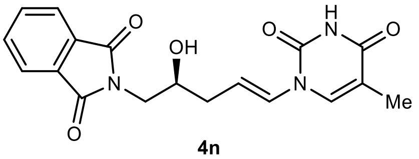

4n

***(S,E)*-1-(5-(isoindoline-1,3-dione)-4-hydroxypent-1-en-1-yl)-5'-methylpyrimidine-2',4'-(1H,3H)-dione (4n)**. R_f = 0.38 (silica gel, PE:acetone = 1:1, UV); elution solvent is PE:EtOAc = 1:1, white solid (379 mg, 90% yield). MP: 200.3–201.0 °C. $[\alpha]_D^{20}$ = –1.13 (*c* = 0.20, MeOH). ^{1}H NMR (400 MHz, CD_3OD) δ 7.87–7.64 (m, 4H), 7.64 (s, 1H), 6.94 (d, *J* = 14.4 Hz, 1H), 5.86 (dt, *J* = 14.4, 7.4 Hz, 1H), 4.08–4.00 (m, 1H), 3.77 (dd, *J* = 13.8, 7.6 Hz, 1H), 3.69 (dd, *J* = 13.8, 5.2 Hz, 1H), 2.46–2.34 (m, 2H), 1.90 (s, 3H). ^{13}C NMR (100 MHz, CD_3OD) δ 170.0 (2), 166.2, 151.3, 138.3, 135.4 (2), 133.4 (2), 127.0 (2), 124.1, 116.6, 111.2, 69.5, 44.5, 36.7, 12.3. HRMS (ESI) m/z calcd. for $C_{18}H_{17}N_3NaO_5$ $[M+Na]^+$: 378.1066, found: 378.1062. IR: 3488, 3016, 2970, 1738, 1437, 1365, 1228, 1217, 750 cm^{-1}.

Substrate scope (I).

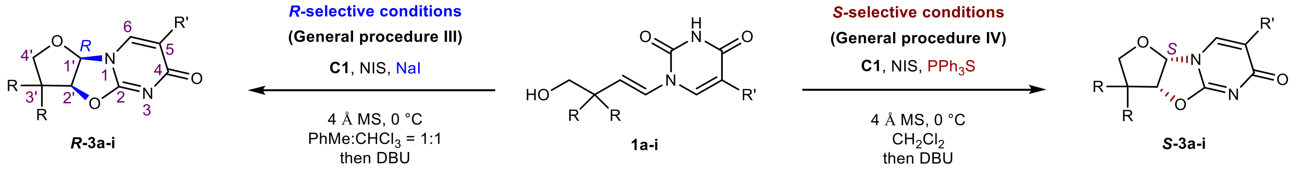

General procedure III: the catalytic enantioselective synthesis of substrates *R*-3(a-i).

Under an atmosphere of argon, a mixture of **1a-i** (0.1 mmol), **C1** (7.5 mg, 0.01 mmol), NaI (1.5 mg, 0.01 mmol) and 4 Å MS (60 mg/mmol) was dissolved in anhydrous PhMe (1.2 mL) and $CHCl_3$ (1.2 mL) at 0 °C and stirred for 15 min. After adding *N*-iodosuccinimide (NIS, 2.5 mg, 0.11 mmol), the reaction mixture was stirred for 8 h at 0 °C. After that, 1,8-diazabicyclo[5.4.0]undec-7-ene (DBU, 0.1 mmol) was added to the reaction mixture. After 30 min, the solution was diluted with $CHCl_3$ (2 mL) and saturated NH_4Cl aq. (2 mL). The layers were separated, and the aqueous layer was extracted

with $CHCl_3$ (2 mL x 4). The combined organic layer was washed with brine (2 mL x 4), dried over Na_2SO_4 and filtered, concentrated in vacuo. The crude material was purified via silica gel column chromatography to obtain ***R*-3a-i**. According to this procedure, compounds **5a-n** were synthesized.

General procedure IV: the catalytic enantioselective synthesis of substrates *S*-3(a-i).

Under an atmosphere of argon, a mixture of **1a-i** (0.1 mmol), **C1** (7.5 mg, 0.01 mmol), PPh_3S (3.0 mg, 0.01 mmol) and 4 Å MS (60 mg/mmol) was dissolved in anhydrous CH_2Cl_2 (2.4 mL) at 0 °C and stirred for 15 min. After adding NIS (22.5 mg, 0.11 mmol), the reaction mixture was stirred for 8 h at 0 °C. After that, DBU (0.1 mmol) was added to the reaction mixture. After 30 min, the solution was diluted with $CHCl_3$ (2 mL) and saturated NH_4Cl aq. (2 mL). The layers were separated, and the aqueous layer was extracted with $CHCl_3$ (2 mL x 4). The combined organic layer was washed with brine (2 mL x 4), dried over Na_2SO_4 and filtered, concentrated in vacuo. The crude material was purified via silica gel column chromatography to obtain ***S*-3a-i**. According to this procedure, compounds **6a-n** were synthesized.

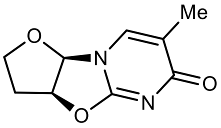

***R*-3a**

(1'*R*,2'*S*)-5-methyl-1',2',3',4'-tetrahydro-6H-furo oxazolo pyrimidin-4-one (*R*-3a). R_f = 0.30 (silica gel, PE:acetone = 1:2, UV); elution solvent is CH_2Cl_2:MeOH = 10:1, white solid (18 mg, 93% yield). MP: 185.2–186.06 °C. $[\alpha]_D^{20}$ = –26.21 (*c* = 0.30, acetone). ^{1}H NMR (400 MHz, CD_3OD) δ 7.69 (s, 1H), 6.28 (d, *J* = 5.2 Hz, 1H), 5.57 (t, *J* = 5.4 Hz, 1H), 4.20–4.16 (m, 1H), 3.73–3.66 (m, 1H), 2.38 (dd, *J* = 14.4, 4.8 Hz, 1H), 2.28– 2.16 (m, 1H), 1.94 (s, 3H). ^{13}C NMR (100 MHz, CD_3OD) δ 175.5, 162.4, 134.4, 119.4, 92.8, 85.7, 67.5, 33.6, 13.8. HRMS (ESI) m/z calcd. for $C_9H_{10}N_2NaO_3$ $[M+Na]^+$: 217.0584, found: 217.0589. IR: 3459, 3080, 2960, 1737, 1663, 1552, 1492, 1371, 1216 cm^{-1}.

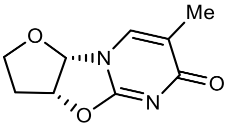

***S*-3a**

(1'*S*,2'*R*)-5-methyl-1',2',3',4'-tetrahydro-6H-furo oxazolo pyrimidin-4-one (*S*-3a). R_f = 0.30 (silica gel, PE:acetone = 1:2, UV); elution solvent is CH_2Cl_2:MeOH = 10:1, white solid (18 mg, 94% yield). MP: 175.4–176.3 °C. $[\alpha]_D^{20}$ = +27.32 (*c* = 0.30, acetone). ^{1}H NMR (400 MHz, CD_3OD) δ 7.68 (s, 1H), 6.28 (d, *J* = 5.2 Hz, 1H), 5.56 (t, *J* = 5.4 Hz, 1H), 4.23–4.14 (m, 1H), 3.73–3.66 (m, 1H), 2.38 (dd, *J* = 14.4, 4.8 Hz, 1H), 2.27–2.15 (m, 1H), 1.94 (s, 3H). ^{13}C NMR (100 MHz, CD_3OD) δ 175.5,

162.4, 134.4, 119.4, 92.8, 85.7, 67.5, 33.6, 13.8. HRMS (ESI) m/z calcd. for $C_9H_{10}N_2NaO_3$ $[M+Na]^+$: 217.0584, found: 217.0587. IR: 3459, 3077, 2960, 1737, 1660, 1548, 1492, 1371, 1202 cm^{-1}.

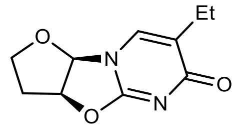

***R*-3b**

(1'*R*, 2'*S*)-5-ethyl-1',2',3',4'-tetrahydro-6H-furo oxazolo pyrimidin-4-one (*R*-3b). R_f = 0.32 (silica gel, PE:acetone = 1:2, UV); elution solvent is CH_2Cl_2:MeOH = 10:1, white solid (19 mg, 92% yield). MP: 168.6–169.7 °C. $[\alpha]_D^{20}$ = –62.12 (*c* = 0.20, acetone). ^1H NMR (400 MHz, CD_3OD) δ 7.64 (s, 1H), 6.30 (d, *J* = 5.2 Hz, 1H), 5.56 (t, *J* = 5.4 Hz, 1H), 4.23–4.15 (m, 1H), 3.73–3.67 (m, 1H), 2.44–2.34 (m, 3H), 2.28–2.17 (m, 1H), 1.15 (t, *J* = 7.4 Hz, 3H). ^{13}C NMR (100 MHz, CD_3OD) δ 174.9, 162.2, 133.6, 125.0, 92.8, 85.7, 67.5, 33.6, 22.1, 12.7. HRMS (ESI) m/z calcd. for $C_{10}H_{12}N_2NaO_3$ $[M+Na]^+$: 231.0746, found: 31.0746. IR: 3450, 2969, 1738, 1662, 1632, 1552, 1487, 1366, 1234, 1063, 796 cm^{-1}.

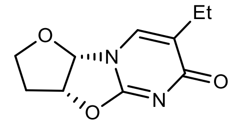

***S*-3b**

(1'*S*, 2'*R*)-5-ethyl-1',2',3',4'-tetrahydro-6H-furo oxazolo pyrimidin-4-one (*S*-3b). R_f = 0.32 (silica gel, PE:acetone = 1:2, UV); elution solvent is CH_2Cl_2:MeOH = 10:1, white solid (20 mg, 93% yield). MP: 159.8–161.4 °C. $[\alpha]_D^{20}$ = +64.10 (*c* = 0.20, acetone). ^1H NMR (400 MHz, CD_3OD) δ 7.64 (s, 1H), 6.29 (d, *J* = 5.2 Hz, 1H), 5.56 (t, *J* = 5.4 Hz, 1H), 4.22–4.15 (m, 1H), 3.73–3.67 (m, 1H), 2.43–2.35 (m, 3H), 2.27–2.18 (m, 1H), 1.15 (t, *J* = 7.4 Hz, 3H). ^{13}C NMR (100 MHz, CD_3OD) δ 174.9, 162.2, 133.6, 125.0, 92.8, 85.7, 67.5, 33.6, 22.1, 12.7. HRMS (ESI) m/z calcd. for $C_{10}H_{12}N_2NaO_3$ $[M+Na]^+$: 231.0746, found: 231.0745. IR: 3451, 2974, 1738, 1660, 1632, 1555, 1487, 1366, 1230, 1063, 796 cm^{-1}.

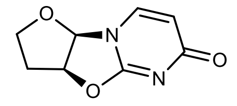

***R*-3c**

(1'*R*, 2'*S*)-5-H-1',2',3',4'-tetrahydro-6H-furo oxazolo pyrimidin-4-one (*R*-3c). R_f = 0.20 (silica gel, PE:acetone = 1:2, UV); elution solvent is CH_2Cl_2:MeOH = 8:1, white solid (16 mg, 89% yield). MP: 158.2–159.0 °C. $[\alpha]_D^{20}$ = –32.17 (*c* = 0.20, acetone). ^1H NMR (400 MHz, CD_3OD) δ 7.83 (d, *J* = 7.4 Hz, 1H), 6.31 (d, *J* = 5.2 Hz, 1H), 6.07 (d, *J* = 7.4 Hz, 1H), 5.60 (t, *J* = 5.4 Hz, 1H), 4.23–4.17 (m, 1H),

3.76–3.70 (m, 1H), 2.40 (dd, J = 14.4, 4.6 Hz, 1H), 2.29–2.19 (m, 1H). ^{13}C NMR (100 MHz, CD_3OD) δ 175.6, 162.9, 138.9, 110.0, 92.6, 86.1, 67.6, 33.6. HRMS (ESI) m/z calcd. for $C_8H_8N_2NaO_3$ $[M+Na]^+$: 203.0433, found: 203.0438. IR: 3365, 2964, 1650, 1521, 1472, 1241, 1063, 830, 746 cm^{-1}.

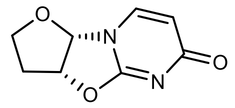

***S*-3c**

(1'*S*, 2'*R*)-5-H-1',2',3',4'-tetrahydro-6H-furo oxazolo pyrimidin-4-one (*S*-3c). R_f = 0.20 (silica gel, PE:acetone = 1:2, UV); elution solvent is CH_2Cl_2:MeOH = 8:1, white solid (17 mg, 90% yield). MP: 150.2–150.9 °C. $[\alpha]_D^{20}$ = +34.24 (c = 0.20, acetone). ^{1}H NMR (400 MHz, CD_3OD) δ 7.83 (d, J = 7.4 Hz, 1H), 6.30 (d, J = 5.2 Hz, 1H), 6.07 (d, J = 7.4 Hz, 1H), 5.60 (t, J = 5.4 Hz, 1H), 4.22–4.18 (m, 1H), 3.79–3.69 (m, 1H), 2.40 (dd, J = 14.4, 4.6 Hz, 1H), 2.28–2.18 (m, 1H). ^{13}C NMR (100 MHz, CD_3OD) δ 175.6, 162.9, 138.9, 110.0, 92.6, 86.1, 67.6, 33.6. HRMS (ESI) m/z calcd. for $C_8H_8N_2NaO_3$ $[M+Na]^+$: 203.0433, found: 203.0435. IR: 3365, 2966, 1649, 1521, 1470, 1241, 1063, 829, 746 cm^{-1}.

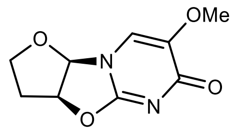

***R*-3d**

(1'*R*, 2'*S*)-5-methoxy-1',2',3',4'-tetrahydro-6H-furo oxazolo pyrimidin-4-one (*R*-3d). R_f = 0.25 (silica gel, PE:acetone = 1:2, UV); elution solvent is CH_2Cl_2:MeOH = 10:1, white solid (20 mg, 93% yield). MP: 148.9–150.3 °C. $[\alpha]_D^{20}$ = –21.33 (c = 0.20, acetone). ^{1}H NMR (400 MHz, CD_3OD) δ 7.47 (s, 1H), 6.32 (d, J = 5.2 Hz, 1H), 5.60 (t, J = 5.4 Hz, 1H), 4.22–4.18 (m, 1H), 3.77 (s, 3H), 3.75–3.68 (m, 1H), 2.38 (dd, J = 14.4, 4.6 Hz, 1H), 2.29–2.18 (m, 1H). ^{13}C NMR (100 MHz, CD_3OD) δ 169.9, 159.5, 143.8, 115.3, 93.6, 86.1, 67.5, 57.0, 33.7. HRMS (ESI) m/z calcd. for $C_9H_{10}N_2NaO_4$ $[M+Na]^+$: 233.0539, found: 233.0535. IR: 3402, 2970, 1737, 1552, 1446, 1365, 1217, 1002 cm^{-1}.

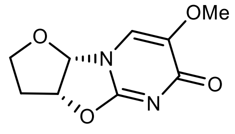

***S*-3d**

(1'*S*, 2'*R*)-5-methoxy-1',2',3',4'-tetrahydro-6H-furo oxazolo pyrimidin-4-one (*S*-3d). R_f = 0.25 (silica gel, PE:acetone = 1:2, UV); elution solvent is CH_2Cl_2:MeOH = 10:1, white solid (20 mg, 95% yield). MP: 146.2–147.6 °C. $[\alpha]_D^{20}$ = +22.87 (c = 0.20, acetone). ^{1}H NMR (400 MHz, CD_3OD) δ 7.47 (s, 1H), 6.32 (d, J = 5.2 Hz, 1H), 5.60 (t, J = 5.4 Hz, 1H), 4.22–4.18 (m, 1H), 3.78 (s, 3H), 3.75–3.68 (m, 1H), 2.39 (dd, J = 14.4, 4.6 Hz, 1H), 2.29–2.19 (m, 1H). ^{13}C NMR (100 MHz, CD_3OD) δ 169.9,

159.5, 143.8, 115.3, 93.6, 86.1, 67.5, 57.0, 33.7. HRMS (ESI) m/z calcd. for $C_9H_{10}N_2NaO_4$ $[M+Na]^+$: 233.0539, found: 233.0535. IR: 3400, 2970, 1735, 1552, 1448, 1365, 1217, 1002 cm^{-1}.

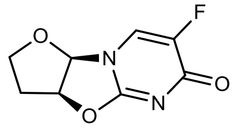

***R*-3e**

(1'*R*, 2'*S*)-5-fluoro-1',2',3',4'-tetrahydro-6H-furo oxazolo pyrimidin-4-one (*R*-3e). R_f = 0.33 (silica gel, PE:acetone = 1:2, UV); elution solvent is CH_2Cl_2:MeOH = 10:1, white solid (18 mg, 90% yield). MP: 164.5–165.3 °C. $[\alpha]_D^{20}$ = –81.92 (*c* = 0.20, acetone). ^{1}H NMR (400 MHz, CD_3OD) δ 8.05 (d, *J* = 3.8 Hz, 1H), 6.30 (d, *J* = 5.0 Hz, 1H), 5.64 (t, *J* = 5.4 Hz, 1H), 4.23–4.19 (t, *J* = 8.6 Hz, 1H), 3.82–3.74 (m, 1H), 2.40 (dd, *J* = 14.4, 4.6 Hz, 1H), 2.29–2.19 (m, 1H). ^{13}C NMR (100 MHz, CD_3OD) δ 167.3 (d, *J* = 17 Hz), 160.2, 147.4 (d, *J* = 249 Hz), 122.9 (d, *J* = 36 Hz), 93.4, 86.9, 67.7, 33.5. HRMS (ESI) m/z calcd. for $C_8H_7FN_2NaO_3$ $[M+Na]^+$: 221.0339, found: 221.0339. IR: 3456, 2966, 2855, 1643, 1554, 1503, 1307, 1248, 1065, 682 cm^{-1}.

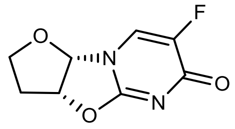

***S*-3e**

(1'*S*, 2'*R*)-5-fluoro-1',2',3',4'-tetrahydro-6H-furo oxazolo pyrimidin-4-one (*S*-3e). R_f = 0.33 (silica gel, PE:acetone = 1:2, UV); elution solvent is CH_2Cl_2:MeOH = 10:1, white solid (18 mg, 92% yield). MP: 156.8–157.9 °C. $[\alpha]_D^{20}$ = +83.45 (*c* = 0.20, acetone). ^{1}H NMR (400 MHz, CD_3OD) δ 8.05 (d, *J* = 3.8 Hz, 1H), 6.30 (d, *J* = 5.0 Hz, 1H), 5.64 (t, *J* = 5.4 Hz, 1H), 4.23–4.19 (t, *J* = 8.6 Hz, 1H), 3.81–3.73 (m, 1H), 2.40 (dd, *J* = 14.4, 4.6 Hz, 1H), 2.28–2.18 (m, 1H). ^{13}C NMR (100 MHz, CD_3OD) δ 167.3 (d, *J* = 17 Hz), 160.2, 147.4 (d, *J* = 249 Hz), 122.9 (d, *J* = 36 Hz), 93.4, 86.9, 67.7, 33.5. HRMS (ESI) m/z calcd. for $C_8H_7FN_2NaO_3$ $[M+Na]^+$: 221.0339, found: 221.0338. IR: 3454, 2966, 2855, 1636, 1554, 1503, 1307, 1250, 1065, 681 cm^{-1}.

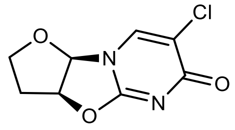

***R*-3f**

(1'*R*, 2'*S*)-5-chloro-1',2',3',4'-tetrahydro-6H-furo oxazolo pyrimidin-4-one (*R*-3f). R_f = 0.31 (silica gel, PE:acetone = 1:2, UV); elution solvent is CH_2Cl_2:MeOH = 10:1, white solid (19 mg, 89% yield). MP: 188.2–188.9 °C. $[\alpha]_D^{20}$ = –44.25 (*c* = 0.10, acetone). ^{1}H NMR (400 MHz, CD_3OD) δ 8.21

(s, 1H), 6.31 (d, J = 5.2 Hz, 1H), 5.62 (t, J = 5.6 Hz, 1H), 4.24–4.18 (m, 1H), 3.81–3.75 (m, 1H), 2.41 (dd, J = 14.4, 4.6 Hz, 1H), 2.30–2.20 (m, 1H). ^{13}C NMR (100 MHz, CD_3OD) δ 169.6, 161.8, 135.9, 118.4, 93.1, 86.8, 67.8, 33.5. HRMS (ESI) m/z calcd. for $C_8H_7ClN_2NaO_3$ $[M+Na]^+$: 237.0043, found: 237.0042, 239.0009. IR: 3418, 2920, 1738, 1634, 1538, 1486, 1365, 1233, 1076 cm^{-1}.

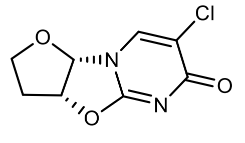

***S*-3f**

(1'*S*, 2'*R*)-5-chloro-1',2',3',4'-tetrahydro-6H-furo oxazolo pyrimidin-4-one (*S*-3f). R_f = 0.31 (silica gel, PE:acetone = 1:2, UV); elution solvent is CH_2Cl_2:MeOH = 10:1, white solid (20 mg, 91% yield). MP: 185.0–185.8 °C. $[\alpha]_D^{20}$ = +46.20 (c = 0.10, acetone). ^{1}H NMR (400 MHz, CD_3OD) δ 8.21 (s, 1H), 6.31 (d, J = 5.2 Hz, 1H), 5.62 (t, J = 5.6 Hz, 1H), 4.24–4.18 (m, 1H), 3.81–3.75 (m, 1H), 2.41 (dd, J = 14.4, 4.6 Hz, 1H), 2.30–2.20 (m, 1H). ^{13}C NMR (100 MHz, CD_3OD) δ 169.6, 161.8, 135.9, 118.4, 93.1, 86.8, 67.8, 33.5. HRMS (ESI) m/z calcd. for $C_8H_7ClN_2NaO_3$ $[M+Na]^+$: 237.0043, found: 237.0043, 239.0010. IR: 3420, 2920, 1734, 1634, 1538, 1486, 1360, 1233, 1077 cm^{-1}.

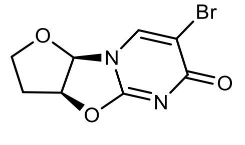

***R*-3g**

(1'*R*, 2'*S*)-5-bromo-1',2',3',4'-tetrahydro-6H-furo oxazolo pyrimidin-4-one (*R*-3g). R_f = 0.32 (silica gel, PE:acetone = 1:2, UV); elution solvent is CH_2Cl_2:MeOH = 10:1, white solid (23 mg, 88% yield). MP: 218.3–219.6 °C. $[\alpha]_D^{20}$ = −170.96 (c = 0.20, acetone). ^{1}H NMR (400 MHz, CD_3OD) δ 8.31 (s, 1H), 6.32 (d, J = 5.2 Hz, 1H), 5.63 (t, J = 5.6 Hz, 1H), 4.21 (t, J = 8.6 Hz, 1H), 3.81–3.75 (m, 1H), 2.40 (dd, J = 14.4, 4.6 Hz, 1H), 2.29–2.19 (m, 1H). ^{13}C NMR (100 MHz, CD_3OD) δ 169.7, 162.2, 138.6, 107.5, 93.0, 86.8, 67.8, 33.5. HRMS (ESI) m/z calcd. for $C_8H_7BrN_2NaO_3$ $[M+Na]^+$: 280.9538, found: 280.9538, 282.9509. IR: 3439, 2970, 1738, 1632, 1533, 1473, 1366, 1230, 1216, 1065 cm^{-1}.

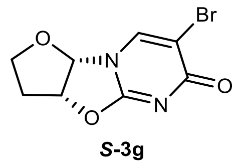

***S*-3g**

(1'*S*, 2'*R*)-5-bromo-1',2',3',4'-tetrahydro-6H-furo oxazolo pyrimidin-4-one (*S*-3g). R_f = 0.32 (silica gel, PE:acetone = 1:2, UV); elution solvent is CH_2Cl_2:MeOH = 10:1, white solid (22 mg, 85% yield). MP: 210.9–211.7 °C. $[\alpha]_D^{20}$ = +173.65 (c = 0.20, acetone). ^{1}H NMR (400 MHz, CD_3OD) δ 8.31 (s, 1H), 6.32 (d, J = 5.2 Hz, 1H), 5.63 (t, J = 5.6 Hz, 1H), 4.21 (t, J = 8.6 Hz, 1H), 3.81–3.75 (m, 1H),

2.40 (dd, *J* = 14.4, 4.6 Hz, 1H), 2.29–2.19 (m, 1H). ^{13}C NMR (100 MHz, CD_3OD) δ 169.7, 162.2, 138.6, 107.5, 93.0, 86.8, 67.8, 33.5. HRMS (ESI) m/z calcd. for $C_8H_7BrN_2NaO_3$ [M+Na]$^+$: 280.9538, found: 280.9537, 282.9509. IR: 3440, 2970, 1737, 1630, 1533, 1470, 1366, 1230, 1216, 1065 cm^{-1}.

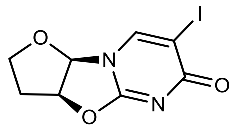

***R*-3h**

(1'*R*, 2'*S*)-5-iodo-1',2',3',4'-tetrahydro-6H-furo oxazolo pyrimidin-4-one (*R*-3h). R_f = 0.33 (silica gel, PE:acetone = 1:2, UV); elution solvent is CH_2Cl_2:MeOH = 10:1, white solid (23 mg, 86% yield). MP: 85.8–86.7 °C. $[\alpha]_D^{20}$ = –156.21 (*c* = 0.20, acetone). ^{1}H NMR (400 MHz, CD_3OD) δ 8.37 (s, 1H), 6.31 (d, *J* = 5.2 Hz, 1H), 5.62 (t, *J* = 5.4 Hz, 1H), 4.20 (t, *J* = 8.6 Hz, 1H), 3.80–3.73 (m, 1H), 2.40 (dd, *J* = 14.4, 4.6 Hz, 1H), 2.28–2.18 (m, 1H). ^{13}C NMR (100 MHz, CD_3OD) δ 171.1, 162.9, 143.8, 92.7, 86.6, 81.1, 67.8, 33.5. HRMS (ESI) m/z calcd. for $C_8H_7IN_2NaO_3$ [M+Na]$^+$: 328.9399, found: 328.9396. IR: 3455, 3010, 2970, 1738, 1436, 1365, 1228, 1217 cm^{-1}.

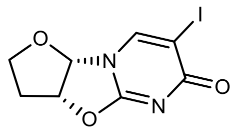

***S*-3h**

(1'*S*, 2'*R*)-5-iodo-1',2',3',4'-tetrahydro-6H-furo oxazolo pyrimidin-4-one (*S*-3h). R_f = 0.33 (silica gel, PE:acetone = 1:2, UV); elution solvent is CH_2Cl_2:MeOH = 10:1, white solid (28 mg, 90% yield). MP: 84.3–85.2 °C. $[\alpha]_D^{20}$ = +158.04 (*c* = 0.20, acetone). ^{1}H NMR (400 MHz, CD_3OD) δ 8.37 (s, 1H), 6.31 (d, *J* = 5.2 Hz, 1H), 5.62 (t, *J* = 5.4 Hz, 1H), 4.20 (t, *J* = 8.6 Hz, 1H), 3.80–3.73 (m, 1H), 2.40 (dd, *J* = 14.4, 4.6 Hz, 1H), 2.28–2.18 (m, 1H). ^{13}C NMR (100 MHz, CD_3OD) δ 171.1, 162.9, 143.8, 92.7, 86.6, 81.1, 67.8, 33.5. HRMS (ESI) m/z calcd. for $C_8H_7IN_2NaO_3$ [M+Na]$^+$: 328.9399, found: 328.9395. IR: 3455, 3010, 2970, 1743, 1440, 1365, 1228, 1214 cm^{-1}.

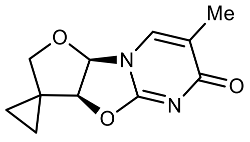

***R*-3i**

(1'*R*, 2'*S*)-5-methyl-1',2'4'-trihydro-3'-spiro(cyclopropane) furo oxazolo pyrimidin-4-one (*R*-3i). R_f = 0.35 (silica gel, PE:acetone = 1:2, UV); elution solvent is CH_2Cl_2:MeOH = 10:1, white solid (21 mg, 94% yield). MP: 219.2–220.4 °C. $[\alpha]_D^{20}$ = –113.54 (*c* = 0.20, acetone). ^{1}H NMR (400 MHz, CD_3OD) δ 7.71 (s, 1H), 6.41 (d, *J* = 5.2 Hz, 1H), 5.04 (d, *J* = 5.2 Hz, 1H), 4.00 (d, *J* = 10.0 Hz, 1H),

3.62 (d, J = 10.0 Hz, 1H), 1.96 (s, 3H), 1.25–1.20 (m, 1H), 1.05–0.99 (m, 1H), 0.98–0.91 (m, 2H). ^{13}C NMR (100 MHz, CD_3OD) δ 175.5, 162.3, 134.4, 119.4, 93.1, 91.4, 73.6, 28.0, 15.3, 13.8, 4.0. HRMS (ESI) m/z calcd. for $C_{11}H_{12}N_2NaO_3$ [M+Na]$^+$: 243.0746 found: 243.0750. IR: 3060, 2970, 1738, 1632, 1555, 1365, 1216, 1016 cm^{-1}.

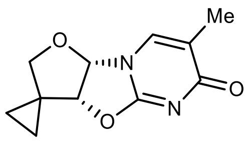

***S*-3i**

(1'*S*, 2'*R*)-5-methyl-1',2'4'-trihydro-3'-spiro(cyclopropane) furo oxazolo pyrimidin-4-one (*S*-3i). R_f = 0.35 (silica gel, PE:acetone = 1:2, UV); elution solvent is CH_2Cl_2:MeOH = 10:1, white solid (21 mg, 95% yield). MP: 218.5–220.4 °C. $[\alpha]_D^{20}$ = +114.68 (c = 0.20, acetone). ^{1}H NMR (400 MHz, CD_3OD) δ 7.71 (s, 1H), 6.41 (d, J = 5.2 Hz, 1H), 5.04 (d, J = 5.2 Hz, 1H), 4.00 (d, J = 10.0 Hz, 1H), 3.62 (d, J = 10.0 Hz, 1H), 1.96 (s, 3H), 1.25–1.20 (m, 1H), 1.05–0.99 (m, 1H), 0.98–0.91 (m, 2H). ^{13}C NMR (100 MHz, CD_3OD) δ 175.5, 162.3, 134.4, 119.4, 93.1, 91.4, 73.6, 28.0, 15.3, 13.8, 4.0. HRMS (ESI) m/z calcd. for $C_{11}H_{12}N_2NaO_3$ [M+Na]$^+$: 243.0746 found: 243.0749. IR: 3055, 2970, 1738, 1632, 1558, 1365, 1216, 1016 cm^{-1}.

Substrate scope (II).

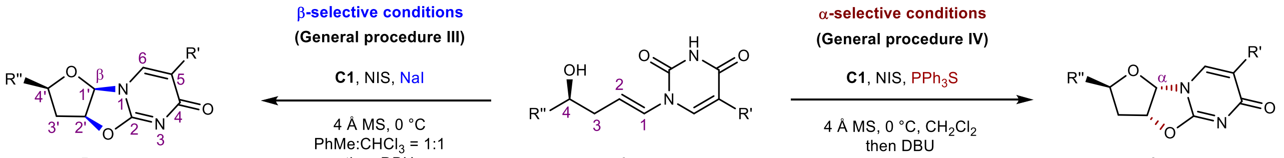

5a-n **4a-n** **6a-n**

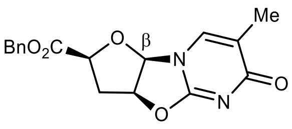

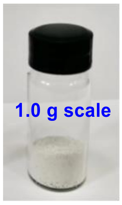

5a

Benzyl (1'*R*,2'*S*,4'*S*)-5-methyl-4-oxo-1',2',3',4'-tetrahydro-6H-furo oxazolo pyrimidine-4'-carboxylate (5a). R_f = 0.36 (silica gel, PE:acetone = 1:2, UV); elution solvent is PE:acetone = 2:1, white solid (915 mg, 92% yield). MP: 175.8–176.3 °C. $[\alpha]_D^{20}$ = –44.92 (c = 0.20, acetone). ^{1}H NMR (400 MHz, $(CD_3)_2SO$) δ 7.78 (s, 1H), 7.43–7.18 (m, 5H), 6.33 (d, J = 5.0 Hz, 1H), 5.54 (t, J = 5.0 Hz, 1H), 5.14–4.81 (m, 3H), 2.72–2.58 (m, 2H), 1.76 (s, 3H). ^{13}C NMR (100 MHz, $(CD_3)_2SO$) δ 171.7, 171.2, 159.8, 135.2, 132.5, 128.5 (2), 128.2, 128.1(2), 116.6, 91.7, 82.9, 77.6, 66.5, 35.6, 13.5. HRMS (ESI) m/z calcd. for $C_{17}H_{16}N_2NaO_5$ [M+Na]$^+$: 351.0951, found: 351.0954. IR: 3430, 3016, 2921, 1663,

1642, 1555, 1484, 1244, 1129, 1070, 1042, 927 cm^{-1}.

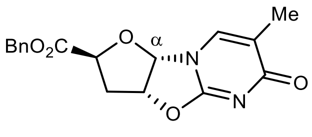

6a

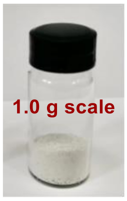

Benzyl (1’*S*,2’*R*,4’*S*)-5-methyl-4-oxo-1’,2’,3’,4’-tetrahydro-6H-furo oxazolo pyrimidine-4’-carboxylate (6a). R_f = 0.38 (silica gel, PE:acetone = 1:2, UV); elution solvent is PE:acetone = 2:1, white solid (924 mg, 93% yield). MP: 214.4–215.1 °C. $[\alpha]_D^{20}$ = +11.12 (*c* = 0.20, acetone). ^{1}H NMR (400 MHz, $(CD_3)_2SO$) δ 7.77 (s, 1H), 7.49–7.25 (m, 5H), 6.35 (d, *J* = 5.2 Hz, 1H), 5.57 (t, *J* = 5.4 Hz, 1H), 5.24–5.14 (m, 2H), 4.78 (dd, *J* = 11.0, 5.2 Hz, 1H), 2.63 (dd, *J* = 14.4, 5.4 Hz, 1H), 2.34–2.26 (m, 1H), 1.79 (s, 3H). ^{13}C NMR (100 MHz, $(CD_3)_2SO$) δ 171.6, 168.8, 159.9, 135.5, 132.3, 128.5 (2), 128.3, 128.1 (2), 116.9, 90.6, 82.6, 75.4, 66.4, 35.6, 13.5. HRMS (ESI) m/z calcd. for $C_{17}H_{16}N_2NaO_5$ $[M+Na]^+$: 351.0951, found: 351.0948. IR: 3433, 2921, 1663, 1642, 1555, 1484, 1289, 1289, 1129, 1070, 927 cm^{-1}.

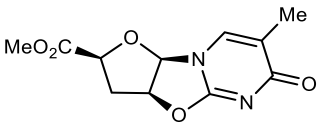

5b

Methyl (1’*R*,2’*S*,4’*S*)-5-methyl-4-oxo-1’,2’,3’,4’-tetrahydro-6H-furo oxazolo pyrimidine-4’-carboxylate (5b). R_f = 0.34 (silica gel, PE:acetone = 1:2, UV); elution solvent is PE:acetone = 2:1, white solid (22 mg, 87% yield). MP: 170.0–170.8 °C. $[\alpha]_D^{20}$ = –147.21 (*c* = 0.20, acetone). ^{1}H NMR (400 MHz, $CDCl_3$) δ 7.31 (s, 1H), 6.20 (d, *J* = 5.2 Hz, 1H), 5.45 (t, *J* = 5.4 Hz, 1H), 4.87 (d, *J* = 8.8 Hz, 1H), 3.62 (s, 3H), 2.86 (d, *J* = 15.2 Hz, 1H), 2.88–2.59 (m, 1H), 2.02 (s, 3H). ^{13}C NMR (100 MHz, $CDCl_3$) δ 172.8, 171.0, 160.2, 130.9, 119.1, 92.1, 82.6, 78.4, 53.2, 36.7, 14.2. HRMS (ESI) m/z calcd. for $C_{11}H_{12}N_2NaO_5$ $[M+Na]^+$: 275.0638, found: 275.0642. IR: 3392, 2970, 1735, 1665, 1557, 1491, 1258, 1139, 1075, 788 cm^{-1}.

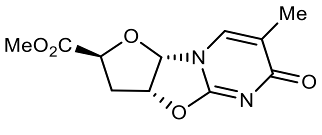

6b

Methyl (1’*S*,2’*R*,4’*S*)-5-methyl-4-oxo-1’,2’,3’,4’-tetrahydro-6H-furo oxazolo pyrimidine-4’-carboxylate (6b). R_f = 0.36 (silica gel, PE:acetone = 1:2, UV); elution solvent is PE:acetone = 2:1, white solid (23 mg, 92% yield). MP: 183.4–185.0 °C. $[\alpha]_D^{20}$ = +50.41 (*c* = 0.20, acetone). ^{1}H NMR (400 MHz, $CDCl_3$) δ 7.23 (s, 1H), 6.32 (d, *J* = 5.2 Hz, 1H), 5.54 (t, *J* = 5.4 Hz, 1H), 4.58 (dd, *J* = 11.0, 5.4 Hz, 1H), 3.79 (s, 3H), 2.78 (dd, *J* = 14.6, 5.4 Hz, 1H), 2.38–2.28 (m, 1H), 1.93 (s, 3H). ^{13}C NMR

(100 MHz, $CDCl_3$) δ 172.3, 169.1, 160.1, 130.5, 119.6, 91.1, 82.6, 75.9, 53.0, 36.5, 14.1. HRMS (ESI) m/z calcd. for $C_{11}H_{12}N_2NaO_5$ $[M+Na]^+$: 275.0638, found: 275.0640. IR: 3390, 2970, 1738, 1667, 1489, 1365, 1217, 168, 779 cm^{-1}.

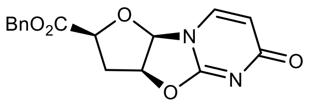

5c

Benzyl (1'*R*,2'*S*,4'*S*)-5-H-4-oxo-1',2',3',4'-tetrahydro-6H-furo oxazolo pyrimidine-4'-carboxylate (5c). R_f = 0.30 (silica gel, PE:acetone = 1:3, UV); elution solvent is PE:acetone = 1:1, white solid (28 mg, 88% yield). MP: 189.1–190.1 °C. $[\alpha]_D^{20}$ = –99.54 (*c* = 0.20, acetone). ^{1}H NMR (400 MHz, CD_3OD) δ 7.84 (d, *J* = 7.4 Hz, 1H), 7.45–7.20 (m, 5H), 6.39 (d, *J* = 5.2 Hz, 1H), 5.99 (d, *J* = 7.4 Hz, 1H), 5.62–5.59 (m, 1H), 5.18 (d, *J* = 12.2 Hz, 1H), 5.04–4.98 (m, 1H), 4.92 (d, *J* = 12.2 Hz, 1H), 2.76–2.73 (m, 2H). ^{13}C NMR (100 MHz, CD_3OD) δ 175.6, 172.6, 162.6, 139.0, 136.6, 129.7 (2), 129.6, 129.6 (2), 109.9, 93.7, 85.6, 79.9, 68.5, 37.0. HRMS (ESI) m/z calcd. for $C_{16}H_{14}N_2NaO_5$ $[M+Na]^+$: 337.0795, found: 337.0795. IR: 3474, 3016, 2919, 1737, 1475, 1370, 1217, 1077, 1035 cm^{-1}.

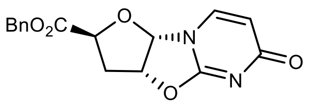

6c

Benzyl (1'*S*,2'*R*,4'*S*)-5-H-4-oxo-1',2',3',4'-tetrahydro-6H-furo oxazolo pyrimidine-4'-carboxylate (6c). R_f = 0.34 (silica gel, PE:acetone = 1:3, UV); elution solvent is PE:acetone = 1:1, white solid (24 mg, 75% yield). MP: 175.1–175.9 °C. $[\alpha]_D^{20}$ = +18.00 (*c* = 0.20, acetone). ^{1}H NMR (400 MHz, CD_3OD) δ 7.85 (d, *J* = 7.4 Hz, 1H), 7.47–7.24 (m, 5H), 6.41 (d, *J* = 5.2 Hz, 1H), 6.07 (d, *J* = 7.4 Hz, 1H), 5.64 (t, *J* = 5.6 Hz, 1H), 5.22 (s, 2H), 4.79–4.74 (m, 1H), 2.76 (dd, *J* = 14.6, 5.4 Hz, 1H), 2.43–2.35 (m, 1H). ^{13}C NMR (100 MHz, CD_3OD) δ 175.4, 170.4, 162.6, 138.8, 136.8, 129.6 (2), 129.6, 129.4 (2), 110.1, 92.8, 85.4, 77.4, 68.4, 37.0. HRMS (ESI) m/z calcd. for $C_{16}H_{14}N_2NaO_5$ $[M+Na]^+$: 337.0795, found: 337.0796. IR: 3474, 3016, 2970, 1739, 1650, 1524, 1365, 1228, 1075, 1033 cm^{-1}.

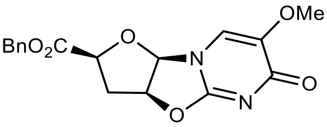

5d

Benzyl (1'*R*,2'*S*,4'*S*)-5-methoxy-4-oxo-1',2',3',4'-tetrahydro-6H-furo oxazolo pyrimidine-4'-carboxylate (5d). R_f = 0.38 (silica gel, PE:acetone = 1:2, UV); elution solvent is PE:acetone = 2:1,

white solid (31 mg, 91% yield). MP: 175.8–176.9 °C. $[\alpha]_D^{20}$ = –111.21 (*c* = 0.20, acetone). ^{1}H NMR (400 MHz, CD_3OD) δ 7.44 (s, 1H), 7.42–7.17 (m, 5H), 6.38 (d, *J* = 5.2 Hz, 1H), 5.61 (t, *J* = 4.4 Hz, 1H), 5.17 (d, *J* = 12.2 Hz, 1H), 5.02 (dd, *J* = 7.4, 2.8 Hz, 1H), 4.87 (d, *J* = 12.2 Hz, 1H), 3.67 (s, 3H), 2.78–2.69 (m, 2H). ^{13}C NMR (100 MHz, CD_3OD) δ 172.6, 169.9, 159.3, 143.7, 136.4, 129.7 (2), 129.6, 129.6 (2), 115.5, 94.6, 85.6, 79.8, 68.6, 56.8, 37.0. HRMS (ESI) m/z calcd. for $C_{17}H_{16}N_2NaO_6$ $[M+Na]^+$: 367.0906, found: 367.0906. IR: 3441, 3016, 2917, 1634, 1567, 1500, 1300, 1260, 1073, 793 cm^{-1}.

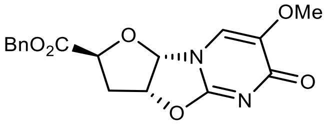

6d

Benzyl (1’*S*,2’*R*,4’*S*)-5-methoxy-4-oxo-1’,2’,3’,4’-tetrahydro-6H-furo oxazolo pyrimidine-4’-carboxylate (6d). R_f = 0.40 (silica gel, PE:acetone = 1:2, UV); elution solvent is PE:acetone = 2:1, white solid (32 mg, 94% yield). MP: 90.5–91.6 °C. $[\alpha]_D^{20}$ = +32.33 (*c* = 0.20, acetone). ^{1}H NMR (400 MHz, $(CD_3)_2CO$) δ 7.51–7.33 (m, 5H), 7.30 (s, 1H), 6.41 (d, *J* = 5.2 Hz, 1H), 5.68 (t, *J* = 5.6 Hz, 1H), 5.24 (s, 2H), 4.83 (dd, *J* = 11.0, 5.4 Hz, 1H), 3.69 (s, 3H), 2.73 (dd, *J* = 14.4, 5.4 Hz, 1H), 2.42 (ddd, *J* = 14.4, 10.0, 6.0 Hz, 1H). ^{13}C NMR (100 MHz, $(CD_3)_2CO$) δ 169.6, 166.4, 158.6, 143.6, 136.7, 129.4 (2), 129.2, 129.1 (2), 114.1, 92.8, 84.0, 76.7, 67.5, 56.5, 37.1. HRMS (ESI) m/z calcd. for $C_{17}H_{16}N_2NaO_6$ $[M+Na]^+$: 367.0906, found: 367.0905. IR: 3341, 3016, 2917, 1738, 1440, 1365, 1226, 1091, 774 cm^{-1}.

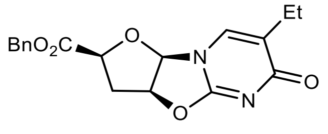

5e

Benzyl (1’*R*,2’*S*,4’*S*)-5-ethyl-4-oxo-1’,2’,3’,4’-tetrahydro-6H-furo oxazolo pyrimidine-4’-carboxylate (5e). R_f = 0.38 (silica gel, PE:acetone = 1:2, UV); elution solvent is PE:acetone = 2:1, white solid (32 mg, 93% yield). MP: 149.5–150.0 °C. $[\alpha]_D^{20}$ = –58.12 (*c* = 0.20, acetone). ^{1}H NMR (400 MHz, $CDCl_3$) δ 7.49–7.19 (m, 5H), 7.15 (s, 1H), 6.24 (d, *J* = 5.2 Hz, 1H), 5.48 (t, *J* = 5.4 Hz, 1H), 5.16 (d, *J* = 12.2 Hz, 1H), 4.88 (d, *J* = 12.2 Hz, 1H), 4.86 (s, 1H), 2.83 (d, *J* = 15.2 Hz, 1H), 2.66–2.58 (m, 1H), 2.40–2.23 (m, 2H), 1.07 (t, *J* = 7.4 Hz, 3H). ^{13}C NMR (100 MHz, $CDCl_3$) δ 172.2, 170.5, 159.9, 134.7, 129.7, 128.8 (2), 128.7, 128.7 (2), 124.6, 92.2, 82.5, 78.5, 68.0, 36.5, 21.2, 11.9. HRMS (ESI) m/z calcd. for $C_{18}H_{18}N_2NaO_5$ $[M+Na]^+$: 365.1108, found: 365.1110. IR: 3445, 2970, 1739, 1635, 1555, 1485, 1365, 1217, 1075, 873 cm^{-1}.

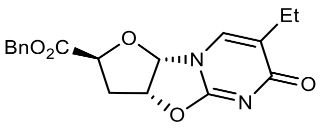

6e

Benzyl (1'*S*,2'*R*,4'*S*)-5-ethyl-4-oxo-1',2',3',4'-tetrahydro-6H-furo oxazolo pyrimidine-4'-carboxylate (6e). R_f = 0.40 (silica gel, PE:acetone = 1:2, UV); elution solvent is PE:acetone = 2:1, white solid (32 mg, 92% yield). MP: 192.2–193.5 °C. $[\alpha]_D^{20}$ = +49.46 (*c* = 0.20, acetone). ^{1}H NMR (400 MHz, $CDCl_3$) δ 7.51–7.27 (m, 5H), 7.15 (s, 1H), 6.32 (d, *J* = 5.2 Hz, 1H), 5.52 (t, *J* = 5.4 Hz, 1H), 5.26–5.16 (m, 2H), 4.60 (dd, *J* = 11.0, 5.2 Hz, 1H), 2.77 (dd, *J* = 14.4, 5.4 Hz, 1H), 2.36 (q, *J* = 7.4 Hz, 2H), 2.33–2.22 (m, 1H), 1.11 (t, *J* = 7.4 Hz, 3H). ^{13}C NMR (100 MHz, $CDCl_3$) δ 171.9, 168.6, 159.8, 134.7, 129.6, 128.9 (2), 128.9 128.6 (2), 125.0, 91.2, 82.5, 75.9, 67.8, 36.5, 21.2, 11.9. HRMS (ESI) m/z calcd. for $C_{18}H_{18}N_2NaO_5$ $[M+Na]^+$: 365.1108, found: 365.1111. IR: 3449, 2970, 1738, 1639, 1558, 1462, 1365, 1217, 1083, 900 cm^{-1}.

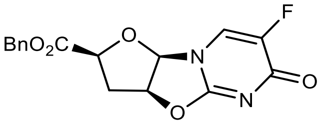

5f

Benzyl (1'*R*,2'*S*,4'*S*)-5-fluoro-4-oxo-1',2',3',4'-tetrahydro-6H-furo oxazolo pyrimidine-4'-carboxylate (5f). R_f = 0.42 (silica gel, PE:acetone = 1:2, UV); elution solvent is PE:acetone = 3:1, white solid (30 mg, 89% yield). MP: 175.4–175.9 °C. $[\alpha]_D^{20}$ = –99.93 (*c* = 0.20, acetone). ^{1}H NMR (400 MHz, CD_3OD) δ 8.06 (d, *J* = 4.0 Hz, 1H), 7.46–7.20 (m, 5H), 6.38 (d, *J* = 5.2 Hz, 1H), 5.67–5.64 (m, 1H), 5.18 (d, *J* = 12.2 Hz, 1H), 5.06–5.00 (m, 1H), 4.94 (d, *J* = 12.2 Hz, 1H), 2.76–2.73 (m, 2H). ^{13}C NMR (100 MHz, CD_3OD) δ 172.7, 167.3 (d, *J* = 17 Hz), 160.0, 147.3 (d, *J* = 249 Hz), 136.5, 129.7 (2), 129.7, 129.6 (2), 123.2 (d, *J* = 37 Hz), 94.4, 86.4, 80.0, 68.6, 37.0. HRMS (ESI) m/z calcd. for $C_{16}H_{13}FN_2NaO_5$ $[M+Na]^+$: 355.0701, found: 355.0702. IR: 3016, 2970, 1741, 1656, 1558, 1494, 1217, 1075 cm^{-1}.

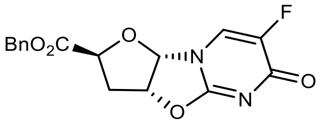

6f

Benzyl (1'*S*,2'*R*,4'*S*)-5-fluoro-4-oxo-1',2',3',4'-tetrahydro-6H-furo oxazolo pyrimidine-4'-carboxylate (6f). R_f = 0.44 (silica gel, PE:acetone = 1:2, UV); elution solvent is PE:acetone = 3:1, white solid (28 mg, 83% yield). MP: 186.2–187.5 °C. $[\alpha]_D^{20}$ = +11.37 (*c* = 0.20, acetone). ^{1}H NMR (400 MHz, CD_3OD) δ 8.08 (d, *J* = 4.0 Hz, 1H), 7.46–7.20 (m, 5H), 6.41 (d, *J* = 5.2 Hz, 1H), 5.69 (t, *J*

= 5.6 Hz, 1H), 5.22 (s, 2H), 4.82 (dd, *J* = 11.0, 5.6 Hz, 1H), 2.75 (dd, *J* = 14.6, 5.4 Hz, 1H), 2.42–2.35 (m, 1H). ^{13}C NMR (100 MHz, CD_3OD) δ 170.3, 167.3 (d, *J* = 18 Hz), 160.0, 147.4 (d, *J* = 249 Hz) 136.80, 129.6 (2), 129.6, 129.5 (2), 122.8, (d, *J* = 37 Hz), 93.4, 86.3, 77.5, 68.4, 37.0. HRMS (ESI) m/z calcd. for $C_{16}H_{13}FN_2NaO_5$ $[M+Na]^+$: 355.0701, found: 355.0700. IR: 3016, 2970, 1738, 1658, 1558, 1497, 1365, 1217, 1066 cm^{-1}.

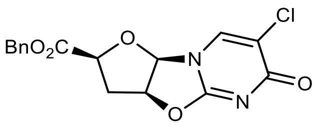

5g

Benzyl (1’*R*,2’*S*,4’*S*)-5-chloro-4-oxo-1’,2’,3’,4’-tetrahydro-6H-furo oxazolo pyrimidine-4’-carboxylate (5g). R_f = 0.35 (silica gel, PE:acetone = 1:2, UV); elution solvent is PE:acetone = 2:1, white solid (31 mg, 88% yield). MP: 193.5–195.1 °C. $[\alpha]_D^{20}$ = –66.58 (*c* = 0.20, acetone). ^{1}H NMR (400 MHz, CD_3OD) δ 8.23 (s, 1H), 7.41–7.24 (m, 5H), 6.40 (d, *J* = 5.2 Hz, 1H), 5.66–5.63 (m, 1H), 5.19 (d, *J* = 12.2 Hz, 1H), 5.06–5.01 (m, 1H), 4.92 (d, *J* = 12.2 Hz, 1H), 2.76–2.74 (m, 2H). ^{13}C NMR (100 MHz, CD_3OD) δ 172.7, 169.6, 161.6, 136.4, 136.1, 129.7 (2), 129.7, 129.6 (2), 118.3, 94.1, 86.3, 80.0, 68.6, 37.0. HRMS (ESI) m/z calcd. for $C_{16}H_{13}ClN_2NaO_5$ $[M+Na]^+$: 371.0405, found: 371.0401, 373.0411. IR: 2921, 1740, 1638, 1542, 1487, 1259, 1022, 797 cm^{-1}.

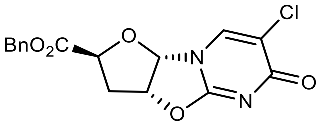

6g

Benzyl (1’*S*,2’*R*,4’*S*)-5-chloro-4-oxo-1’,2’,3’,4’-tetrahydro-6H-furo oxazolo pyrimidine-4’-carboxylate (6g). R_f = 0.37 (silica gel, PE:acetone = 1:2, UV); elution solvent is PE:acetone = 2:1, white solid (28 mg, 80% yield). MP: 213.4–213.9 °C. $[\alpha]_D^{20}$ = +61.37 (*c* = 0.20, acetone). ^{1}H NMR (400 MHz, $(CD_3)_2SO$) δ 8.42 (s, 1H), 7.41–7.33 (m, 5H), 6.35 (d, *J* = 5.2 Hz, 1H), 5.62 (t, *J* = 5.6 Hz, 1H), 5.24–5.15 (m, 2H), 4.93 (dd, *J* = 11.2, 5.6 Hz, 1H), 2.65 (dd, *J* = 14.4, 5.2 Hz, 1H), 2.34–2.26 (m, 1H). ^{13}C NMR (100 MHz, $(CD_3)_2SO$) δ 168.6, 165.6, 159.4, 135.5, 134.3, 128.5 (2), 128.2, 128.1 (2), 116.1, 90.9, 83.8, 75.5, 66.4, 35.4. HRMS (ESI) m/z calcd. for $C_{16}H_{13}ClN_2NaO_5$$[M+Na]^+$: 371.0405, found: 371.0401, 373.0411. IR: 3001, 2970, 1739, 1606, 1534, 1366, 1217, 1096, 790 cm^{-1}.

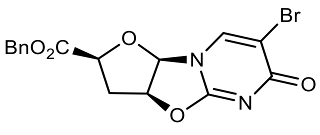

5h

Benzyl (1'*R*,2'*S*,4'*S*)-5-bromo-4-oxo-1',2',3',4'-tetrahydro-6H-furo oxazolo pyrimidine-4'-carboxylate (5h). R_f = 0.35 (silica gel, PE:acetone = 1:2, UV); elution solvent is PE:acetone = 2:1, white solid (35 mg, 90% yield). MP: 180.3–181.5 °C. $[\alpha]_D^{20}$ = –69.13 (*c* = 0.20, acetone). ^{1}H NMR (400 MHz, $(CD_3)_2CO$) δ 8.26 (s, 1H), 7.41–7.33 (m, 5H), 6.49 (d, *J* = 5.2 Hz, 1H), 5.73 (t, *J* = 5.4 Hz, 1H), 5.19 (d, *J* = 12.2 Hz, 1H), 5.07 (dd, *J* = 9.0, 1.6 Hz, 1H), 4.94 (d, *J* = 12.2 Hz, 1H), 2.82–2.72 (m, 2H). ^{13}C NMR (100 MHz, $(CD_3)_2CO$) δ 172.0, 166.5, 161.0, 137.0, 136.4, 129.4 (2), 129.3, 129.2 (2), 107.7, 93.2, 85.0, 79.3, 67.9, 37.0. HRMS (ESI) m/z calcd. for $C_{16}H_{13}BrN_2NaO_5$ $[M+Na]^+$: 414.9900, found: 414.9901, 416.9869. IR: 3469, 3016, 2970, 1738, 1434, 1365, 1217, 902 cm^{-1}.

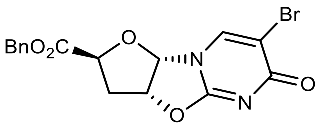

6h

Benzyl (1'*S*,2'*R*,4'*S*)-5-bromo-4-oxo-1',2',3',4'-tetrahydro-5H-furo oxazolo pyrimidine-4'-carboxylate (56h). R_f = 0.37 (silica gel, PE:acetone = 1:2, UV); elution solvent is PE:acetone = 2:1, white solid (32 mg, 82% yield). MP: 185.8–187.2 °C. $[\alpha]_D^{20}$ = +32.79 (*c* = 0.20, acetone). ^{1}H NMR (400 MHz, $(CD_3)_2SO$) δ 8.49 (s, 1H), 7.47–7.28 (m, 5H), 6.34 (d, *J* = 5.2 Hz, 1H), 5.62 (t, *J* = 5.8 Hz, 1H), 5.23–5.16 (m, 2H), 4.93 (dd, *J* = 11.2, 5.2 Hz, 1H), 2.64 (dd, *J* = 14.4, 5.4 Hz, 1H), 2.33–2.27 (m, 1H). ^{13}C NMR (100 MHz, $(CD_3)_2SO$) δ 168.7, 165.9, 159.8, 137.0, 135.5, 128.5 (2), 128.3, 128.1 (2), 106.2, 90.8, 83.9, 75.5, 66.4, 35.5. HRMS (ESI) m/z calcd. for $C_{16}H_{13}BrN_2NaO_5$ $[M+Na]^+$: 414.9900, found: 414.9901, 416.9871. IR: 3470, 3016, 2970, 1738, 1605, 1529, 1478, 1738, 1605, 1478, 1365, 1217, 1092 cm^{-1}.

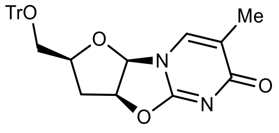

5i

(1'*R*,2'*S*,4'*S*)-5-methyl-4'-((trityloxy)methyl)-1',2',3',4'-tetrahydro-6H-furo oxazolo pyrimidin-6-one (5i). R_f = 0.34 (silica gel, PE:acetone = 1:1, UV); elution solvent is PE:acetone = 4:1, white solid (42 mg, 89% yield). MP: 218.0–219.2 °C. $[\alpha]_D^{20}$ = –36.75 (*c* = 0.40, acetone). ^{1}H NMR (400 MHz, $(CD_3)_2SO$) δ 7.82 (s, 1H), 7.37–7.15 (m, 15H), 6.20 (d, *J* = 5.6 Hz, 1H), 5.48 (t, *J* = 5.8 Hz, 1H), 4.58–4.52 (m, 1H), 2.86 (dd, *J* = 10.2, 4.0 Hz, 1H), 2.76 (dd, *J* = 10.2, 7.8 Hz, 1H), 2.49–2.40 (m, 1H), 2.13 (d, *J* = 15.0 Hz, 1H), 1.78 (s, 3H). ^{13}C NMR (100 MHz, $(CD_3)_2SO$) δ 171.5, 159.1, 143.4 (3), 132.3, 128.0 (6), 127.9 (6), 127.0 (3), 116.7, 91.0, 85.8, 83.6, 80.2, 65.7, 33.3, 13.6. HRMS (ESI) m/z calcd. for $C_{29}H_{26}N_2NaO_4$ $[M+Na]^+$: 489.1785, found: 489.1790. IR: 3498, 2917, 1644, 1555, 1487,

1261, 1077, 785, 706 cm^{-1}.

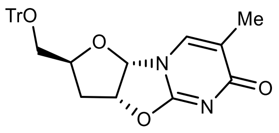

6i

(1’*S*,2’*R*,4’*S*)-5-methyl-4’-((trityloxy)methyl)-1’,2’,3’,4’-tetrahydro-6H-furo oxazolo pyrimidin-6-one (6i). R_f = 0.36 (silica gel, PE:acetone = 1:1, UV); elution solvent is PE:acetone = 4:1, white solid (43 mg, 93% yield). MP: 223.0–225.0 °C. $[\alpha]_D^{20}$ = +9.04 (*c* = 0.40, acetone). ^{1}H NMR (400 MHz, $CDCl_3$) δ 7.42 (s, 1H), 7.42–7.23 (m, 15H), 6.16 (d, *J* = 5.2 Hz, 1H), 5.44 (t, *J* = 5.6 Hz, 1H), 4.18–4.12 (m, 1H), 3.35 (dd, *J* = 10.4, 3.8 Hz, 1H), 3.24 (dd, *J* = 10.4, 4.8 Hz, 1H), 2.39 (dd, *J* = 14.4, 4.8 Hz, 1H), 2.13–2.05 (m, 1H), 1.99 (s, 3H). ^{13}C NMR (100 MHz, $CDCl_3$) δ 172.6, 160.3, 143.6 (3), 130.4, 128.7 (6), 128.1 (6), 127.4 (3), 119.6, 90.7, 87.1, 83.2, 77.9, 63.7, 35.2, 14.2. HRMS (ESI) m/z calcd. for $C_{29}H_{26}N_2NaO_4$ $[M+Na]^+$: 489.1785, found: 489.1781. IR: 3499, 2917, 1749, 1628, 1549, 1253, 1116, 1073, 1025, 797 cm^{-1}.

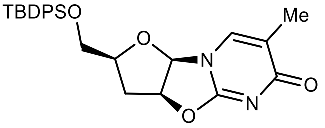

5j

(1’*R*,2’*S*,4’*S*)-5-methyl-4’-(((tert-butyldiphenylsilyl)oxy)methyl)-1’,2’,3’,4’-tetrahydro-6H-furo oxazolo pyrimidin-6-one (5j). R_f = 0.36 (silica gel, PE:acetone = 1:1, UV); elution solvent is PE:acetone = 4:1, white solid (40 mg, 87% yield). MP: 120.2–120.8 °C. $[\alpha]_D^{20}$ = –33.19 (*c* = 0.40, acetone). ^{1}H NMR (400 MHz, $CDCl_3$) δ 7.57–7.53 (m, 4H), 7.45–7.32 (m, 6H), 7.14 (s, 1H), 6.07 (d, *J* = 5.6 Hz, 1H), 5.45–5.41 (m, 1H), 4.49–4.39 (m, 1H), 3.52–3.37 (m, 2H), 2.51–2.39 (m, 2H), 1.88 (s, 3H), 0.99 (s, 9H). ^{13}C NMR (100 MHz, $CDCl_3$) δ 172.4, 159.5, 135.5 (2), 133.0, 132.8, 130.4, 130.1 (2), 130.1 (2), 128.0 (2), 127.9 (2), 119.2, 91.1, 83.6, 82.1, 65.4, 33.7, 26.8 (3), 19.3, 14.1. HRMS (ESI) m/z calcd. for $C_{26}H_{30}N_2NaO_4Si$ $[M+Na]^+$: 485.1867, found: 485.1872. IR: 3450, 2930, 2857, 1737, 1662, 1554, 1485, 1428, 1363, 1217, 1113, 1074, 703 cm^{-1}.

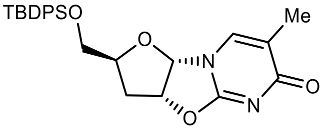

6j

(1’*S*,2’*R*,4’*S*)-5-methyl-4’-((trityloxy)methyl)-1’,2’,3’,4’-tetrahydro-6H-furo oxazolo pyrimidin-6-one (6i). R_f = 0.36 (silica gel, PE:acetone = 1:1, UV); elution solvent is PE:acetone = 4:1, white

solid (42 mg, 90% yield). MP: 128.4–129.1 °C. $[\alpha]_D^{20}$ = +33.17 (c = 0.40, acetone). ^{1}H NMR (400 MHz, $CDCl_3$) δ 7.65–7.62 (m, 4H), 7.49–7.34 (m, 6H), 7.19 (s, 1H), 6.09 (d, J = 5.2 Hz, 1H), 5.44 (t, J = 5.6 Hz, 1H), 4.19–4.10 (m, 1H), 3.87 (dd, J = 11.6, 3.6 Hz, 1H), 3.76 (dd, J = 11.6, 4.0 Hz, 1H), 2.39 (dd, J = 14.4, 4.8 Hz, 1H), 2.24–2.17 (m, 1H), 1.98 (s, 3H), 1.05 (s, 9H). ^{13}C NMR (100 MHz, $CDCl_3$) δ 172.7, 160.3, 135.7 (2), 135.6, 133.0, 133.0, 130.7 (2), 130.0 (2), 127.9 (2), 127.9 (2), 119.3, 90.8, 83.6, 79.2, 63.6, 34.4, 26.9 (3), 19.4, 14.1. HRMS (ESI) m/z calcd. for $C_{26}H_{30}N_2NaO_4Si$ $[M+Na]^+$: 485.1867, found: 485.1867. IR: 3450, 2930, 1663, 1555, 1466, 1428, 1289, 1132, 1111, 994, 703 cm^{-1}.

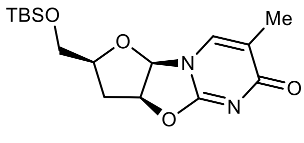

5k

(1’*R*,2’*S*,4’*S*)-5-methyl-4’-(((tert-butyldimethylsilyl)oxy)methyl)-1’,2’,3’,4’-tetrahydro-6H-furo oxazolo pyrimidin-6-one (5k). R_f = 0.35 (silica gel, PE:acetone = 1:1, UV); elution solvent is PE:acetone = 4:1, white solid (28 mg, 83% yield). MP: 151.2–152.3 °C. $[\alpha]_D^{20}$ = –35.52 (c = 0.40, acetone). ^{1}H NMR (400 MHz, $(CD_3)_2SO$) δ 7.75 (s, 1H), 6.18 (d, J = 5.4 Hz, 1H), 5.51 (t, J = 5.6 Hz, 1H), 4.40–4.32 (m, 1H), 3.42 (dd, J = 11.2, 4.8 Hz, 1H), 3.30 (d, J = 6.8 Hz, 1H), 2.48–2.42 (m, 1H), 2.19 (d, J = 14.8 Hz, 1H), 1.78 (s, 3H), 0.78 (s, 9H), –0.07 (s, 3H), –0.09 (s, 3H). ^{13}C NMR (100 MHz, $(CD_3)_2SO$) δ 171.6, 159.3, 132.3, 116.7, 90.9, 83.7, 81.6, 64.7, 32.7, 25.7, 18.0 (3), 13.5, –5.5, –5.6. HRMS (ESI) m/z calcd. for $C_{16}H_{26}N_2NaO_4Si$ $[M+Na]^+$: 361.1554, found: 361.1554. IR: 3016, 2970, 1738, 1663, 1486, 1365, 1217, 1091, 800 cm^{-1}.

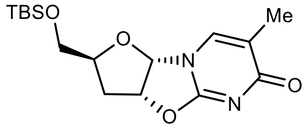

6k

(1’*S*,2’*R*,4’*S*)-5-methyl-4’-(((tert-butyldimethylsilyl)oxy)methyl)-1’,2’,3’,4’-tetrahydro-6H-furo oxazolo pyrimidin-6-one (6k). R_f = 0.37 (silica gel, PE:acetone = 1:1, UV); elution solvent is PE:acetone = 4:1, white solid (31 mg, 91% yield). MP: 158.5–160.9 °C. $[\alpha]_D^{20}$ = +59.18 (c = 0.40, acetone). ^{1}H NMR (400 MHz, $(CD_3)_2SO$) δ 7.75 (s, 1H), 6.24 (d, J = 5.2 Hz, 1H), 5.53 (t, J = 5.6 Hz, 1H), 4.06–4.00 (m, 1H), 3.80 (dd, J = 11.6, 3.0 Hz, 1H), 3.66 (dd, J = 11.6, 4.4 Hz, 1H), 2.25 (dd, J = 14.4, 4.6 Hz, 1H), 2.05–1.97 (m, 1H), 1.79 (s, 3H), 0.86 (s, 9H), 0.05 (s, 3H), 0.04 (s, 3H). ^{13}C NMR (100 MHz, $(CD_3)_2SO$) δ 171.6, 160.0, 132.4, 116.8, 90.4, 83.4, 78. 7, 62.8, 33.4, 25.8, 18.0 (3), 13.5, –5.3, –5.4. HRMS (ESI) m/z calcd. for $C_{16}H_{26}N_2NaO_4Si$ $[M+Na]^+$: 361.1554, found: 361.1553. IR: 3017, 2970, 1738, 1664, 1544, 1485, 1365, 1229, 1075, 785 cm^{-1}.

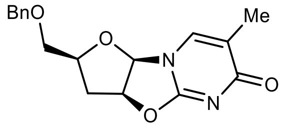

5l

(1’*R*,2’*S*,4’*S*)-5-methyl-4’-((benzyloxy)methyl)-1’,2’,3’,4’-tetrahydro-6H-furo oxazolo pyrimid-in-6-one (5l). R_f = 0.32 (silica gel, PE:acetone = 1:1, UV); elution solvent is PE:acetone = 4:1, white solid (24 mg, 76% yield). MP: 135.9–136.7°C. $[\alpha]_D^{20}$ = –9.62 (*c* = 0.20, acetone). ^{1}H NMR (400 MHz, $CDCl_3$) δ 7.38–7.22 (m, 5H), 7.21 (s, 1H), 6.10 (d, *J* = 5.6 Hz, 1H), 5.43 (t, *J* = 6.2 Hz, 1H), 4.57–4.52 (m, 1H), 4.46–4.30 (m, 2H), 3.34 (dd, *J* = 10.4, 4.2 Hz, 1H), 3.21 (dd, *J* = 10.4, 4.8 Hz, 1H), 2.57–2.35 (m, 2H), 1.99 (s, 3H). ^{13}C NMR (100 MHz, $CDCl_3$) δ 172.8, 160.1, 137.2, 130.4, 128.6 (2), 128.3 (2), 128.1, 119.0, 91.4, 83.4, 81.0, 73.7, 70.9, 34.4, 14.2. HRMS (ESI) m/z calcd. for $C_{10}H_{12}N_2NaO_4$ $[M+Na]^+$: 337.1159, found: 337.1163. IR: 3407, 2980, 1759, 1666, 1555, 1487, 1261, 1072, 785 cm^{-1}.

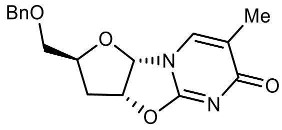

6l

(1’*S*,2’*R*,4’*S*)-5-methyl-4’-((benzyloxy)methyl)-1’,2’,3’,4’-tetrahydro-6H-furo oxazolo pyramid-in-6-one (6l). R_f = 0.34 (silica gel, PE:acetone = 1:1, UV); elution solvent is PE:acetone = 4:1, white solid (30 mg, 94% yield). MP: 138.0–139.1 °C. $[\alpha]_D^{20}$ = +40.29 (*c* = 0.20, acetone). ^{1}H NMR (400 MHz, $CDCl_3$) δ 7.36–7.29 (m, 5H), 7.20 (s, 1H), 6.15 (d, *J* = 4.4 Hz, 1H), 5.44 (t, *J* = 5.6 Hz, 1H), 4.56 (s, 2H), 4.30–4.17 (m, 1H), 3.70 (dd, *J* = 11.0, 2.8 Hz, 1H), 3.55 (dd, *J* = 11.0, 4.8 Hz, 1H), 2.38 (dd, *J* = 14.4, 4.6 Hz, 1H), 2.19–2.07 (m, 1H), 1.95 (s, 3H). ^{13}C NMR (100 MHz, $CDCl_3$) δ 172.5, 160.2, 137.5, 130.6, 128.6 (2), 128.1 (2), 127.9, 119.3, 90.8, 83.3, 78.0, 73.7, 69.5, 34.6, 14.2. HRMS (ESI) m/z calcd. for $C_{10}H_{12}N_2NaO_4$ $[M+Na]^+$: 337.1159, found: 337.1161. IR: 3001, 2919, 1757, 1660, 1627, 1558, 1480, 1490, 1087, 919 cm^{-1}.

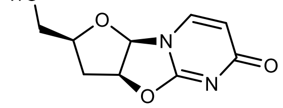

5m

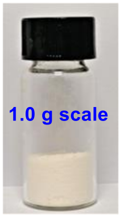

(1’*R*,2’*S*,4’*S*)-4’-((trityloxy)methyl)-1’,2’,3’,4’-tetrahydro-6H-furo oxazolo pyrimidin-6-one (5m). R_f = 0.25 (silica gel, PE:acetone = 1:1, UV); elution solvent is PE:acetone = 4:1, white solid (43 mg, 94% yield of 0.1 mmol scale; 916 mg, 92% yield of 1.0 g scale). MP: 101.5–102.6 °C. $[\alpha]_D^{20}$ = –

33.11 (c = 0.20, acetone). ^{1}H NMR (400 MHz, (CD_3OD) δ 7.78 (d, J = 7.4 Hz, 1H), 7.33–7.18 (m, 15H), 6.21 (d, J = 5.6 Hz, 1H), 5.96 (d, J = 7.4 Hz, 1H), 5.50 (t, J = 6.4 Hz, 1H), 4.67–4.61 (m, 1H), 2.96 (dd, J = 10.2, 4.6 Hz, 1H), 2.85 (dd, J = 10.2, 7.4 Hz, 1H), 2.51 (dt, J = 15.4, 7.8 Hz, 1H), 2.28–2.25 (m, 1H). ^{13}C NMR (100 MHz, (CD_3OD) δ 175.2, 161.7, 144.9 (3), 138.6, 129.6 (6), 128.9 (6), 128.3 (3), 110.1, 93.0, 87.9, 86.4, 82.4, 66.67, 34.6. HRMS (ESI) m/z calcd. for $C_{28}H_{24}N_2NaO_4$ $[M+Na]^+$: 475.1634, found: 475.1639. IR: 3488, 2924, 1647, 1555, 1460, 1265, 1154, 785 cm^{-1}.

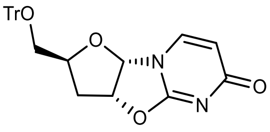

6m

(1'*S*,2'*R*,4'*S*)-4'-((trityloxy)methyl)-1',2',3',4'-tetrahydro-6H-furo oxazolo pyrimidin-6-one (6m). R_f = 0.28 (silica gel, PE:acetone = 1:1, UV); elution solvent is PE:acetone = 4:1, white solid (42 mg, 93% yield). MP: 264.0–265.2 °C. $[\alpha]_D^{20}$ = +15.25 (c = 0.20, acetone). ^{1}H NMR (400 MHz, $(CD_3)_2SO$) δ 7.90 (d, J = 7.4 Hz, 1H), 7.39–7.25 (m, 15H), 6.30 (d, J = 5.2 Hz, 1H), 5.87 (d, J = 7.4 Hz, 1H), 5.56–5.53 (m, 1H), 4.21–4.15 (m, 1H), 3.19 (dd, J = 10.4, 3.2 Hz, 1H), 3.09 (dd, J = 10.4, 5.2 Hz, 1H), 2.31 (dd, J = 14.2, 4.4 Hz, 1H), 2.07–1.99 (m, 1H). ^{13}C NMR (100 MHz, $(CD_3)_2SO$) δ 171.1, 160.3, 143.5 (3), 137.0, 128.2 (6), 127.9 (6), 127.1 (3), 108.8, 90.2, 86.0, 83.6, 77.1, 63.8, 34.3. HRMS (ESI) m/z calcd. for $C_{28}H_{24}N_2NaO_4$ $[M+Na]^+$: 475.1634, found: 475.1638. IR: 3500, 2853, 1643, 1471, 1253, 1075, 1021, 797, 756cm^{-1}.

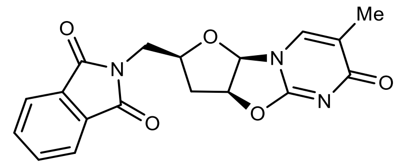

5n

(1'*R*,2'*S*,4'*S*)-5-methyl-4'-(isoindoline-1,3-dione)-1',2',3',4'-tetrahydro-6H-furo oxazolo pyrimidin-6-one (5n). R_f = 0.40 (silica gel, PE:acetone = 1:1, UV); elution solvent is PE:acetone = 4:1, white solid (32 mg, 90% yield). MP: 180.3–181.2 °C. $[\alpha]_D^{20}$ = +128.26 (c = 0.20, MeOH). ^{1}H NMR (400 MHz, CD_3OD) δ 7.79 (s, 4H), 7.59 (s, 1H), 6.21 (d, J = 5.2 Hz, 1H), 5.60 (t, J = 5.6 Hz, 1H), 4.8–4.77 (m, 1H), 3.65 (d, J = 6.0 Hz, 2H), 2.71–2.52 (m, 2H), 1.98 (s, 3H). ^{13}C NMR (100 MHz, CD_3OD) δ 175.7 (2), 169.3, 161.6, 135.5 (2), 134.6, 133.1 (2), 124.2 (2), 119.5, 93.5, 86.6, 80.6, 43.1, 35.1, 13.9. HRMS (ESI) m/z calcd. for $C_{18}H_{15}N_3NaO_5$ $[M+Na]^+$: 376.0910, found: 376.0908. IR: 3455, 3016, 2970, 1736, 1434, 1365, 1217, 1091 cm^{-1}.

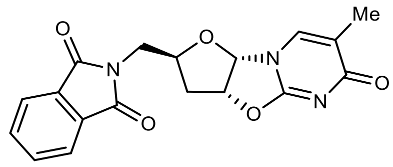

6n

(1’*S*,2’*R*,4’*S*)-5-methyl-4’-(isoindoline-1,3-dione)-1’,2’,3’,4’-tetrahydro-6H-furo oxazolo pyrimidin-6-one (6n). R_f = 0.42 (silica gel, PE:acetone = 1:1, UV); elution solvent is PE:acetone = 4:1, white solid (33 mg, 92% yield). MP: 259.1–260.2 °C. $[\alpha]_D^{20}$ = –0.46 (*c* = 0.20, MeOH). ^{1}H NMR (400 MHz, $(CD_3)_2SO$) δ 7.89–7.83 (m, 4H), 7.73 (s, 1H), 6.22 (d, *J* = 5.2 Hz, 1H), 5.49 (t, *J* = 5.4 Hz, 1H), 4.25–4.18 (m, 1H), 3.88–3.75 (m, 2H), 2.45 (dd, *J* = 14.4, 4.4 Hz, 1H), 2.01–1.93 (m, 1H), 1.76 (s, 3H). ^{13}C NMR (100 MHz, $(CD_3)_2SO$) δ 171.6 (2), 167.7, 160.0, 134.5 (2), 132.4, 131.6 (2), 123.1 (2), 116.8, 90.3, 83.4, 79.2, 75.7, 36.0, 13.5. HRMS (ESI) m/z calcd. for $C_{18}H_{15}N_3NaO_5$ $[M+Na]^+$: 376.0910, found: 376.0909. IR: 3455, 3016, 2970, 1736, 1718, 1550, 1484, 1365, 1217, 1060 cm^{-1}.

Derivatizations of β-nucleoside 7.

Synthesis of compound 8.

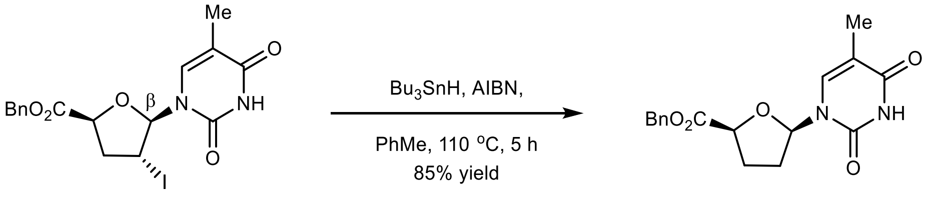

7 **8**

General procedure V:

Under an atmosphere of argon, compound **7** (228 mg, 0.5 mmol) and azodiisobutyronitrile (AIBN, 62 mg, 0.4 mmol) was dissolved in anhydrous PhMe (5 mL). After adding Bu_3SnH (218 mg, 0.8 mmol), the reaction mixture was warmed up to 110 °C and stirred for 5h. The solution was quenched with H_2O (2 mL) and diluted with EtOAc (2 mL), then filtered. The layers were separated, and the aqueous layer was extracted with EtOAc (4 mL x 4). The combined organic layer was washed with brine (4 mL x 4), dried over Na_2SO_4 and filtered, concentrated in vacuo. The crude material was purified via silica gel column chromatography to obtain **8**.

Benzyl (1’*R*,4’*S*)-1’-(5-methyl-2,4-dioxo-3,4-dihydropyrimidin-1(2H)-yl)tetrahydrofuran-4’-carboxylate (8). R_f = 0.30 (silica gel, PE:EtOAc = 1:1, UV); elution solvent is PE:EtOAc = 4:1, white solid (141 mg, 85% yield). MP: 127.1–128.0 °C. $[\alpha]_D^{20}$ = +14.28 (*c* = 0.20, acetone). ^{1}H NMR (400 MHz, CD_3OD) δ 8.07 (d, *J* = 1.0 Hz, 1H), 7.50–7.21 (m, 5H), 6.13 (t, *J* = 5.6 Hz, 1H), 5.24 (s, 2H), 4.66 (dd, *J* = 8.0, 5.6 Hz, 1H), 2.48–2.32 (m, 2H), 2.16–1. 98 (m, 2H), 1.78 (d, *J* = 1.0 Hz, 3H). ^{13}C NMR (100 MHz, CD_3OD) δ 173.9, 166.4, 152.3, 138.2, 137.0, 129.7 (2), 129.6, 129.4 (2), 111.1, 88.7,

79.0, 68.2, 32.1, 29.9, 12.6. HRMS (ESI) m/z calcd. for $C_{17}H_{18}N_2NaO_5$ $[M+Na]^+$: 353.1108, found: 353.1110. IR: 3016, 2970, 1738, 1466, 1365, 1229, 1216, 756 cm^{-1}.

Synthesis of β-D-ddT (9).

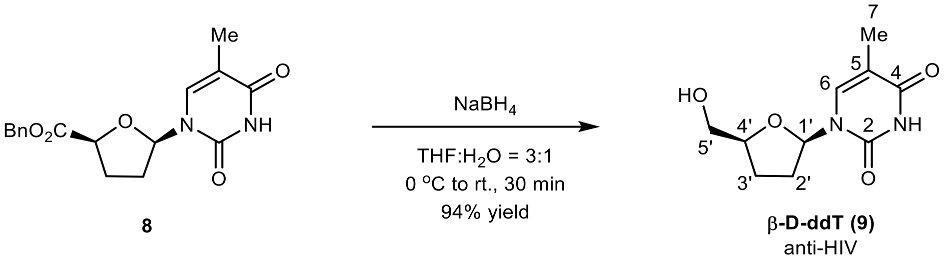

General procedure VI:

To a solution of THF (1.5 mL) and H_2O (0.5 mL) a mixture of **8** (99 mg, 0.3 mmol) and $NaBH_4$ (23 mg, 0.6 mmol) was added at 0 °C for 30 min. After that, the solution was quenched with saturated NH_4Cl aq. (1 mL), the THF was removed under vacuo and the aqueous layer was extracted with $CHCl_3$ (3 mL x 3). The combined organic layer was washed with brine (3 mL x 4) and dried over Na_2SO_4 and filtered, concentrated in vacuo. The crude material was purified via silica gel column chromatography to obtain **9**.

((1'*R*,4'*S*)-4'-(hydroxymethyl)tetrahydrofuran-1'-yl)-5-methylpyrimidine-2,4(1H,3H)-dione (9). R_f = 0.32 (silica gel, PE:Acetone = 1:3, UV); elution solvent is CH_2Cl_2:MeOH = 10:1, white solid (64 mg, 94% yield). MP: 154.8–155.4 °C; lit.[1] MP: 154–156 °C. $[\alpha]_D^{25}$ = +29.50 (*c* = 0.60, MeOH); lit.[2] $[\alpha]_D^{25}$ = +27.00 (*c* = 0.62, MeOH). ^1H NMR (400 MHz, CD_3OD) δ 7.92 (s, 1H), 6.05 (dd, *J* = 7.0, 3.6 Hz, 1H), 4.15–4.10 (m, 1H), 3.88 (dd, *J* = 12.2, 3.0 Hz, 1H), 3.68 (dd, *J* = 12.2, 3.8 Hz, 1H), 2.44–1.94 (m, 4H), 1.88 (s, 3H). ^{13}C NMR (100 MHz, CD_3OD) δ 166.6, 152.4, 138.4, 110.8, 87.3, 83.2, 63.7, 33.3, 25.9, 12.5. ^1H NMR (400 MHz, $(CD_3)_2SO$) δ 11.20 (br, 1H), 7.79 (d, *J* = 1.2 Hz, 1H), 5.96 (dd, *J* = 7.0, 3.6 Hz, 1H), 5.01 (t, *J* = 5.4 Hz, 1H), 4.03–3.97 (m, 1H), 3.70–3.65 (m, 1H), 3.56–3.50 (m, 1H), 2.30–2.17 (m, 1H), 1.99–1.83 (m, 3H), 1.77 (d, *J* = 1.2 Hz, 3H). ^{13}C NMR (100 MHz, $(CD_3)_2SO$) δ 163.8, 150.4, 136.3, 108.7, 84.7, 81.2, 62.1, 31.5, 24.9, 12.2. HRMS (ESI) m/z calcd. for $C_{10}H_{14}N_2NaO_4$ $[M+Na]^+$: d 249.0846, found: 249.0848. IR: 3411, 2929, 1682, 1476, 1460, 1270, 1178, 1079, 771 cm^{-1}.

Synthesis of compound 10.

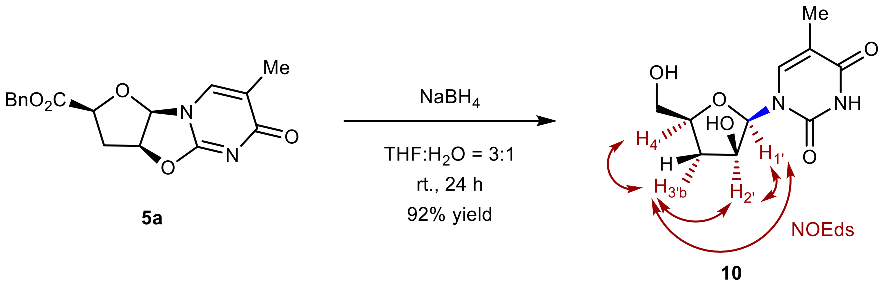

The procedure to obtain **10** refers to **General procedure VI** expect for changing the stirring time to about 24 h at room temperature.

((1'*R*,2'*S*,4'*S*)-2'-hydroxy-4'-(hydroxymethyl)tetrahydrofuran-1'-yl)-5-methylpyrimidine-2,4(1H,3H)-dione (10). R_f = 0.40 (silica gel, CH_2Cl_2:MeOH = 10:1, UV); elution solvent is CH_2Cl_2:MeOH =20:1, white solid (223 mg, 92% yield). MP: 112.2–113.4 °C. $[\alpha]_D^{20}$ = +33.42 (*c* = 0.20, MeOH). ^{1}H NMR (400 MHz, CD_3OD) δ 7.79 (d, *J* = 1.2 Hz, 1H), 5.94 (d, *J* = 4.4 Hz, 1H), 4.45–4.42 (m, 1H), 4.21–4.15 (m, 1H), 3.82 (dd, *J* = 12.0, 3.2 Hz, 1H), 3.70 (dd, *J* = 12.0, 4.4 Hz, 1H), 2.43–2.36 (m, 1H), 1.95–1.90 (m, 1H), 1.88 (d, *J* = 1.2 Hz, 3H). ^{13}C NMR (100 MHz, CD_3OD) δ 167.0, 152.8, 139.9, 109.5, 88.2, 79.3, 71.3, 64.3, 35.3, 12.5. HRMS (ESI) m/z calcd. for $C_{10}H_{14}N_2NaO_5$ $[M+Na]^+$: 265.0795, found: 265.0792. IR: 3456, 3016, 2970, 1738, 1439, 1365, 1217, 1091, 900 cm^{-1}.

Synthesis of compound 11.

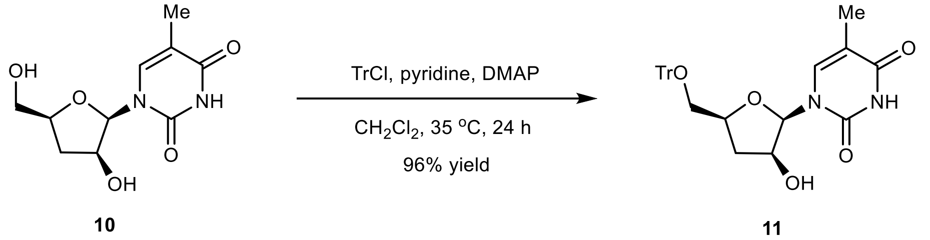

General procedure VII:

Under an atmosphere of argon, a mixture of **10** (194 mg, 0.8 mmol), DMAP (10 mg, 0.08 mmol) and pyridine (95 mg, 1.2 mmol) was dissolved in anhydrous CH_2Cl_2 (5 mL) and stirred at 0 °C for 10 min. After adding TrCl (279 mg, 1.0 mmol), the reaction mixture was warmed up to 35 °C and stirred for 24 h. The solution was diluted with $CHCl_3$ (5 mL) and saturated NH_4Cl aq. (5 mL), then filtered. The layers were separated and the aqueous layer was extracted with $CHCl_3$ (5 mL x 4). The combined organic layer was washed with brine (5 mL x 4), dried over Na_2SO_4 and filtered, concentrated in vacuo. The crude material was purified via silica gel column chromatography to obtain **11**.

((1'*R*,2'*S*,4'*S*)-2'-hydroxy-4'-((trityloxy)methyl)tetrahydrofuran-2-yl)-5-methylpyrimidine-2,4(1H,3H)-dione (11). R_f = 0.40 (silica gel, PE:acetone = 3:1, UV); elution solvent is PE:EtOAc =

10:1, white solid (372 mg, 96% yiel). MP: 215.2–216.1 °C. $[\alpha]_D^{20}$ = +50.29 (*c* = 0.30, $CHCl_3$). ^{1}H NMR (400 MHz, CD_3OD) δ 7.66 (s, 1H), 7.49–7.24 (m, 15H), 6.02 (d, *J* = 5.0 Hz, 1H), 4.51 (q, *J* = 5.6 Hz, 1H), 4.23–4.17 (m, 1H), 3.41 –3.33 (m, 2H), 2.35 (dt, *J* = 13.6, 7.0 Hz, 1H), 1.99 (ddd, *J* = 13.6, 8.0, 5.8 Hz, 1H), 1.60 (s, 3H). ^{13}C NMR (100 MHz, CD_3OD) δ 166.6, 152.5, 145.2 (3), 139.7, 130.0 (6), 128.9 (6), 128.3 (3), 88.1, 87.7, 77.7, 71.6, 66.2, 35.8, 12.5. HRMS (ESI) m/z calcd. for $C_{29}H_{28}N_2NaO_5$ $[M+Na]^+$: 507.1896, found: 507.1892. IR: 3450, 3002, 2928, 1702, 1452, 1259, 1075, 750 cm^{-1}.

Synthesis of compound 12.

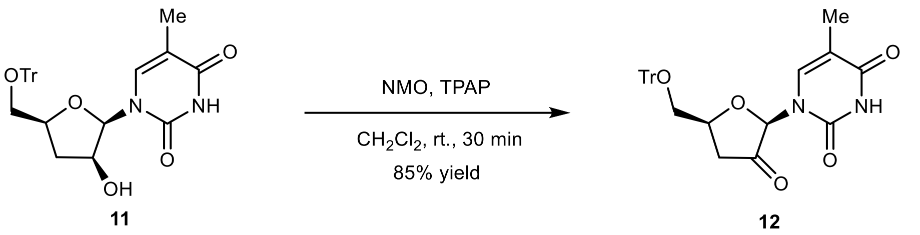

A mixture of compound **11** (387 mg, 0.8 mmol), *N*-methylmorpholine *N*-oxide (NMO, 184 mg, 1.6 mmol) and tetrapropylammonium perruthenate (TPAP, 28 mg, 0.08 mmol) was dissolved in anhydrous CH_2Cl_2 (5 mL) and stirred at room temperature for 30 min. The solution was quenched with H_2O (1 mL) and diluted with $CHCl_3$ (1 mL), then filtered. The layers were separated, and the aqueous layer was extracted with $CHCl_3$ (5 mL x 4). The combined organic layer was washed with brine (5 mL x 4), dried over Na_2SO_4 and filtered, concentrated in vacuo. The crude material was purified via silica gel column chromatography to obtain **12**.

((1'*R*,4'*S*)-2'-oxo-4'-((trityloxy)methyl)tetrahydrofuran-2-yl)-5-methylpyrimidine-2,4(1H,3H)-dione (12). R_f = 0.45 (silica gel, PE:acetone = 2:1, UV); elution solvent is PE:EtOAc = 8:1, white solid (328 mg, 85% yiel). MP: 103.6–104.7 °C. $[\alpha]_D^{20}$ = –0.43 (*c* = 0.20, acetone). ^{1}H NMR (400 MHz, $CDCl_3$) δ 8.28 (s, 1H), 7.46–7.24 (m, 15H), 7.03 (s, 1H), 5.38 (s, 1H), 4.58–4.52 (m, 1H), 3.48 (dd, *J* = 10.4, 5.4 Hz, 1H), 3.41 (dd, *J* = 10.4, 3.6 Hz, 1H), 2.86 (dd, *J* = 18.8, 8.0 Hz, 1H), 2.61 (dd, *J* = 18.8, 7.6 Hz, 1H), 1.77 (s, 3H). ^{13}C NMR (100 MHz, $CDCl_3$) δ 206.7, 164.0, 150.4, 143.6 (3), 138.7, 128.8 (6), 128.0 (6), 127.3 (3), 111.9, 87.1, 85.9, 75.2, 65.6, 36.8, 12.1. HRMS (ESI) m/z calcd. for $C_{29}H_{26}N_2NaO_5$ $[M+Na]^+$: 505.1734, found: 505.1735. IR: 3356, 3016, 2970, 1738, 1449, 1366, 1261, 1216, 1064, 703 cm^{-1}.

Synthesis of compound 13.

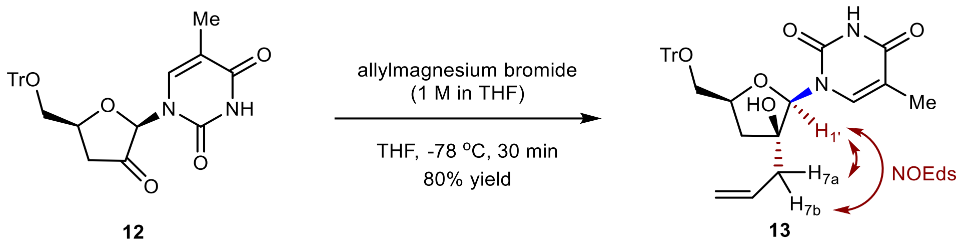

Under an atmosphere of argon, **12** (48 mg, 0.1 mmol) was dissolved in anhydrous THF (1 mL), then the reaction mixture was cooled to –78 °C. After that, allylmagnesium bromide (1 M in THF, 0.12 mmol) was added dropwise via syringe and stirred for 30 min. The solution was diluted with EtOAc (2 mL) and saturated NH_4Cl aq. (2 mL), then filtered. The layers were separated, and the aqueous layer was extracted with EtOAc (2 mL x 4). The combined organic layer was washed with brine (2 mL x 4), dried over Na_2SO_4 and filtered, concentrated in vacuo. The crude material was purified via silica gel column chromatography to obtain **13**.

((1'*R*,2'*S*,4'*S*)-2'-allyl-2'-hydroxy-4'-((trityloxy)methyl)tetrahydrofuran-1'-yl)-5-methylpyrimidine-2,4(1H,3H)-dione (13). R_f = 0.25 (silica gel, PE:acetone = 2:1, UV); elution solvent is PE:EtOAc = 6:1, white solid (42 mg, 80% yield). MP: 90.2–90.8 °C. $[\alpha]_D^{20}$ = +15.54 (*c* = 0.20, acetone). ^{1}H NMR (400 MHz, $CDCl_3$) δ 8.62 (br, 1H), 7.73 (s, 1H), 7.47–7.26 (m, 15H), 5.98–5.88 (m, 1H), 5.86 (s, 1H), 5.22–5.14 (m, 2H), 4.26–4.23 (m, 1H), 3.62 (dd, *J* = 10.8, 2.6 Hz, 1H), 3.56 (br, 1H), 3.23 (dd, *J* = 10.8, 3.4 Hz, 1H), 2.51 (dd, *J* = 14.2, 6.8 Hz, 1H), 2.36 (dd, *J* = 14.2, 7.8 Hz, 1H), 2.22 (dd, *J* = 13.8, 8.4 Hz, 1H), 1.95 (dd, *J* = 13.8, 6.4 Hz, 1H), 1.72 (s, 3H). ^{13}C NMR (100 MHz, $CDCl_3$) δ 164.1, 151.3, 143.3 (3), 137.8, 132.6, 128.8 (6), 128.2 (6), 127.6 (3), 119.8, 109.4, 89.1, 88.1, 79.5, 76.0, 65.2, 41.6, 38.2, 12.5. HRMS (ESI) m/z calcd. for $C_{32}H_{32}N_2NaO_5$ $[M+Na]^+$: 547.2203, found: 547.2199. IR: 3459, 3016, 2970, 2986, 1738, 1435, 1365, 1217, 1091, 901, 738 cm^{-1}.

Synthesis of compound 14.

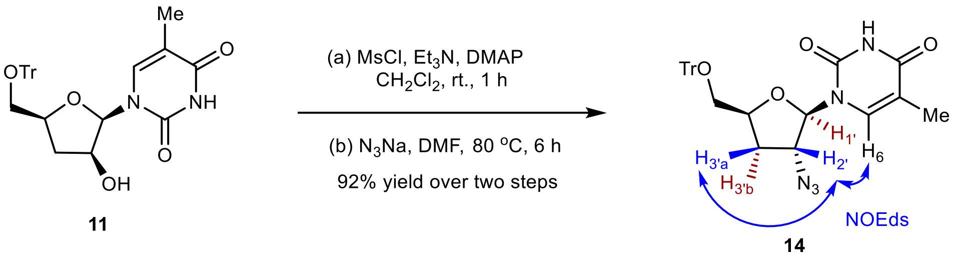

Under an atmosphere of argon, a mixture of **11** (150 mg, 0.3 mmol), DMAP (4 mg, 0.03 mmol) and Et_3N (52 μL, 0.4 mmol) was dissolved in anhydrous CH_2Cl_2 (3 mL) and stirred at room temperature for 10 min. After adding MsCl (29 μL, 0.4 mmol), the reaction mixture was stirred for 1

h. The solution was diluted with CH_2Cl_2 (5 mL) and saturated NH_4Cl aq. (5 mL), then filtered. The layers were separated, and the aqueous layer was extracted with CH_2Cl_2 (5 mL x 4). The combined organic layer was washed with brine (5 mL x 4), dried over Na_2SO_4 and filtered, concentrated in vacuo. The crude material was purified via silica gel column chromatography to obtain the protected intermediate.

Under an atmosphere of argon, a mixture of the protected intermediate (170 mg, 0.3 mmol) and N_3Na (98 mg, 1.5 mmol) was dissolved in anhydrous DMF (3 mL) and stirred at 80 °C for 6 h. The solution was diluted with EtOAc (2 mL) and saturated NH_4Cl aq. (2 mL), then filtered. The layers were separated, and the aqueous layer was extracted with EtOAc (4 mL x 4). The combined organic layer was washed with brine (4 mL x 4), dried over Na_2SO_4 and filtered, concentrated in vacuo. The crude material was purified via silica gel column chromatography to obtain **14**.

((1’*R*,2’*R*,4’*S*)-2’-azide-4’-((trityloxy)methyl)tetrahydrofuran-2-yl)-5-methylpyrimidine-2,4(1H,3H)-dione (14). R_f = 0.50 (silica gel, PE: EtOAc = 1:1, UV); elution solvent is PE:EtOAc = 5:1, white solid (145 mg, 92% yiel over two steps). MP: 164.4–165.1 °C. $[\alpha]_D^{20}$ = –25.90 (*c* = 0.58, $CHCl_3$). ^1H NMR (400 MHz, $CDCl_3$) δ 8.90 (br, 1H), 7.65 (s, 1H), 7.43–7.25 (m, 15H), 5.86 (d, *J* = 1.6 Hz, 1H), 4.48–4.42 (m, 1H), 4.35 (d, *J* = 6.2 Hz, 1H), 3.58 (dd, *J* = 11.0, 2.4 Hz, 1H), 3.32 (dd, *J* = 11.0, 3.4 Hz, 1H), 2.34 (ddd, *J* = 13.4, 10.2, 6.2 Hz, 1H), 1.97 (ddd, *J* = 13.4, 5.4, 2.2 Hz, 1H), 1.46 (s, 3H). ^{13}C NMR (100 MHz, $CDCl_3$) δ 164.0, 150.3, 143.4 (3), 135.0, 128.7 (6), 128.2 (6), 127.6 (3), 110.8, 90.6, 87.4, 80.5, 66.5, 63.4, 31.2, 12.2. HRMS (ESI) m/z calcd. for $C_{29}H_{27}N_5NaO_4$ $[M+Na]^+$: 532.1961, found: 532.1958. IR: 3005, 2980, 1746, 1505, 1275, 1261, 764 cm^{-1}.

Synthesis of compound 15.

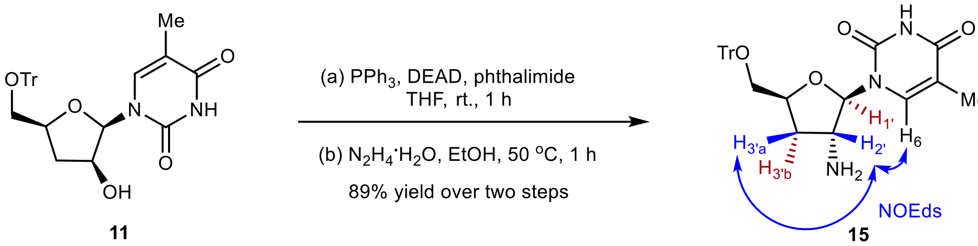

11 15

Under an atmosphere of argon, a mixture of **11** (100 mg, 0.2 mmol), PPh_3 (108 mg, 0.4 mmol) and diethyl azodicarboxylate (DEAD, 75 μL, 0.5 mmol) was dissolved in anhydrous THF (5 mL) and stirred at room temperature for 10 min. After adding phthalimide (76 mg, 0.5 mmol), the reaction mixture was stirred for 1 h. The solution was diluted with EtOAc (10 mL) and saturated NH_4Cl aq. (5 mL), then filtered. The layers were separated and the aqueous layer was extracted with EtOAc (5 mL x 4). The combined organic layer was washed with brine (5 mL x 4), dried over Na_2SO_4 and filtered, concentrated in vacuo. The crude material was purified via silica gel column chromatography to obtain

the intermediate.

Under an atmosphere of argon, a mixture of the intermediate (115 mg, 0.2 mmol) and $N_2H_4 \cdot H_2O$ (19 μL, 1.5 mmol) was dissolved in EtOH (5 mL) and stirred at 50 °C for 1 h. The solution was diluted with CH_2Cl_2 (5 mL), then filtered. The layers were separated, and the aqueous layer was extracted with CH_2Cl_2 (4 mL x 4). The combined organic layer was washed with brine (4 mL x 4), dried over Na_2SO_4 and filtered, concentrated in vacuo. The crude material was purified via silica gel column chromatography to obtain **15**.

((1’*R*,2’*R*,4’*S*)-2’-amido-4’-((trityloxy)methyl)tetrahydrofuran-2-yl)-5-methylpyrimidine-2,4(1H,3H)-dione (15). R_f = 0.30 (silica gel, $CHCl_3$: EtOH = 15:1, UV); elution solvent is $CHCl_3$: EtOH = 25:1, white solid (89 mg, 89% yiel over two steps). MP: 88.1–88.9 °C. $[\alpha]_D^{20}$ = –10.37 (*c* = 0.54, $CHCl_3$). ^{1}H NMR (400 MHz, $CDCl_3$) δ 7.70 (s, 1H), 7.46–7.24 (m, 15H), 5.73 (d, *J* = 4.2 Hz, 1H), 4.51–4.47 (m, 1H), 3.73–3.69 (m, 1H), 3.42 (dd, *J* = 10.6, 2.4 Hz, 1H), 3.27 (dd, *J* = 10.6, 3.8 Hz, 1H), 2.40 (dt, *J* = 13.0, 7.4 Hz, 1H), 1.95 (ddd, *J* = 12.8, 7.2, 5.4 Hz, 1H), 1.42 (s, 3H). ^{13}C NMR (100 MHz, $CDCl_3$) δ 166.4, 152.7, 145.0 (3), 137.5, 129.9 (6), 129.0 (6), 128.4 (3), 111.23, 93.3, 88.5, 80.0, 66.2, 58.3, 35.1, 12.2. HRMS (ESI) m/z calcd. for $C_{29}H_{29}N_3NaO_4$ $[M+Na]^+$: 506.2056, found: 506.2057. IR: 2926, 2902, 1725, 1465, 1276, 1261, 1074, 764, 750 cm^{-1}.

Synthesis of stavudine.

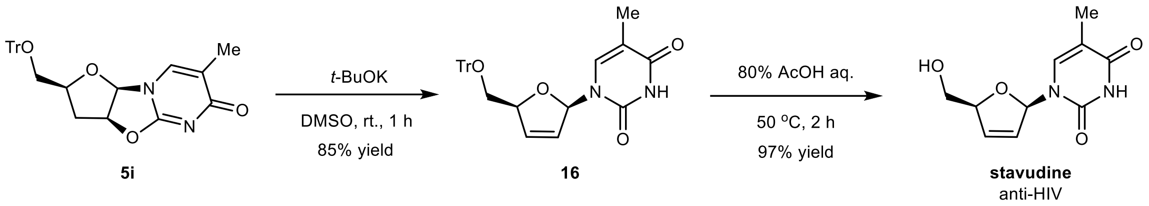

General procedure VIII:

Under an atmosphere of argon, a mixture of the Tr protected intermediate (326 mg, 0.7 mmol) and *t*-BuOK (314 mg, 2.8 mmol) was dissolved in anhydrous dimethyl sulfoxide (DMSO, 3 mL) and stirred at room temperature for 1 h. The solution was diluted with EtOAc (2 mL) and saturated NH_4Cl aq. (2 mL), then filtered. The layers were separated, and the aqueous layer was extracted with EtOAc (4 mL x 4). The combined organic layer was washed with brine (4 mL x 4), dried over Na_2SO_4 and filtered, concentrated in vacuo. The crude material was purified via silica gel column chromatography to obtain **16**.

General procedure IX:

To a round bottom flask **16** (94 mg, 0.2 mmol) in AcOH (1.6 mL) and H_2O (0.4 mL) was added and stirred at 50 °C for 2 h. The AcOH was removed under vacuo. The crude material was purified via silica gel column chromatography to obtain stavudine.

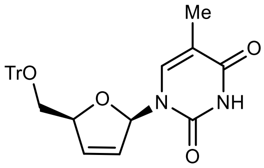

16

((1’*R*,4’*S*)-4’-((trityloxy)methyl)-1’,4’-dihydrofuran-1’-yl)-5-methylpyrimidine-2,4(1H,3H)-dione (16). R_f = 0.50 (silica gel, PE:acetone = 2:1, UV); elution solvent is PE:EtOAc = 2:1, white solid (277 mg, 85% yield). MP: 108.6–109.6 °C. $[\alpha]_D^{20}$ = –2.72 (*c* = 0.20, acetone). ^{1}H NMR (400 MHz, $(CD_3)_2SO$) δ 11.35 (br, 1H), 7.37–7.24 (m, 15H), 7.22 (s, 1H), 6.85 (d, *J* = 1.8 Hz, 1H), 6.52 (dd, *J* = 6.0, 1.8 Hz, 1H), 6.01 (dd, *J* = 6.0, 2.0 Hz, 1H), 4.96 (s, 1H), 3.25–3.14 (m, 2H), 1.24 (s, 3H). ^{13}C NMR (100 MHz, $(CD_3)_2SO$) δ 163.7, 150.7, 143.3 (3), 135.7, 134.4, 128.3 (6), 127.9 (6), 127.1(3), 126.0, 109.5, 89.1, 86.1, 85.1, 65.2, 11.3. HRMS (ESI) m/z calcd. for $C_{29}H_{26}N_2NaO_4$ $[M+Na]^+$: 489.1785, found: 489.1782. IR: 3016, 2970, 1739, 1440, 1365, 1228, 1217, 1010, 739 cm^{-1}.

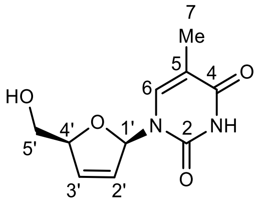

stavudine
anti-HIV

((1’*R*,4’*S*)-4’-(hydroxymethyl)-1’,4’-dihydrofuran-1’-yl)-5-methylpyrimidine-2,4(1H,3H)-dione (stavudine). R_f = 0.30 (silica gel, CH_2Cl_2:MeOH = 10:1, UV); elution solvent is CH_2Cl_2:MeOH = 10:1, white solid (44 mg, 97% yield). MP: 164.5–165.3 °C; lit.[4] MP: 164–165 °C. $[\alpha]_D^{20}$ = –32.35 (*c* = 0.70, H_2O); lit.[4] $[\alpha]_D^{20}$ = –32.50 (*c* = 0.70, H_2O). ^{1}H NMR (400 MHz, CD_3OD) δ 7.75 (d, *J* = 1.2 Hz, 1H), 6.96–6.94 (m, 1H), 6.40 (dt, *J* = 6.0, 1.8 Hz, 1H), 5.90 (dt, *J* = 6.0, 1.8 Hz, 1H), 4.87–4.85 (m, 1H), 3.81–3.72 (m, 2H), 1.84 (d, *J* = 1.2 Hz, 3H). ^{13}C NMR (100 MHz, CD_3OD) δ 166.6, 152.9, 138.9, 135.9, 127.3, 111.2, 91.1, 89.0, 63.8, 12.4. HRMS (ESI) m/z calcd. for $C_{10}H_{12}N_2NaO_4$ $[M+Na]^+$: 247.0689, found: 247.0690. IR: 3397, 3016, 2926, 1687, 1472, 1400, 1253, 1074, 974, 779 cm^{-1}.

Synthesis of 5-methyluridine (17).

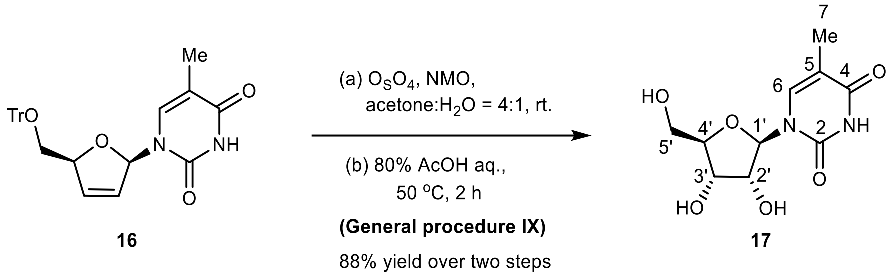

16 17

General procedure X:

Adding OsO_4 (1% in water, 50 μL) to the reaction mixture of **16** (94 mg, 0.2 mmol) and NMO (46 mg, 0.4 mmol) in acetone (2.4 mL) and H_2O (0.6 mL). The reaction mixture was stirred for 32 h at room temperature. The solution was quenched with $Na_2S_2O_3$ (4 mL) and diluted with $CHCl_3$ (4 mL). The layers were separated, and the aqueous layer was extracted with $CHCl_3$ (2 mL x 4). The combined organic layer was washed with brine (4 mL x 4), dried over Na_2SO_4 and filtered, concentrated in vacuo. **((1'*R*,2'*R*,3'*S*,4'*R*)-2',3'-dihydroxy-4'-(hydroxymethyl)tetrahydrofuran-1'-yl)-5-methylpyrimidine-2,4(1H,3H)-dione (17).** R_f = 0.30 (silica gel, CH_2Cl_2:MeOH = 5:1, UV); elution solvent is CH_2Cl_2:MeOH = 10:1, white solid (44 mg, 88% yield over two steps). MP: 181.9–183.0 °C; lit [5] MP: 180−182 °C. $[\alpha]_D^{22}$ = –13.06 (*c* = 0.43, MeOH); lit [6] $[\alpha]_D^{22}$ = –12.90 (*c* = 0.43, MeOH). ^1H NMR (400 MHz, D_2O) δ 7.61 (d, *J* = 1.6 Hz, 1H), 5.83 (d, *J* = 4.6 Hz, 1H), 4.26 (t, *J* = 5.2 Hz, 1H), 4.16 (t, *J* = 5.4 Hz, 1H), 4.07–4.00 (m, 1H), 3.83 (dd, *J* = 12.8, 3.0 Hz, 1H), 3.73 (dd, *J* = 12.8, 4.2 Hz, 1H), 1.81 (s, 3H). ^{13}C NMR (100 MHz, D_2O) δ 166.6, 151.9, 137.5, 111.6, 89.1, 84.2, 73.6, 69.5, 60.8, 11.6. HRMS (ESI) m/z calcd. for $C_{10}H_{12}N_2NaO_6$ $[M+Na]^+$: 281.0744, found: 281.0743. IR: 3456, 3016, 2970, 1738, 1445, 1385, 1217, 1092, 995, 759 cm^{-1}.

Synthesis of molnupiravir.

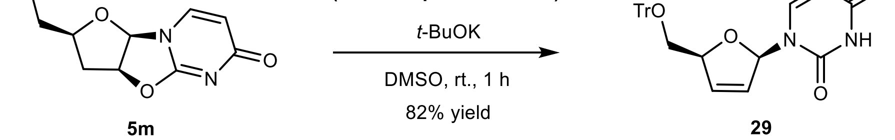

5m 29

((1'*R*,4'*S*)-4'-((trityloxy)methyl)-1',4'-dihydrofuran-1'-yl)-2,4(1H,3H)-dio-ne (29). R_f = 0.40 (silica gel, PE:acetone = 2:1, UV); elution solvent is PE:EtOAc = 2:1, white solid (410 mg, 82% yield). MP: 192.5–193.6 °C. $[\alpha]_D^{20}$ = –30.10 (*c* = 0.20, $CHCl_3$). ^1H NMR (400 MHz, $CDCl_3$) δ 8.78 (br, 1H), 7.79 (d, *J* = 8.2 Hz, 1H), 7.37–7.24 (m, 15H), 7.02 (s, 1H), 6.34 (d, *J* = 5.8 Hz, 1H), 5.88 (d, *J* = 5.8 Hz, 1H), 5.03 (dd, *J* = 8.2, 2.2 Hz, 1H), 4.95 (s, 1H), 3.49 (dd, *J* = 10.8, 3.4 Hz, 1H), 3.42 (dd, *J* =

10.8, 2.8 Hz, 1H). ^{13}C NMR (100 MHz, $CDCl_3$) δ 163.3, 150.7, 143.2 (3), 141.4, 134.6, 128.9 (6), 128.1 (6), 127.6 (3), 126.5, 102.4, 89.8, 87.6, 86.1, 64.6. HRMS (ESI) m/z calcd. for $C_{28}H_{24}N_2NaO_4$ $[M+Na]^+$: 475.1634, found: 475.1638. IR: 3057, 2885, 2864, 1688, 1448, 1367, 1246, 1033, 760, 702 cm^{-1}.

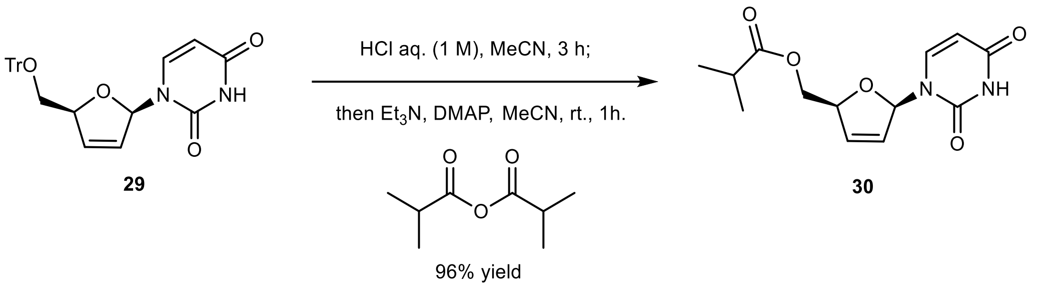

Compound **29** (350 mg, 0.8 mmol) was dissolved in MeCN (8 mL) with addition HCl aq. (2 M, 1 mL) The mixture was stirred at room temperature for 3 h. Then the reaction mixture was dried over Na_2SO_4 and filtered, concentrated in vacuo. Under an atmosphere of argon, a mixture of intermediate, DMAP (9 mg, 0.08 mmol) and Et_3N (129 μL, 0.9 mmol) was dissolved in anhydrous MeCN (5 mL) and stirred at room temperature for 10 min. After adding isobutyric anhydride (147 mg, 0.9 mmol), the reaction mixture and was stirred for 1 h. The solution was diluted with $CHCl_3$ (5 mL) and saturated NH_4Cl aq. (5 mL), then filtered. The layers were separated, and the aqueous layer was extracted with $CHCl_3$ (5 mL x 4). The combined organic layer was washed with brine (5 mL x 4), dried over Na_2SO_4 and filtered, concentrated in vacuo. The crude material was purified via silica gel column chromatography to obtain **30**.

((1'*R*,4'*S*)-1'-(2,4-dioxo-3,4-dihydropyrimidin-1(2H)-yl)-2',3'-dihydrofuran-4'-yl)methyl iso-Butyrate. (30). R_f = 0.35 (silica gel, PE:acetone = 2:1, UV); elution solvent is PE:EtOAc = 2:1, white solid (238 mg, 96% yield). MP: 138.3–138.9 °C. $[\alpha]_D^{20}$ = –43.40 (*c* = 0.20, $CHCl_3$). ^1H NMR (400 MHz, $CDCl_3$) δ 8.94 (br, 1H), 7.49 (d, *J* = 8.2 Hz, 1H), 6.98 (dd, *J* = 3.8, 2.0 Hz, 1H), 6.28 (d, *J* = 6.0 Hz, 1H), 5.92 (d, *J* = 6.0 Hz, 1H), 5.72 (d, *J* = 8.2 Hz, 1H), 5.08–5.05 (m, 1H), 4.42 (dd, *J* = 12.4, 3.8 Hz, 1H), 4.21 (dd, *J* = 12.4, 3.0 Hz, 1H), 2.53 (hept, *J* = 7.0 Hz, 1H), 1.18 (s, 3H), 1.16 (s, 3H). ^{13}C NMR (100 MHz, $CDCl_3$) δ 176.8, 163.1, 150.7, 139.9, 133.5, 127.3, 102.8, 90.2, 84.7, 64.6, 34.1, 19.1 (2). HRMS (ESI) m/z calcd. for $C_{13}H_{16}N_2NaO_5$ $[M+Na]^+$: 303.0957, found: 303.0953. IR: 3050, 2976, 2804, 1687, 1462, 1247, 1080, 851, 765 cm^{-1}.

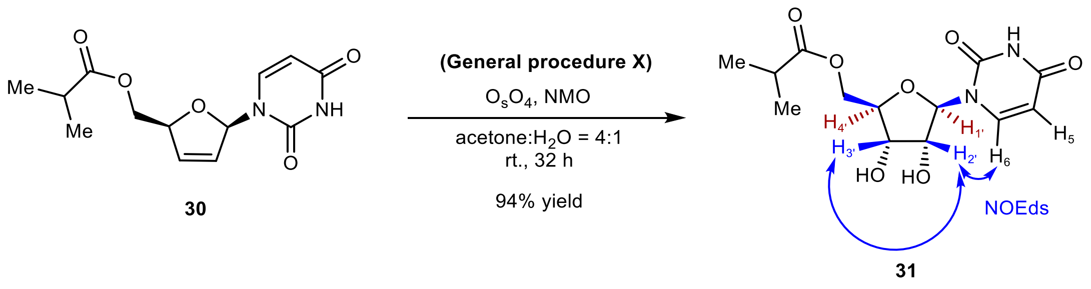

((1’*R*,2’*R*,3’*S*,4’*R*)-1’-(2,4-dioxo-3,4-dihydropyrimidin-1(2H)-yl)-2’,3’-dihydroxytetrahydro-furan-4’-yl)methyl isobutyrate (31). R_f = 0.15 (silica gel, PE:acetone = 1:1, UV); elution solvent is PE: acetone = 3:1, white solid (158 mg, 94% yield). MP: 161.5–162.4 °C. $[\alpha]_D^{20}$ = 18.00 (*c* = 0.20, MeOH). ^{1}H NMR (400 MHz, CD_3OD) δ 7.69 (d, *J* = 8.2 Hz, 1H), 5.83 (d, *J* = 4.0 Hz, 1H), 5.72 (d, *J* = 8.2 Hz, 1H), 4.38–4.30 (m, 2H), 4.21–4.18 (m, 1H), 4.16–4.14 (m, 1H), 4.12–4.09 (m, 1H), 2.62 (hept, *J* = 7.0 Hz, 1H), 1.19 (s, 3H), 1.18 (s, 3H). ^{13}C NMR (100 MHz, CD_3OD) δ 178.2, 166.2, 152.3, 142.3, 102.8, 91.7, 82.9, 75.2, 71.2, 64.6, 35.2, 19.4, 19.3. HRMS (ESI) m/z calcd. for $C_{13}H_{18}N_2NaO_7$ $[M+Na]^+$: 337.1012, found: 337.1006. IR: 3385, 2980, 1687, 1468, 1269, 1107, 814 cm^{-1}.

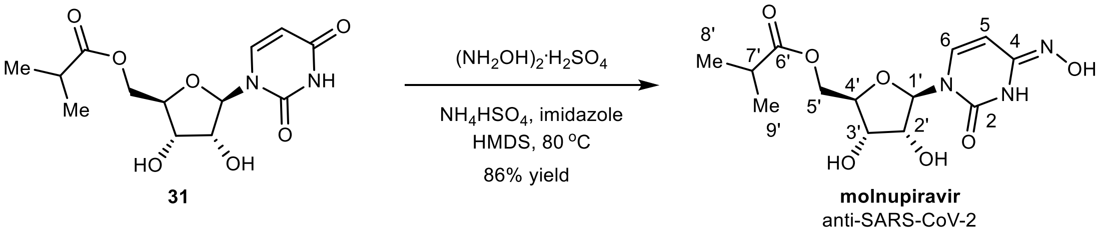

Under an atmosphere of argon, a mixture of imidazole (4 mg, 0.05 mmol), ammonium hydrogen sulfate (35 mg, 0.3 mmol) and hexamethyldisilazane (HMDS, 0.2 ml, 1.0 mmol) was stirred at 75 °C for 1 h. Adding hydroxylamine sulfate (33 mg, 0.2 mmol) and **31** (31 mg, 0.1 mmol) to the reaction mixture with stirring at 80 °C for 6 h and the mixture was cooled to room temperature. The solution was diluted with MeOH (1 mL), then filtered, concentrated in vacuo. The crude material was purified via silica gel column chromatography to obtain molnupiravir.

((1’*R*,2’*R*,3’*S*,4’*R*)-2’,3’-dihydroxy-1’-((*Z*)-4-(hydroxyimino)-2-oxo-3,4-dihydropyrimidin-(2H)-yl)tetrahydrofuran-4’-yl)methyl isobutyrate (molnupiravir). R_f = 0.40 (silica gel, CH_2Cl_2:MeOH = 6:1, UV); elution solvent is CH_2Cl_2:MeOH = 20:1, white solid (28 mg, 86% yield). MP: 159.8–160.5 °C. $[\alpha]_D^{20}$ = –6.00 (*c* = 0.20, MeOH). ^{1}H NMR (400 MHz, CD_3OD) δ 6.90 (d, *J* = 8.2 Hz, 1H), 5.81 (d, *J* = 4.8 Hz, 1H), 5.61 (d, *J* = 8.2 Hz, 1H), 4.29 (d, *J* = 3.4 Hz, 2H), 4.13 (t, *J* = 4.8 Hz, 1H), 4.10–4.06 (m, 2H), 2.62 (hept, *J* = 7.0 Hz, 1H), 1.19 (s, 3H), 1.17 (s, 3H). ^{13}C NMR (100 MHz, CD_3OD) δ 178.3, 151.5, 146.2, 131.8, 99.6, 90.5, 82.6, 74.4, 71.5, 64.9, 35.2, 19.4, 19.3. HRMS (ESI) m/z calcd. for $C_{13}H_{20}N_3O_7$ $[M+H]^+$: 330.1301, found: 330.1305. IR: 3390, 3160, 2975, 1682, 1406, 1270, 1113, 957 cm^{-1}.

Derivatizations of α-nucleoside 19.

Synthesis of compound 20.

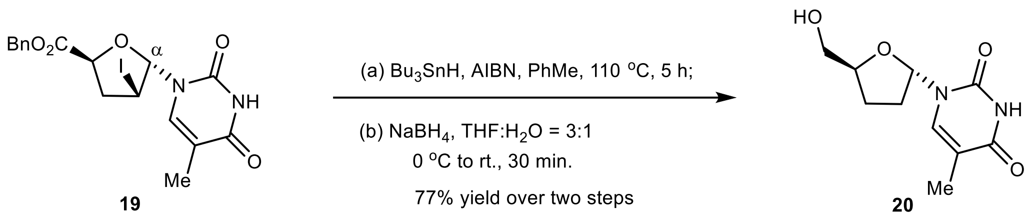

The procedure to obtain **20** refers to **General procedure V** and **General procedure VI.**

((1’*S*,4’*S*)-4’-(hydroxymethyl)tetrahydrofuran-1’-yl)-5-methylpyrimidine-2,4(1H,3H)-dione(20). R_f = 0.34 (silica gel, PE:Acetone = 1:3, UV); elution solvent is CH_2Cl_2:MeOH = 10:1, white solid (35 mg, 77% yield over two steps). MP: 152.3–152.9 °C. $[\alpha]_D^{20}$ = –10.42 (*c* = 0.20, MeOH). ^{1}H NMR (400 MHz, CD_3OD) δ 7.46 (d, *J* = 1.2 Hz, 1H), 6.08 (dd, *J* = 6.4, 4.8 Hz, 1H), 4.51–4.42 (m, 1H), 3.64 (dd, *J* = 11.8, 3.8 Hz, 1H), 3.53 (dd, *J* = 11.8, 5.2 Hz, 1H), 2.49–2.39 (m, 1H), 2.15–2.03 (m, 2H), 1.94–1.86 (m, 1H), 1.90 (d, *J* = 1.2 Hz, 3H). ^{13}C NMR (100 MHz, CD_3OD) δ 166.6, 152.3, 137.8, 111.2, 88.6, 83.2, 65.2, 33.2, 27.0, 12.4. HRMS (ESI) m/z calcd. for $C_{10}H_{14}N_2NaO_4$ $[M+Na]^+$: 249.0846, found: 249.0848. IR: 3455, 3016, 2970, 1738, 1435, 1366, 1217, 1092, 896 cm^{-1}.

Synthesis of compound 21.

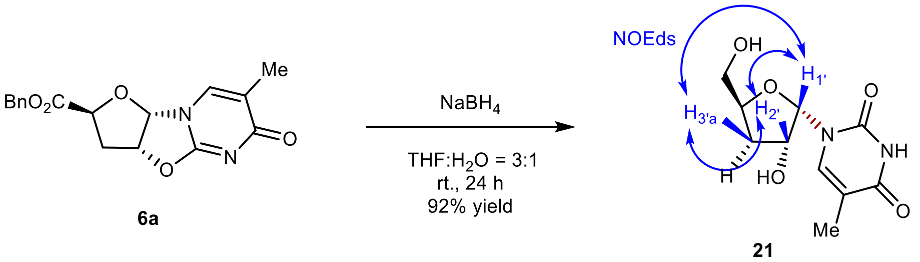

The procedure to obtain **21** refers to **General procedure VI** expect for changing the stirring time to about 24 h at room temperature.

((1’*S*,2’*R*,4’*S*)-2’-hydroxy-4’-(hydroxymethyl)tetrahydrofuran-1’-yl)-5-methylpyrimidine-2,4(1H,3H)-dione (21). R_f = 0.42 (silica gel, CH_2Cl_2:MeOH = 10:1, UV); elution solvent is CH_2Cl_2:MeOH =20:1, white solid (22 mg, 92% yield). MP: 186.2–188.0 °C. $[\alpha]_D^{20}$ = –134.92 (*c* = 0.20, MeOH). ^{1}H NMR (400 MHz, CD_3OD) δ 7.47 (d, *J* = 1.2 Hz, 1H), 6.02 (d, *J* = 3.6 Hz, 1H), 4.58–4.52 (m, 1H), 4.51–4.46 (m, 1H), 3.74 (dd, *J* = 12.0, 3.2 Hz, 1H), 3.54 (dd, *J* = 12.0, 4.8 Hz, 1H), 2.17–2.00 (m, 2H), 1.88 (d, *J* = 1.2 Hz, 3H). ^{13}C NMR (100 MHz, CD_3OD) δ 166.7, 152.4, 139.8, 109.4, 89.5, 81.2, 72.1, 64.9, 36.2, 12.5. HRMS (ESI) m/z calcd. for $C_{10}H_{14}N_2NaO_5$ $[M+Na]^+$: 265.0795, found: 265.0799. IR: 3353, 1668, 1487, 1360, 1276, 1160, 1091, 977 cm^{-1}.

Synthesis of compound 22.

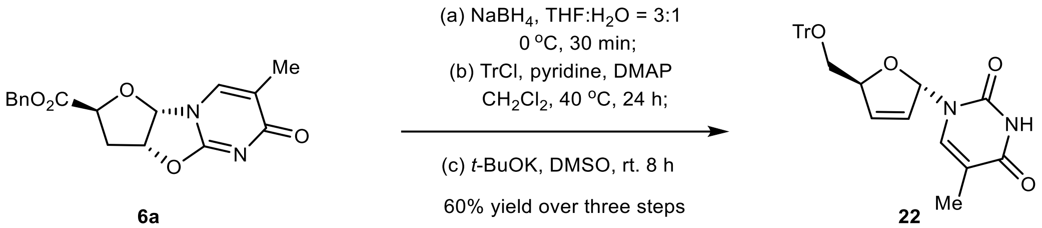

6a **22**

The procedure to obtain **22** refers to **General procedure VI** expect for changing the reaction temperature at 0 °C, **General procedure VII** and **General procedure VIII**.

((1’*S*,4’*S*)-4’-((trityloxy)methyl)-1’,4’-dihydrofuran-1’-yl)-5-methylpyrimidine-2,4(1H,3H)-dione (22). R_f = 0.52 (silica gel, PE:acetone = 2:1, UV); elution solvent is PE:EtOAc = 2:1, white solid (140 mg, 60% yield over three steps). MP: 103.6–104.5 °C. $[\alpha]_D^{20}$ = –95.66 (*c* = 0.20, acetone). ^{1}H NMR (400 MHz, $CDCl_3$) δ 8.71 (s, 1H), 7.48–7.23 (m, 15H), 7.10 (d, *J* = 5.2 Hz, 1H), 6.90 (d, *J* = 1.2 Hz, 1H), 6.36 (d, *J* = 6.0 Hz, 1H), 5.90–5.88 (m, 1H), 5.21–5.16 (m, 1H), 3.28–3.20 (m, 2H), 1.91 (d, *J* = 1.2 Hz, 3H). ^{13}C NMR (100 MHz, $CDCl_3$) δ 163.8, 150.6, 143.8 (3), 135.4, 135.0, 128.8 (6), 128.0 (6), 127.3 (3), 126.4, 111.5, 90.5, 86.9, 86.4, 65.8, 12.7. HRMS (ESI) m/z calcd. for $C_{29}H_{26}N_2NaO_4$ $[M+Na]^+$: 489.1785, found: 489.1783. IR: 3016, 2970, 1738, 1435, 1365, 1217, 1095, 909 cm^{-1}.

Synthesis of compound 23.

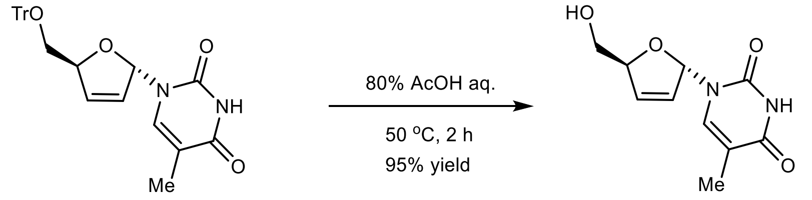

22 **23**

The procedure to obtain **23** refers to **General operation IX**.

((1’*S*,4’*S*)-4’-(hydroxymethyl)-1’,4’-dihydrofuran-1’-yl)-5-methylpyrimidine-2,4(1H,3H)-dione (23). R_f = 0.32 (silica gel, CH_2Cl_2:MeOH = 10:1, UV); elution solvent is CH_2Cl_2:MeOH = 10:1, white solid (21 mg, 95% yield). MP: 113.3–114.1 °C. $[\alpha]_D^{20}$ = –148.58 (*c* = 0.20, MeOH). ^{1}H NMR (400 MHz, $(CD_3)_2SO$) δ 11.27 (br, 1H), 7.12 (s, 1H), 6.86 (d, *J* = 5.2 Hz, 1H), 6.42 (d, *J* = 6.0 Hz, 1H), 5.92 (d, *J* = 5.8 Hz, 1H), 5.08–5.04 (m, 1H), 4.84 (br, 1H), 3.51–3.43 (m, 2H), 1.76 (s, 3H). ^{13}C NMR (100 MHz, $(CD_3)_2SO$) δ 163.9, 150.7, 135.8, 135.1, 125.7, 109.8, 89.5, 87.7, 63.2, 12.1. HRMS (ESI) m/z calcd. for $C_{10}H_{12}N_2NaO_4$ $[M+Na]^+$: 247.0689, found: 247.0692. IR: 3456, 3016, 2970, 1736, 1435, 1365, 1217, 1092, 996 cm^{-1}.

Synthesis of compounds 24 and 25.

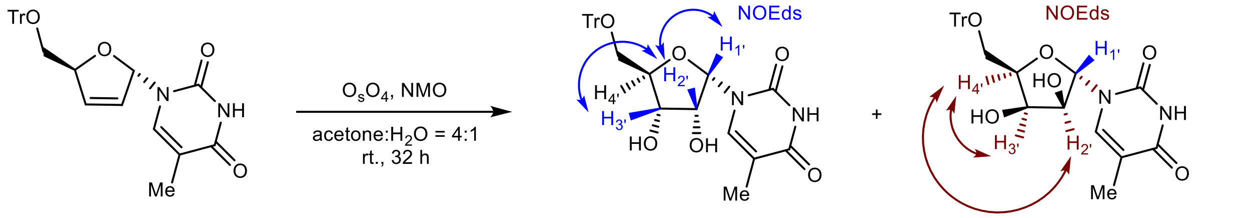

22 **24** (62% yield) **25** (30% yield)

The procedure to obtain **24** and **25** refers to **General procedure X.**

((1'*S*,2'*R*,3'*S*,4'*S*)-2',3'-dihydroxy-4'-((trityloxy)methyl)tetrahydrofuran-1'-yl)-5-methylpyrimidine-2,4(1H,3H)-dione (24). R_f = 0.30 (silica gel, PE:acetone = 2:1, UV); elution solvent is PE:EtOAc = 4:1, white solid (62 mg, 62% yield). MP: 130.1–130.9 °C. $[\alpha]_D^{20}$ = –1.96 (*c* = 0.20, acetone). ^{1}H NMR (400 MHz, CD_3OD) δ 7.60 (d, *J* = 1.2 Hz, 1H), 7.48–7.23 (m, 15H), 6.26 (d, *J* = 4.4 Hz, 1H), 4.43 (t, *J* = 4.2 Hz, 1H), 4.30–4.26 (m, 2H), 3.44 (dd, *J* = 10.4, 2.2 Hz, 1H), 3.17 (dd, *J* = 10.4, 3.4 Hz, 1H), 1.90 (d, *J* = 1.2 Hz, 3H). ^{13}C NMR (100 MHz, CD_3OD) δ 166.7, 152.7, 145.2 (3), 140.2, 129.9 (6), 128.9 (6), 128.2 (3), 109.6, 88.2, 87.6, 84.3, 72.7, 72.3, 65.0, 12.5. HRMS (ESI) m/z calcd. for $C_{29}H_{28}N_2NaO_6$ $[M+Na]^+$: 523.1845, found: 523.1841. IR: 3510, 3016, 2970, 1738, 1450, 1365, 1217, 1053, 1033, 773 cm^{-1}.

((1'*S*,2'*S*,3'*R*,4'*S*)-2',3'-dihydroxy-4'-((trityloxy)methyl)tetrahydrofuran-1'-yl)-5-methylpyrimidine-2,4(1H,3H)-dione (25). R_f = 0.26 (silica gel, PE:acetone = 2:1, UV); elution solvent is PE:EtOAc = 4:1, white solid (30 mg, 30% yield). MP: 126.0–127.0 °C. $[\alpha]_D^{20}$ = –4.91 (*c* = 0.20, acetone). ^{1}H NMR (400 MHz, CD_3OD) δ 7.52 (d, *J* = 1.2 Hz, 1H), 7.48–7.22 (m, 15H), 5.86 (d, *J* = 7.2 Hz, 1H), 4.64–4.60 (m, 1H), 4.49 (dd, *J* = 7.2, 4.6 Hz, 1H), 4.19 (dd, *J* = 4.6, 3.6 Hz, 1H), 3.42 (dd, *J* = 10.0, 7.2 Hz, 1H), 3.33 (dd, *J* = 10.0, 7.2 Hz, 1H), 1.93 (d, *J* = 1.2 Hz, 3H). ^{13}C NMR (100 MHz, CD_3OD) δ 166.5, 152.8, 145.4 (3), 138.9, 129.9 (6), 128.8 (6), 128.1 (3), 111.9, 91.3, 88.1, 82.9, 76.3, 72.8, 64.3, 12.4. HRMS (ESI) m/z calcd. for $C_{29}H_{28}N_2NaO_6$ $[M+Na]^+$: 523.1845, found: 523.1847. IR: 3505, 3010, 2970, 1738, 1445, 1365, 1217, 1057, 1033, 1010, 774 cm^{-1}.

1.3 Crystallographic Data

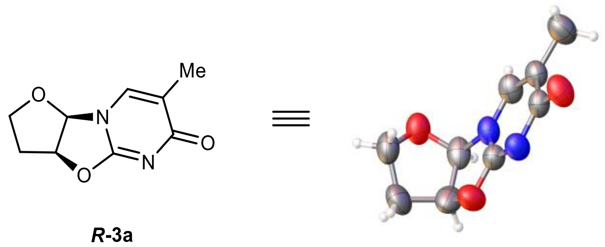

***R*-3a**

X-ray of ***R*-3a**
CCDC No. 2131198

Supplementary Figure 1. X-ray of ***R*-3a.**

Supplementary Table 1. Crystallographic data of compound ***R*-3a.**

Empirical formula	$C_9H_{10}N_2O_3$
Formula weight	194.19
Temperature/K	283.0
Crystal system	monoclinic
Space group	$P2_1$
a/Å	5.3943(3)
b/Å	9.5065(6)
c/Å	9.0535(5)
α/°	90
β/°	98.037(4)
γ/°	90
Volume/Å^3	459.71(5)
Z	2
ρcalcg/cm^3	1.403
μ/mm^{-1}	0.902
F(000)	204.0
Crystal size/mm^3	0.35 × 0.17 × 0.16
Radiation	CuKα (λ = 1.54178)
2Θ range for data collection/°	9.866 to 136.422
Index ranges	-6 ≤ h ≤ 5, -11 ≤ k ≤ 11, -10 ≤ l ≤ 10
Reflections collected	4619
Independent reflections	1656 [R_{int} = 0.0470, R_{sigma} = 0.0480]
Data/restraints/parameters	1656/1/128
Goodness-of-fit on F^2	1.079
Final R indexes [I>=2σ (I)]	R_1 = 0.0404, wR_2 = 0.1076
Final R indexes [all data]	R_1 = 0.0421, wR_2 = 0.1103
Largest diff. peak/hole / e Å^{-3}	0.13/-0.17
Flack parameter	0.04(15)

These data can be obtained free of charge from The Cambridge Crystallographic Data Centre via https://www.ccdc.cam.ac.uk/mystructures/structuredetails/164e3c40-0367-ec11-96a5-00505695281c.

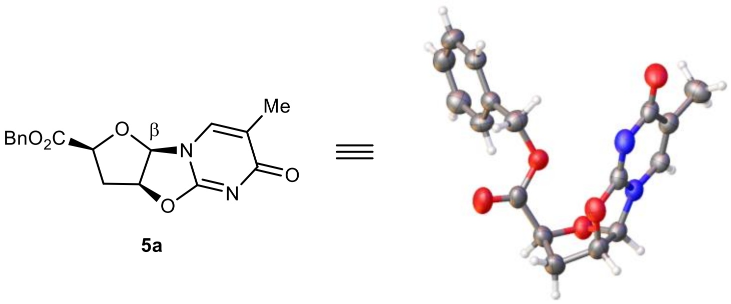

5a

X-ray of **5a**
CCDC No. 2131196

Supplementary Figure 2. X-ray of **5a.**

Supplementary Table 2. Crystallographic data of compound **5a.**

Empirical formula	$C_{17}H_{16}N_2O_5$
Formula weight	328.32
Temperature/K	302.0
Crystal system	monoclinic
Space group	$P2_1$
a/Å	9.8638(2)
b/Å	6.10250(10)
c/Å	13.7753(3)
α/°	90
β/°	107.1550(10)
γ/°	90
Volume/Å^3	792.30(3)
Z	2
ρ_{calc}g/cm^3	1.376
μ/mm^{-1}	0.859
F(000)	344.0
Crystal size/mm^3	0.43 × 0.12 × 0.1
Radiation	CuKα (λ = 1.54178)
2Θ range for data collection/°	6.716 to 134.142
Index ranges	-11 ≤ h ≤ 11, -7 ≤ k ≤ 6, -16 ≤ l ≤ 16
Reflections collected	13279
Independent reflections	2797 [R_{int} = 0.0308, R_{sigma} = 0.0243]
Data/restraints/parameters	2797/1/219
Goodness-of-fit on F^2	1.096
Final R indexes [I>=2σ (I)]	R_1 = 0.0239, wR_2 = 0.0625
Final R indexes [all data]	R_1 = 0.0242, wR_2 = 0.0627
Largest diff. peak/hole / e Å^{-3}	0.12/-0.11
Flack parameter	0.07(5)

These data can be obtained free of charge from The Cambridge Crystallographic Data Centre via https://www.ccdc.cam.ac.uk/mystructures/structuredetails/c616fb23-0367-ec11-96a9-00505695f620.

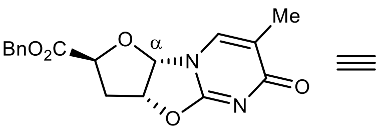

6a

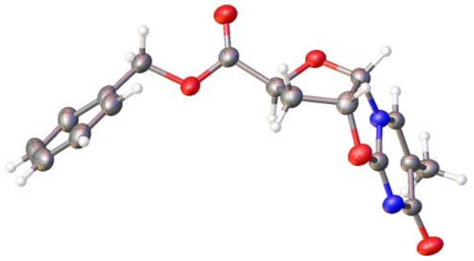

X-ray of **6a**
CCDC No. 2155961

Supplementary Figure 3. X-ray of **6a.**

Supplementary Table 3. Crystallographic data of compound **6a.**

Empirical formula	$C_{17}H_{16}N_2O_5$
Formula weight	328.32
Temperature/K	171.0
Crystal system	monoclinic
Space group	$P2_1$
a/Å	6.1423(7)
b/Å	30.361(4)
c/Å	16.697(2)
α/°	90
β/°	98.701(6)
γ/°	90
Volume/Å^3	3078.0(6)
Z	8
ρ_{calc}g/cm^3	1.417
μ/mm^{-1}	0.885
F(000)	1376.0
Crystal size/mm^3	0.25 × 0.21 × 0.04
Radiation	CuKα (λ = 1.54178)
2Θ range for data collection/°	5.354 to 134.15
Index ranges	-7 ≤ h ≤ 7, -36 ≤ k ≤ 35, -19 ≤ l ≤ 19
Reflections collected	20270
Independent reflections	10245 [R_{int} = 0.0602, R_{sigma} = 0.0779]
Data/restraints/parameters	10245/1/869
Goodness-of-fit on F^2	1.059
Final R indexes [I>=2σ (I)]	R_1 = 0.0614, wR_2 = 0.1660
Final R indexes [all data]	R_1 = 0.0708, wR_2 = 0.1817
Largest diff. peak/hole / e Å^{-3}	0.37/-0.32
Flack parameter	0.04(12)

These data can be obtained free of charge from The Cambridge Crystallographic Data Centre via https://www.ccdc.cam.ac.uk/mystructures/structuredetails/bab92800-3b9a-ec11-96aa-00505695281c.

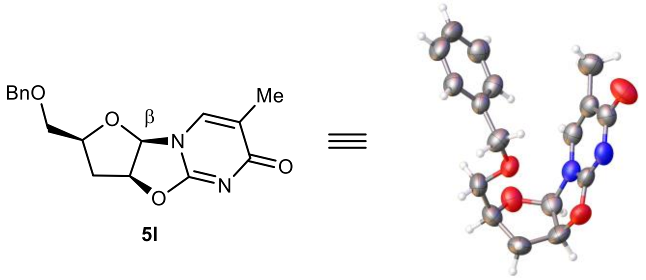

X-ray of **5l**
CCDC No. 2075890

Supplementary Figure 4. X-ray of **5l.**

Supplementary Table 4. Crystallographic data of compound **5l.**

Empirical formula	$C_{17}H_{18}N_2O_4$
Formula weight	314.33
Temperature/K	300.0
Crystal system	orthorhombic
Space group	$P2_12_12_1$
a/Å	5.92720(10)
b/Å	15.9633(3)
c/Å	16.3793(3)
α/°	90
β/°	90
γ/°	90
Volume/Å^3	1549.77(5)
Z	4
ρ_{calc}g/cm^3	1.347
μ/mm^{-1}	0.801
F(000)	664.0
Crystal size/mm^3	0.42 × 0.08 × 0.07
Radiation	CuKα (λ = 1.54178)
2Θ range for data collection/°	7.734 to 136.454
Index ranges	-7 ≤ h ≤ 7, -19 ≤ k ≤ 18, -18 ≤ l ≤ 19
Reflections collected	16748
Independent reflections	2829 [R_{int} = 0.0497, R_{sigma} = 0.0273]
Data/restraints/parameters	2829/0/209
Goodness-of-fit on F^2	1.067
Final R indexes [I>=2σ (I)]	R_1 = 0.0350, wR_2 = 0.0881
Final R indexes [all data]	R_1 = 0.0389, wR_2 = 0.0911
Largest diff. peak/hole / e Å^{-3}	0.08/-0.20
Flack parameter	0.00(11)

These data can be obtained free of charge from The Cambridge Crystallographic Data Centre via https://www.ccdc.cam.ac.uk/mystructures/structuredetails/45dec435-0867-ec11-96a9-00505695f620.

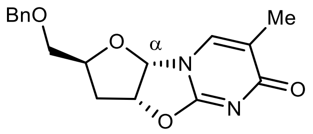

6l

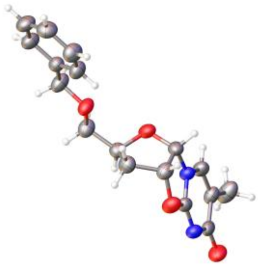

X-ray of **6l**
CCDC No. 2075734

Supplementary Figure 5. X-ray of **6l.**

Supplementary Table 5. Crystallographic data of compound **6l.**

Empirical formula	$C_{17}H_{18}N_2O_4$
Formula weight	314.33
Temperature/K	294.90(10)
Crystal system	orthorhombic
Space group	$P2_12_12_1$
a/Å	6.6705(3)
b/Å	7.0812(2)
c/Å	33.3791(13)
α/°	90
β/°	90
γ/°	90
Volume/Å^3	1576.68(11)
Z	4
ρ_{calc}g/cm^3	1.324
μ/mm^{-1}	0.787
F(000)	664.0
Crystal size/mm^3	0.55 × 0.35 × 0.35
Radiation	CuKα (λ = 1.54184)
2Θ range for data collection/°	10.602 to 143.432
Index ranges	-8 ≤ h ≤ 5, -5 ≤ k ≤ 8, -40 ≤ l ≤ 38
Reflections collected	7426
Independent reflections	3010 [R_{int} = 0.0285, R_{sigma} = 0.0331]
Data/restraints/parameters	3010/0/209
Goodness-of-fit on F^2	1.089
Final R indexes [I>=2σ (I)]	R_1 = 0.0555, wR_2 = 0.1577
Final R indexes [all data]	R_1 = 0.0586, wR_2 = 0.1634
Largest diff. peak/hole / e Å^{-3}	0.22/-0.32
Flack parameter	0.05(17)

These data can be obtained free of charge from The Cambridge Crystallographic Data Centre via https://www.ccdc.cam.ac.uk/mystructures/structuredetails/1bbd405a-0367-ec11-96a9-00505695f620.

2 Supplementary Discussion

2.1 Optimization of the reaction conditions

Supplementary Table 6. Screening the catalysts[a].

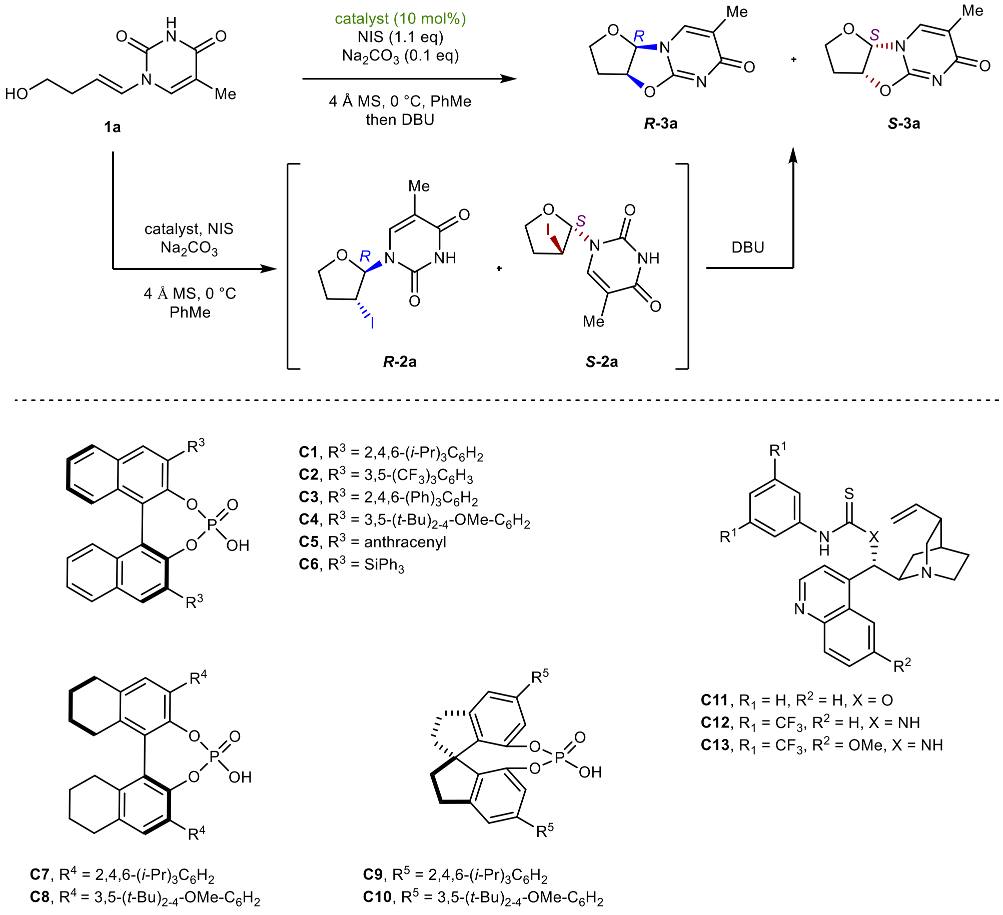

Entry	Catalyst	Yield (%)[b]	ee (%)[c]
1	**C1**	82	52 (R)
2	**C2**	75	<5 (R)
3	**C3**	70	<5 (R)
4	**C4**	78	<5 (R)
5	**C5**	80	40 (R)
6	**C6**	72	10 (R)
7	**C7**	78	20 (R)
8	**C8**	82	<5 (R)
9	**C9**	72	<5 (R)
10	**C10**	75	6 (R)
11	**C11**	65	14 (R)
12	**C12**	trace	-
13	**C13**	trace	-

[a]All reactions were performed on 0.1 mmol scale at 40 mM for 8 h. [b]isolated yield. [c]Enantiomeric excess (*ee*) values were determined by chiral HPLC.

Supplementary Table 7. Screening the solvent[a].

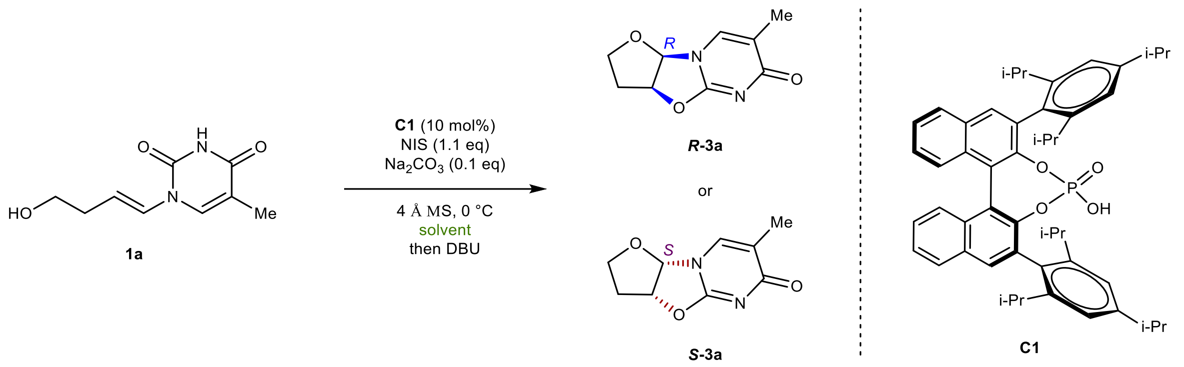

Entry	Solvent	Yield (%)[b]	*ee* (%)[c]
1	PhMe	82	52 (*R*)
2	CH_2Cl_2	80	31 (*R*)
3	$CHCl_3$	86	37 (*R*)
4	EtOAc	75	15 (*R*)
5	DMF	70	5 (*S*)
6	THF	76	6 (*R*)
7	PhMe:$CHCl_3$ = 5:1	83	55 (*R*)
8	PhMe:$CHCl_3$ = 1:1	89	70 (*R*)
9	PhMe:CCl_4 = 1:1	83	20 (*R*)
10	PhMe:$CHCl_3$/HFIP = 5:5:1	50	< 5 (*R*)
11	*o*-xylene:$CHCl_3$ = 1:1	82	54 (*R*)
12	*p*-xylene:$CHCl_3$ = 1:1	82	59 (*R*)

[a]All reactions were performed on 0.1 mmol scale at 40 mM for 8 h; [b]isolated yield; [c]*ee* determined by chiral HPLC.

Supplementary Table 8. Screening the halogen[a].

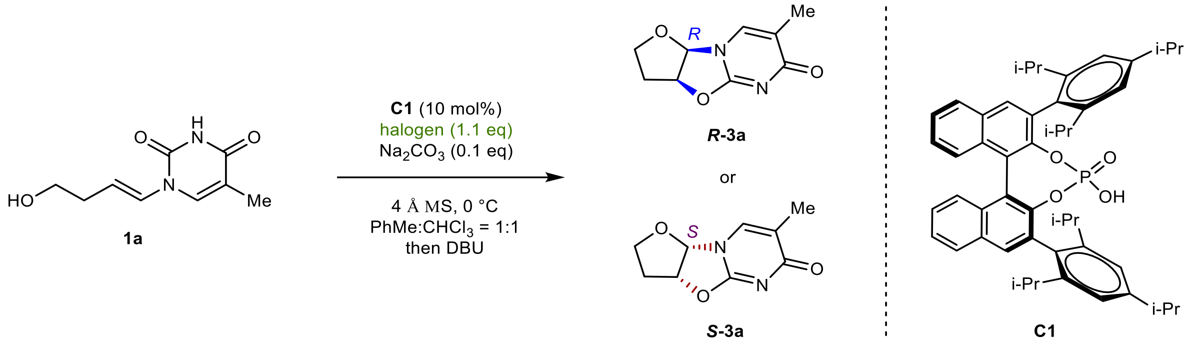

halogen.

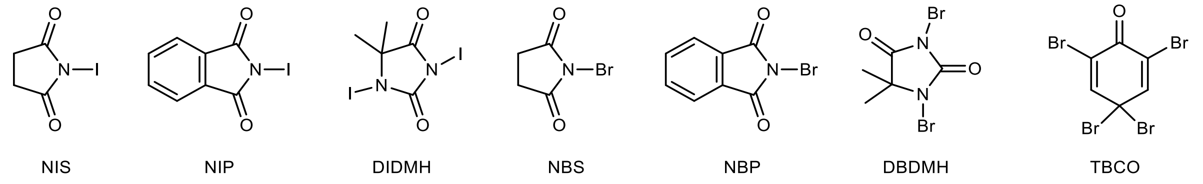

Entry	Halogen	Yield (%)[b]	*ee* (%)[c]
1	I_2	58	8 (*R*)
2	ICl	70	< 5 (*R*)
3	IBr	70	< 5 (*R*)
4	NIS	89	70 (*R*)
5	NIP	84	48 (*R*)
6	DIDMH	79	45 (*R*)
7	NBS	88	45 (*R*)
8	NBP	85	17 (*R*)
9	DBDMH	86	28 (*R*)
10	TBCO	83	20 (*R*)

[a]All reactions were performed on 0.1 mmol scale at 40 mM for 8 h; [b]isolated yield; [c]*ee* determined by chiral HPLC.

Supplementary Table 9. Screening the additive and solvent.

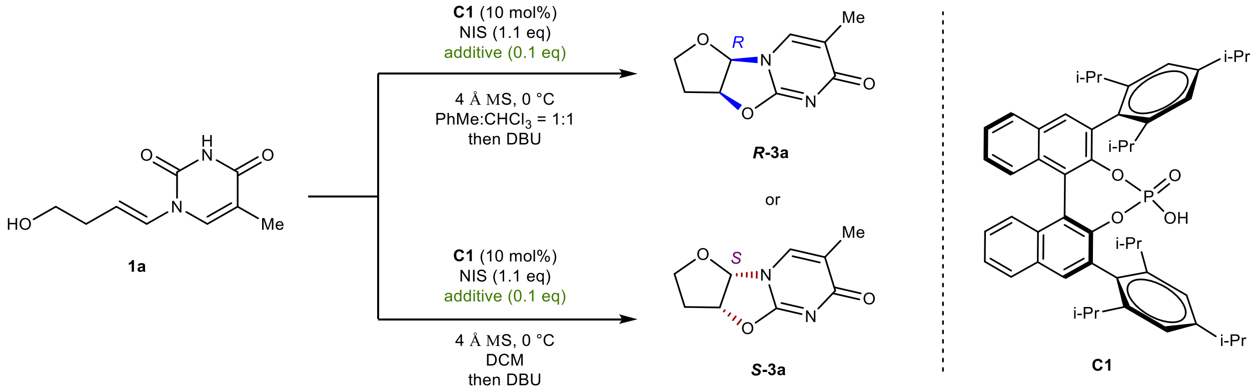

Entry	Additive	Yield (%)[b]	*ee* (%)[c]
1[a]	-	85	20 (*R*)
2[a]	Na_2CO_3	89	70 (*R*)
3[a]	K_2CO_3	89	28 (*R*)
4[a]	K_3PO_4	80	20 (*R*)
5[a]	KI	89	60 (*R*)
6[a]	NaI	93	96 (*R*)
7[e]	NaI	91	82 (*R*)
8[f]	NaI	89	88 (*R*)
9[a]	PPh_3S	92	51 (*S*)
10[g]	PPh_3S	90	85 (*S*)
11[d]	PPh_3S	94	90 (*S*)
12[d]	PPh_3O	80	33 (*S*)
13[d]	*n*-Bu_3PO	70	< 5 (*S*)
14[d]	$(Me_2N)_3PO$	65	12 (*S*)
15[d]	$(Me_2N)_2CS$	trace	-
16[d]	$(Me_2N)_2CO$	82	24 (*S*)
17[d]	Me_2S	60	< 5 (*S*)

[a]All reactions were performed on 0.1 mmol scale at 40 mM (PhMe:$CHCl_3$ = 1:1) for 8 h; [b]isolated yield; [c]*ee* determined by chiral HPLC; [d]all reactions were performed on 0.1 mmol scale at 40 mM (CH_2Cl_2) for 8 h; [e]solvent: $CHCl_3$; [f]solvent: PhMe; [g]solvent: EtOAc.

Supplementary Table 10. Screening the concentration and temperature[a].

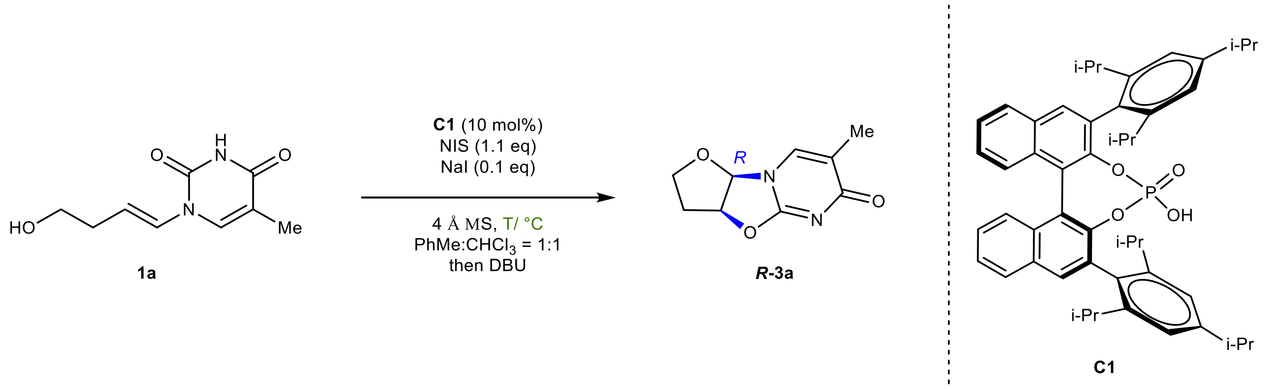

Entry	C (M)	T (° C)	Time (h)	Yield (%)[b]	*ee* (%)[c]
1	0.02	0	48	91	75 (*R*)
2	0.04	0	8	93	96 (*R*)
3	0.08	0	5	88	84 (*R*)
4	0.1	0	2	82	75 (*R*)
5	0.04	25	2	86	48 (*R*)
6	0.04	-20	24	91	91 (*R*)
7	0.04	-60	> 48	83	52 (*R*)

[a]All reactions were performed on 0.1 mmol scale; [b]isolated yield; [c]*ee* determined by chiral HPLC.

Supplementary Table 11. Screening the catalyst amount[a].

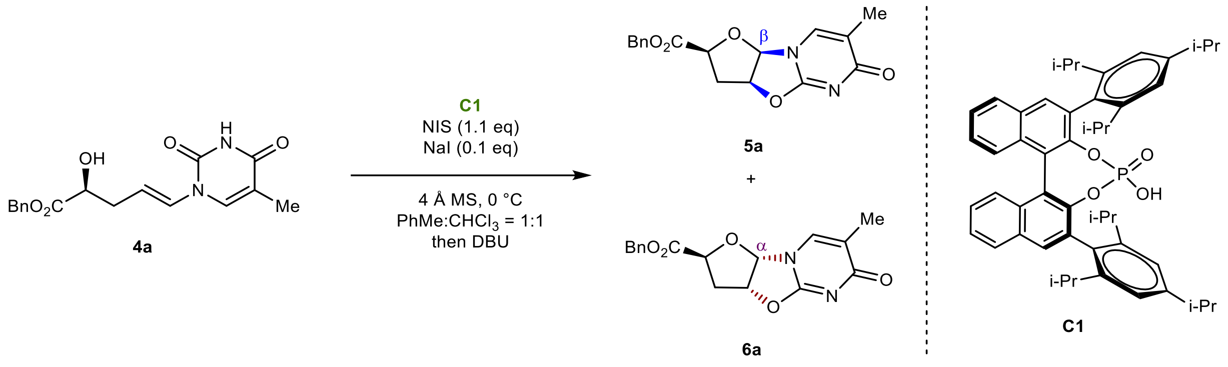

Entry	Catalyst (mol%)	Time (h)	Yield (%)[b]	dr (5a:6a)[c]
1	-	24	40	43:57
2	2	16	81	88:12
3	5	12	89	95:5
4	10	8	93	98:2

[a]All reactions were performed on 0.1 mmol scale at 40 mM; [b]isolated yield of **5a**; [c]the diaostereoselectivites was determined by HPLC analysis.

2.2 Mechanistic Studies.

Supplementary Table 12. Control experiments.[a]

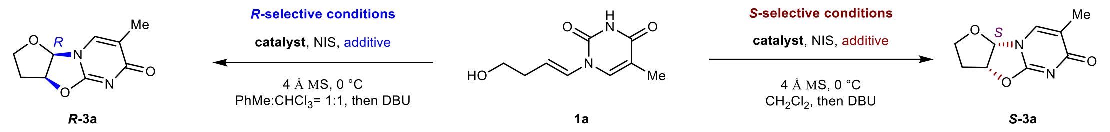

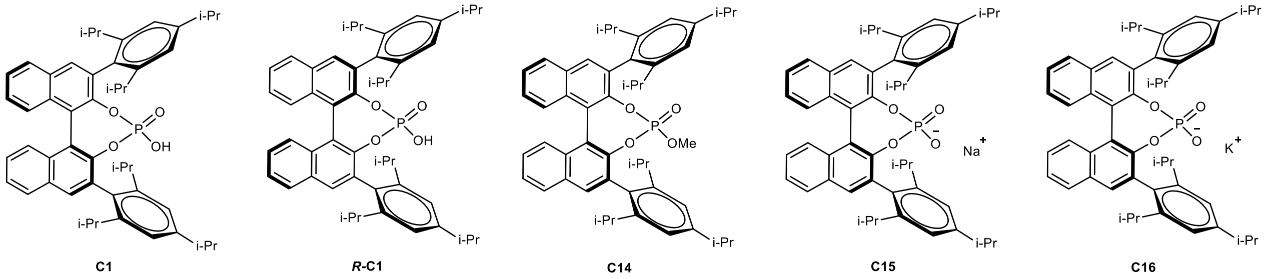

	Entry	Catalyst	Additive	NIS	Yield (%)[b]	*ee* (%)[c]
	1	**C1**	NaI (0.1 eq.)	NIS	93	96 (*R*)
	2	**C1**	NaI (0.1 eq.)	-	0	-
	3	**C1**	-	NIS	85	20 (*R*)
	4	-	NaI (0.1 eq.)	NIS	84	0
	5	***R*-C1**	NaI (0.1 eq.)	NIS	93	90 (*S*)
	6	**C14**	NaI (0.1 eq.)	NIS	80	< 5 (*R*)
***R*-selective**	7	**C15**	-	NIS	88	35 (*R*)
**conditions**	8	**C16**	-	NIS	87	35 (*R*)
	10	**C1**	NaI (0.5 eq.)	NIS	93	93 (*R*)
	11	**C1**	NaI (0.2 eq.)	NIS	93	95 (*R*)
	12	**C1**	NaI (0.07 eq.)	NIS	92	78 (*R*)
	13	**C1**	NaI (0.03 eq.)	NIS	88	31 (*R*)
	14[d]	**C1**	NaI (0.1 eq.)	NIS	91	82 (*R*)
	15[e]	**C1**	NaI (0.1 eq.)	NIS	89	88 (*R*)
	16[f]	**C1**	PPh_3S (0.1 eq.)	NIS	94	90 (*S*)
***S*-selective**	17[f]	**C1**	-	NIS	85	13 (*R*)
**conditions**	18[f]	-	PPh_3S (0.1 eq.)	NIS	86	0
	19[f]	***R*-C1**	PPh_3S (0.1 eq.)	NIS	94	86 (*R*)
	20[f]	**C14**	PPh_3S (0.1 eq.)	NIS	81	< 5 (*S*)

[a]All reactions were performed on 0.1 mmol scale at 40 mM (PhMe:$CHCl_3$ = 1:1) for 8 h; [b]isolated yield; [c]*ee* determined by chiral HPLC; [d]the reactions was performed on 0.1 mmol scale at 40 mM ($CHCl_3$) for 8 h; [e]the reaction was performed on 0.1 mmol scale at 40 mM (PhMe) for 8 h; [f]all reactions were performed on 0.1 mmol scale at 40 mM (CH_2Cl_2) for 8 h.

Supplementary Table 13. Screening the additives in *R*-selective conditions.[a]

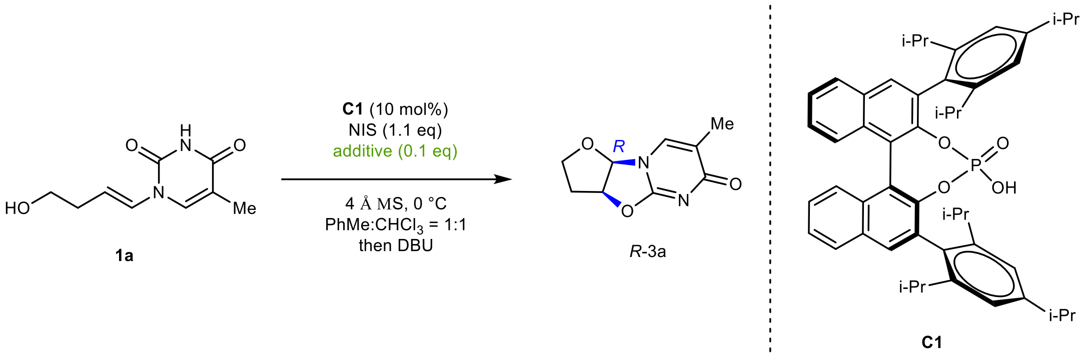

Entry	Additives	Yield (%)[b]	*ee* (%)[c]
1	NaI	93	96 (*R*)
2	NaCl	75	25 (*R*)
3	NaBr	90	70 (*R*)
4	Na_2CO_3	89	70 (*R*)
8	KI	89	60 (*R*)
5	KCl	80	20 (*R*)
6	KBr	83	38 (*R*)
7	K_2CO_3	89	28 (*R*)
9	LiI	91	82 (*R*)
10	CsI	87	50 (*R*)
11	ZnI_2	30	10 (*R*)
12	CuI	78	18 (*R*)
13	NH_4I	36	5 (*R*)

[a]All reactions were performed on 0.1 mmol scale at 40 mM (PhMe:$CHCl_3$ = 1:1) for 8 h; [b]isolated yield; [c]*ee* determined by chiral HPLC.

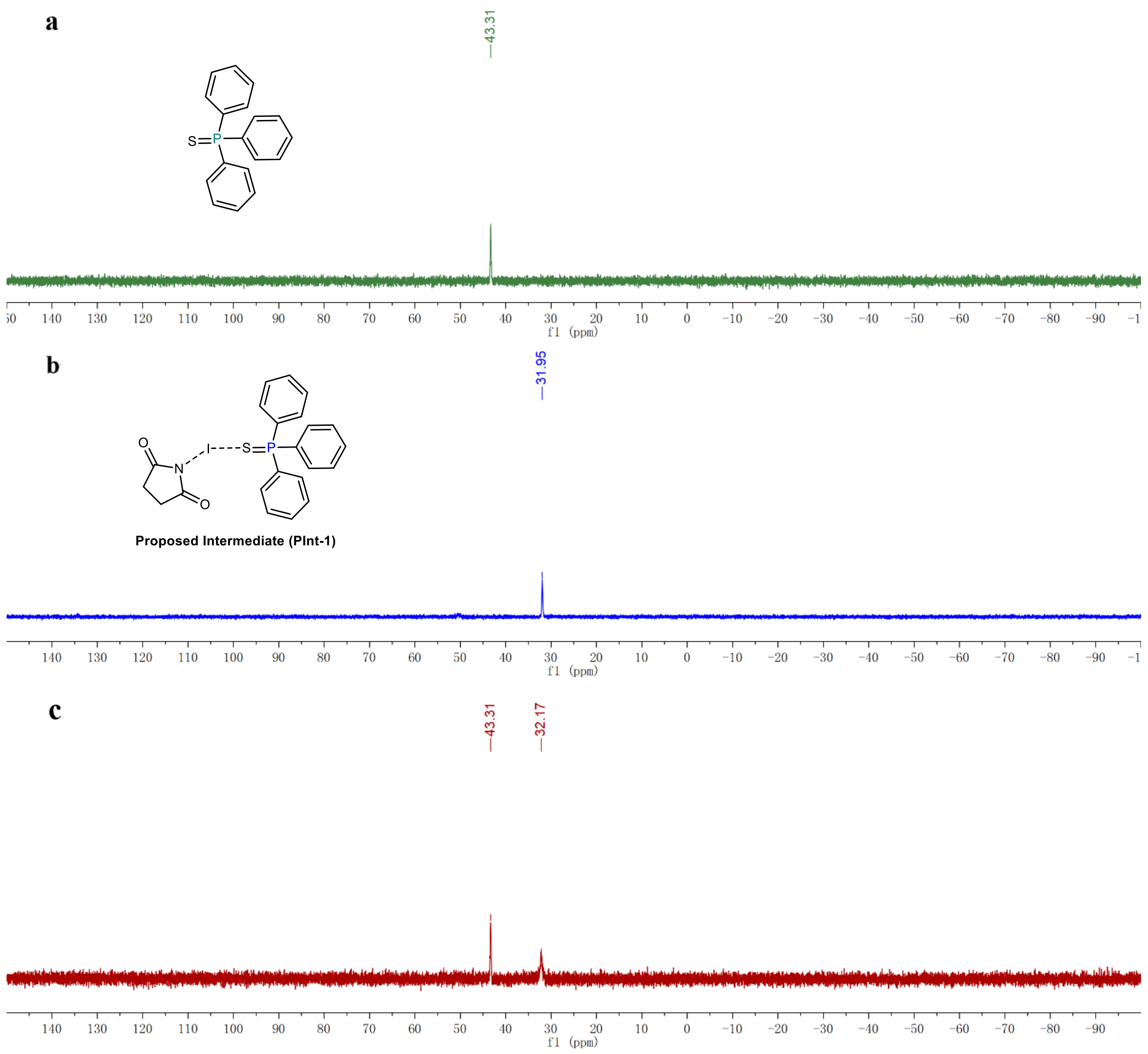

Supplementary Figure 6. Phosphorous NMR suggesting pausible lewis basic mechanism. Results in Supplementary Figure 6 were obtained as following: PPh_3S (1.5 mg, 0.005 mmol) was added to an NMR tube followed by the addition of 500 μL $CDCl_3$, of which a ^{31}P spectrum was obtained (Supplementary Figure 6a). Then, NIS (12.4 mg, 0.055 mmol) was added to the NMR tube which was shaken vigorously for 5 minutes, of which a NMR ^{31}P spectrum was obtained (Supplementary Figure 6b). After obtaining the second ^{31}P spectrum, compound **1a** (9.8 mg, 0.05 mmol) was added to the NMR tube and shaken vigorously for 2h, of which a NMR ^{31}P spectrum (Supplementary Figure 6c) was obtained.

Supplementary Table 14. Diastereoselective catalysis.

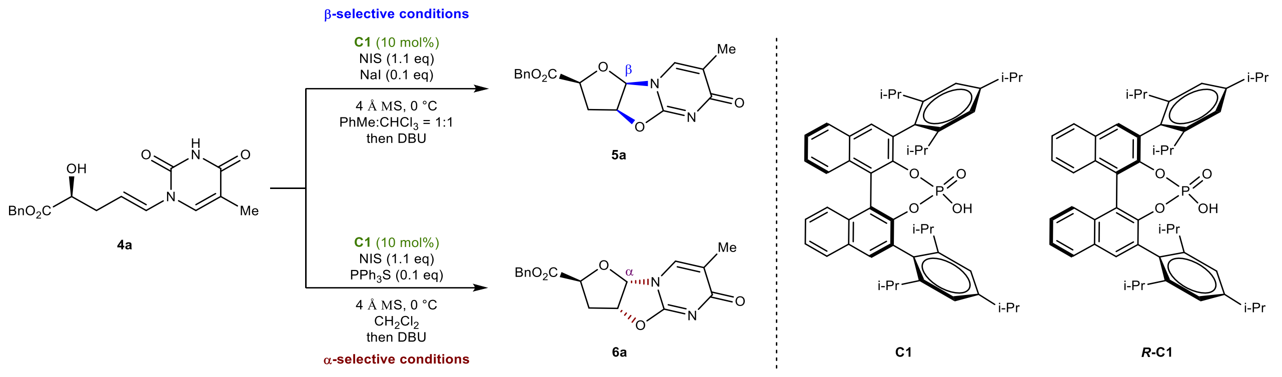

Entry	Catalyst	Additive	Iodide source	Yield (%)[b]	dr (5a:6a)[c]
1[a]	-	-	NIS	42	51:49
2[a]	**C1**	-	NIS	41	48:52
3[a]	-	NaI	NIS	40	43:57
4[a]	**C1**	NaI	NIS	93	98:2
5[a]	***R*-C1**	NaI	NIS	33	35:65
6[d]	-	-	NIS	40	53:47
7[d]	**C1**	-	NIS	46	46:54
8[d]	-	PPh_3S	NIS	47	42:58
9[d]	**C1**	PPh_3S	NIS	94	3:97
10[d]	***R*-C1**	PPh_3S	NIS	90	7:93

[a]All reactions were performed on 0.1 mmol scale at 40 mM (PhMe:$CHCl_3$ = 1:1) for 8 h; [b]isolated yield of **5a** or **6a**; [c]the diaostereoselectivites was determined by HPLC analysis; [d]all reactions were performed on 0.1 mmol scale at 40 mM (CH_2Cl_2) for 8 h.

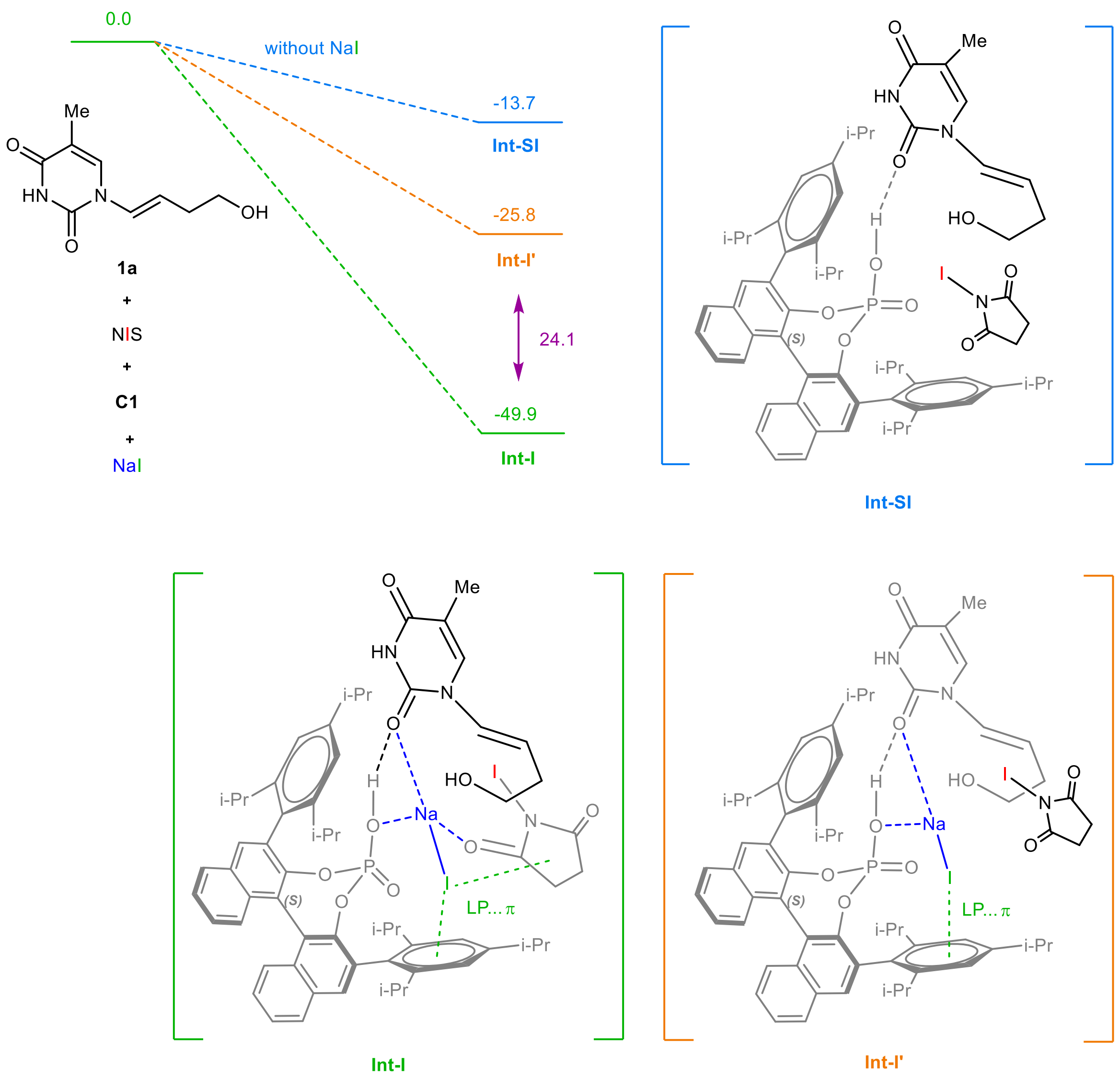

Supplementary Figure 7. Computed pathway for the NaI-controlled stereoselective iodocyclizations.

Computational details

All the species are fully optimized at the M06-2X level[8], the 6-31G(d,p)[9] basis set is used for H, C, N, O, S, P atom, and the LANL2D2 ECP basis set[10] is used for K and I atom. Frequency analyses are performed at the same level to confirm that the characteristics of the structures are minima (without imaginary frequencies) or transition states (only one imaginary frequency). Calculations of the intrinsic reaction coordinates (IRC)[11,12] are calcd. to ensure that the transition states indeed have connected two minima. The single-point energies calcd. at M06-2X Level[8], the 6-311G(d,p)[9] level basis set is used for H, C, N, O, S, P atom, and the def 2-TZVP[13] basis set is used for K and I atom. The single-point energies are added to the Gibbs free energy correction to obtain the Gibbs free energies (Δ*G*). Fuzzy bond orders (FBO)[14] and interaction region indicator (IRI)[15] were performed by Multiwfn[16]. All these calculations are performed with Gaussian 16 program[17].

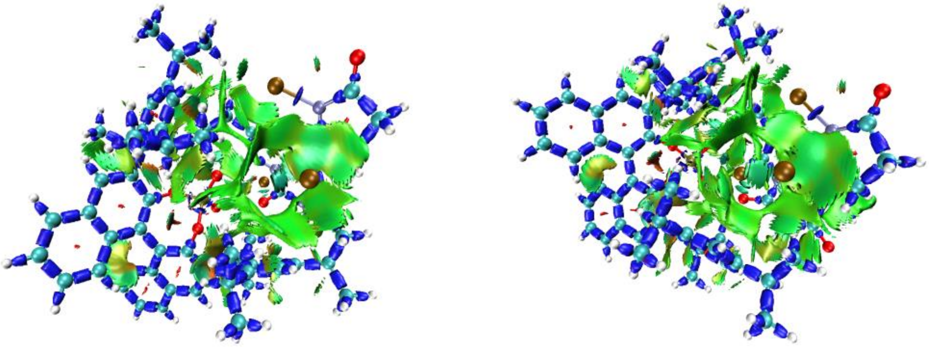

IRI pic. of Int-I　　　　IRI pic. of Int-I-KI

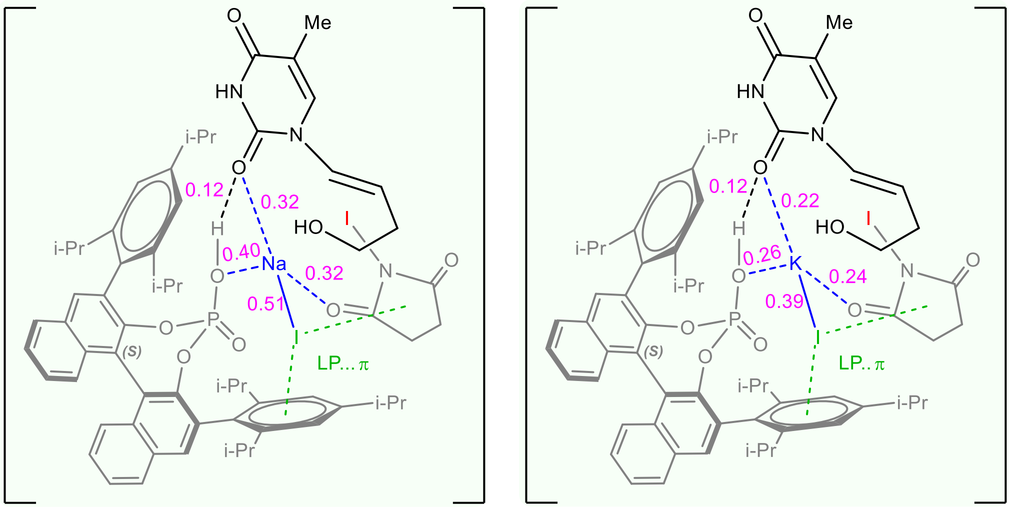

Int-I　　　　Int-I-KI

Supplementary Figure 8. Interaction region indicator (IRI) and fuzzy bond order (FBO) analysis of **Int-I** and **Int-I- KI.** Similar with NaI, KI is also found as a centered role that cooperates with **C1**, NIS and substrate through LP... π interactions and K-O interactions. However, K/Na-O interactions in KI is weaker than that of NaI. The fuzzy bond orders (FBO) of K-O bonds in KI are 0.22, 0.24 and 0.26 respectively, which are weaker than that of Na-O bonds in NaI (0.32, 0.32, 0.40 respectively). Moreover, the FBO value of KI (0.39) is smaller than that of NaI (0.51).

3 Supplementary Figs 9-299

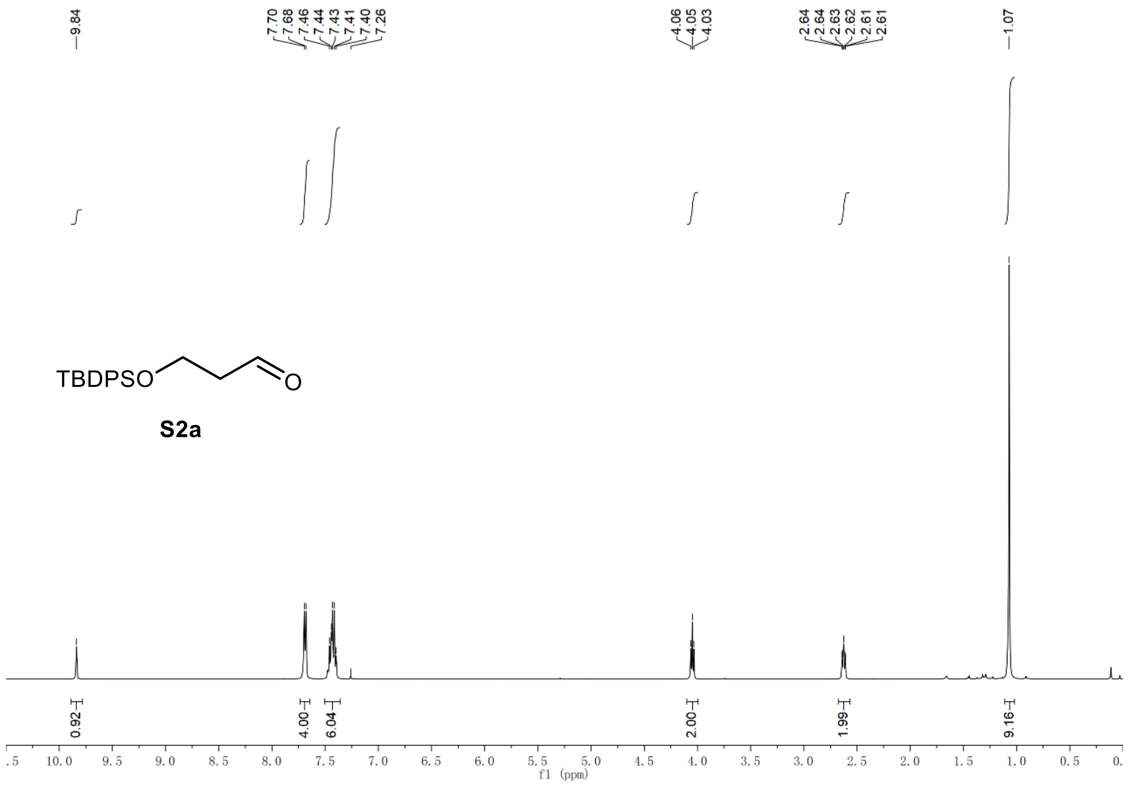

Supplementary Figure 9. ^{1}H NMR (400M, $CDCl_3$) of compound **S2a.**

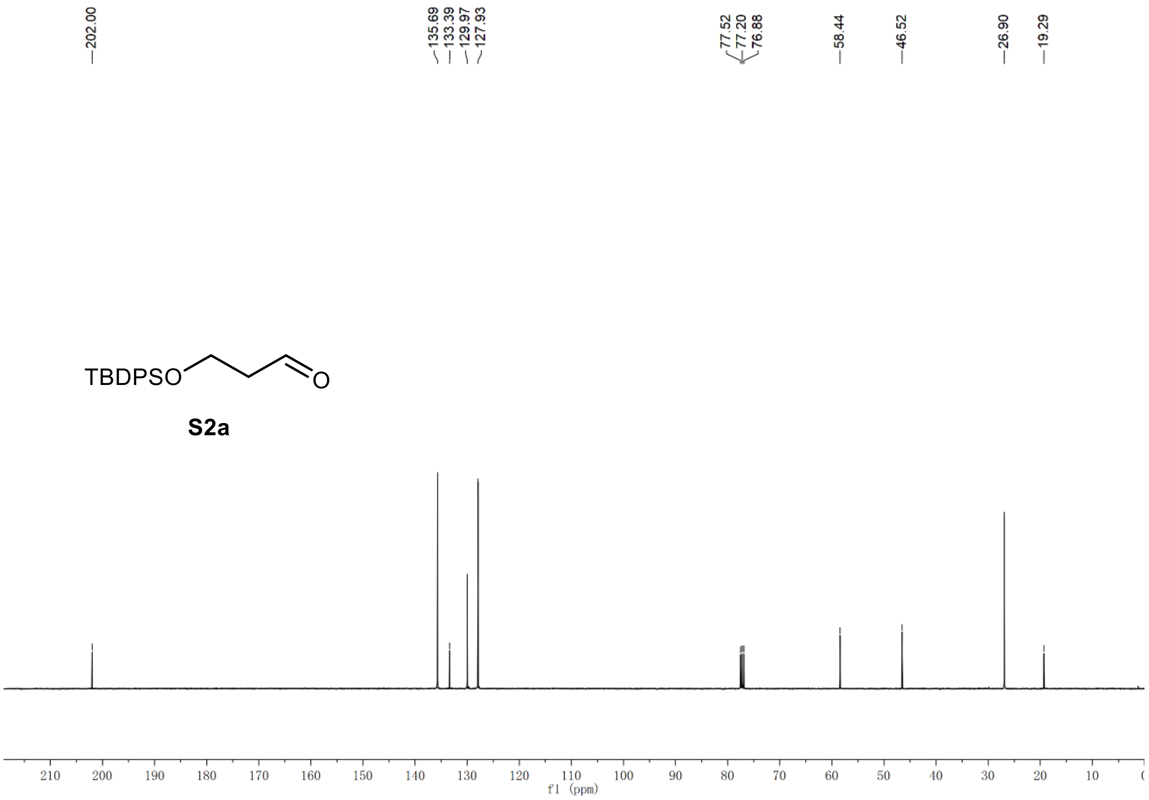

Supplementary Figure 10. ^{13}C NMR (100M, $CDCl_3$) of compound **S2a.**

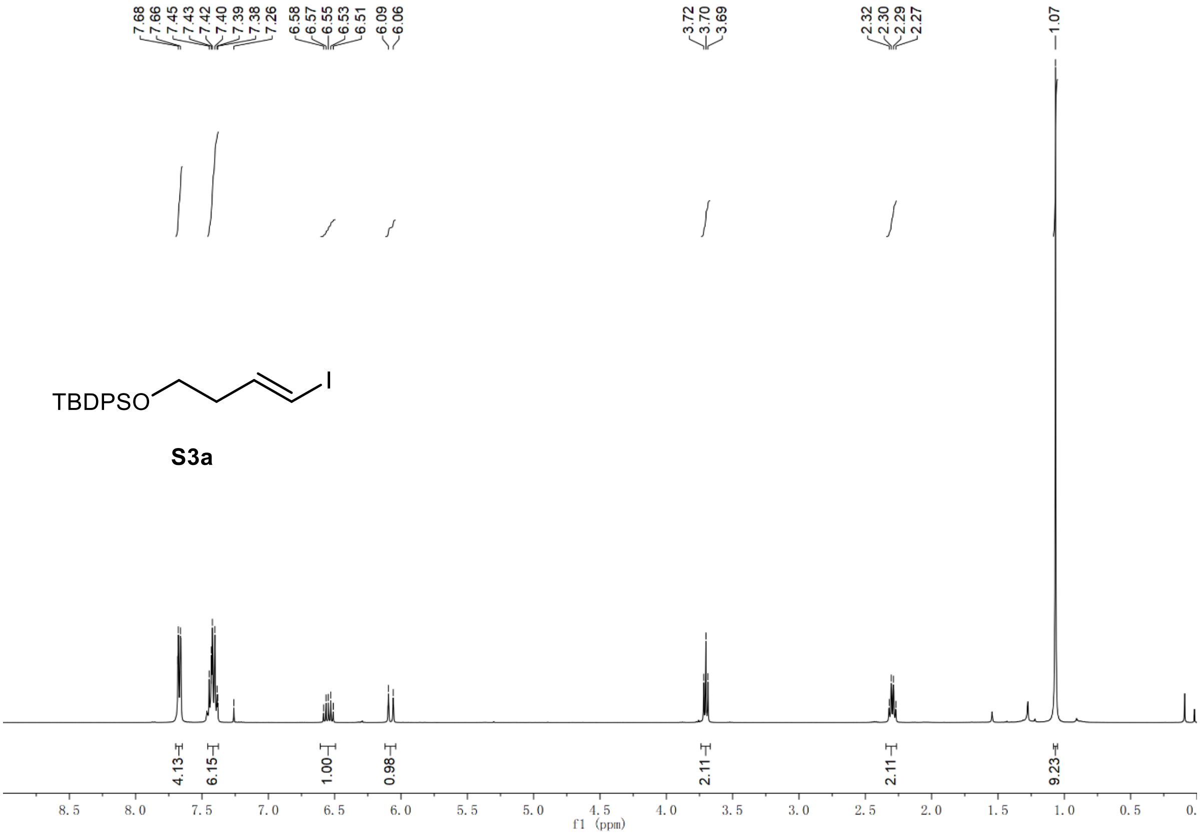

Supplementary Figure 11. ^{1}H NMR (400M, $CDCl_3$) of compound **S3a.**

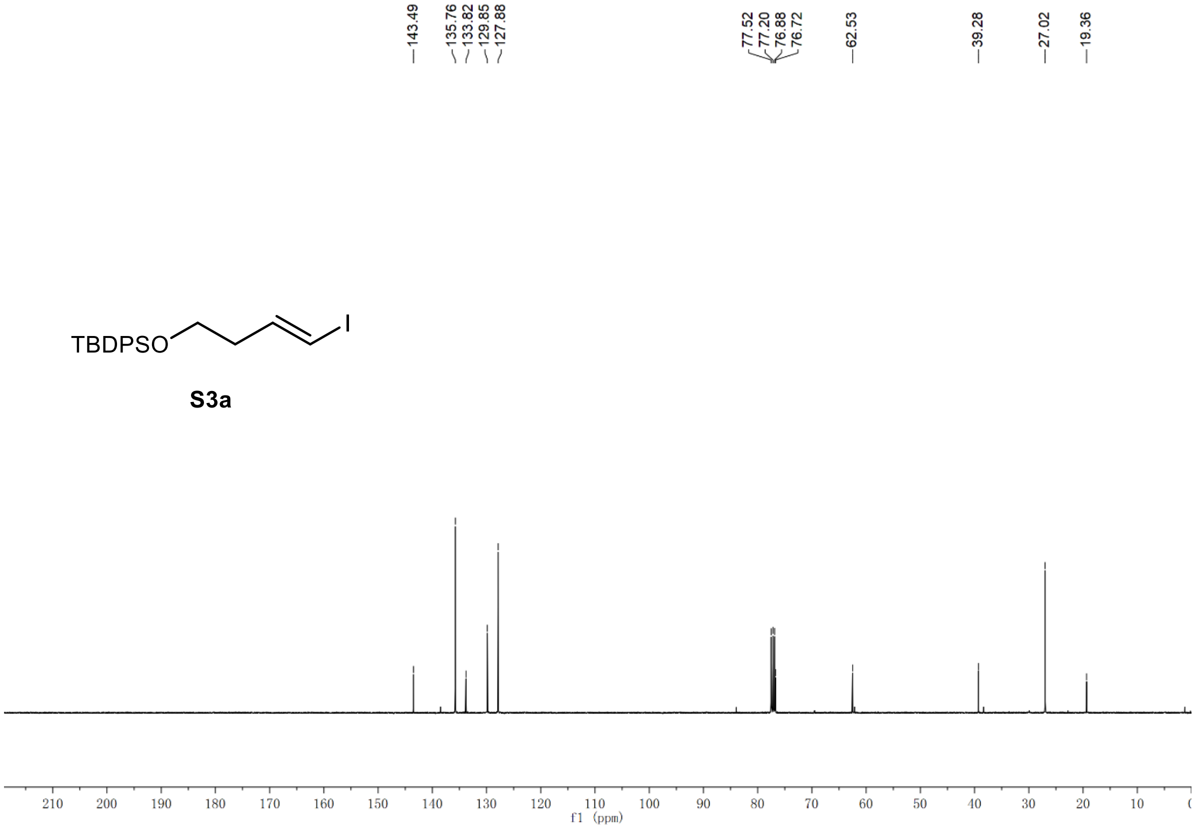

Supplementary Figure 12. ^{13}C NMR (100M, $CDCl_3$) of compound **S3a.**

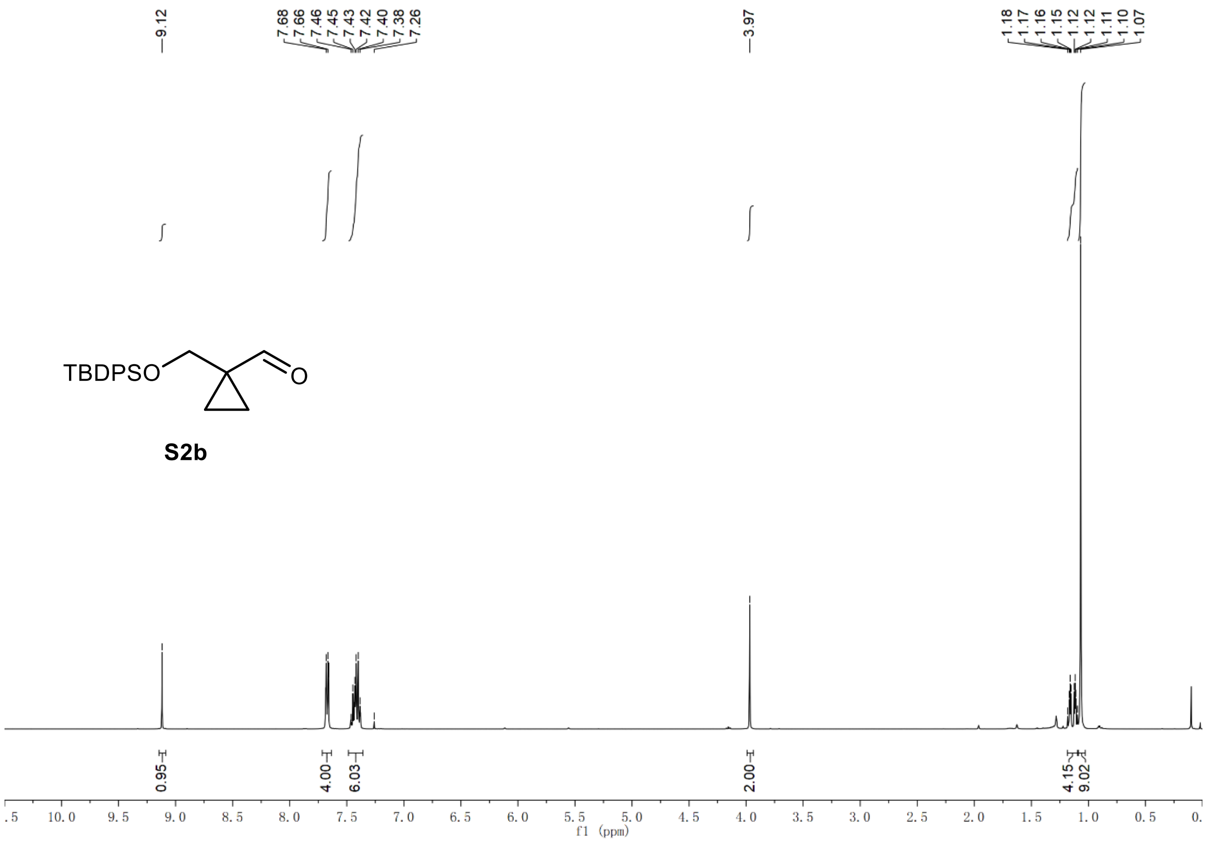

Supplementary Figure 13. ^{1}H NMR (400M, $CDCl_3$) of compound **S2b.**

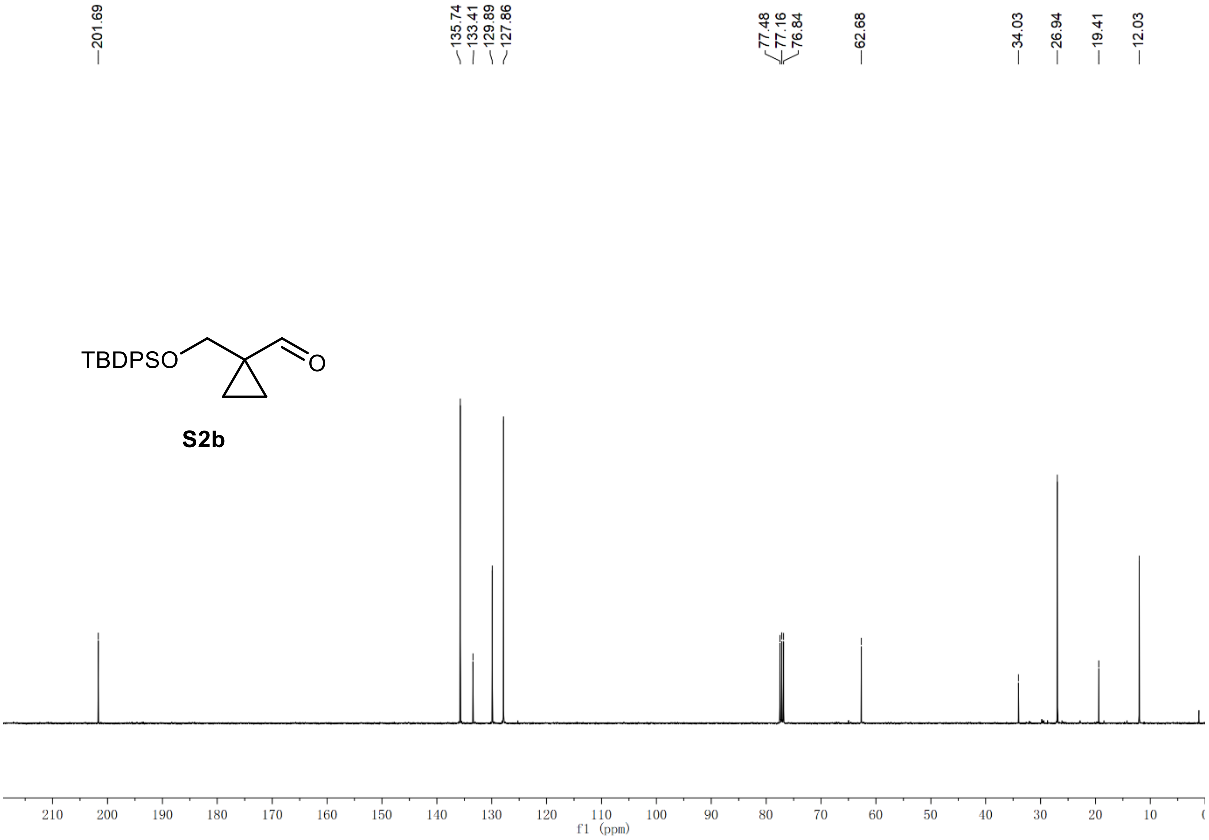

Supplementary Figure 14. ^{13}C NMR (100M, $CDCl_3$) of compound **S2b.**

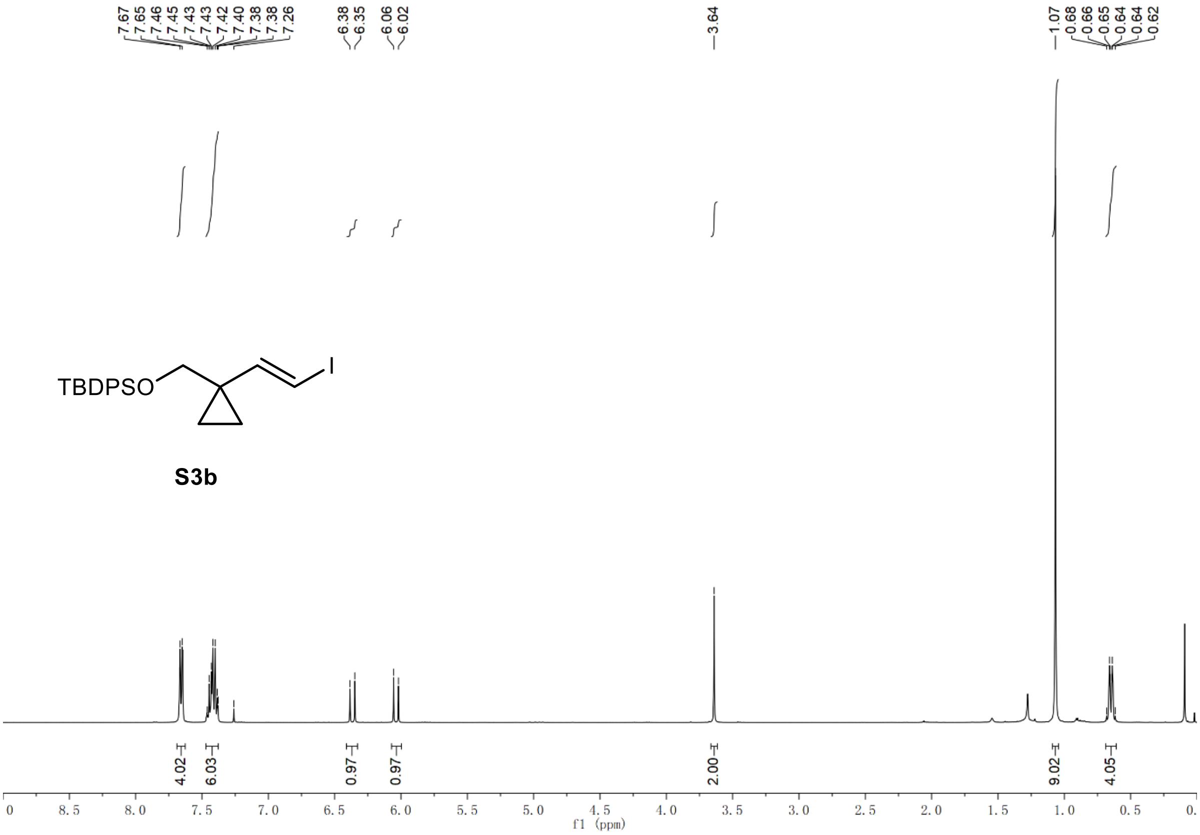

Supplementary Figure 15. ^{1}H NMR (400M, $CDCl_3$) of compound **S3b.**

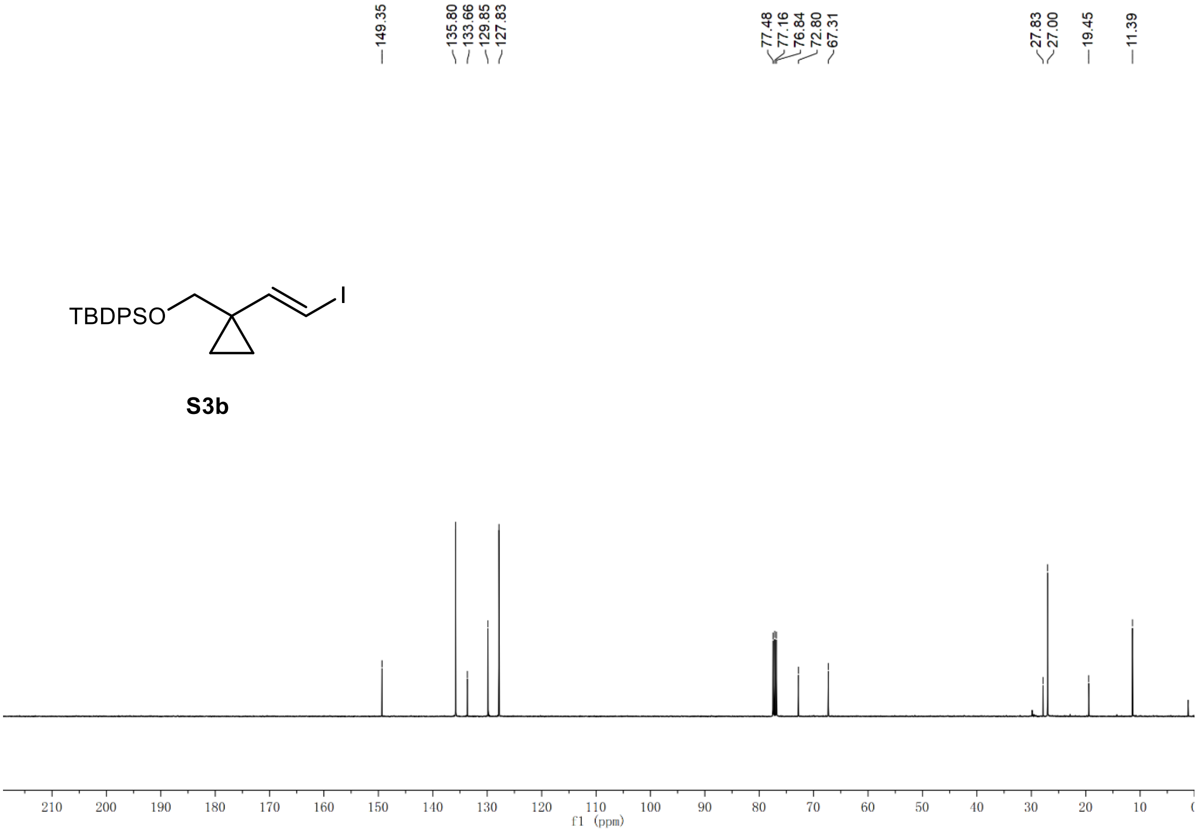

Supplementary Figure 16. ^{13}C NMR (100M, $CDCl_3$) of compound **S3b.**

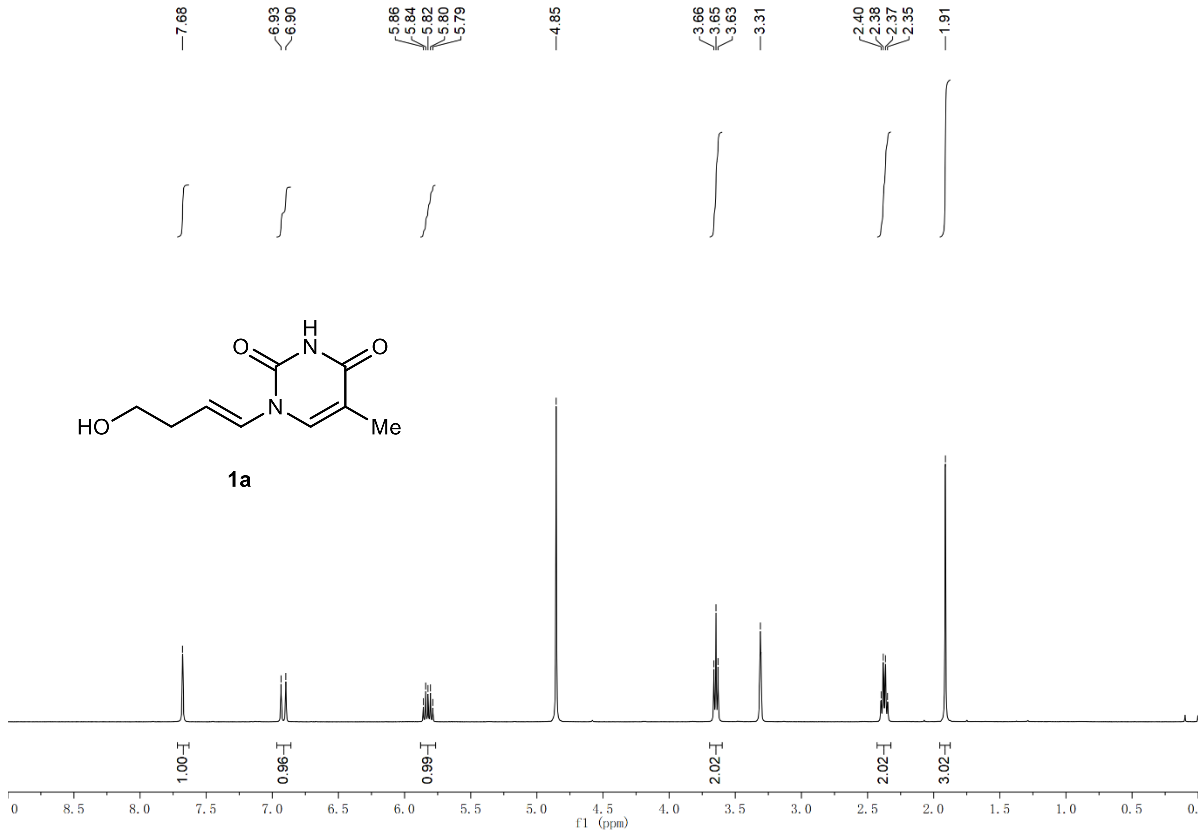

Supplementary Figure 17. ^{1}H NMR (400M, CD_3OD) of compound **1a.**

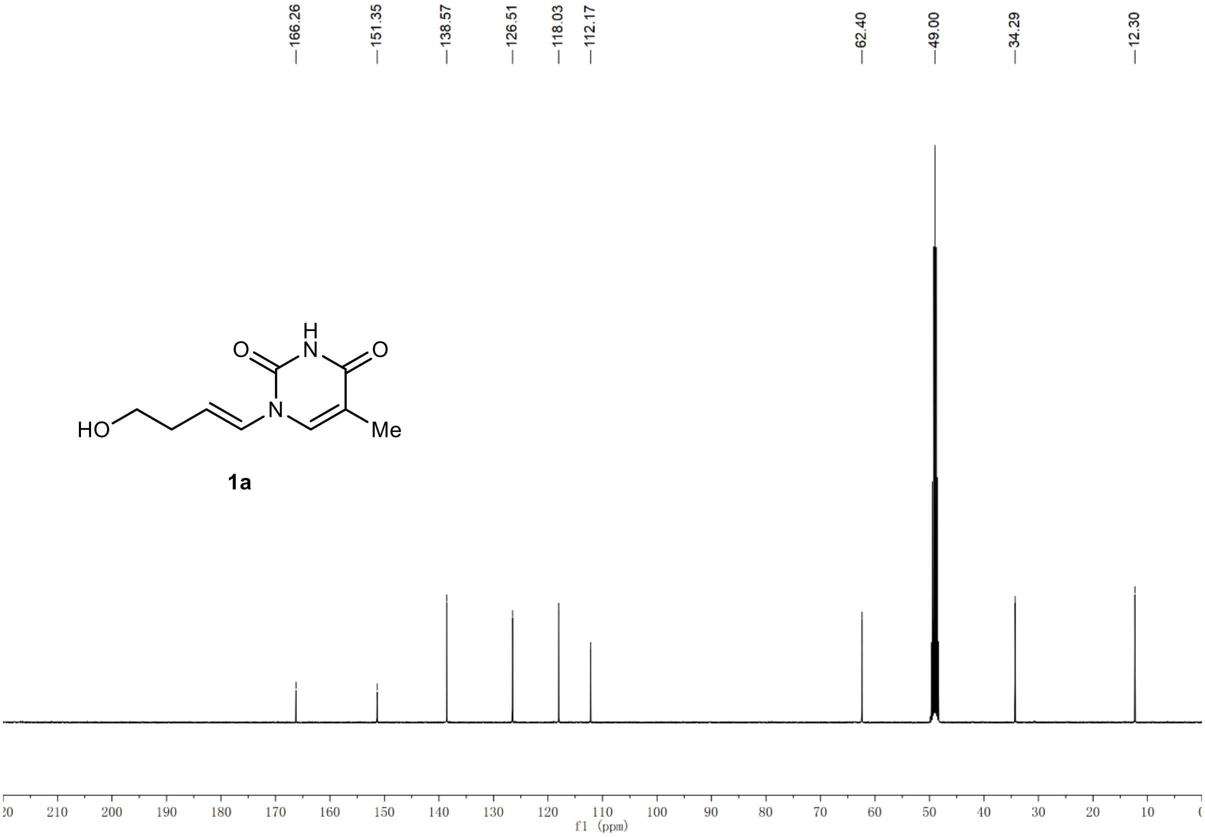

Supplementary Figure 18. ^{13}C NMR (100M, CD_3OD) of compound **1a.**

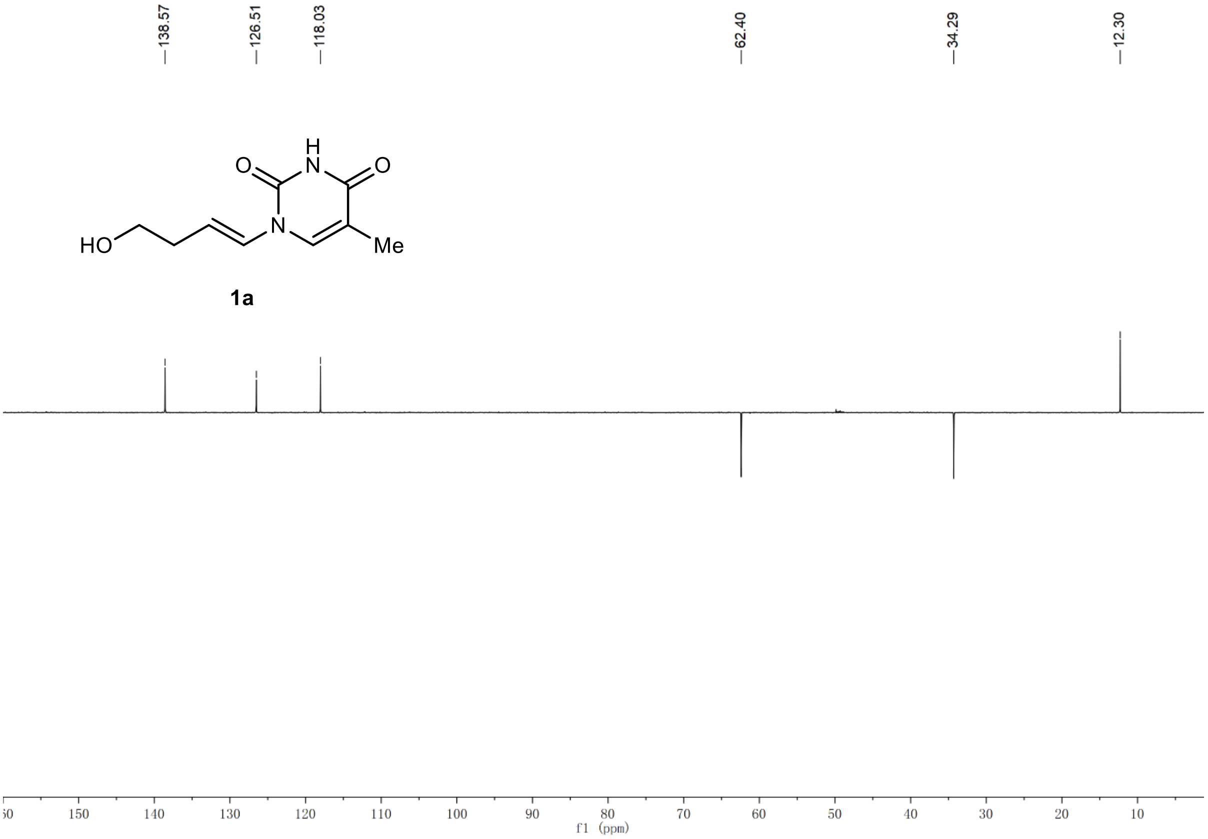

Supplementary Figure 19. DEPT 135° (100M, CD$_3$OD) of compound 1a.

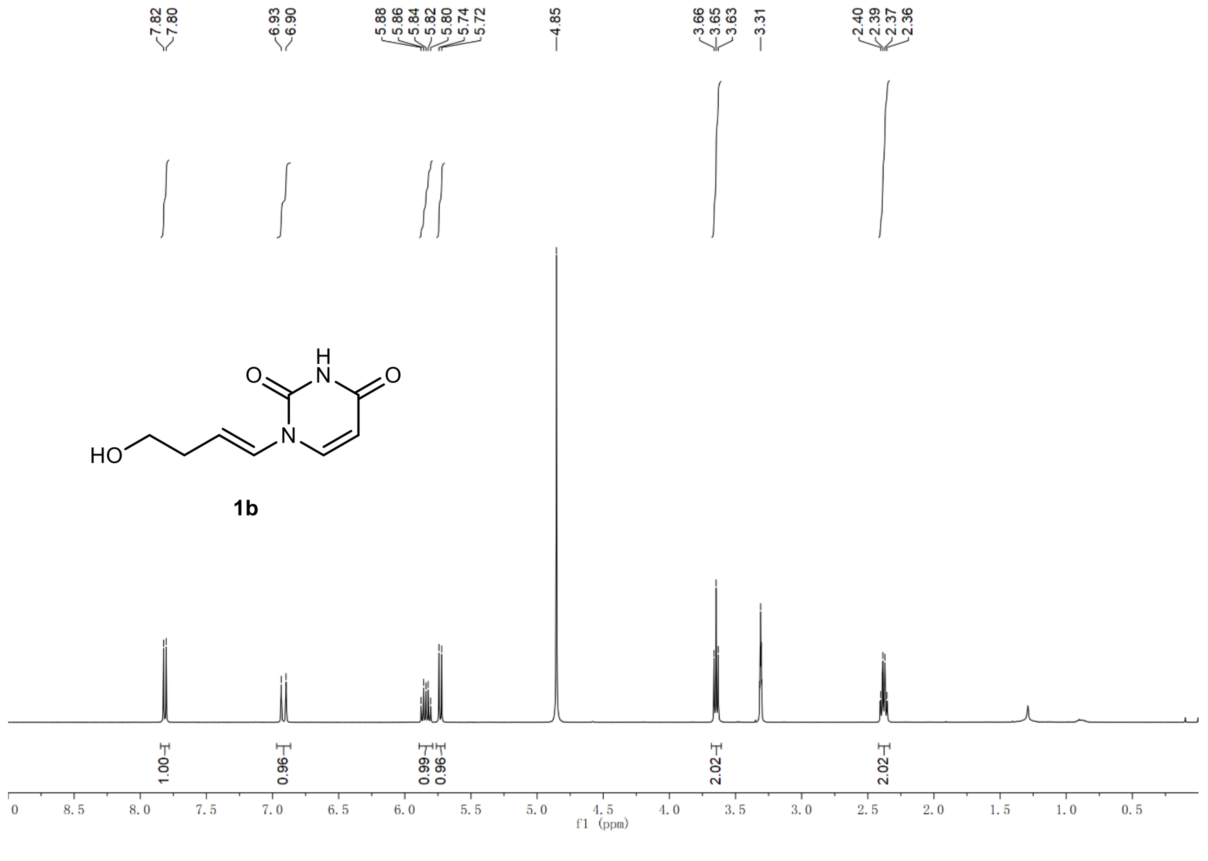

Supplementary Figure 20. ^{1}H NMR (400M, CD$_3$OD) of compound **1b.**

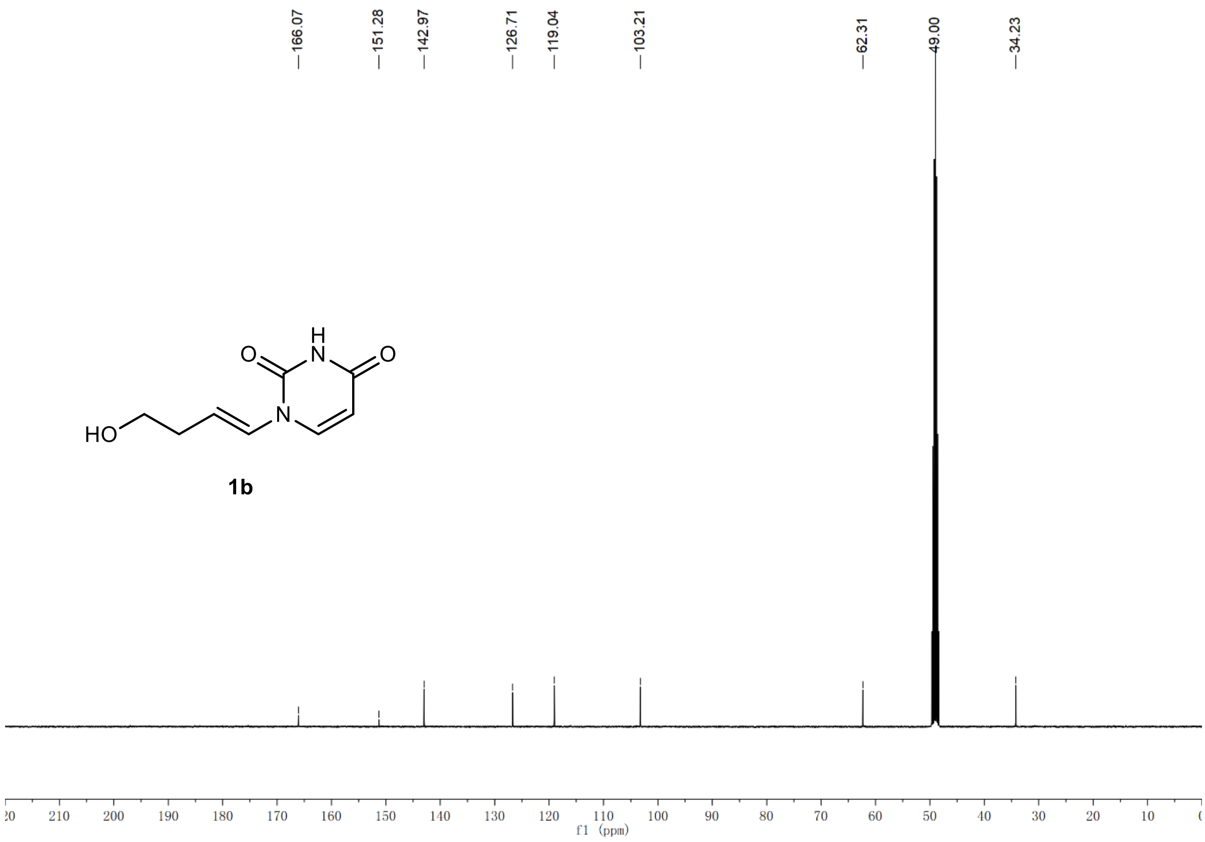

Supplementary Figure 21. ^{13}C NMR (100M, CD_3OD) of compound **1b.**

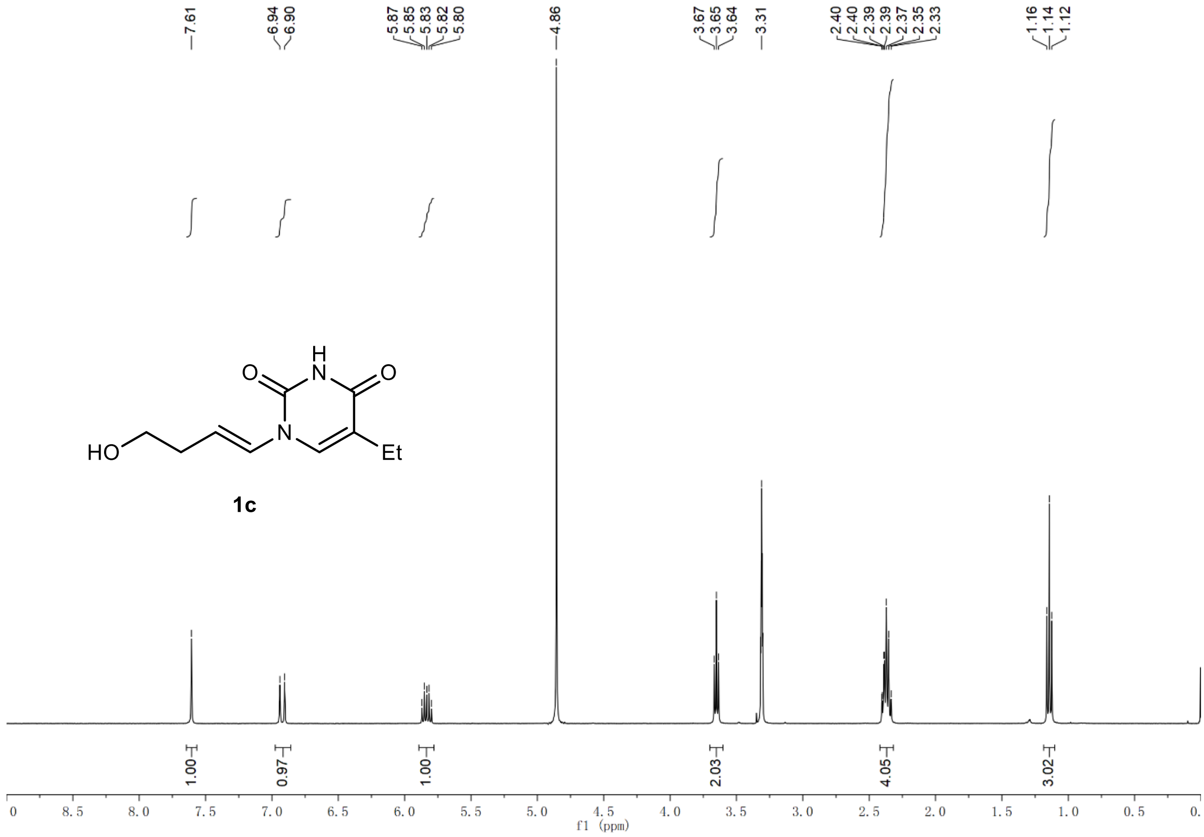

Supplementary Figure 22. ^{1}H NMR (400M, CD_3OD) of compound **1c.**

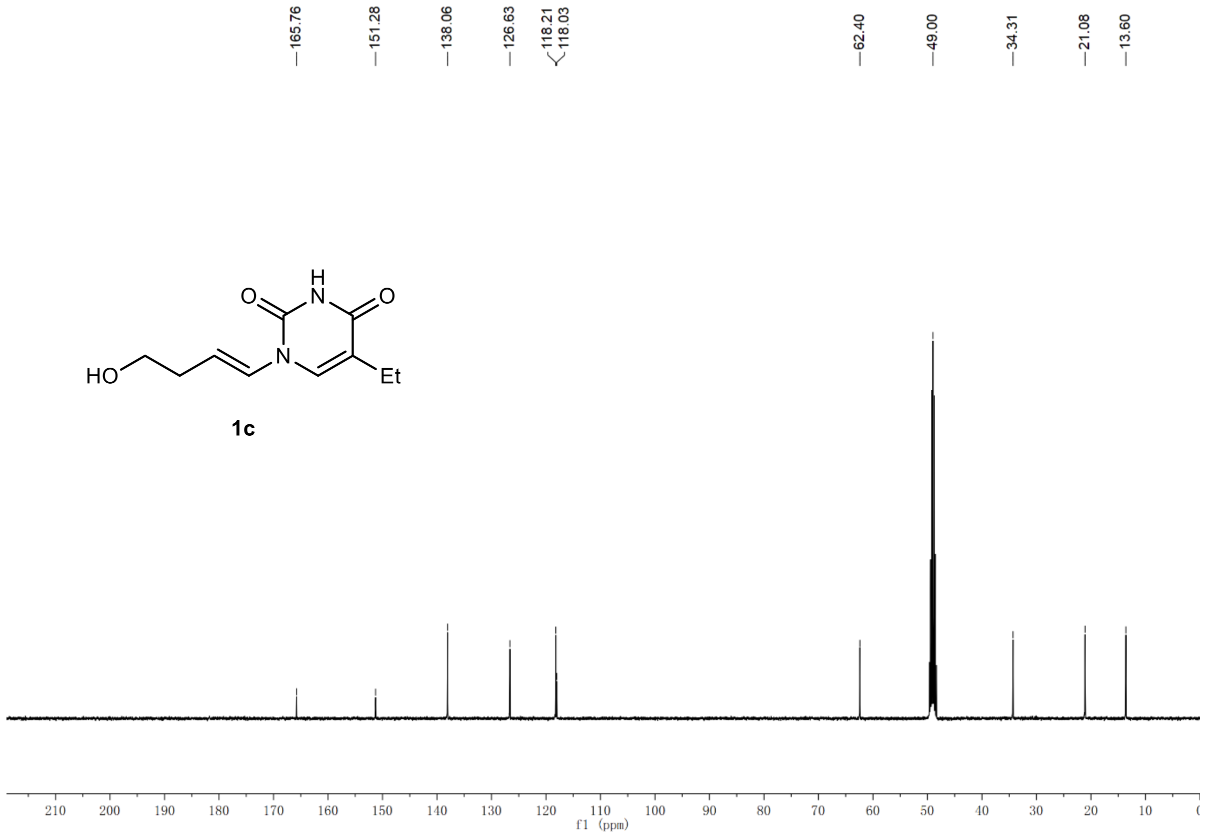

Supplementary Figure 23. ^{13}C NMR (100M, CD_3OD) of compound **1c.**

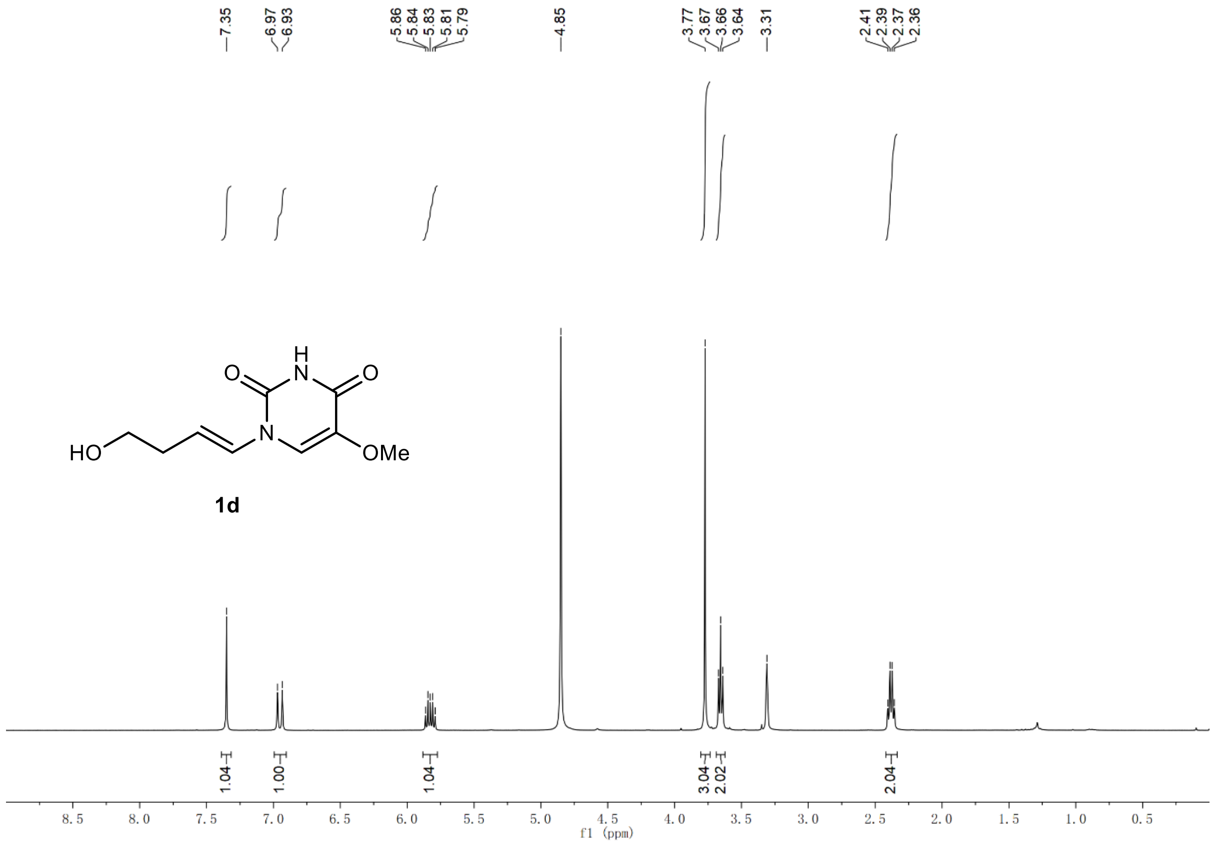

Supplementary Figure 24. ^{1}H NMR (400M, CD_3OD) of compound **1d.**

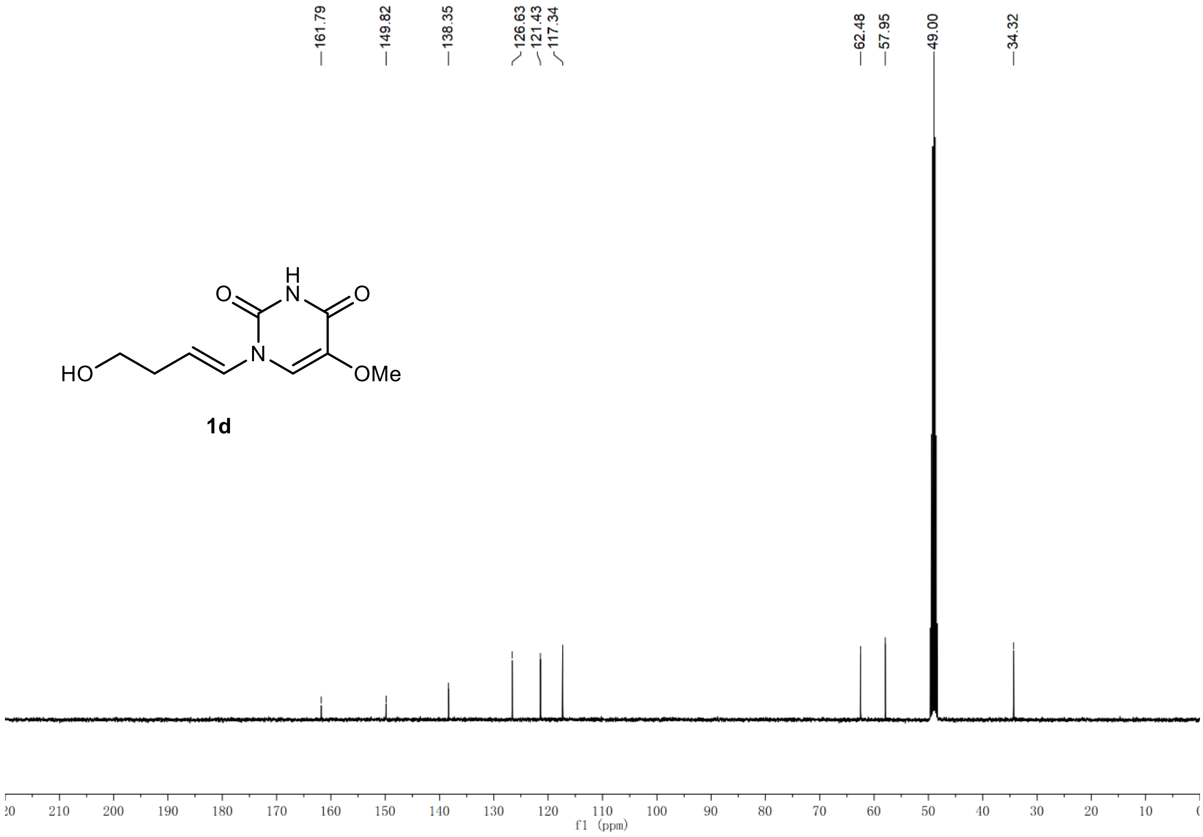

Supplementary Figure 25. ^{13}C NMR (100M, CD_3OD) of compound **1d.**

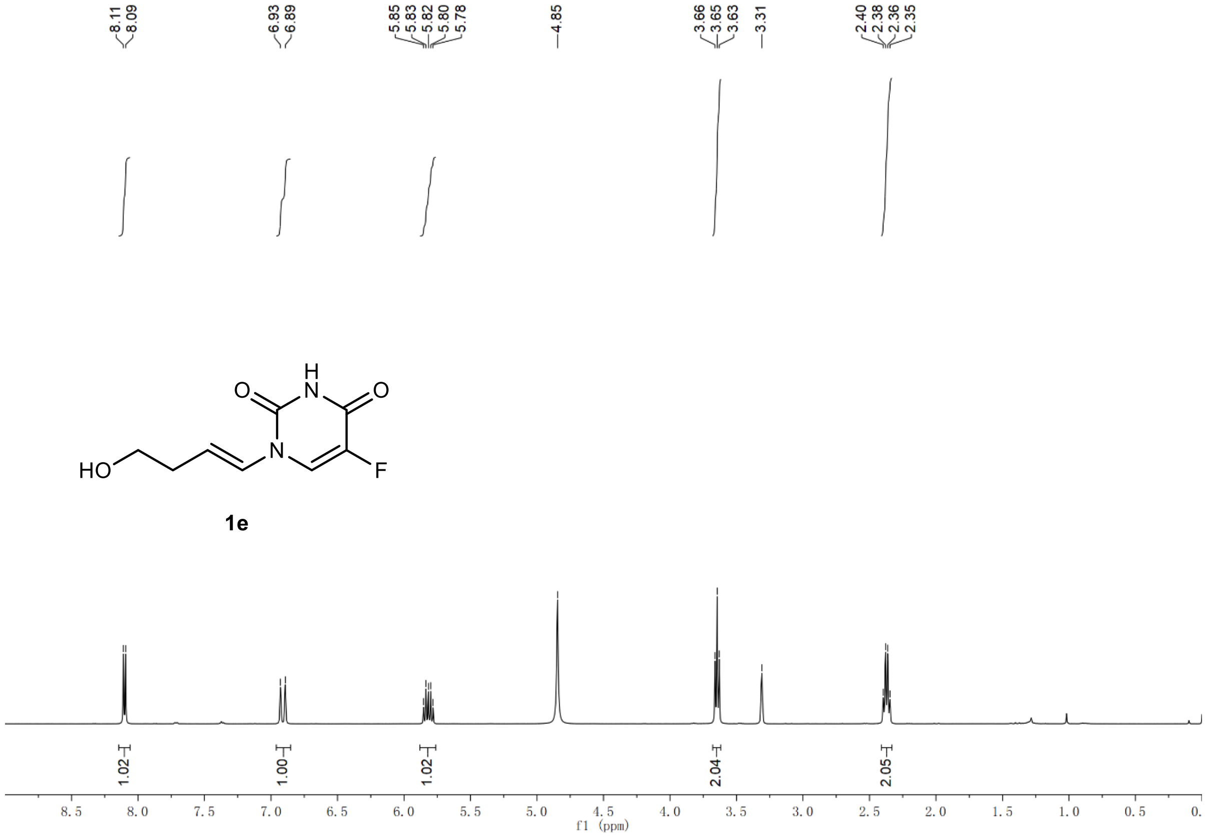

Supplementary Figure 26. ^{1}H NMR (400M, CD_3OD) of compound **1e.**

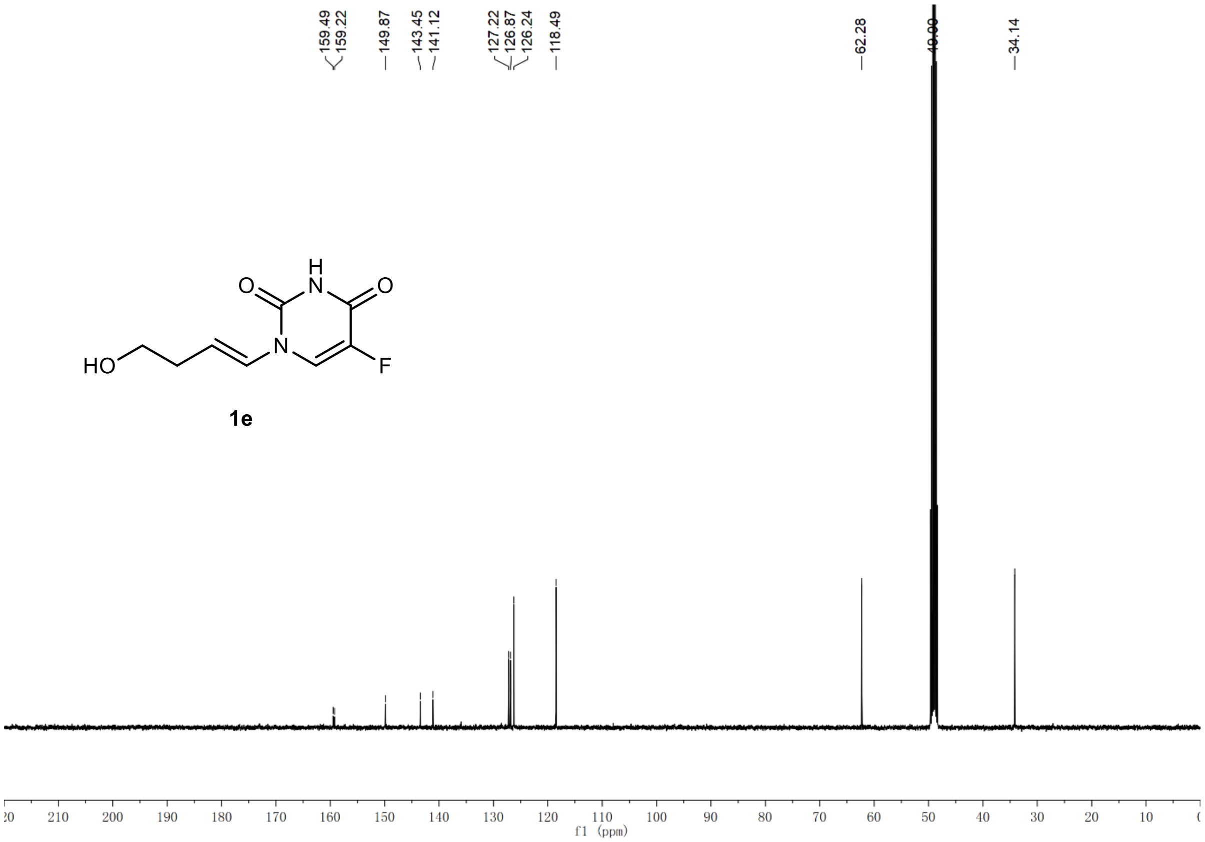

Supplementary Figure 27. ^{13}C NMR (100M, CD_3OD) of compound **1e.**

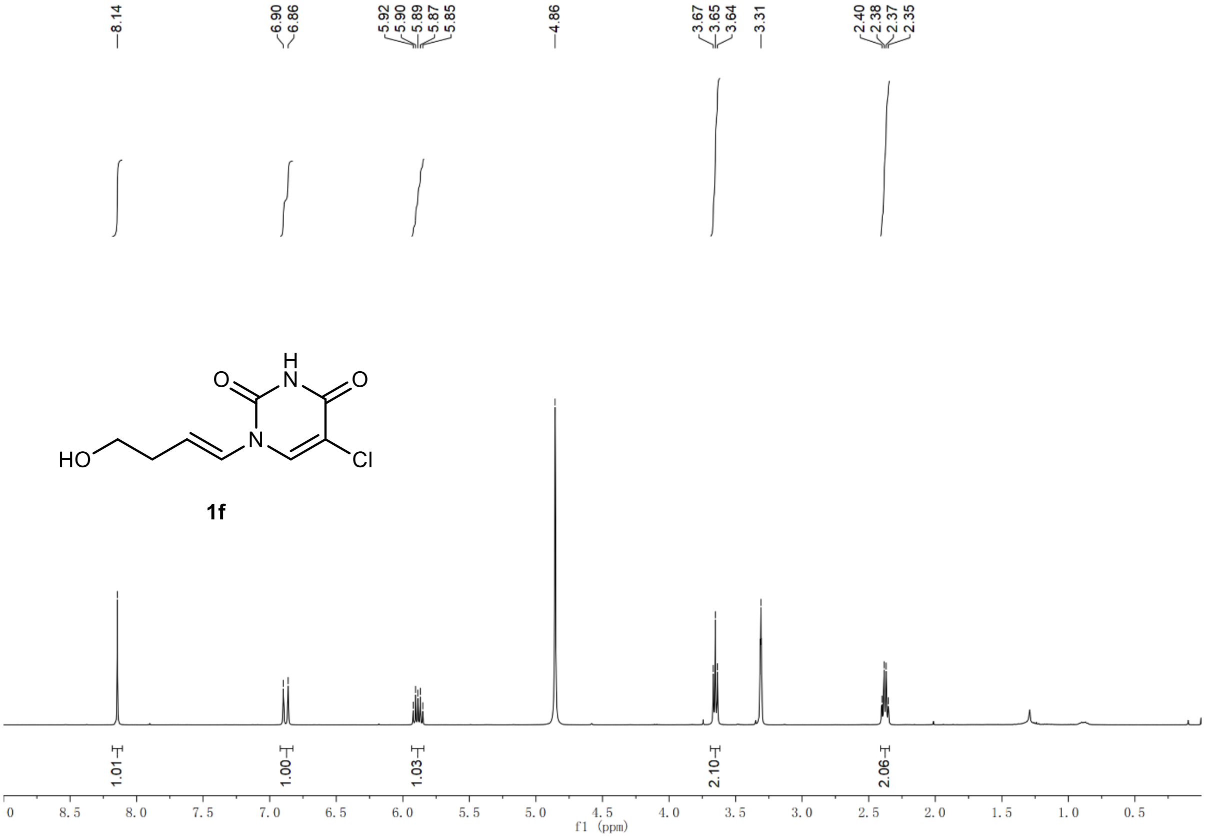

Supplementary Figure 28. ^{1}H NMR (400M, CD_3OD) of compound **1f.**

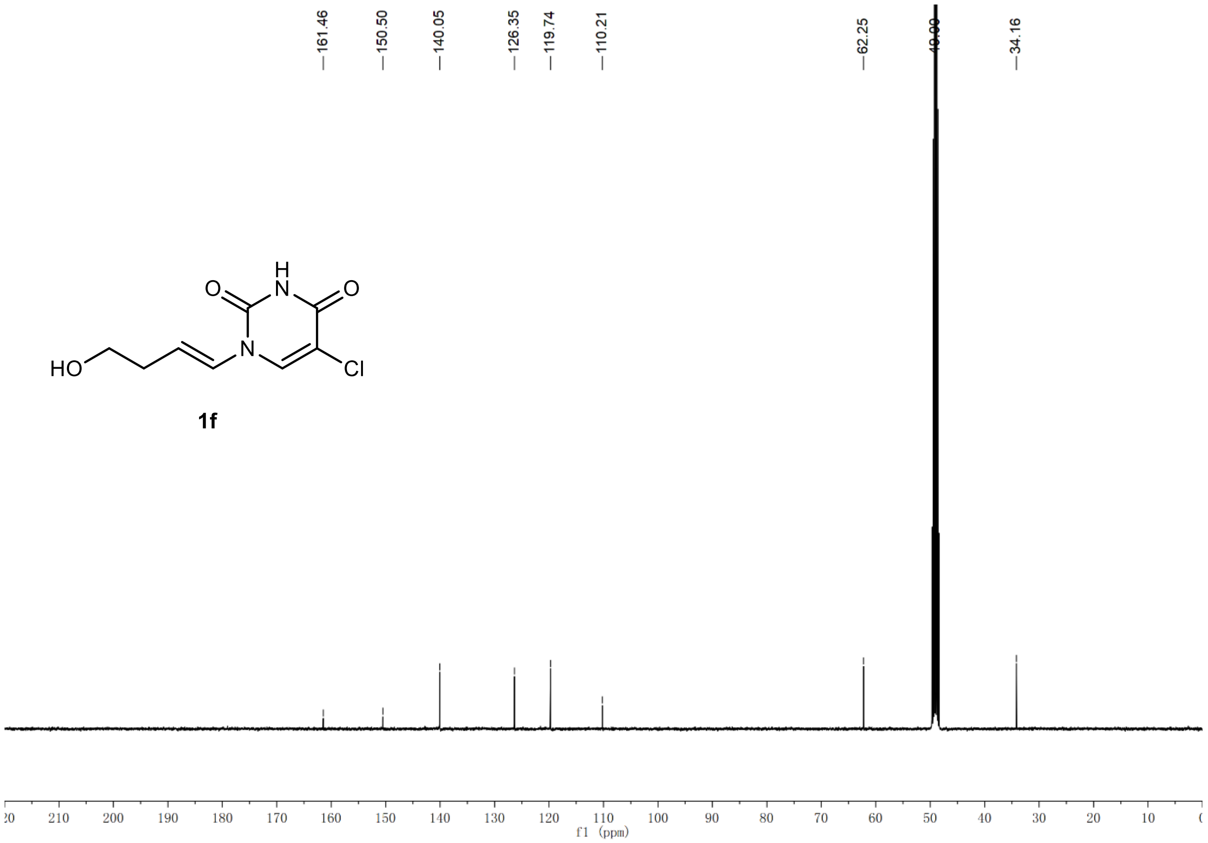

Supplementary Figure 29. ^{13}C NMR (100M, CD_3OD) of compound **1f.**

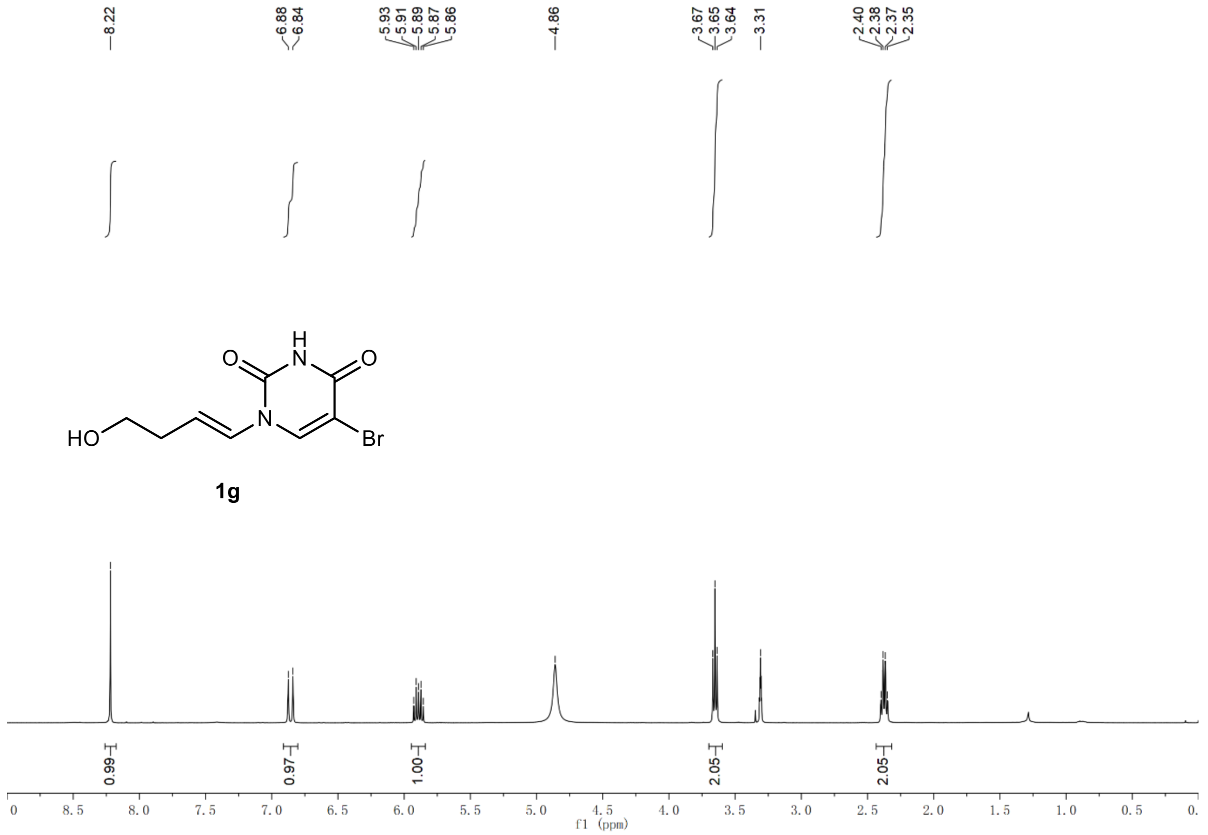

Supplementary Figure 30. ^{1}H NMR (400M, CD_3OD) of compound **1g.**

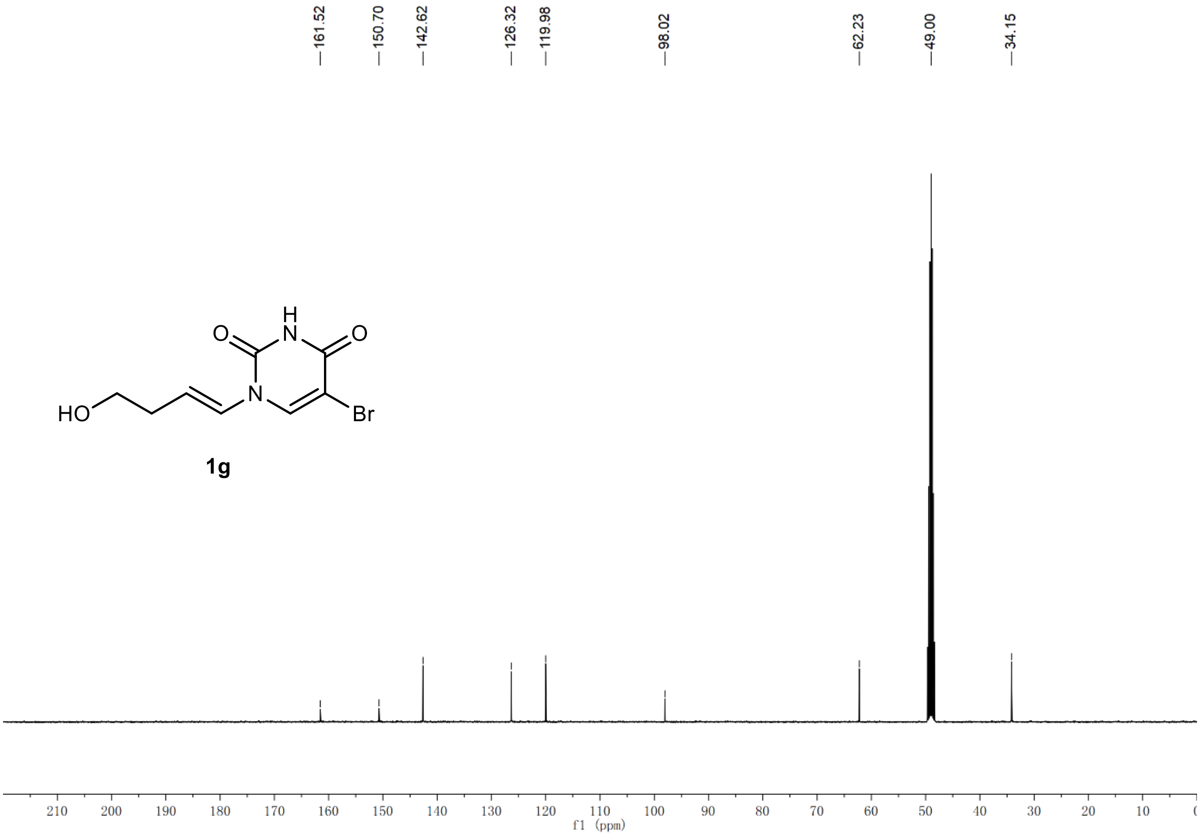

Supplementary Figure 31. ^{13}C NMR (100M, CD_3OD) of compound **1g.**

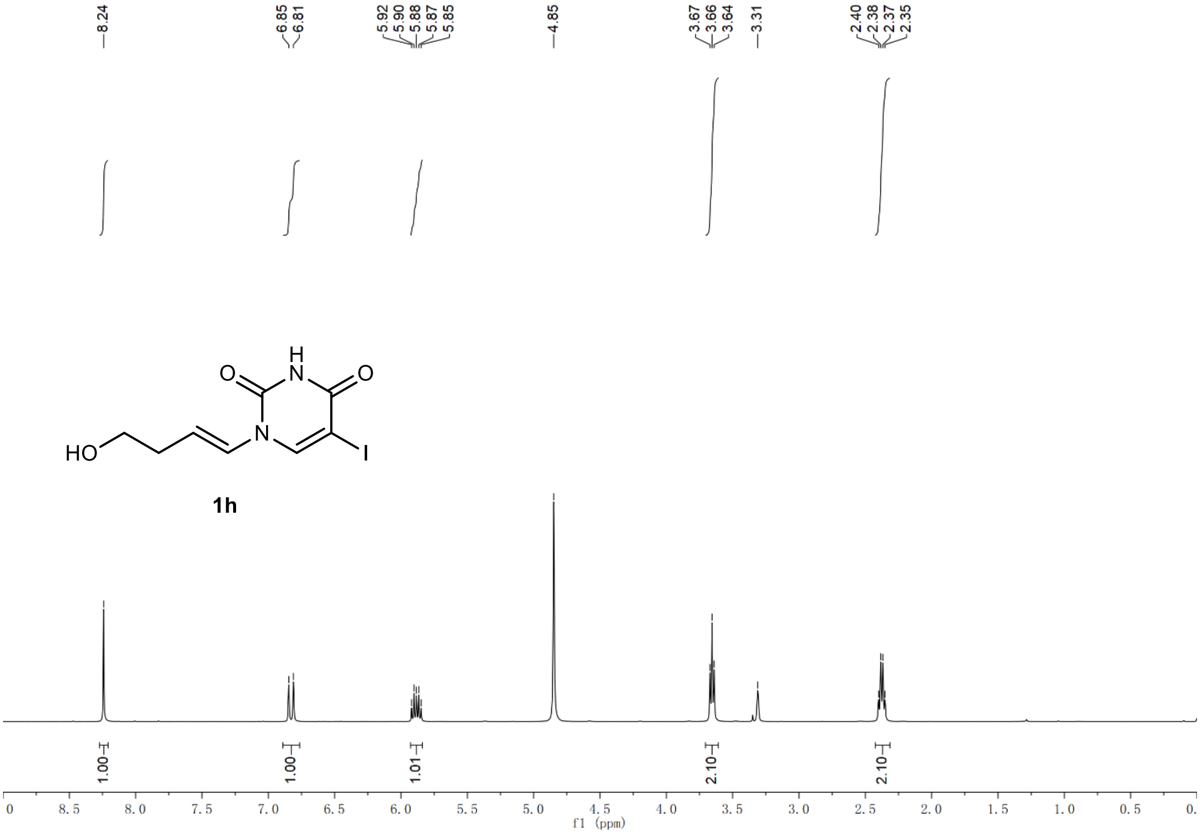

Supplementary Figure 32. ^{1}H NMR (400M, CD_3OD) of compound **1h.**

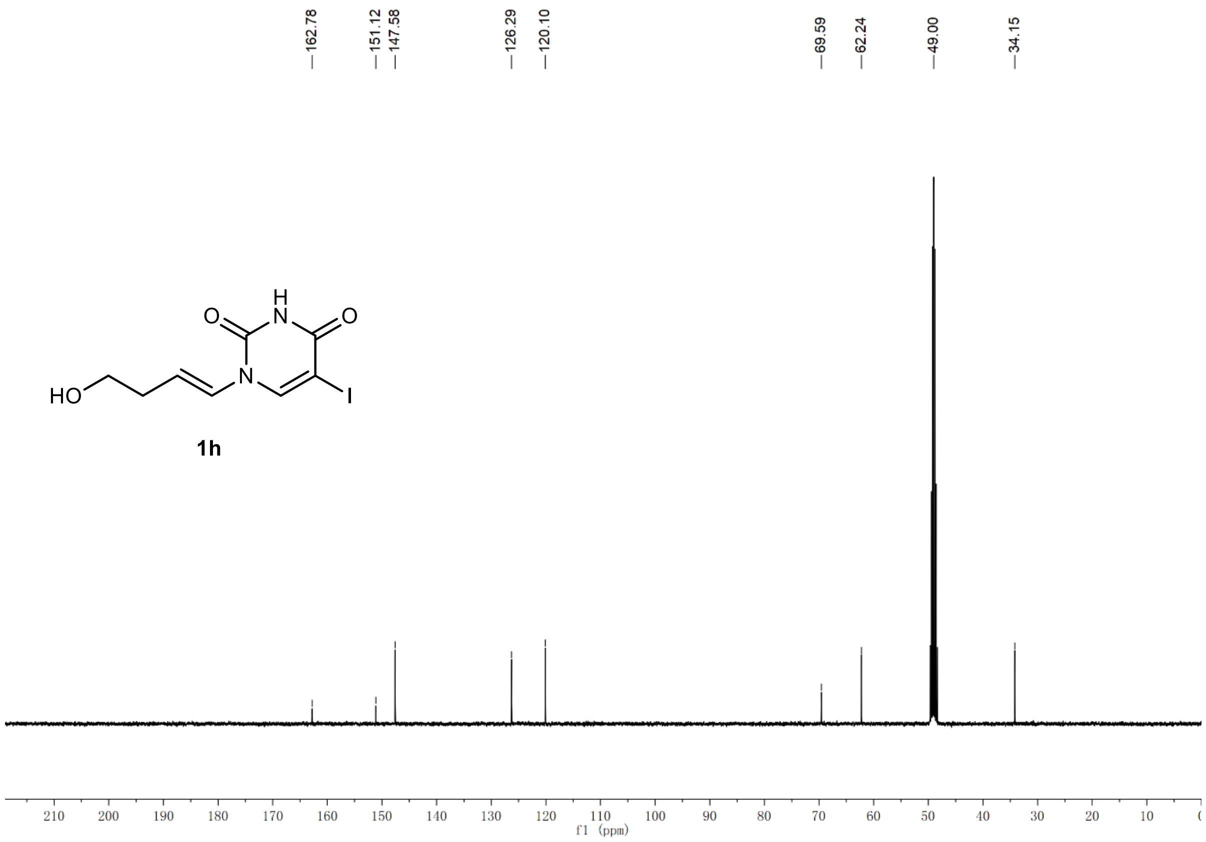

Supplementary Figure 33. ^{13}C NMR (100M, CD_3OD) of compound **1h.**

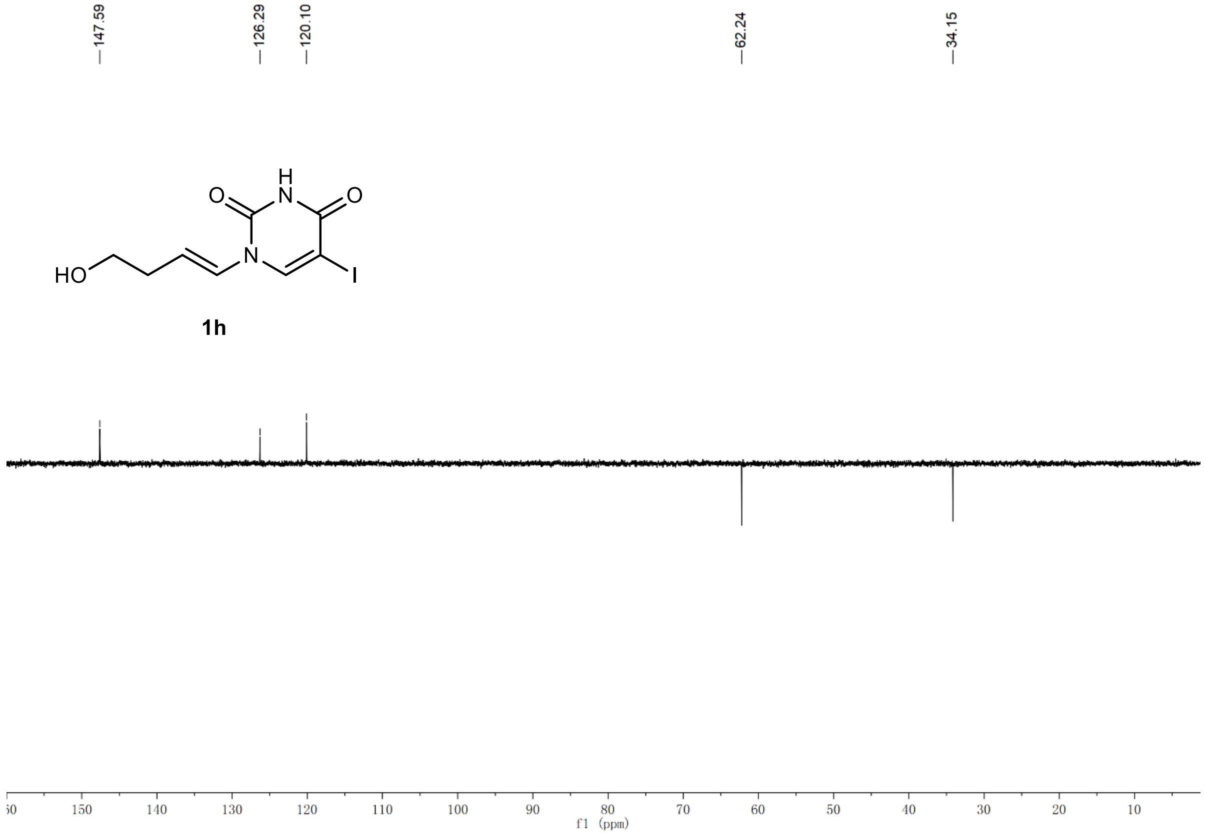

Supplementary Figure 34. DEPT 135° (100M, CD_3OD) of compound **1h.**

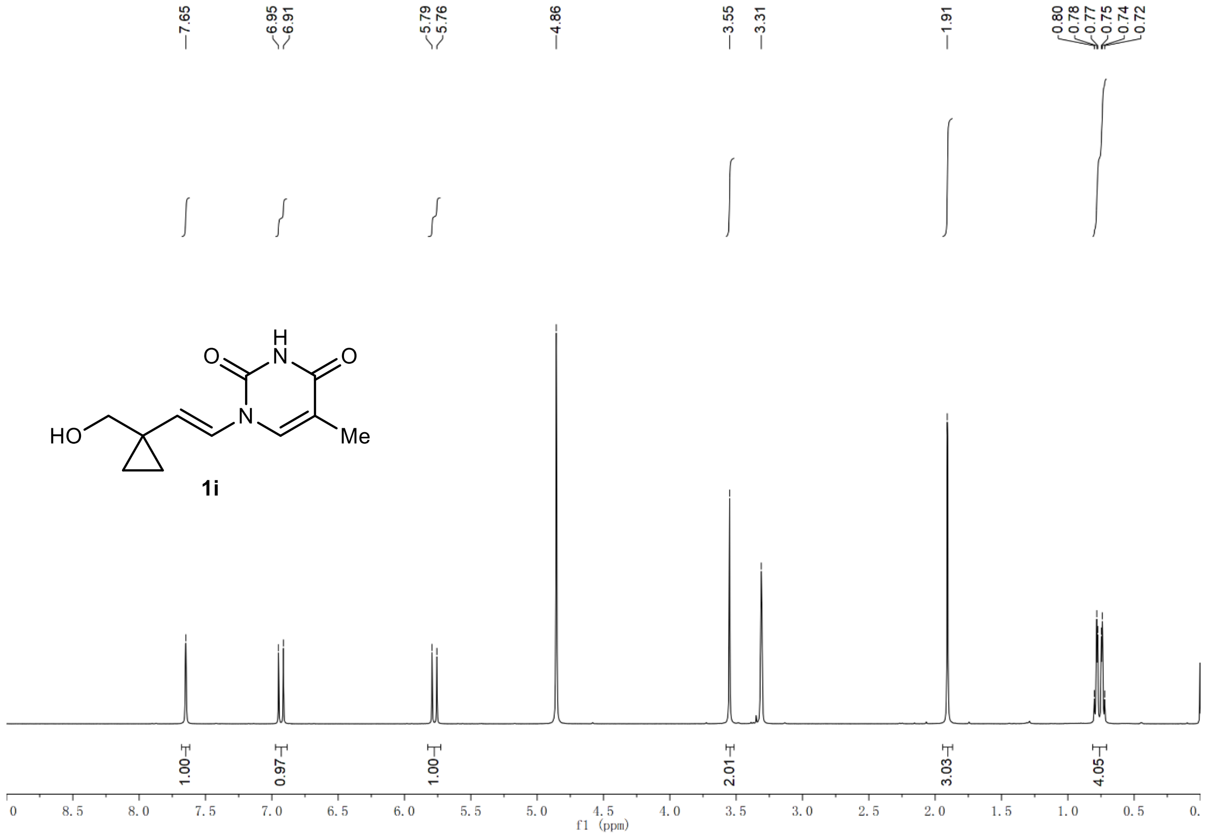

Supplementary Figure 35. ^{1}H NMR (400M, CD_3OD) of compound **1i.**

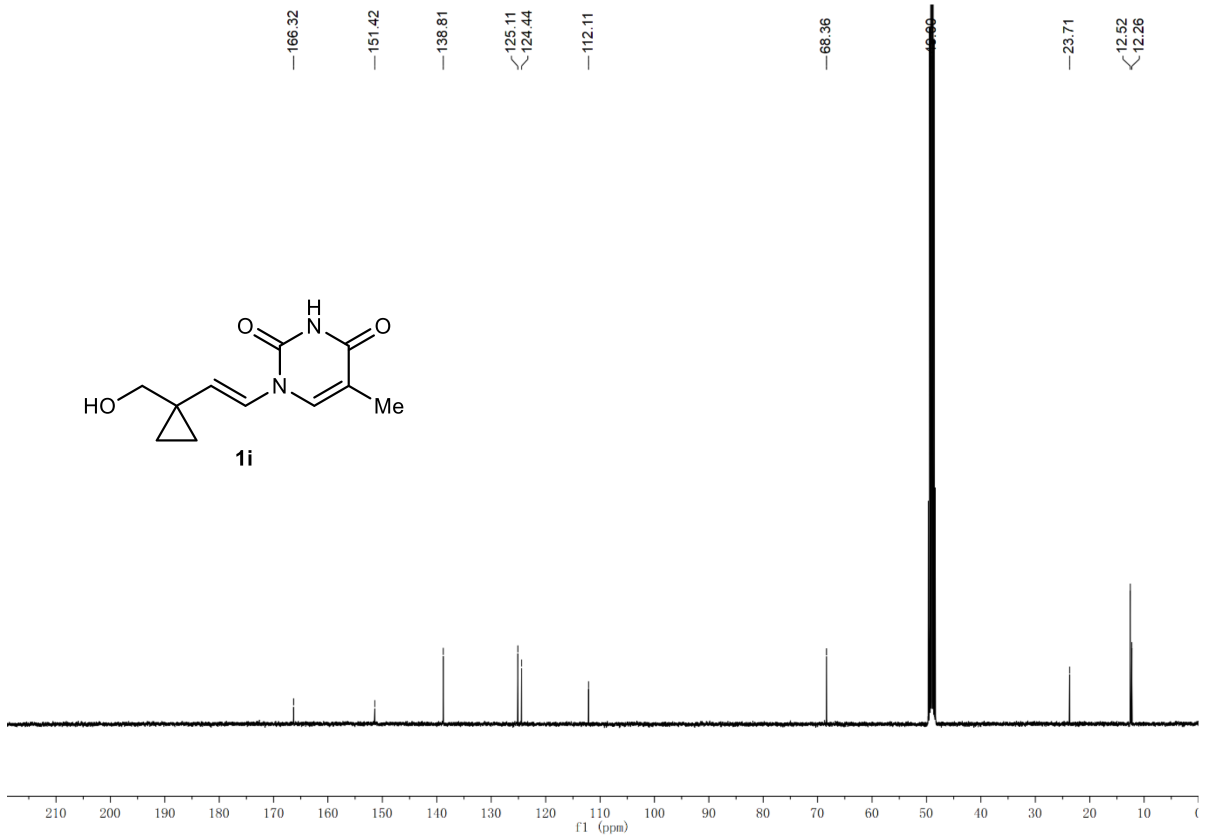

Supplementary Figure 36. ^{13}C NMR (100M, CD_3OD) of compound **1i.**

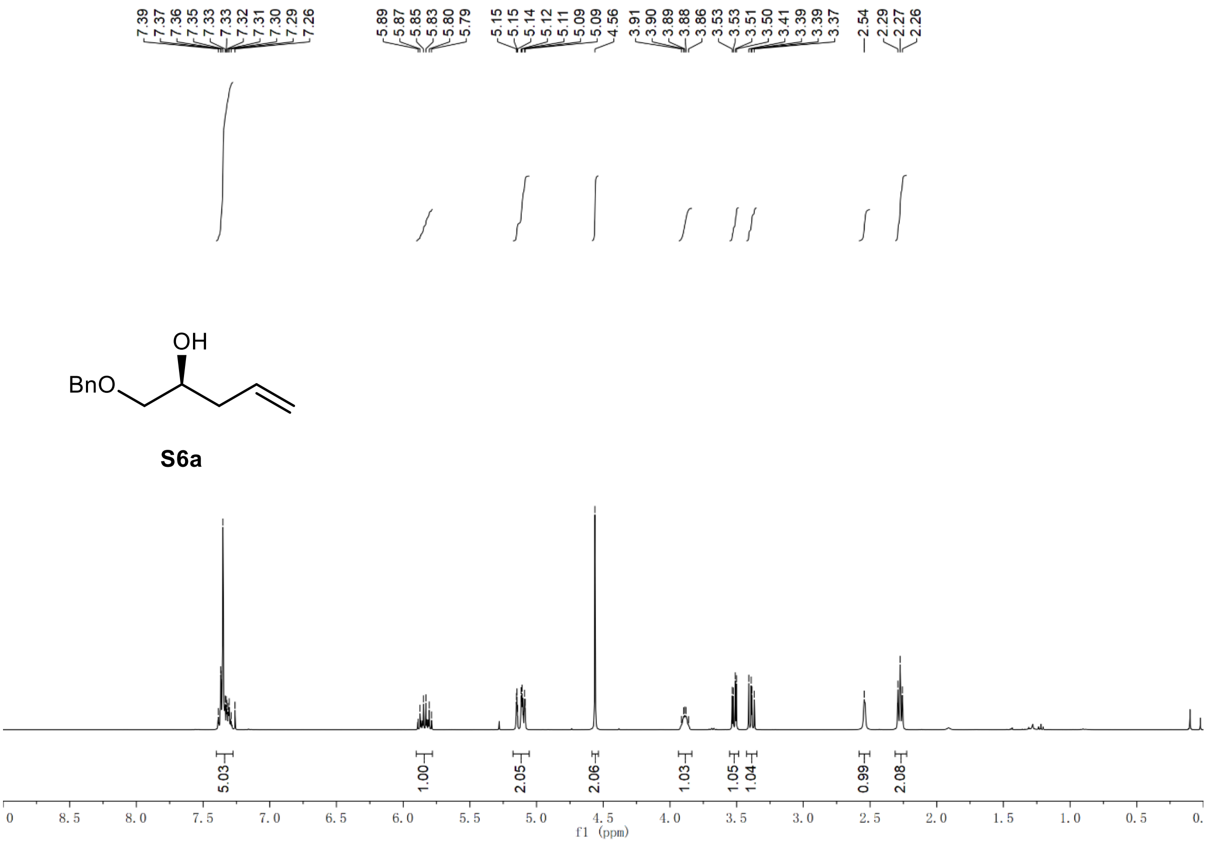

Supplementary Figure 37. ^{1}H NMR (400M, $CDCl_3$) of compound **S6a.**

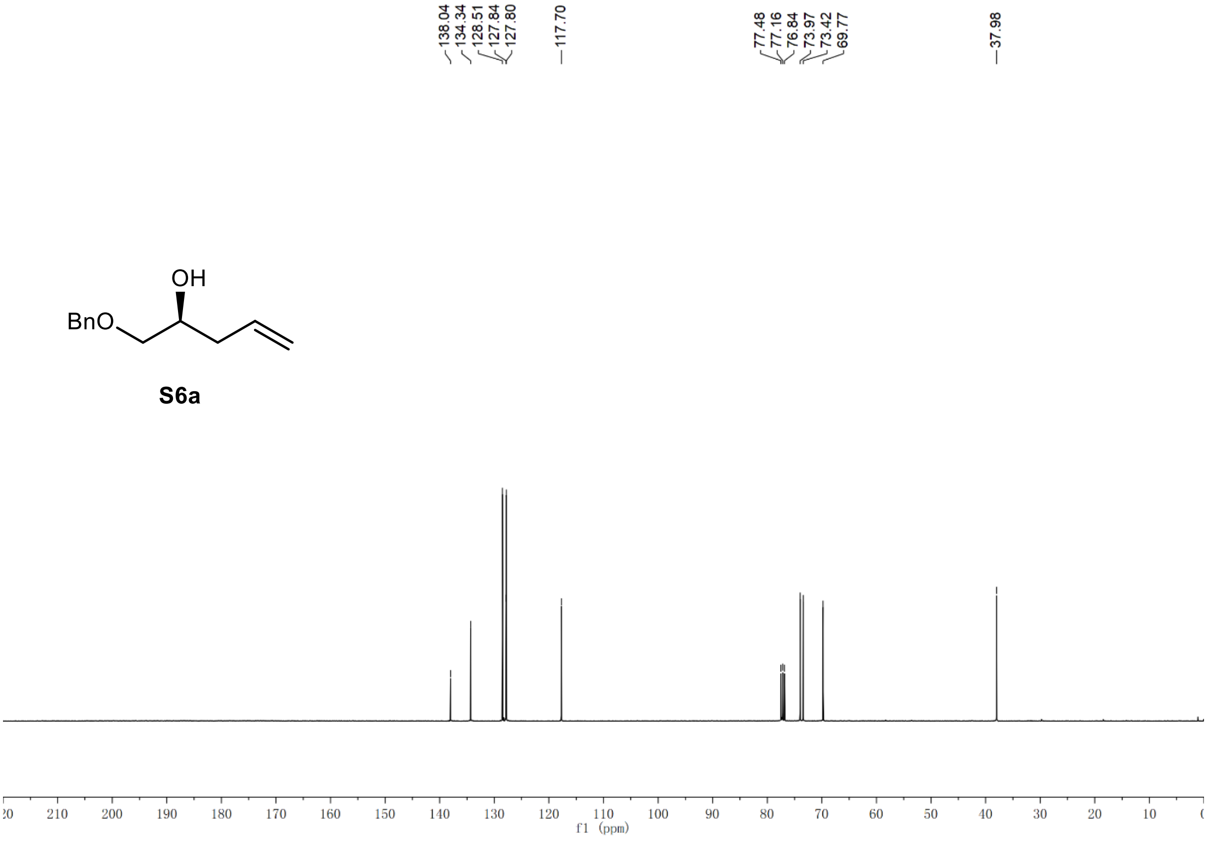

Supplementary Figure 38. ^{13}C NMR (100M, $CDCl_3$) of compound **S6a.**

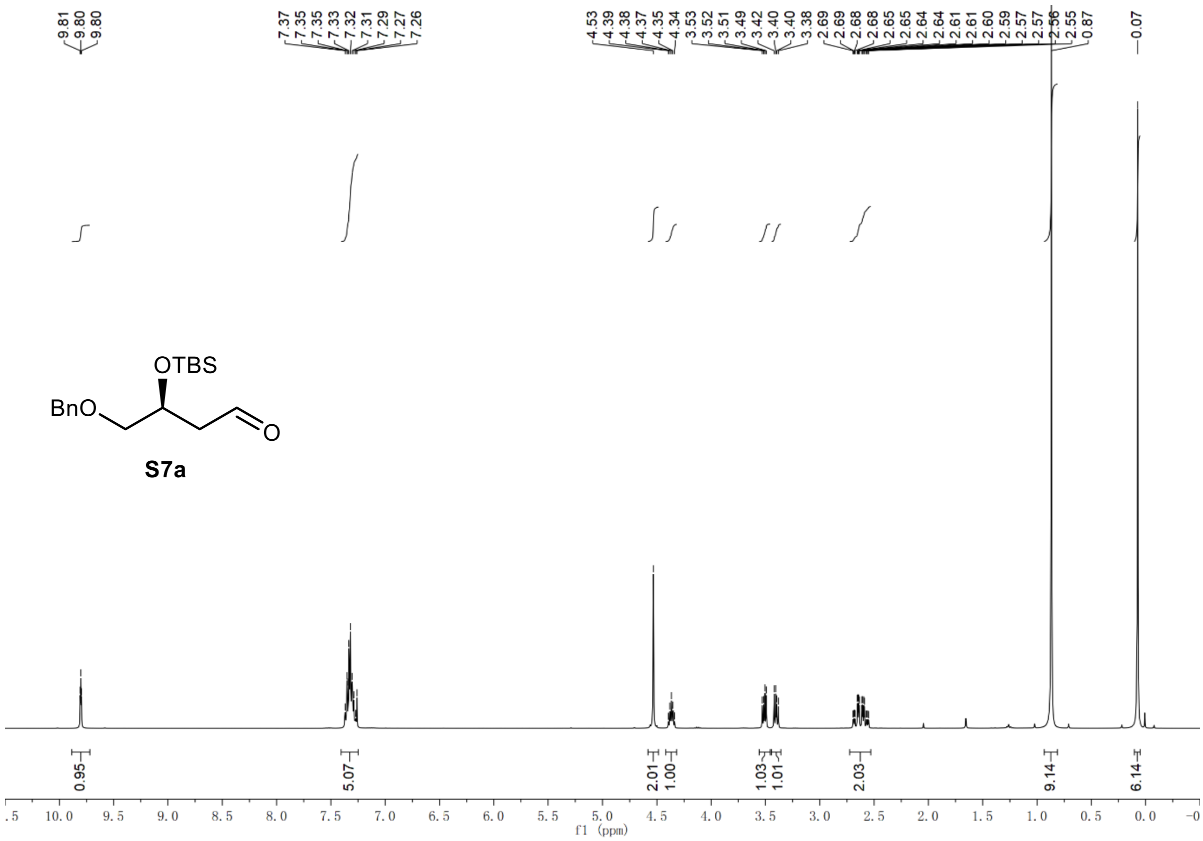

Supplementary Figure 39. ^{1}H NMR (400M, $CDCl_3$) of compound **S7a.**

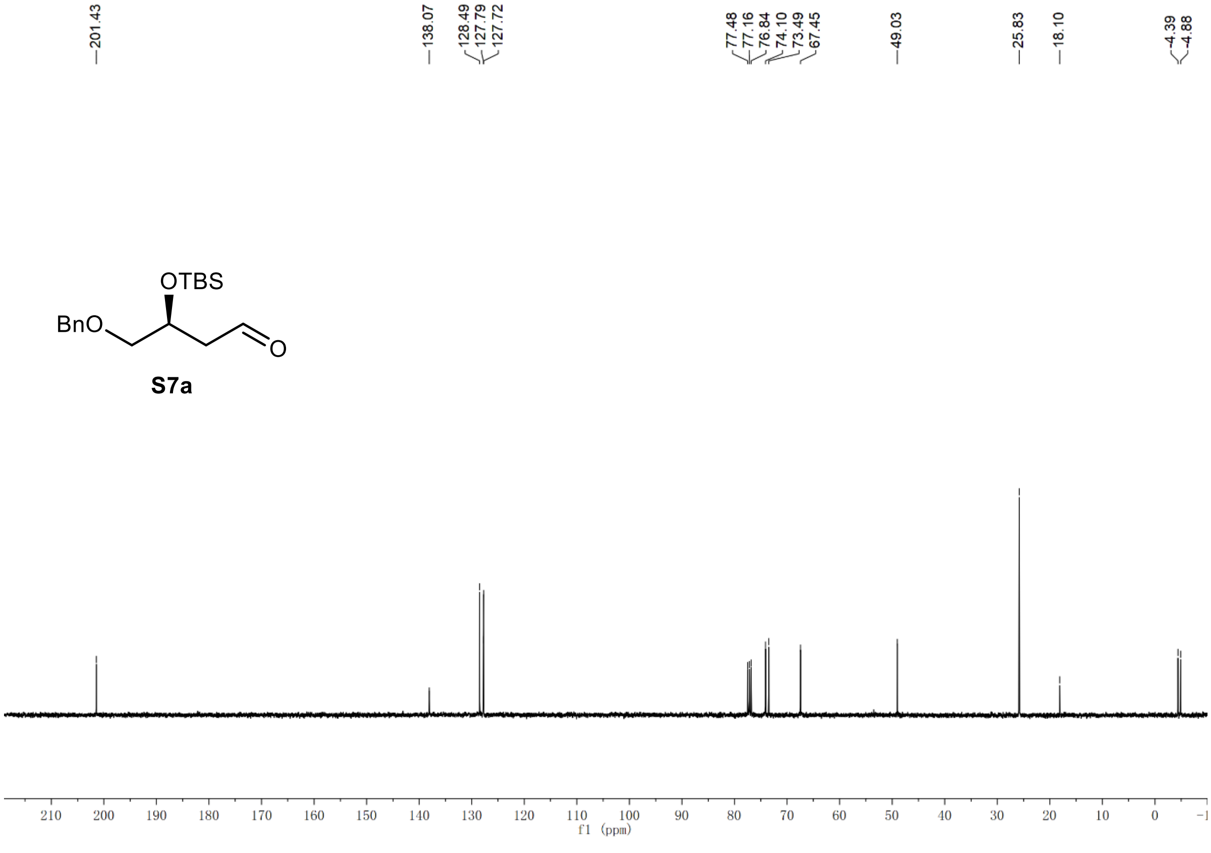

Supplementary Figure 40. ^{13}C NMR (100M, $CDCl_3$) of compound **S7a.**

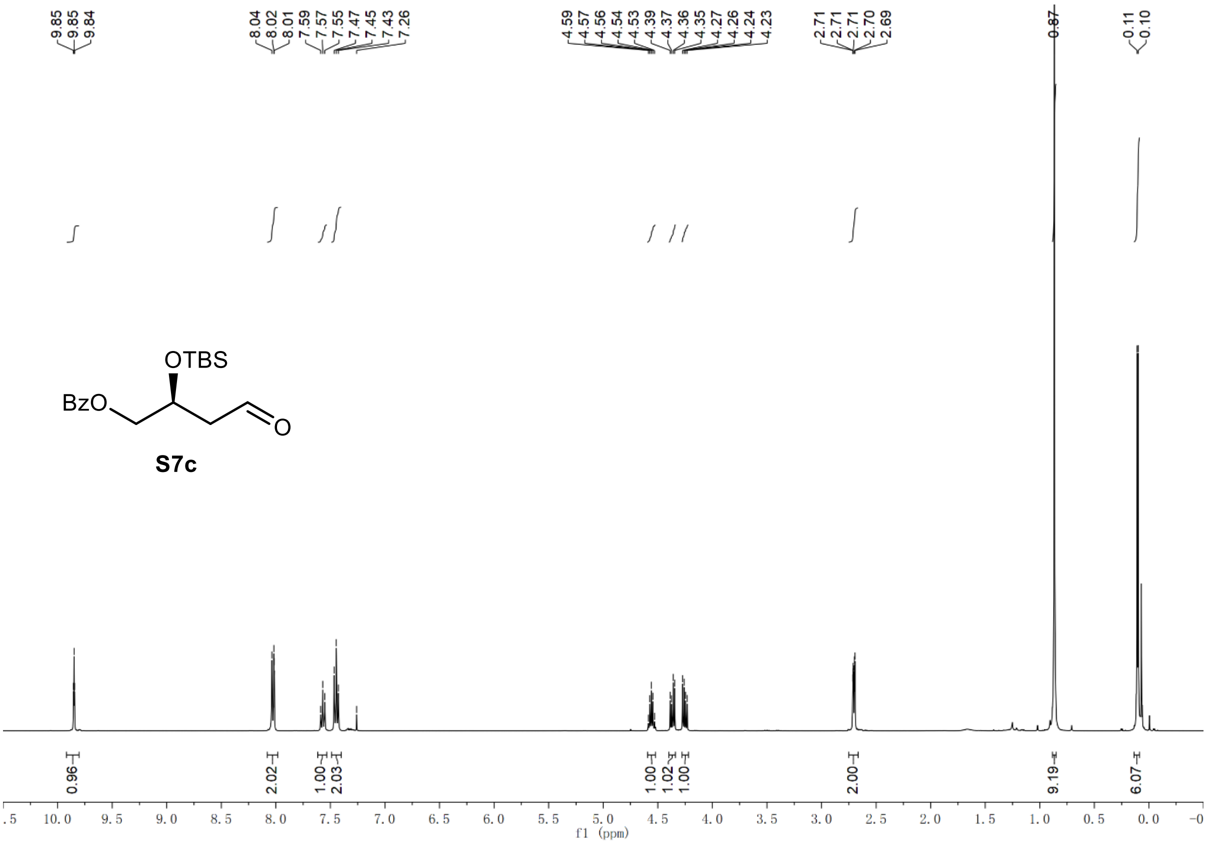

Supplementary Figure 41. ^{1}H NMR (400M, $CDCl_3$) of compound **S7c.**

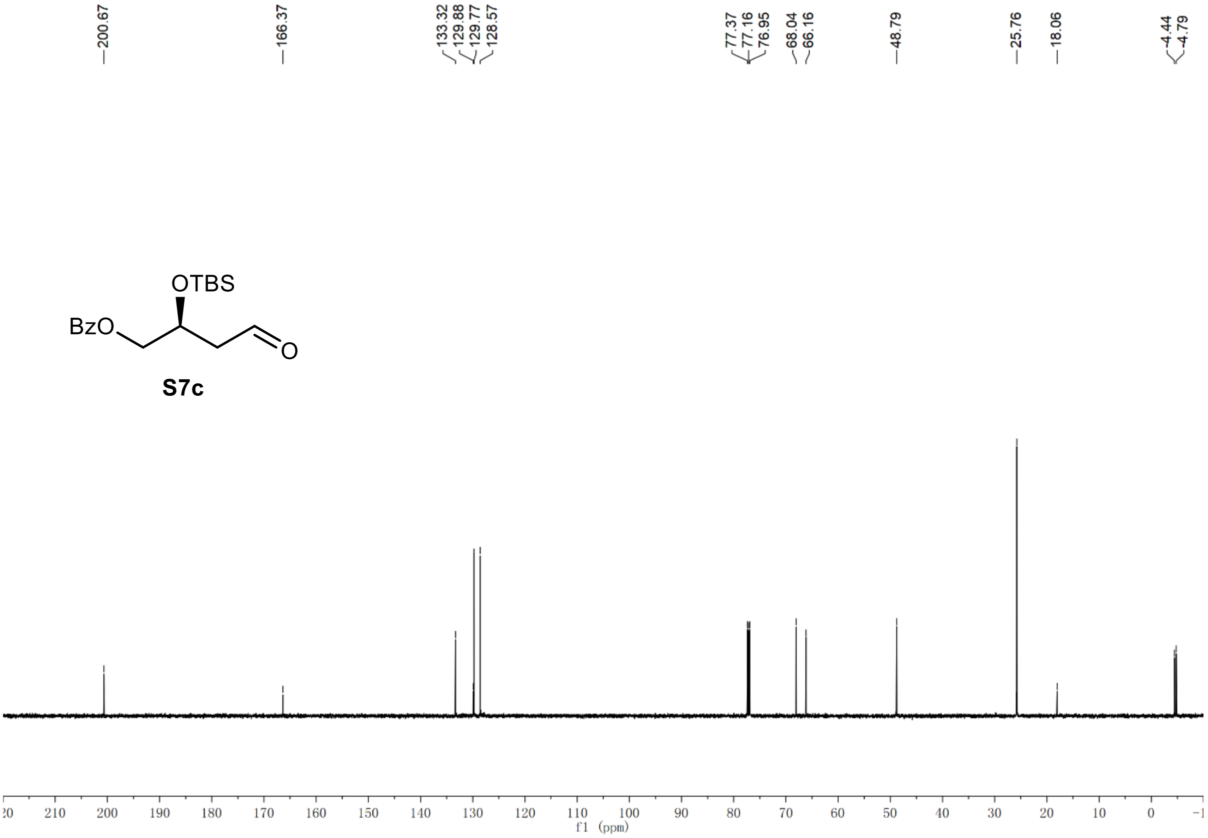

Supplementary Figure 42. ^{13}C NMR (100M, $CDCl_3$) of compound **S7c.**

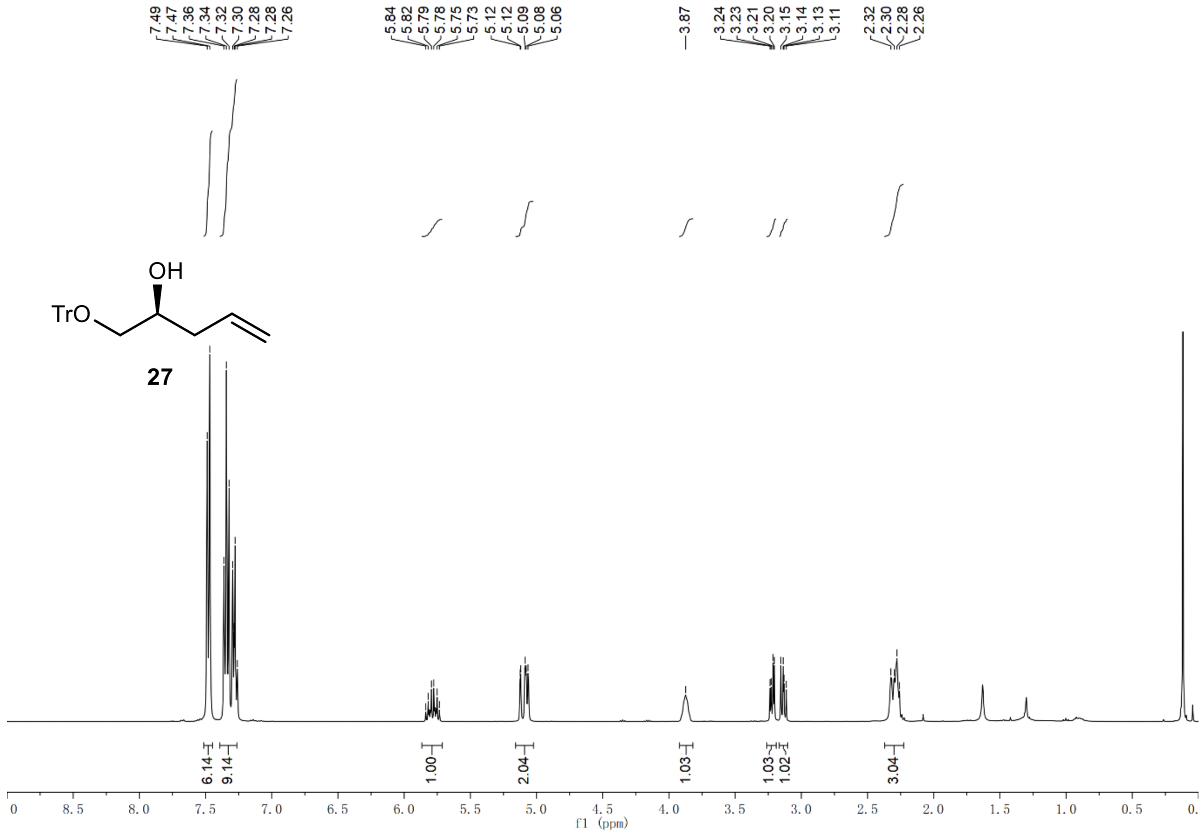

Supplementary Figure 43. ^{1}H NMR (400M, $CDCl_3$) of compound **27.**

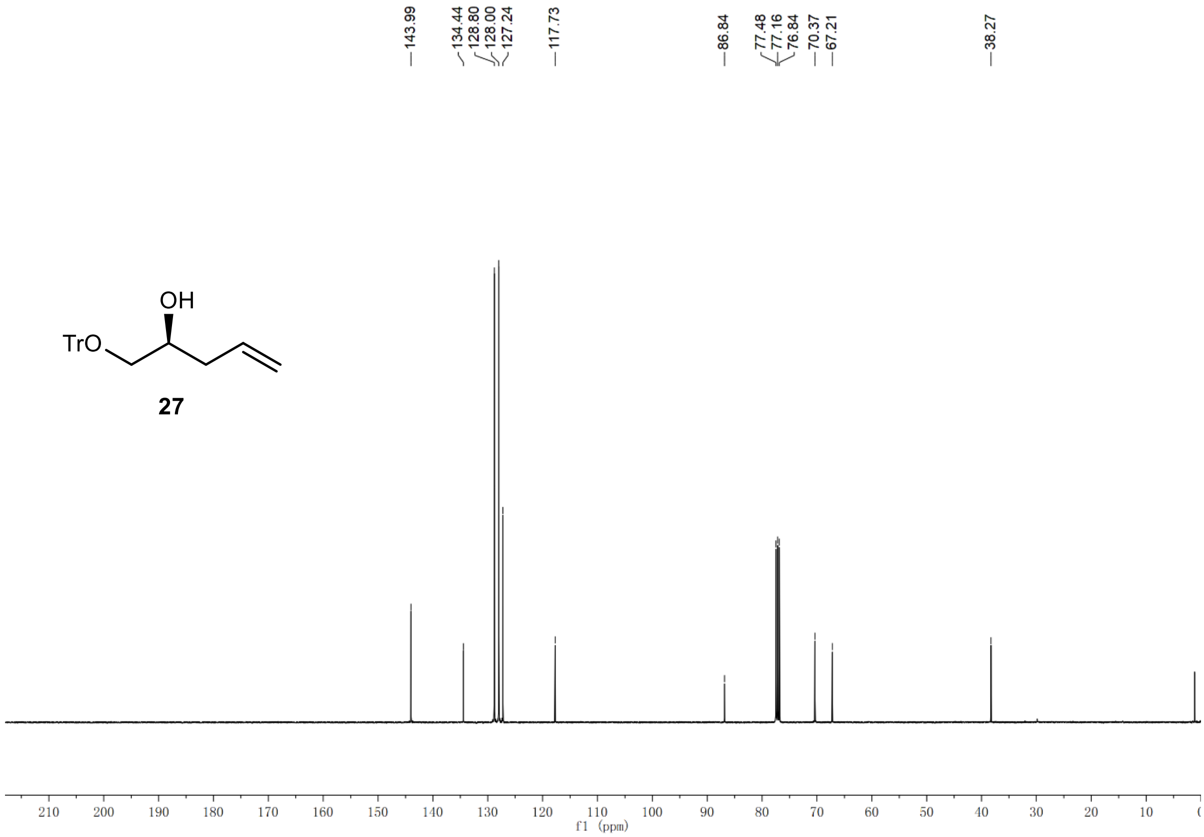

Supplementary Figure 44. ^{13}C NMR (100M, $CDCl_3$) of compound **27.**

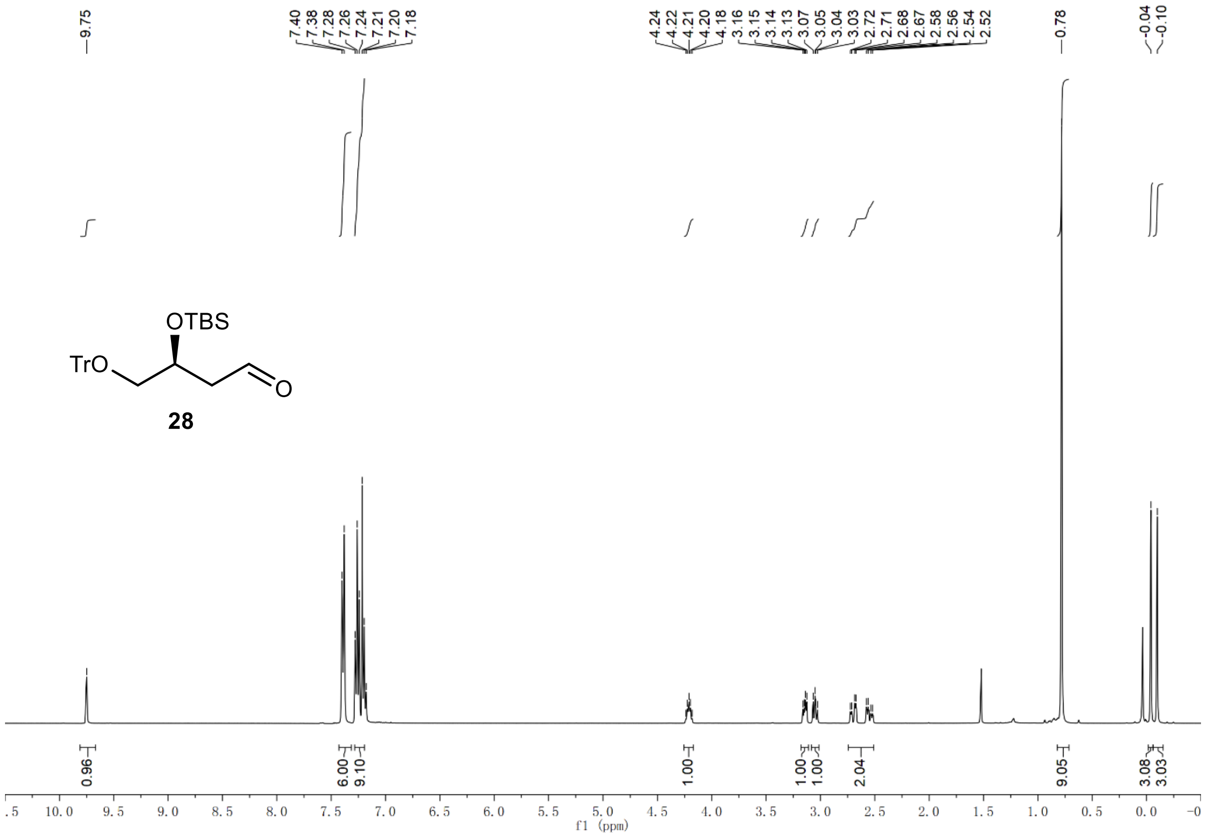

Supplementary Figure 45. ^{1}H NMR (400M, $CDCl_3$) of compound **28.**

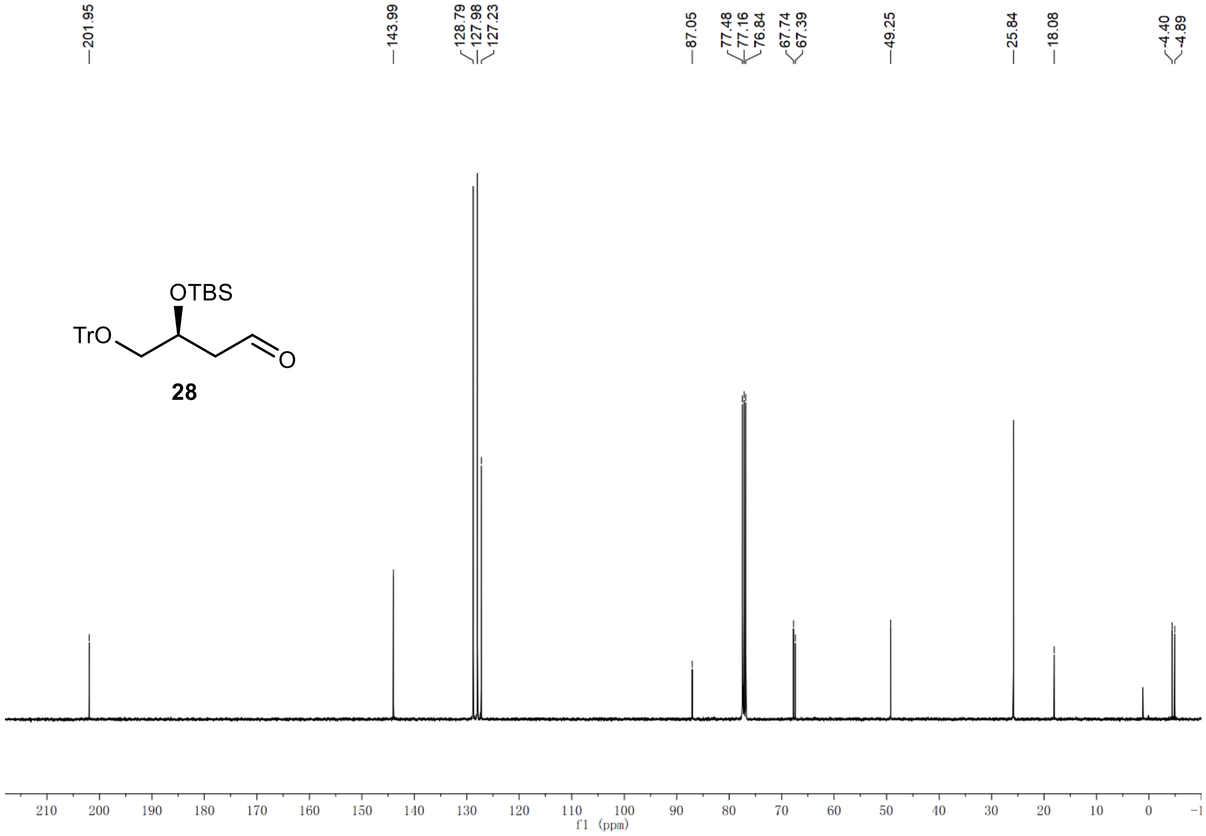

Supplementary Figure 46. ^{13}C NMR (100M, $CDCl_3$) of compound **28.**

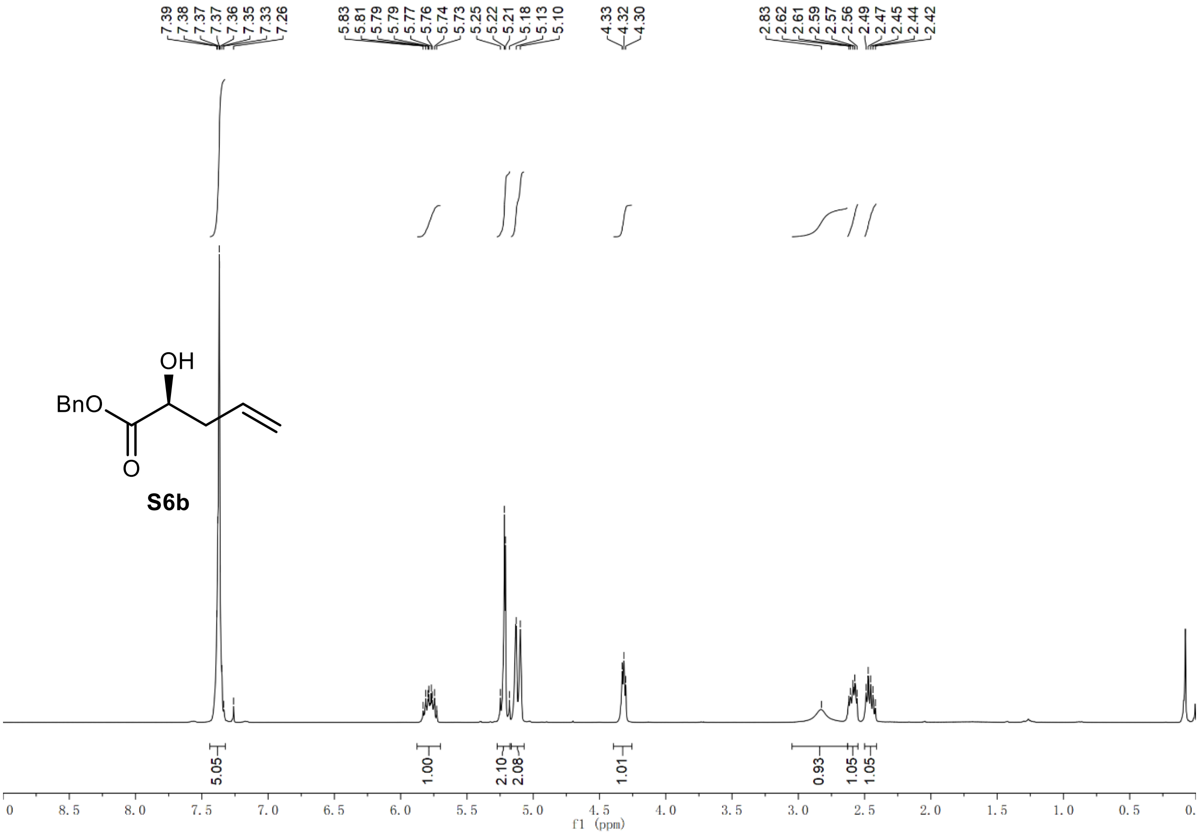

Supplementary Figure 47. ^{1}H NMR (400M, $CDCl_3$) of compound **S6b.**

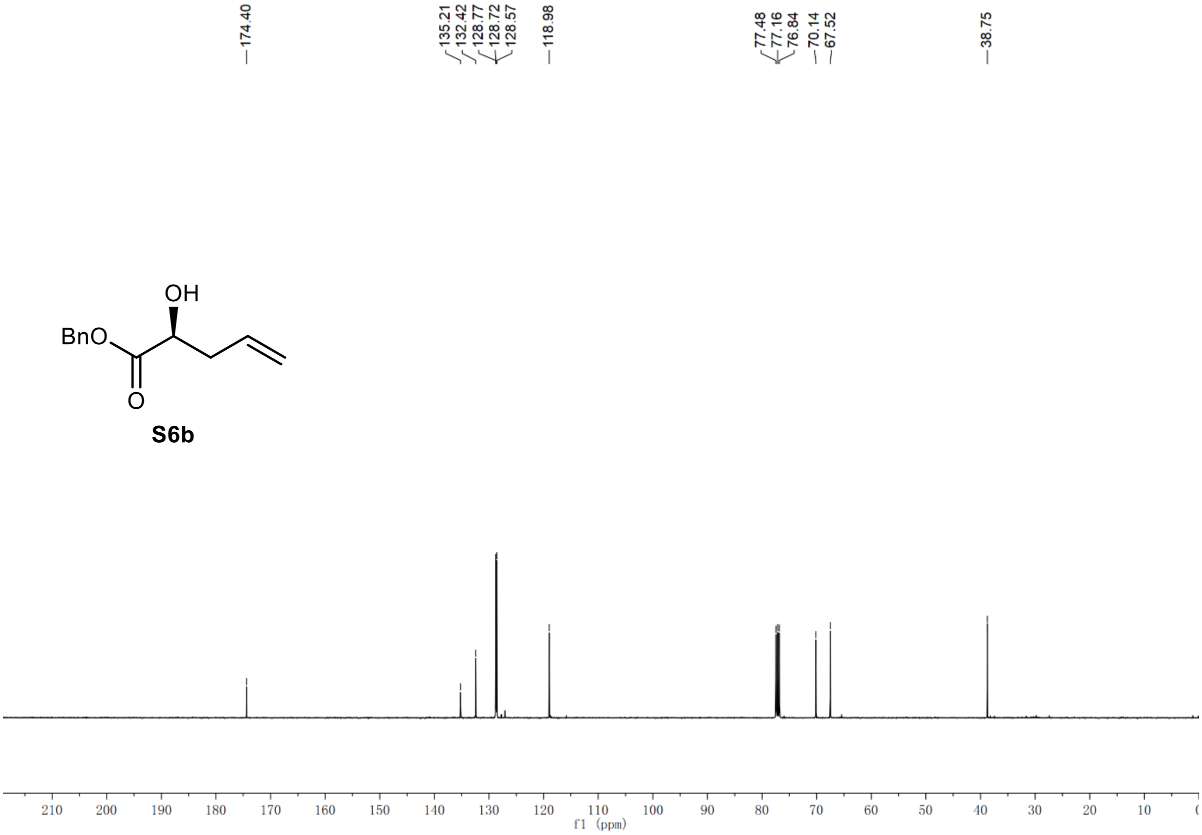

Supplementary Figure 48. ^{13}C NMR (100M, $CDCl_3$) of compound **S6b.**

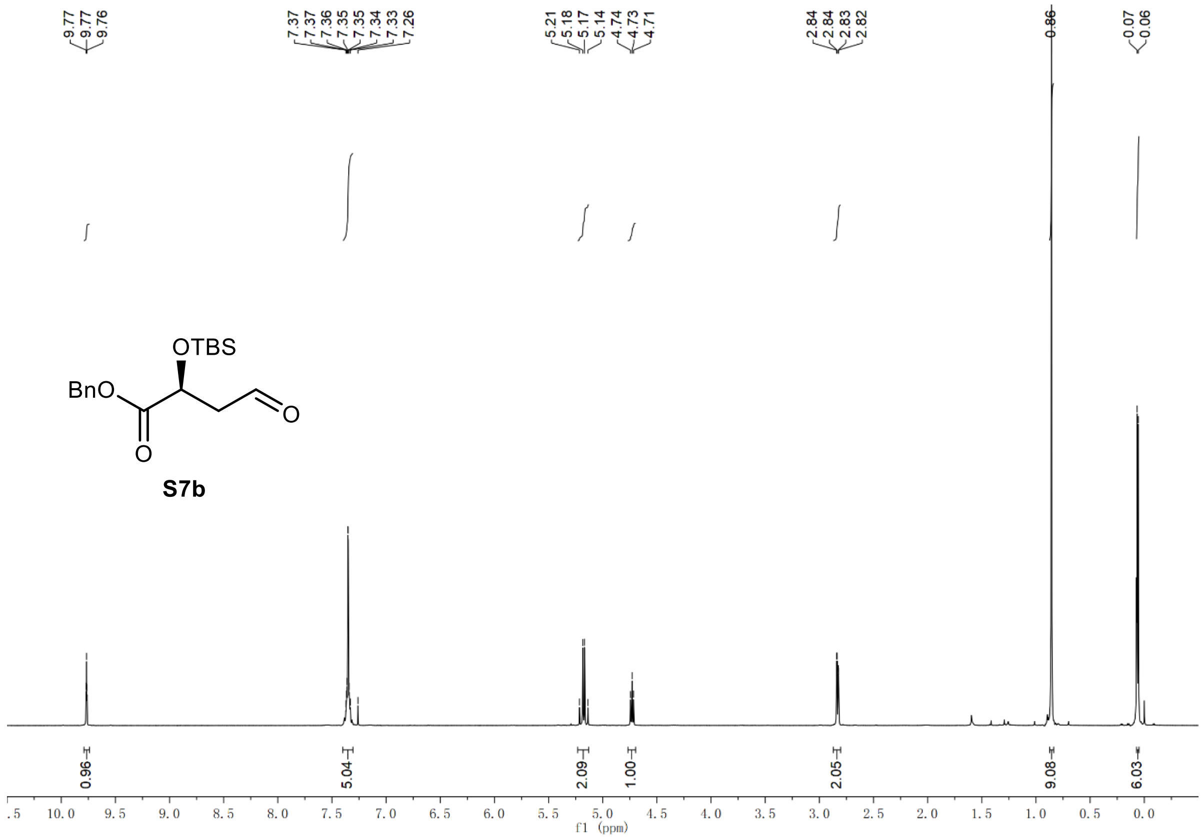

Supplementary Figure 49. ^{1}H NMR (400M, $CDCl_3$) of compound **S7b.**

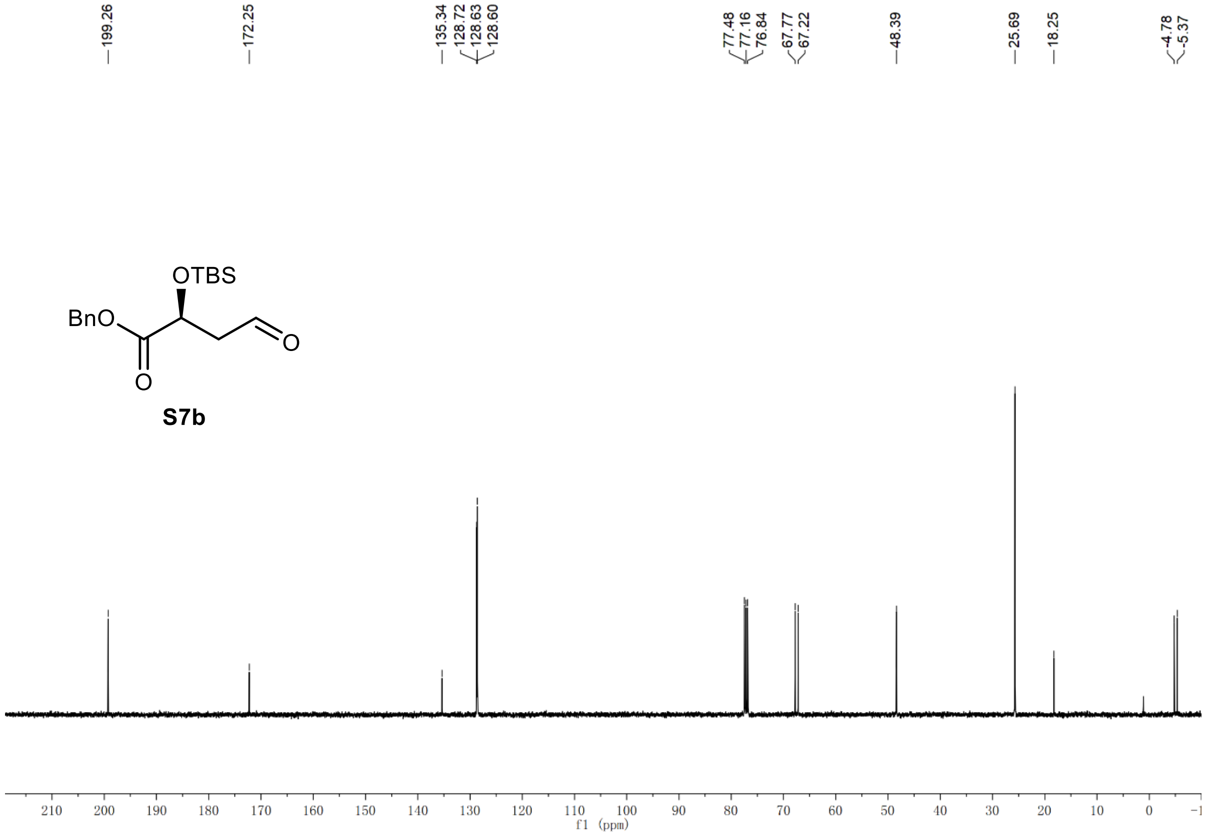

Supplementary Figure 50. ^{13}C NMR (100M, $CDCl_3$) of compound **S7b.**

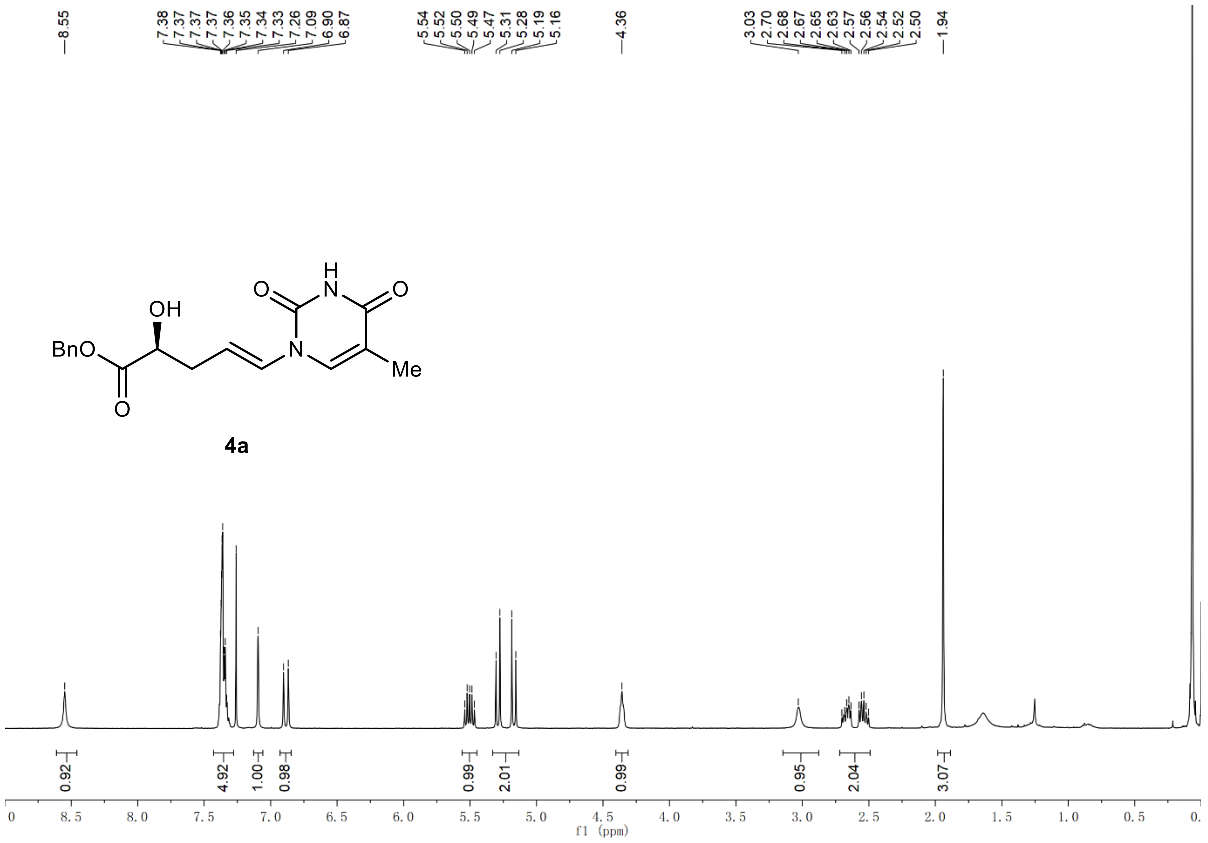

Supplementary Figure 51. ^{1}H NMR (400M, $CDCl_3$) of compound **4a.**

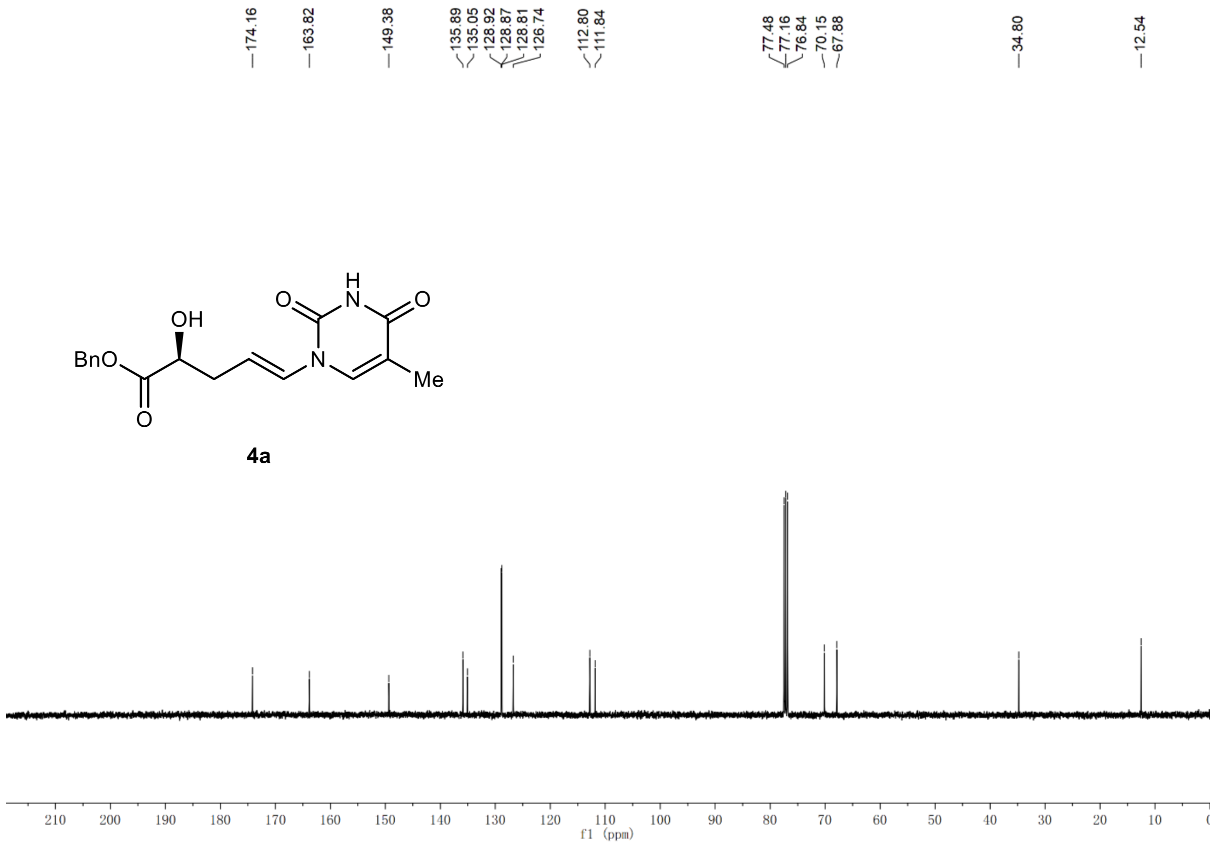

Supplementary Figure 52. ^{13}C NMR (100M, $CDCl_3$) of compound **4a.**

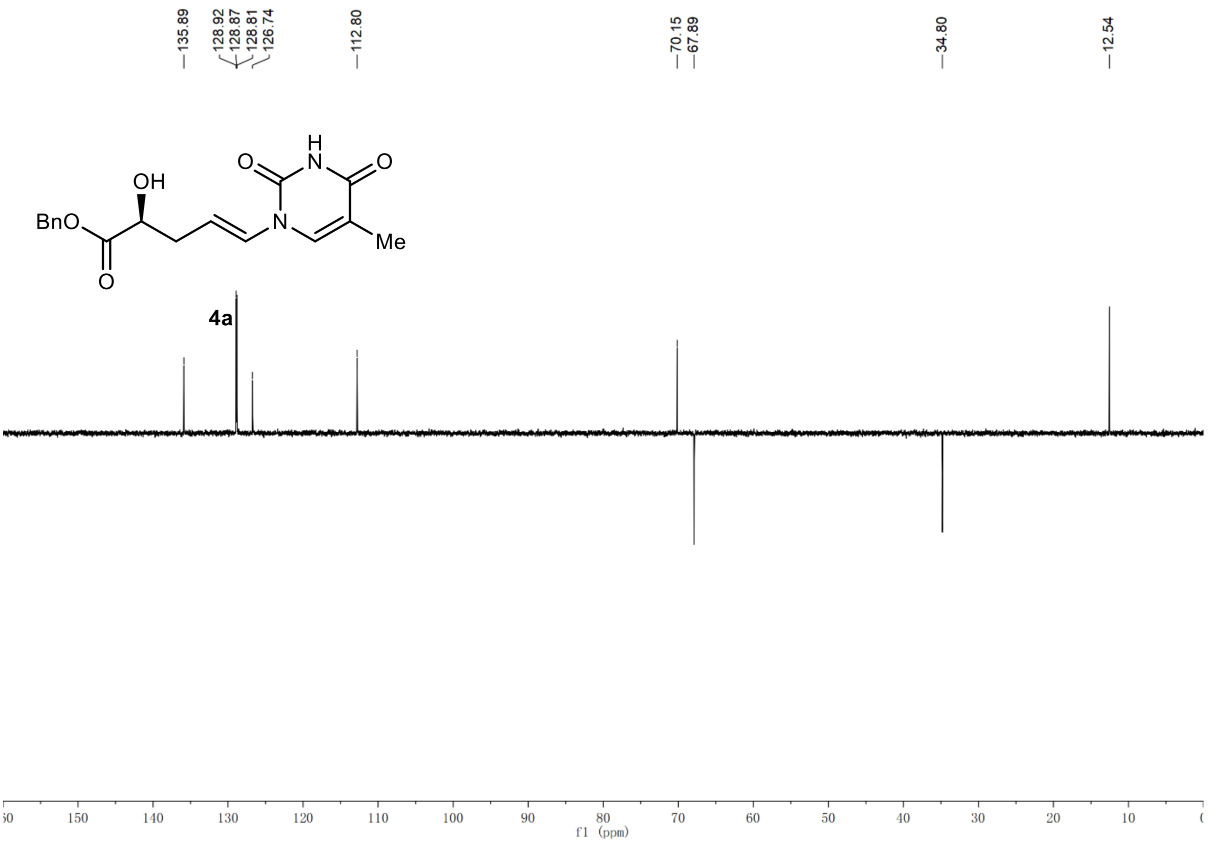

Supplementary Figure 53. DEPT 135° (100M, $CDCl_3$) of compound **4a.**

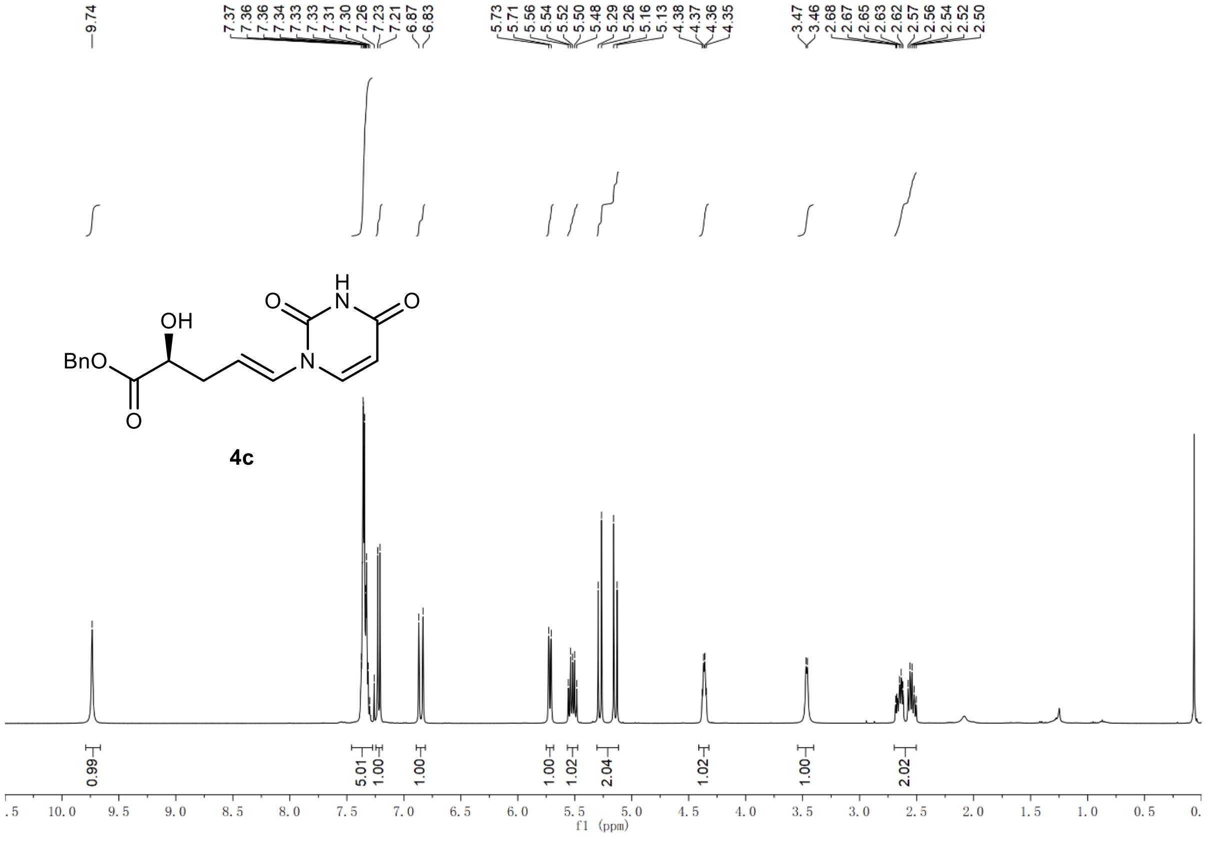

Supplementary Figure 54. ^{1}H NMR (400M, $CDCl_3$) of compound **4c.**

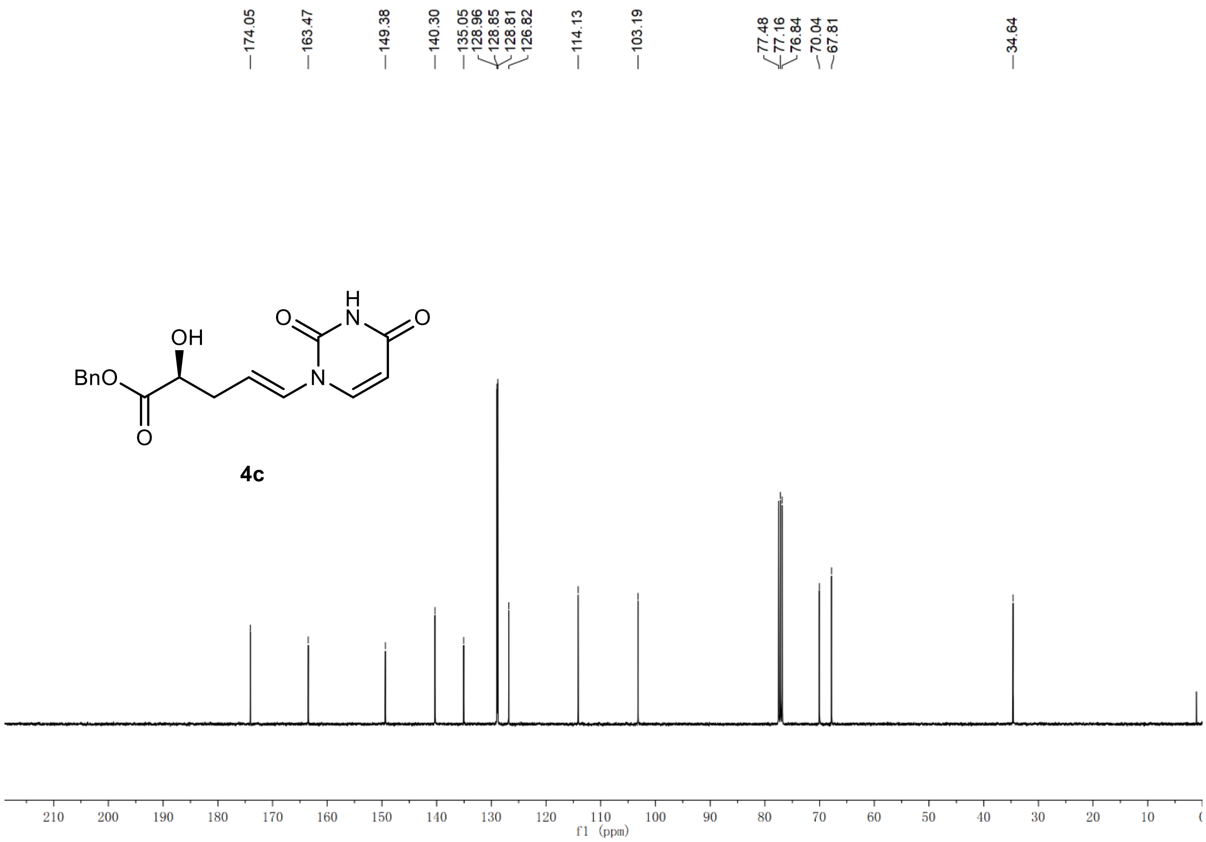

Supplementary Figure 55. ^{13}C NMR (100M, $CDCl_3$) of compound **4c.**

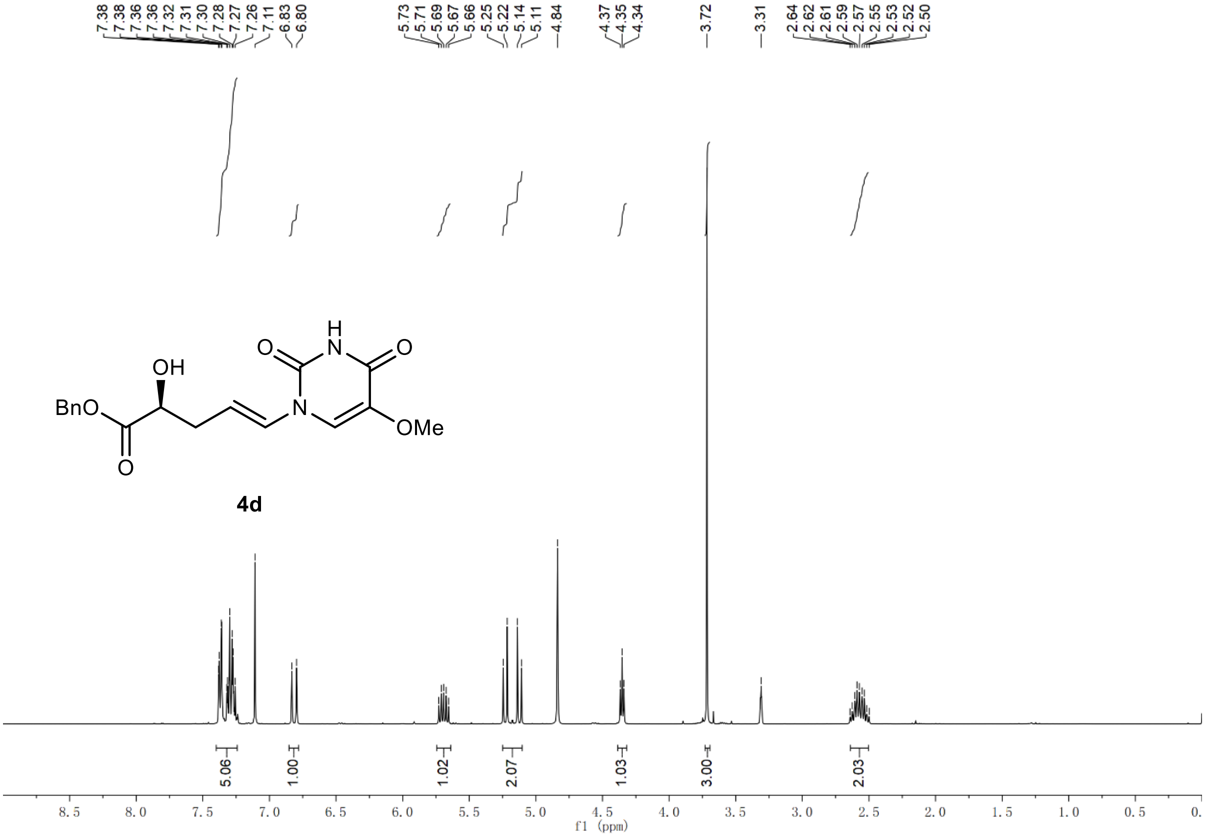

Supplementary Figure 56. ^{1}H NMR (400M, CD_3OD) of compound **4d.**

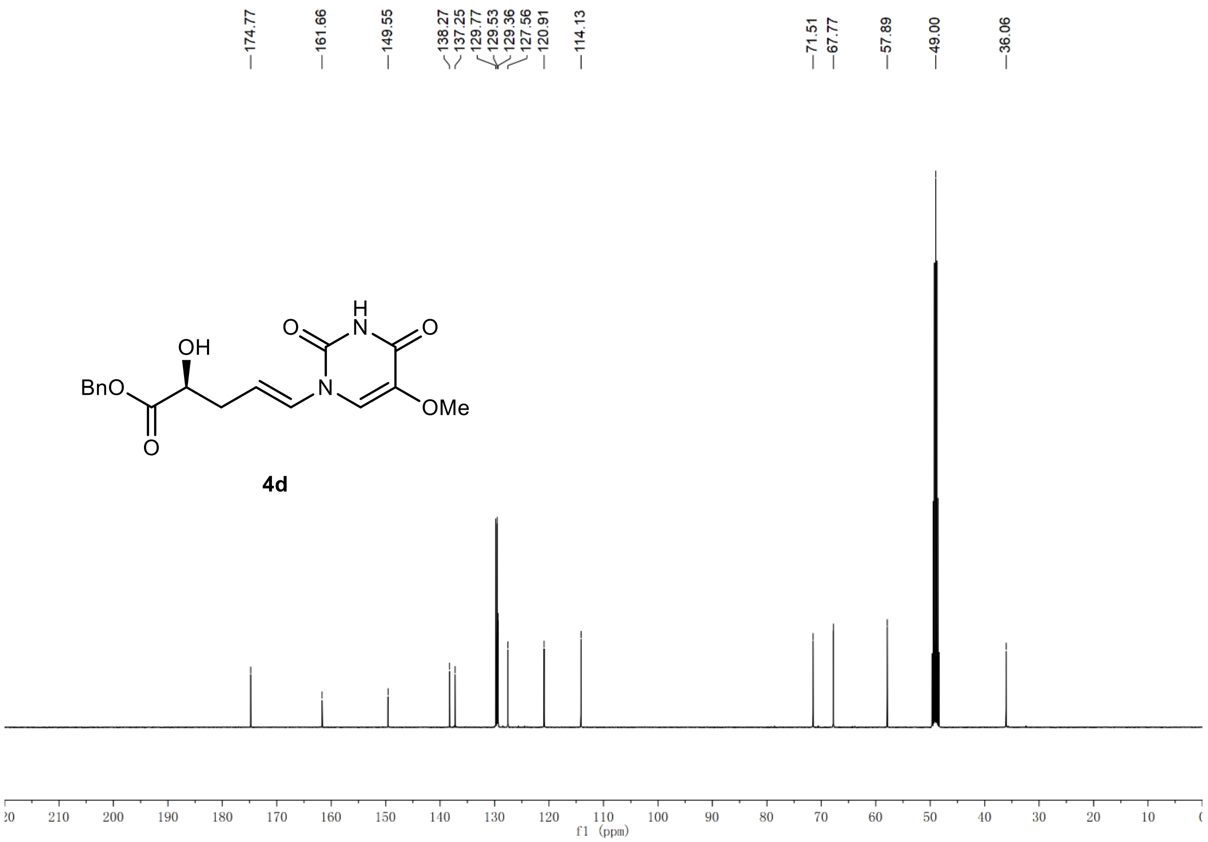

Supplementary Figure 57. ^{13}C NMR (100M, CD_3OD) of compound **4d.**

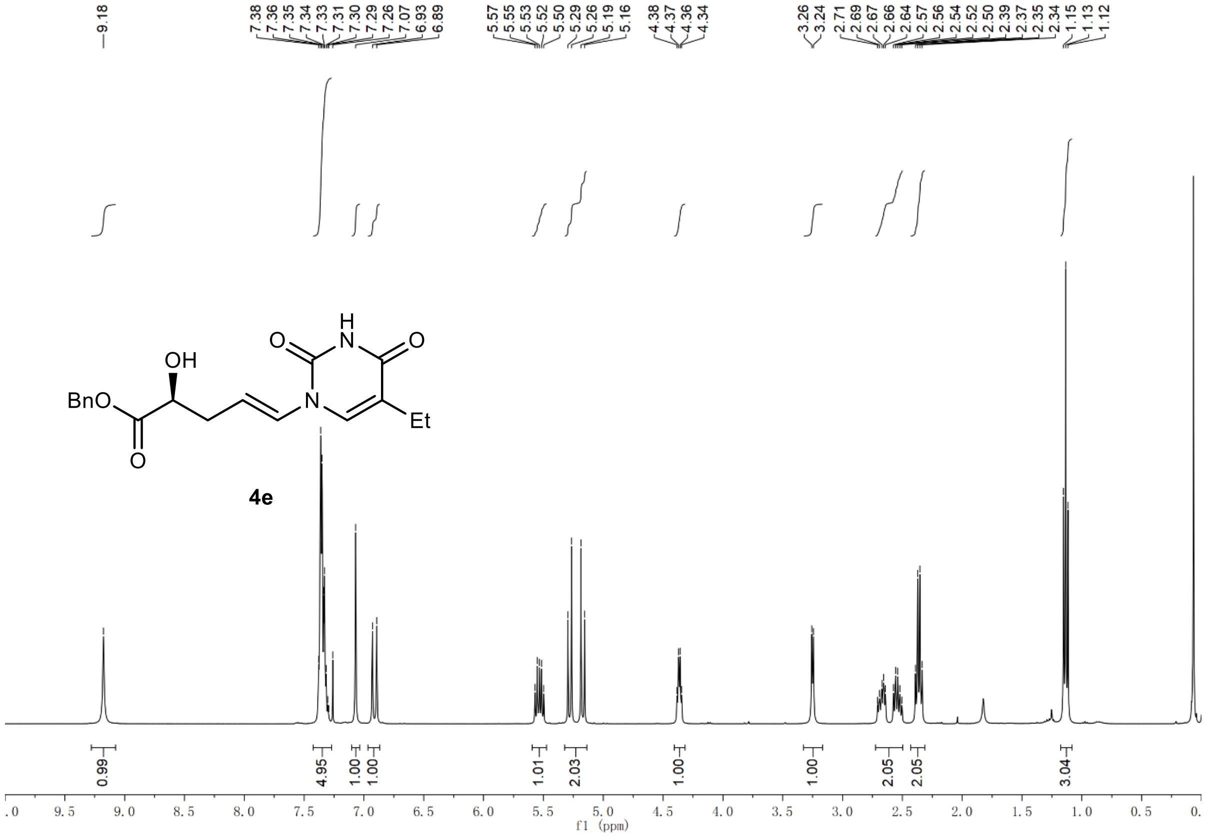

Supplementary Figure 58. ^{1}H NMR (400M, $CDCl_3$) of compound **4e.**

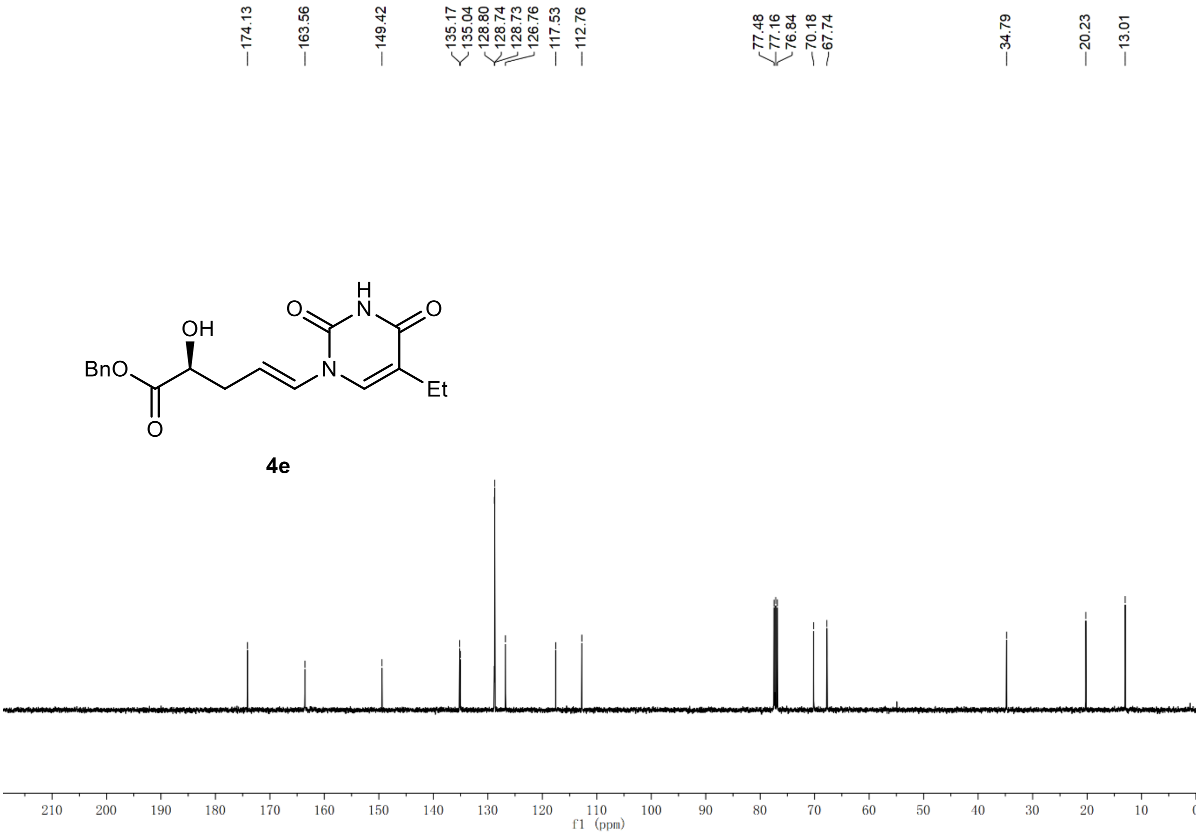

Supplementary Figure 59. ^{13}C NMR (100M, $CDCl_3$) of compound **4e.**

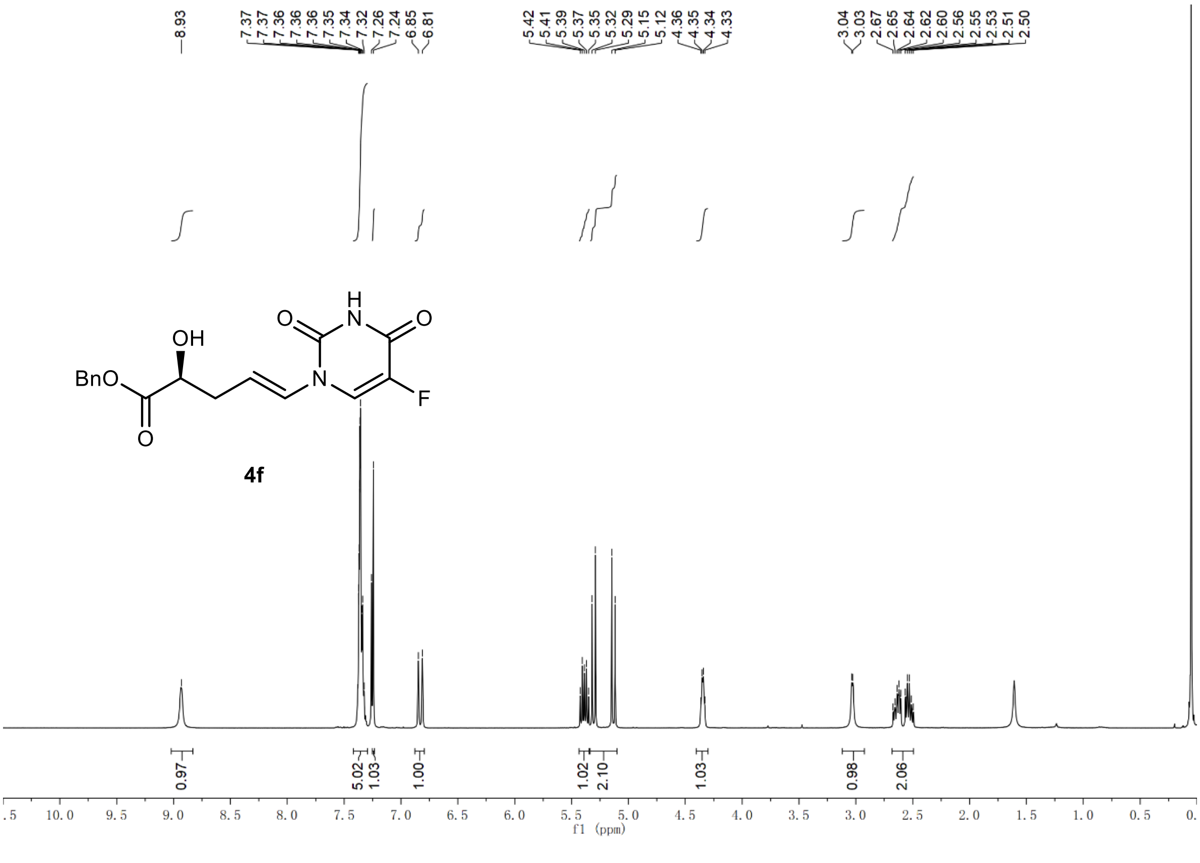

Supplementary Figure 60. ^{1}H NMR (400M, $CDCl_3$) of compound **4f.**

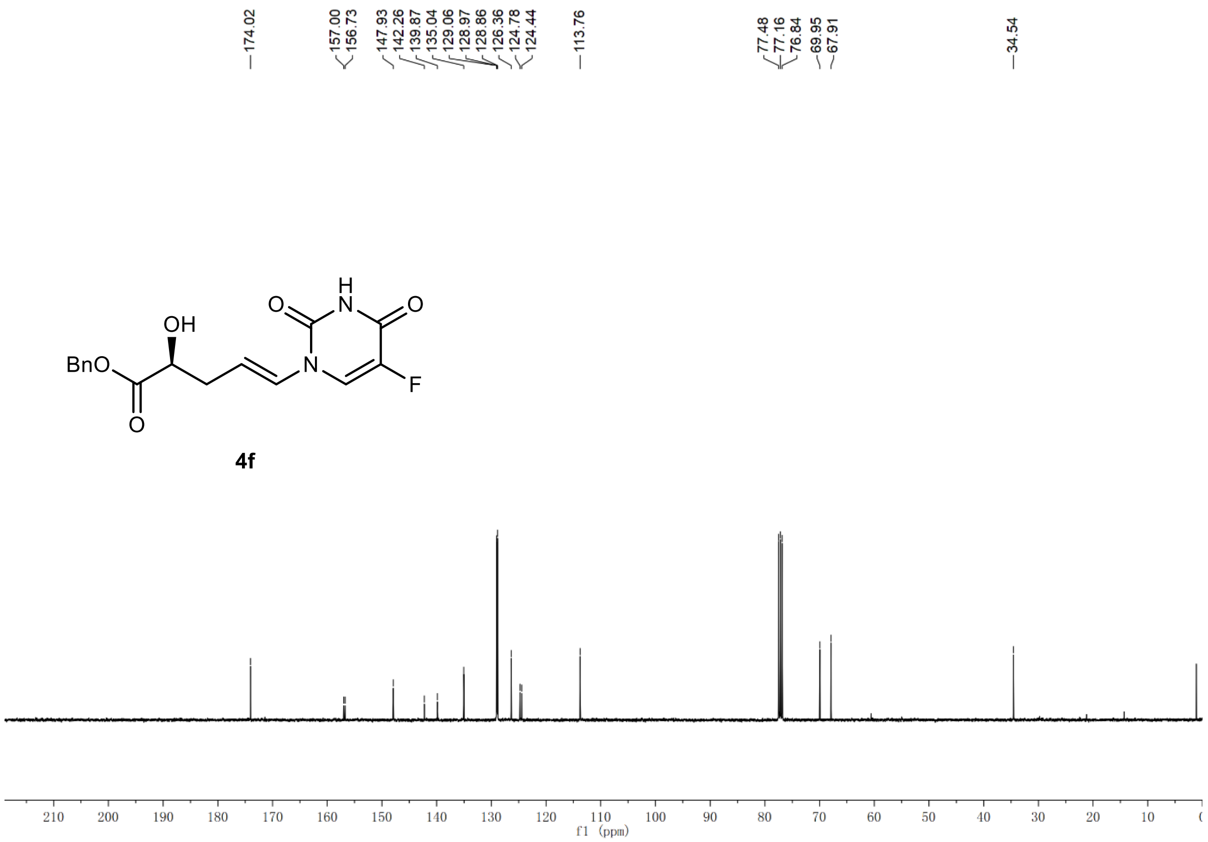

Supplementary Figure 61. ^{13}C NMR (100M, $CDCl_3$) of compound **4f.**

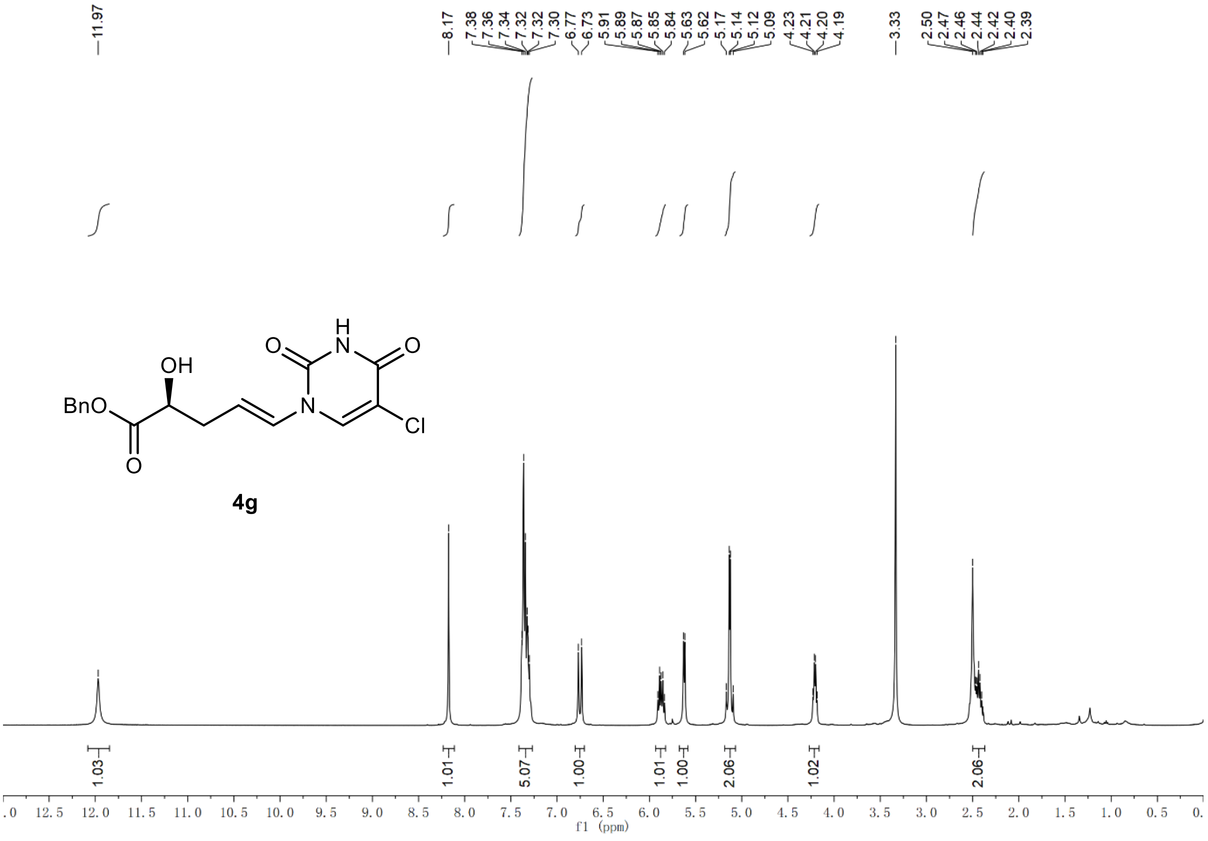

Supplementary Figure 62. ^{1}H NMR (400M, $(CD_3)_2SO$) of compound **4g.**

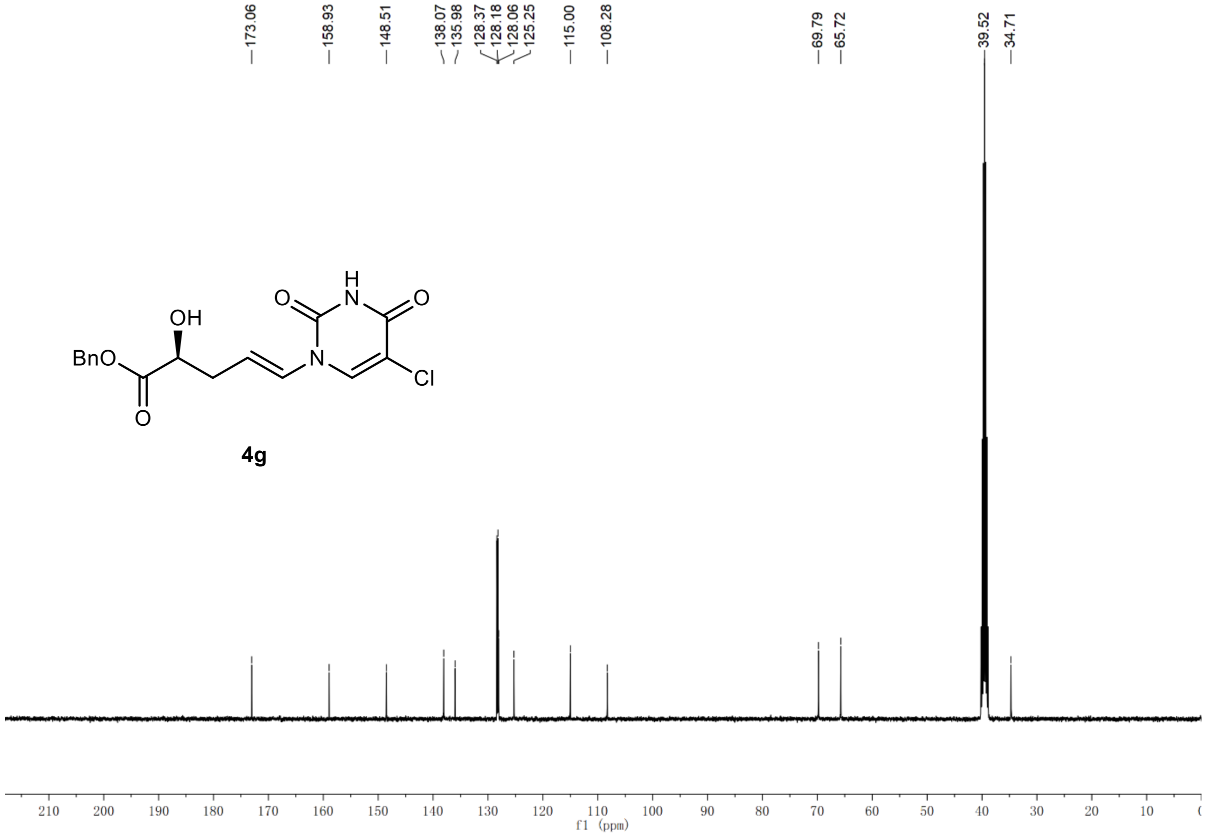

Supplementary Figure 63. ^{13}C NMR (100M, $(CD_3)_2SO$) of compound **4g.**

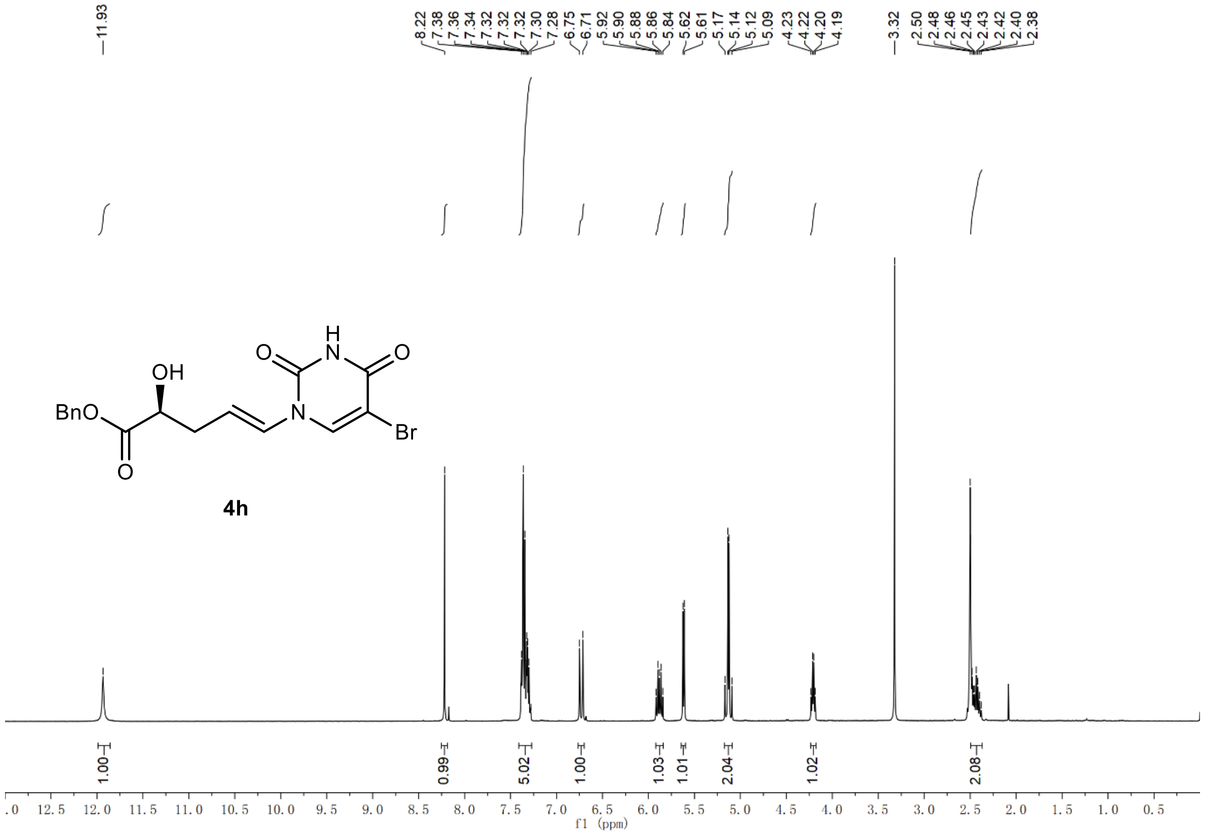

Supplementary Figure 64. ^{1}H NMR (400M, $(CD_3)_2SO$) of compound **4h.**

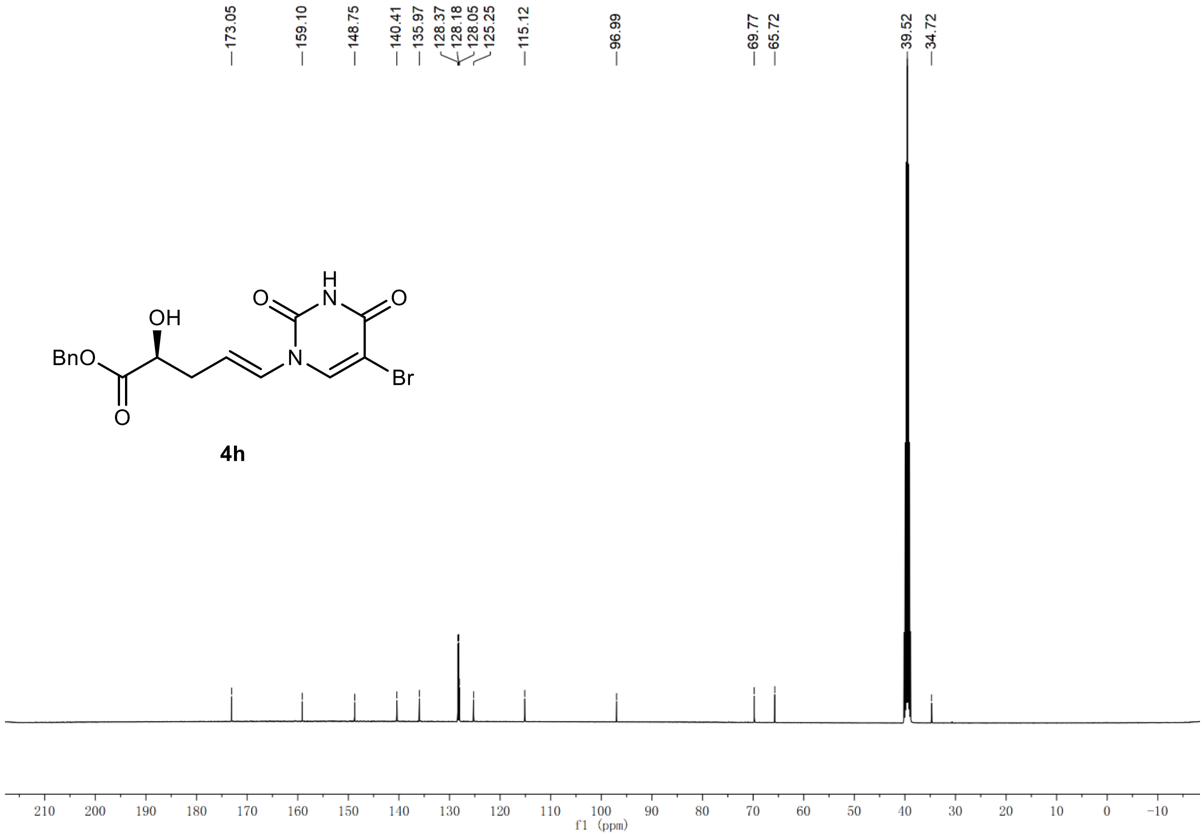

Supplementary Figure 65. ^{13}C NMR (100M, $(CD_3)_2SO$) of compound **4h.**

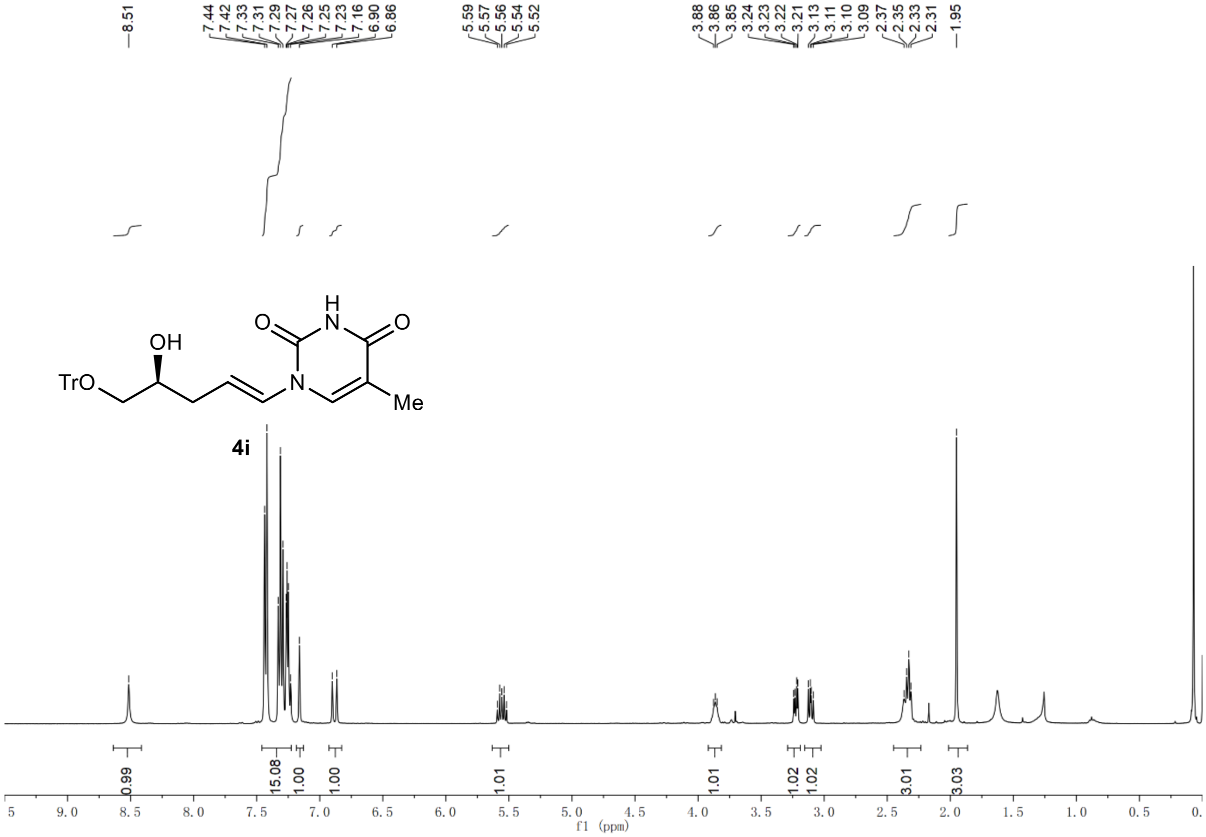

Supplementary Figure 66. ^{1}H NMR (400M, $CDCl_3$) of compound **4i.**

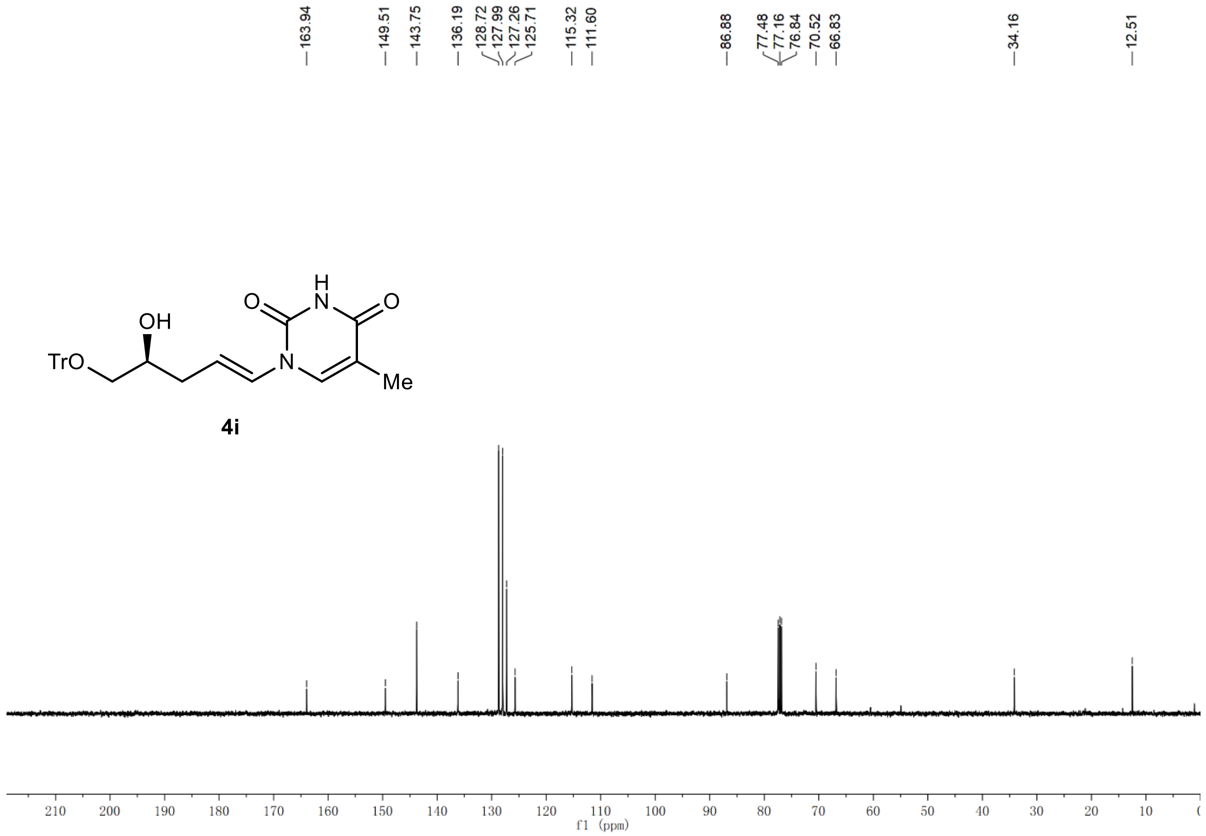

Supplementary Figure 67. ^{13}C NMR (100M, $CDCl_3$) of compound **4i.**

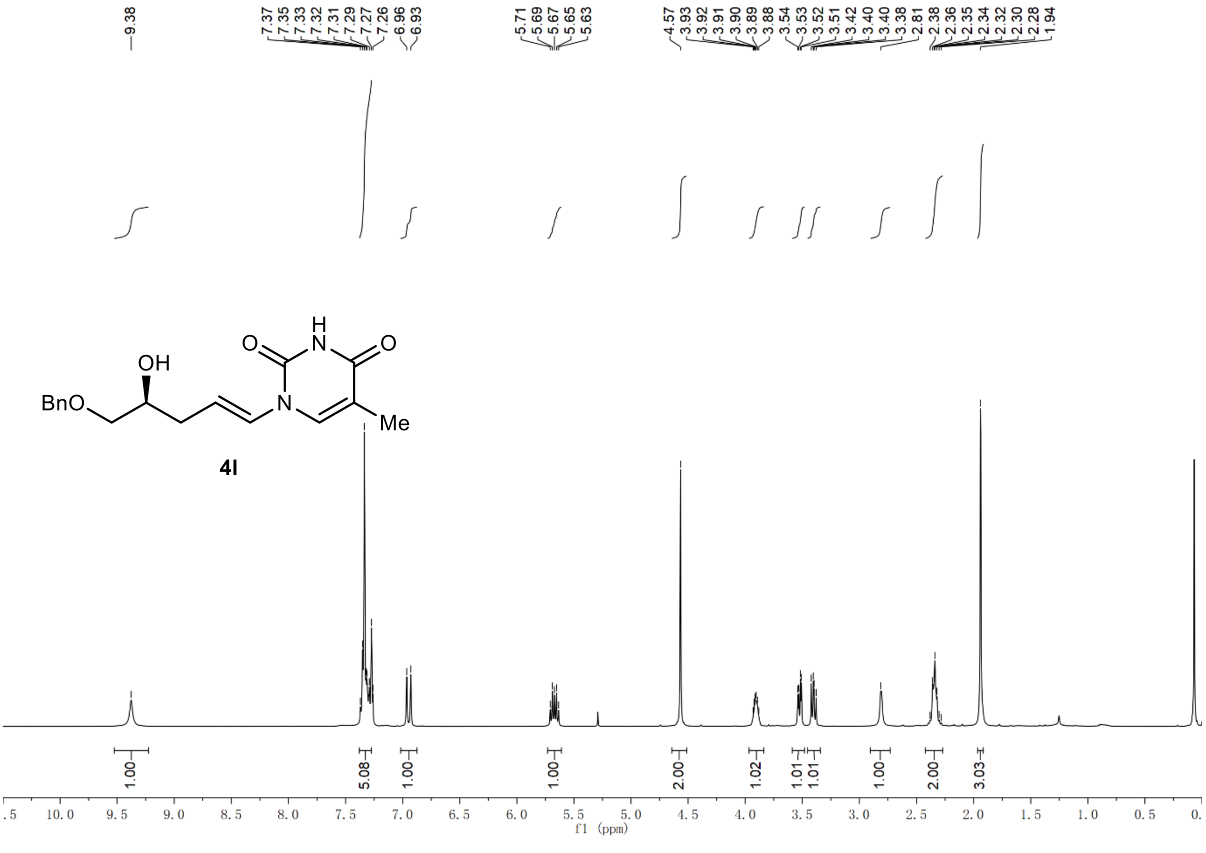

Supplementary Figure 68. ^{1}H NMR (400M, $CDCl_3$) of compound **4l.**

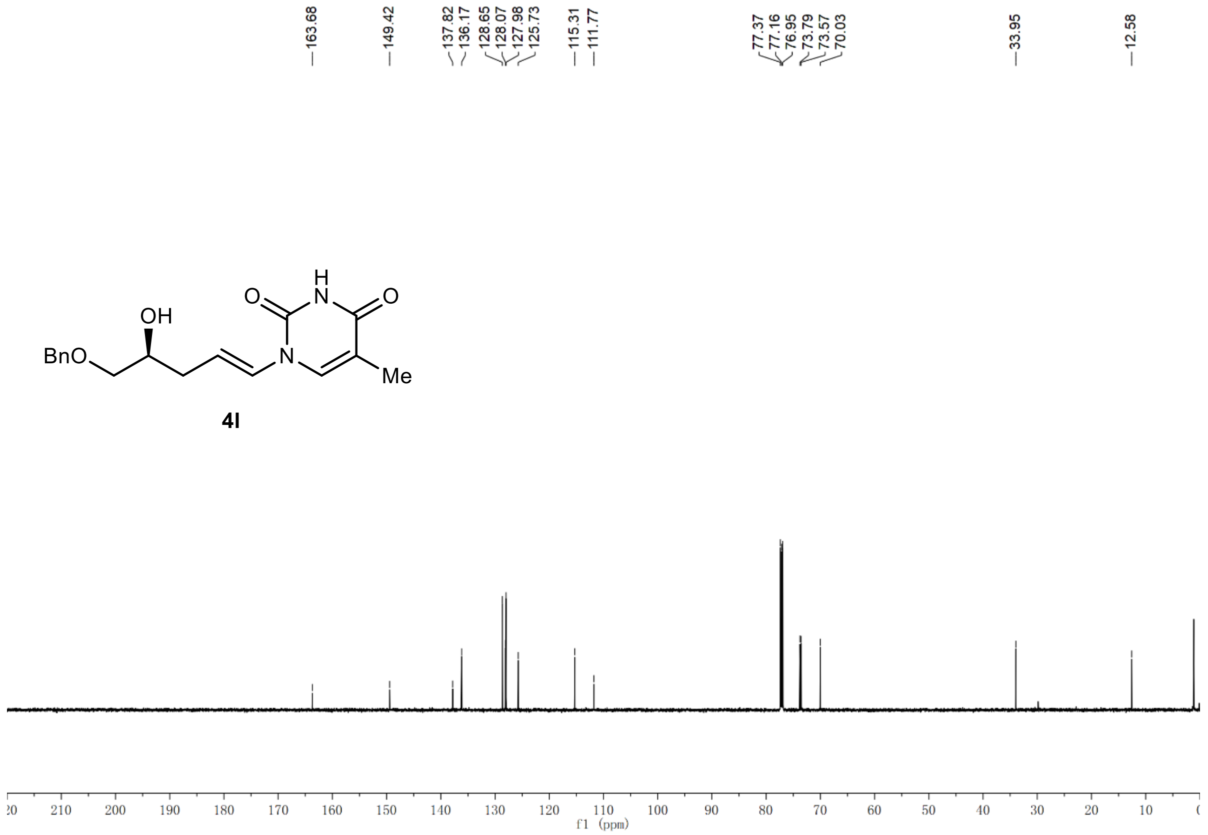

Supplementary Figure 69. ^{13}C NMR (100M, $CDCl_3$) of compound **4l.**

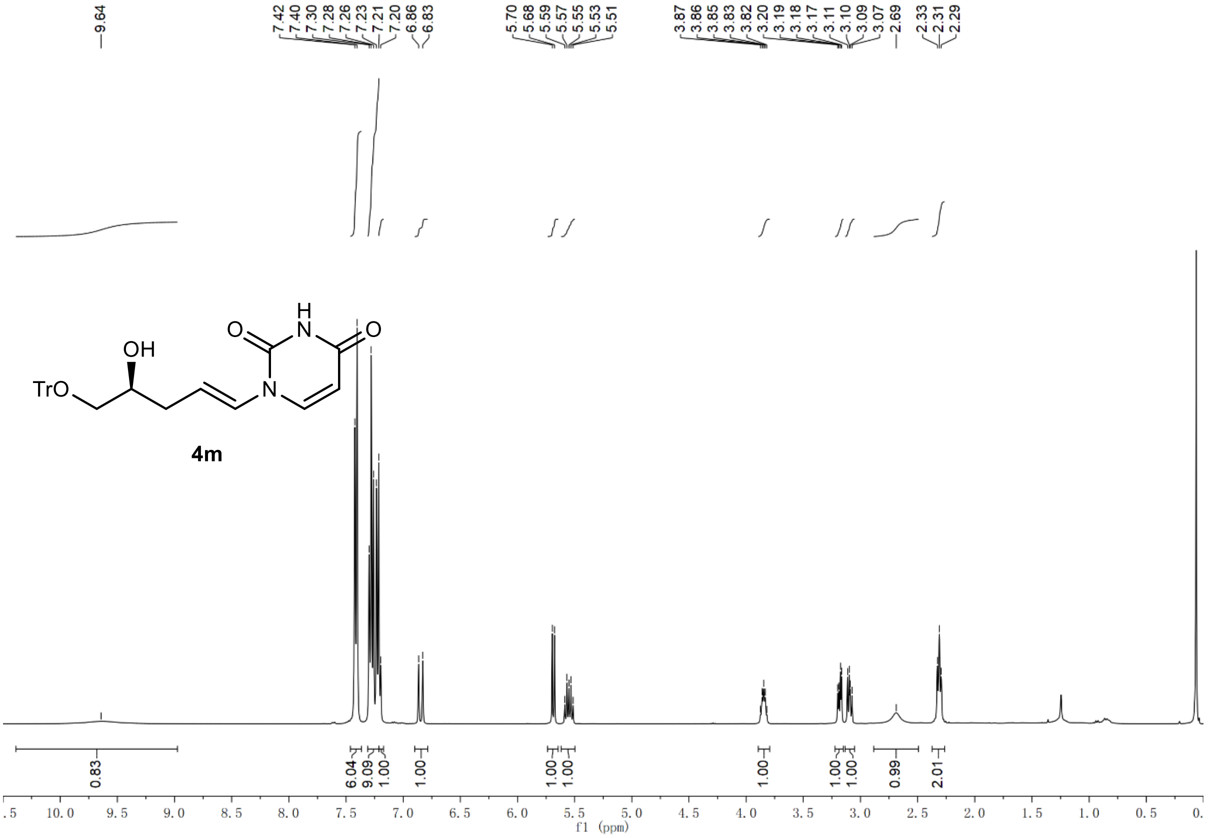

Supplementary Figure 70. ^{1}H NMR (400M, $CDCl_3$) of compound **4m.**

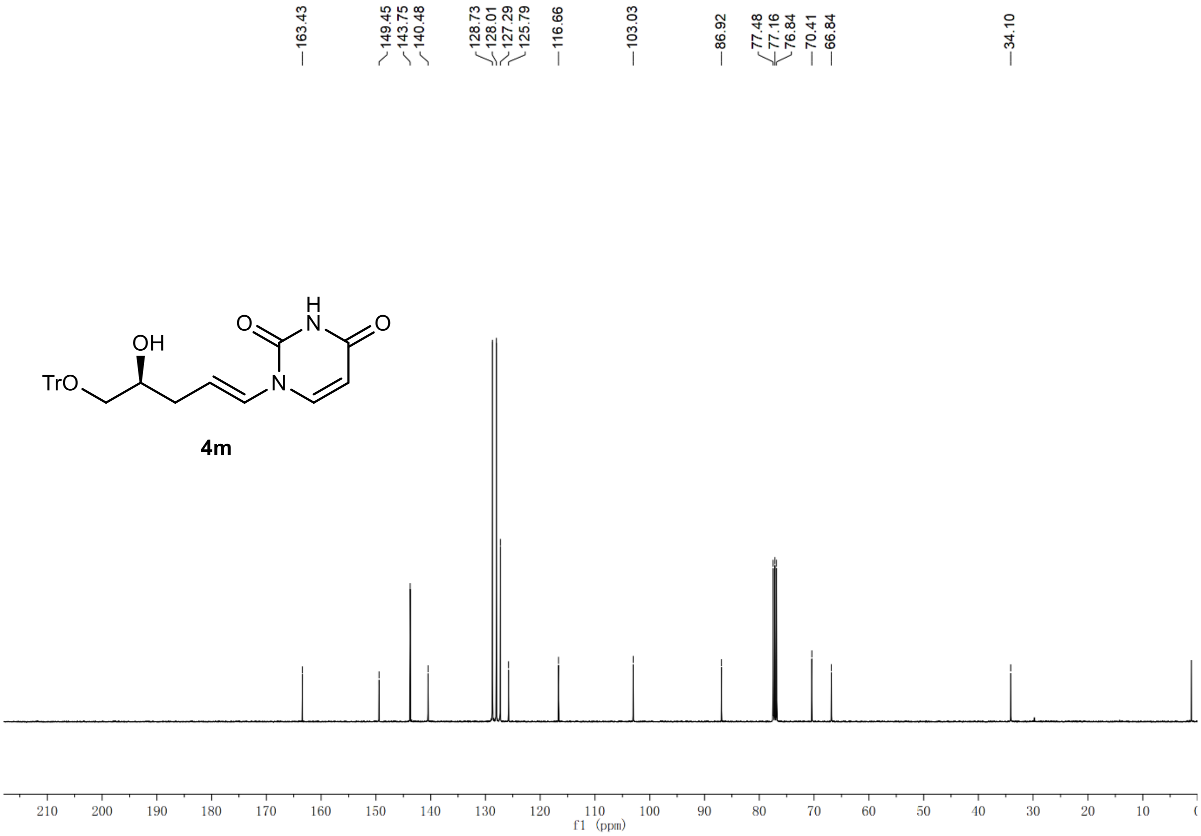

Supplementary Figure 71. ^{13}C NMR (100M, $CDCl_3$) of compound **4m.**

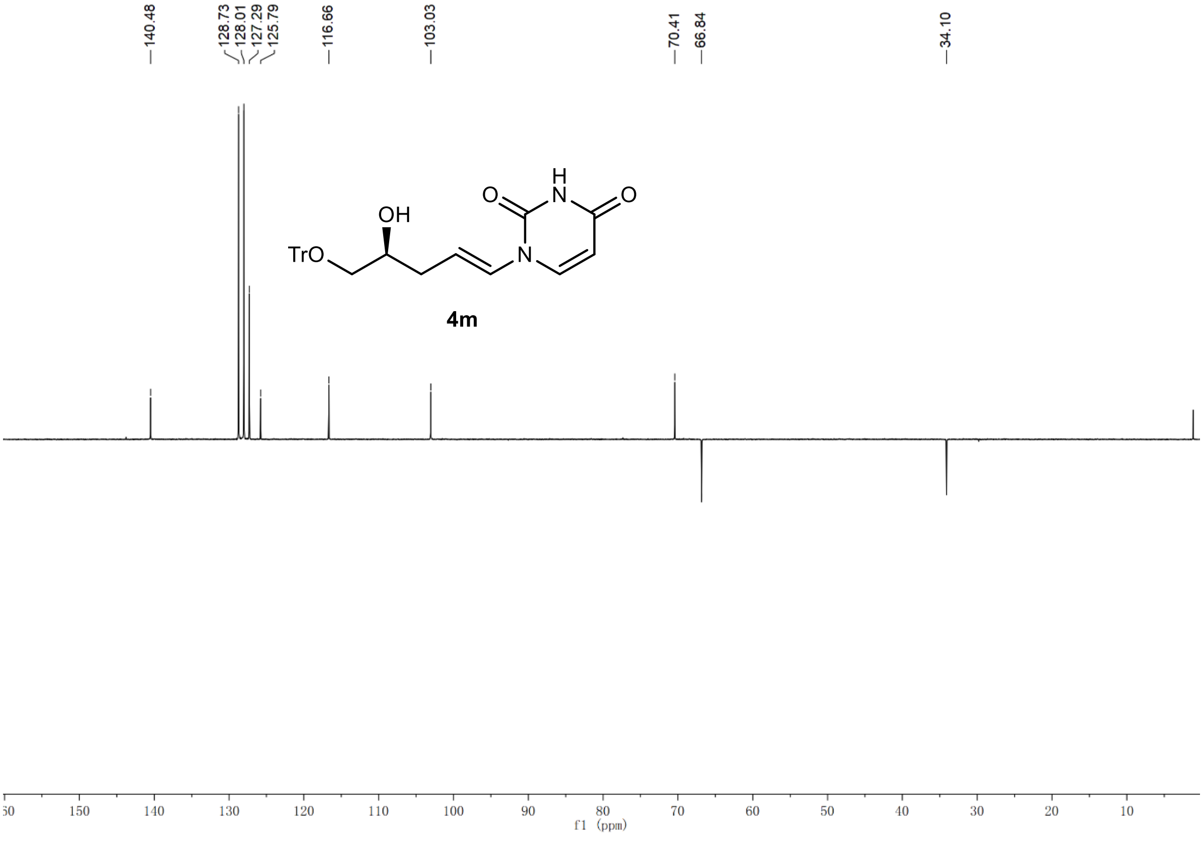

Supplementary Figure 72. DEPT 135° (100M, $CDCl_3$) of compound **4m.**

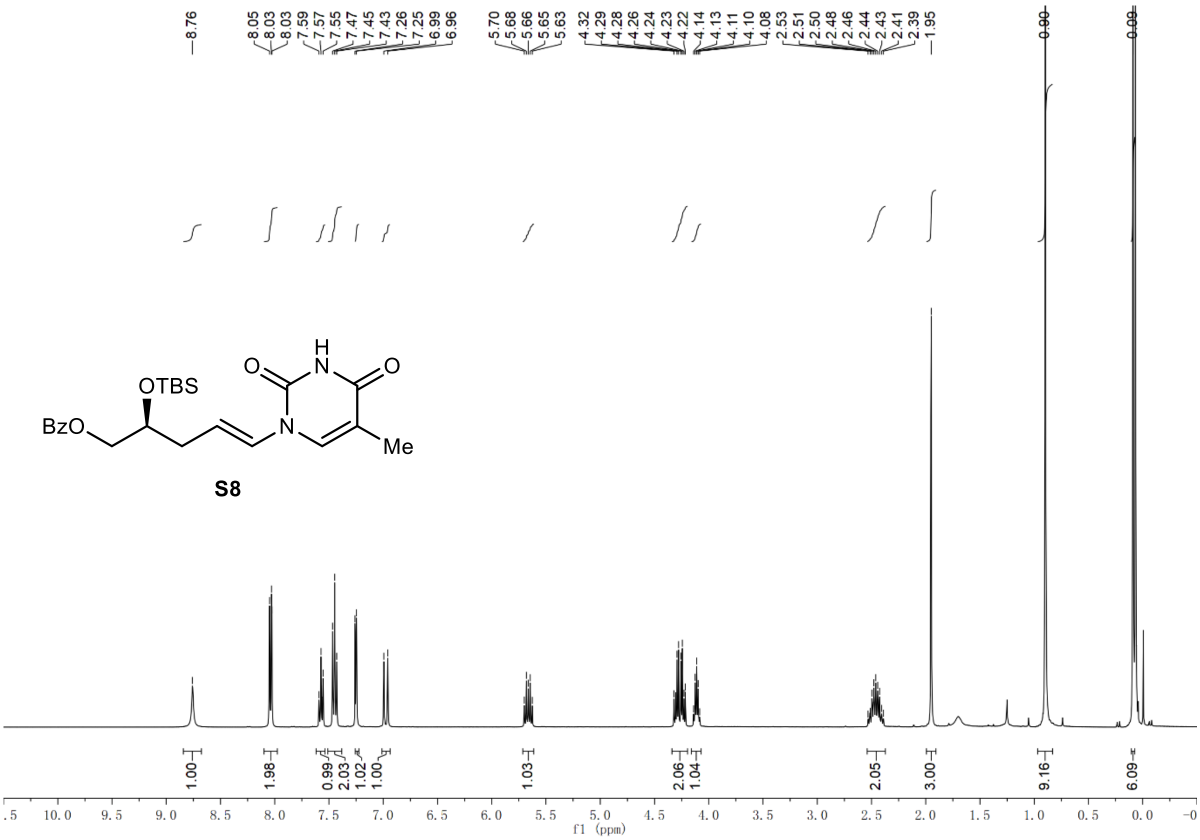

Supplementary Figure 73. ^{1}H NMR (400M, $CDCl_3$) of compound **S8.**

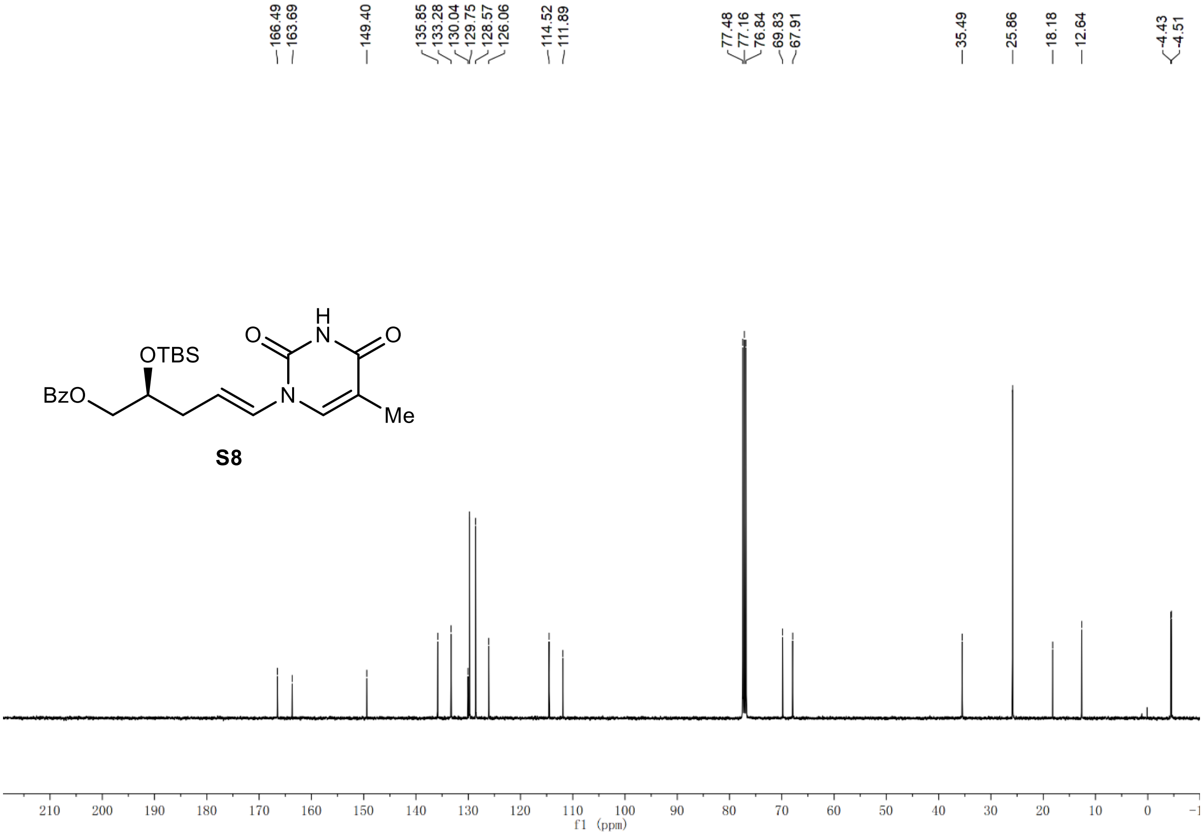

Supplementary Figure 74. ^{13}C NMR (100M, $CDCl_3$) of compound **S8.**

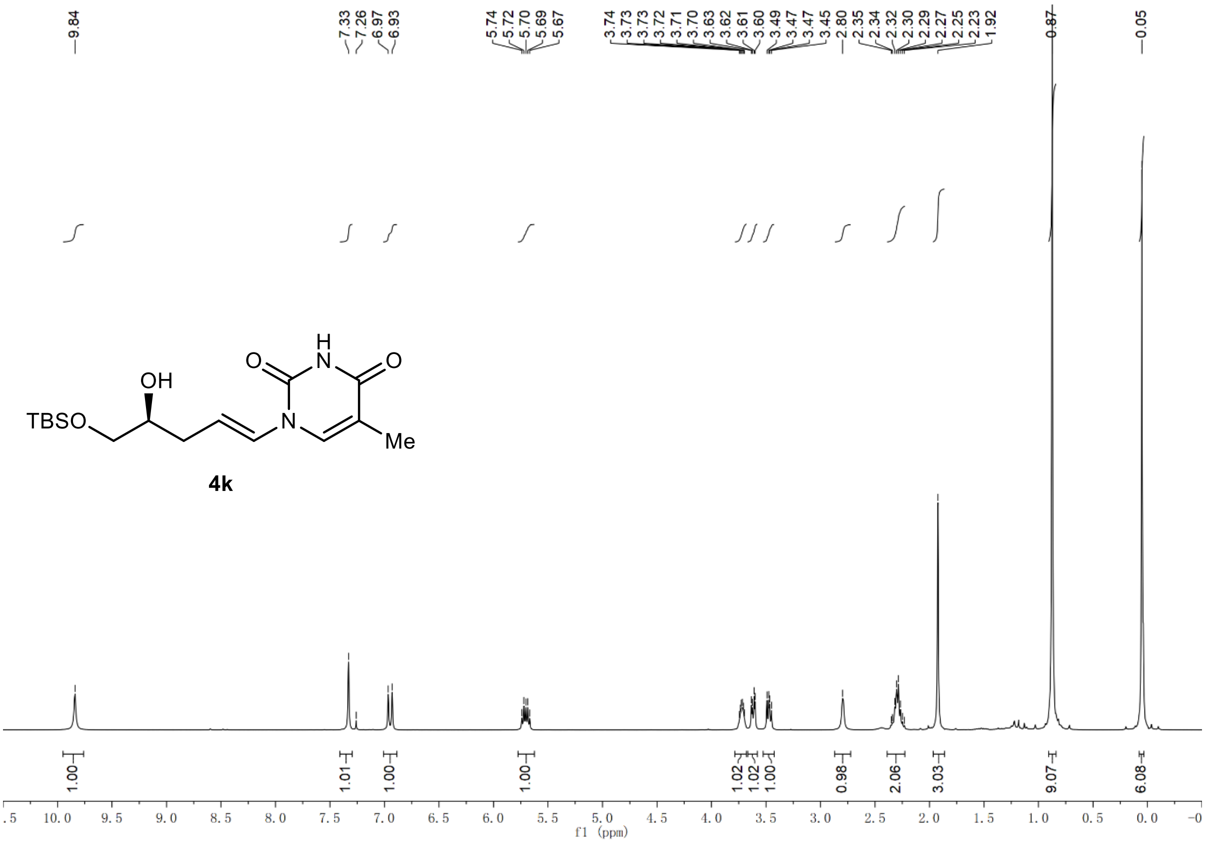

Supplementary Figure 75. ^{1}H NMR (400M, $CDCl_3$) of compound **4k.**

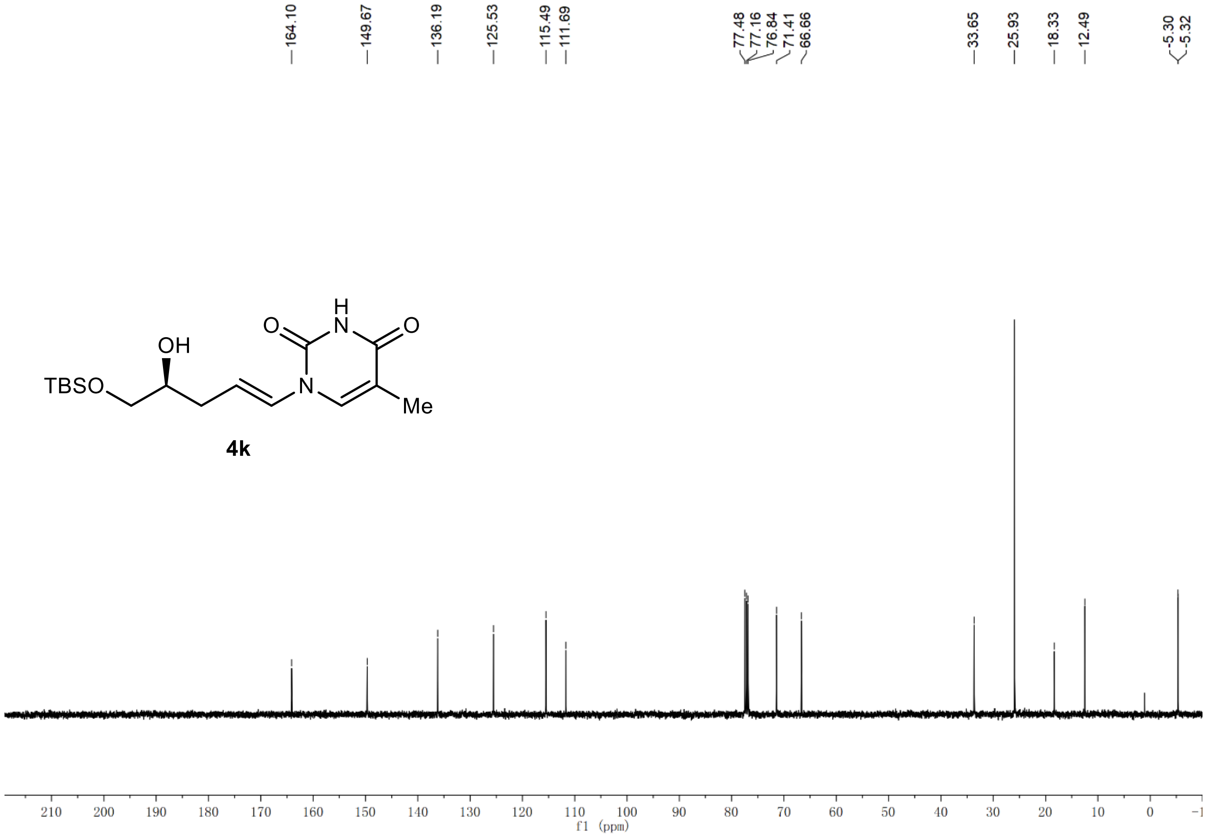

Supplementary Figure 76. ^{13}C NMR (100M, $CDCl_3$) of compound **4k.**

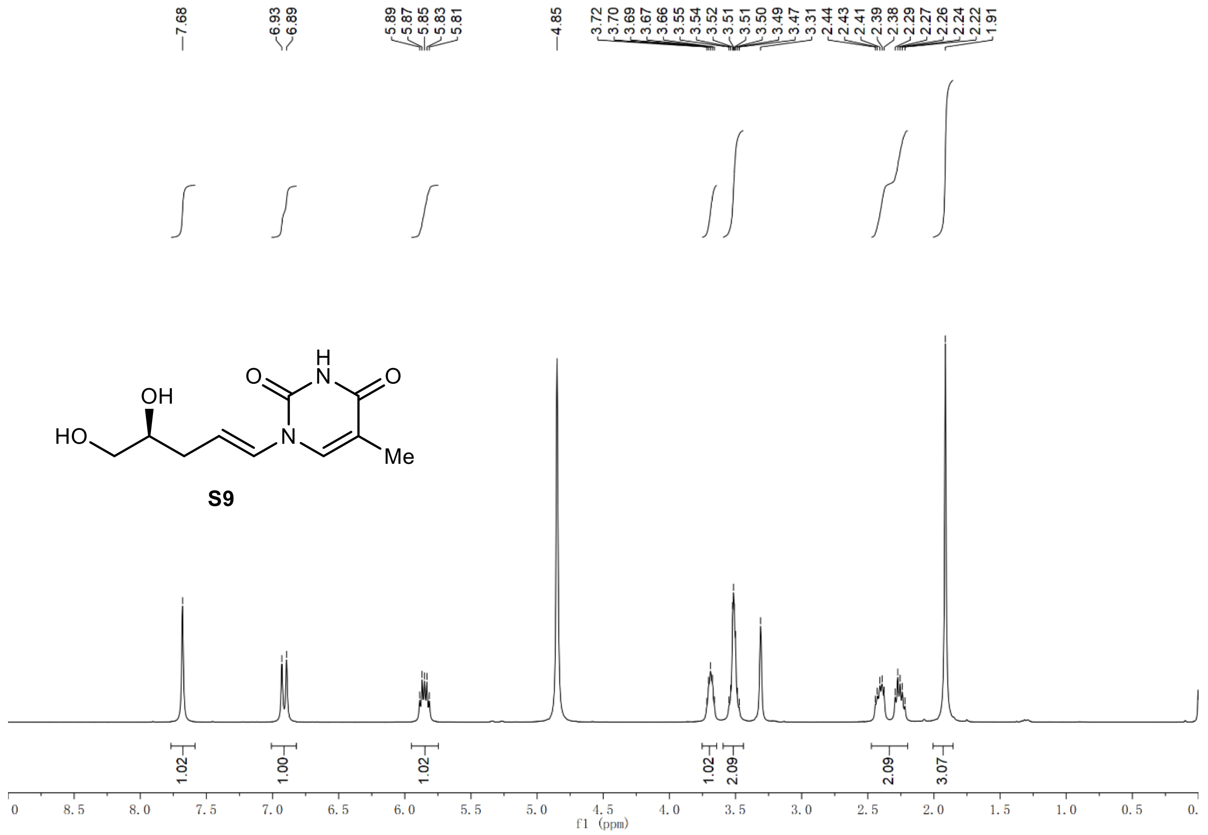

Supplementary Figure 77. ^{1}H NMR (400M, CD_3OD) of compound **S9.**

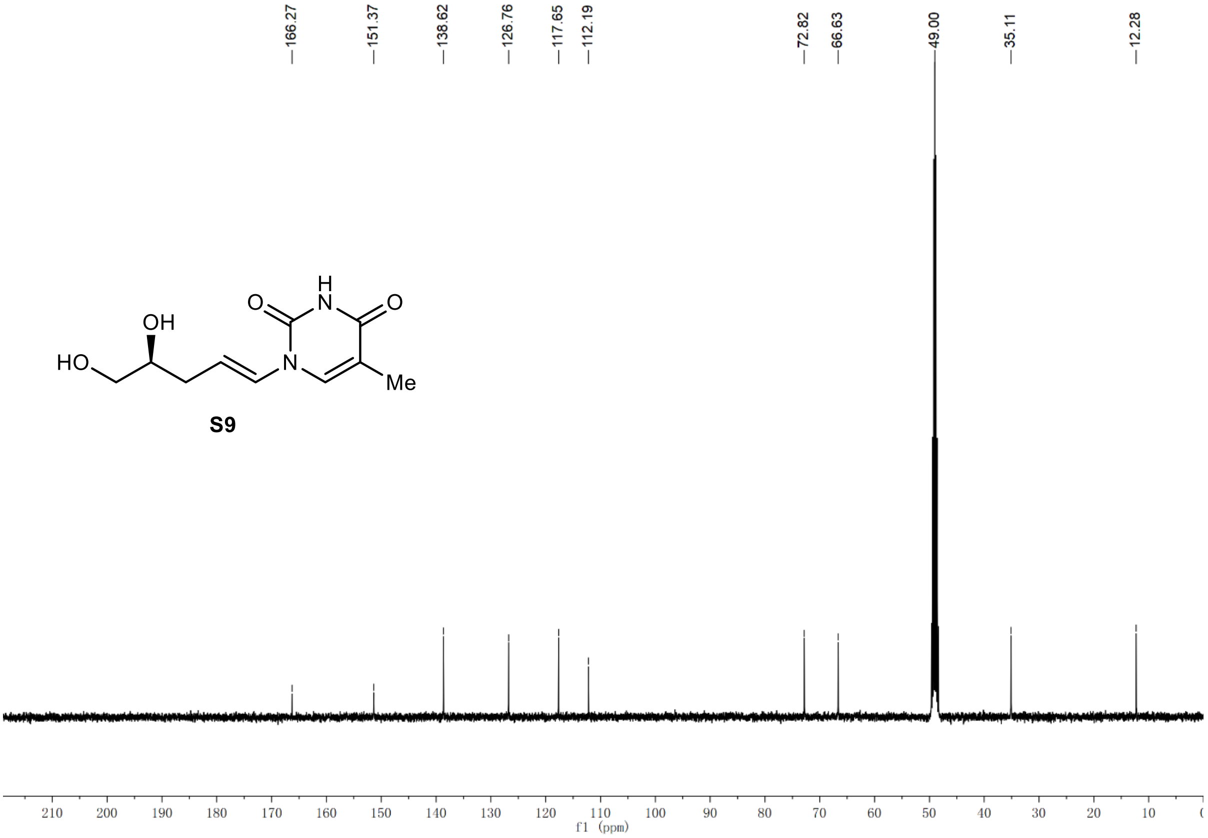

Supplementary Figure 78. ^{13}C NMR (100M, CD_3OD) of compound **S9.**

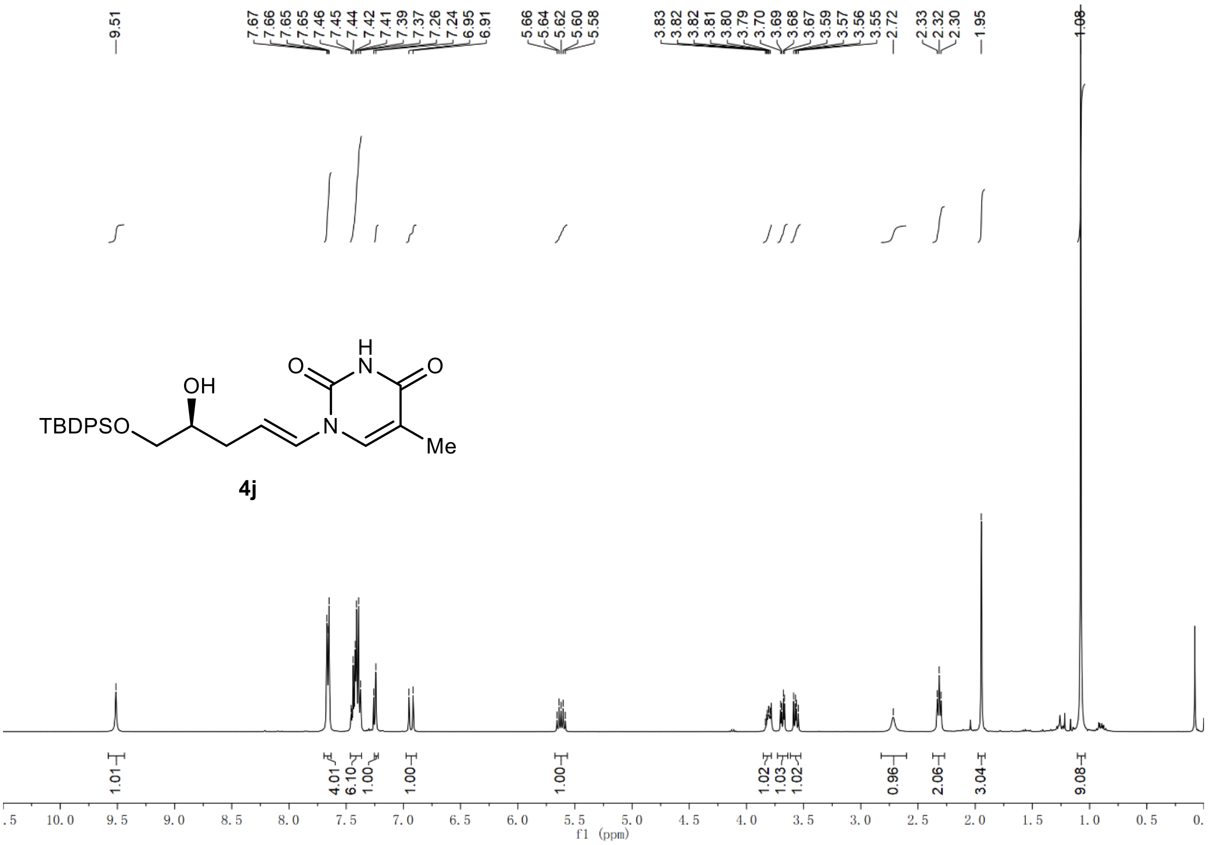

Supplementary Figure 79. ^{1}H NMR (400M, $CDCl_3$) of compound **4j.**

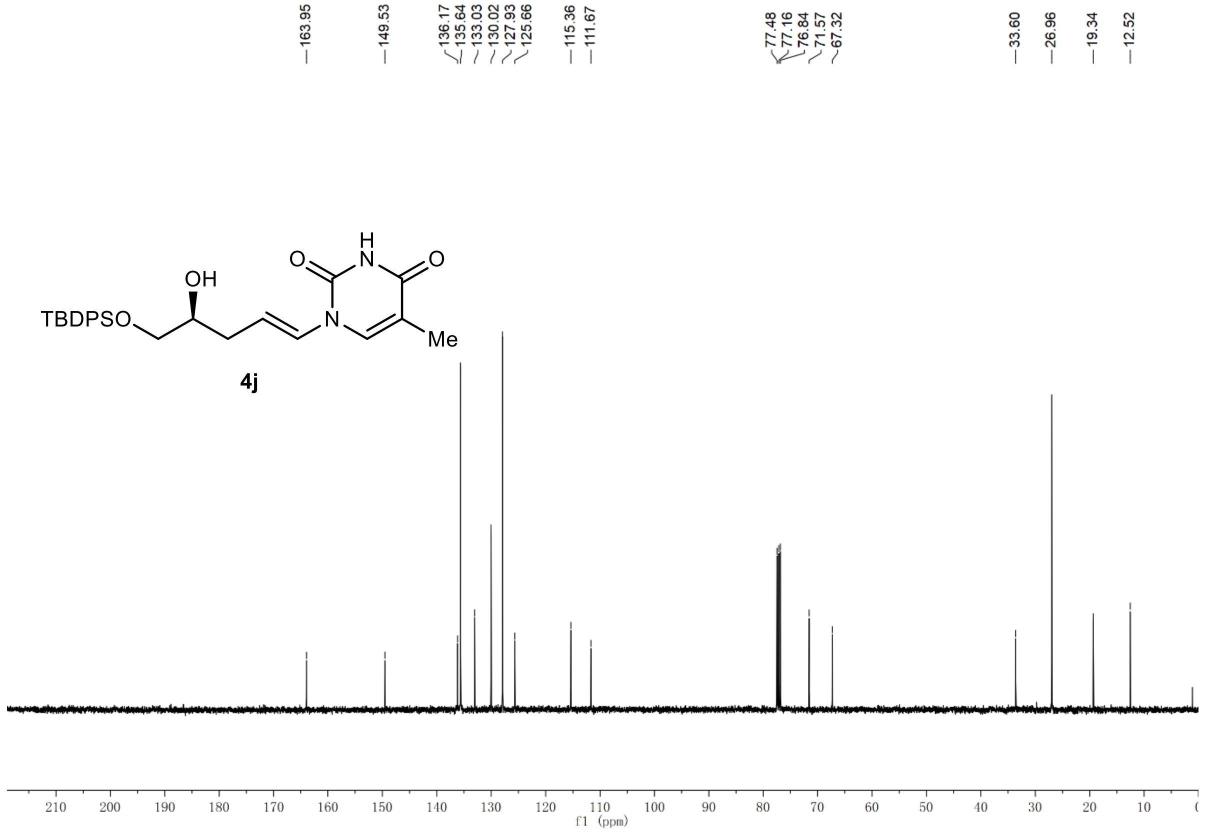

Supplementary Figure 80. ^{13}C NMR (100M, $CDCl_3$) of compound **4j.**

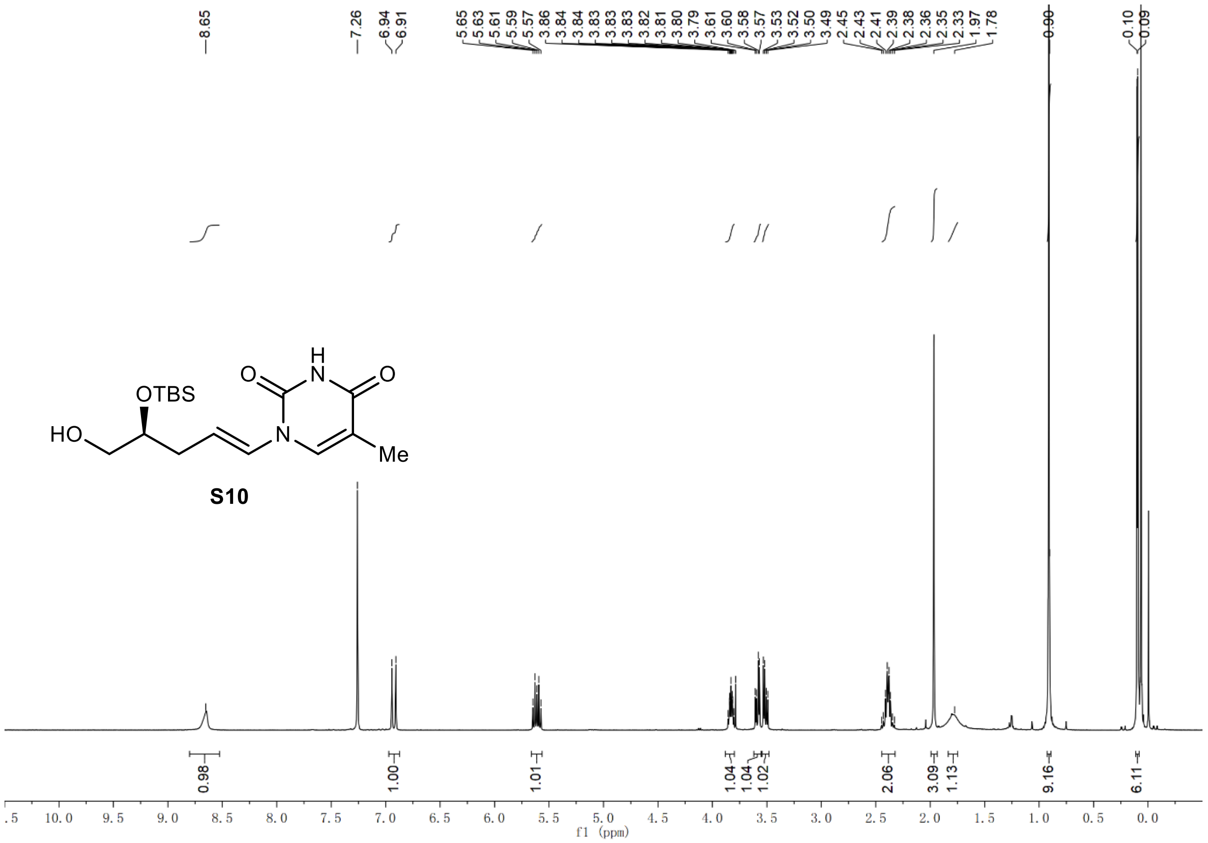

Supplementary Figure 81. ^{1}H NMR (400M, $CDCl_3$) of compound **S10.**

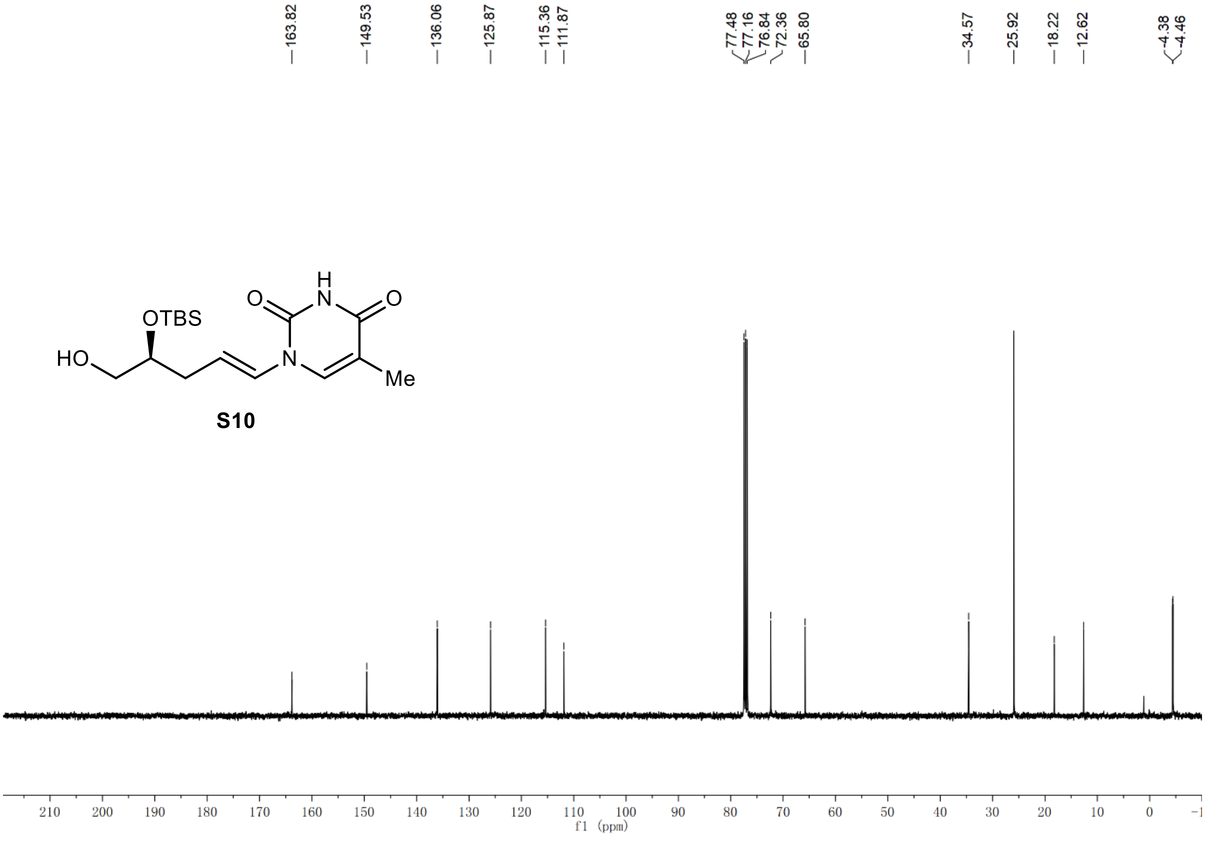

Supplementary Figure 82. ^{13}C NMR (100M, $CDCl_3$) of compound **S10.**

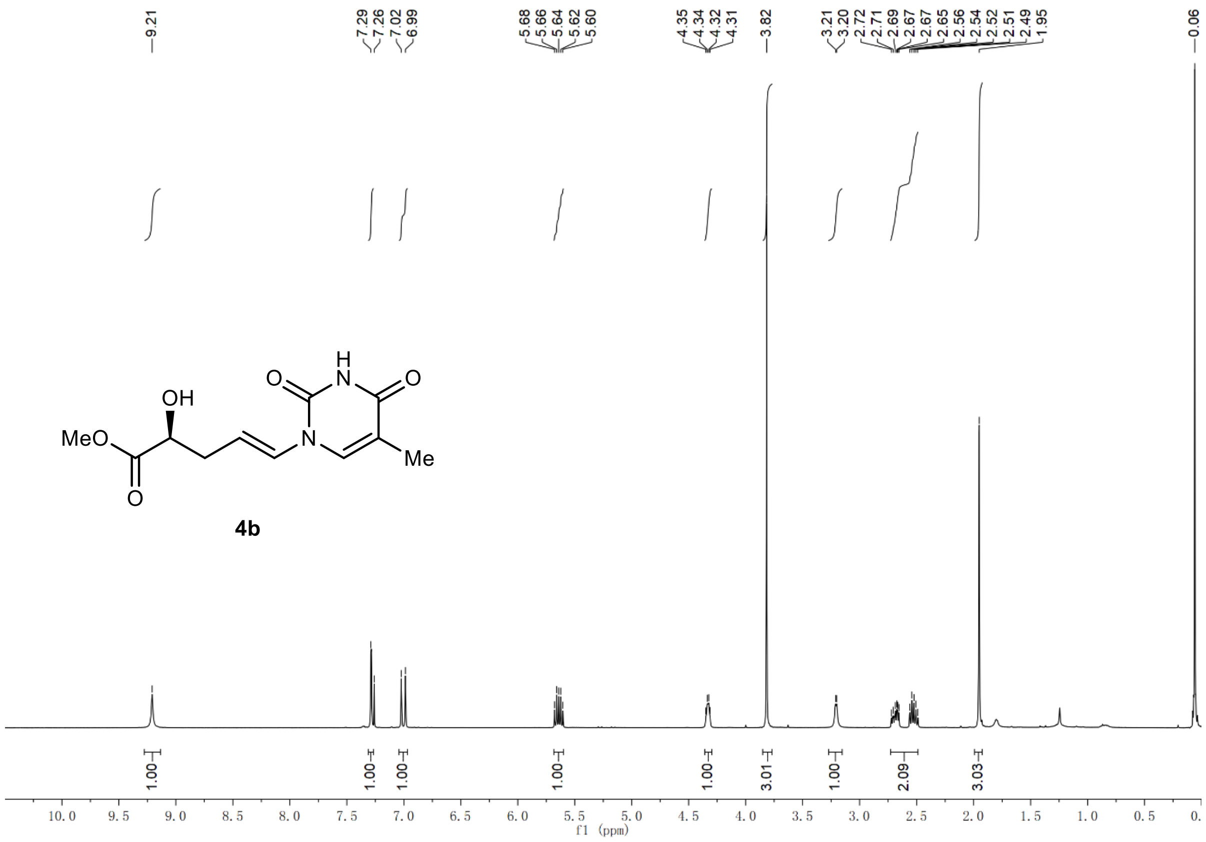

Supplementary Figure 83. ^{1}H NMR (400M, $CDCl_3$) of compound **4b.**

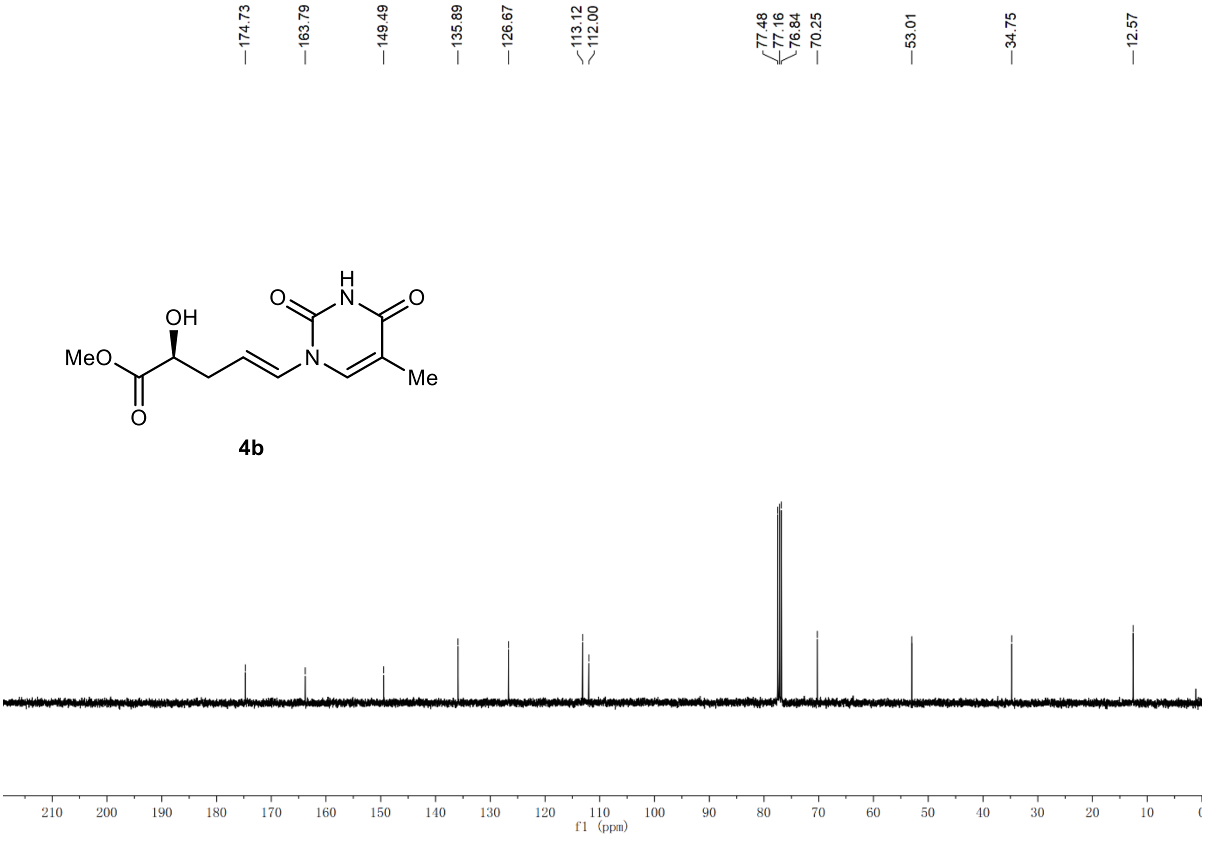

Supplementary Figure 84. ^{13}C NMR (100M, $CDCl_3$) of compound **4b.**

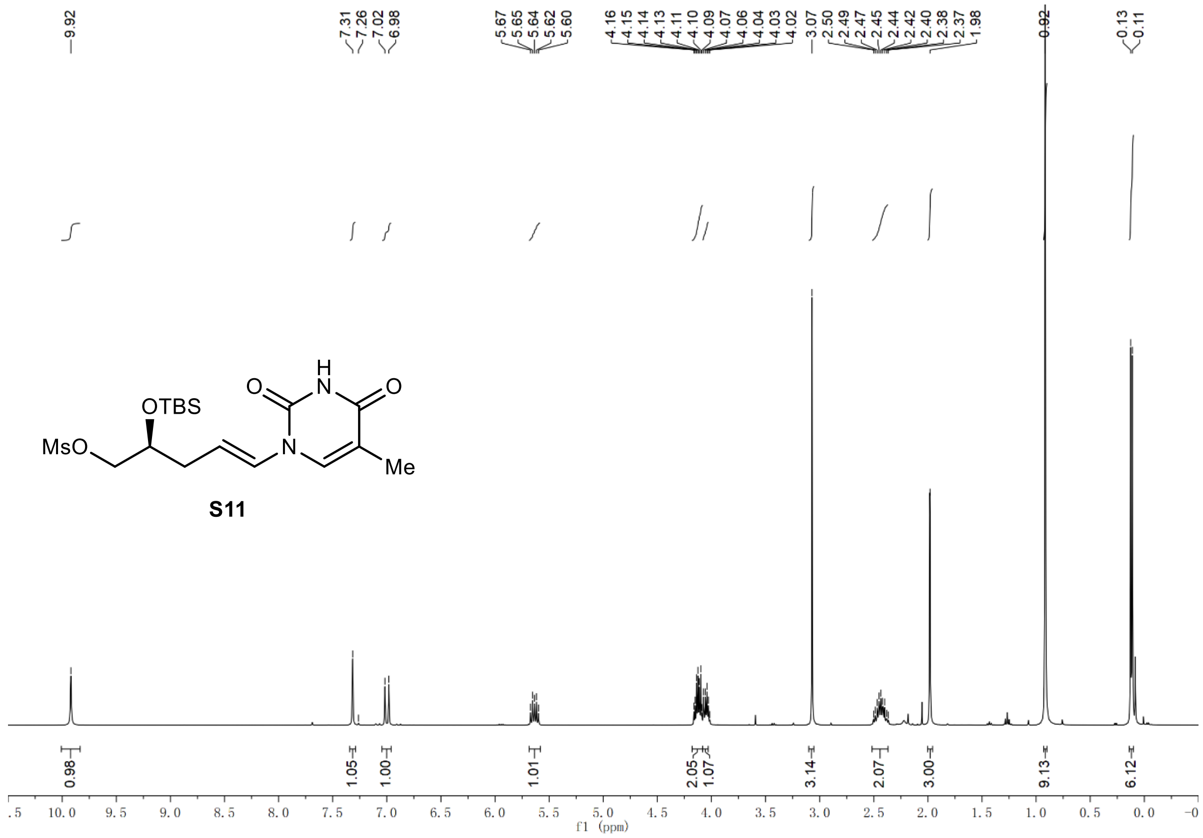

Supplementary Figure 85. ^{1}H NMR (400M, $CDCl_3$) of compound **S11.**

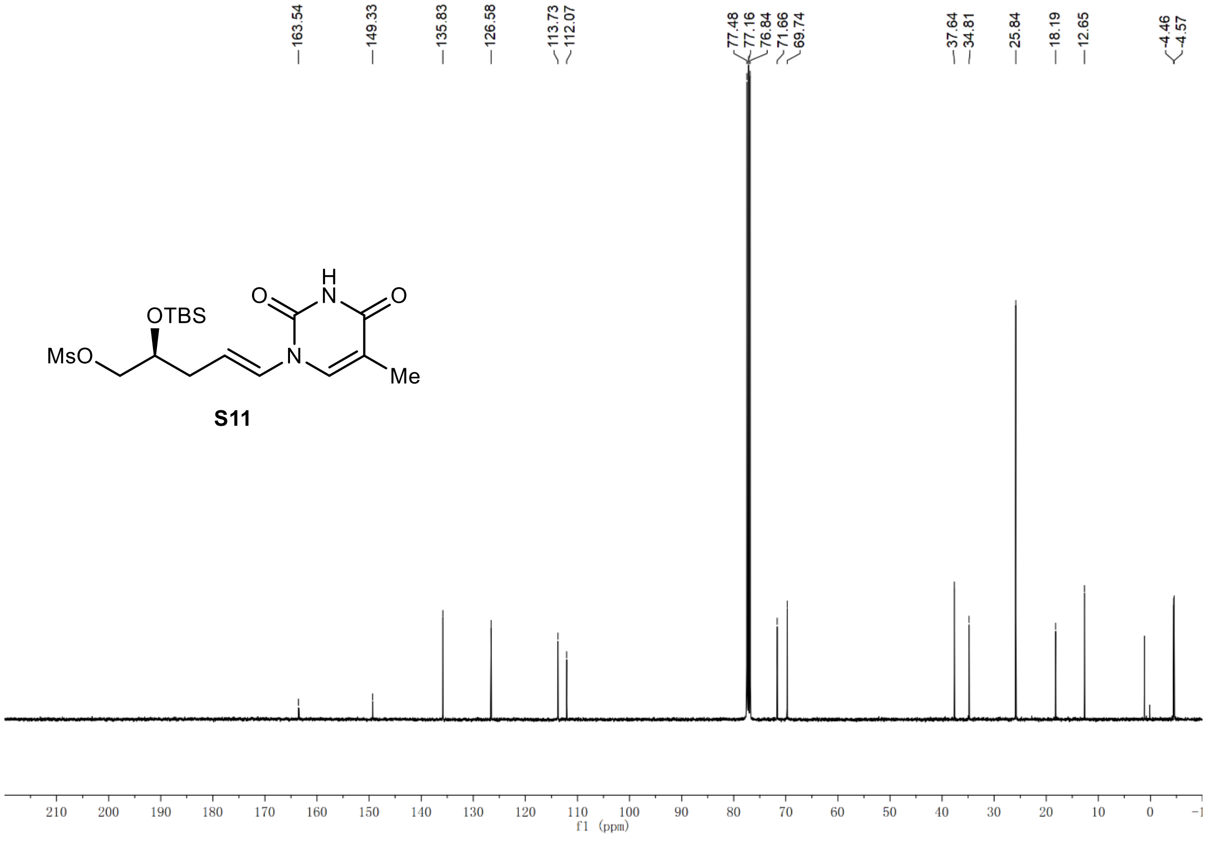

Supplementary Figure 86. ^{13}C NMR (100M, $CDCl_3$) of compound **S11.**

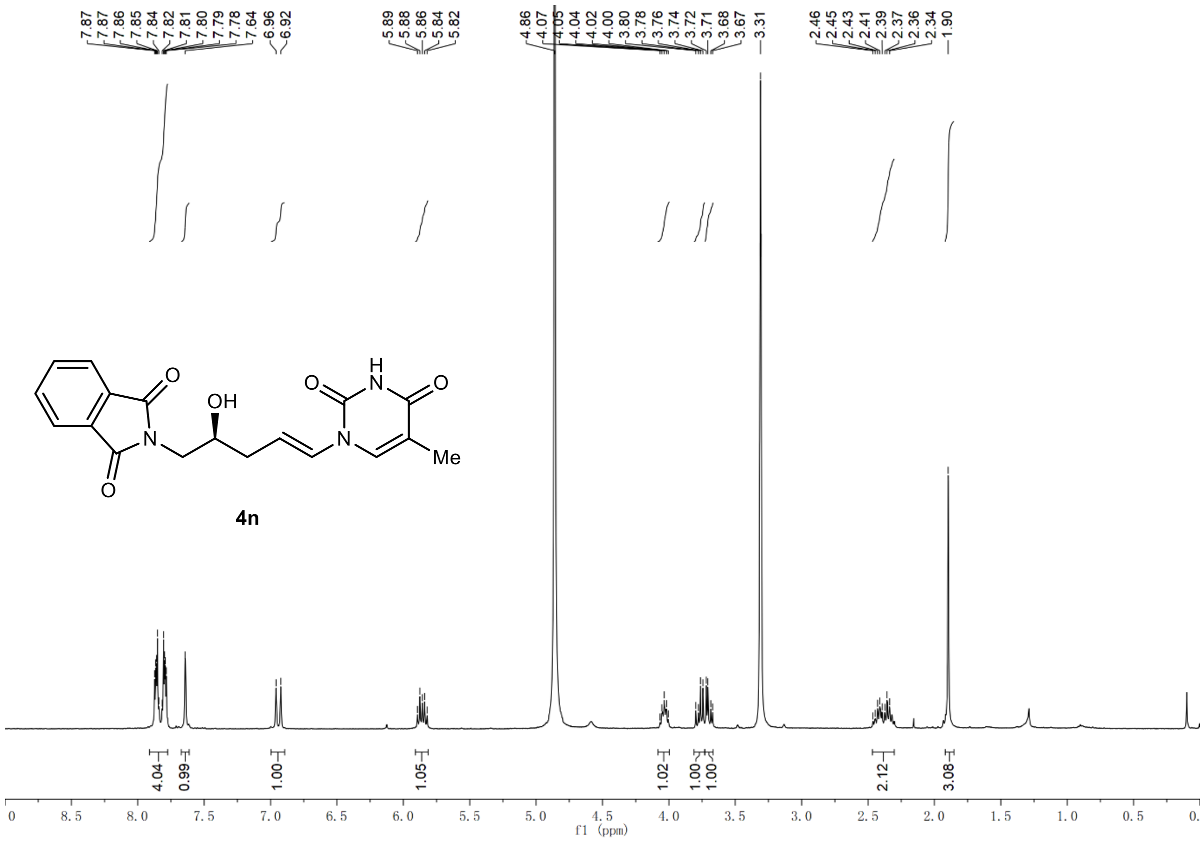

Supplementary Figure 87. ^{1}H NMR (400M, CD_3OD) of compound **4n.**

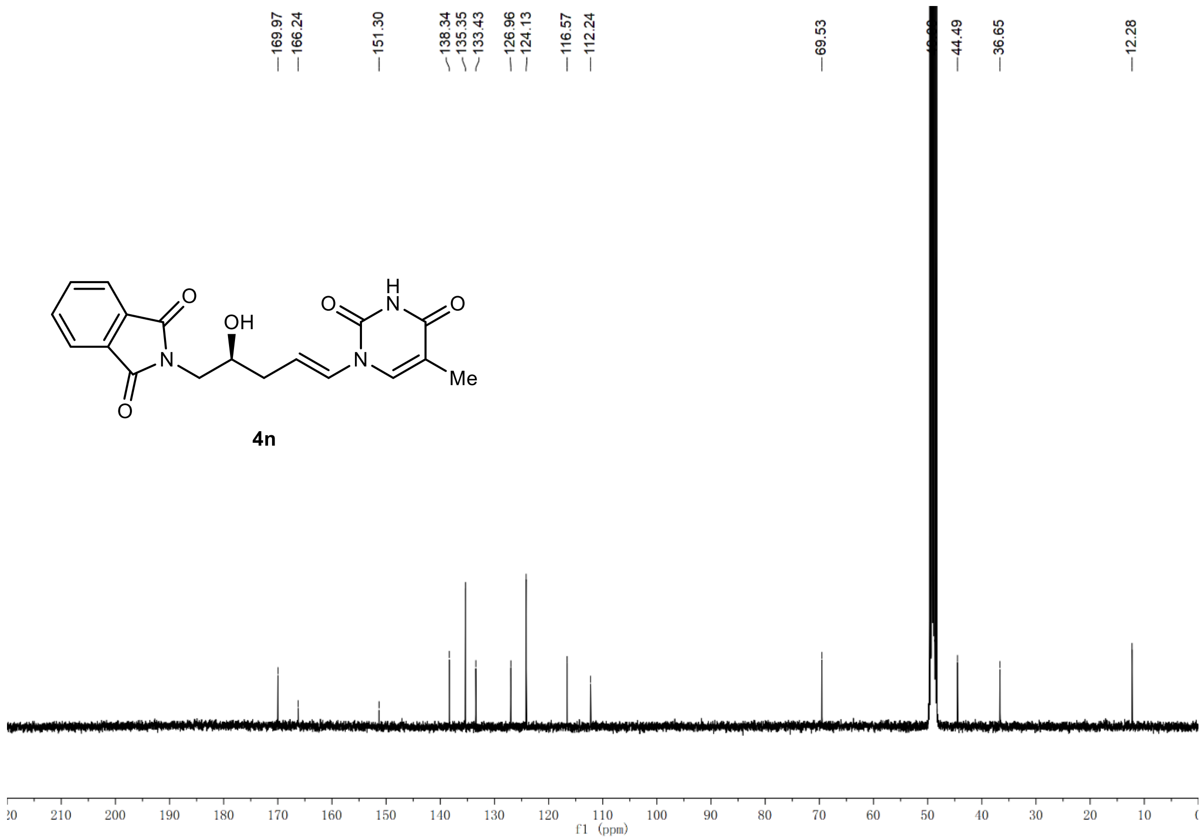

Supplementary Figure 88. ^{13}C NMR (100M, CD_3OD) of compound **4n.**

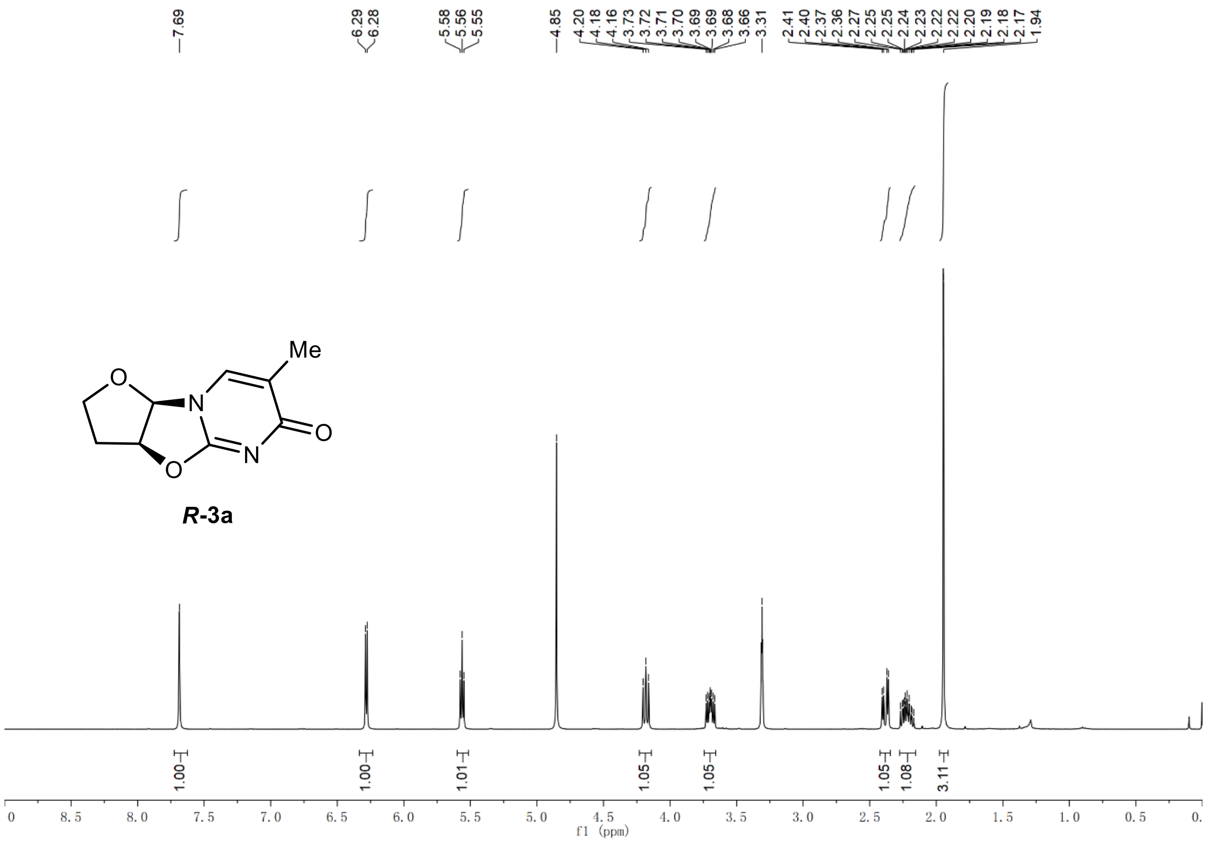

Supplementary Figure 89. ^{1}H NMR (400M, CD_3OD) of compound ***R*-3a.**

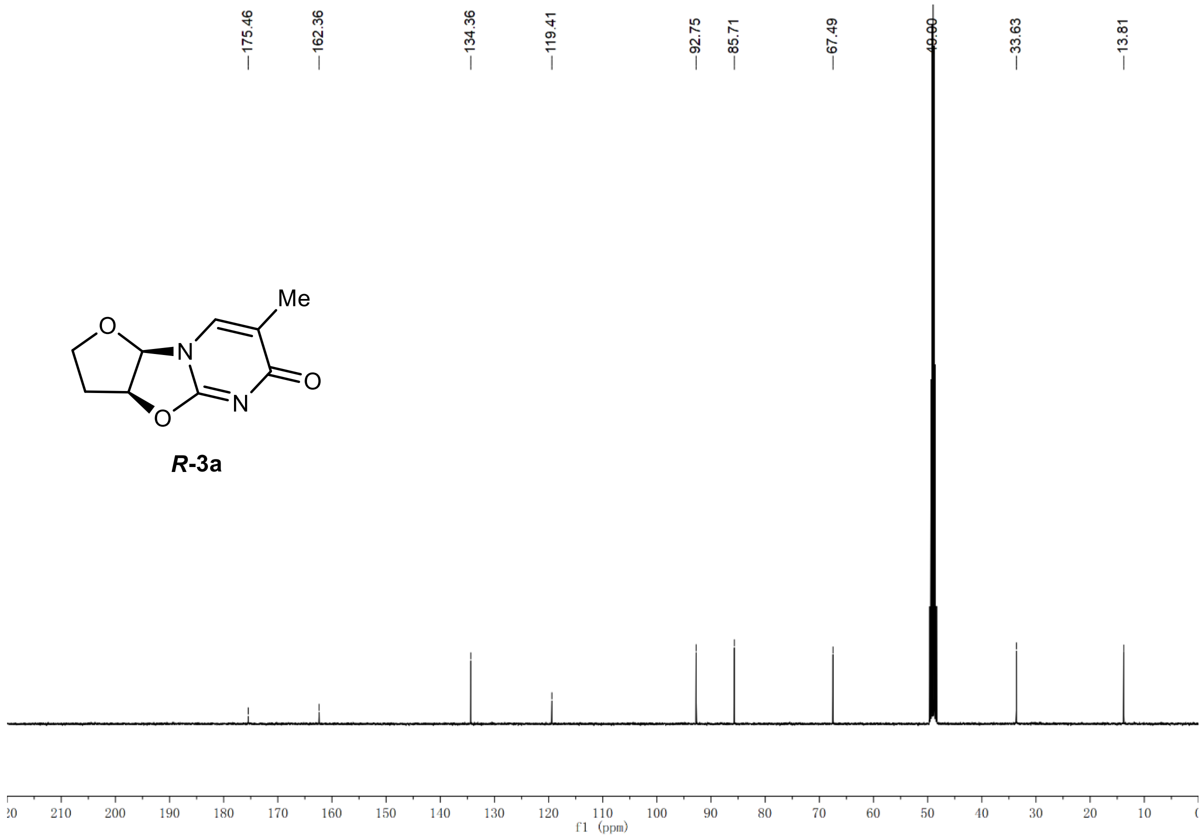

Supplementary Figure 90. ^{13}C NMR (100M, CD_3OD) of compound ***R*-3a.**

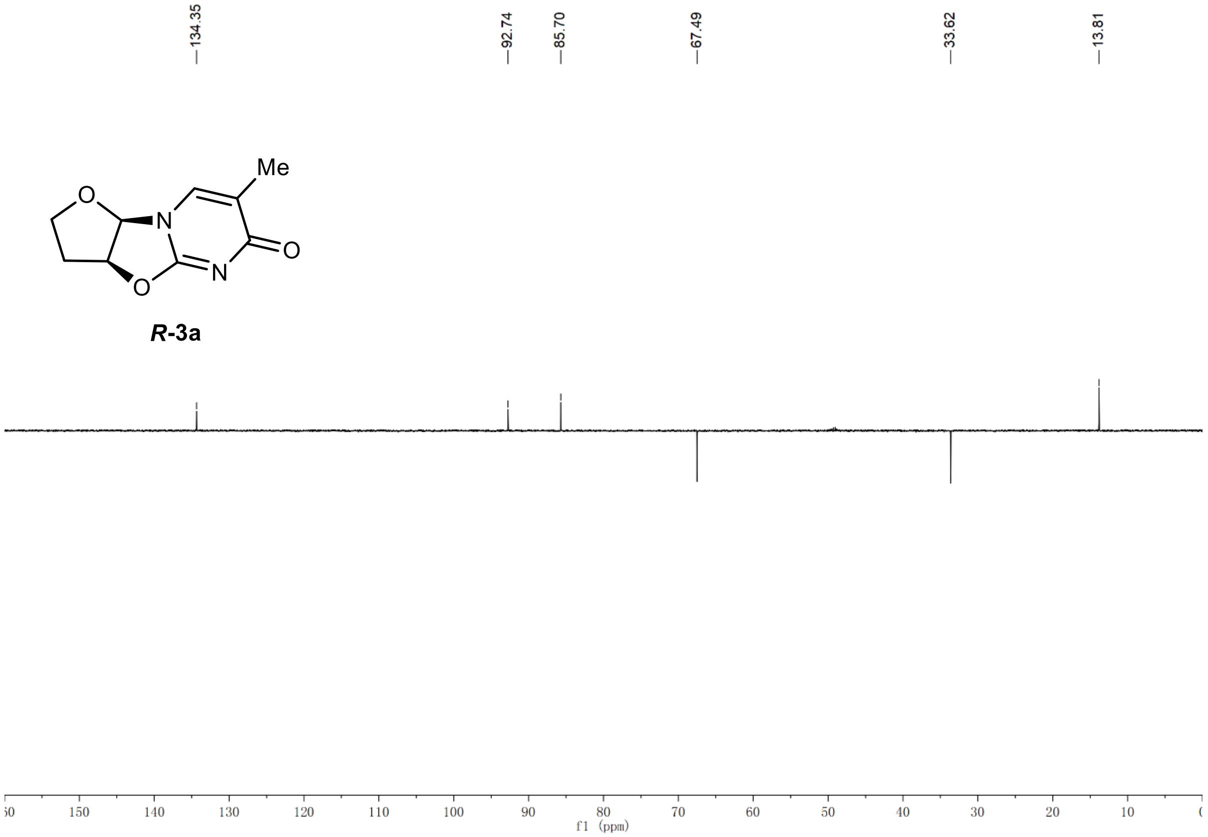

Supplementary Figure 91. DEPT 135° (100M, CD_3OD) of compound ***R*-3a.**

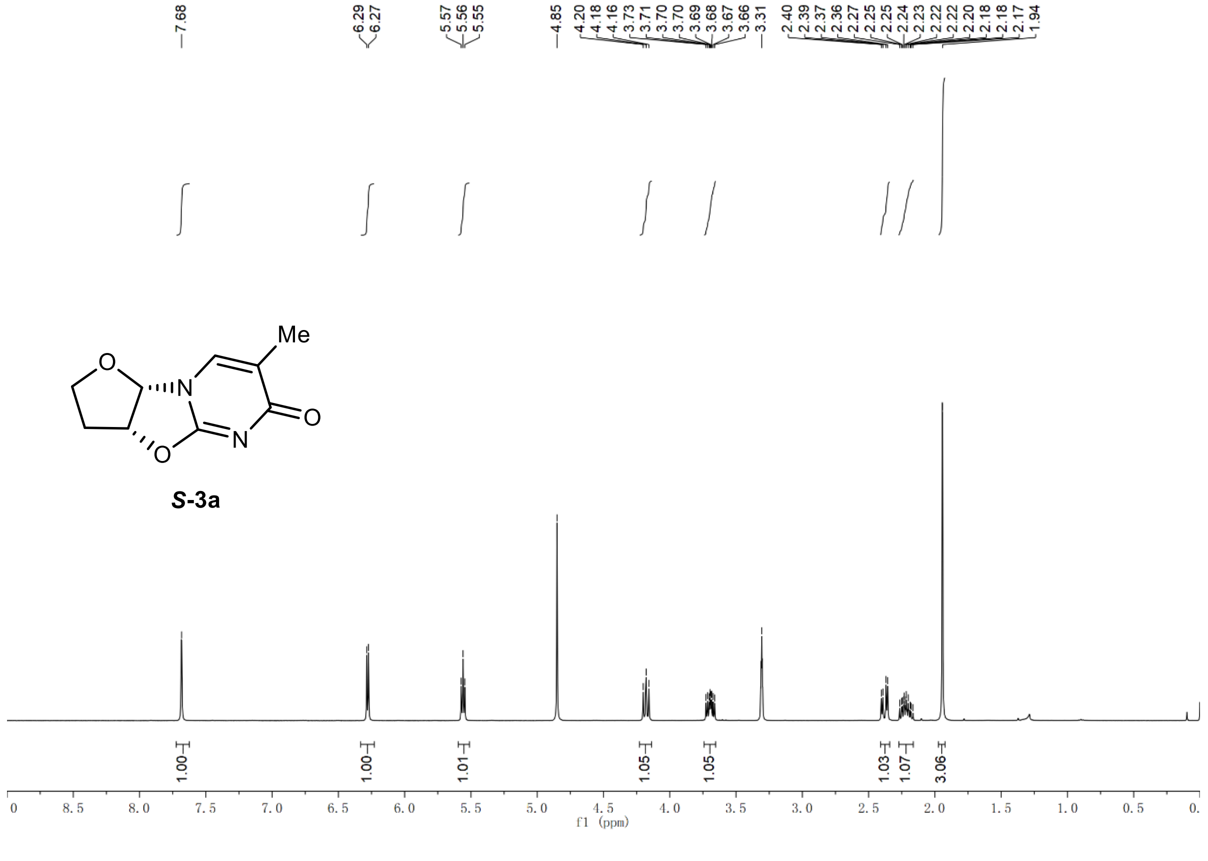

Supplementary Figure 92. ^{1}H NMR (400M, CD_3OD) of compound ***S*-3a.**

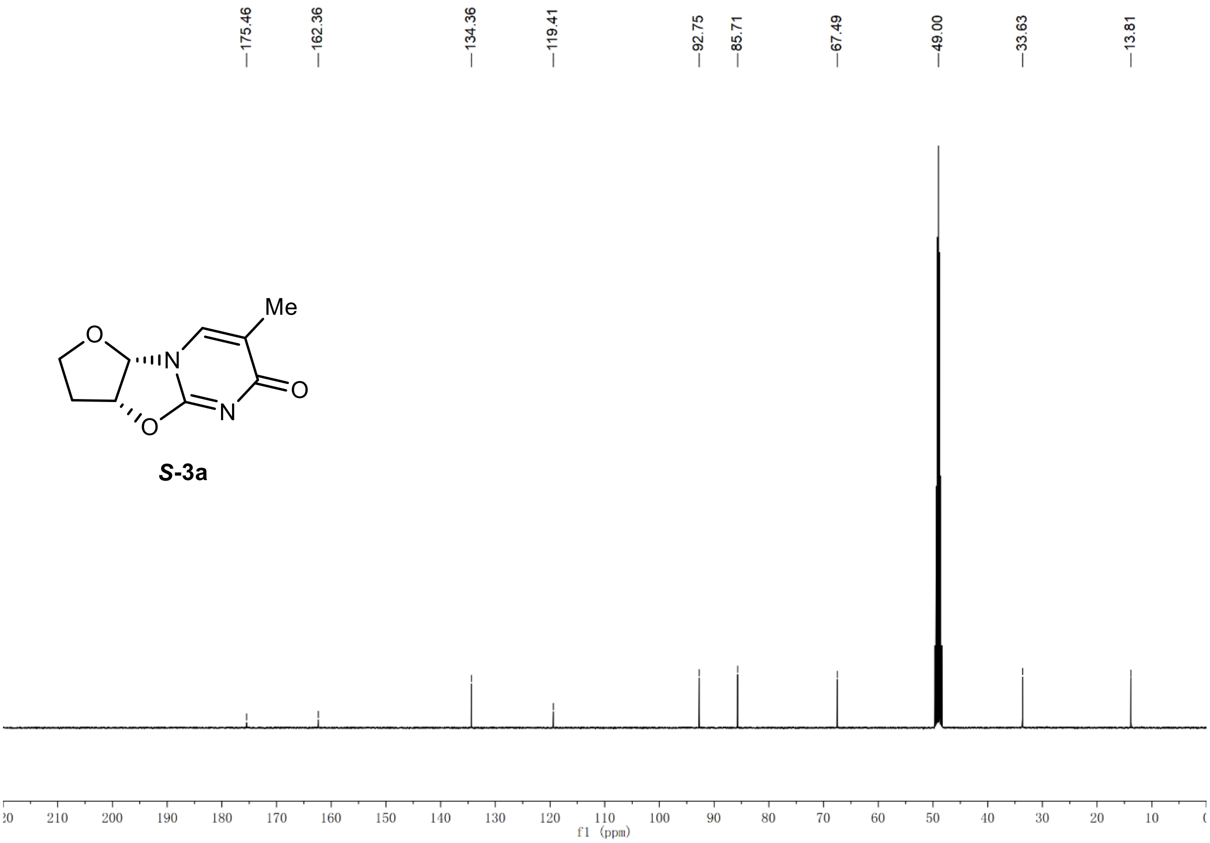

Supplementary Figure 93. ^{13}C NMR (100M, CD_3OD) of compound ***S*-3a.**

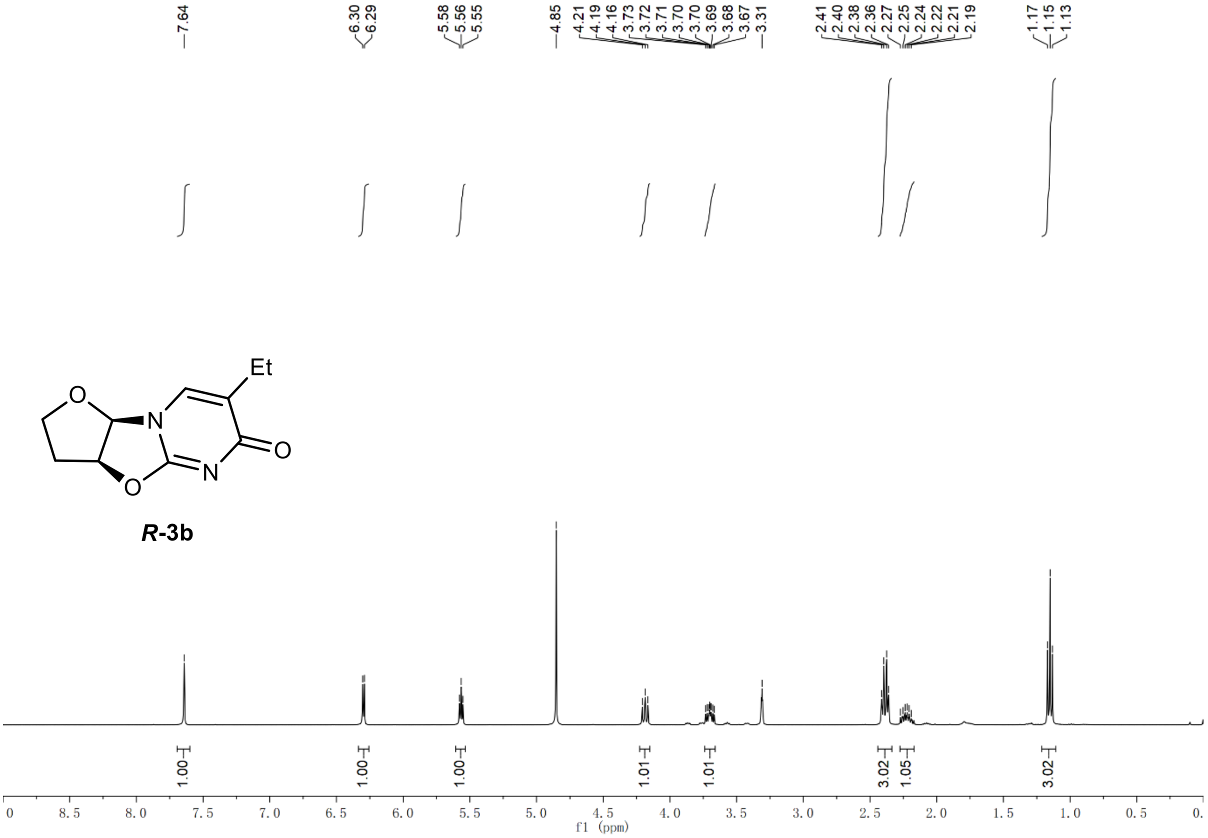

Supplementary Figure 94. ^{1}H NMR (400M, CD_3OD) of compound ***R*-3b.**

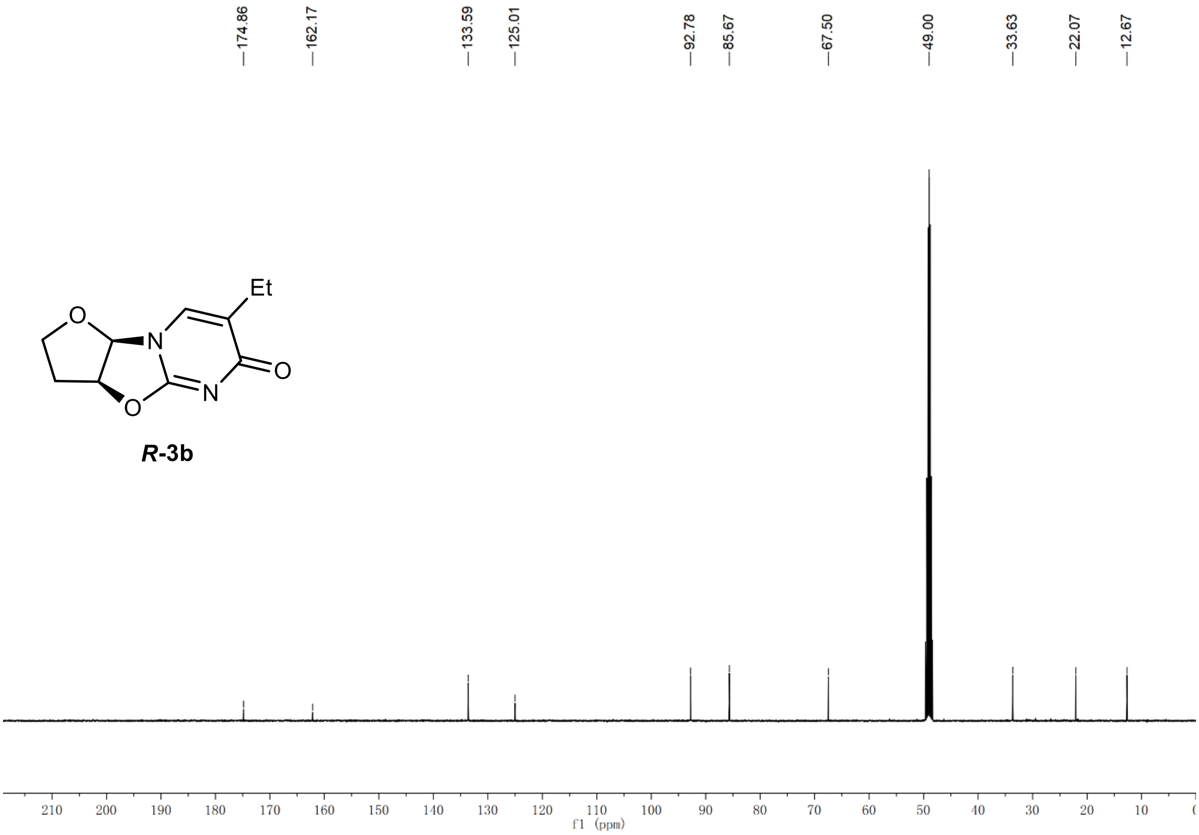

Supplementary Figure 95. ^{13}C NMR (100M, CD_3OD) of compound ***R*-3b.**

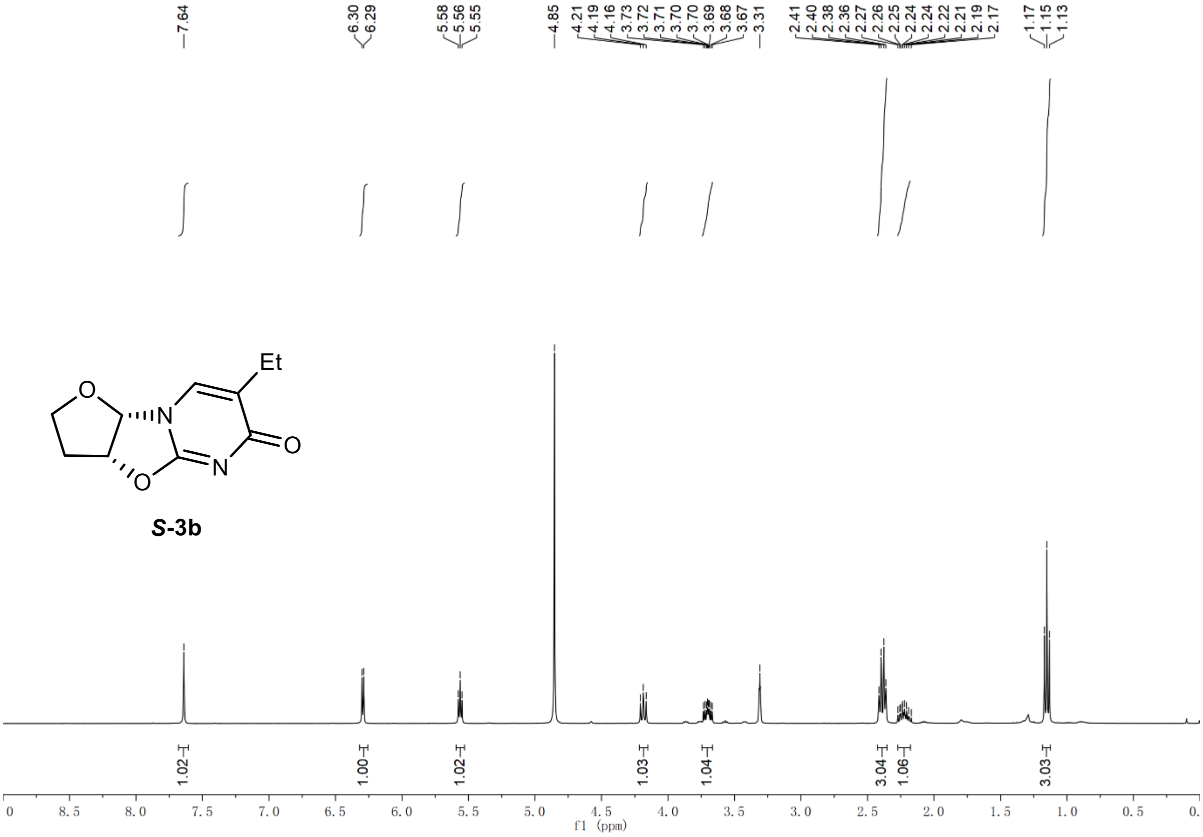

Supplementary Figure 96. ^{1}H NMR (400M, CD_3OD) of compound ***S*-3b.**

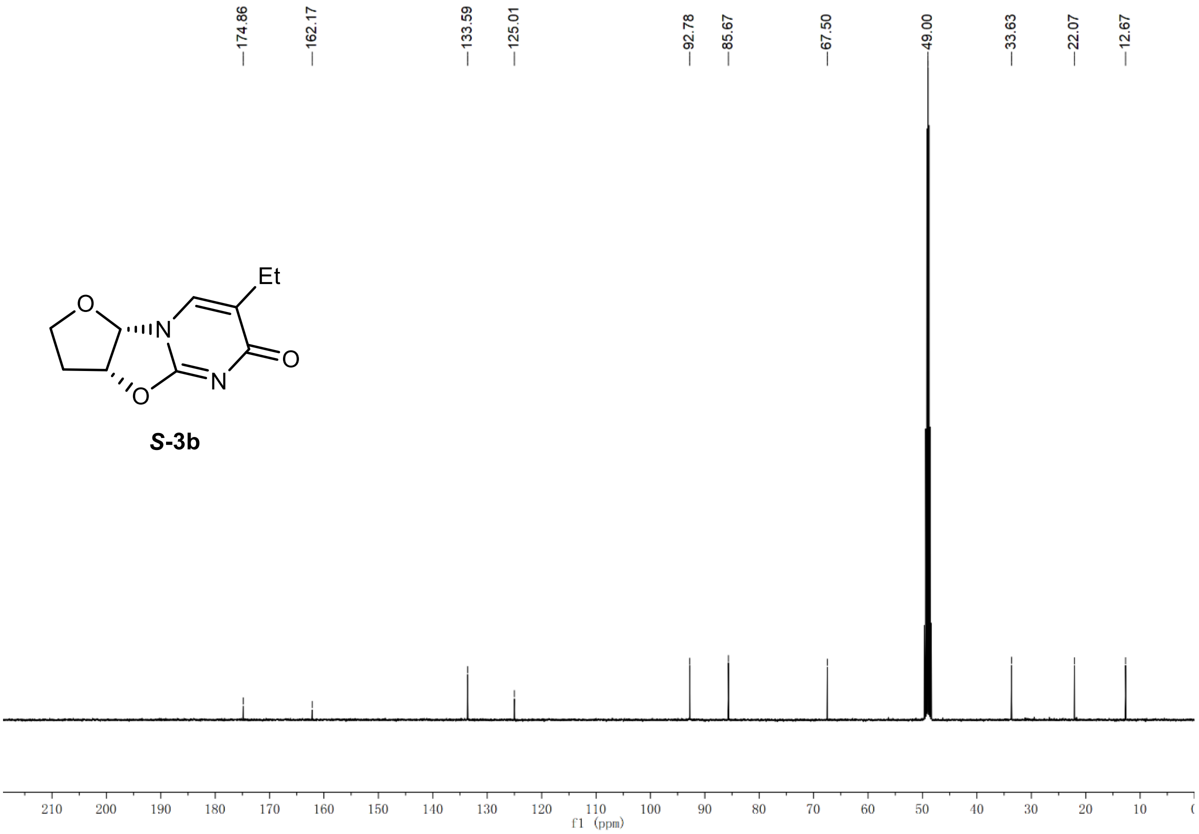

Supplementary Figure 97. ^{13}C NMR (100M, CD_3OD) of compound ***S*-3b.**

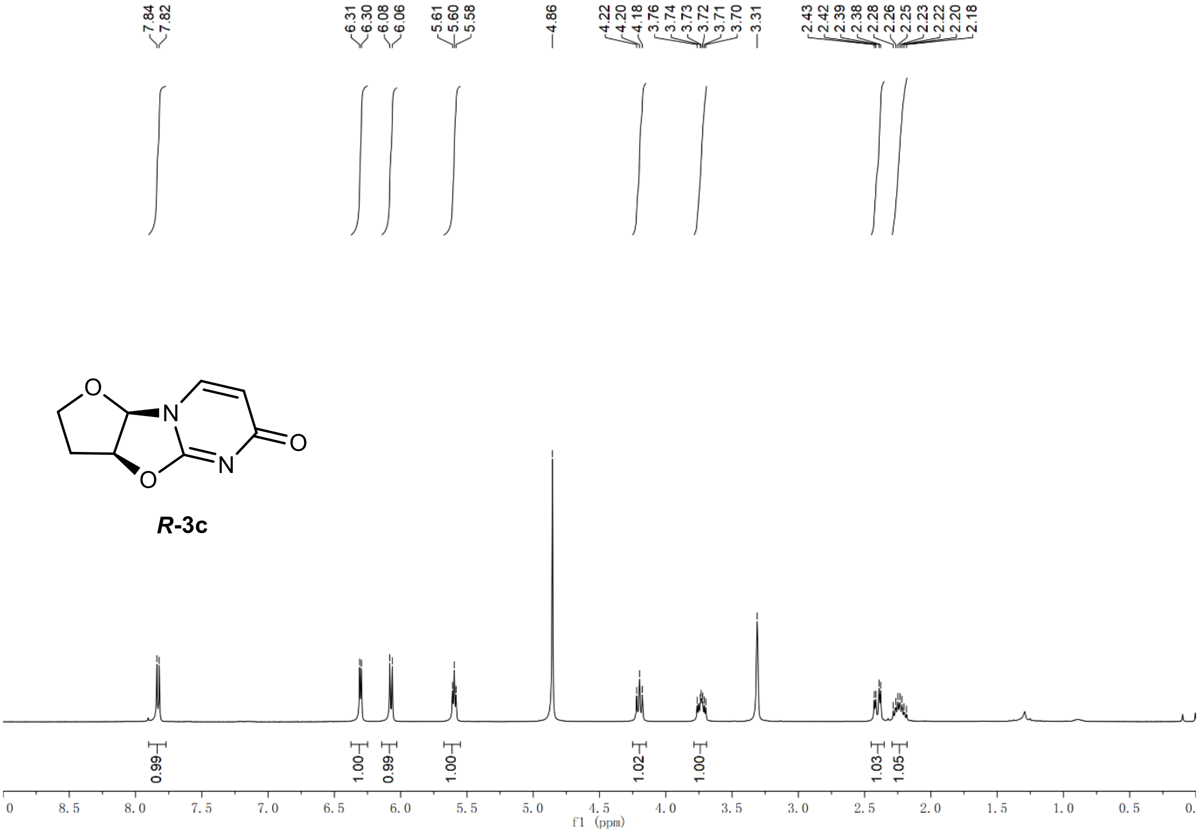

Supplementary Figure 98. ^{1}H NMR (400M, CD_3OD) of compound ***R*-3c.**

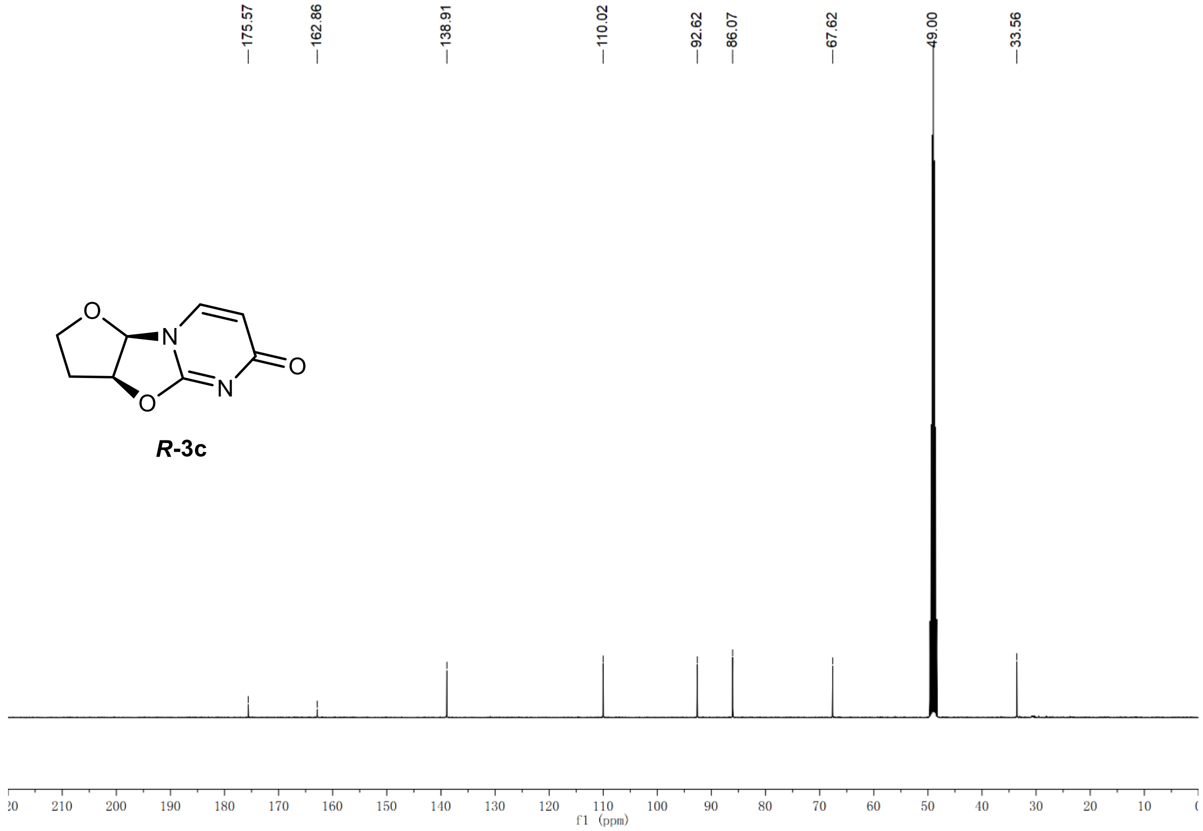

Supplementary Figure 99. ^{13}C NMR (100M, CD_3OD) of compound ***R*-3c.**

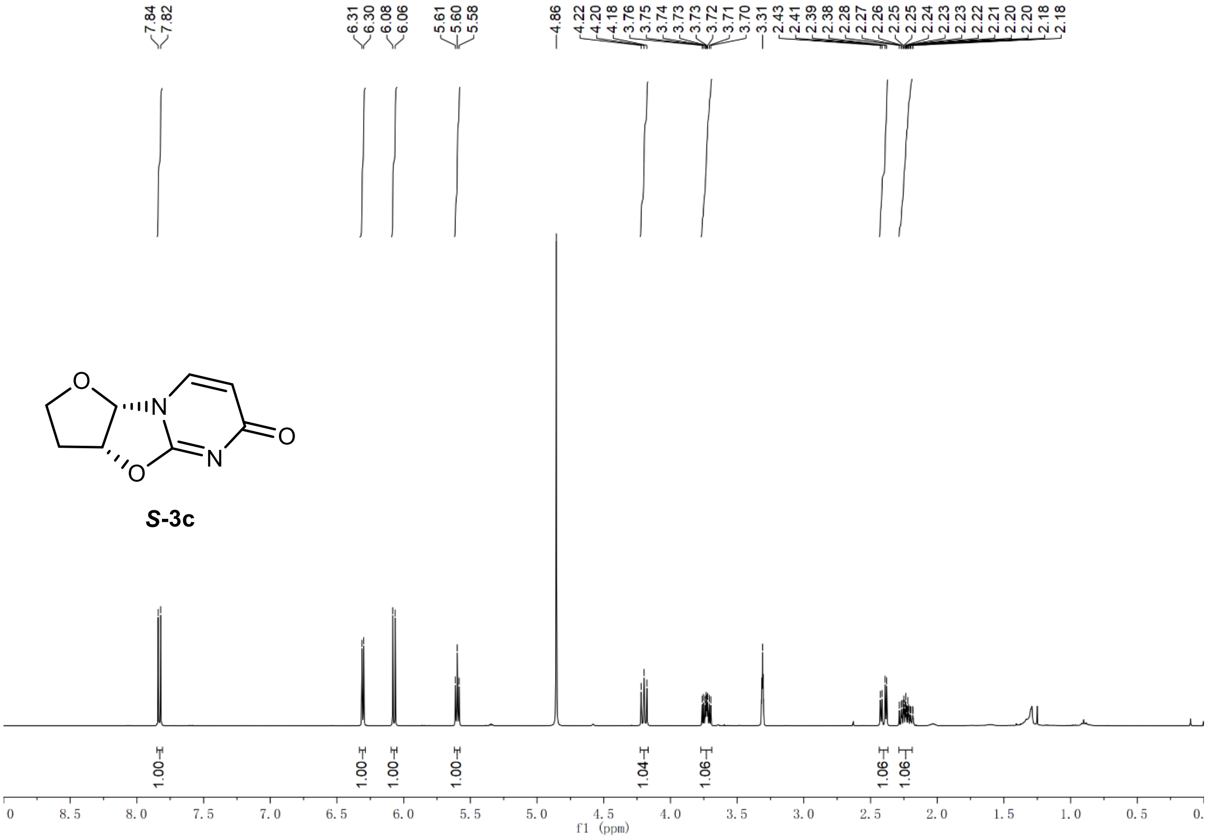

Supplementary Figure 100. ^{1}H NMR (400M, CD_3OD) of compound ***S*-3c.**

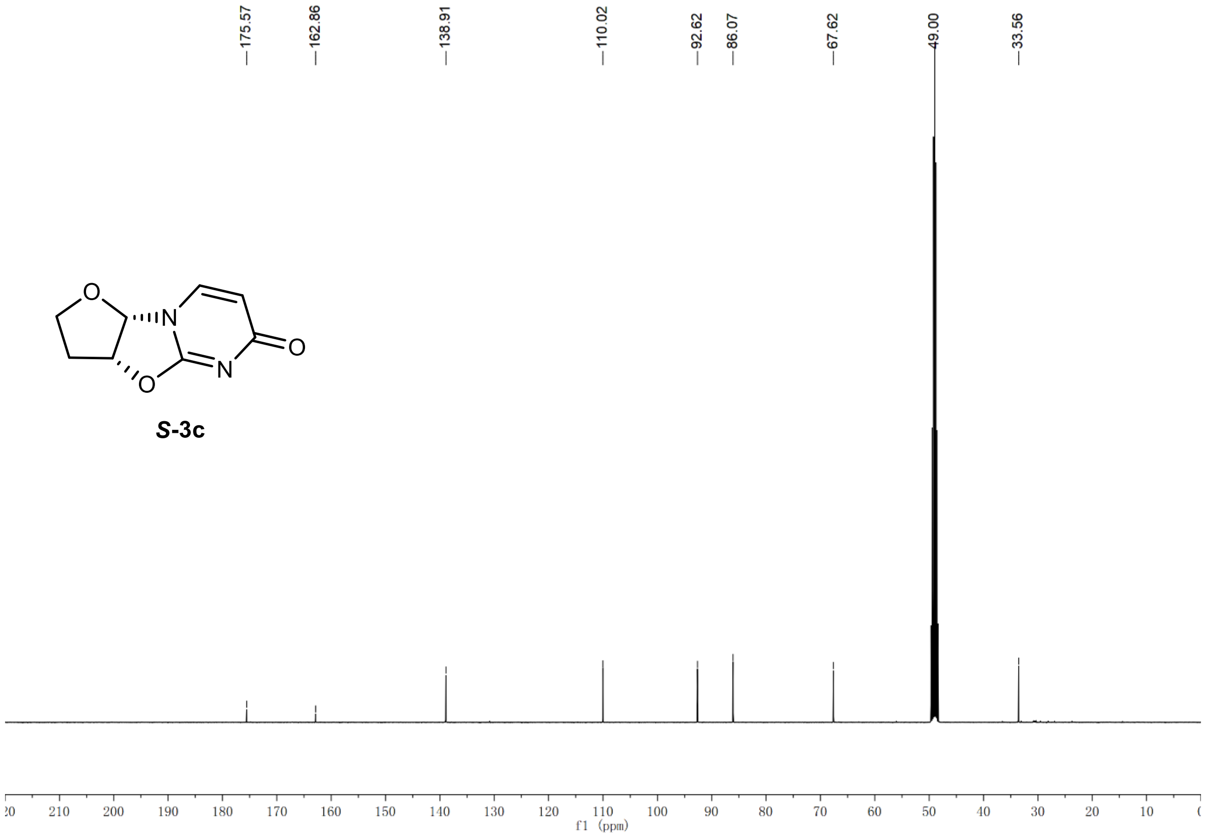

Supplementary Figure 101. ^{13}C NMR (100M, CD_3OD) of compound ***S*-3c.**

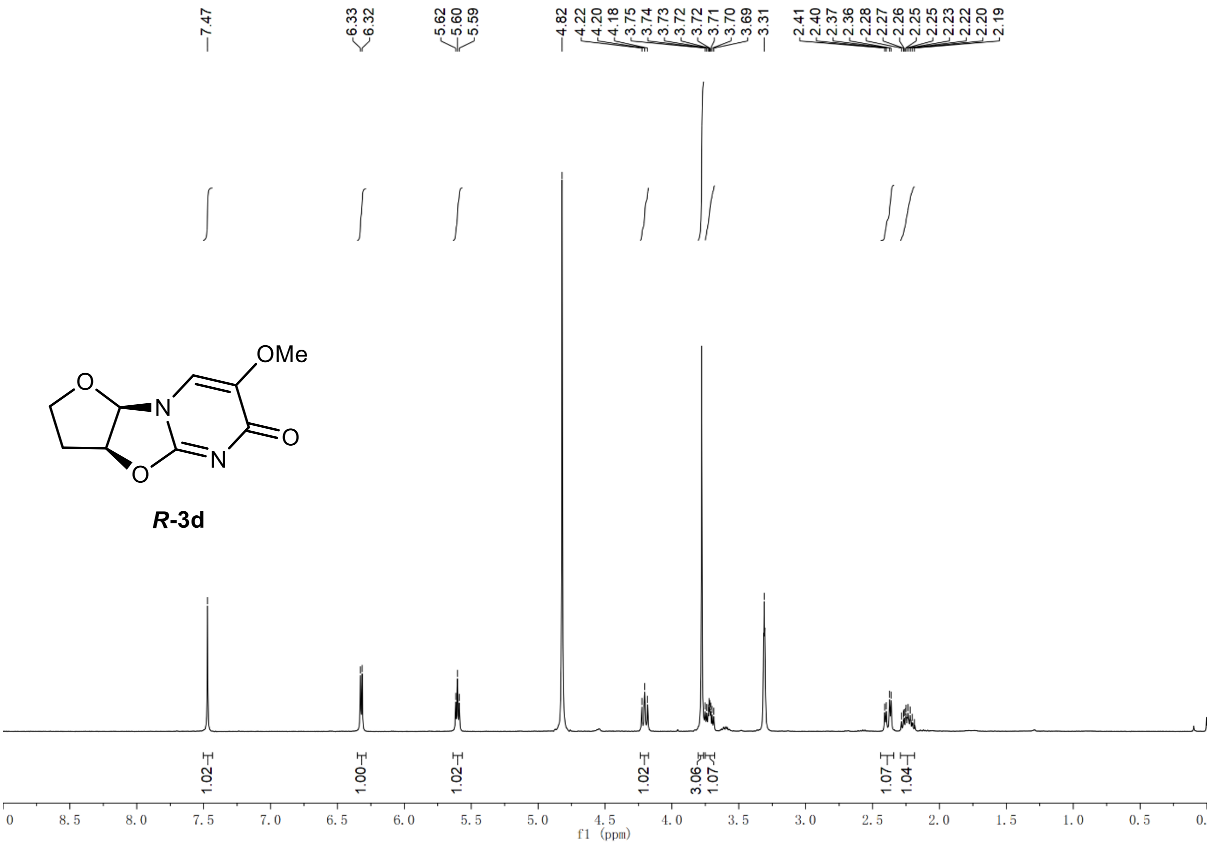

Supplementary Figure 102. ^{1}H NMR (400M, CD_3OD) of compound ***R*-3d.**

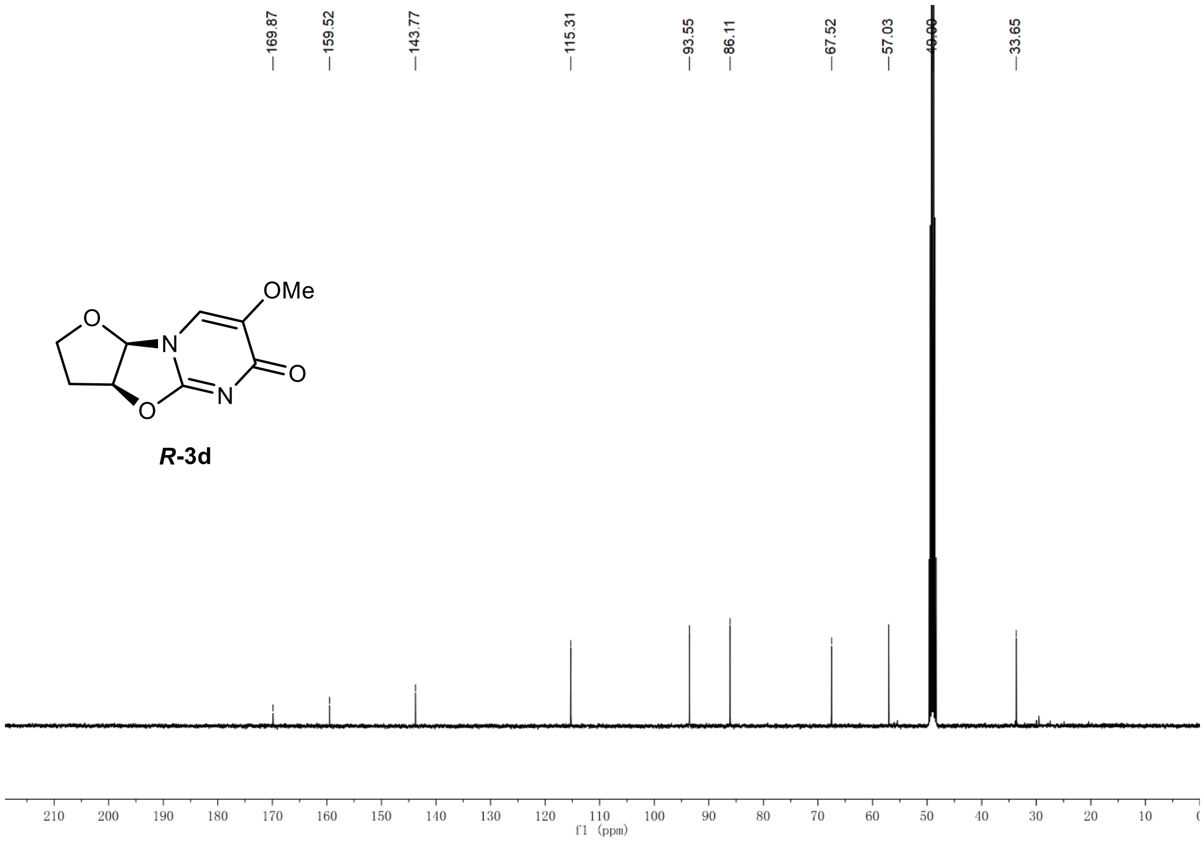

Supplementary Figure 103. ^{13}C NMR (100M, CD_3OD) of compound ***R*-3d.**

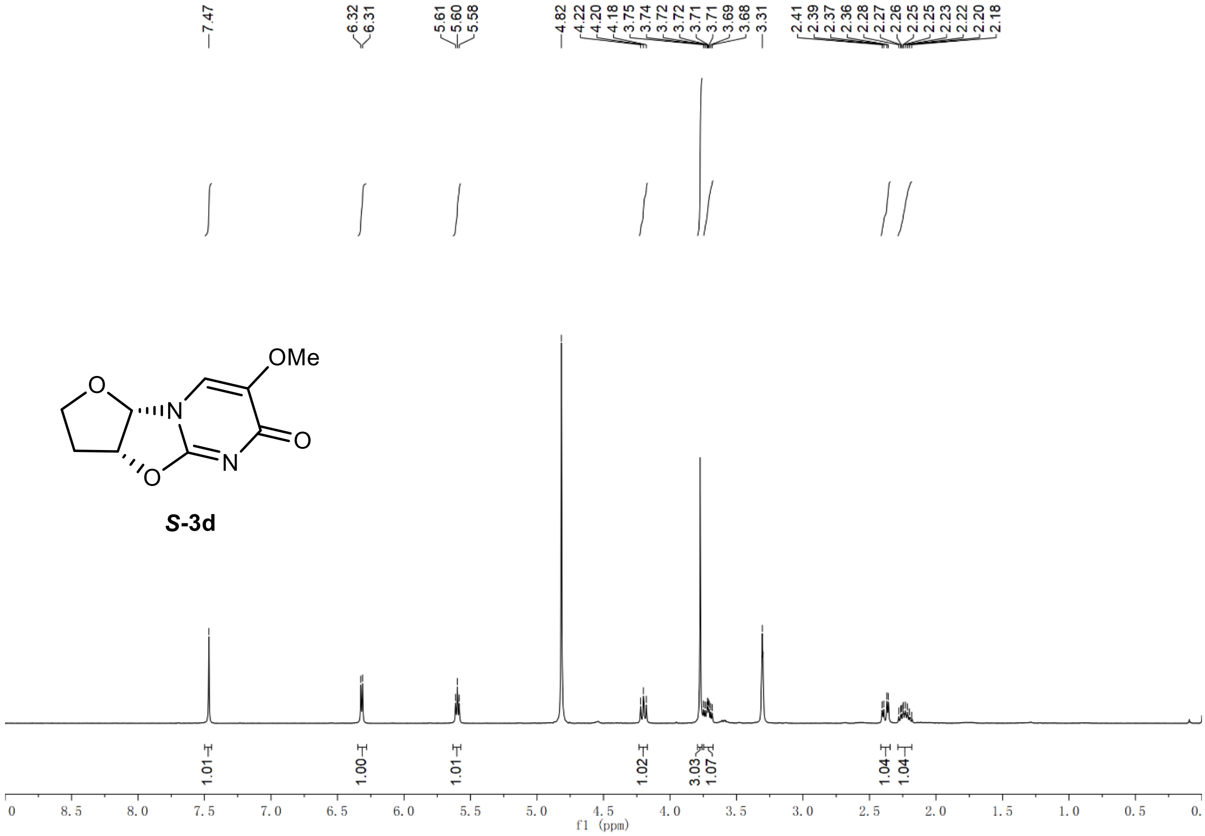

Supplementary Figure 104. ^{1}H NMR (400M, CD_3OD) of compound ***S*-3d.**

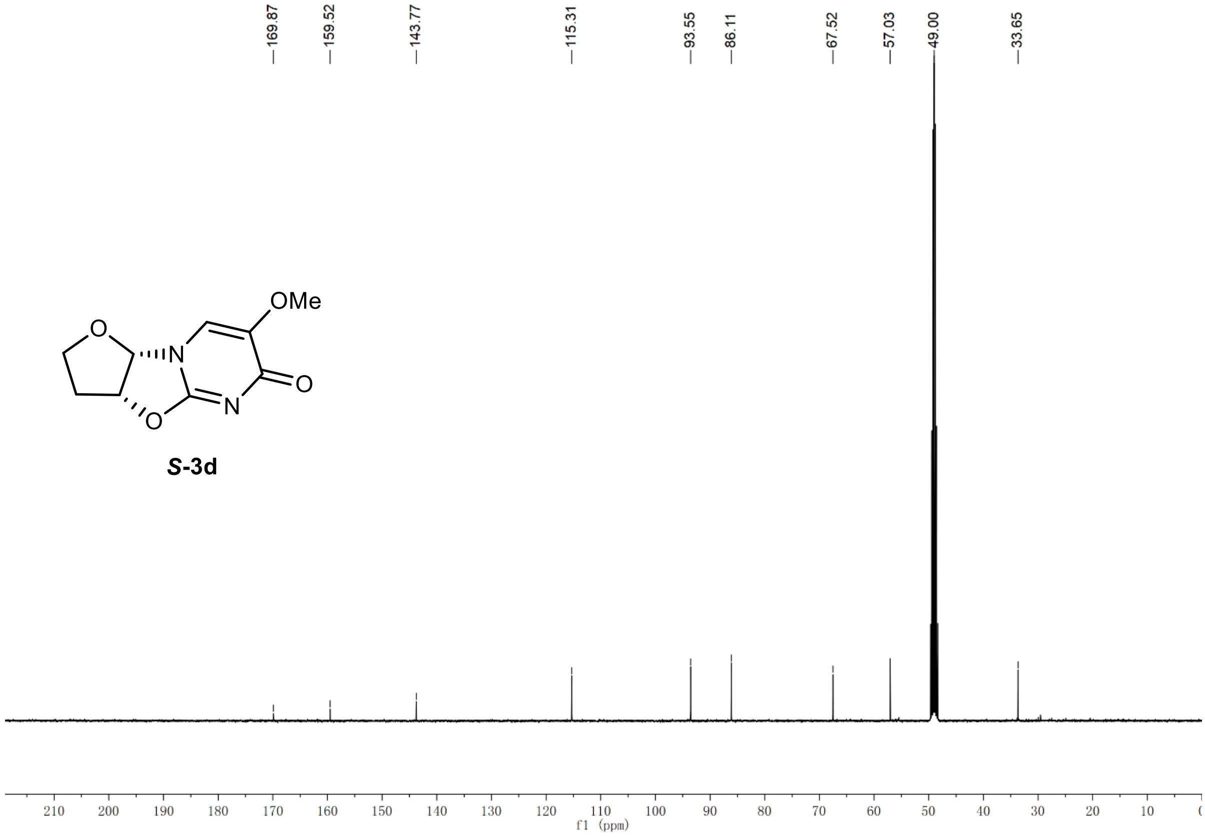

Supplementary Figure 105. ^{13}C NMR (100M, CD_3OD) of compound ***S*-3d.**

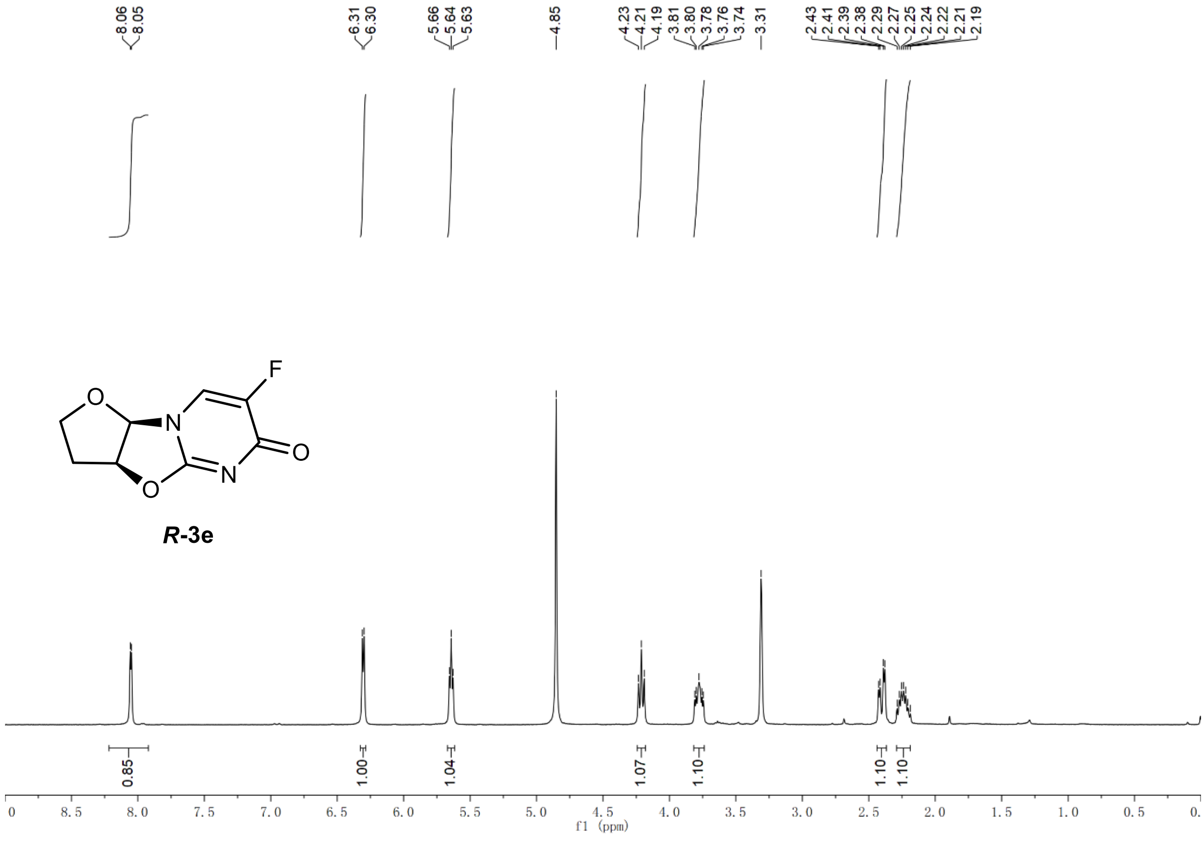

Supplementary Figure 106. ^{1}H NMR (400M, CD_3OD) of compound ***R*-3e.**

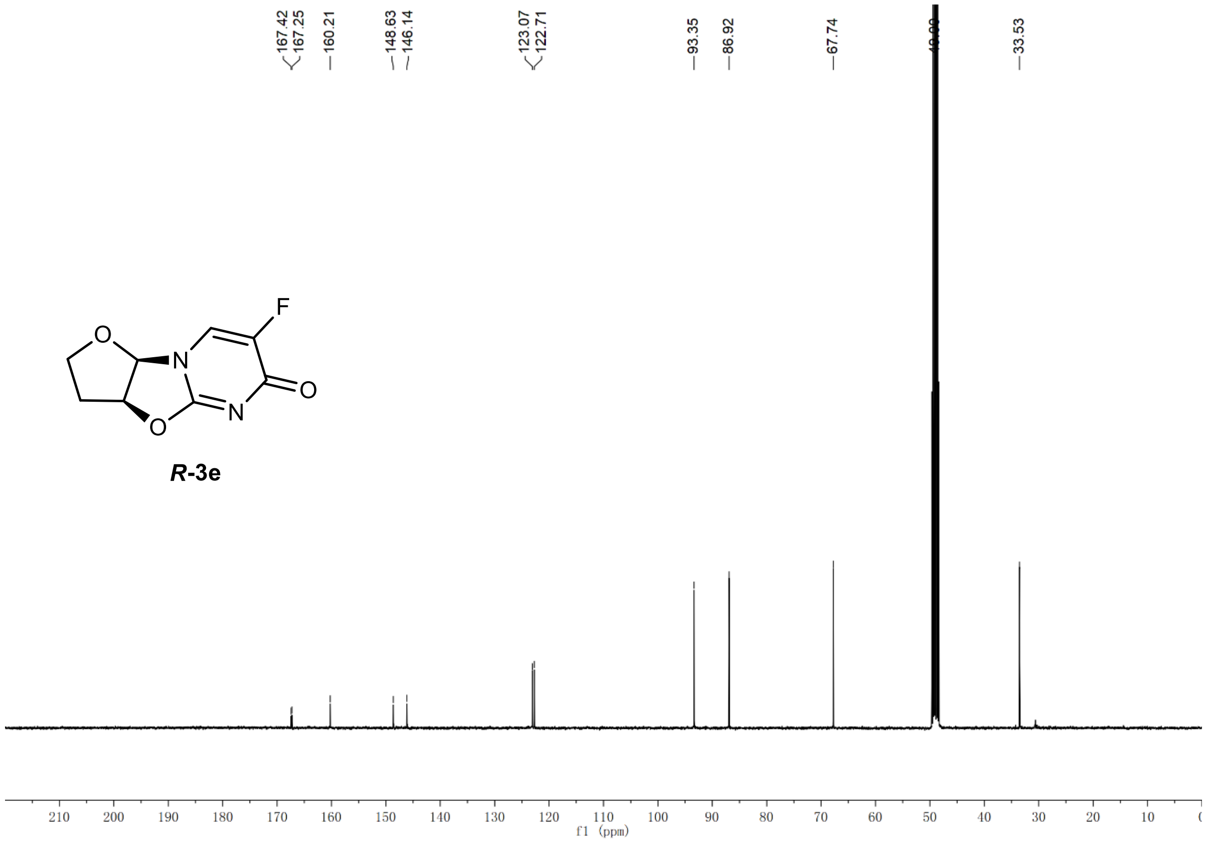

Supplementary Figure 107. ^{13}C NMR (100M, CD_3OD) of compound ***R*-3e.**

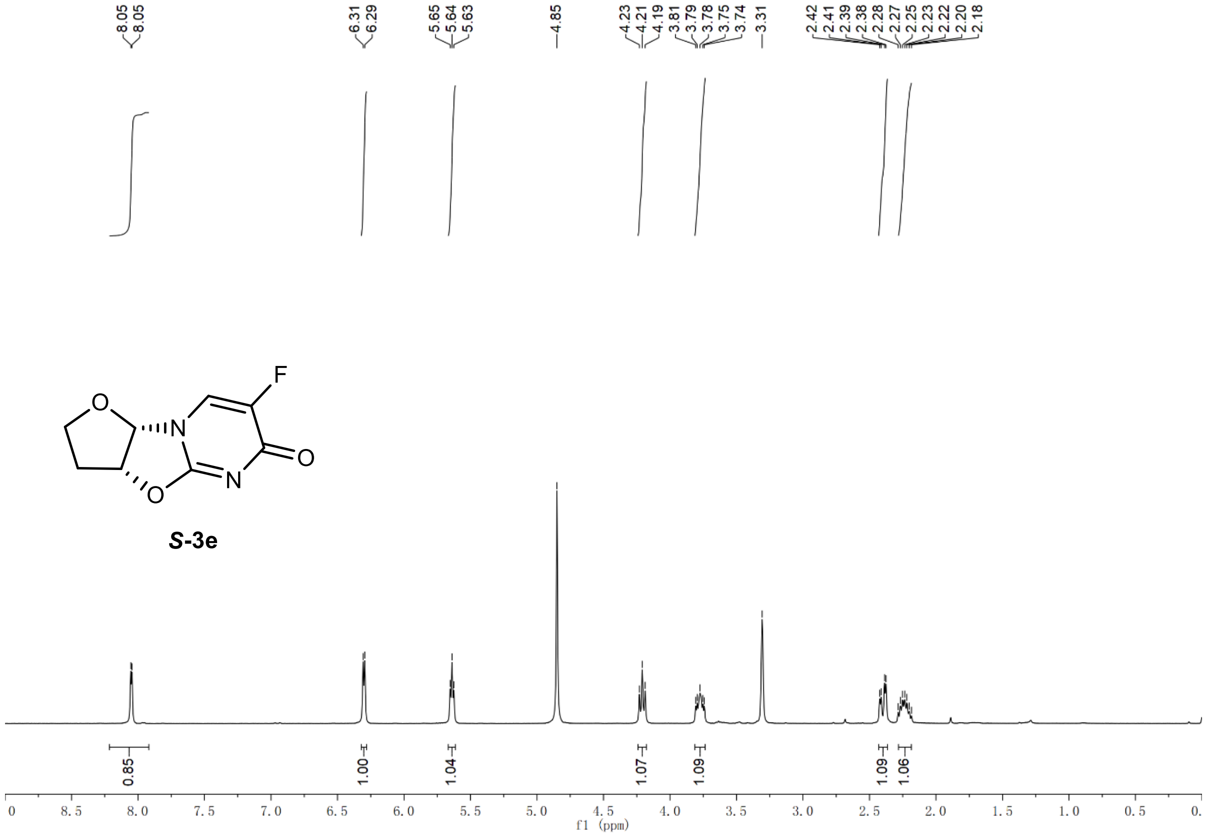

Supplementary Figure 108. ^{1}H NMR (400M, CD_3OD) of compound ***S*-3e.**

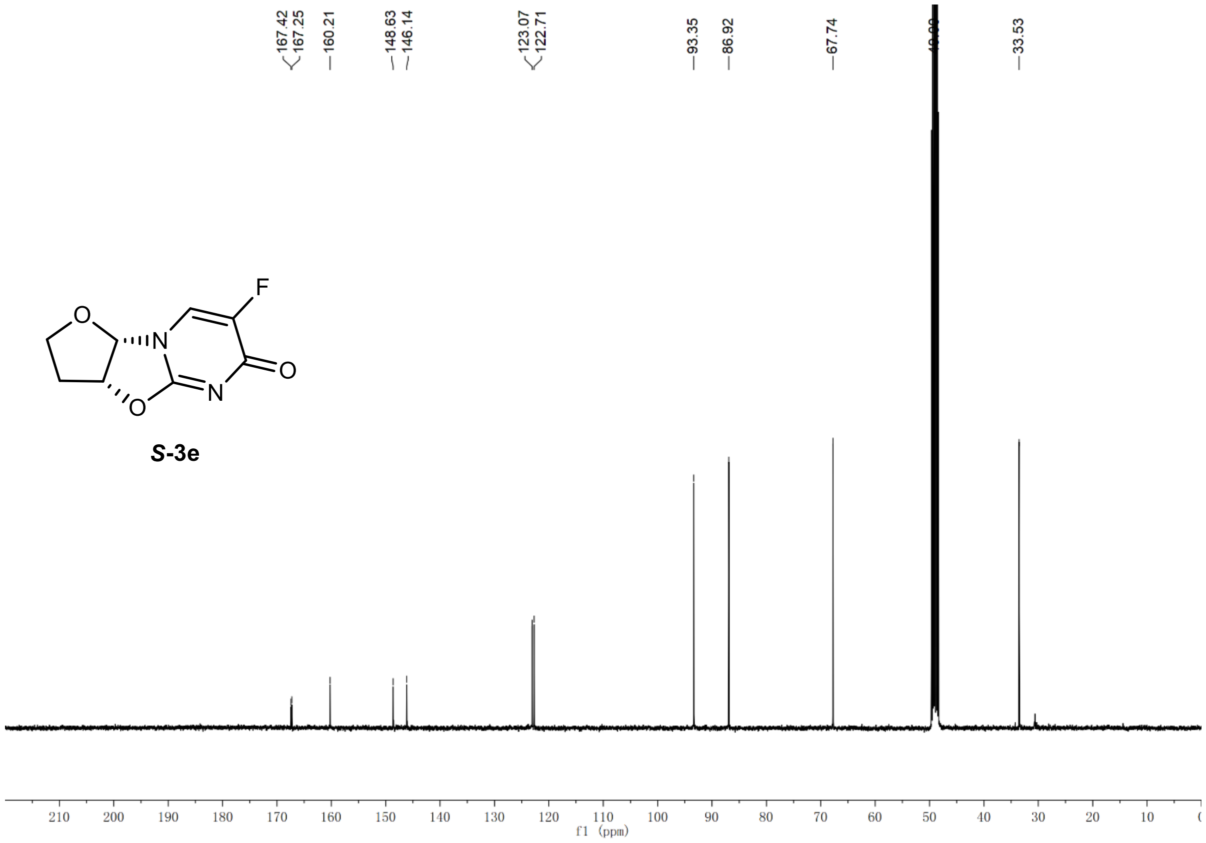

Supplementary Figure 109. ^{13}C NMR (100M, CD_3OD) of compound ***S*-3e.**

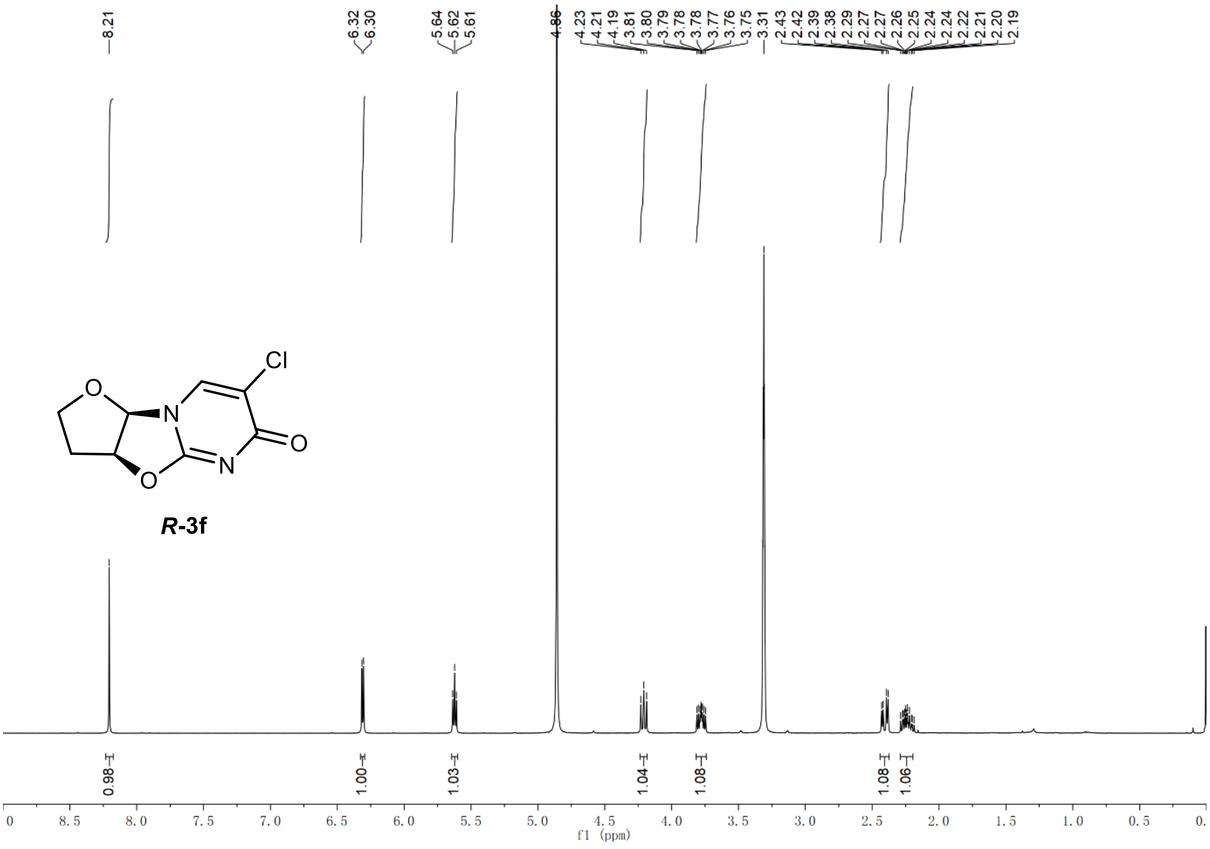

Supplementary Figure 110. ^{1}H NMR (400M, CD_3OD) of compound ***R*-3f.**

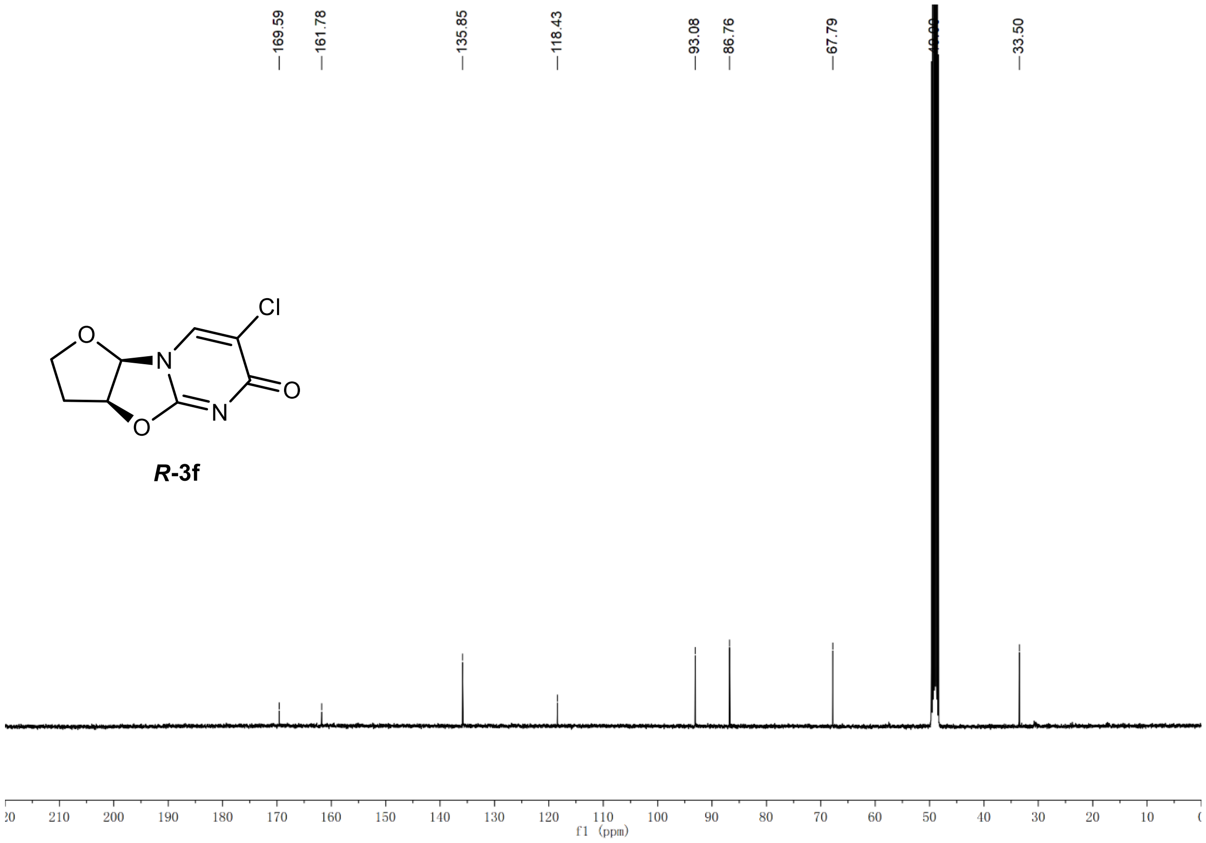

Supplementary Figure 111. ^{13}C NMR (100M, CD_3OD) of compound ***R*-3f.**

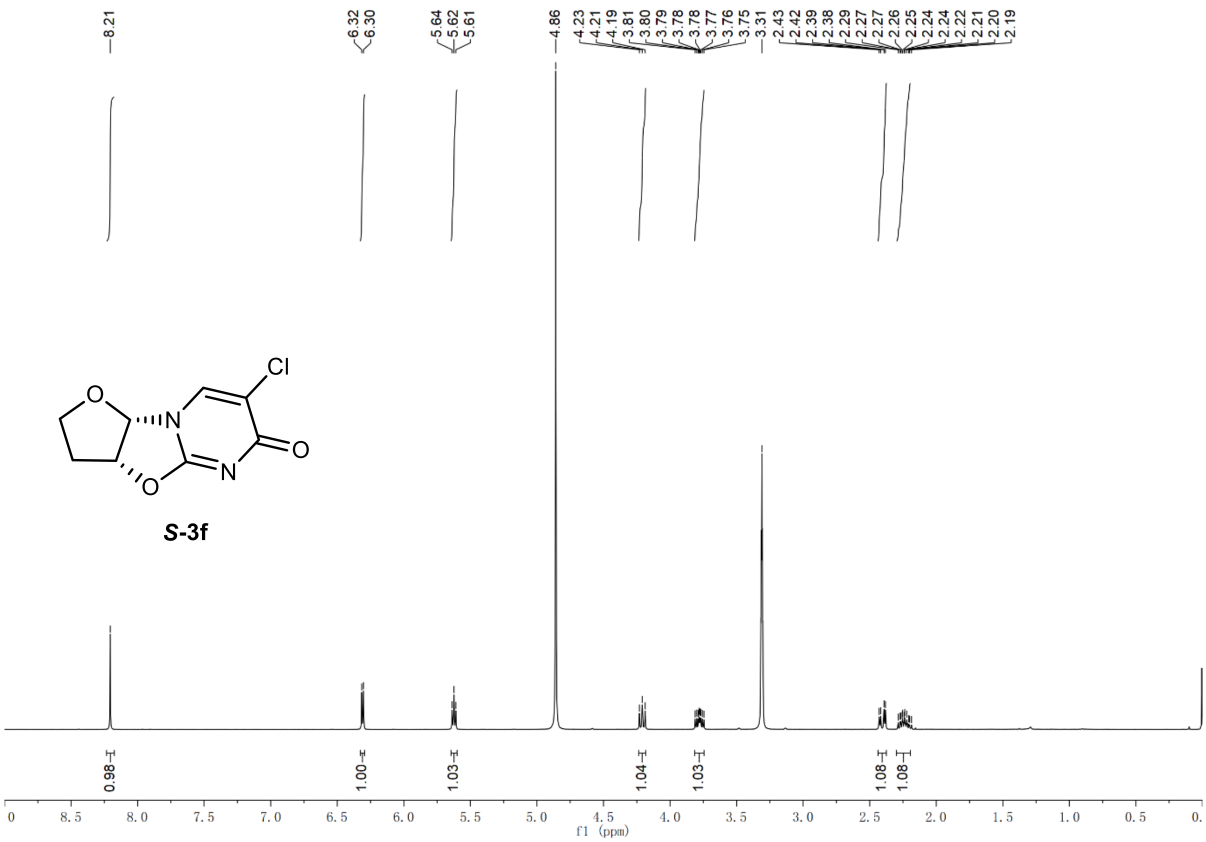

Supplementary Figure 112. ^{1}H NMR (400M, CD_3OD) of compound ***S*-3f.**

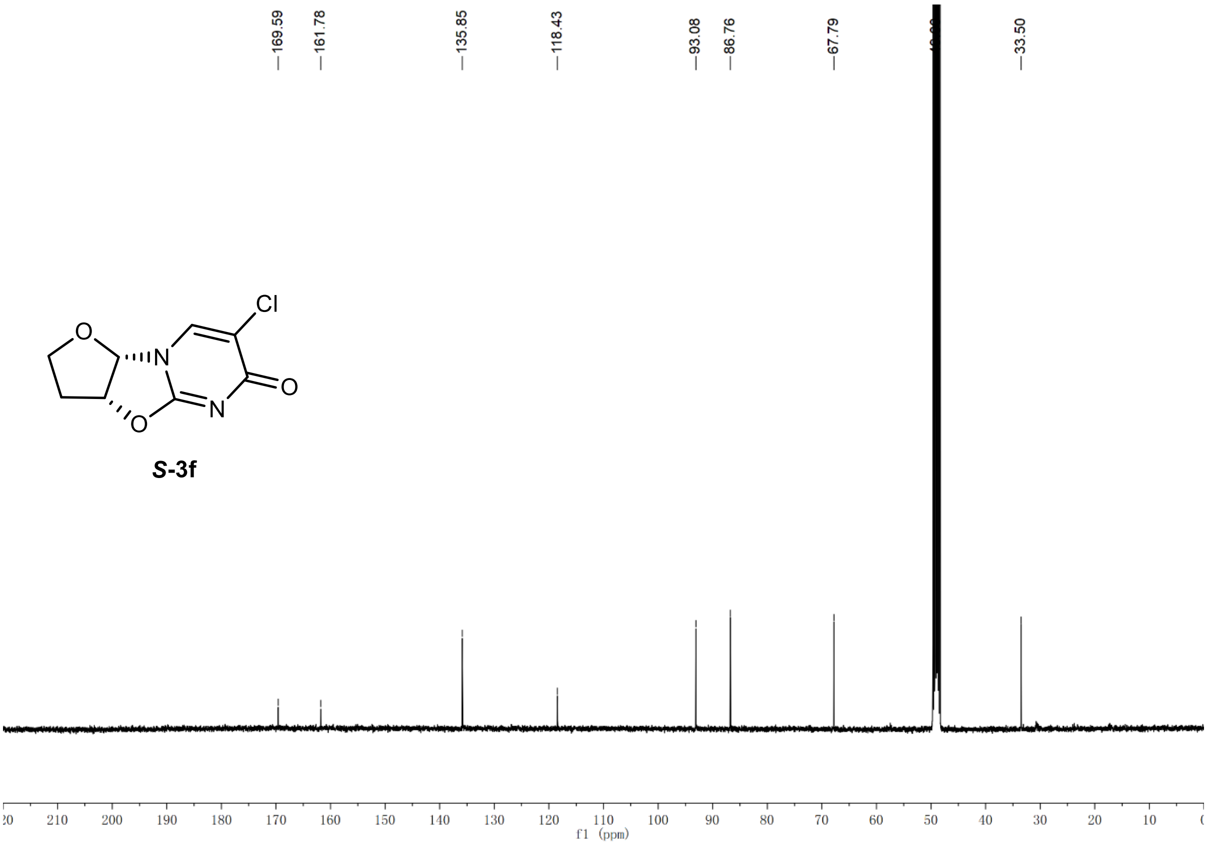

Supplementary Figure 113. ^{13}C NMR (100M, CD_3OD) of compound ***S*-3f.**

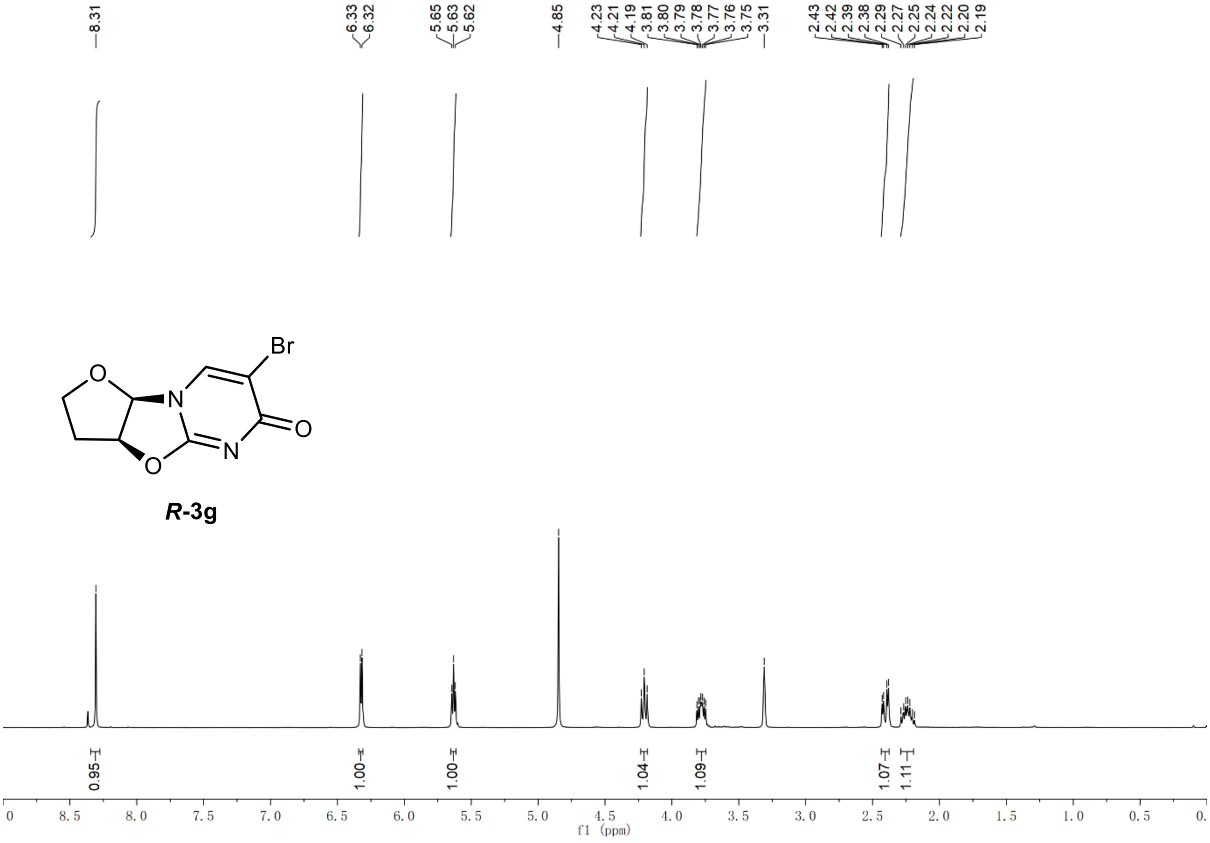

Supplementary Figure 114. ^{1}H NMR (400M, CD_3OD) of compound ***R*-3g.**

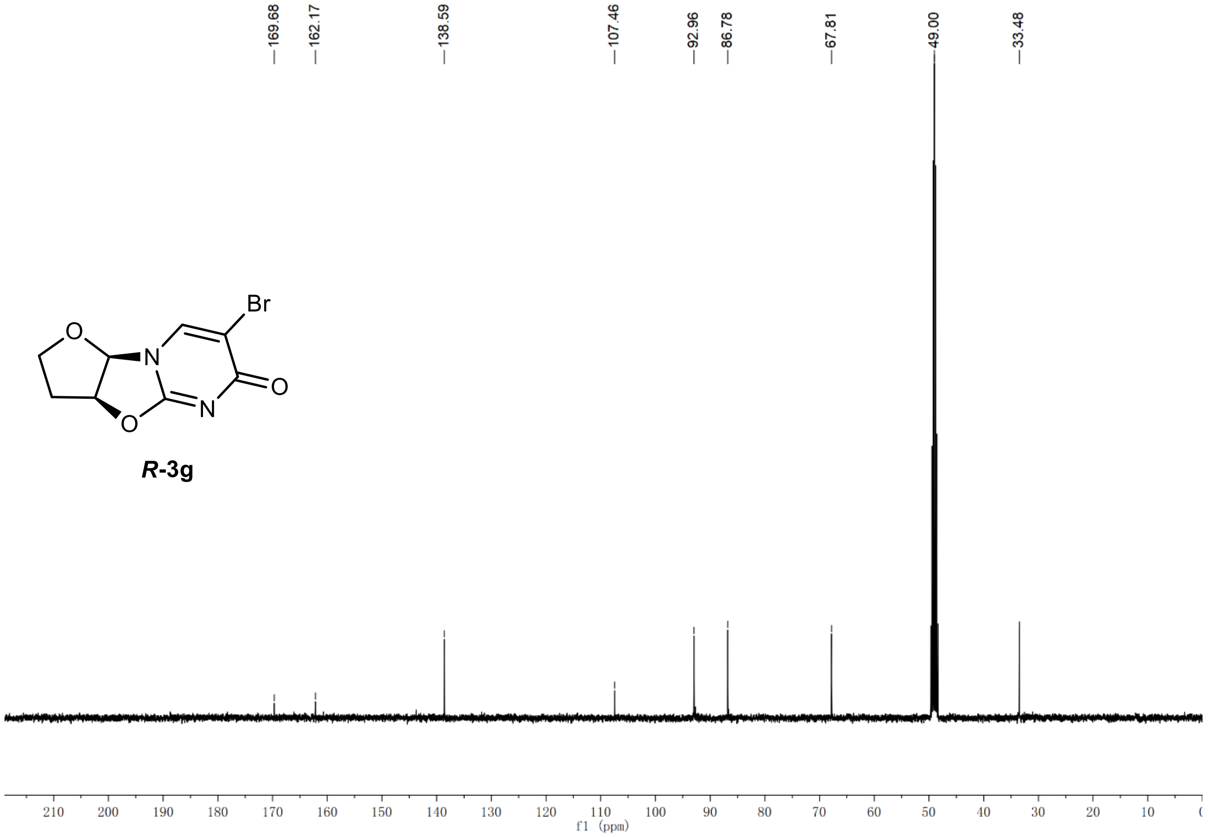

Supplementary Figure 115. ^{13}C NMR (100M, CD_3OD) of compound ***R*-3g.**

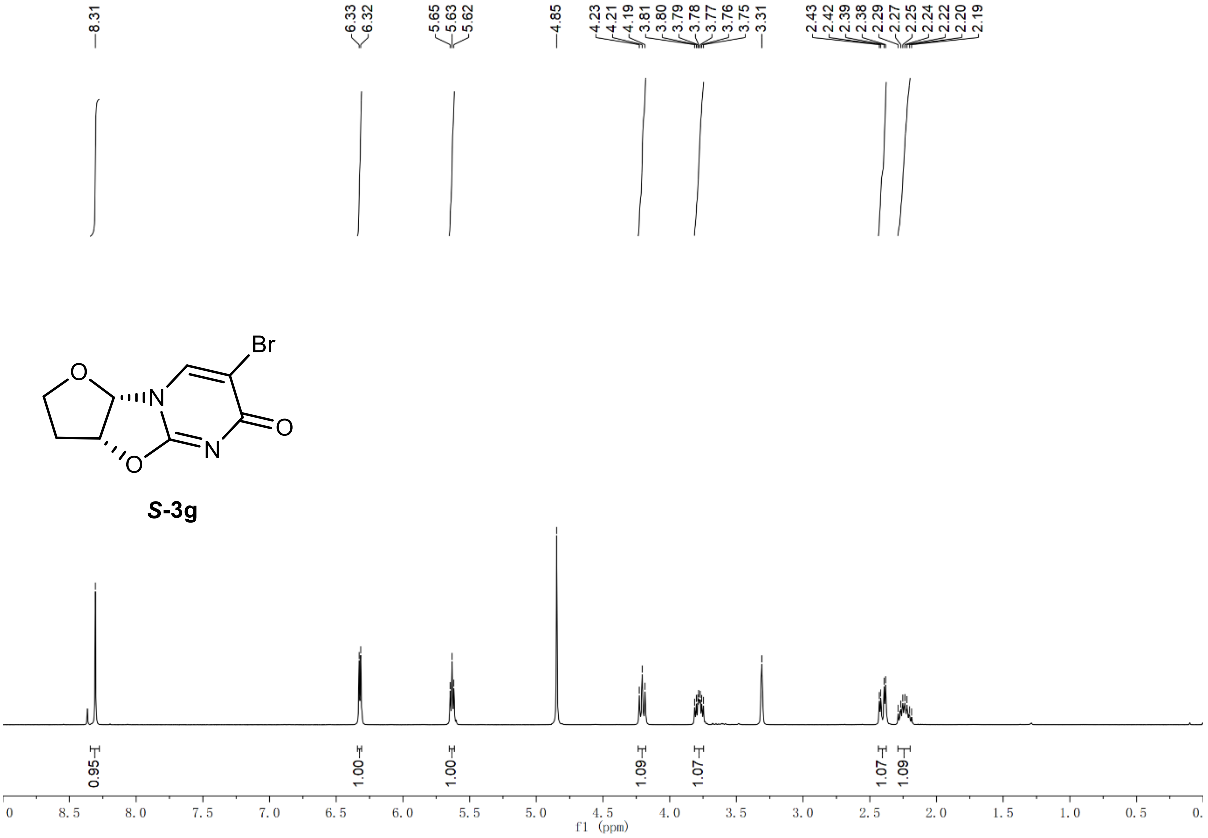

Supplementary Figure 116. ^{1}H NMR (400M, CD_3OD) of compound ***S*-3g.**

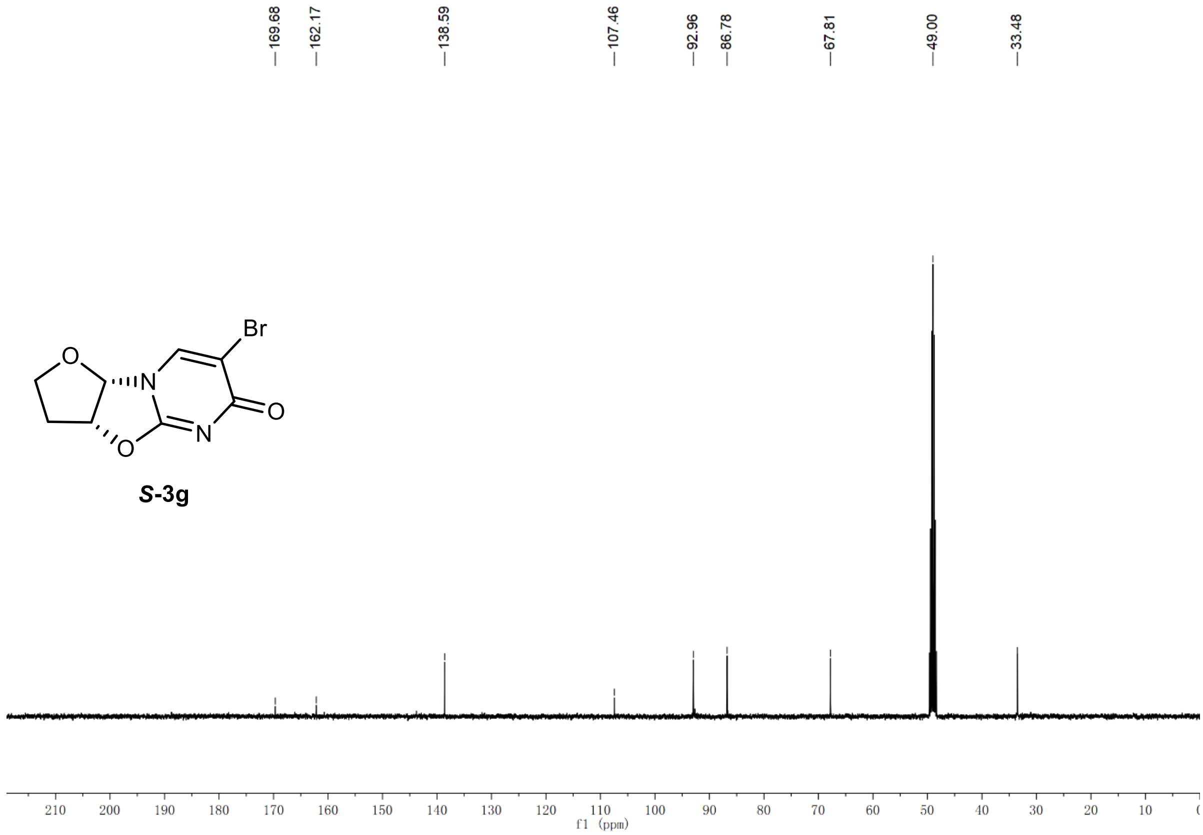

Supplementary Figure 117. ^{13}C NMR (100M, CD_3OD) of compound ***S*-3g.**

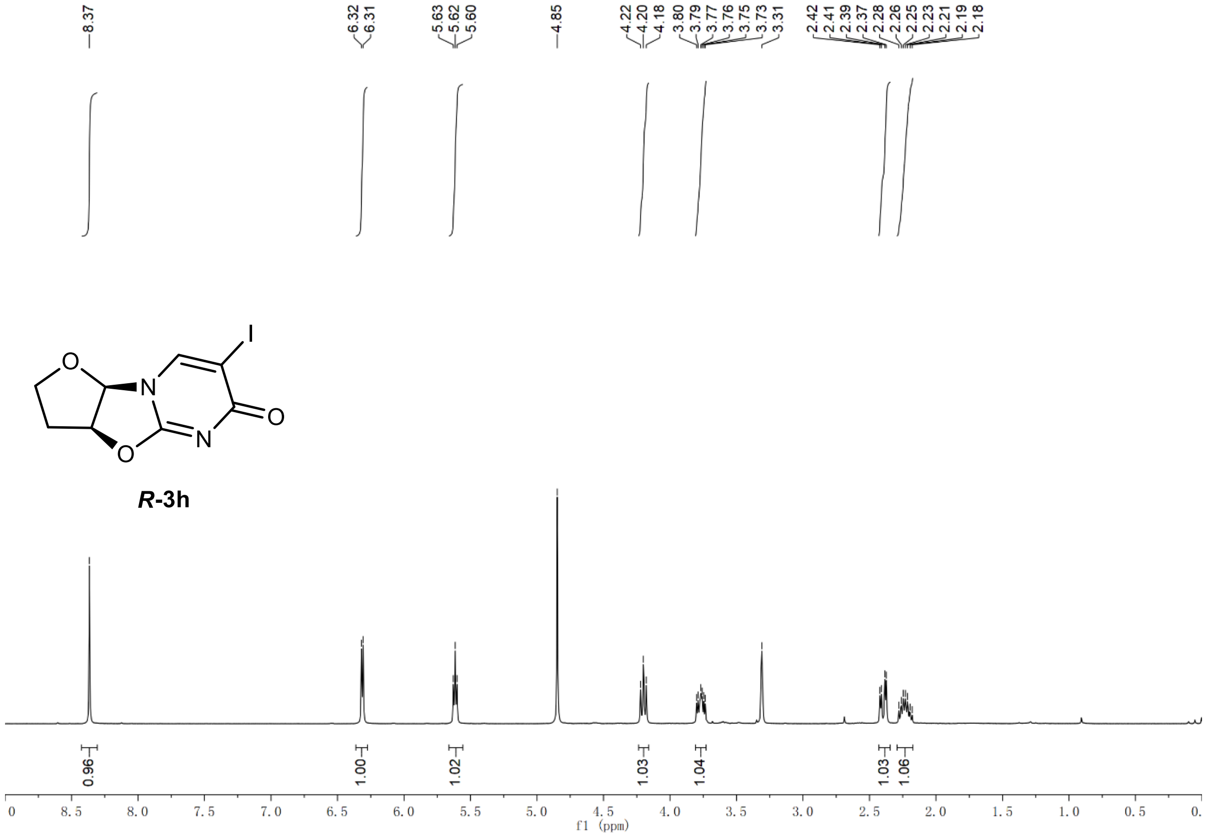

Supplementary Figure 118. ^{1}H NMR (400M, CD_3OD) of compound ***R*-3h.**

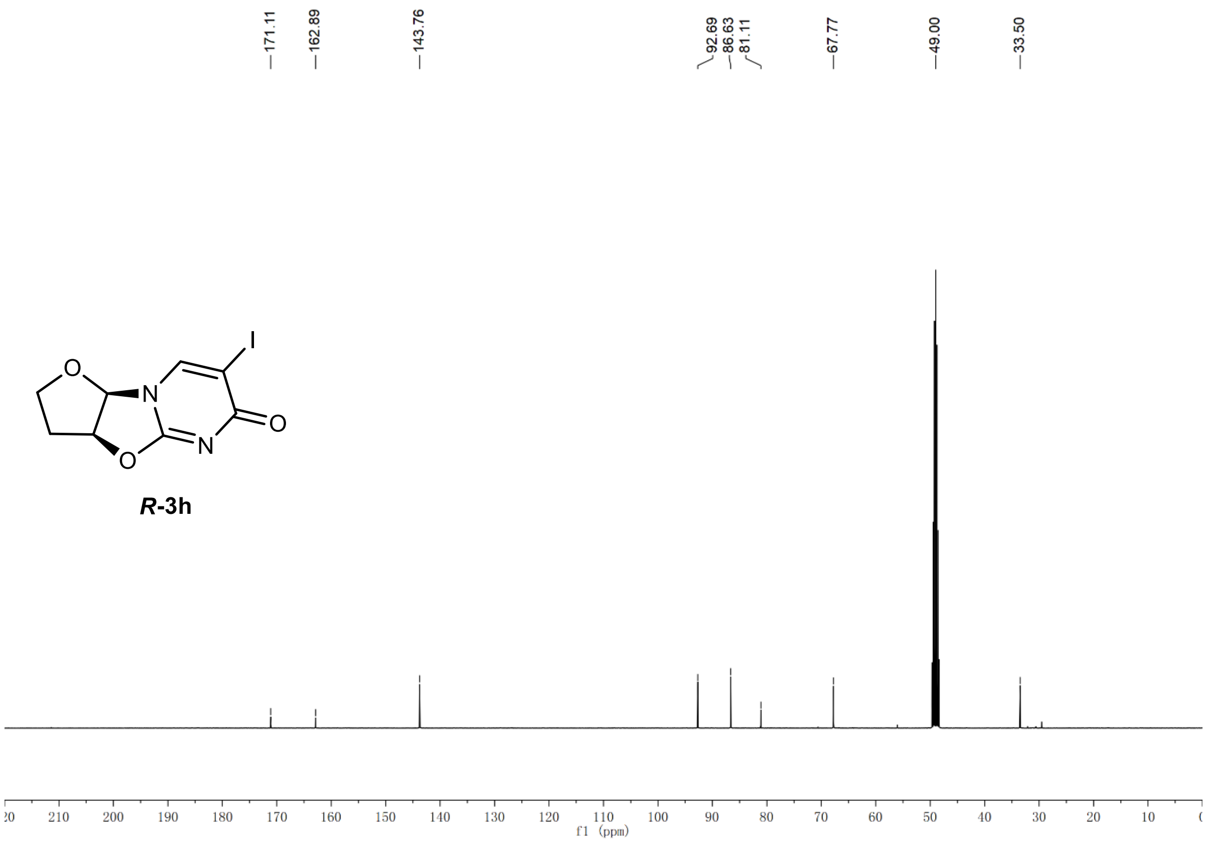

Supplementary Figure 119. ^{13}C NMR (100M, CD_3OD) of compound ***R*-3h.**

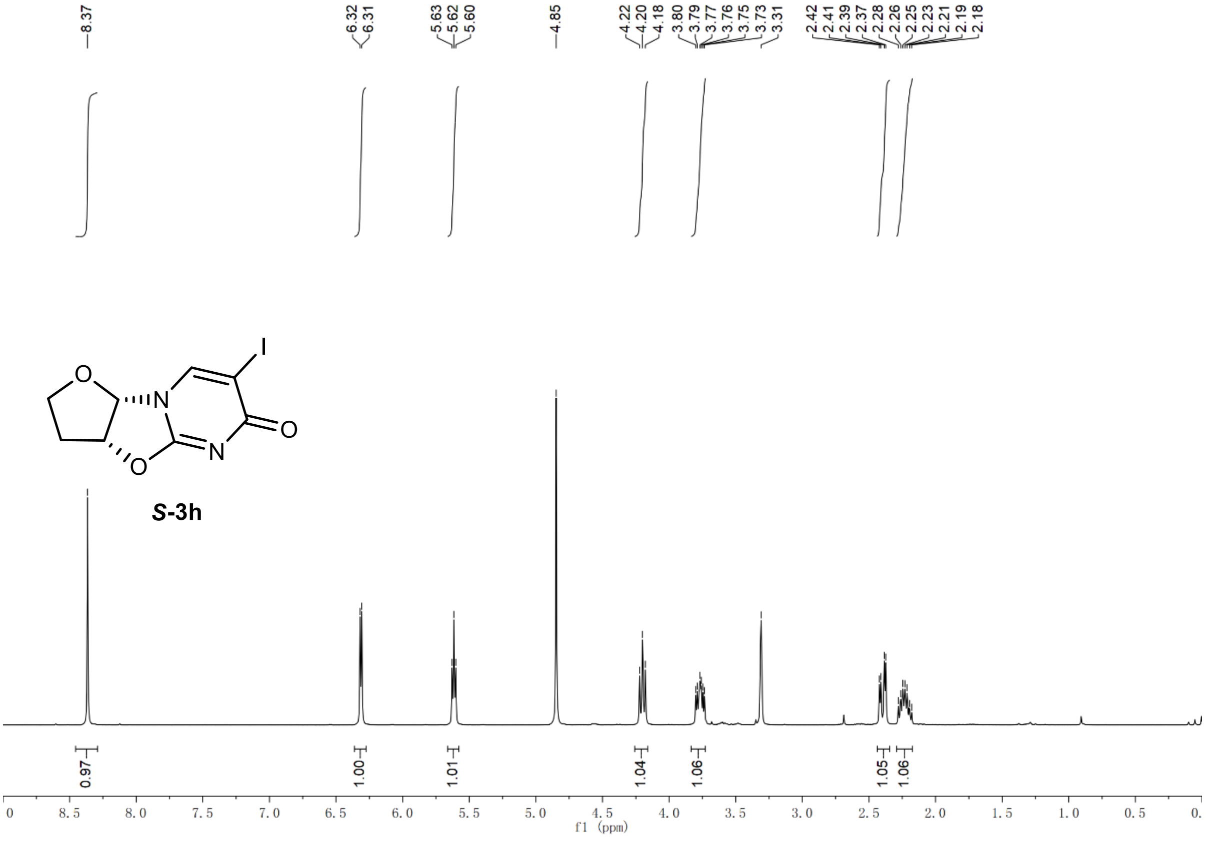

Supplementary Figure 120. ^{1}H NMR (400M, CD_3OD) of compound ***S*-3h.**

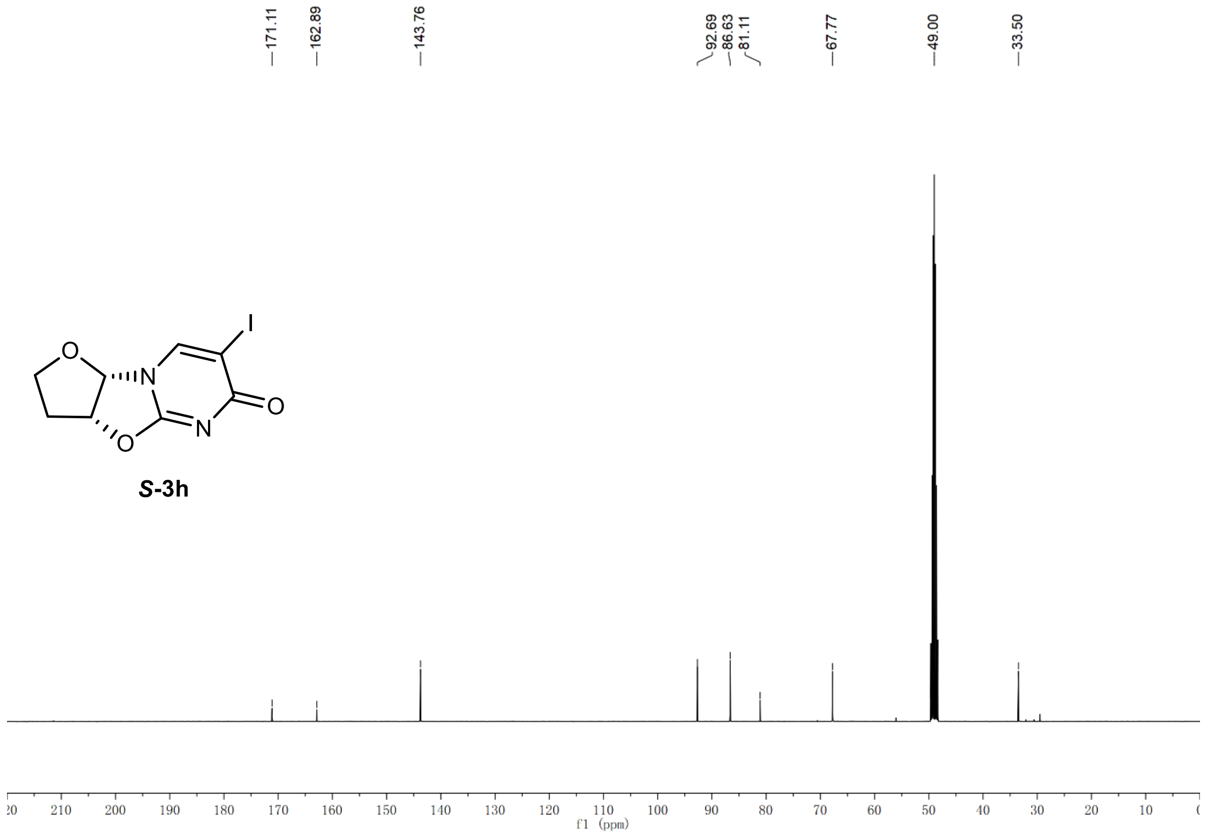

Supplementary Figure 121. ^{13}C NMR (100M, CD_3OD) of compound ***S*-3h.**

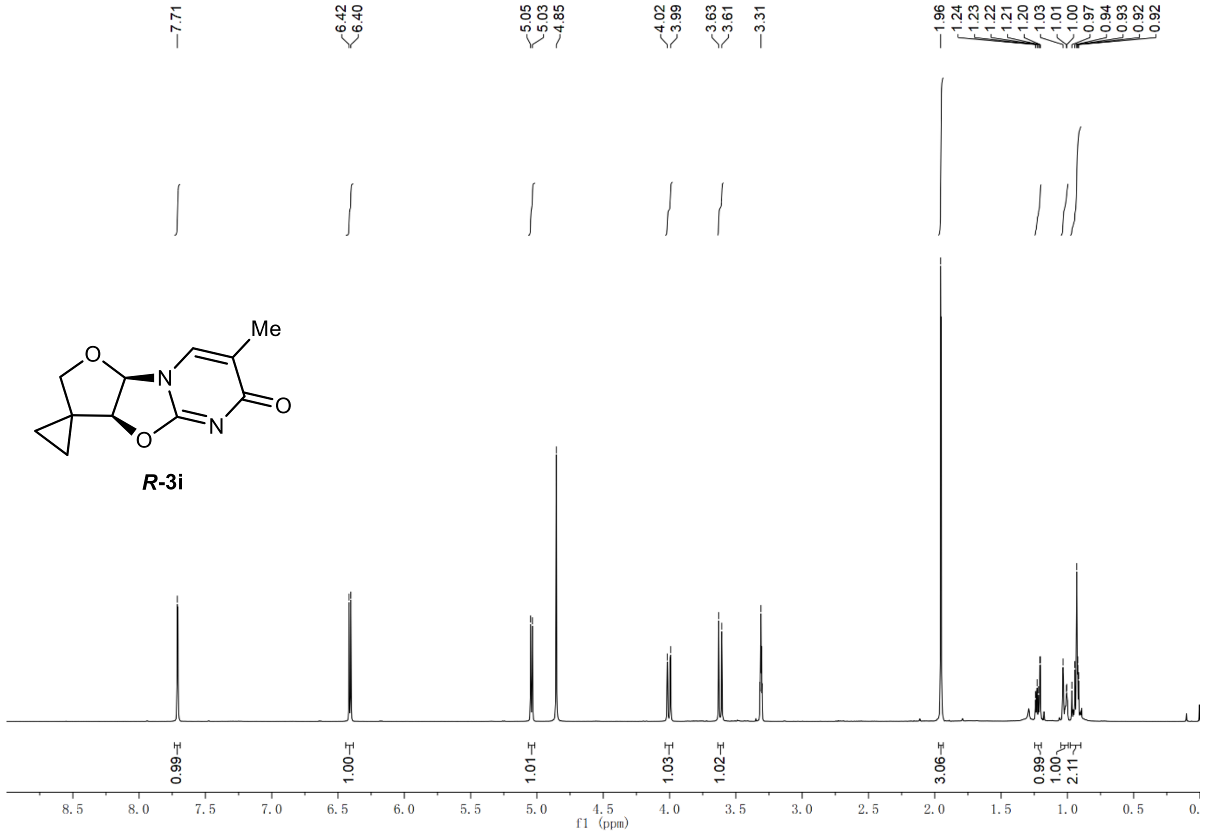

Supplementary Figure 122. ^{1}H NMR (400M, CD_3OD) of compound ***R*-3i.**

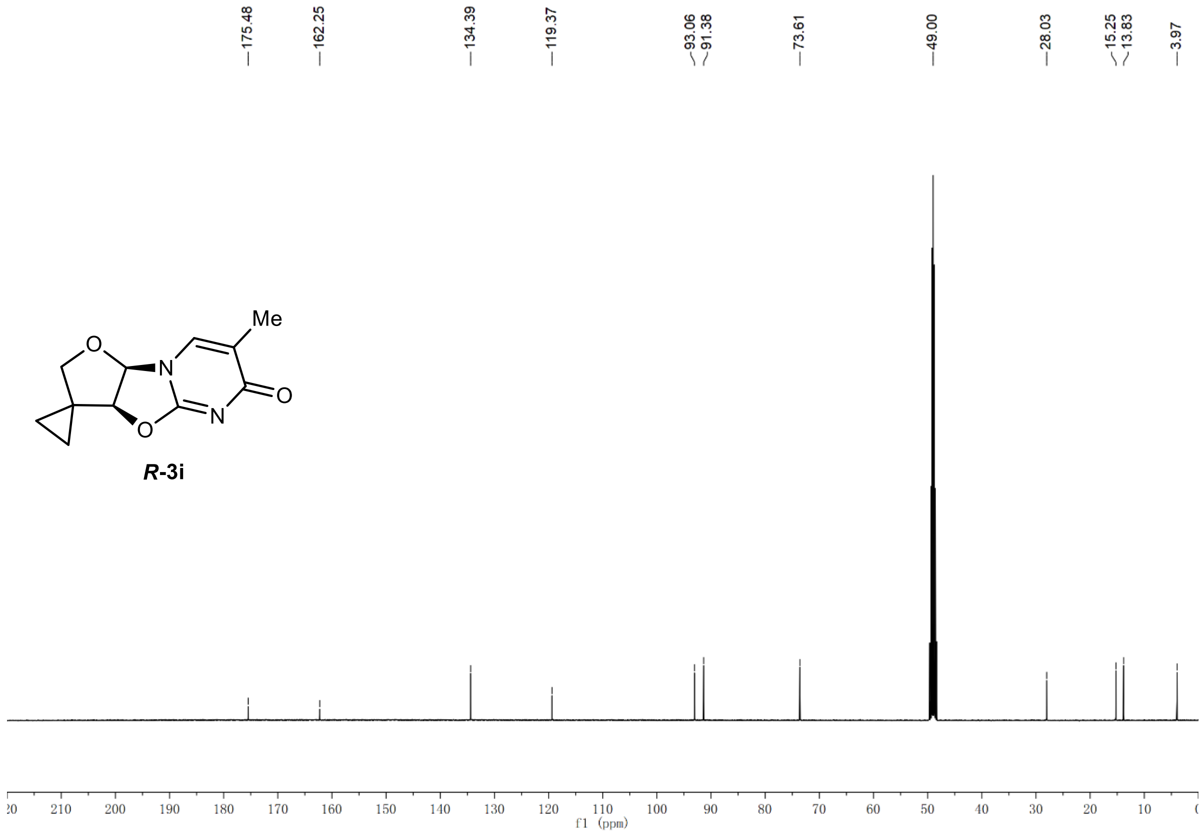

Supplementary Figure 123. ^{13}C NMR (100M, CD_3OD) of compound ***R*-3i.**

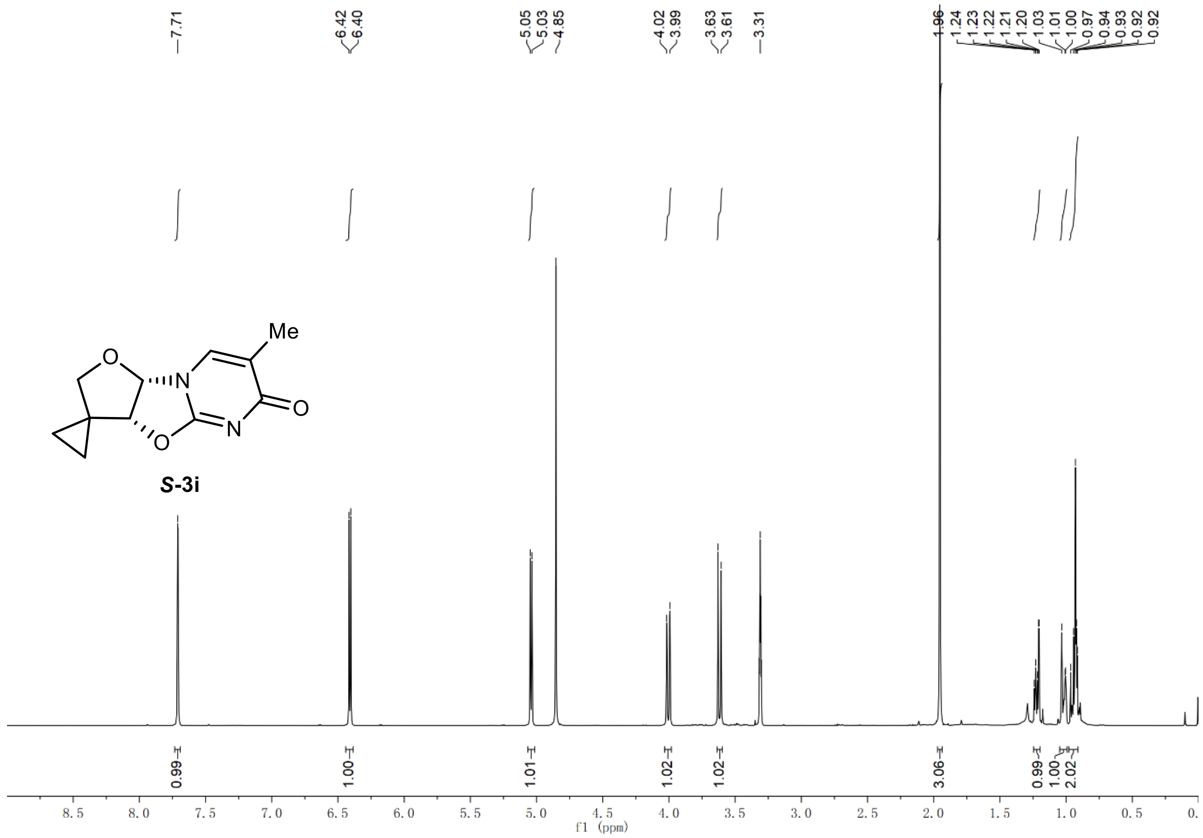

Supplementary Figure 124. ^{1}H NMR (400M, CD_3OD) of compound ***S*-3i.**

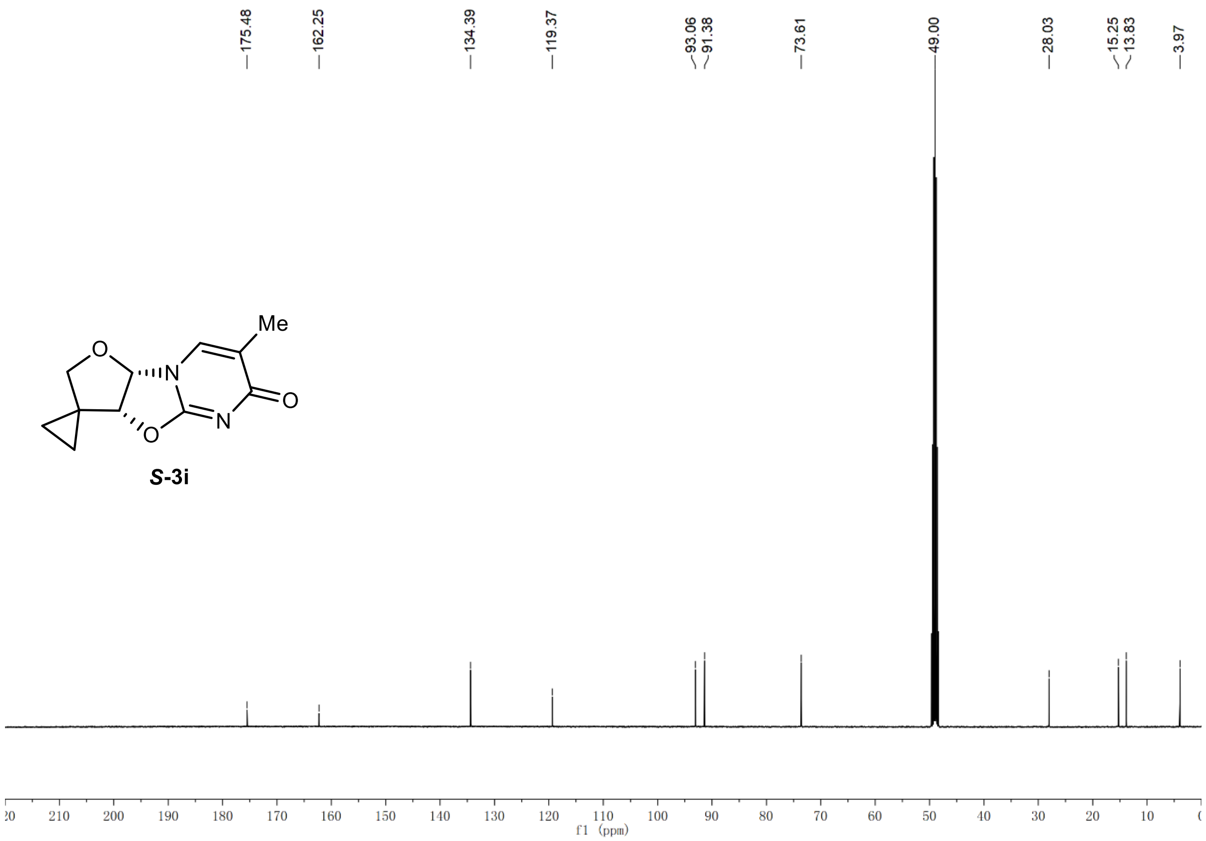

Supplementary Figure 125. ^{13}C NMR (100M, CD_3OD) of compound ***S*-3i.**

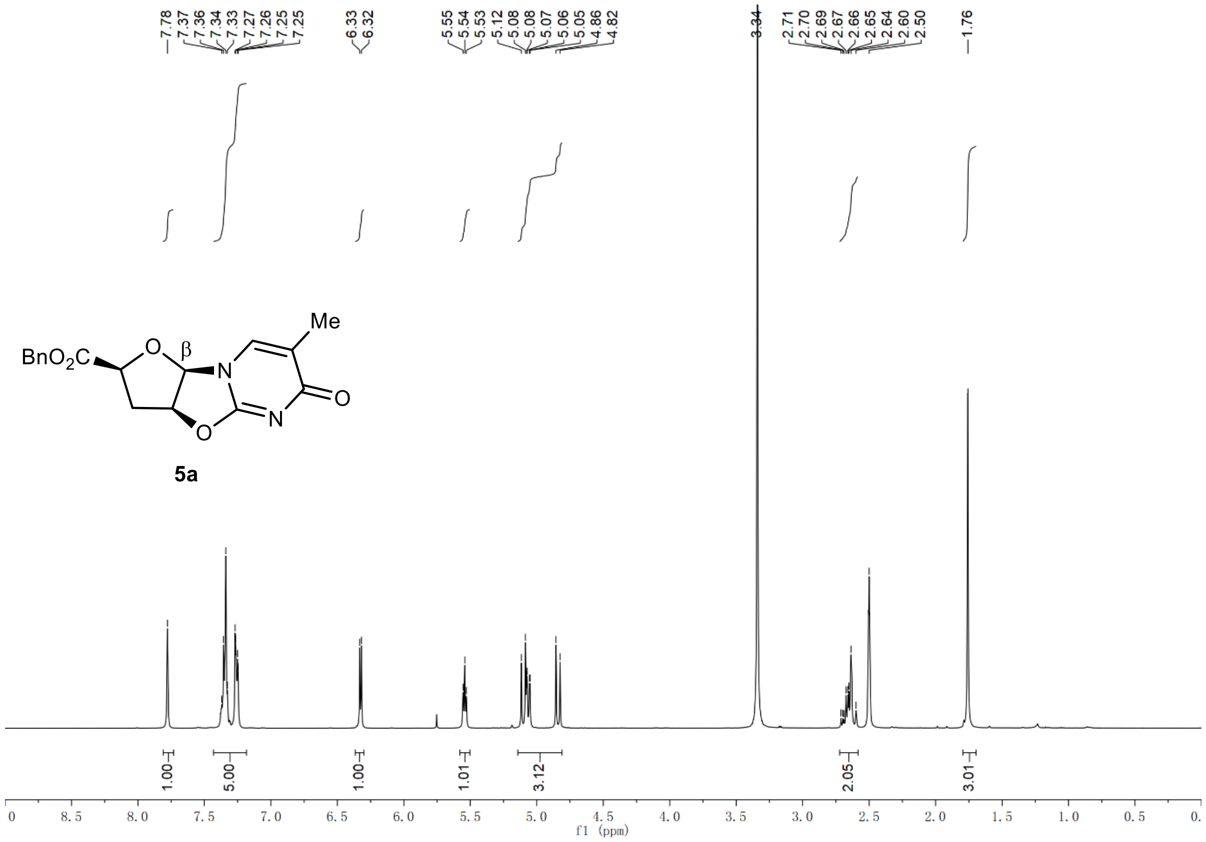

Supplementary Figure 126. ^{1}H NMR (400M, $(CD_3)_2SO$) of compound **5a.**

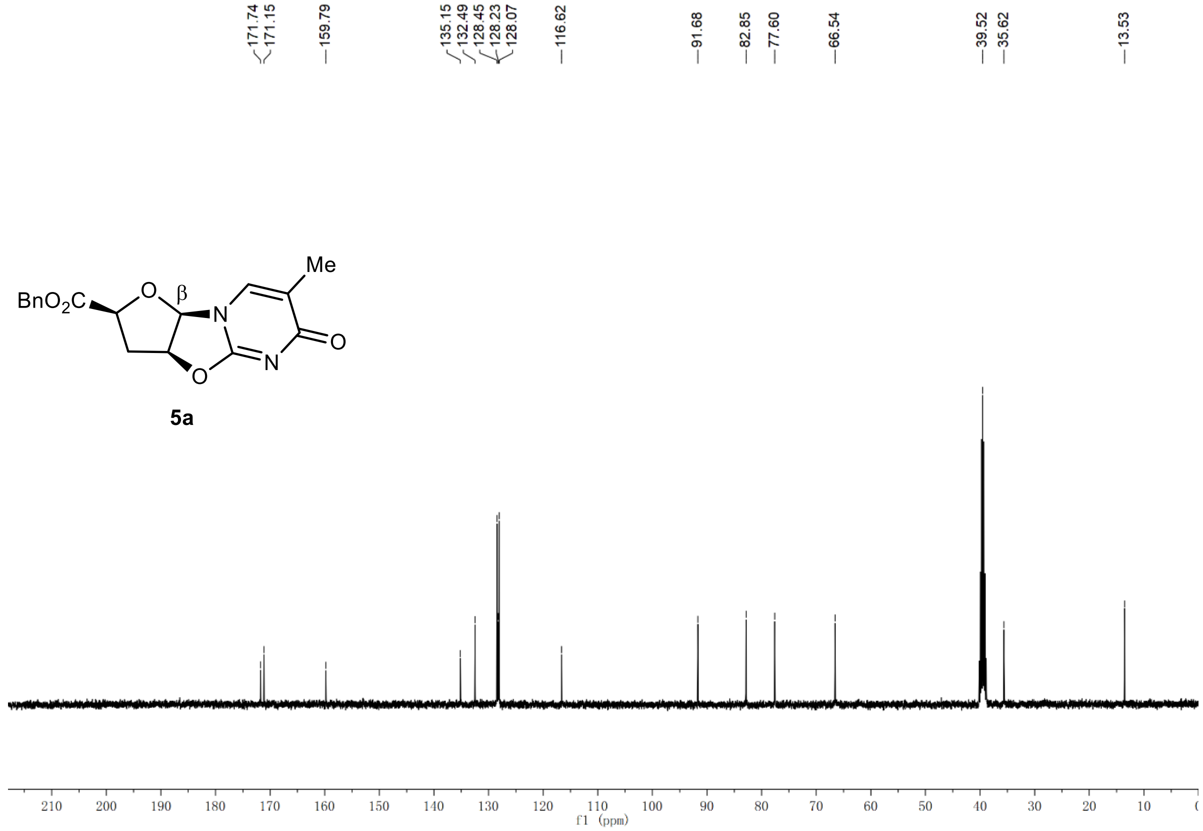

Supplementary Figure 127. ^{13}C NMR (100M, $(CD_3)_2SO$) of compound **5a.**

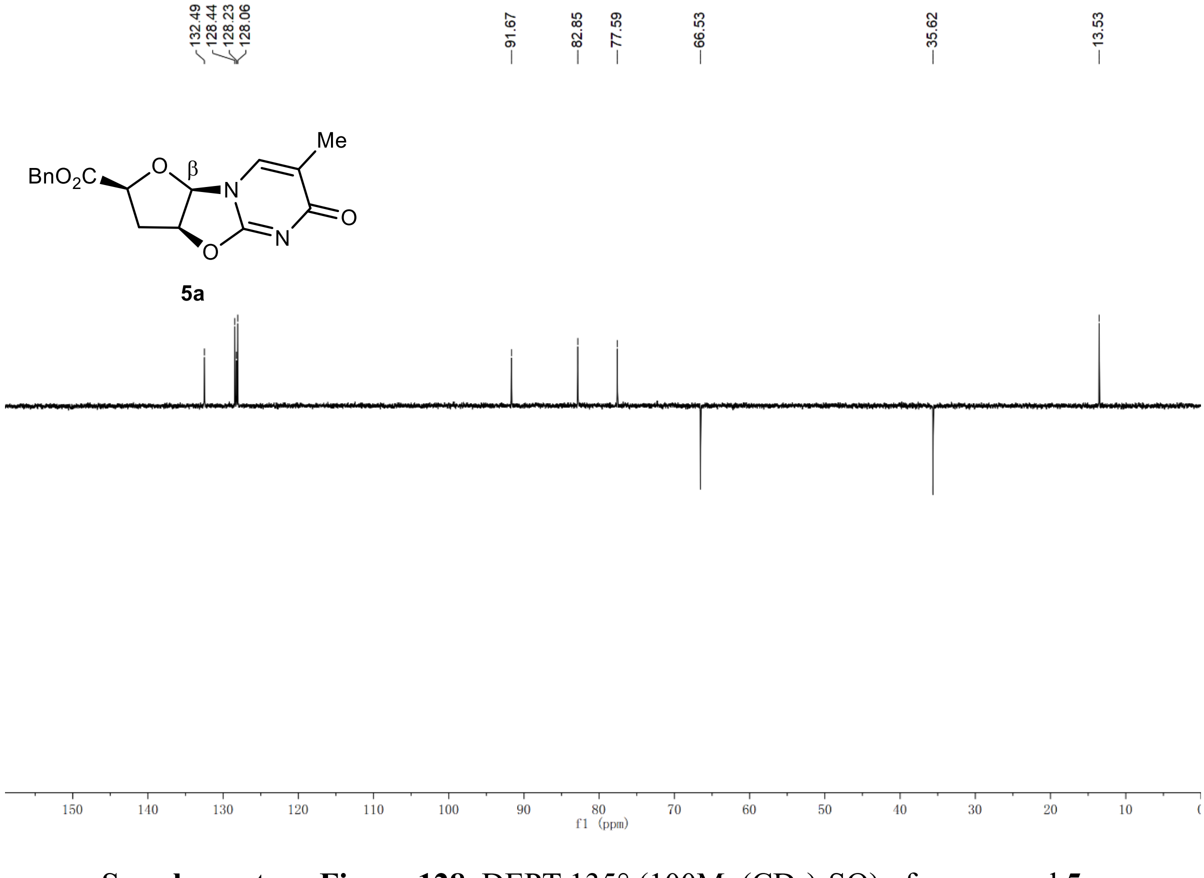

Supplementary Figure 128. DEPT 135° (100M, $(CD_3)_2SO$) of compound **5a.**

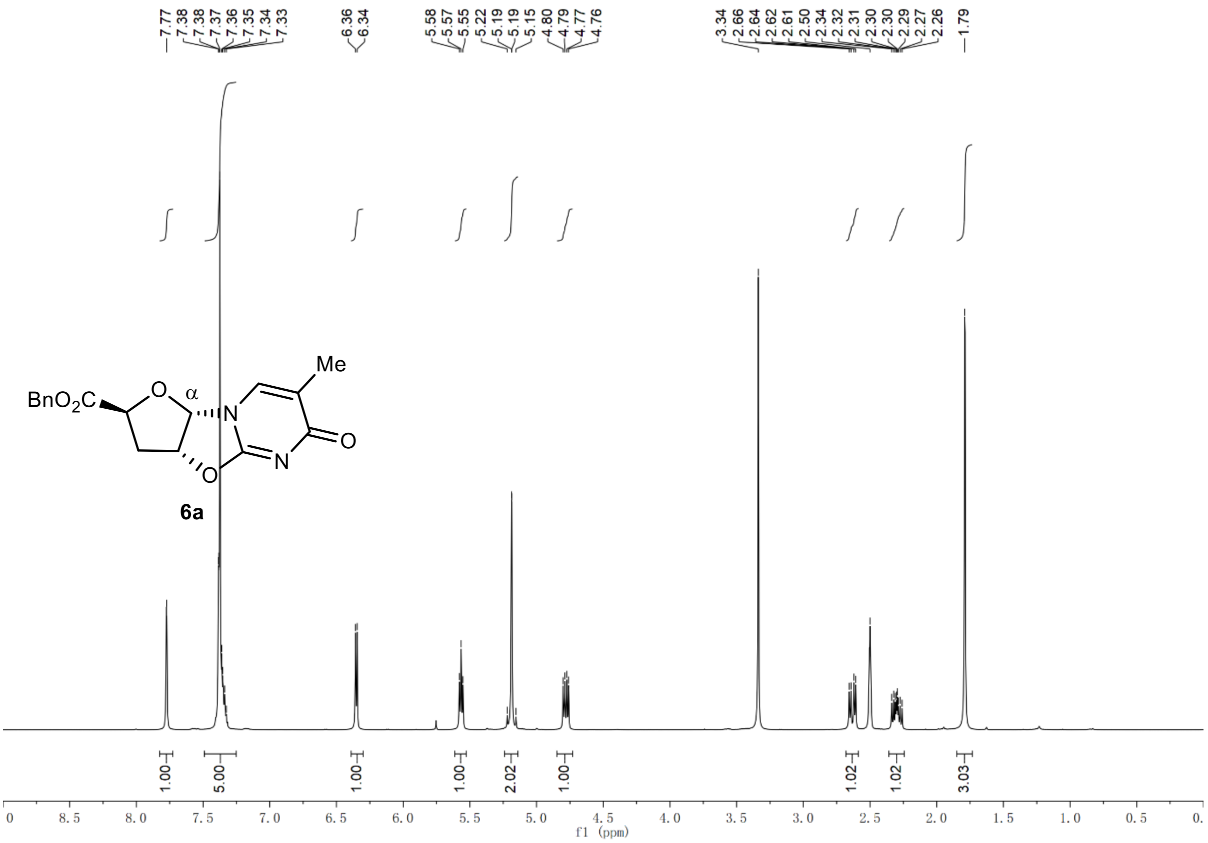

Supplementary Figure 129. ^{1}H NMR (400M, $(CD_3)_2SO$) of compound **6a.**

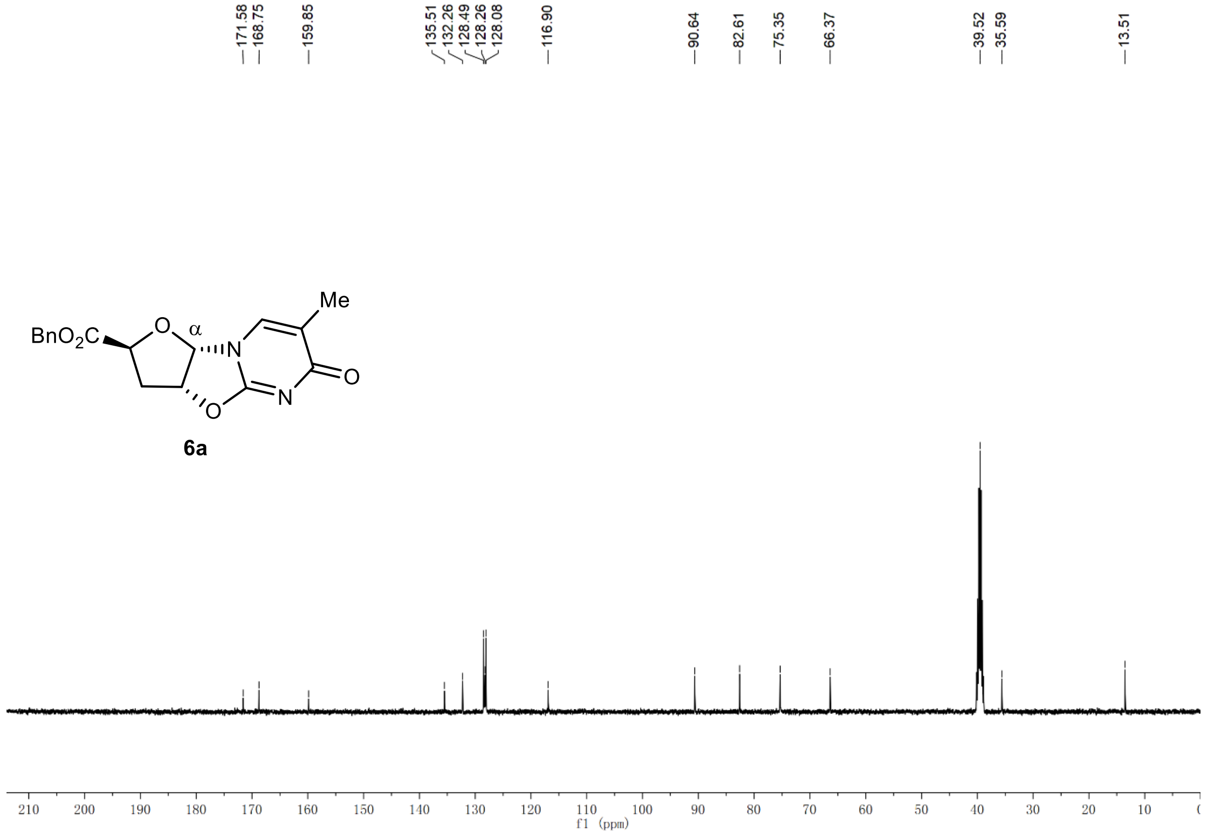

Supplementary Figure 130. ^{13}C NMR (100M, $(CD_3)_2SO$) of compound **6a.**

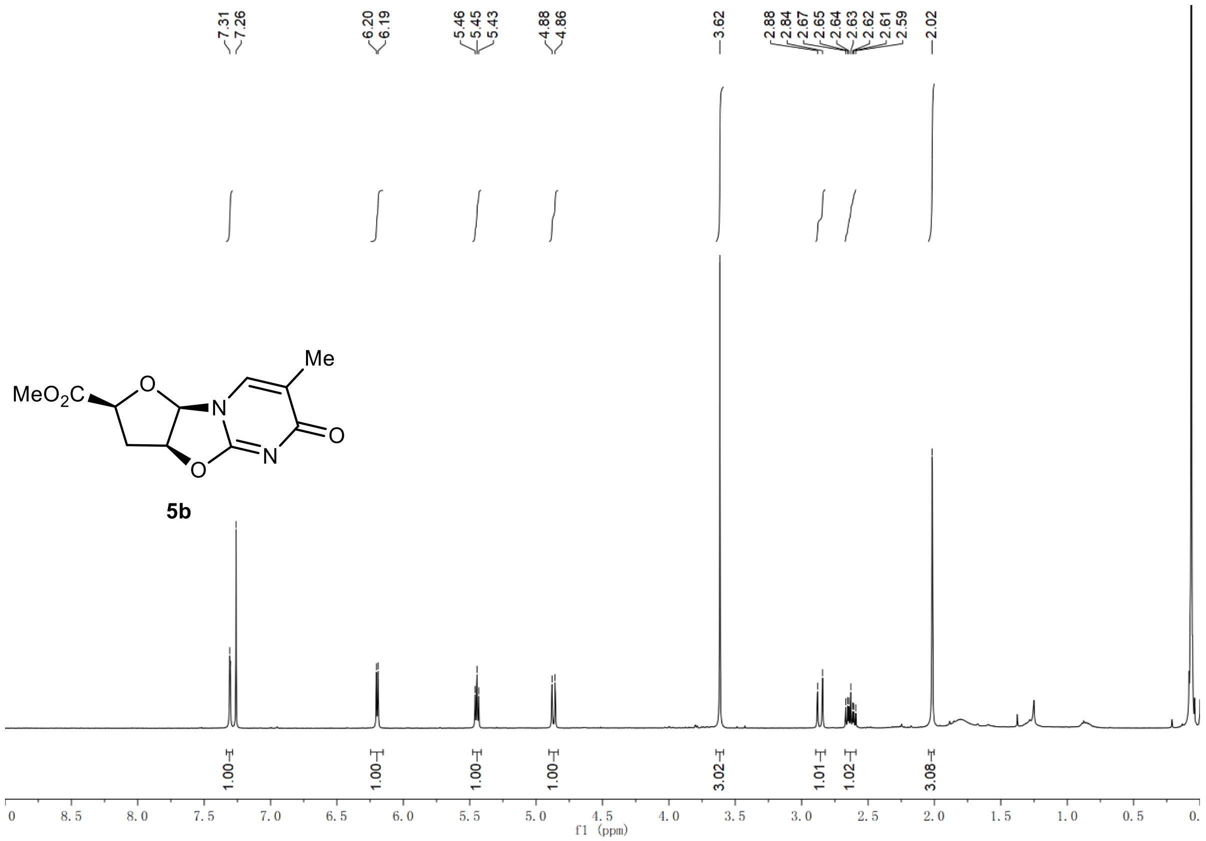

Supplementary Figure 131. ^{1}H NMR (400M, $CDCl_3$) of compound **5b.**

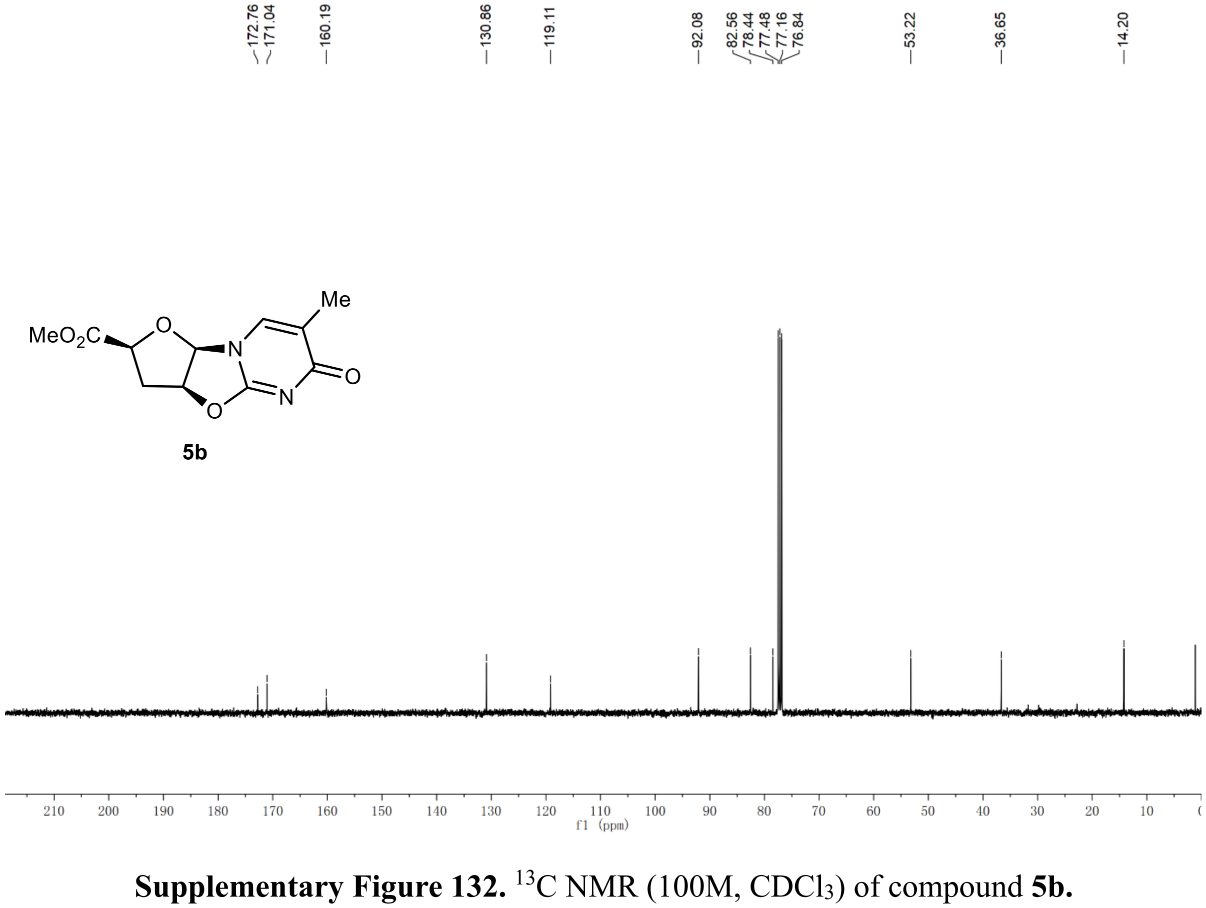

Supplementary Figure 132. ^{13}C NMR (100M, $CDCl_3$) of compound **5b.**

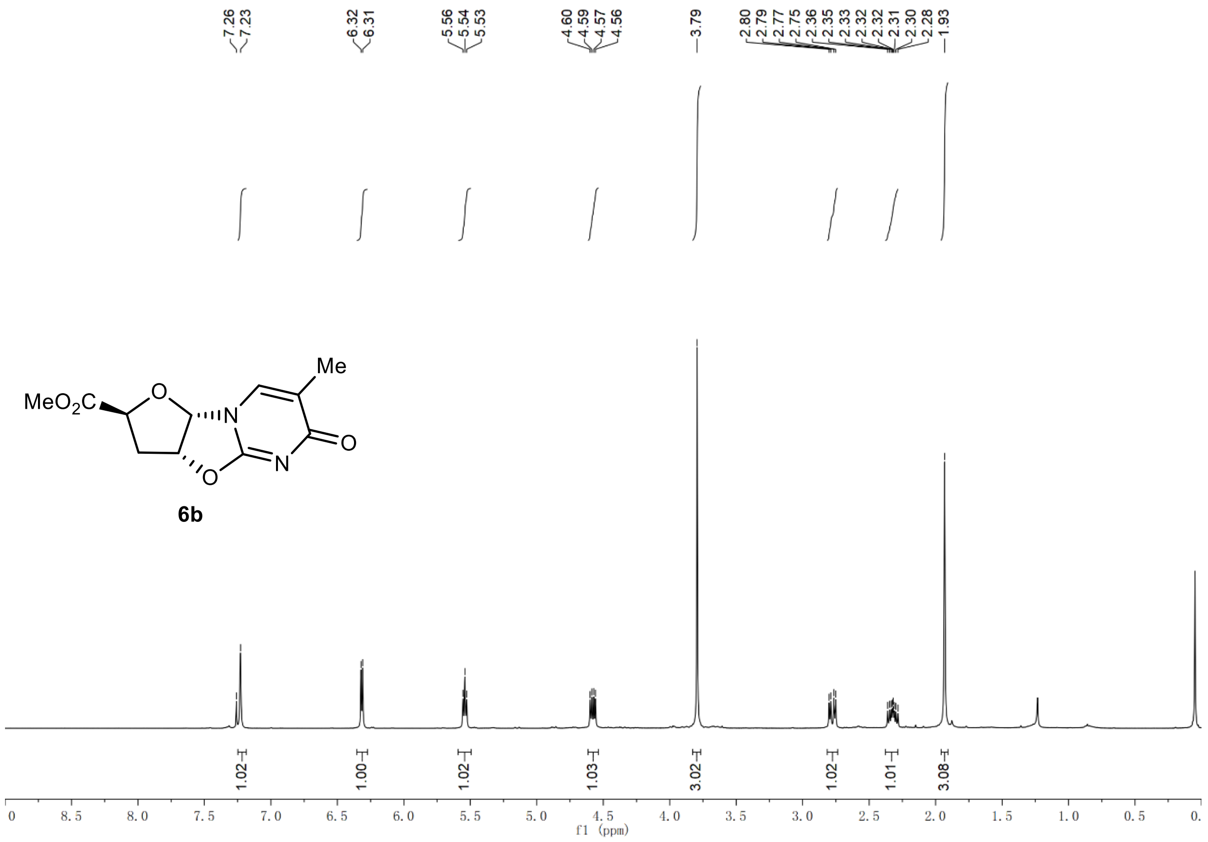

Supplementary Figure 133. ^{1}H NMR (400M, $CDCl_3$) of compound **6b.**

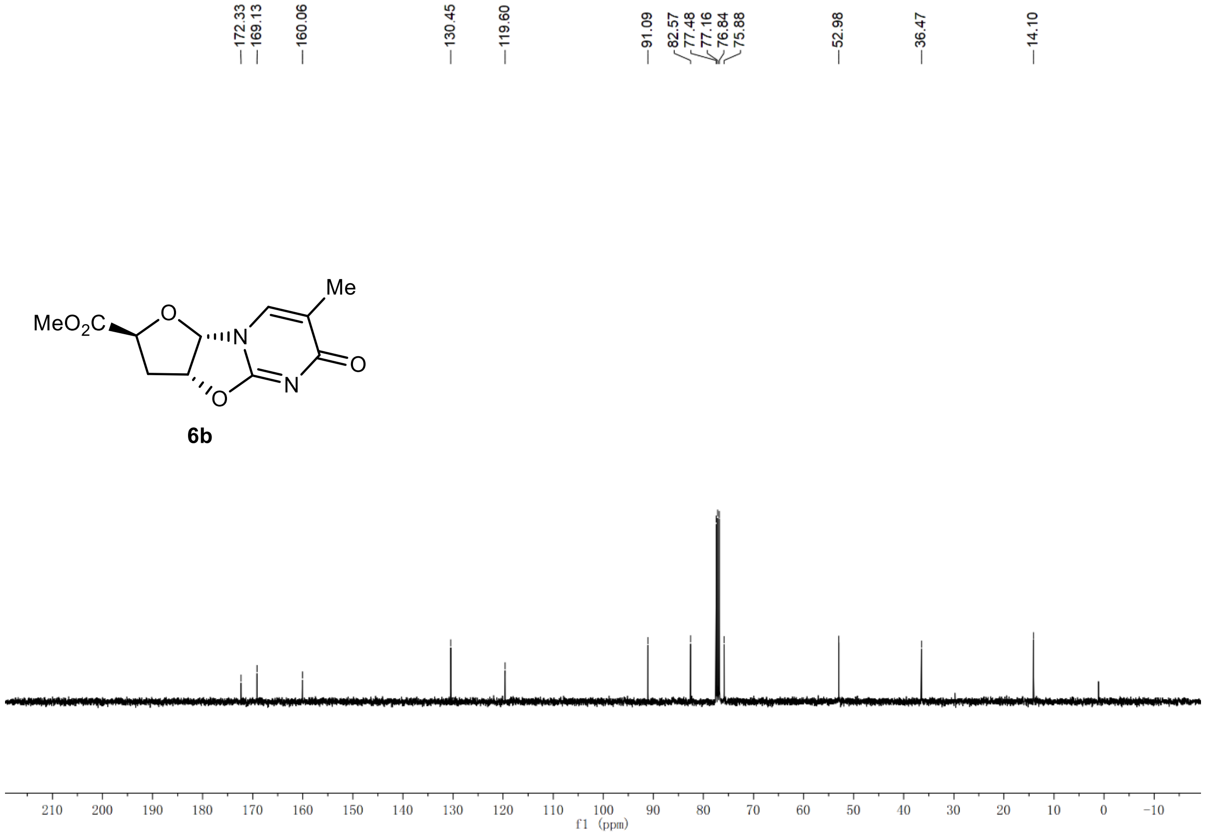

Supplementary Figure 134. ^{13}C NMR (100M, $CDCl_3$) of compound **6b.**

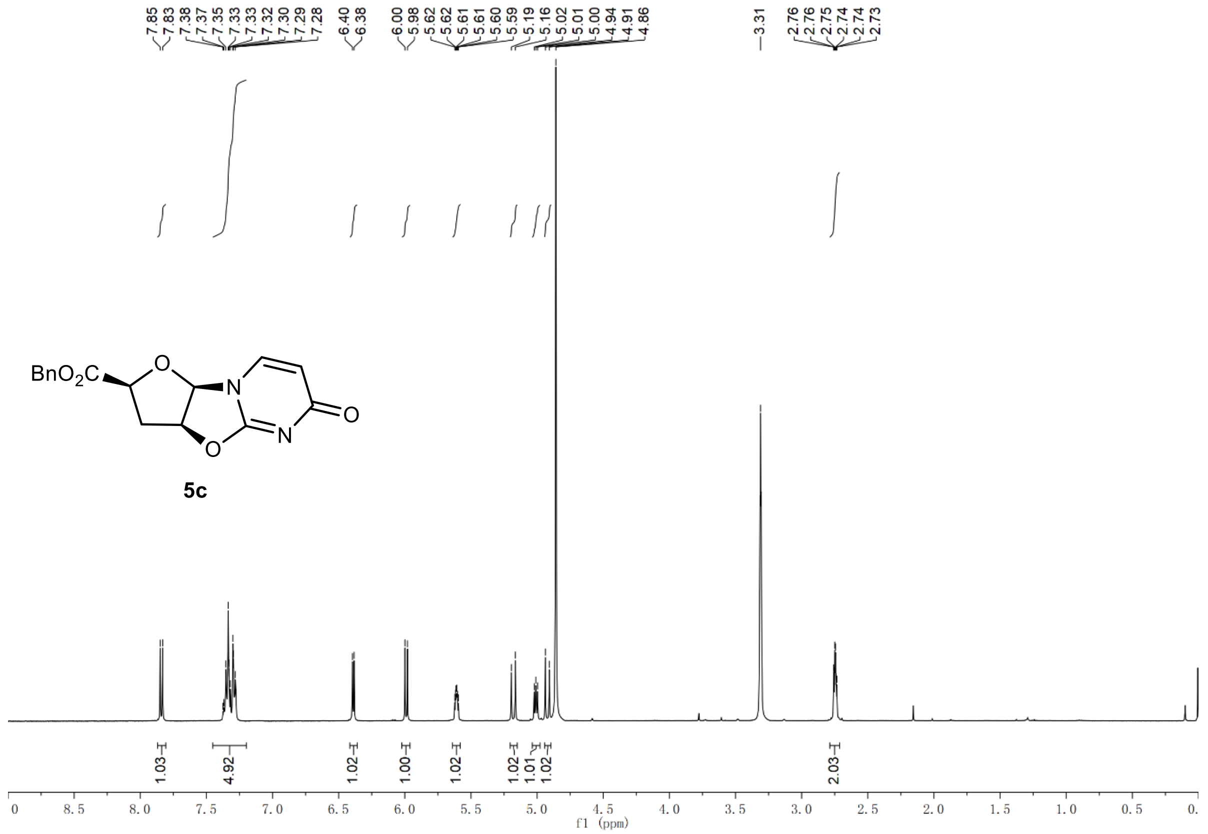

Supplementary Figure 135. ^{1}H NMR (400M, CD_3OD) of compound **5c.**

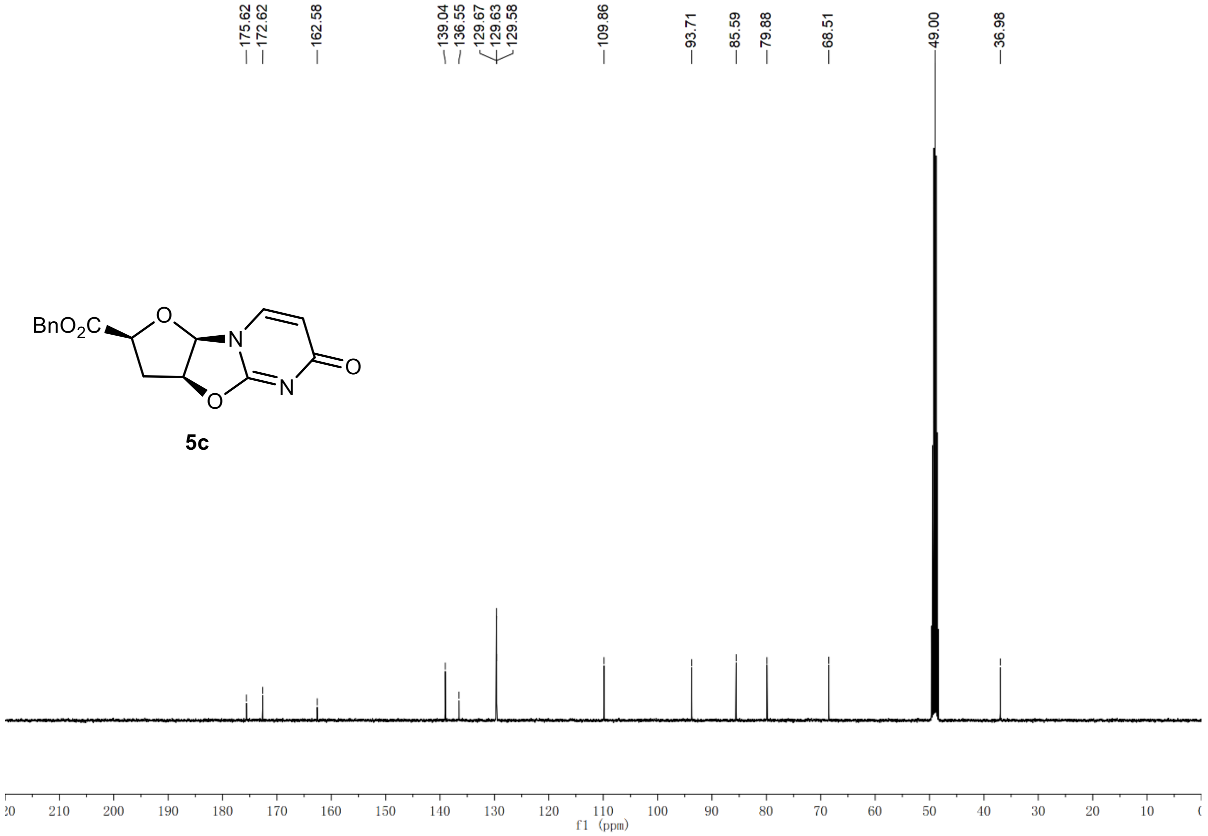

Supplementary Figure 136. ^{13}C NMR (100M, CD_3OD) of compound **5c.**

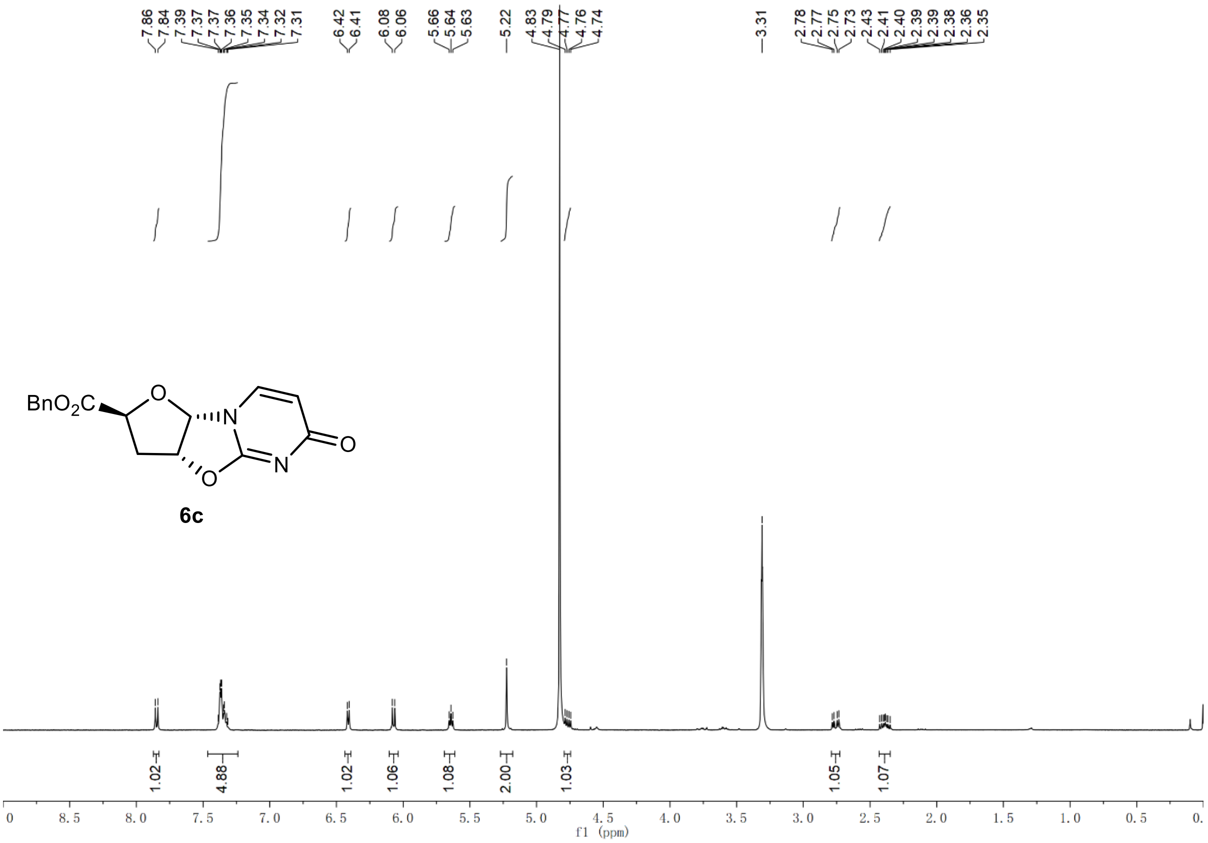

Supplementary Figure 137. ^{1}H NMR (400M, CD_3OD) of compound **6c.**

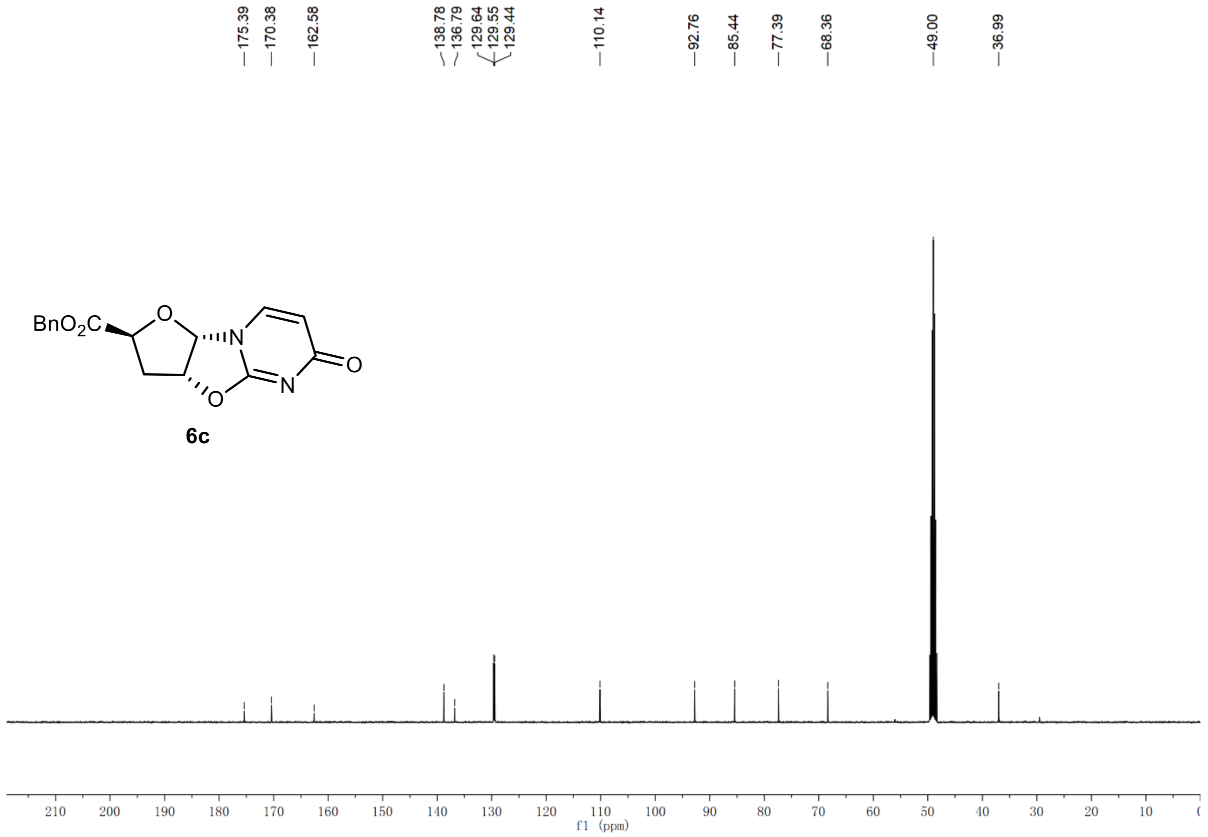

Supplementary Figure 138. ^{13}C NMR (100M, CD_3OD) of compound **6c.**

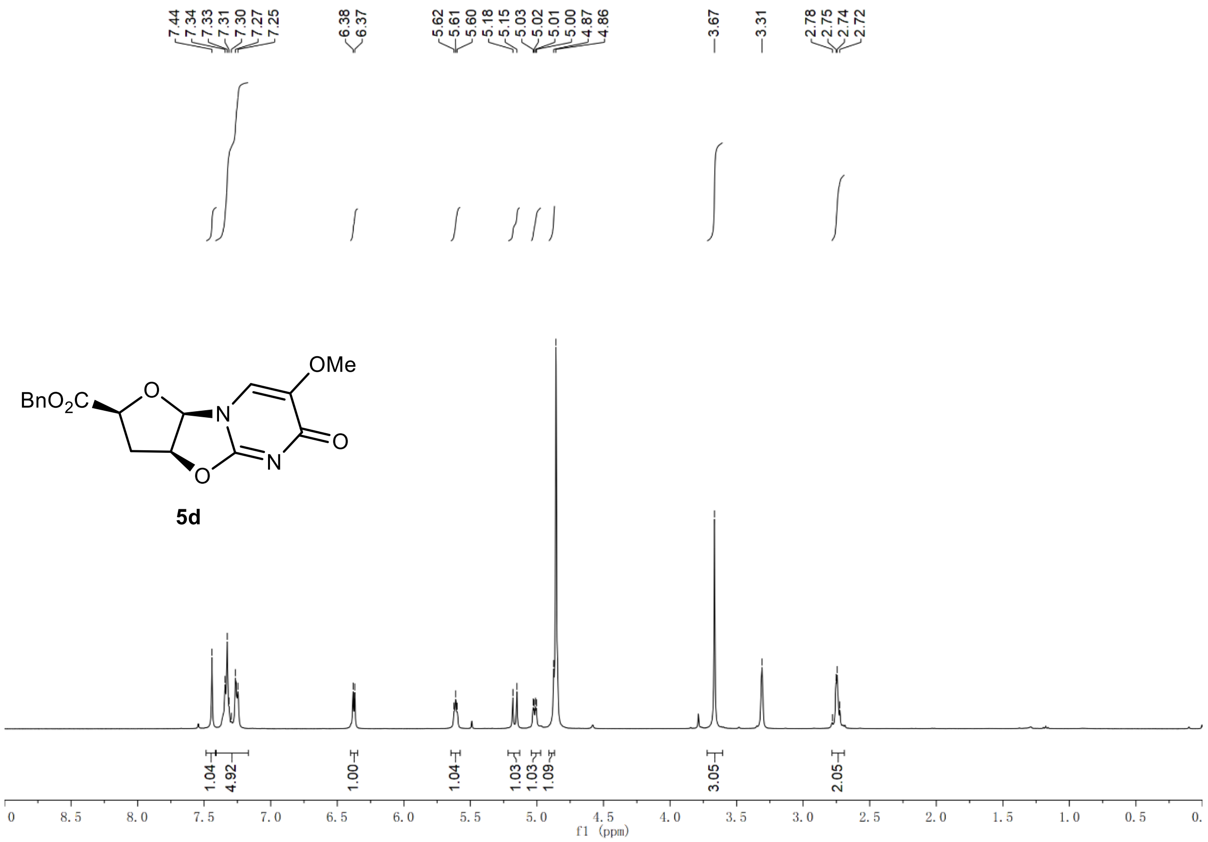

Supplementary Figure 139. ^{1}H NMR (400M, CD_3OD) of compound **5d.**

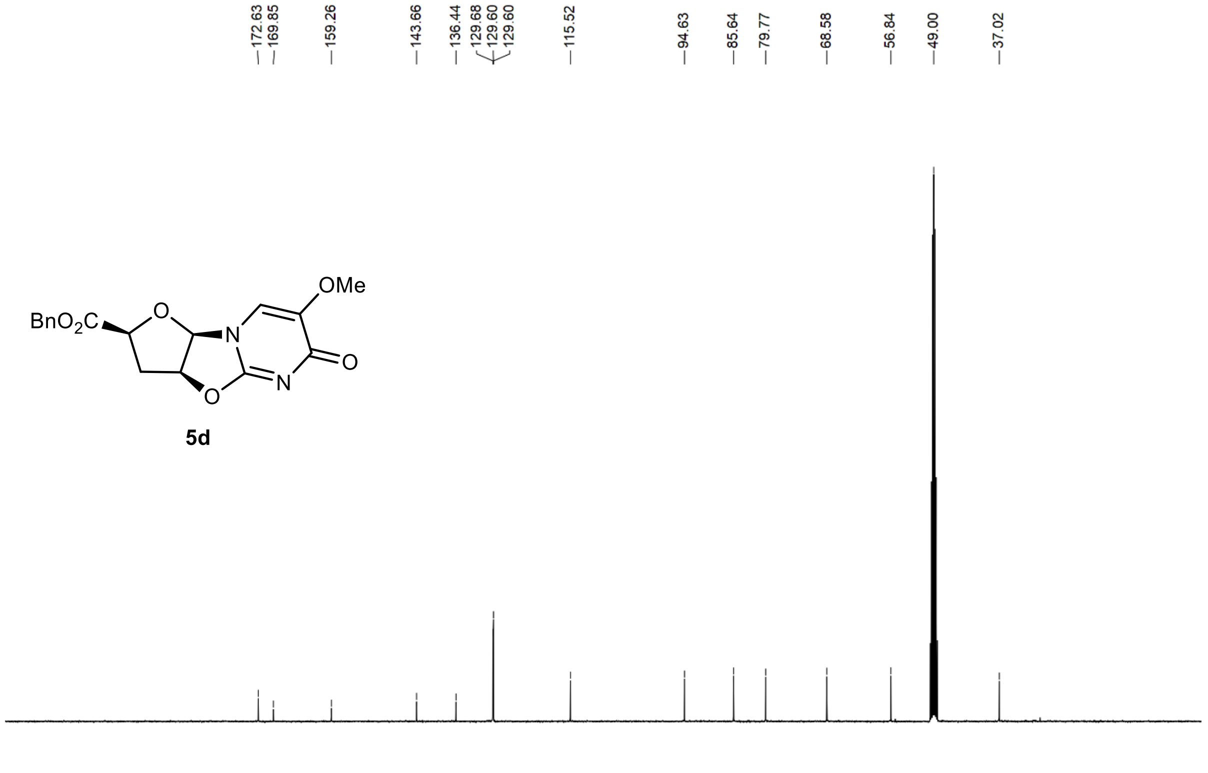

Supplementary Figure 140. ^{13}C NMR (100M, CD_3OD) of compound **5d.**

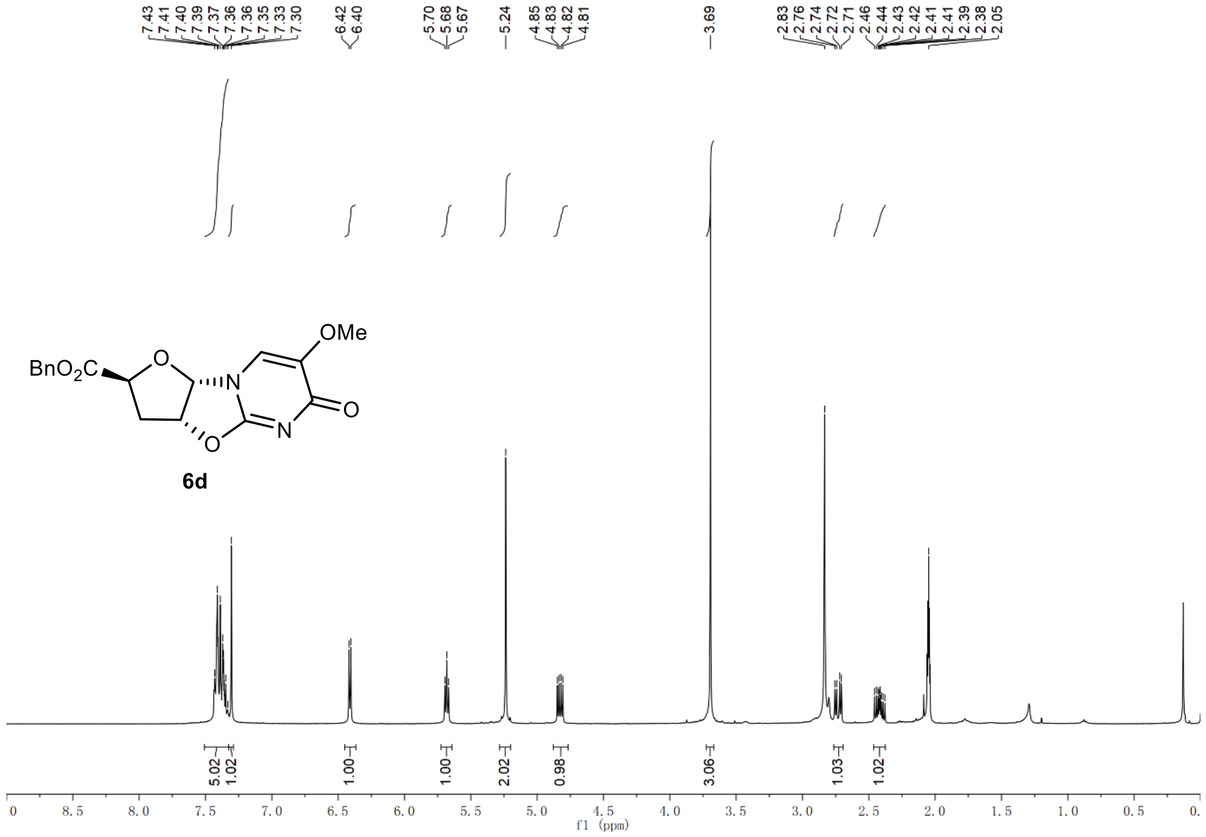

Supplementary Figure 141. ^{1}H NMR (400M, $(CD_3)_2CO$) of compound **6d.**

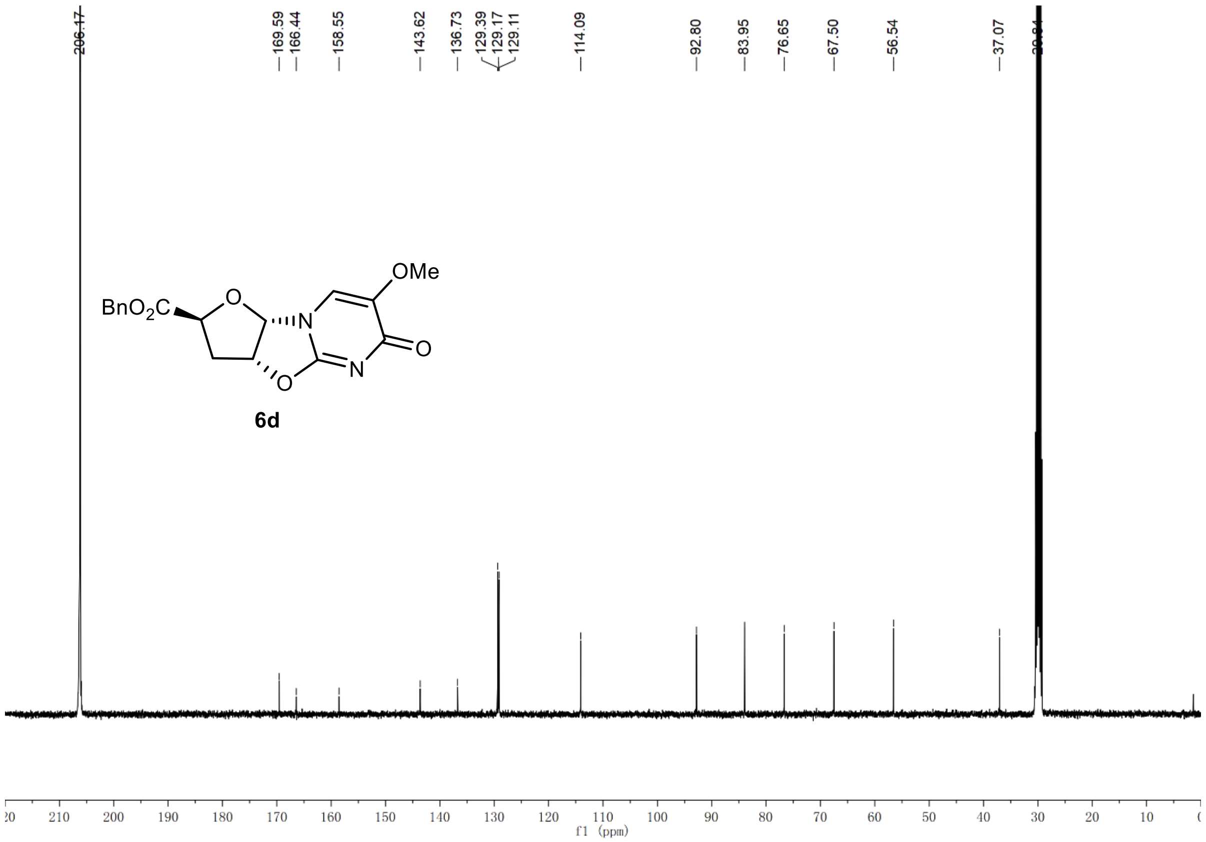

Supplementary Figure 142. ^{13}C NMR (100M, $(CD_3)_2CO$) of compound **6d.**

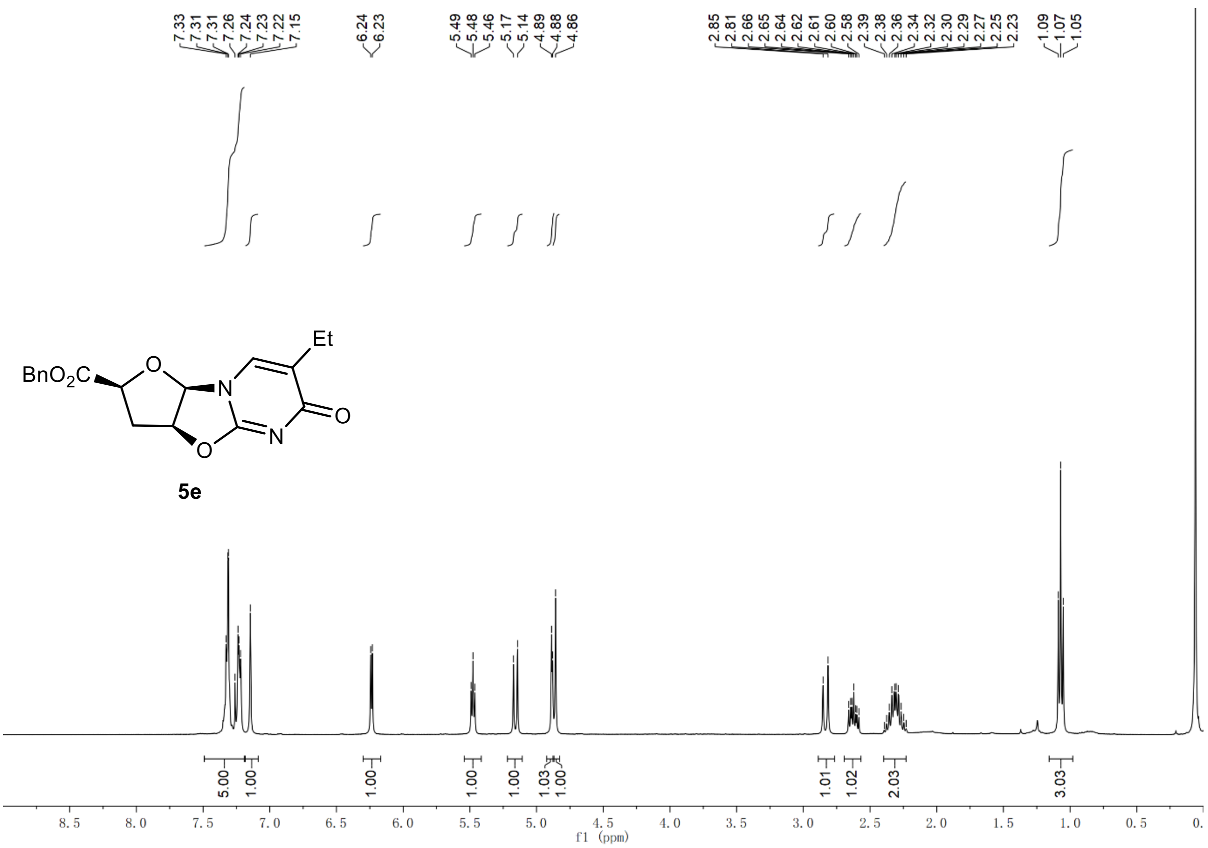

Supplementary Figure 143. ^{1}H NMR (400M, $CDCl_3$) of compound **5e.**

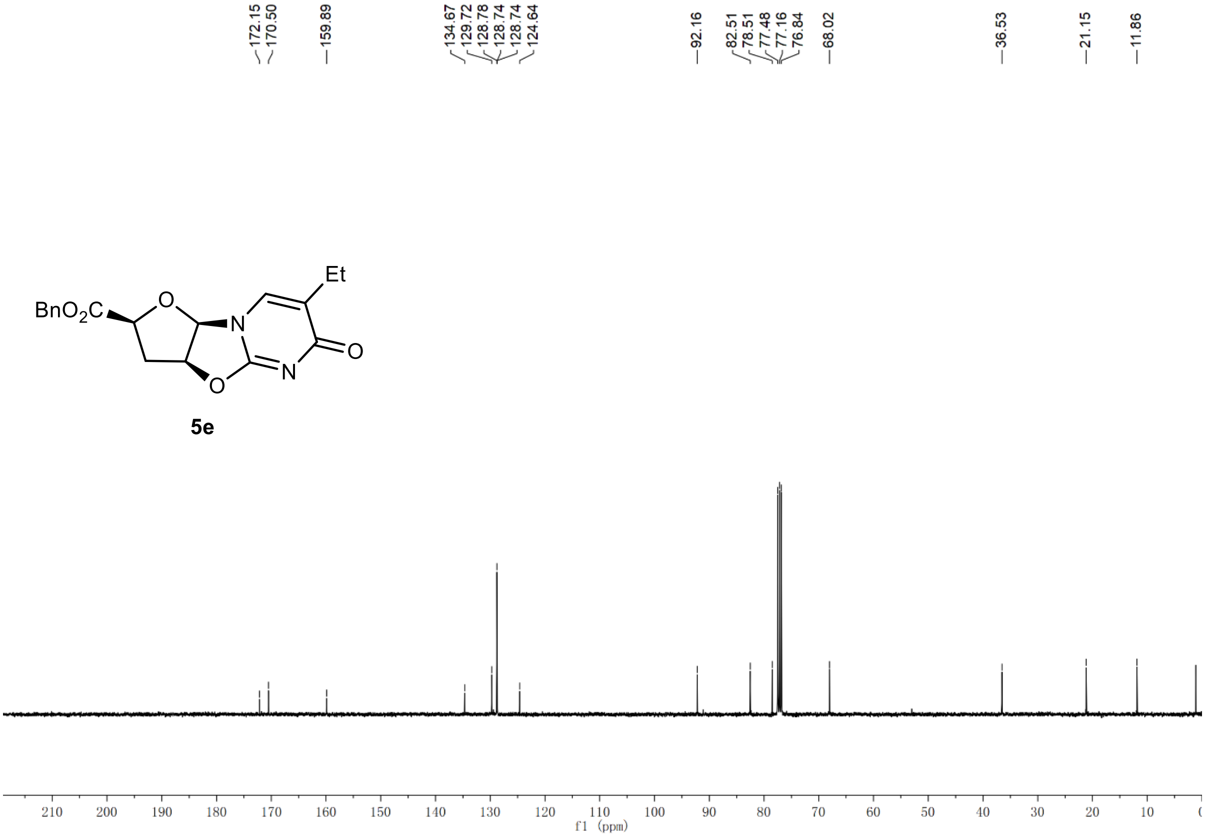

Supplementary Figure 144. ^{13}C NMR (100M, $CDCl_3$) of compound **5e.**

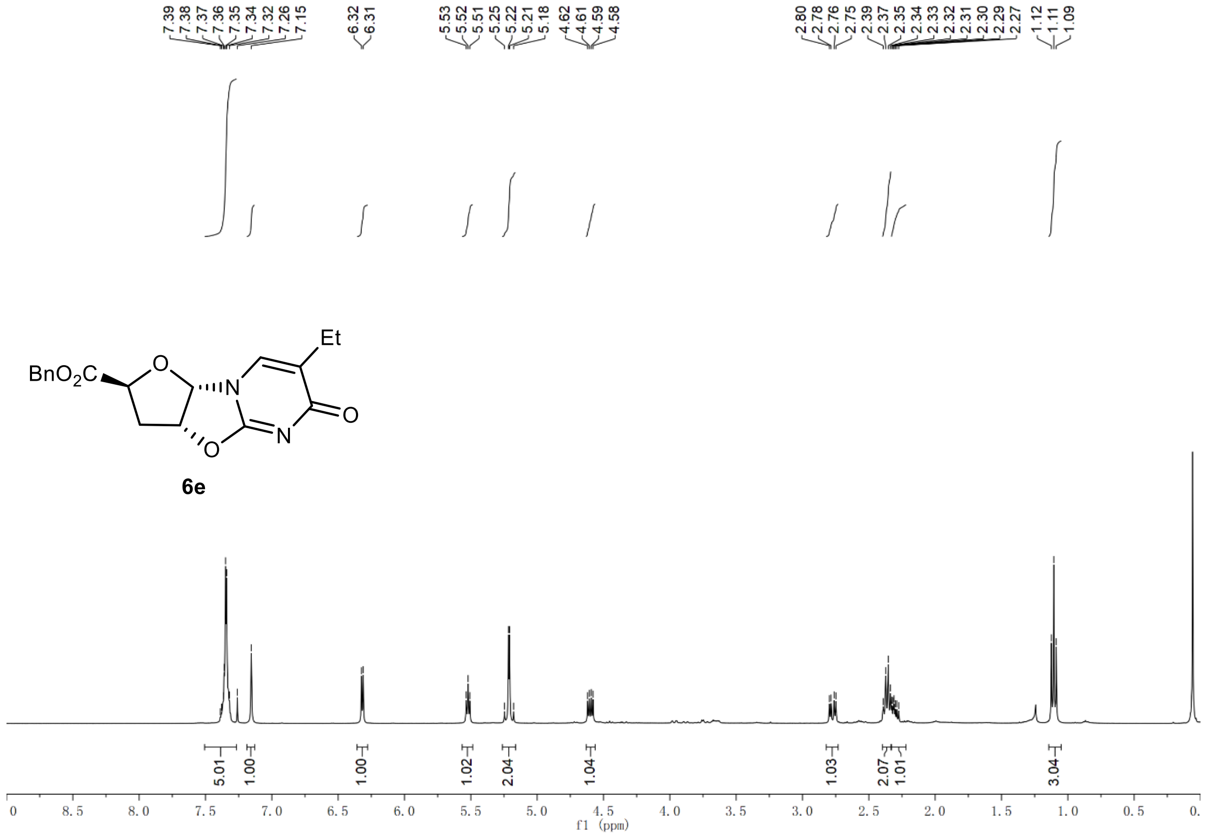

Supplementary Figure 145. ^{1}H NMR (400M, $CDCl_3$) of compound **6e.**

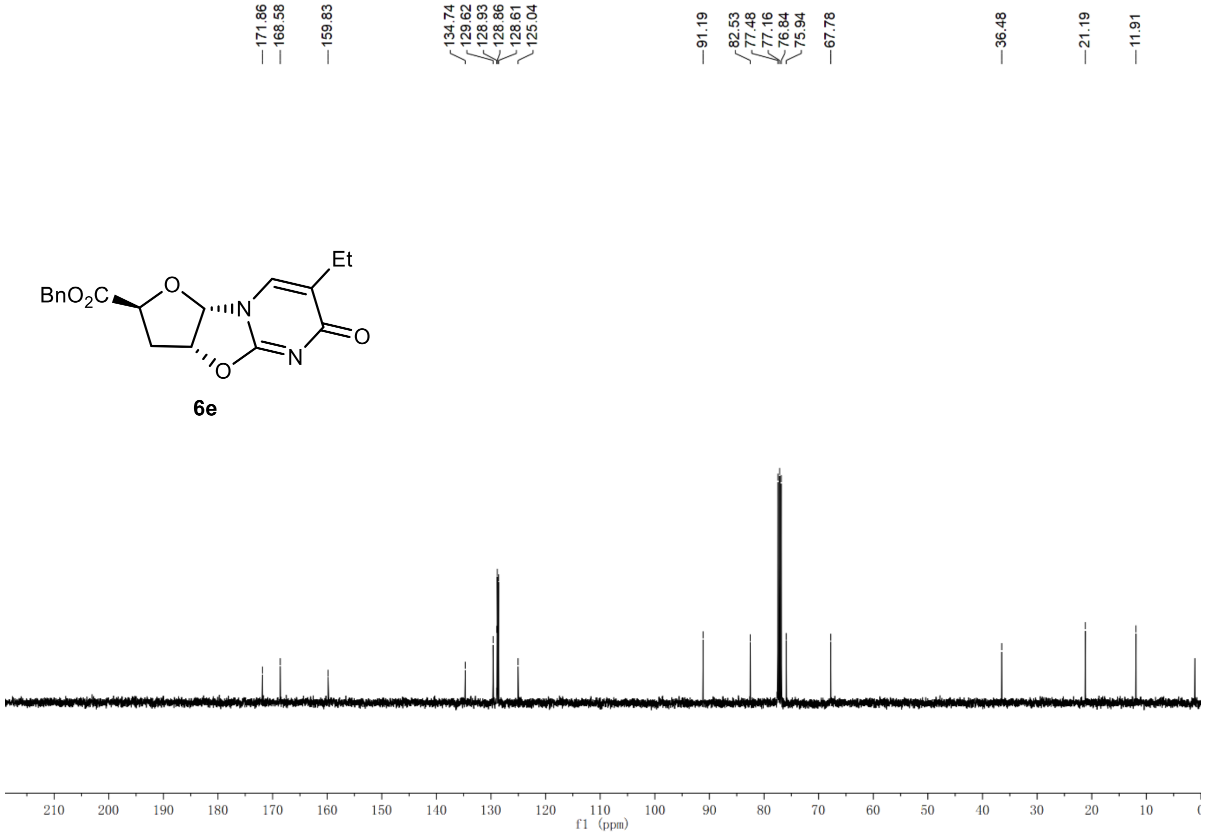

Supplementary Figure 146. ^{13}C NMR (100M, $CDCl_3$) of compound **6e.**

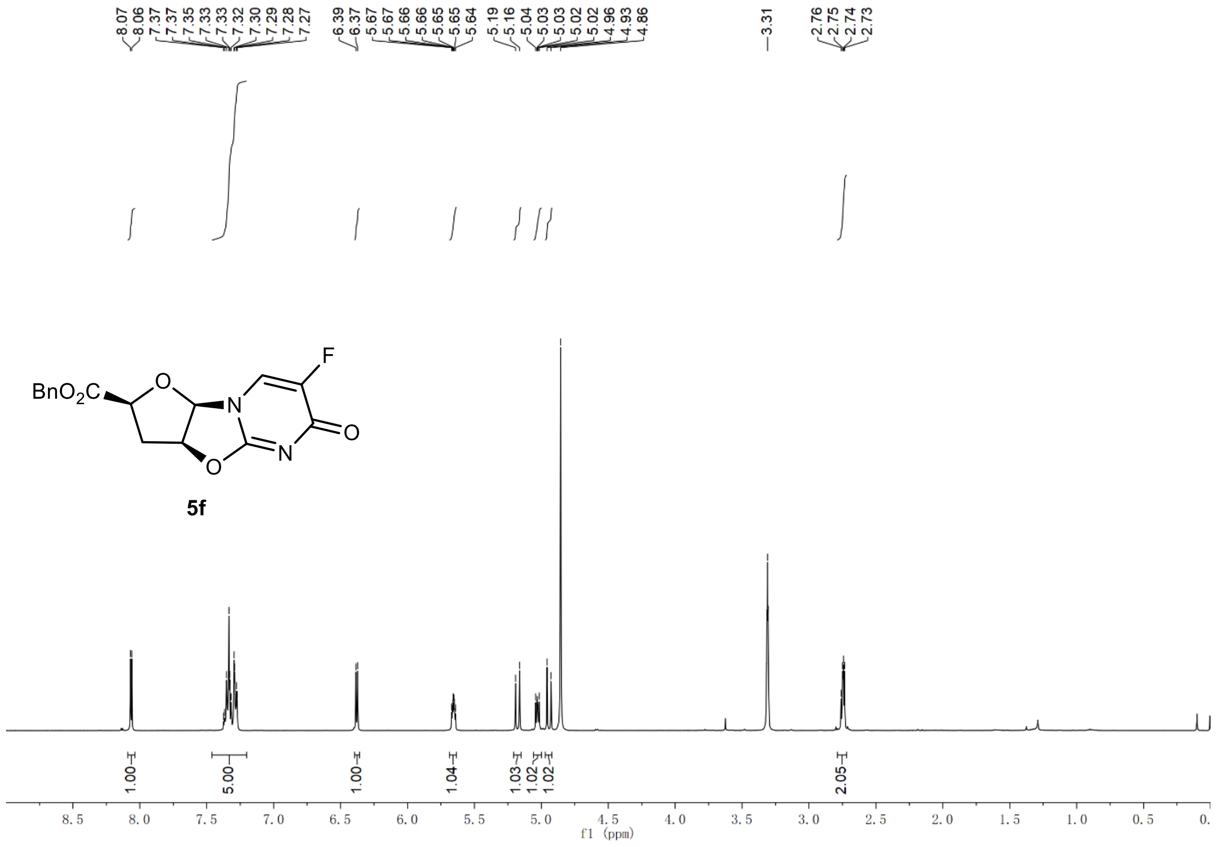

Supplementary Figure 147. ^{1}H NMR (400M, CD_3OD) of compound **5f.**

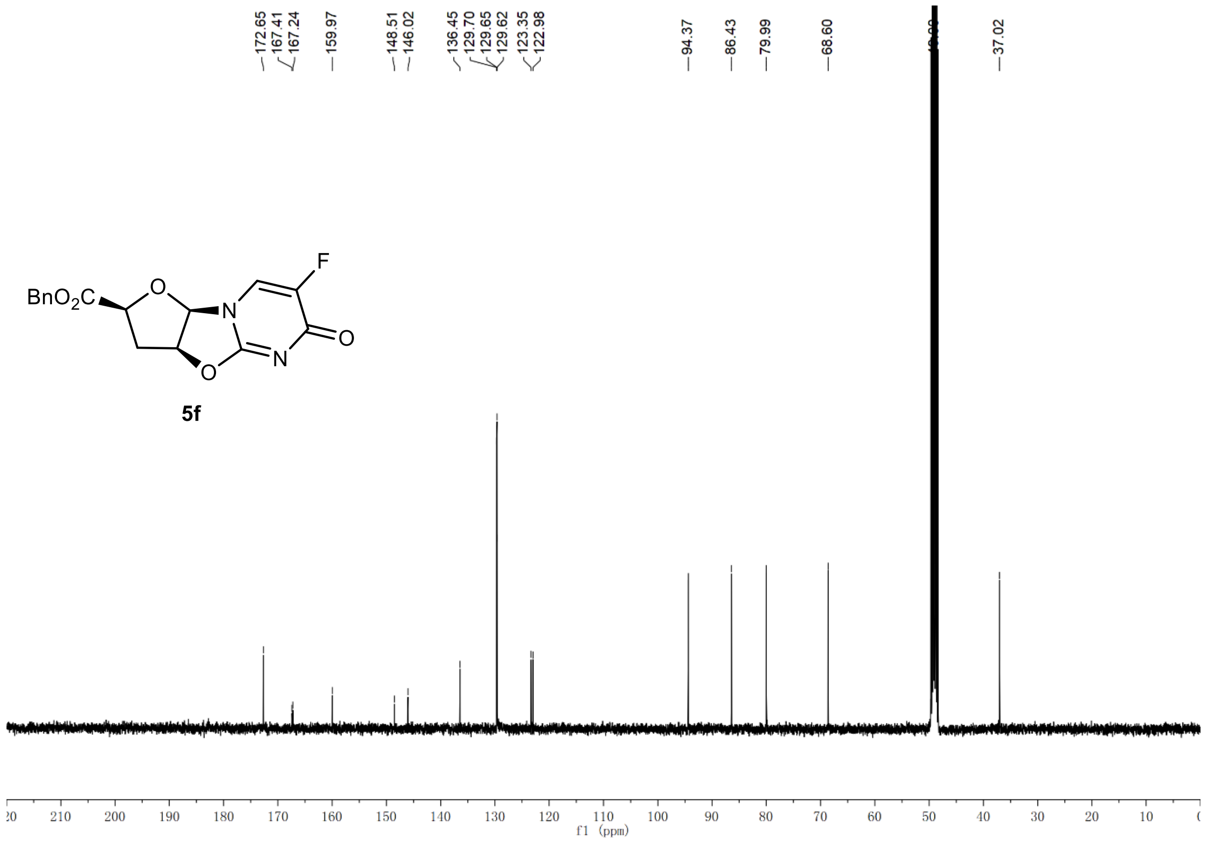

Supplementary Figure 148. ^{13}C NMR (100M, CD_3OD) of compound **5f.**

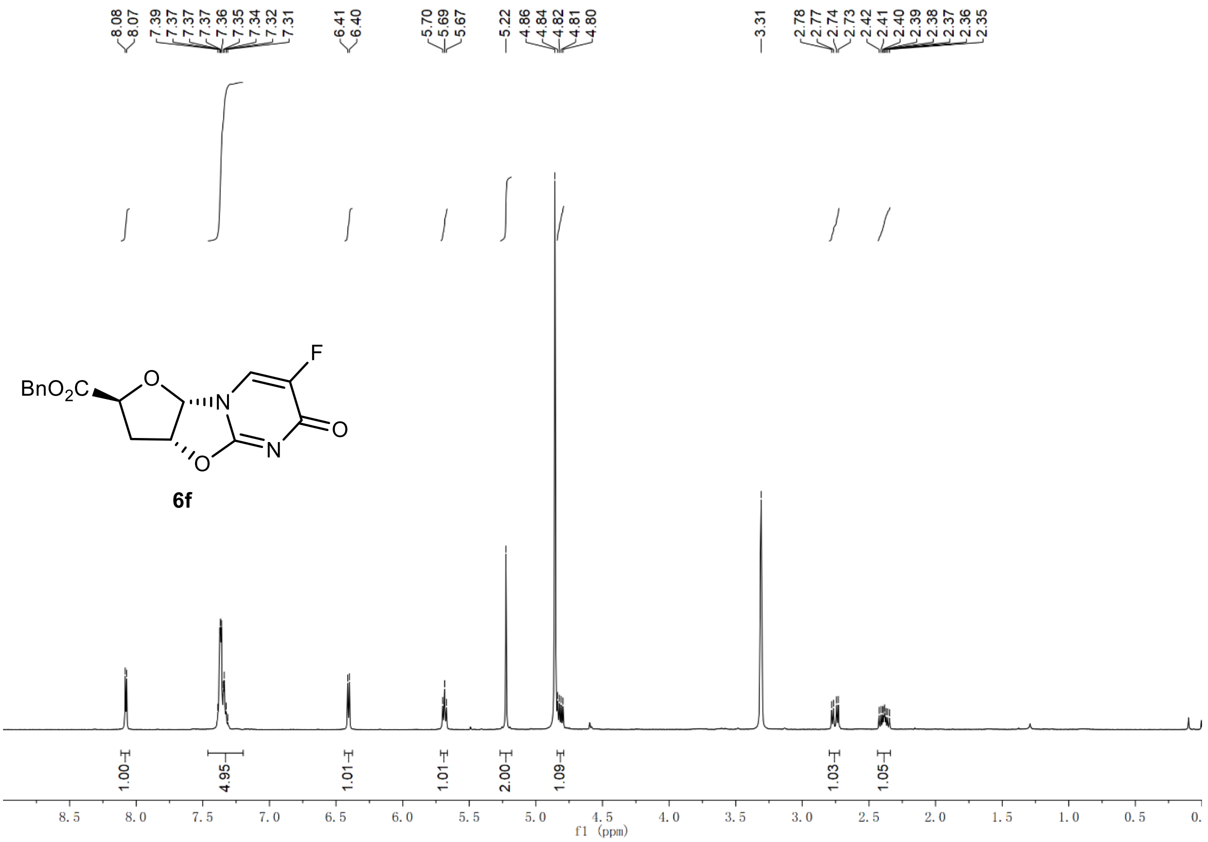

Supplementary Figure 149. ^{1}H NMR (400M, CD_3OD) of compound **6f.**

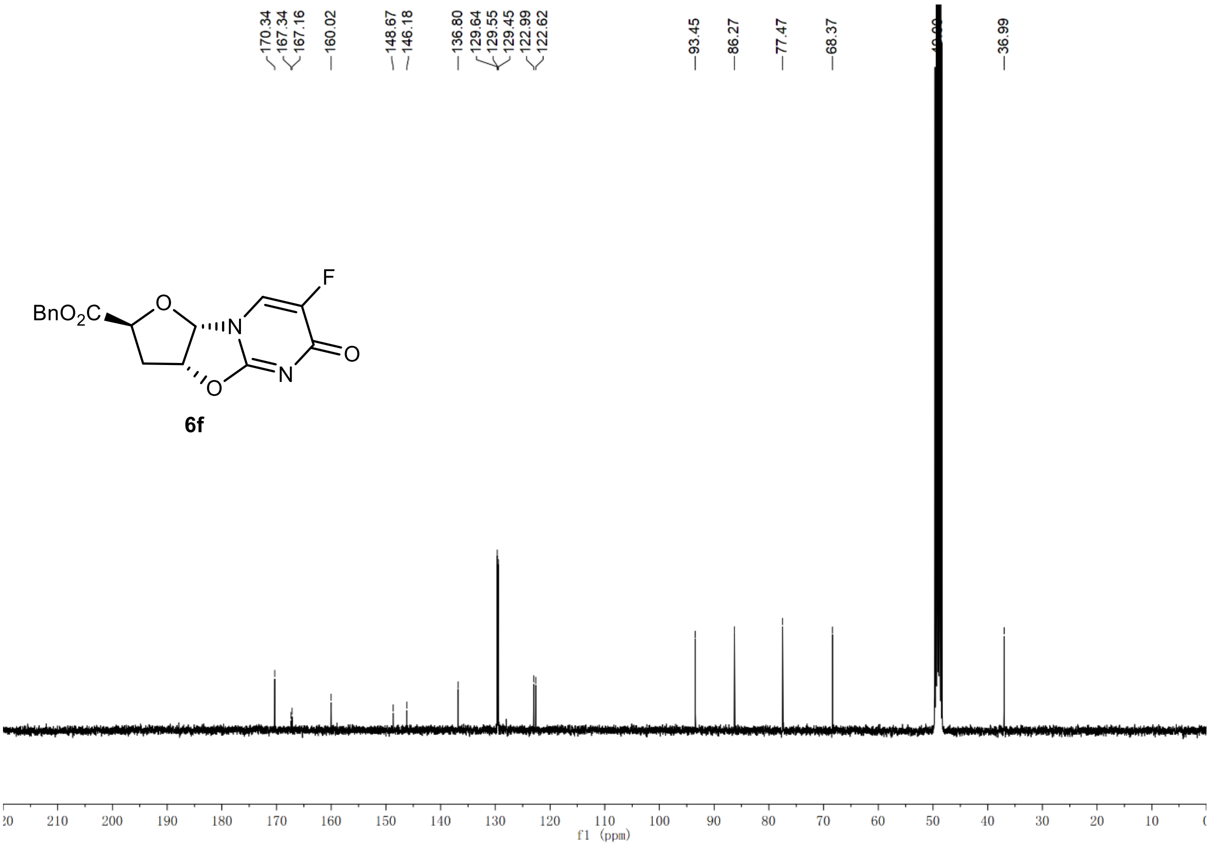

Supplementary Figure 150. ^{13}C NMR (100M, CD_3OD) of compound **6f.**

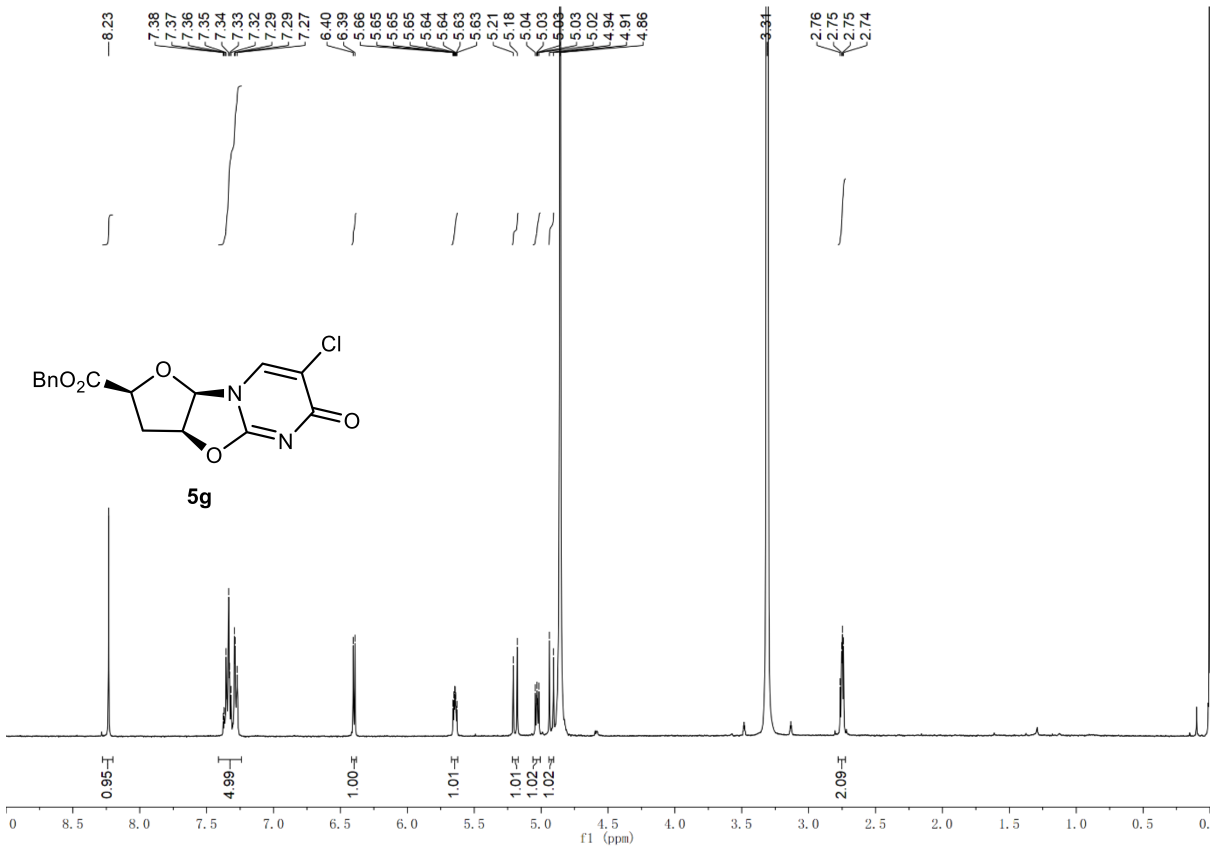

Supplementary Figure 151. ^{1}H NMR (400M, CD_3OD) of compound **5g.**

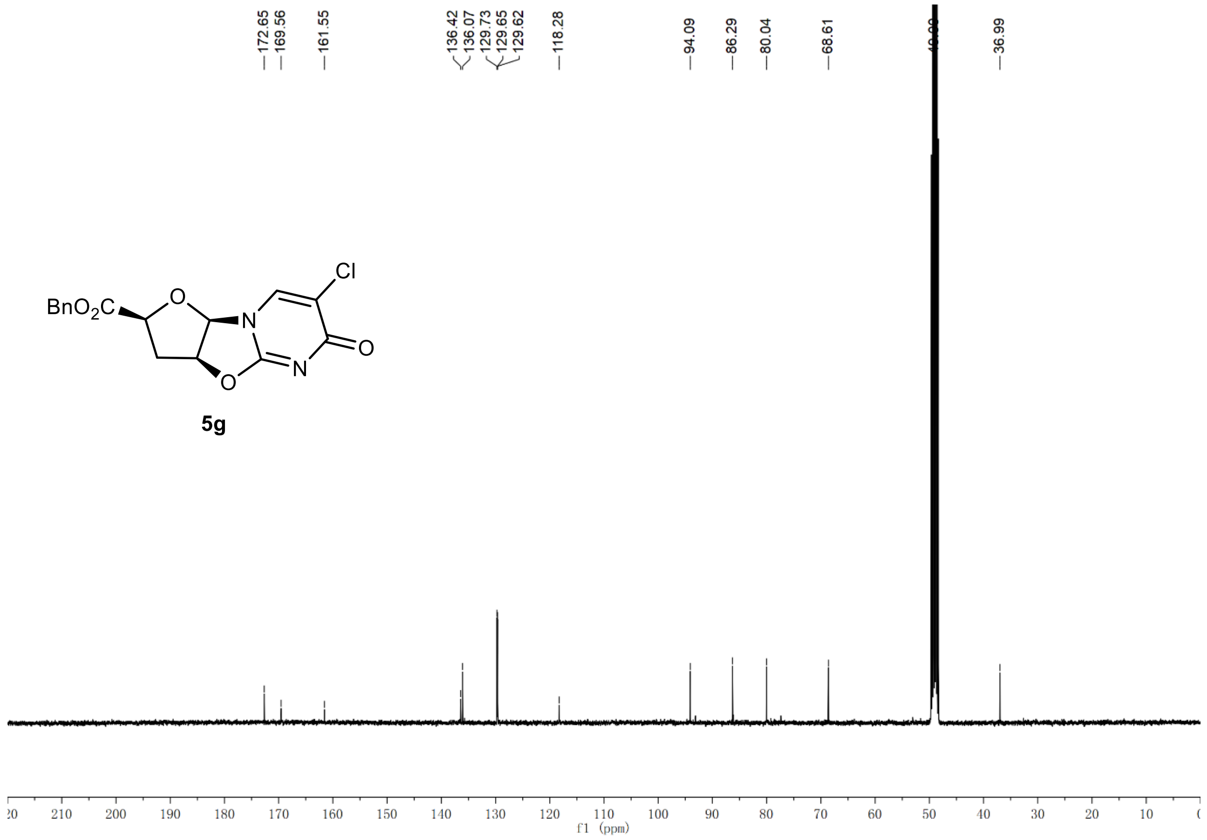

Supplementary Figure 152. ^{13}C NMR (100M, CD_3OD) of compound **5g.**

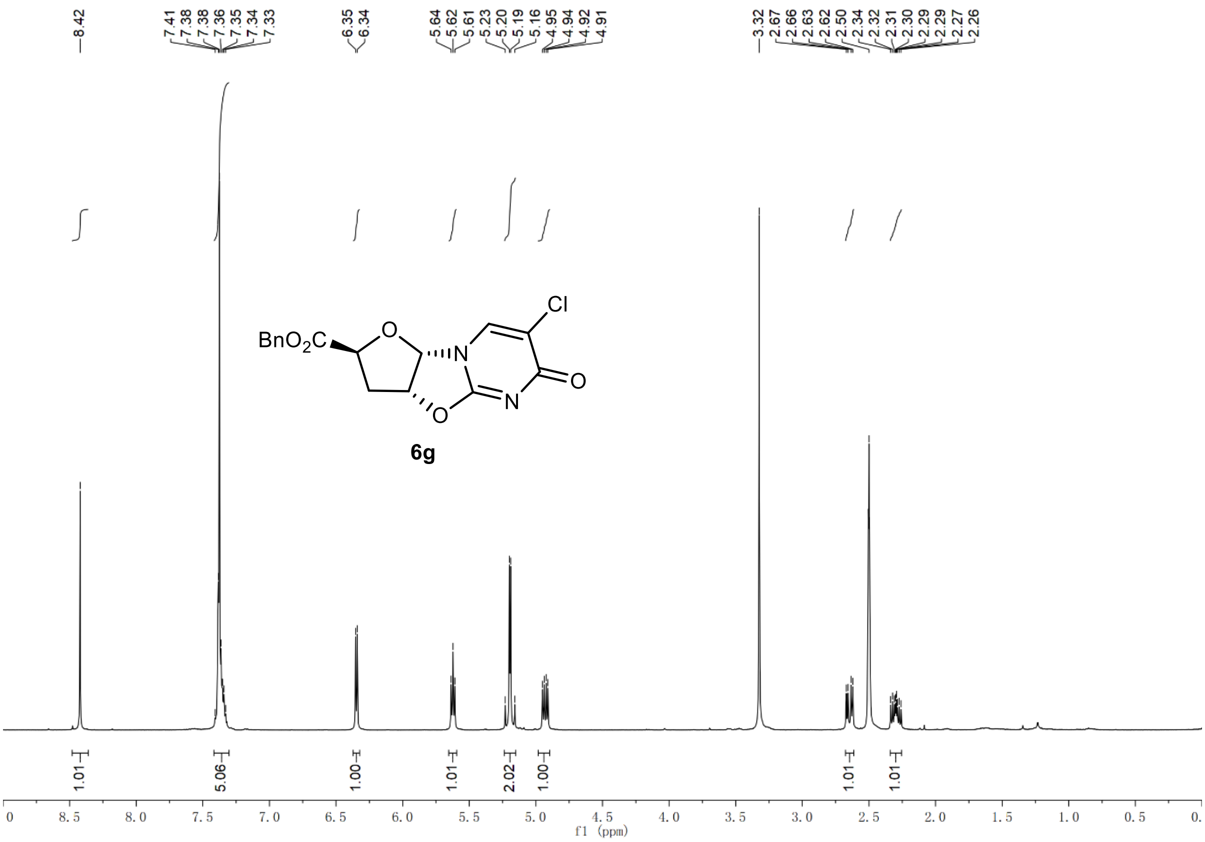

Supplementary Figure 153. ^{1}H NMR (400M, $(CD_3)_2SO$) of compound **6g.**

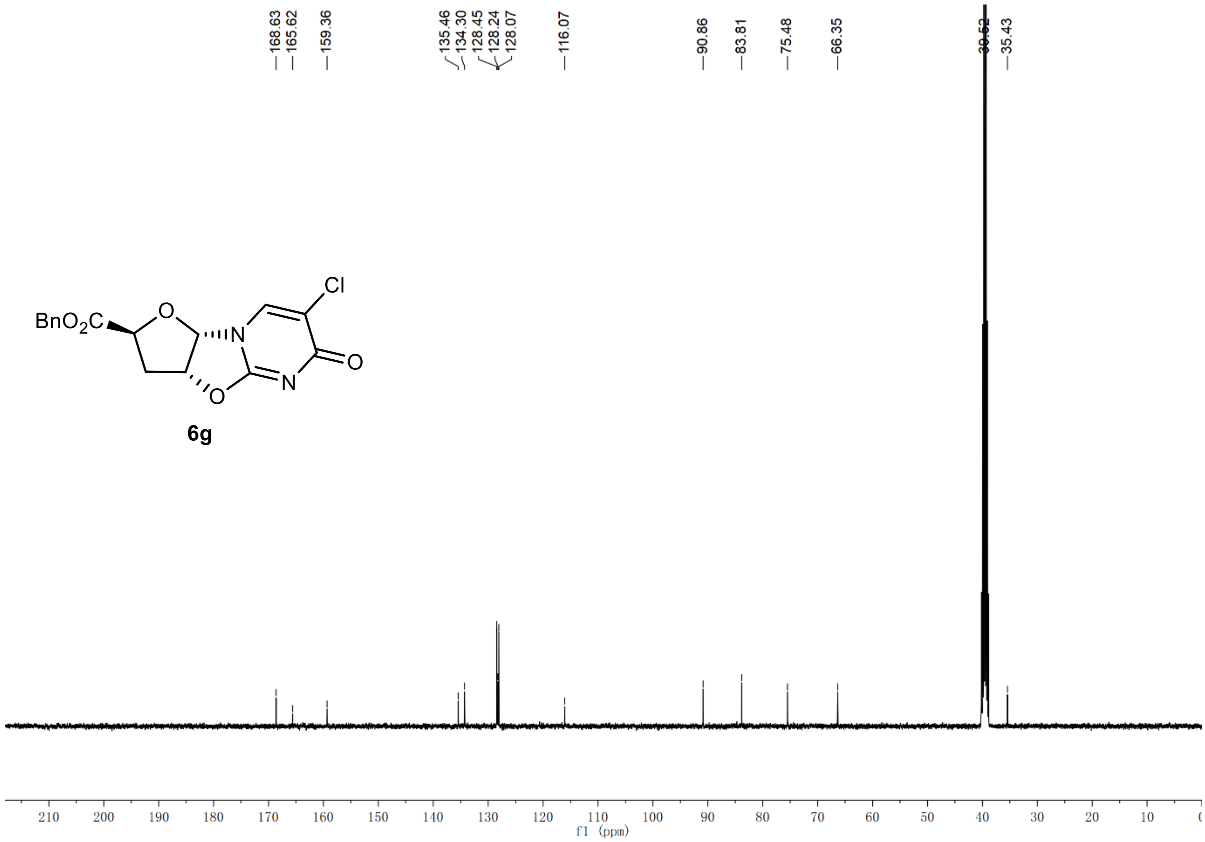

Supplementary Figure 154. ^{13}C NMR (100M, $(CD_3)_2SO$) of compound **6g.**

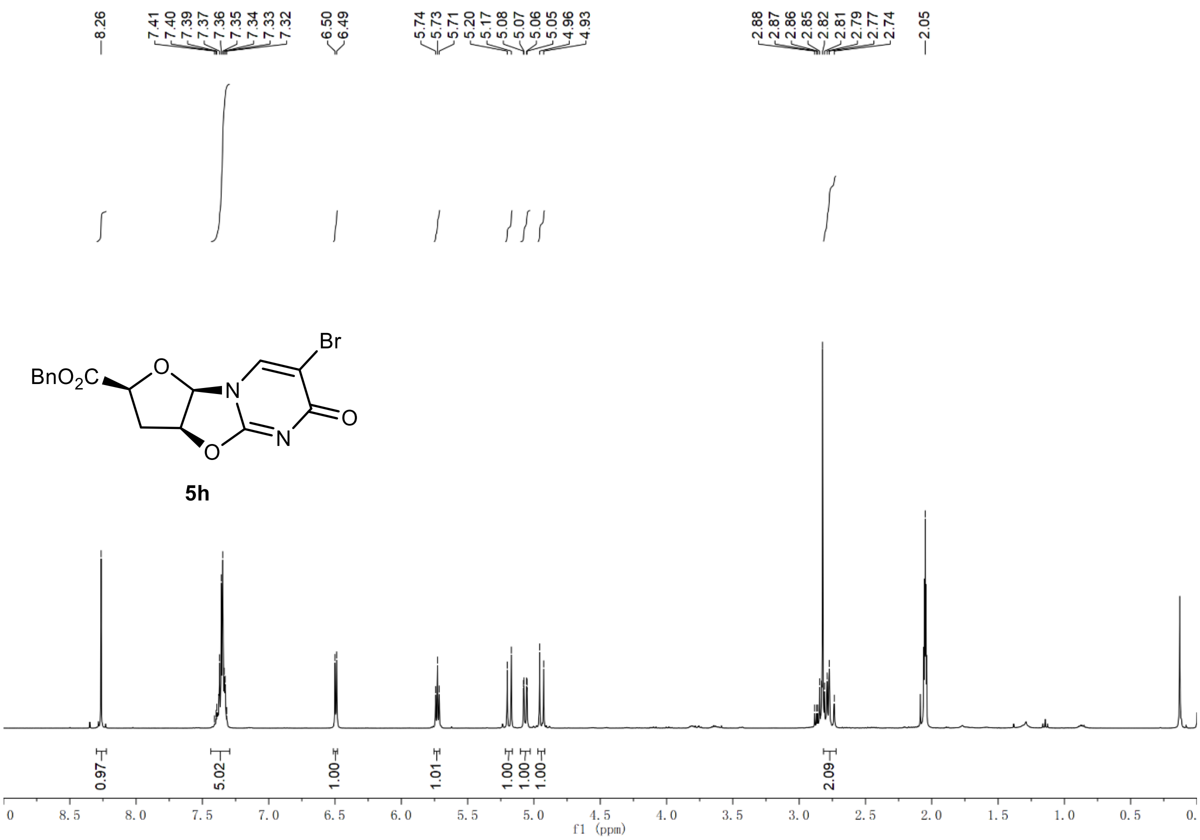

Supplementary Figure 155. ^{1}H NMR (400M, $(CD_3)_2CO$) of compound **5h.**

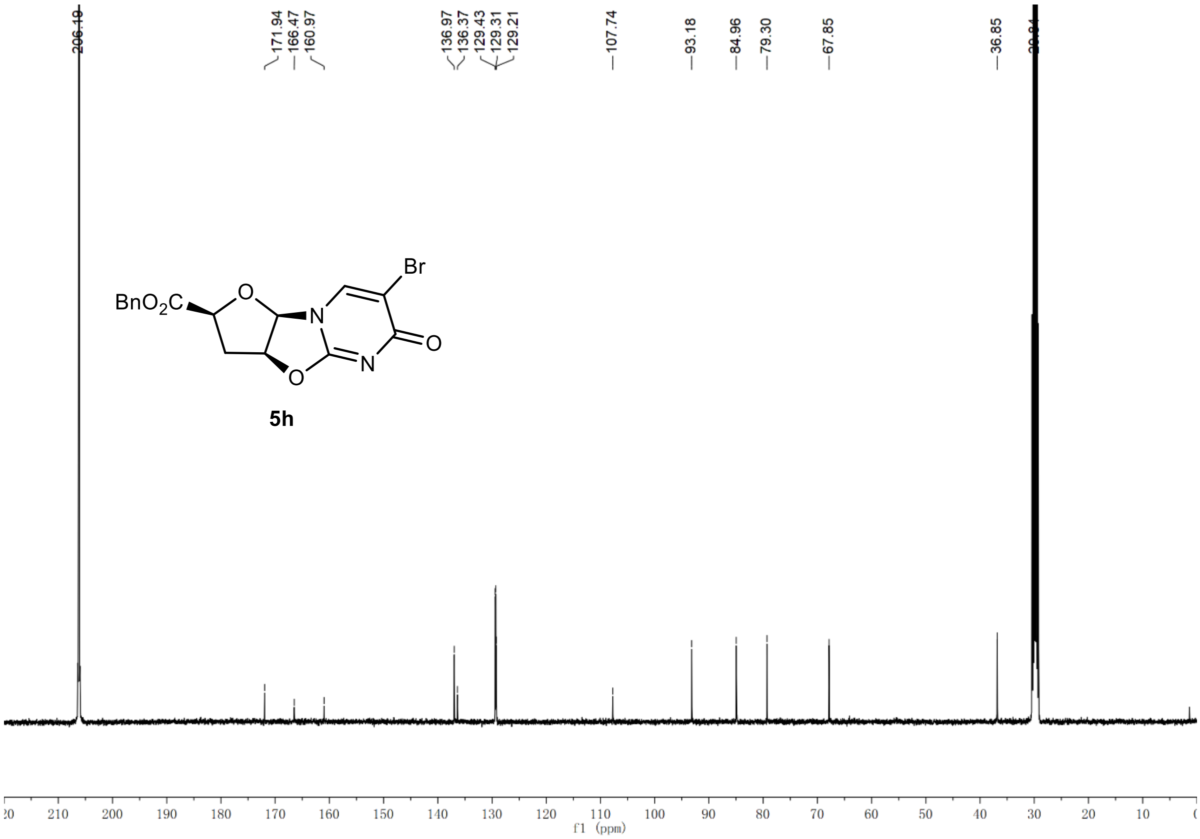

Supplementary Figure 156. ^{13}C NMR (100M, $(CD_3)_2CO$) of compound **5h.**

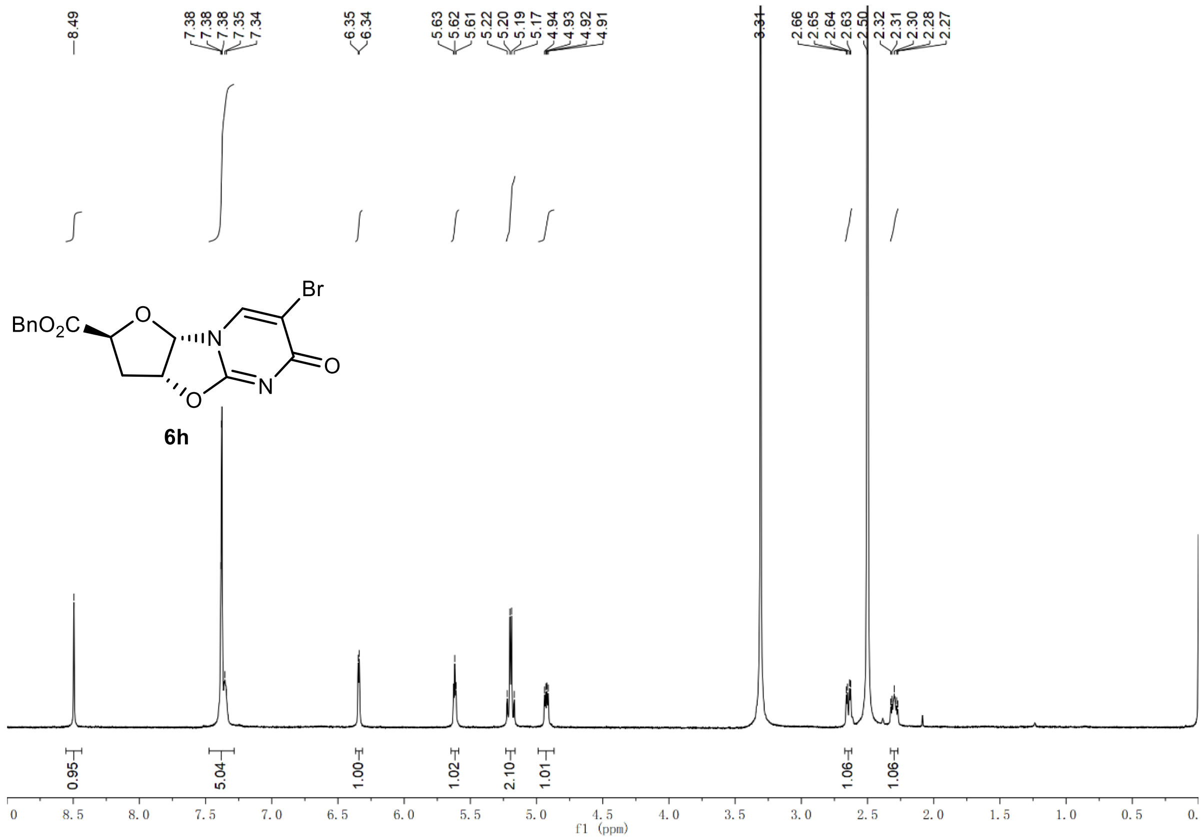

Supplementary Figure 157. ^{1}H NMR (400M, $(CD_3)_2SO$) of compound **6h.**

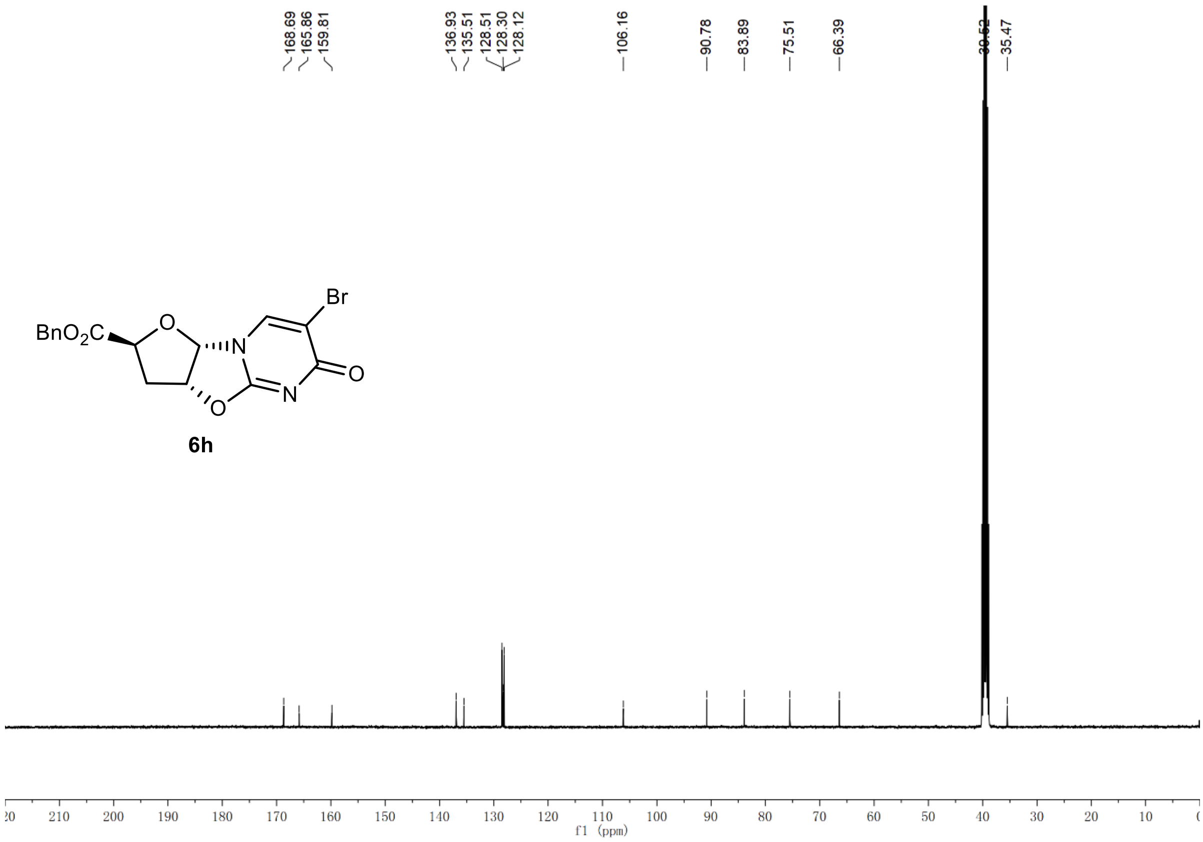

Supplementary Figure 158. ^{13}C NMR (100M, $(CD_3)_2SO$) of compound **6h.**

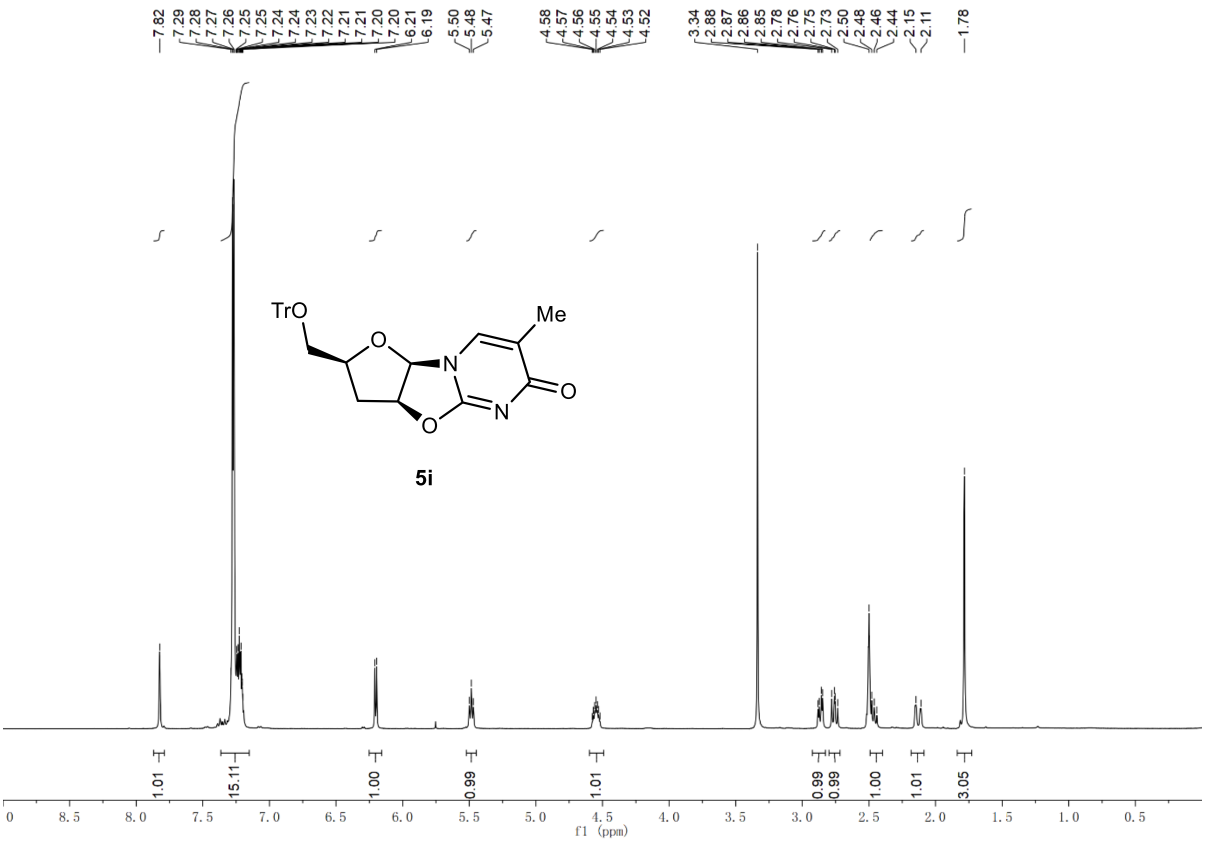

Supplementary Figure 159. ^{1}H NMR (400M, $(CD_3)_2SO$) of compound **5i.**

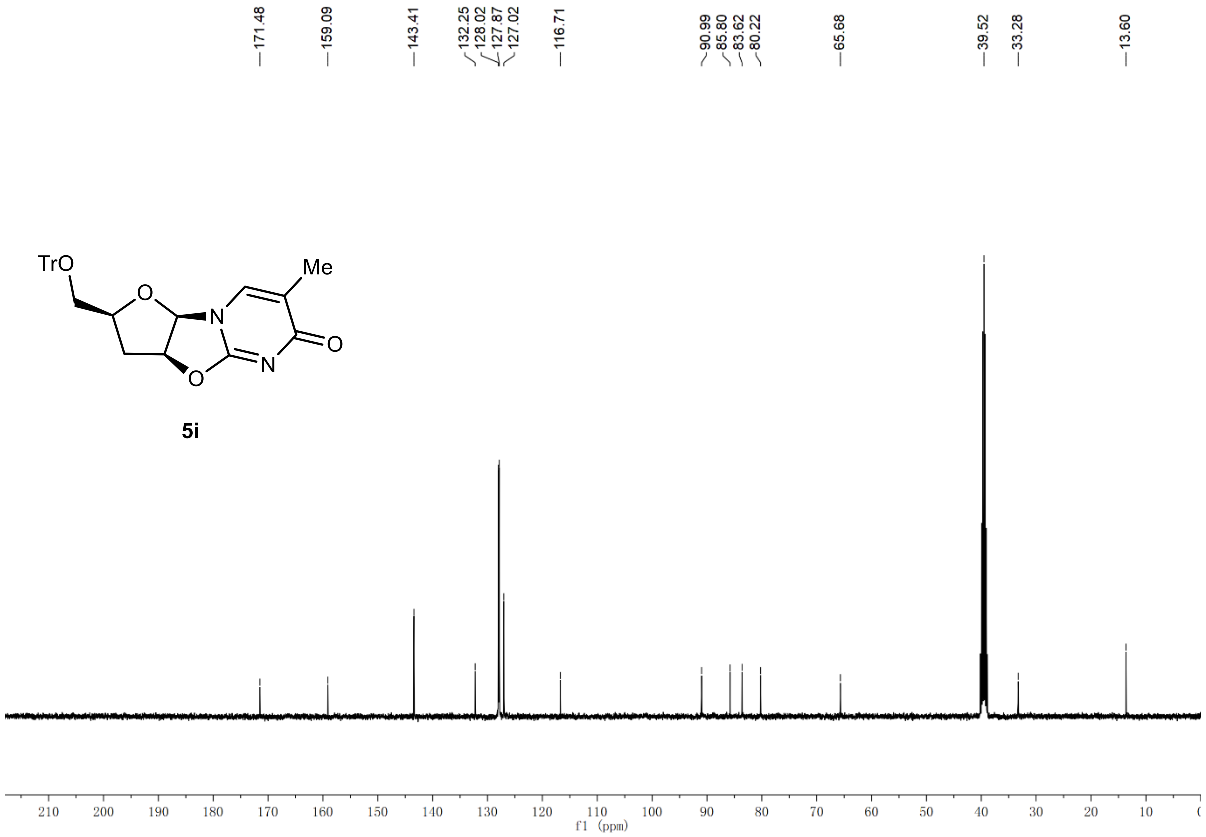

Supplementary Figure 160. ^{13}C NMR (100M, $(CD_3)_2SO$) of compound **5i.**

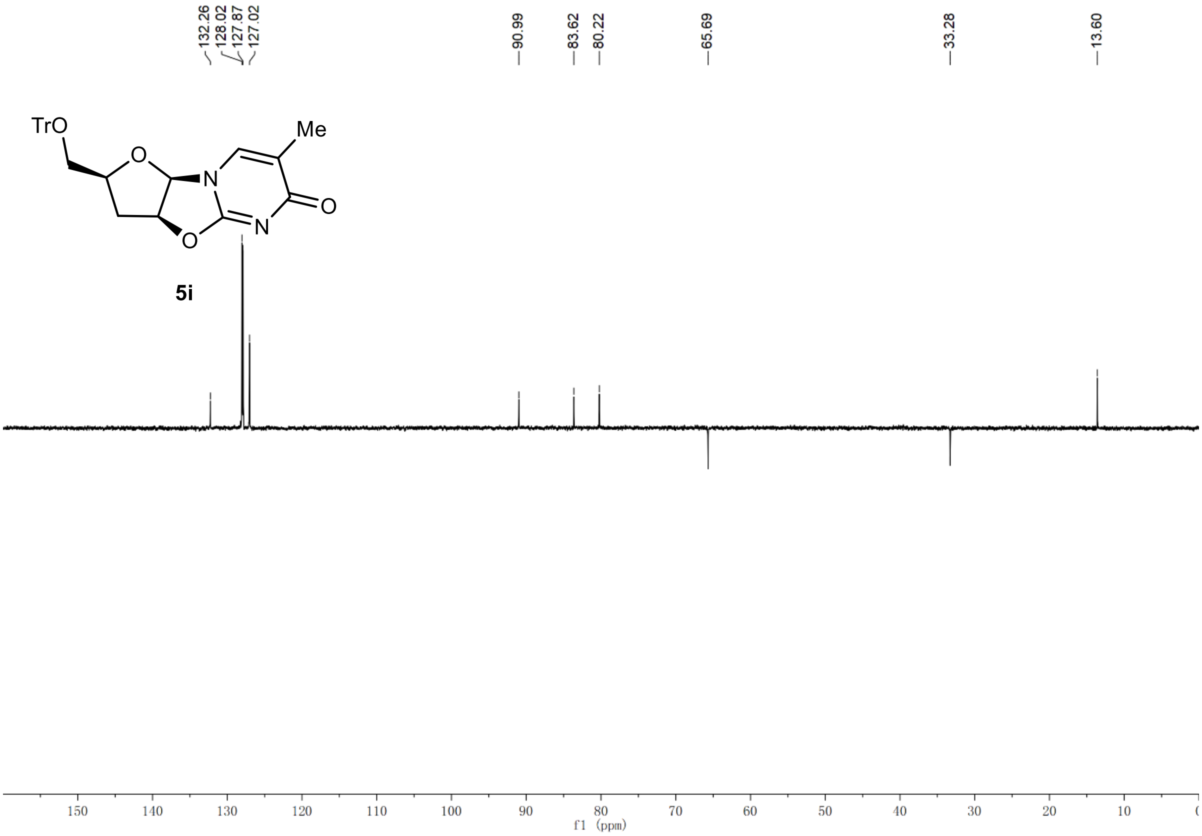

Supplementary Figure 161. DEPT 135° (100M, $(CD_3)_2SO$) of compound **5i.**

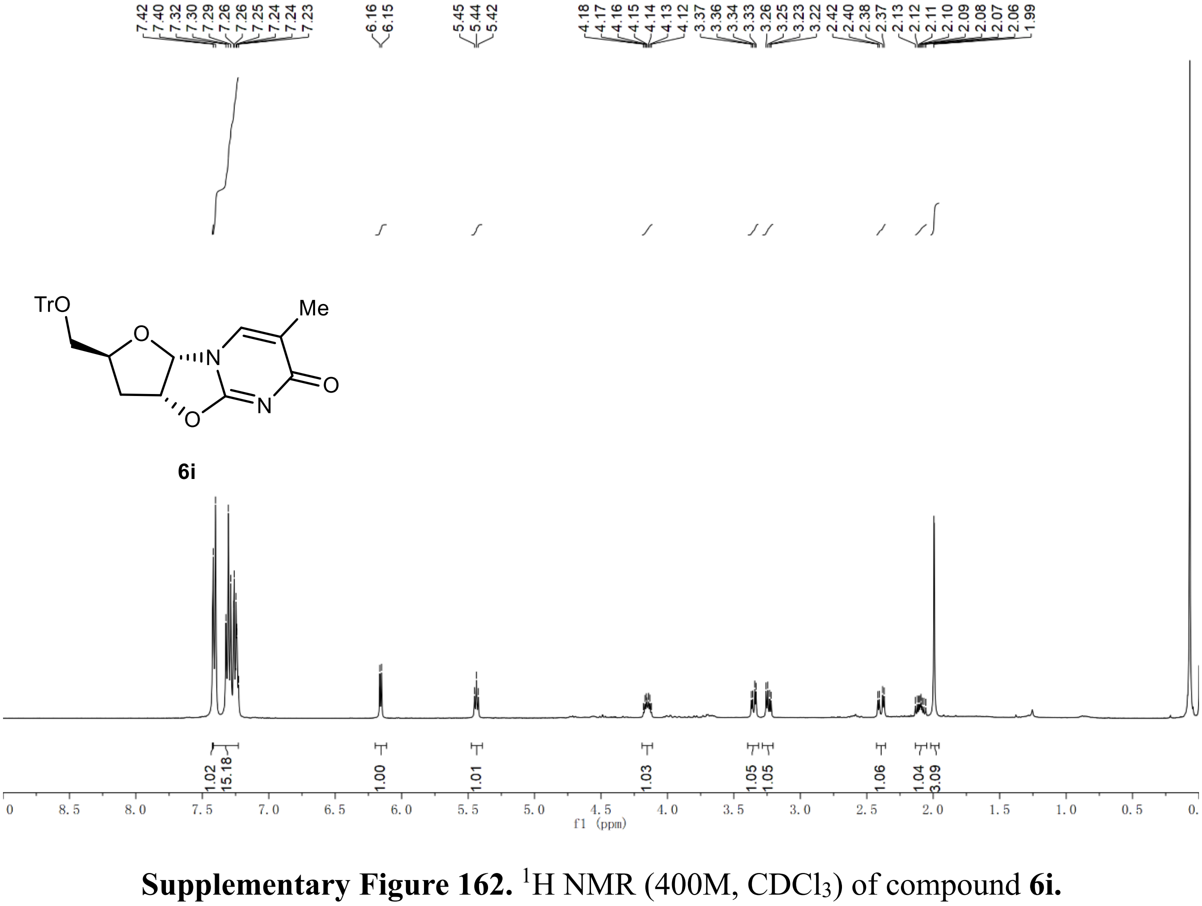

Supplementary Figure 162. ^1H NMR (400M, $CDCl_3$) of compound **6i.**

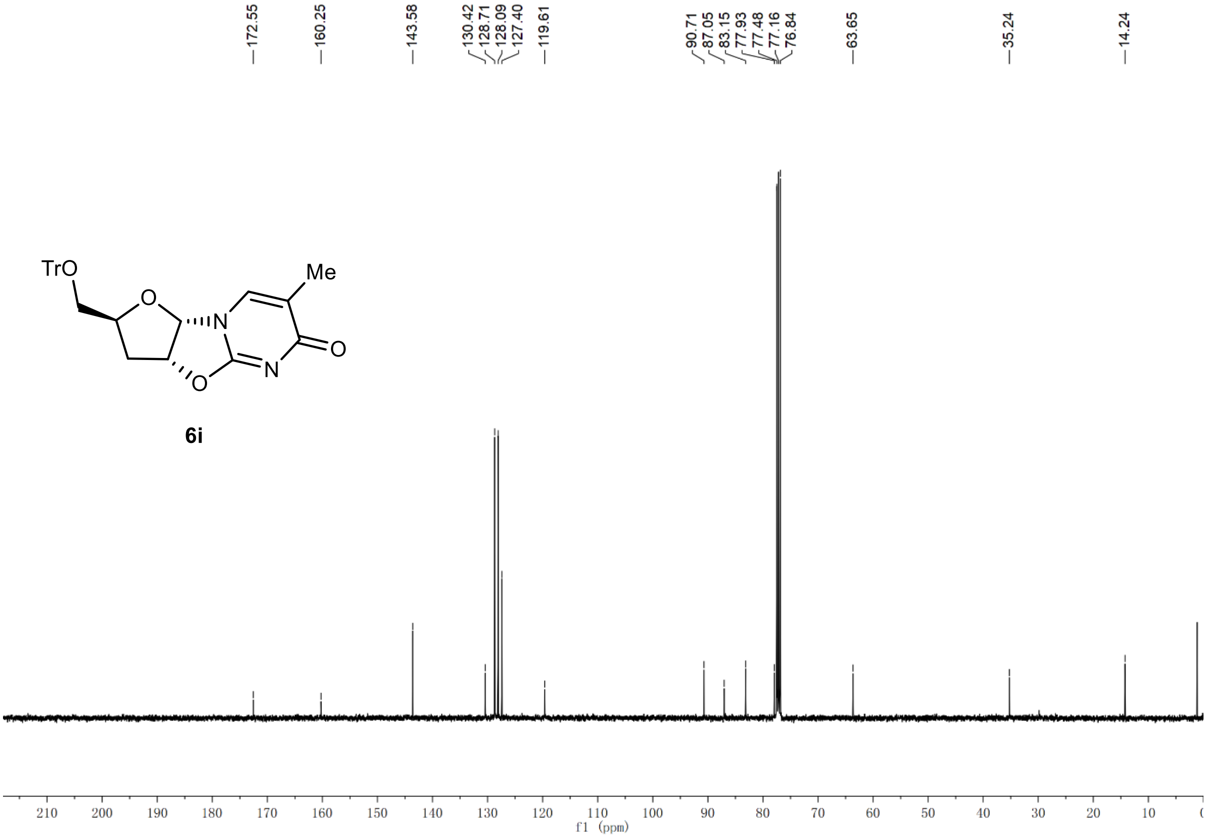

6i

Supplementary Figure 163. ^{13}C NMR (100M, $CDCl_3$) of compound **6i.**

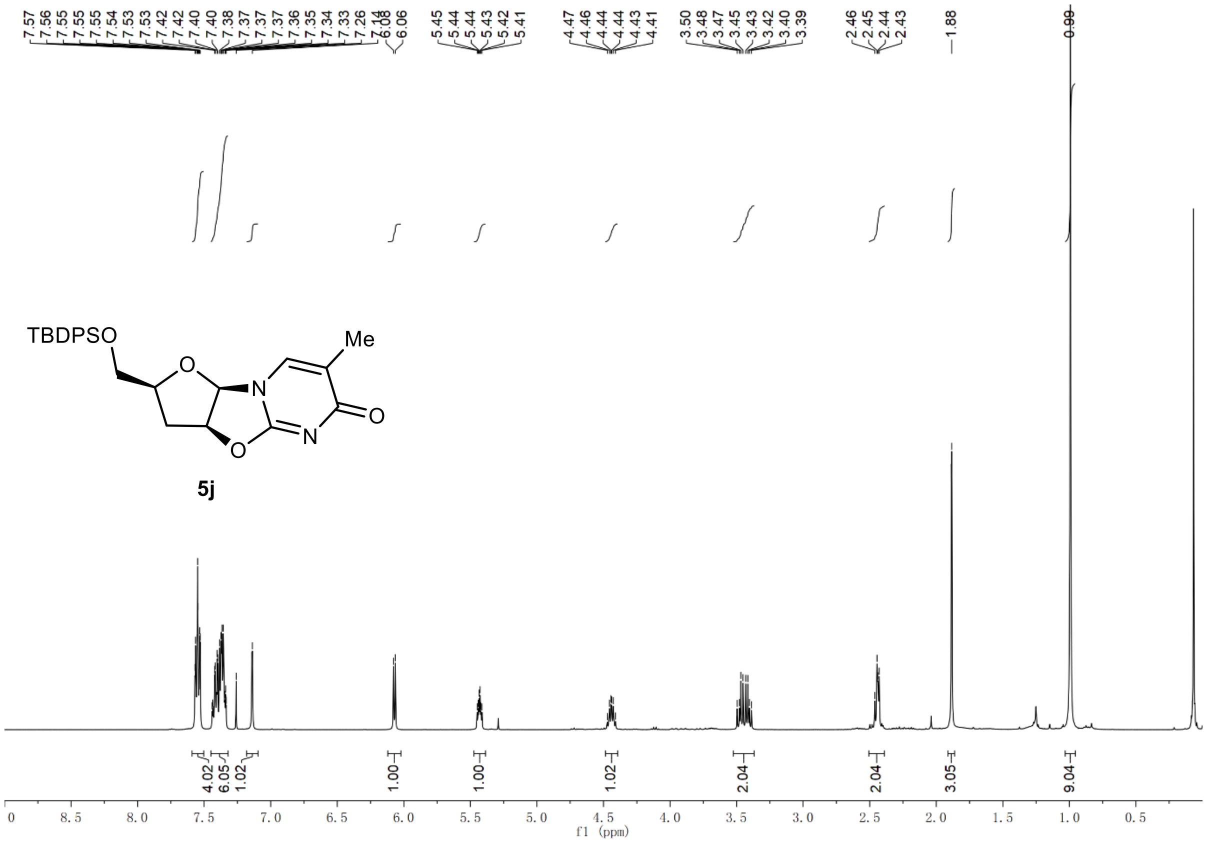

5j

Supplementary Figure 164. ^{1}H NMR (400M, $CDCl_3$) of compound **5j.**

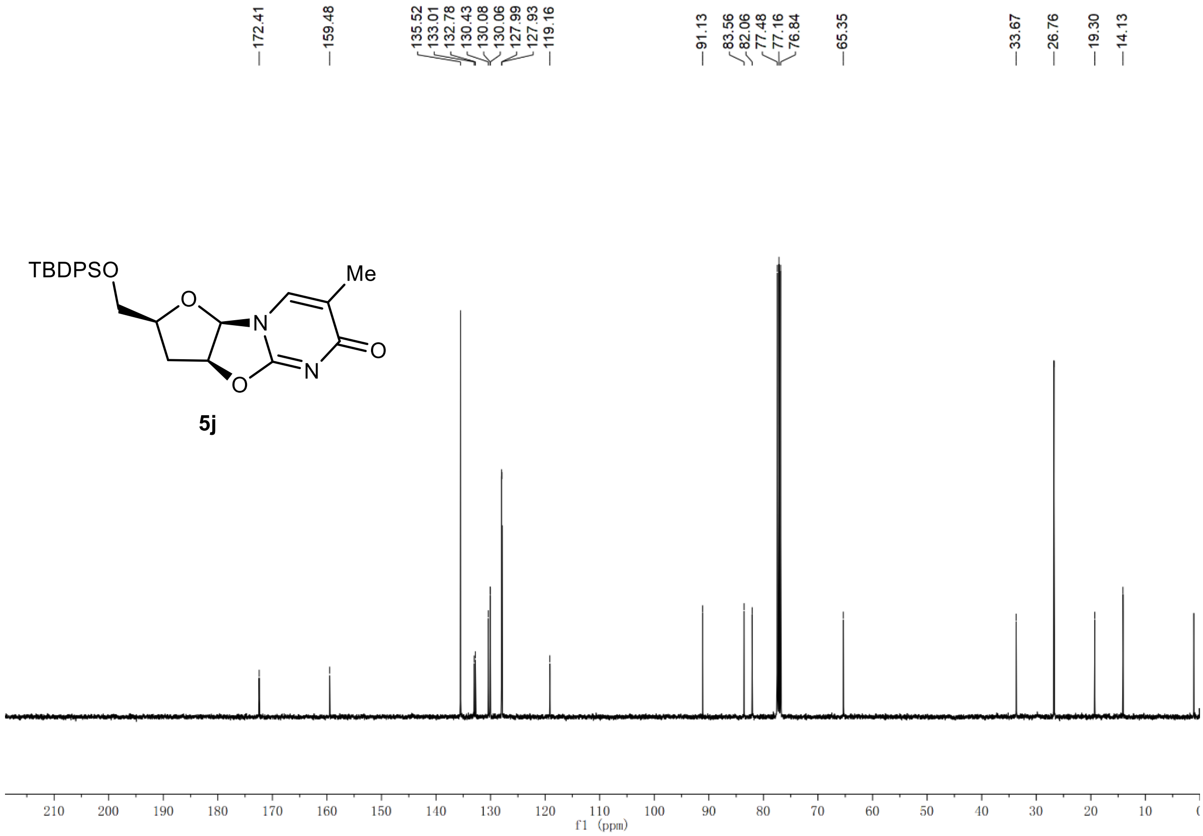

Supplementary Figure 165. ^{13}C NMR (100M, $CDCl_3$) of compound **5j.**

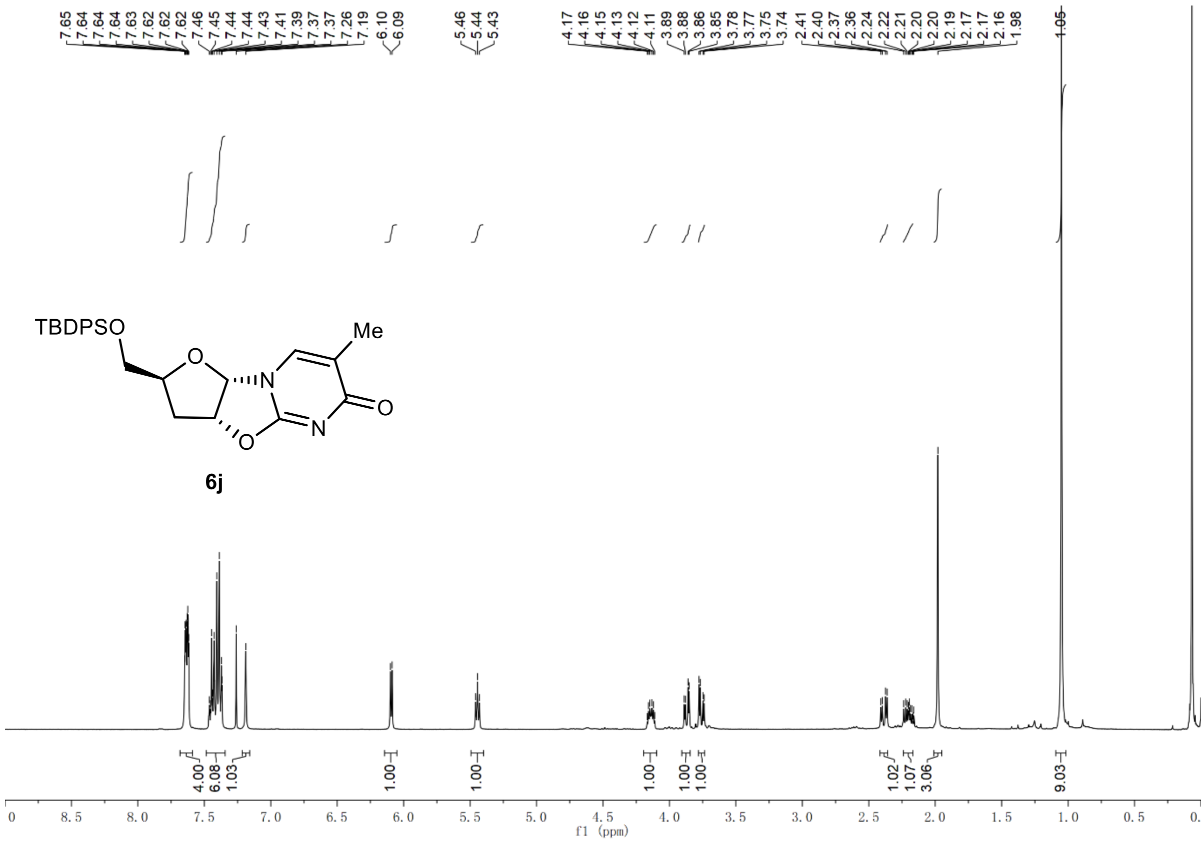

Supplementary Figure 166. ^{1}H NMR (400M, $CDCl_3$) of compound **6j.**

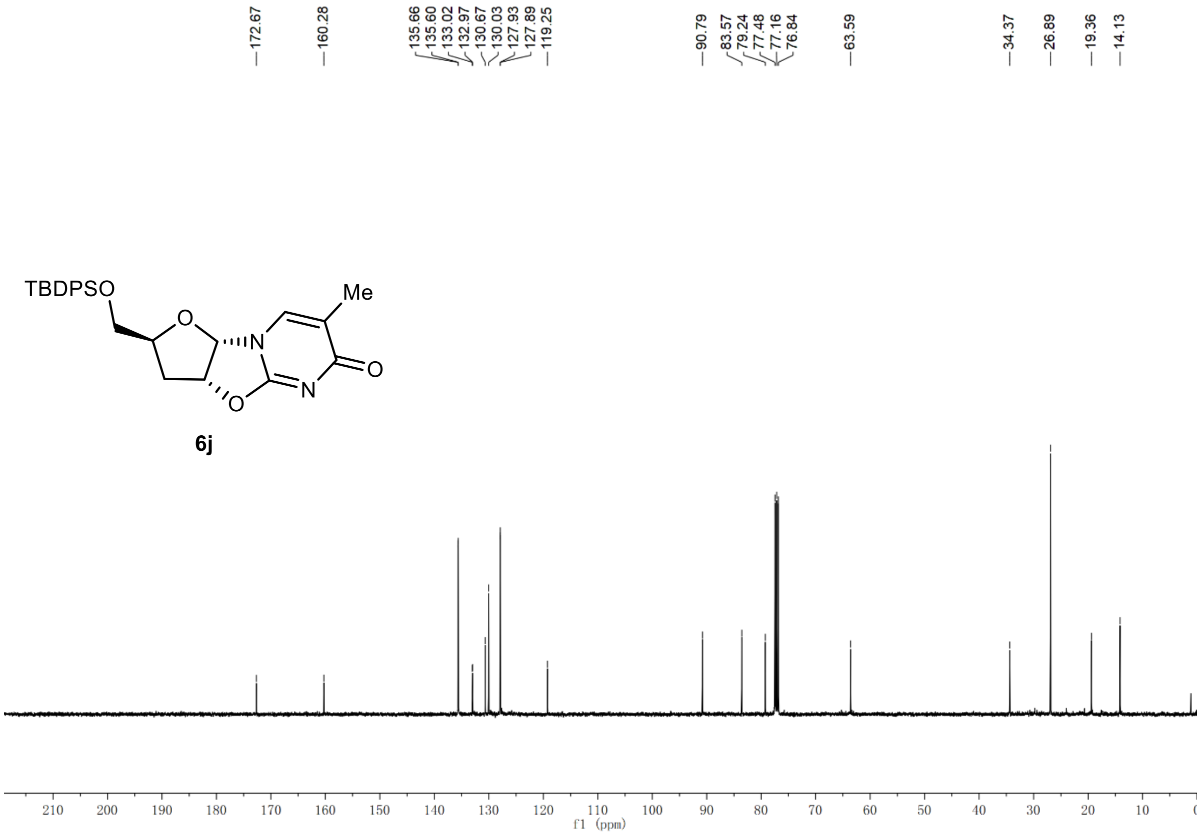

Supplementary Figure 167. ^{13}C NMR (100M, $CDCl_3$) of compound **6j.**

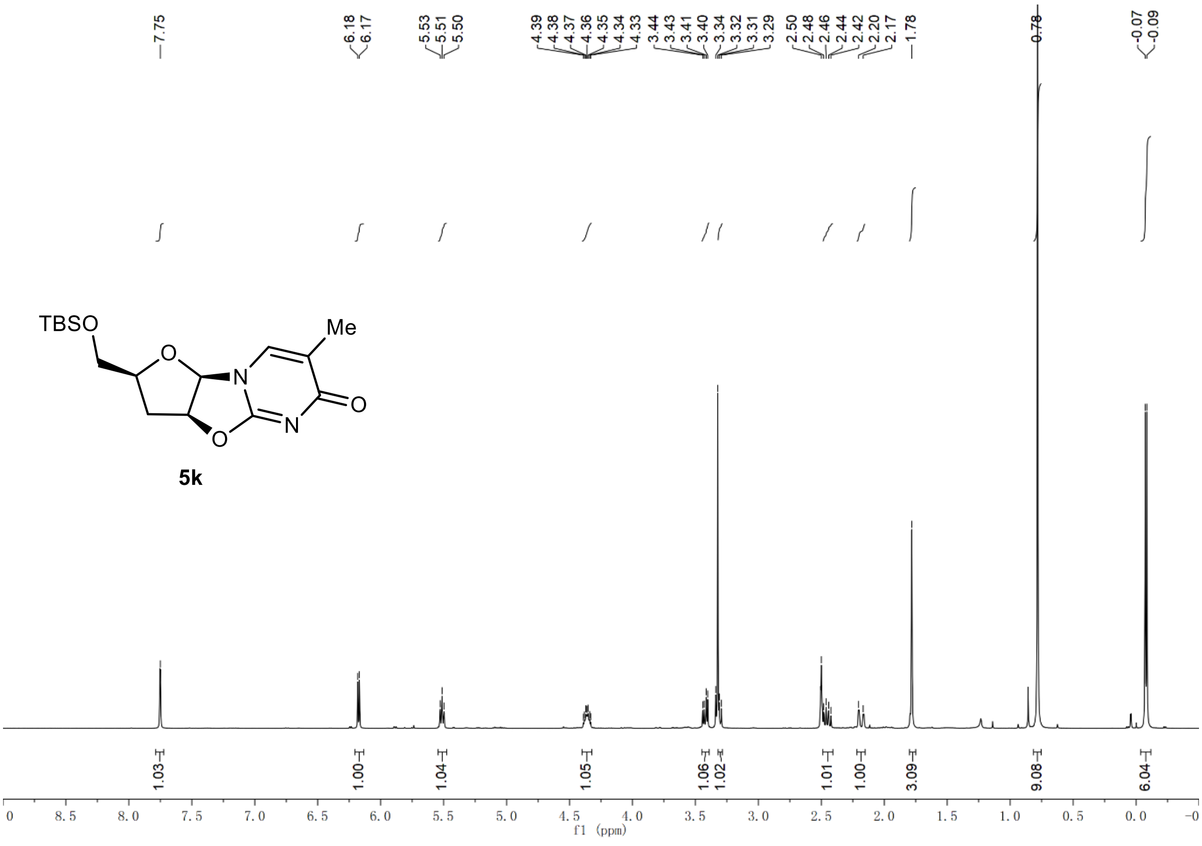

Supplementary Figure 168. ^{1}H NMR (400M, $(CD_3)_2SO$) of compound **5k.**

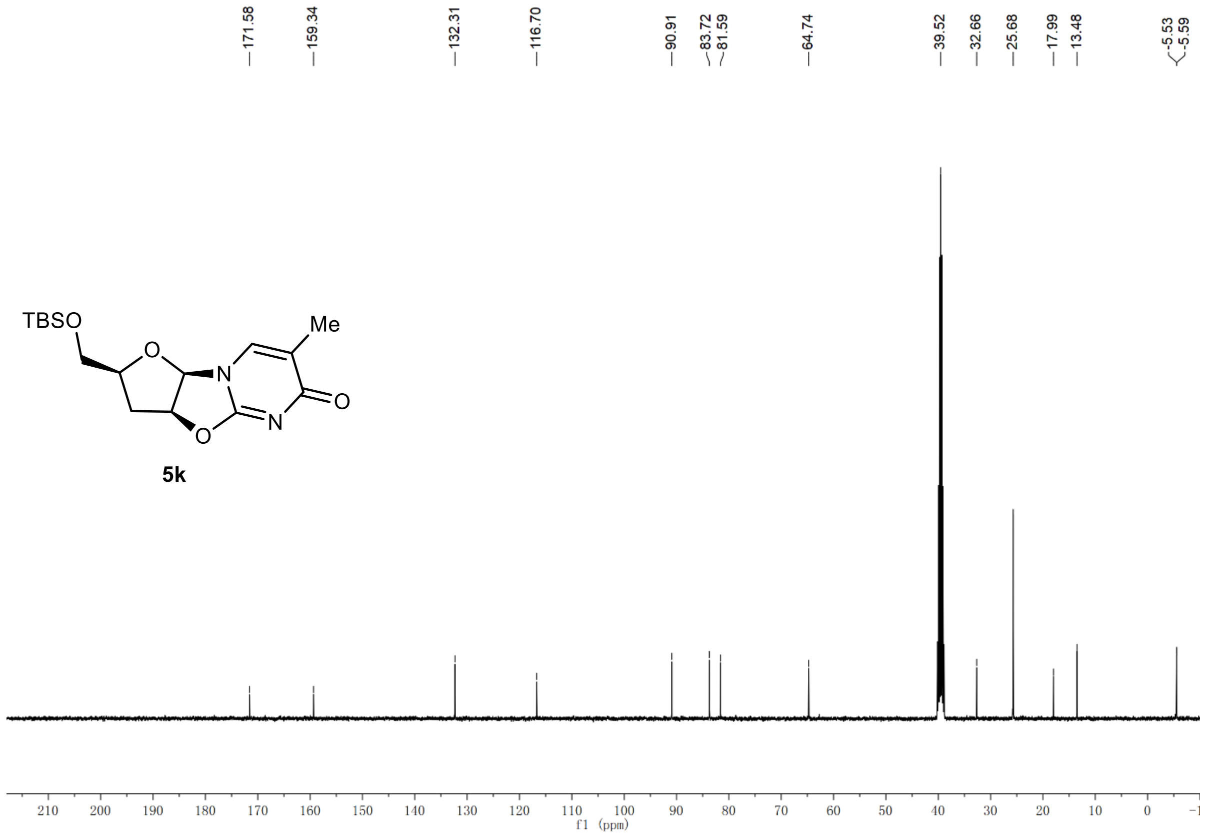

Supplementary Figure 169. ^{13}C NMR (100M, $(CD_3)_2SO$) of compound **5k.**

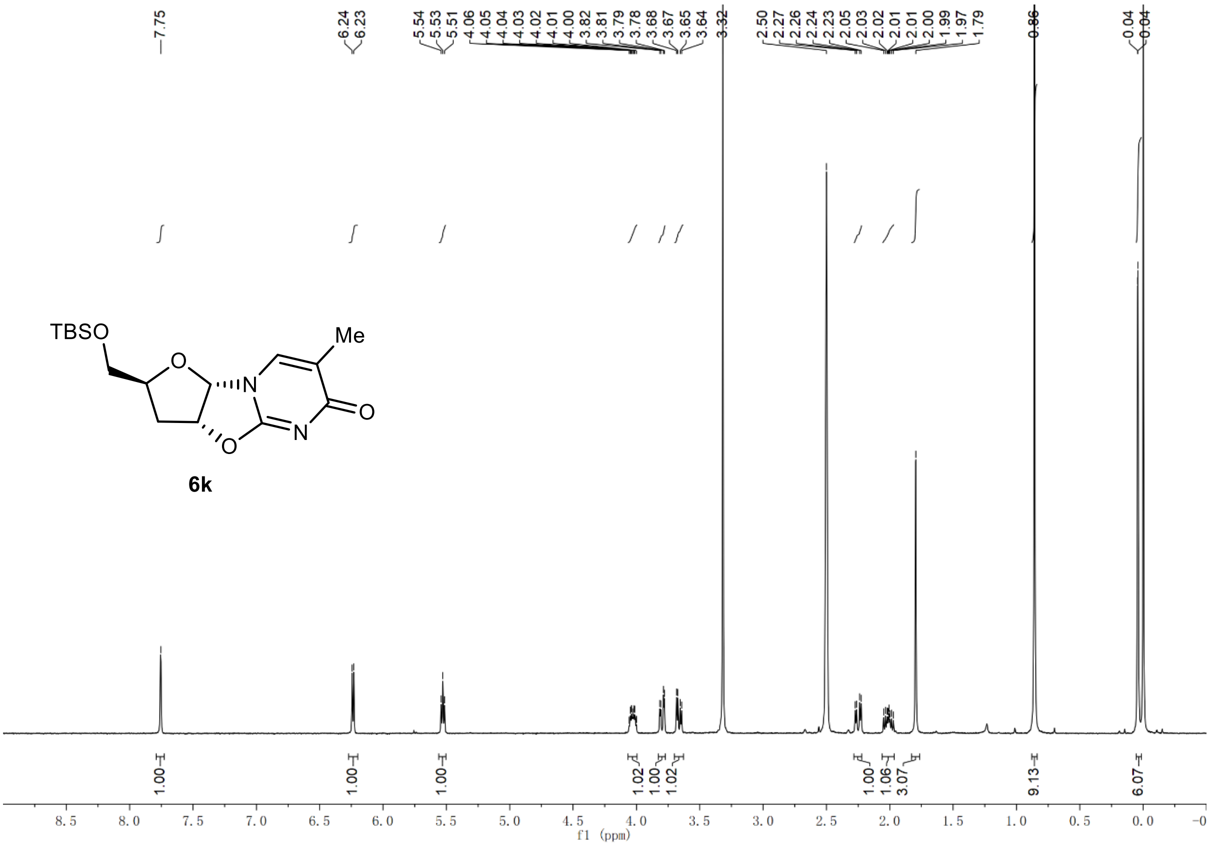

Supplementary Figure 170. ^{1}H NMR (400M, $(CD_3)_2SO$) of compound **6k.**

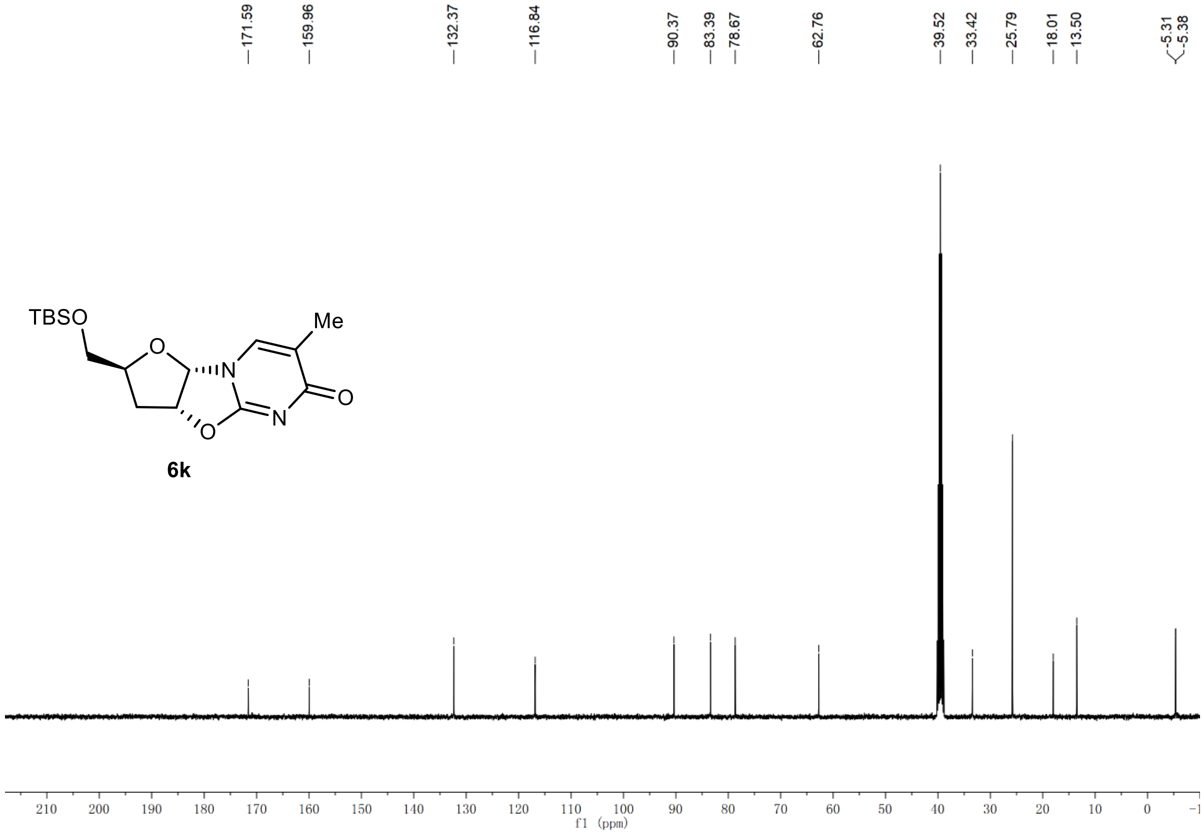

Supplementary Figure 171. ^{13}C NMR (100M, $(CD_3)_2SO$) of compound **6k.**

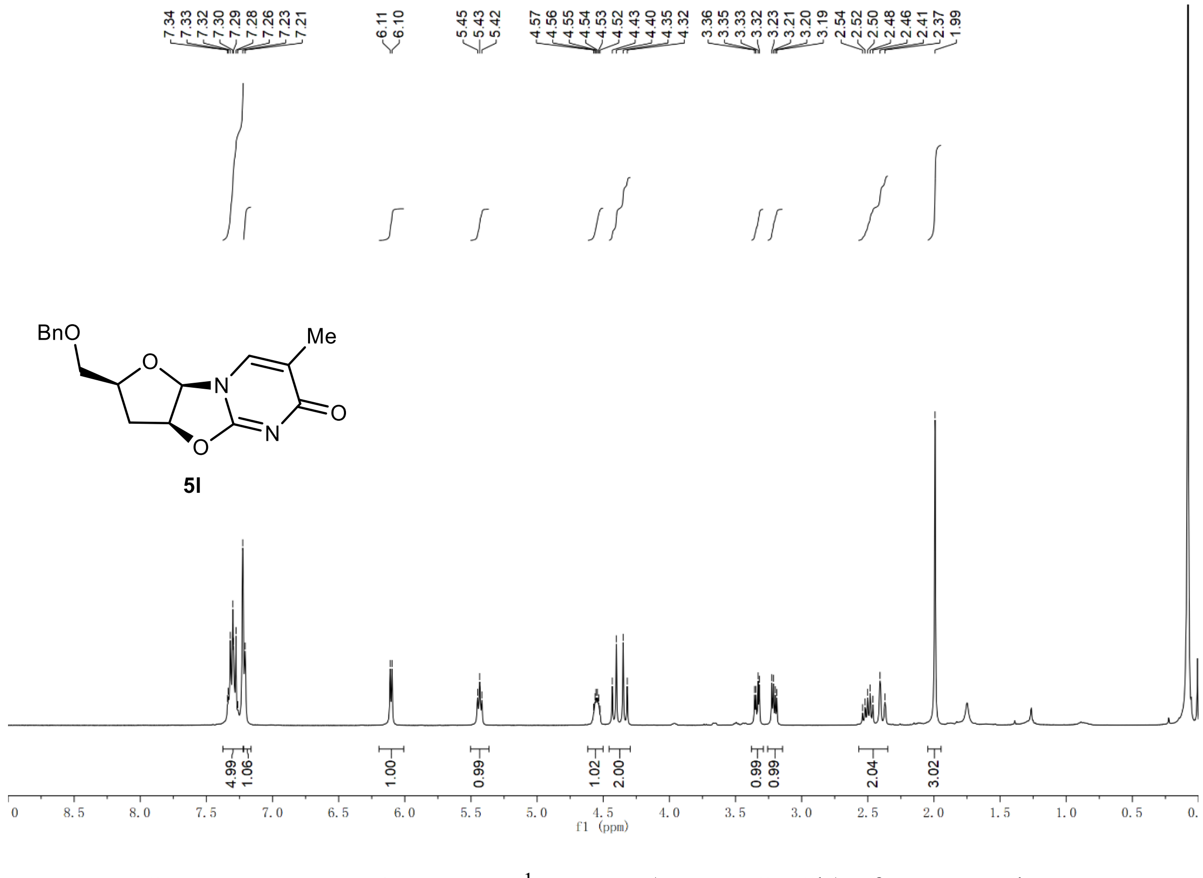

Supplementary Figure 172. ^{1}H NMR (400M, $CDCl_3$) of compound **5l.**

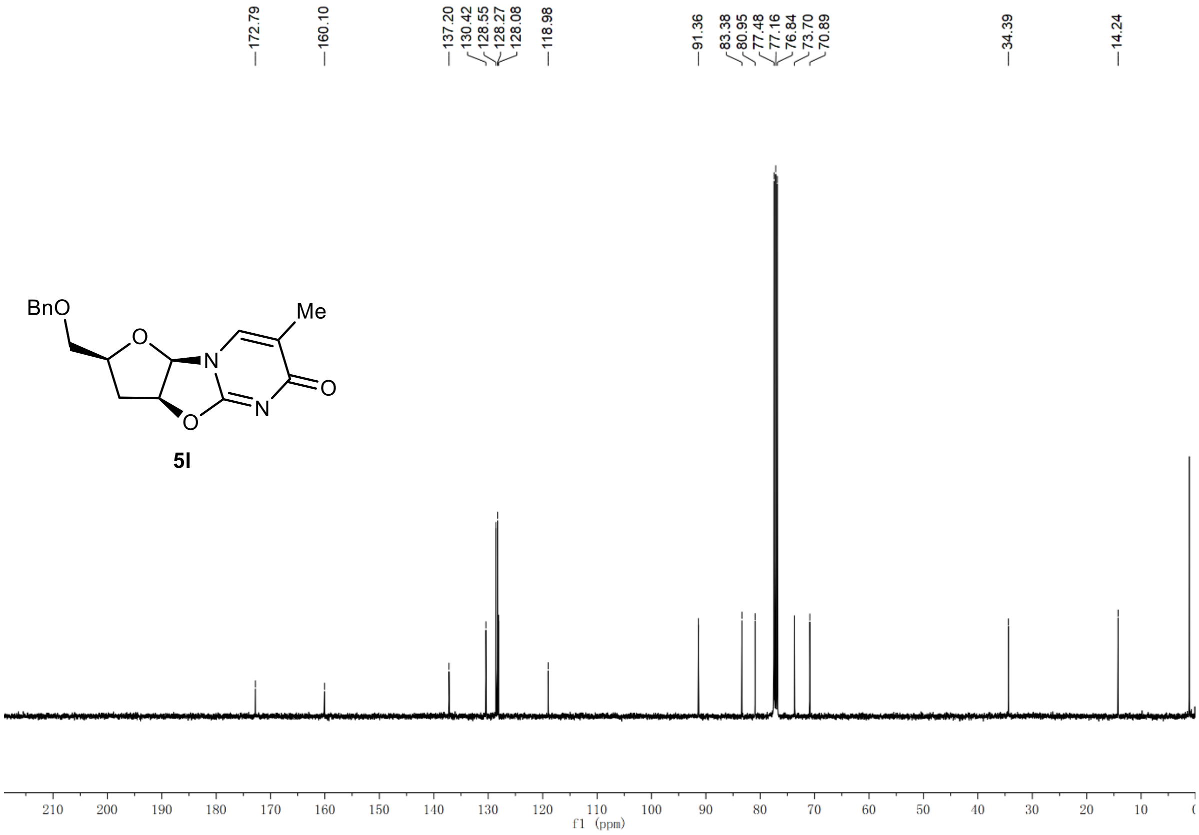

Supplementary Figure 173. ^{13}C NMR (100M, $CDCl_3$) of compound **5l.**

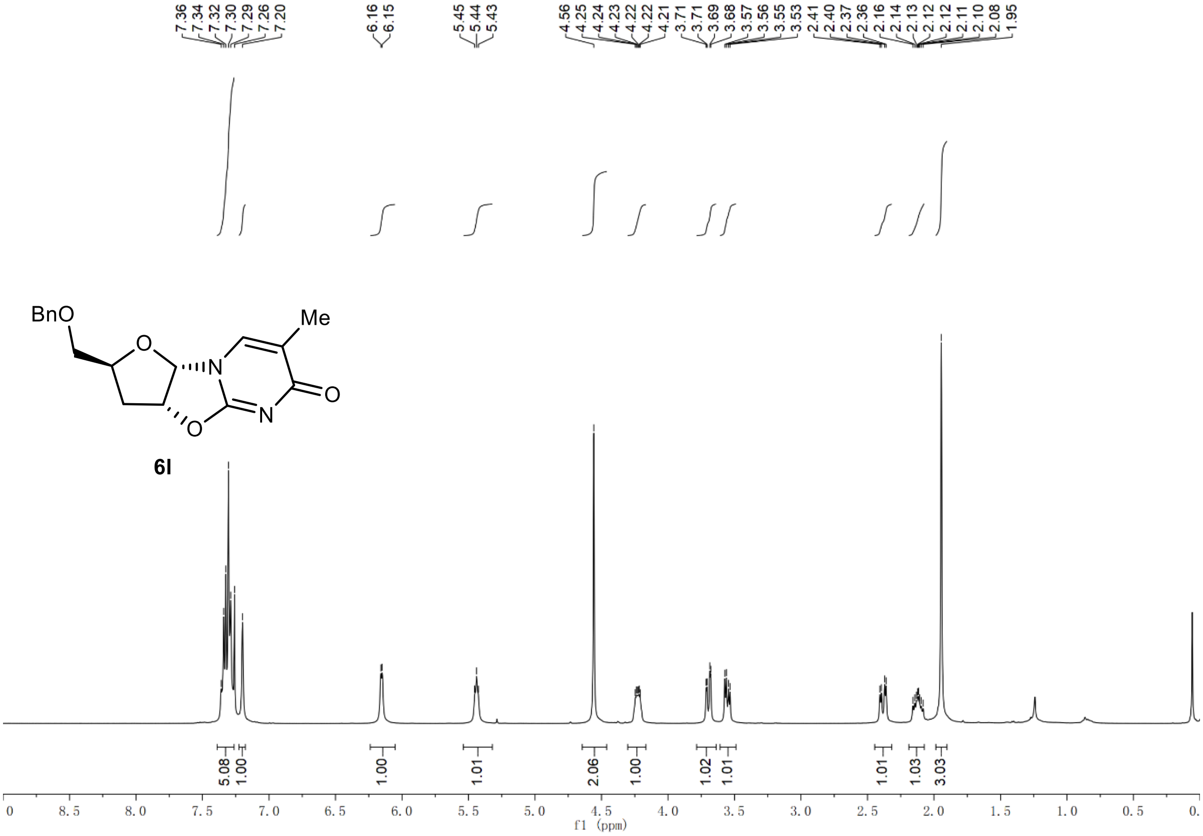

Supplementary Figure 174. ^{1}H NMR (400M, $CDCl_3$) of compound **6l.**

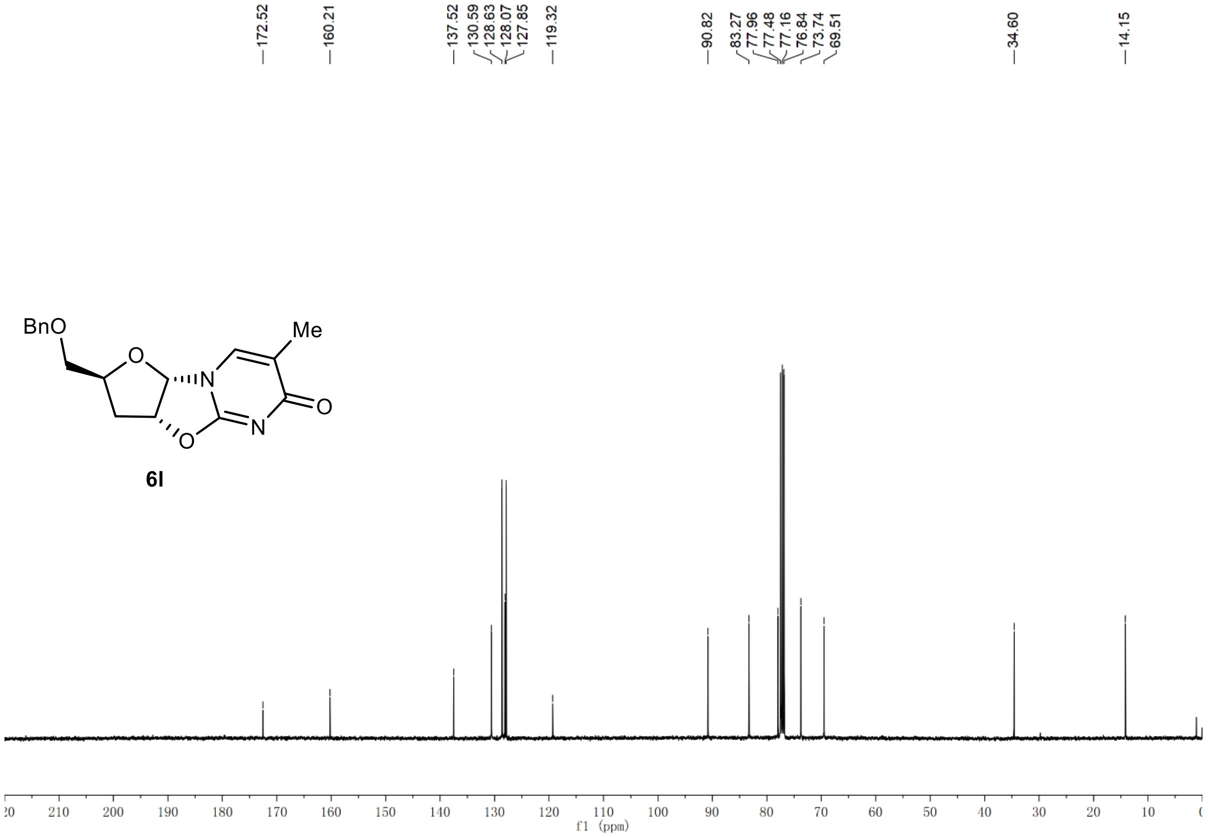

Supplementary Figure 175. ^{13}C NMR (100M, $CDCl_3$) of compound **6l.**

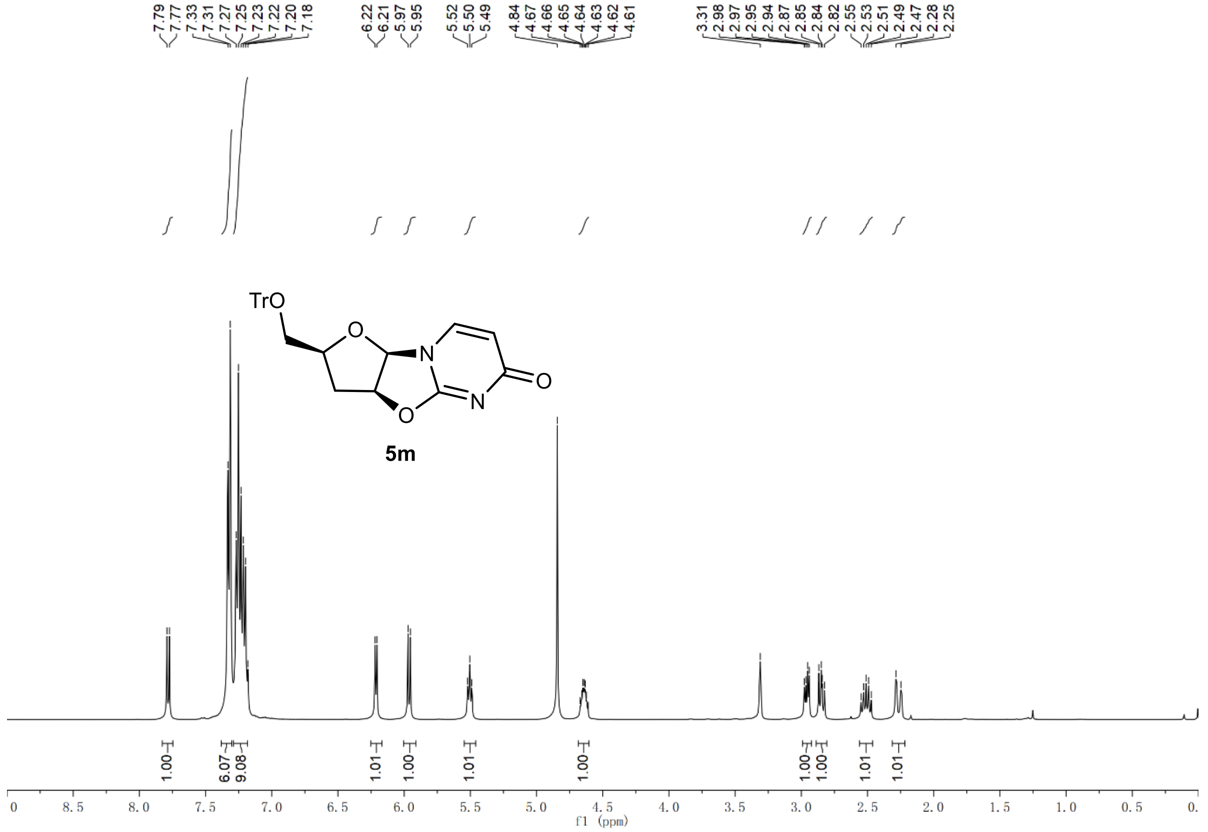

Supplementary Figure 176. ^{1}H NMR (400M, CD_3OD) of compound **5m.**

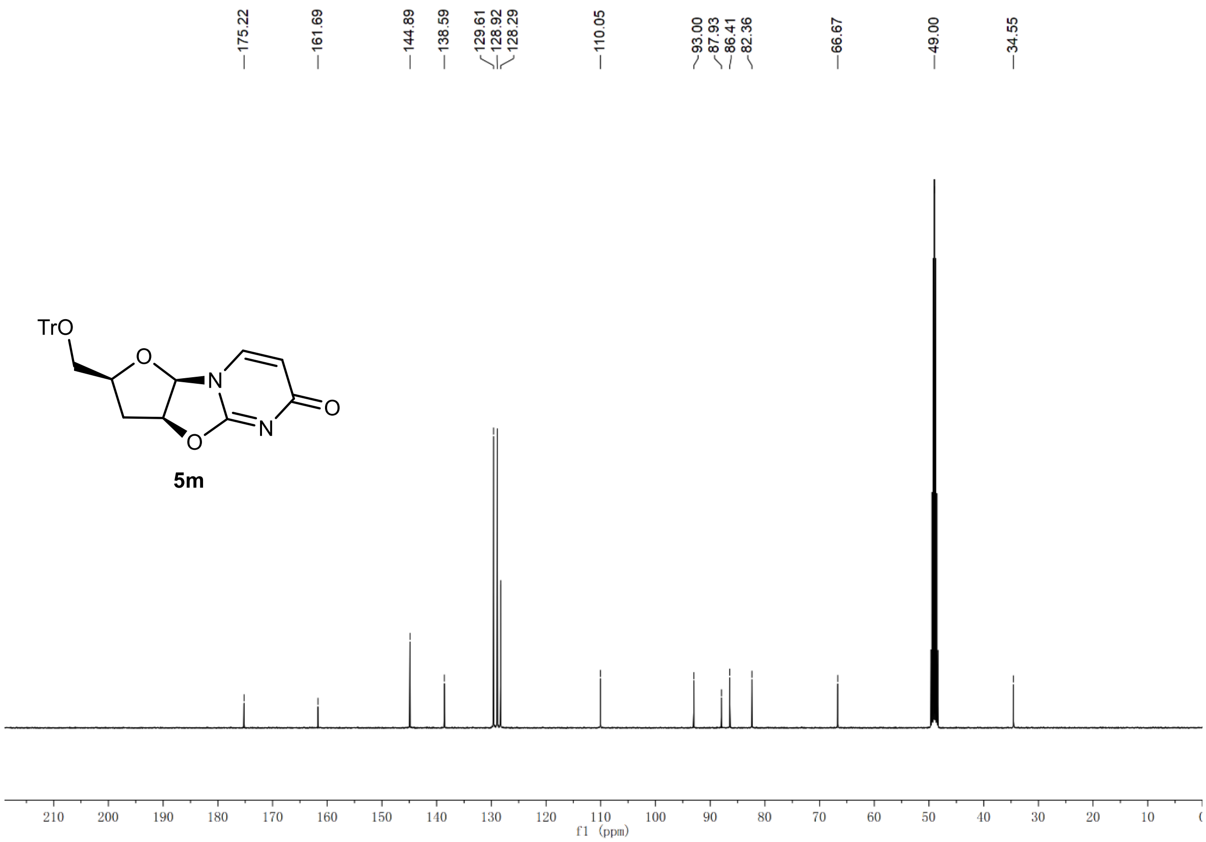

Supplementary Figure 177. ^{13}C NMR (100M, CD_3OD) of compound **5m.**

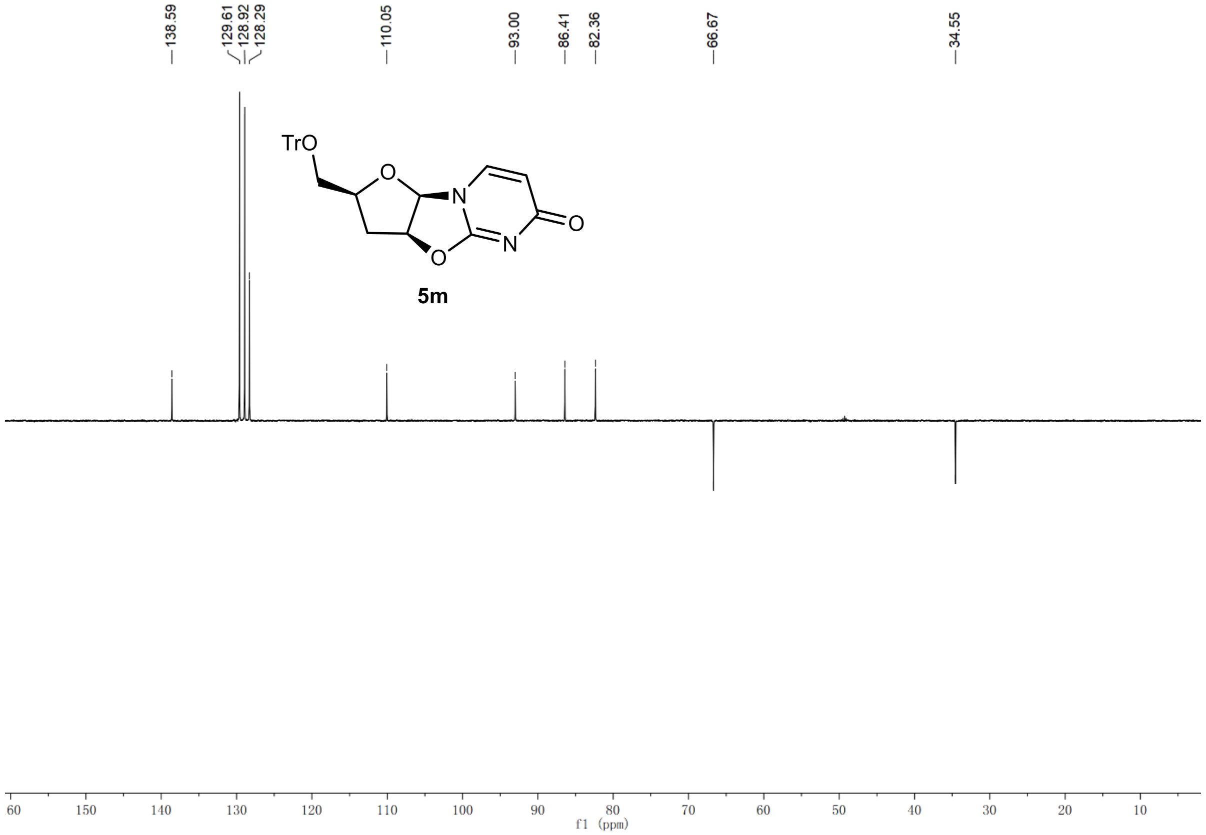

Supplementary Figure 178. DEPT 135° (100M, CD_3OD) of compound **5m.**

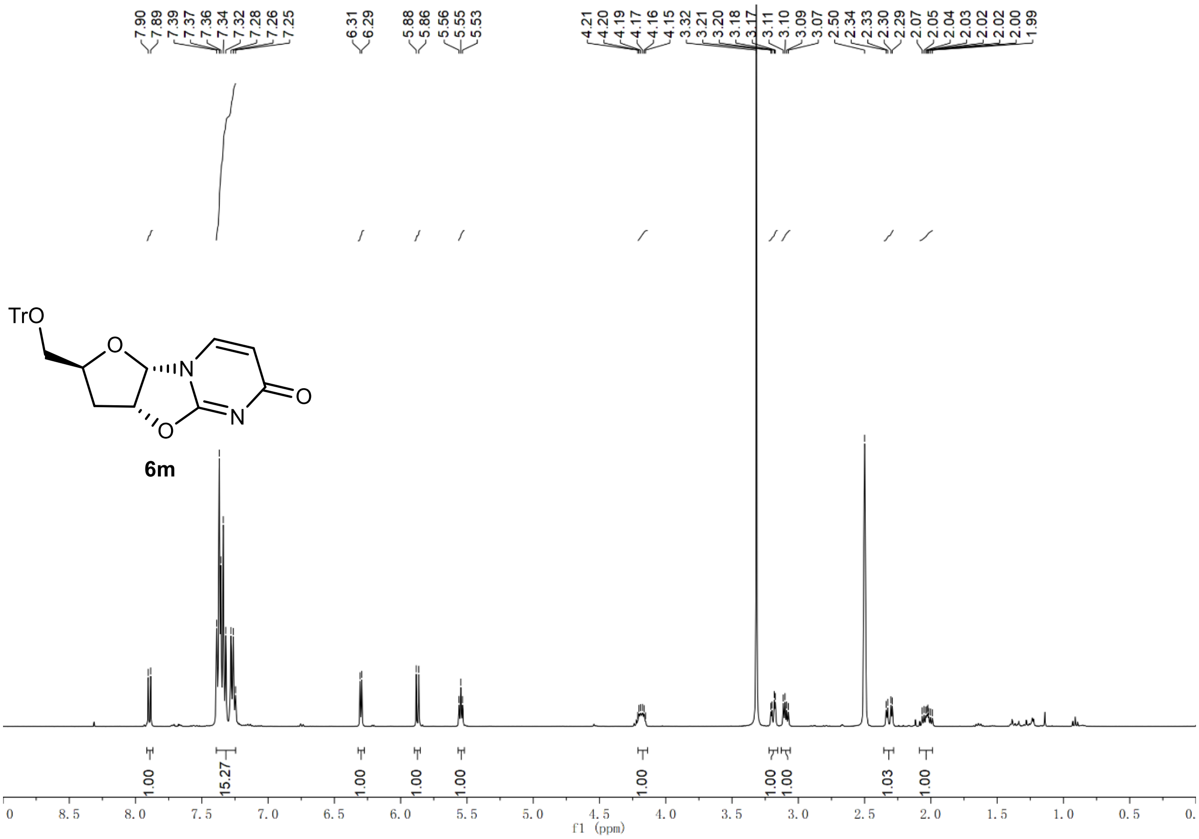

Supplementary Figure 179. ^{1}H NMR (400M, $(CD_3)_2SO$) of compound **6m.**

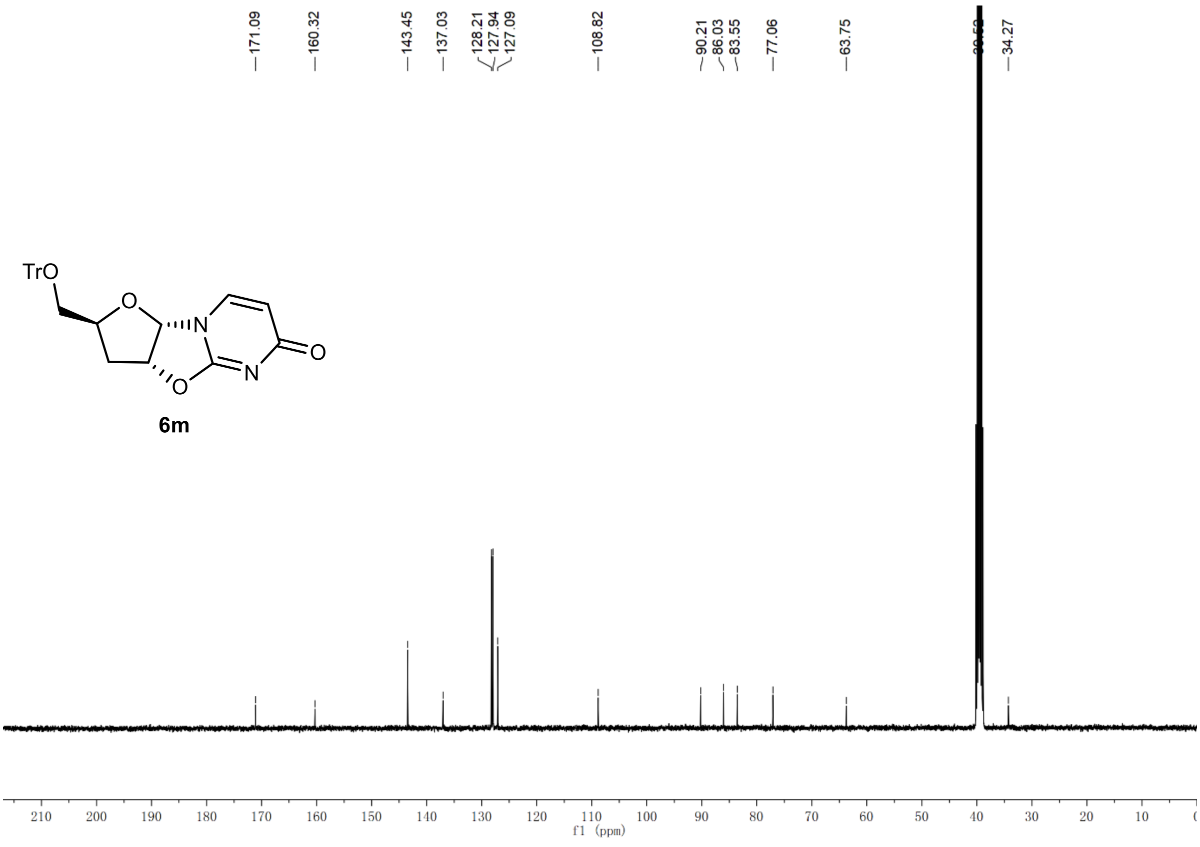

Supplementary Figure 180. ^{13}C NMR (100M, $(CD_3)_2SO$) of compound **6m.**

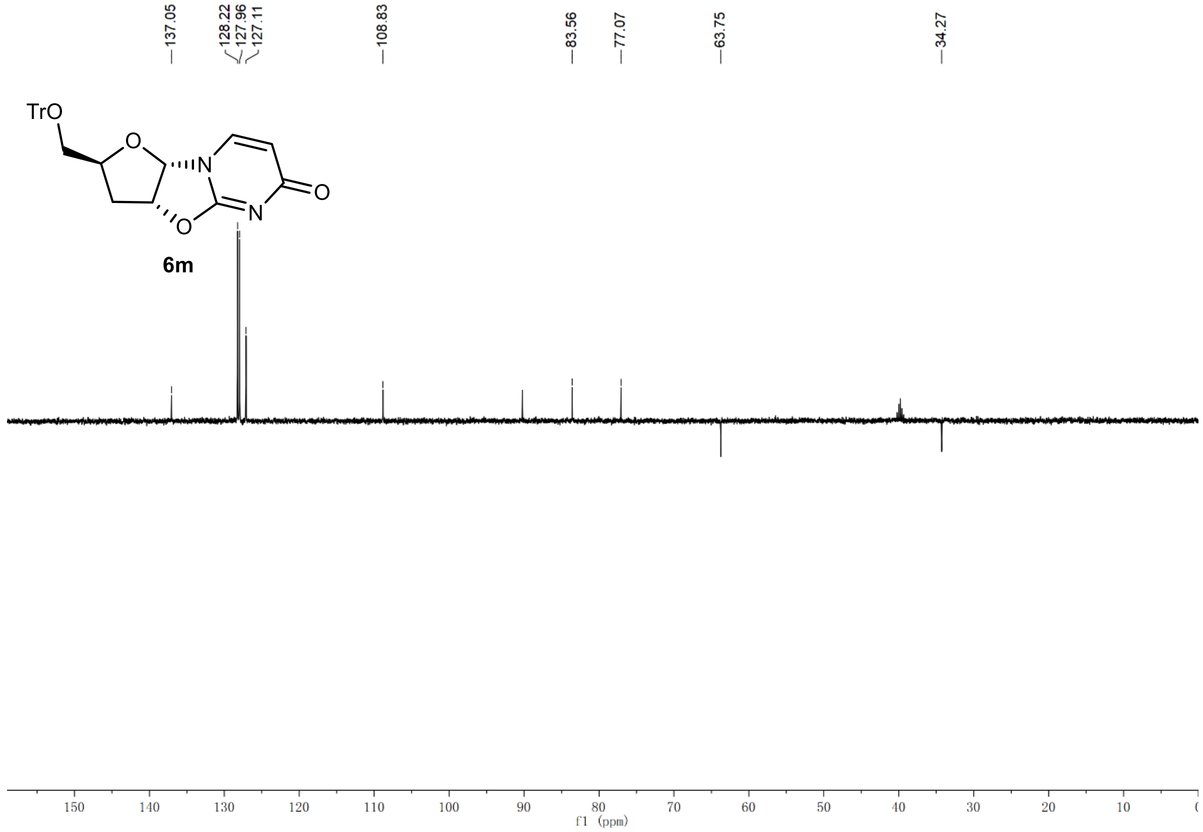

Supplementary Figure 181. DEPT 135° (100M, $(CD_3)_2SO$) of compound **6m.**

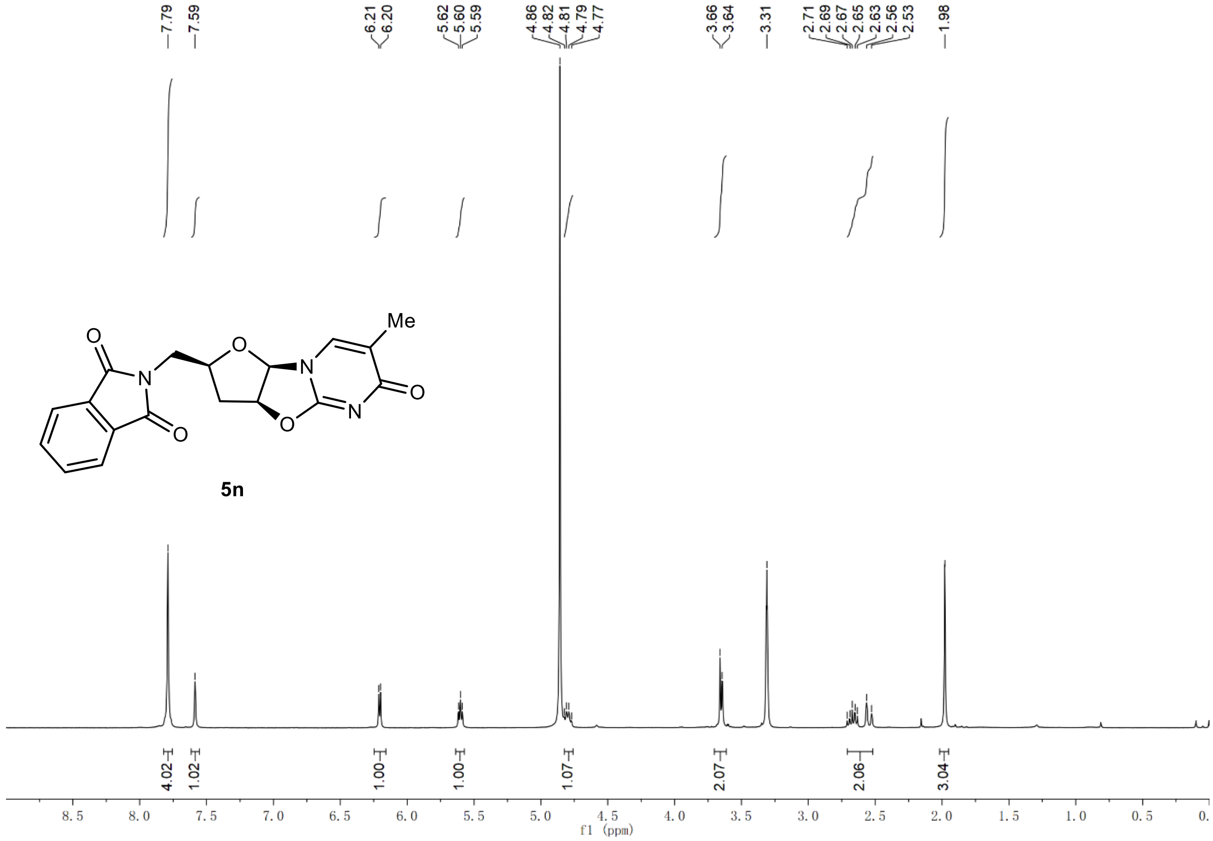

Supplementary Figure 182. ^{1}H NMR (400M, CD_3OD) of compound **5n.**

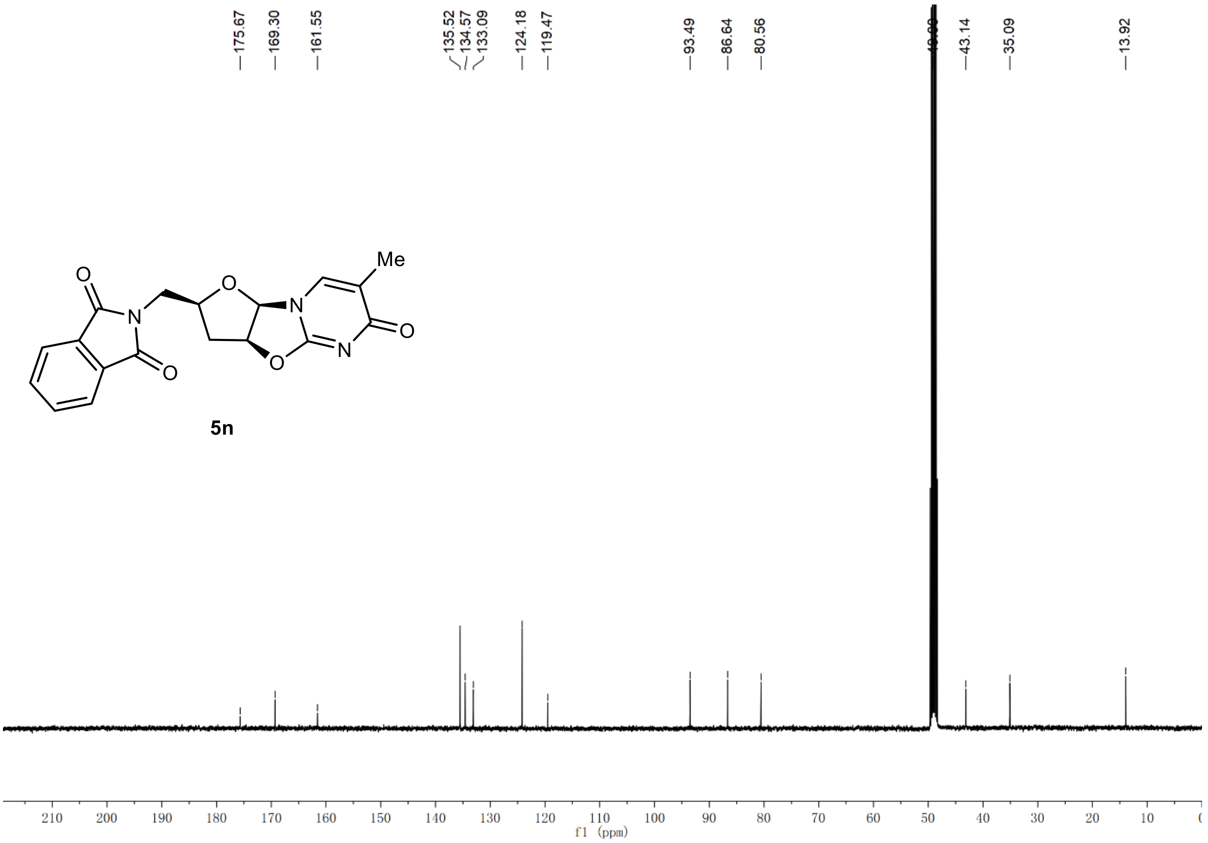

Supplementary Figure 183. ^{13}C NMR (100M, CD_3OD) of compound **5n.**

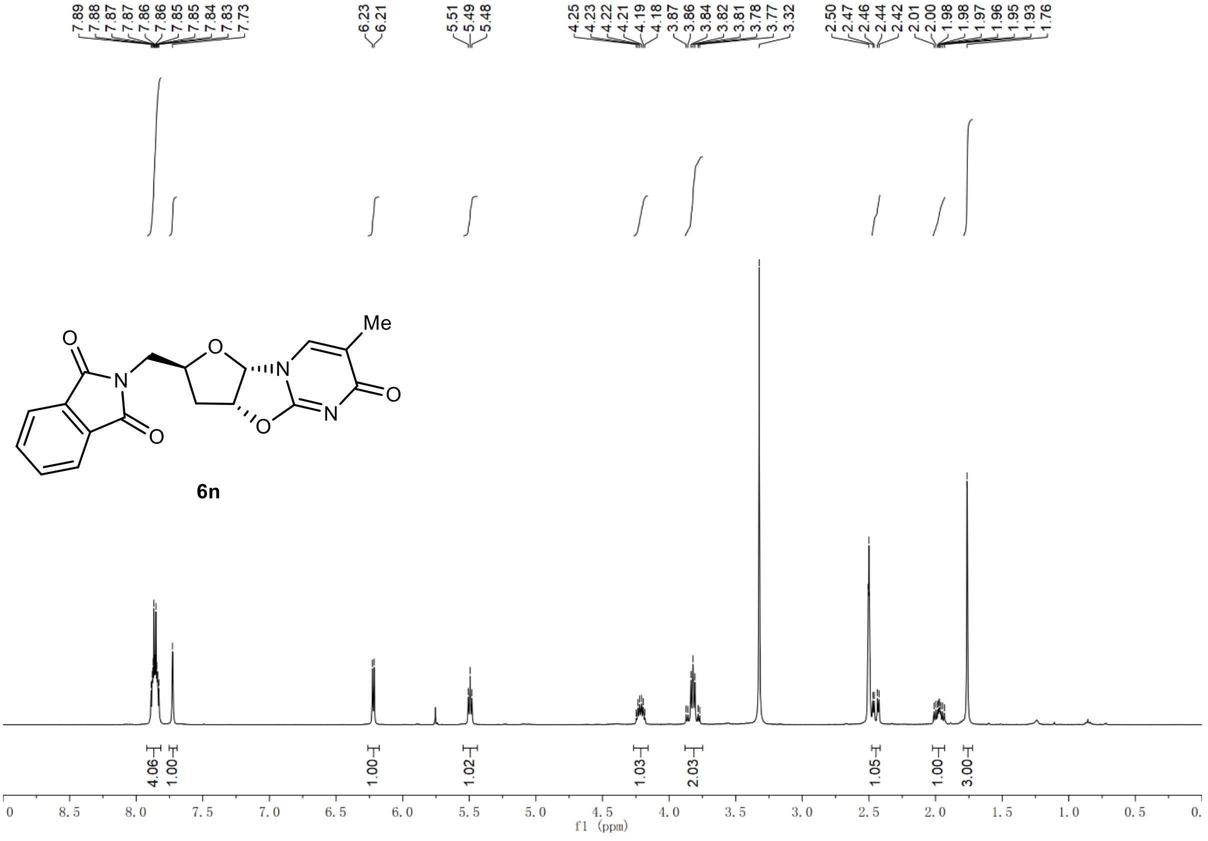

Supplementary Figure 184. ^{1}H NMR (400M, $(CD_3)_2SO$) of compound **6n.**

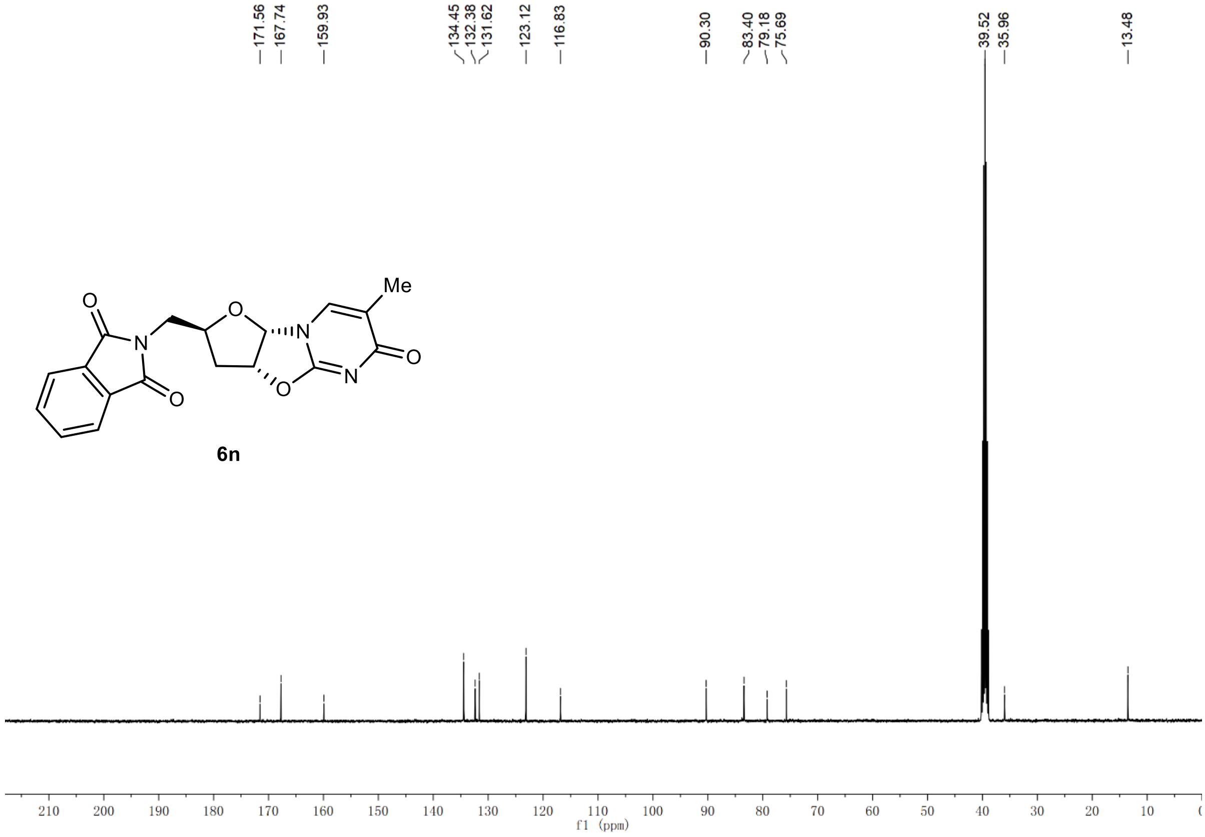

Supplementary Figure 185. ^{13}C NMR (100M, $(CD_3)_2SO$) of compound **6n.**

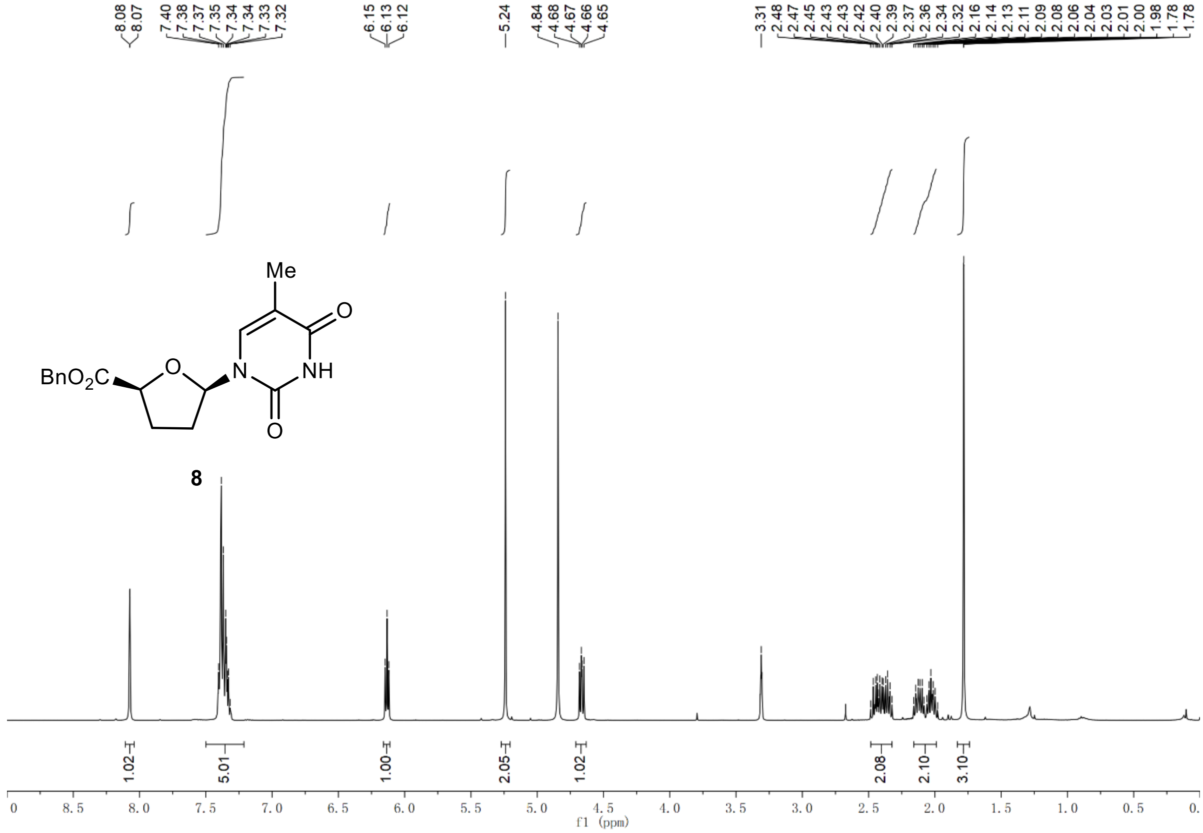

Supplementary Figure 186. ^{1}H NMR (400M, CD_3OD) of compound **8.**

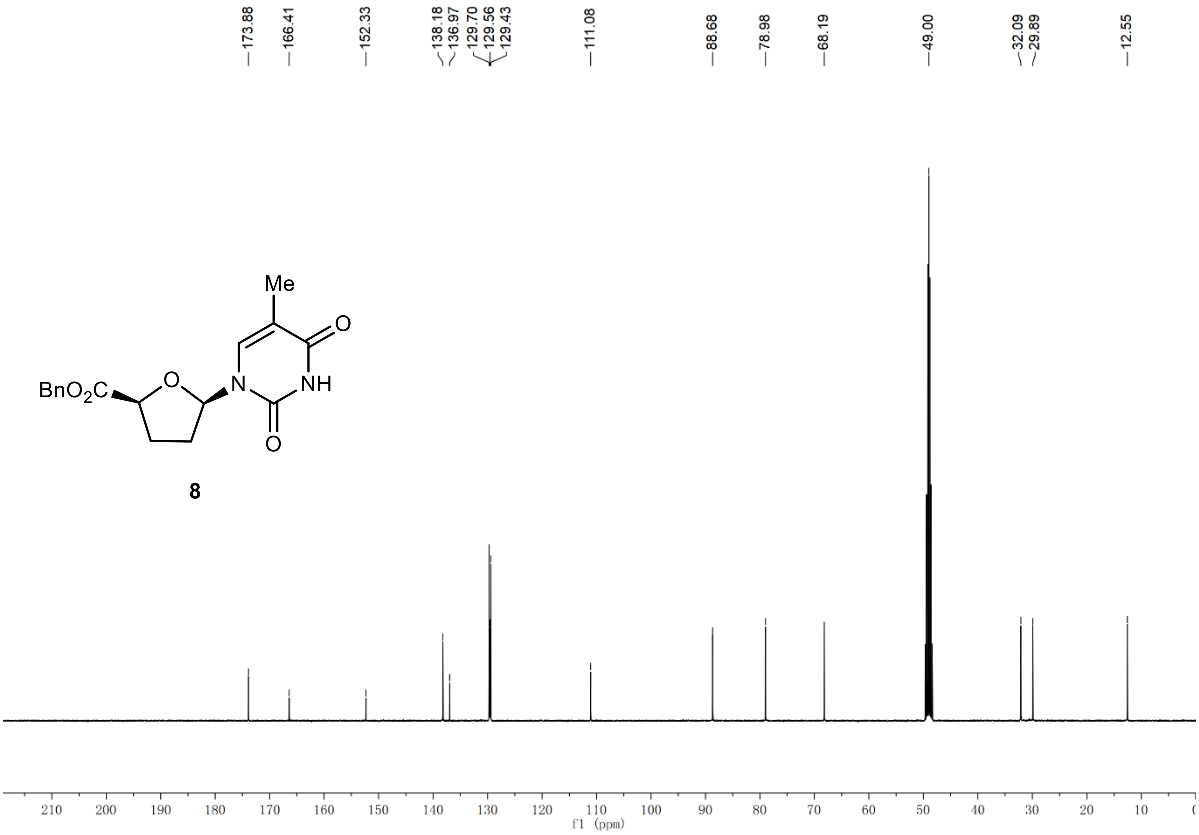

Supplementary Figure 187. ^{13}C NMR (100M, CD_3OD) of compound **8.**

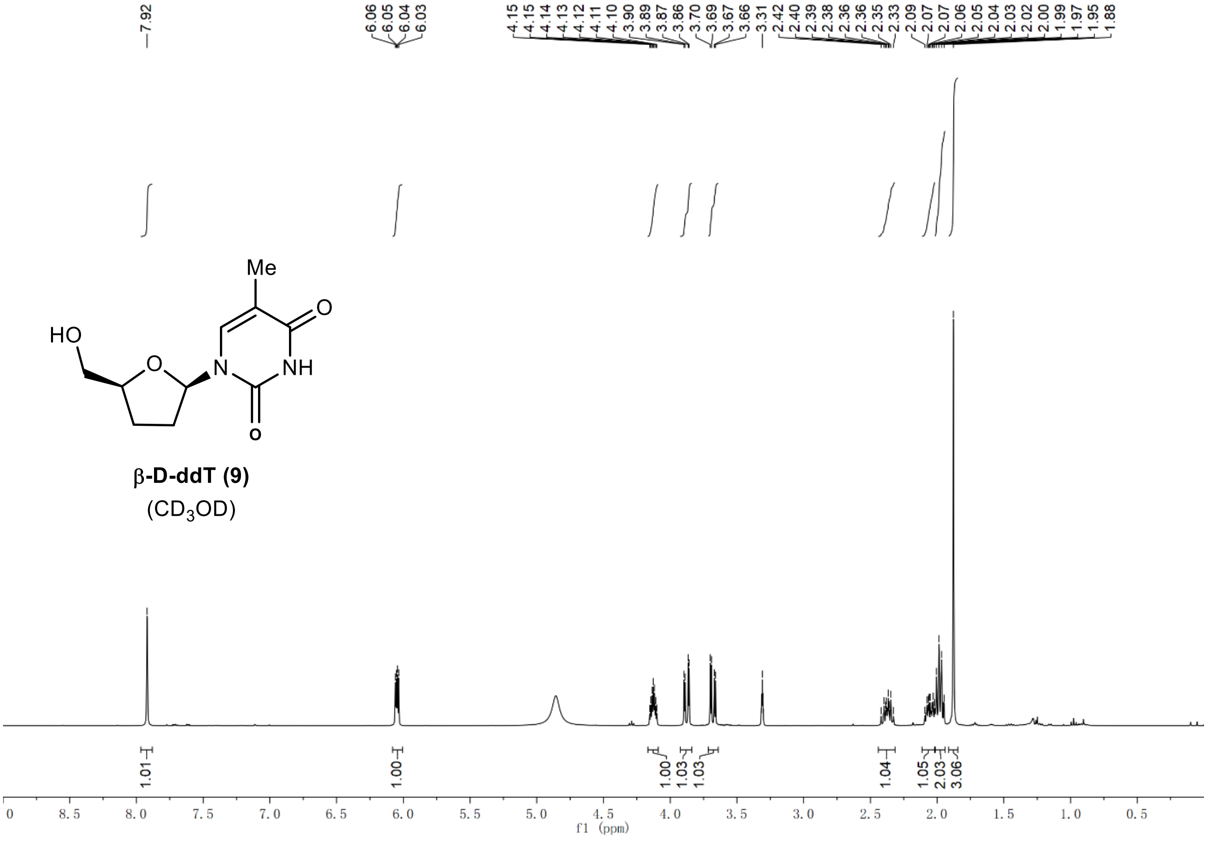

Supplementary Figure 188. ^{1}H NMR (400M, CD_3OD) of compound **9.**

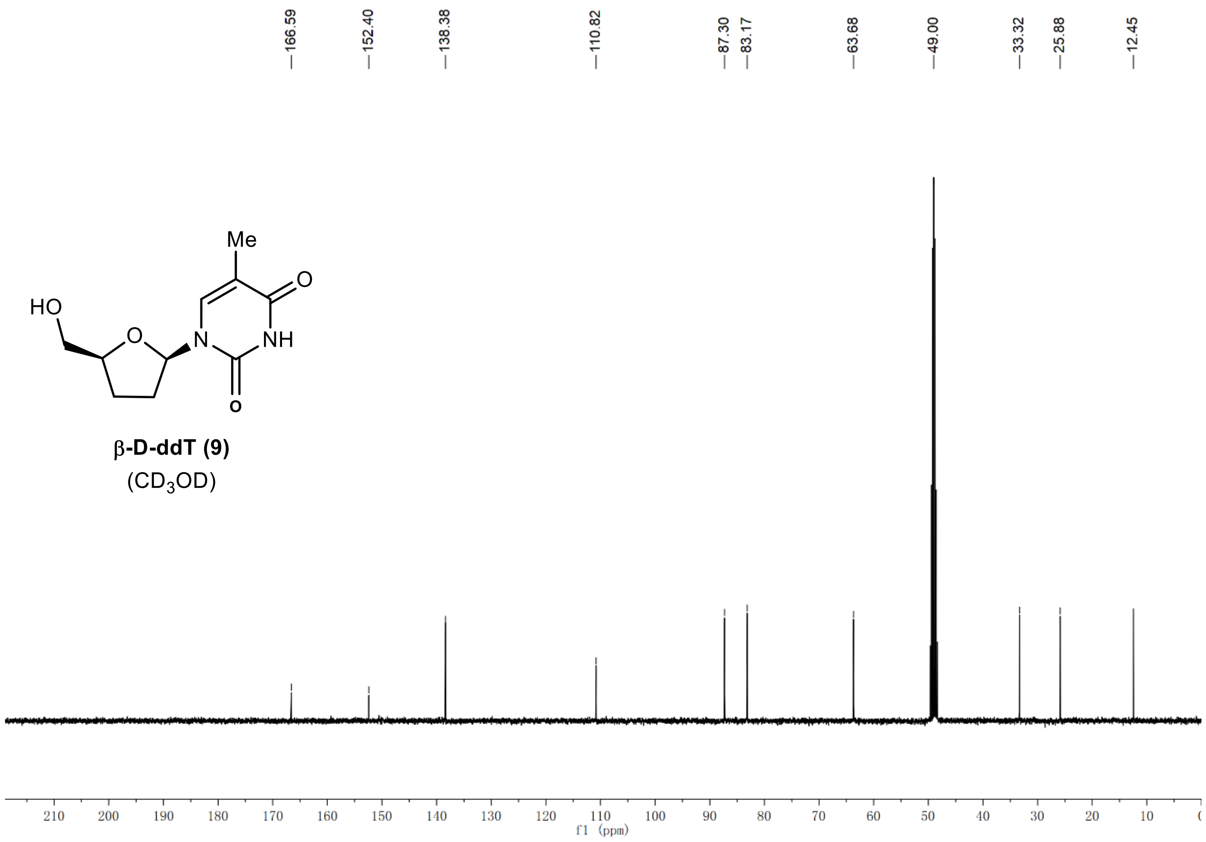

Supplementary Figure 189. ^{13}C NMR (100M, CD_3OD) of compound **9.**

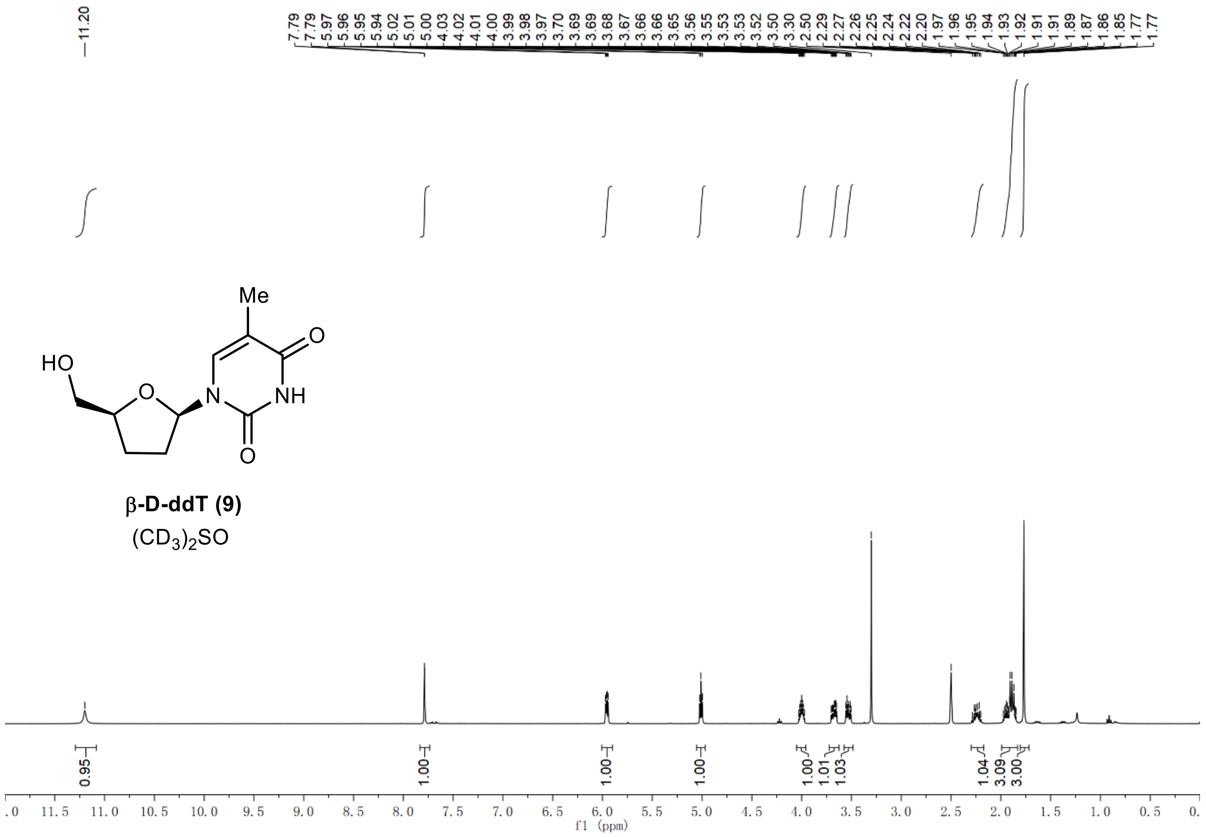

Supplementary Figure 190. ^{1}H NMR (400M, $(CD_3)_2SO$) of compound **9.**

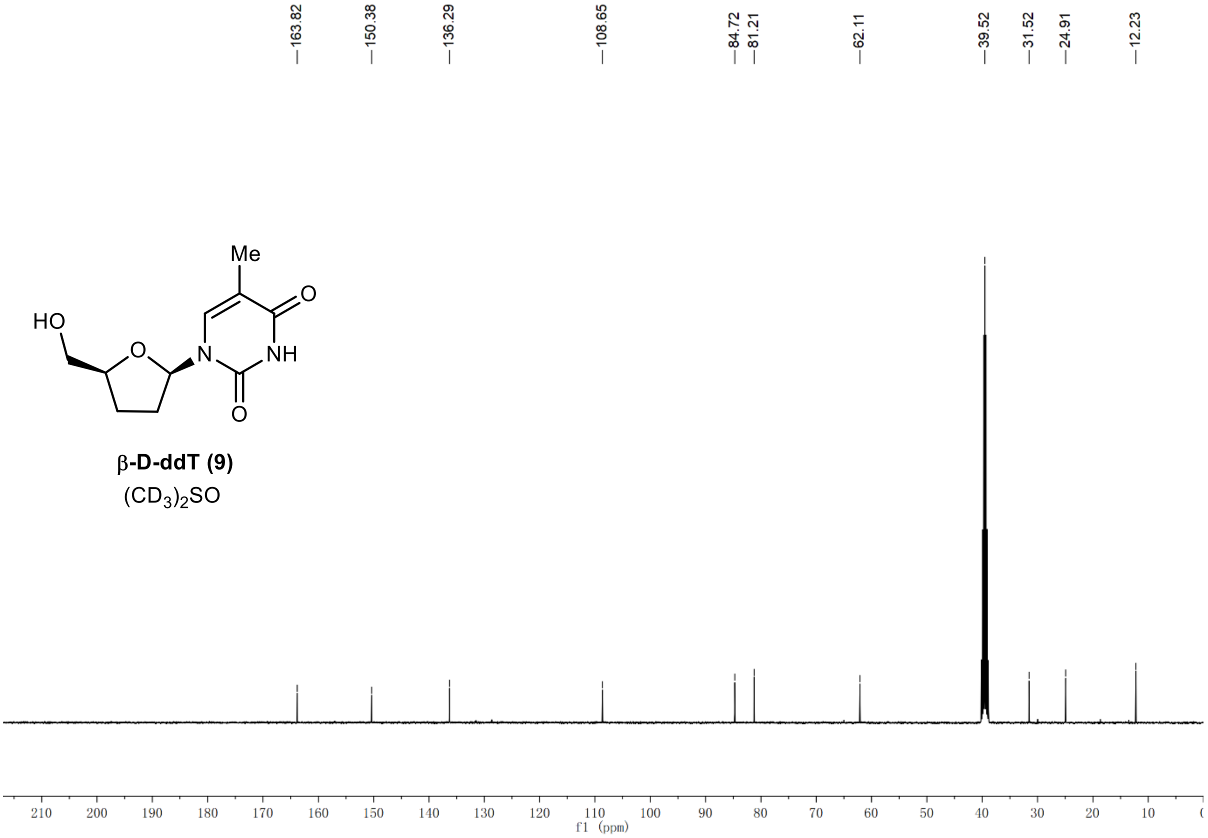

Supplementary Figure 191. ^{13}C NMR (100M, $(CD_3)_2SO$) of compound **9.**

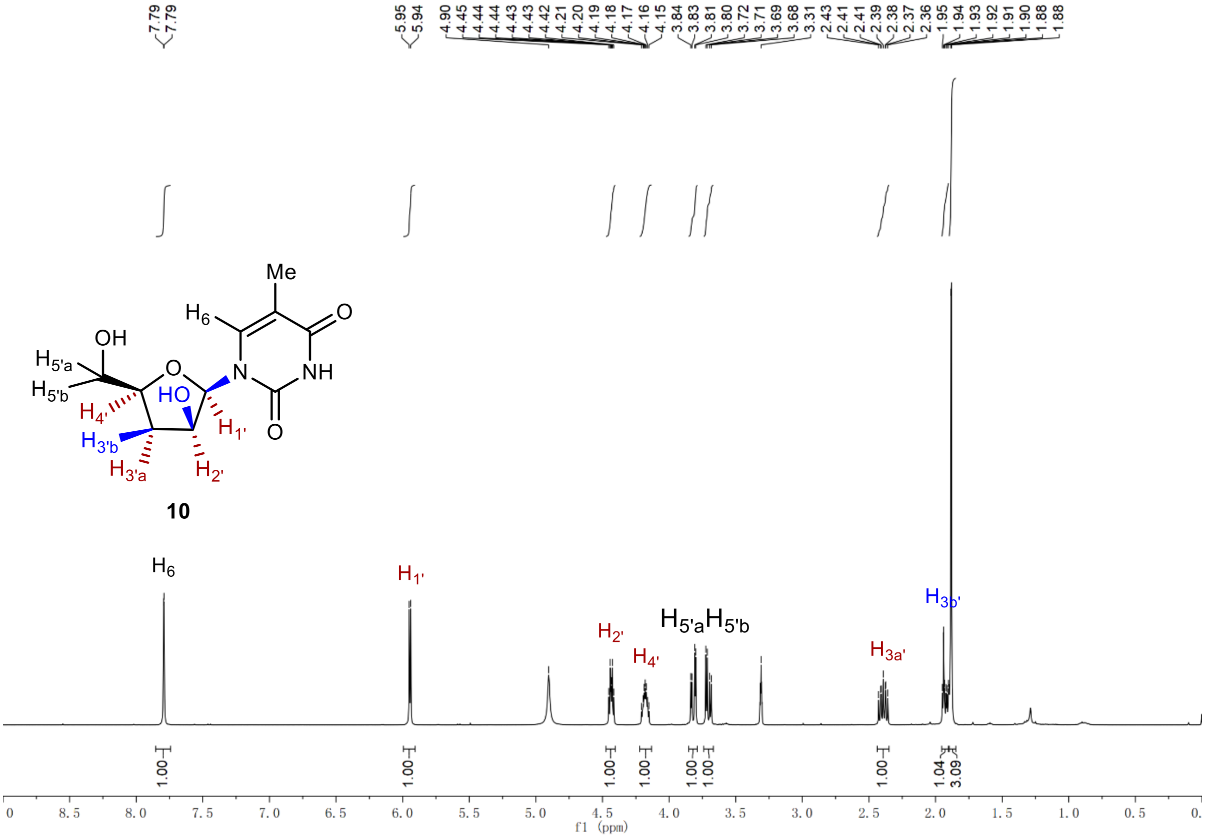

Supplementary Figure 192. ^{1}H NMR (400M, CD_3OD) of compound **10.**

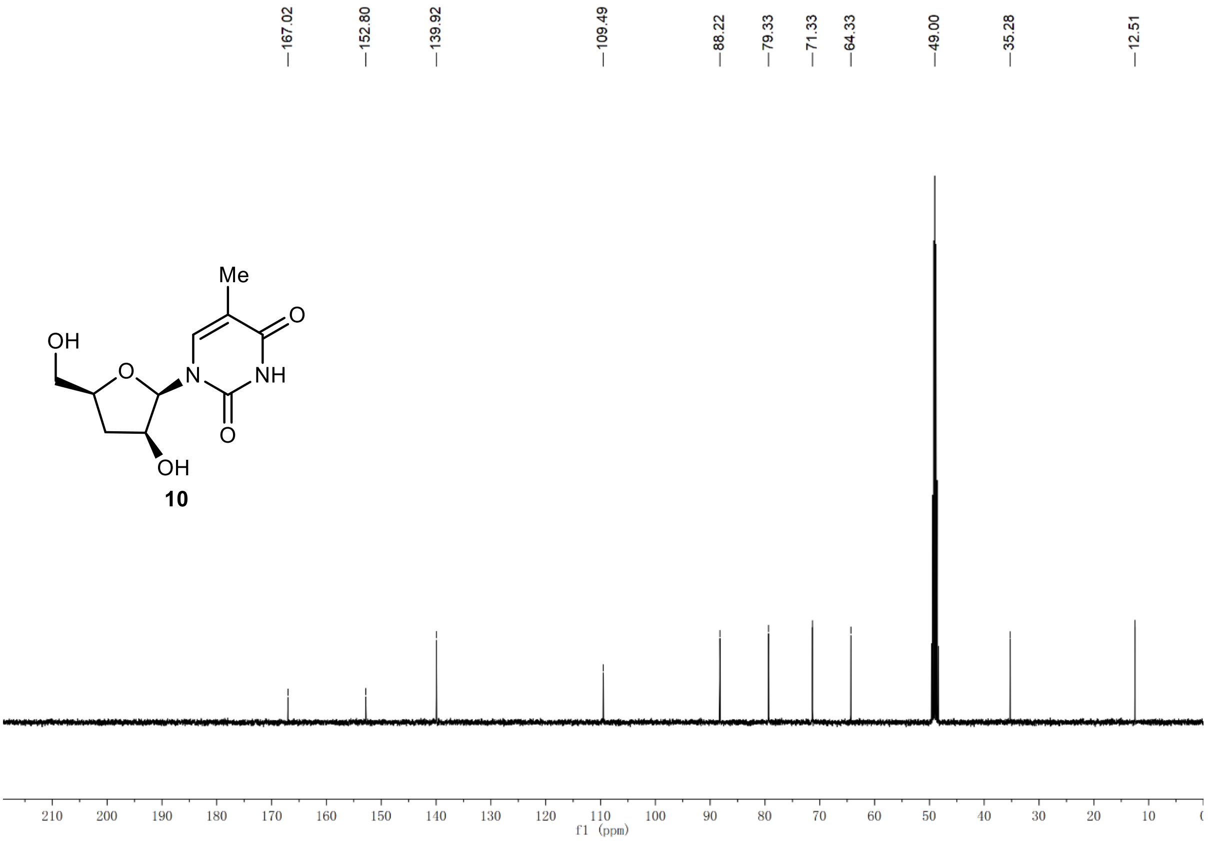

Supplementary Figure 193. ^{13}C NMR (100M, CD_3OD) of compound **10.**

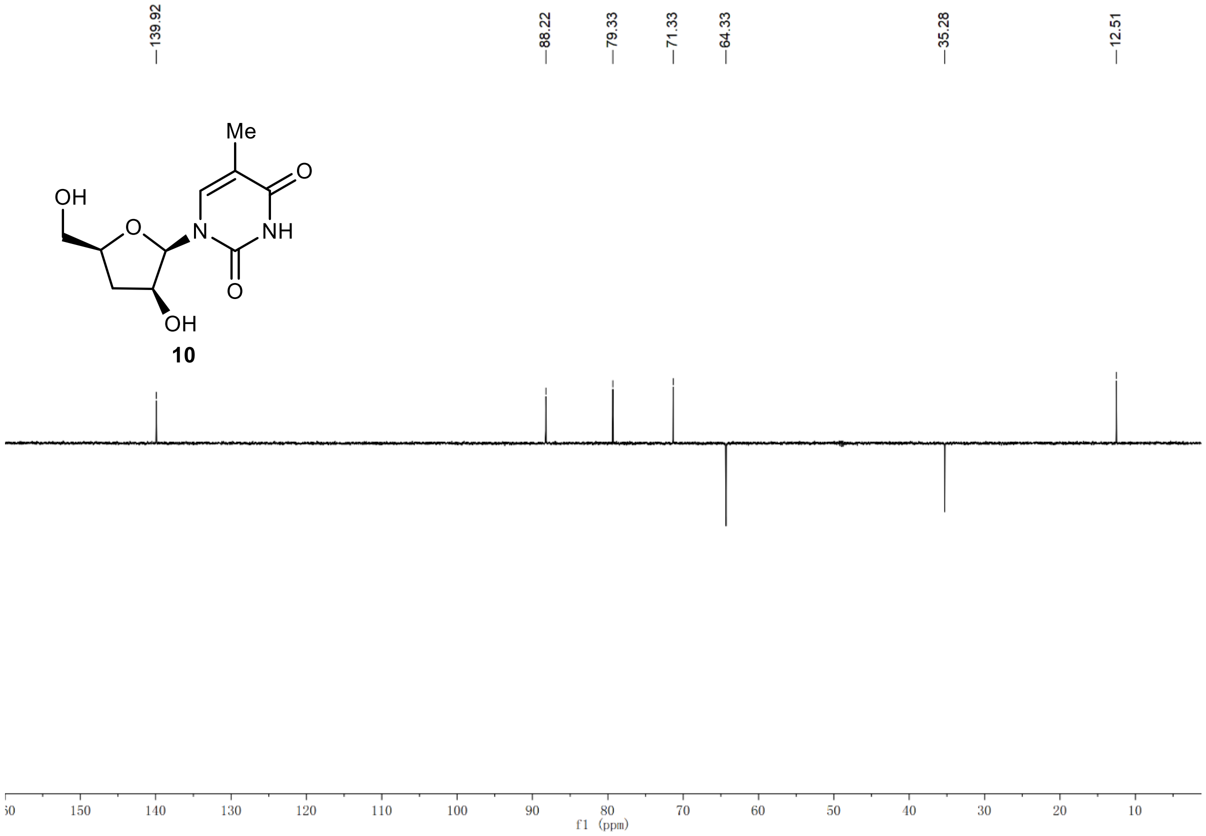

Supplementary Figure 194. DEPT 135° (100M, CD_3OD) of compound **10.**

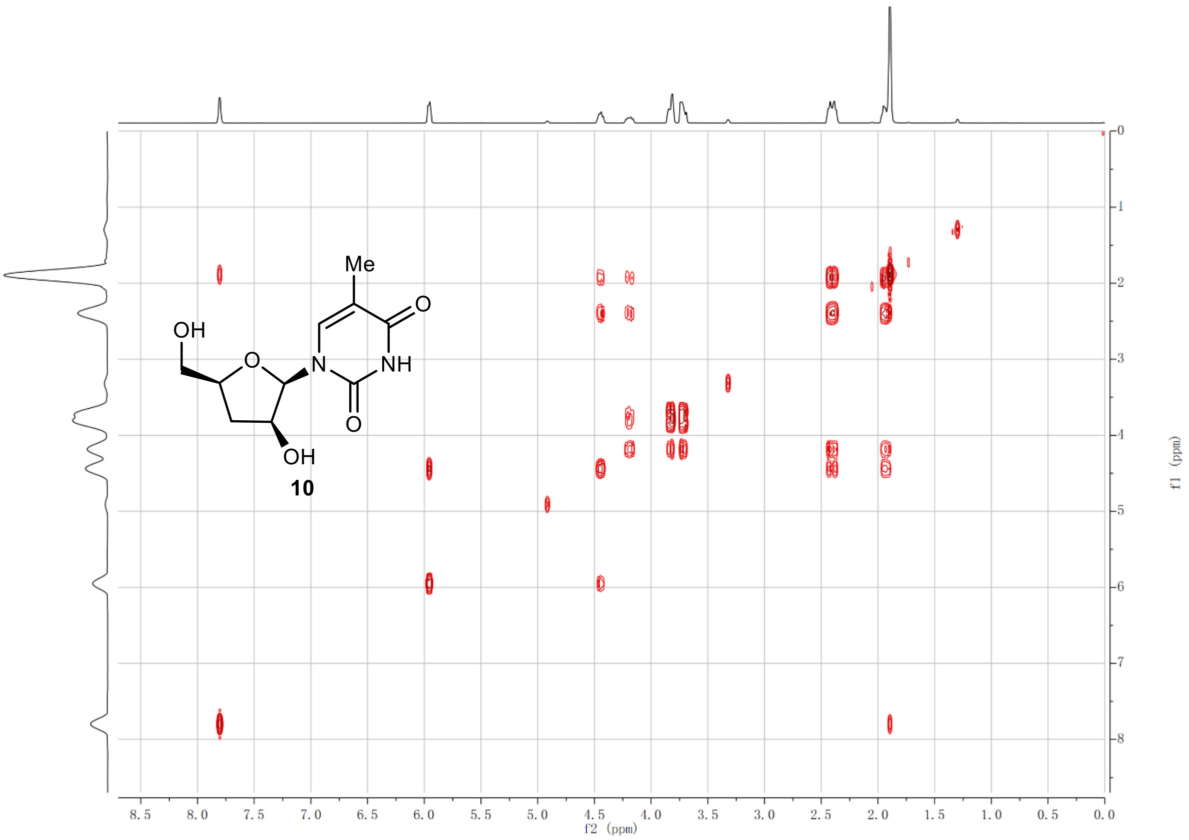

Supplementary Figure 195. ^{1}H-^{1}H COSY (400M, CD_3OD) of compound **10.**

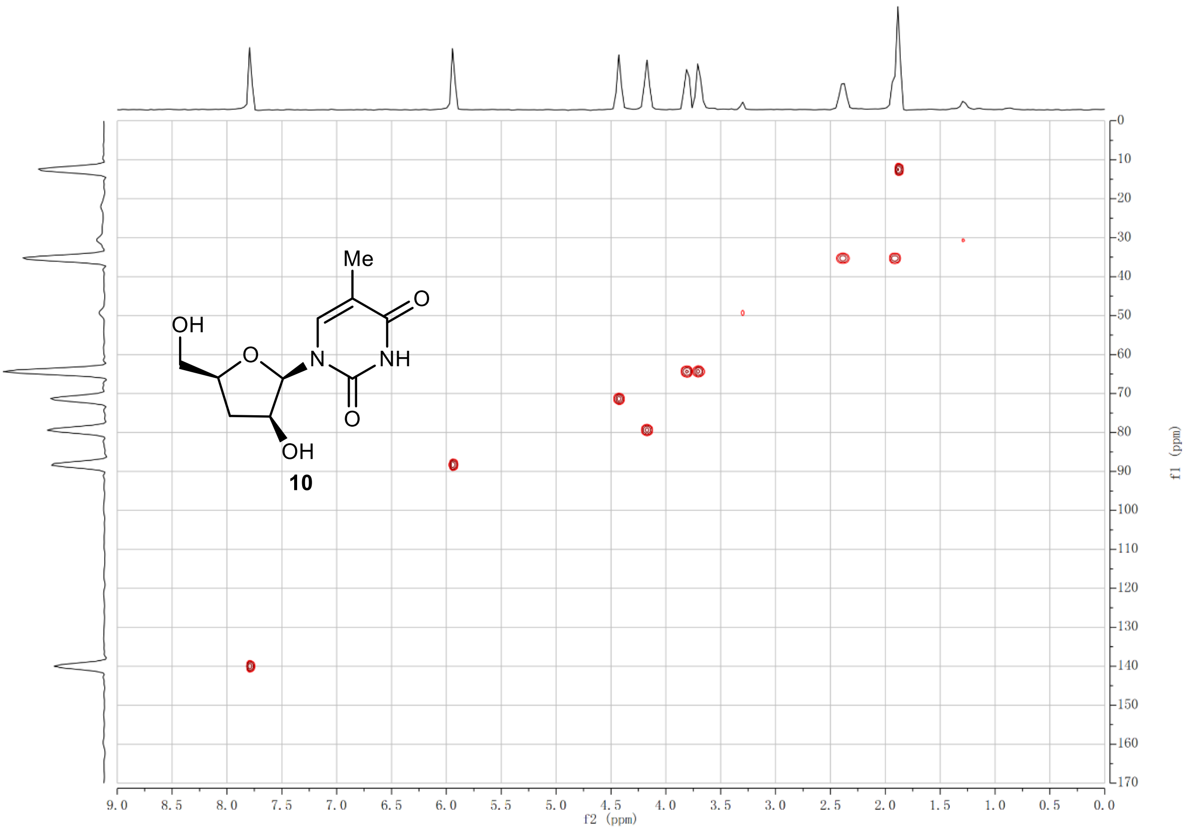

Supplementary Figure 196. ^{1}H-^{13}C HSQC (400M, CD_3OD) of compound **10.**

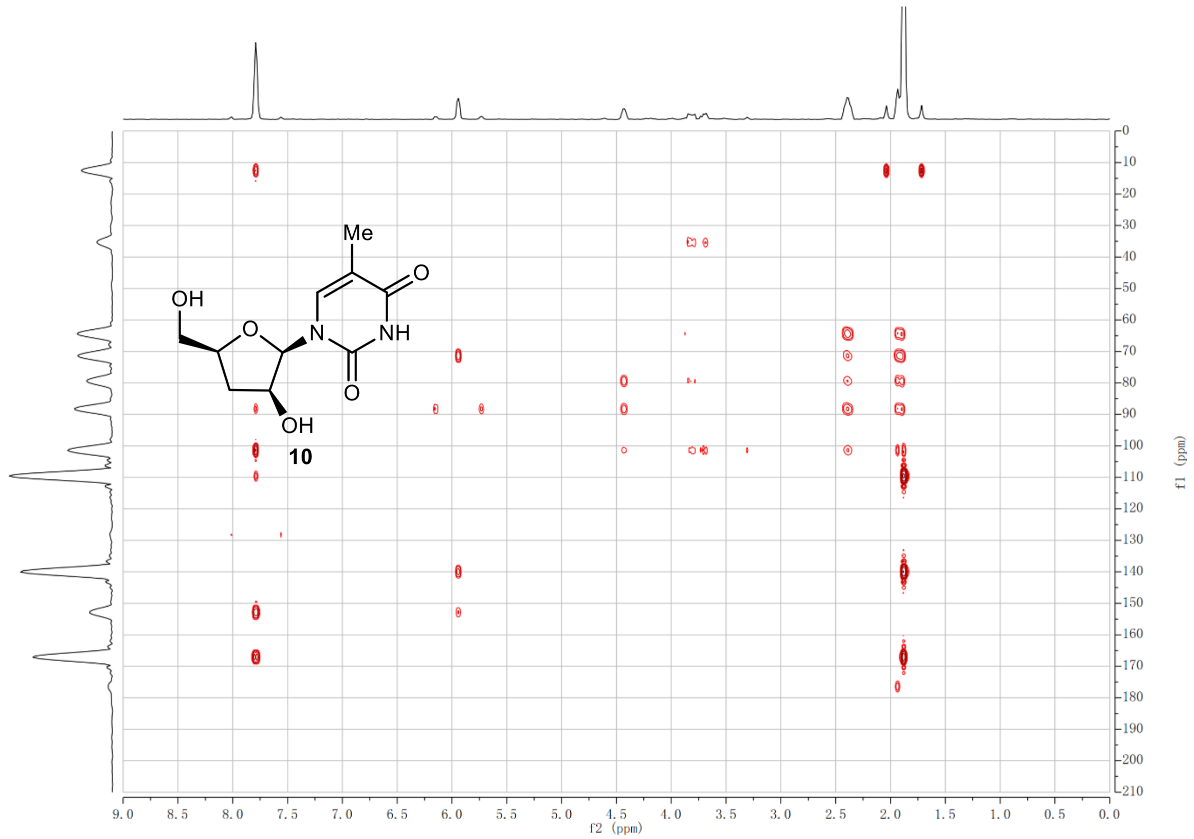

Supplementary Figure 197. ^{1}H-^{13}C HMBC (400M, CD_3OD) of compound **10.**

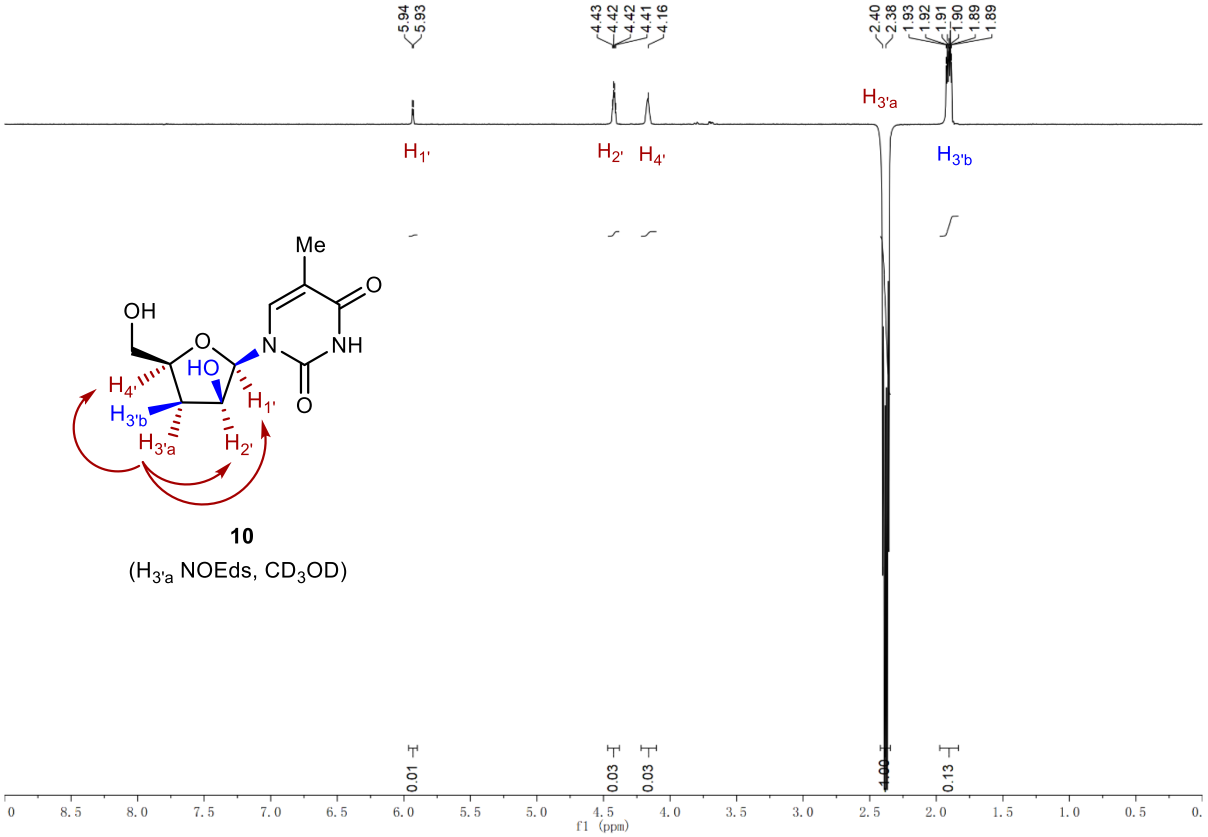

Supplementary Figure 198. $H_{3'a}$ NOEds (600M, CD_3OD) of compound **10.**

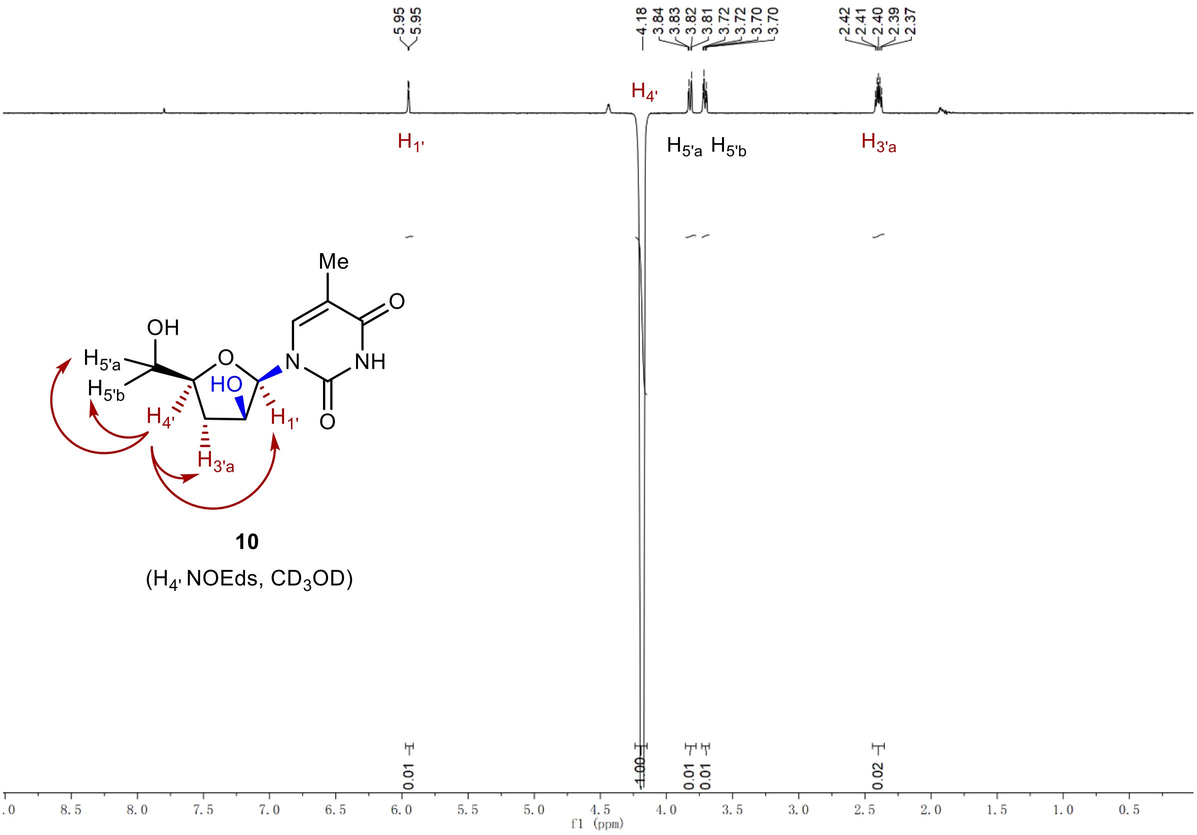

Supplementary Figure 199. H4' NOEds (600M, CD_3OD) of compound **10.**

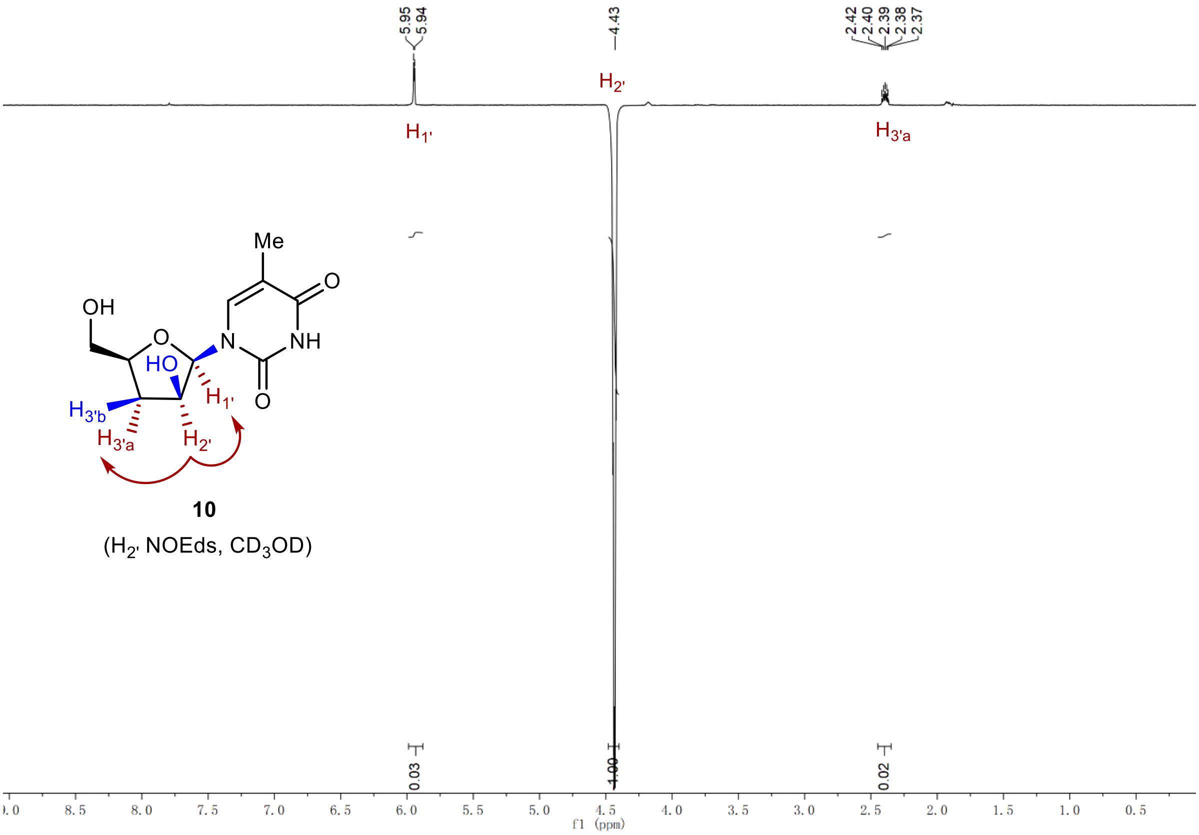

Supplementary Figure 200. H2' NOEds (600M, CD_3OD) of compound **10.**

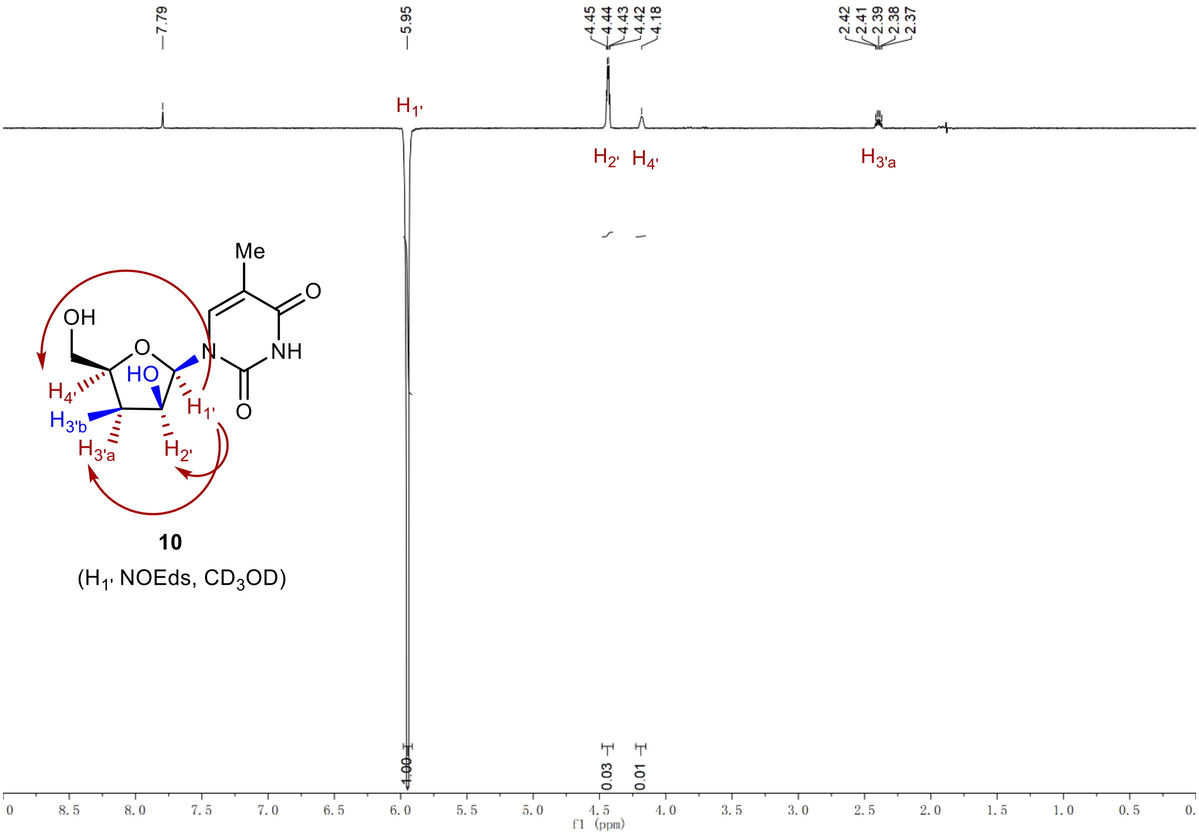

Supplementary Figure 201. $H_{1'}$ NOEds (600M, CD_3OD) of compound **10.**

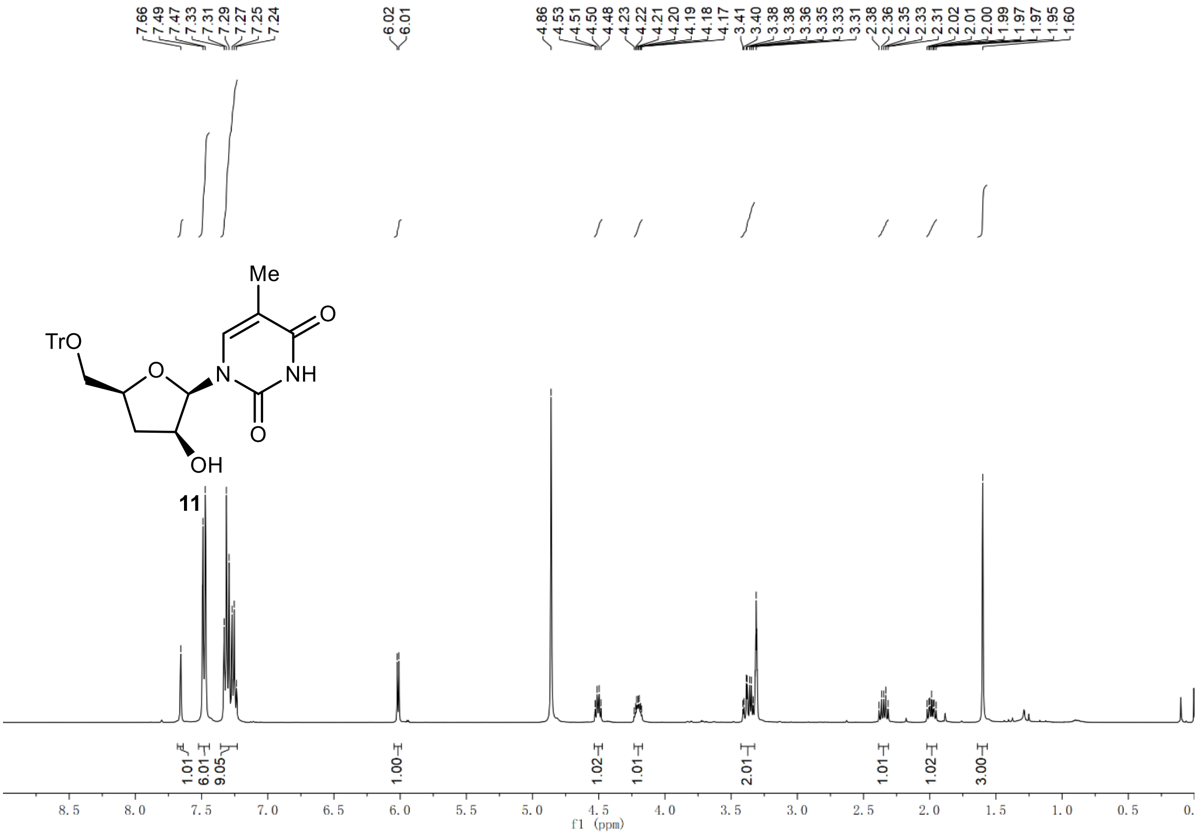

Supplementary Figure 202. ^{1}H NMR (400M, CD_3OD) of compound **11.**

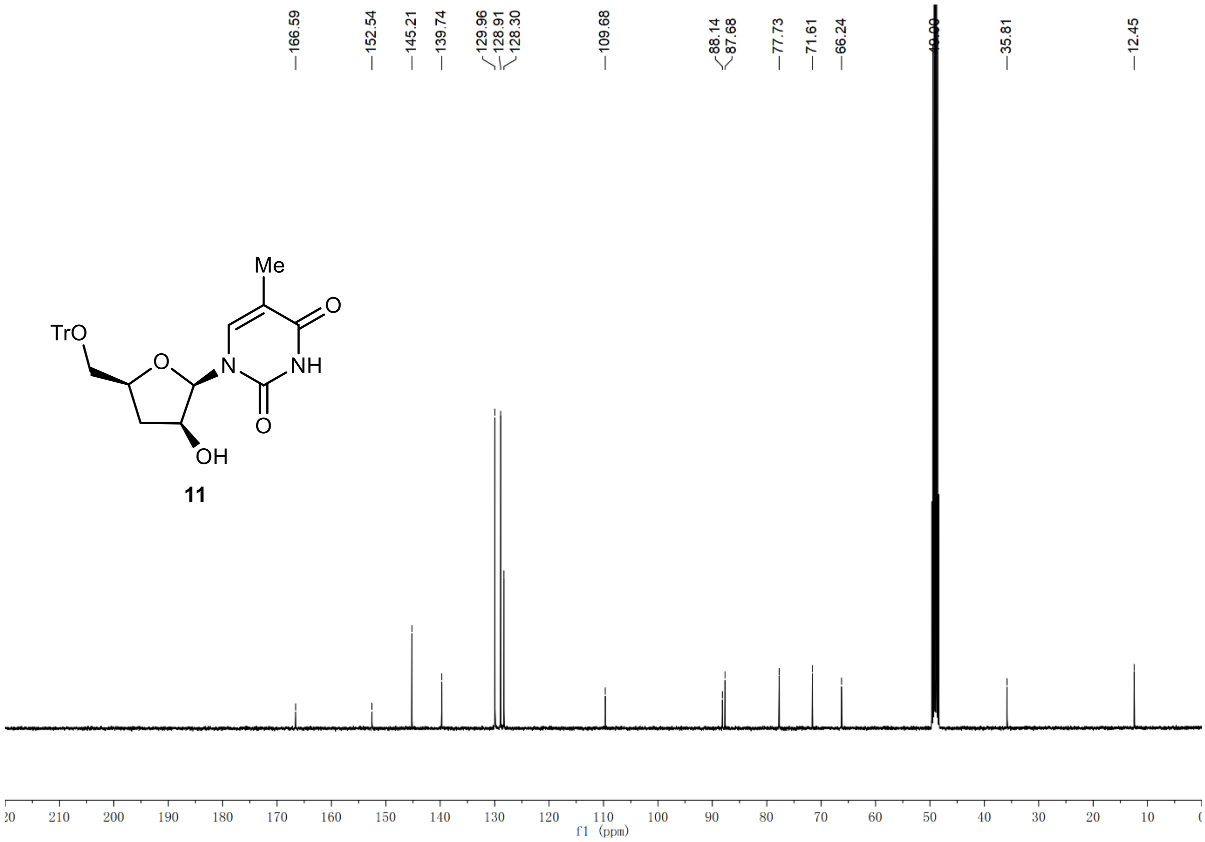

Supplementary Figure 203. ^{13}C NMR (100M, CD_3OD) of compound **11.**

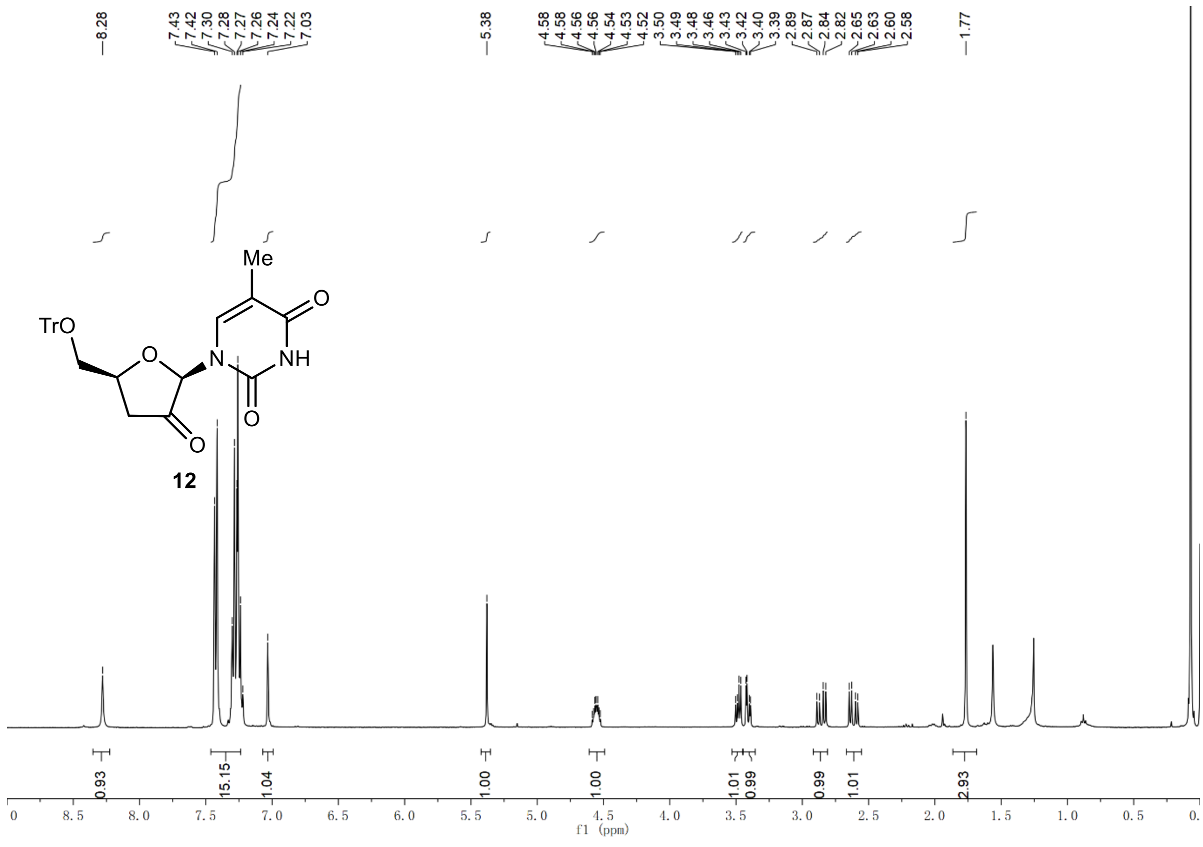

Supplementary Figure 204. ^{1}H NMR (400M, $CDCl_3$) of compound **12.**

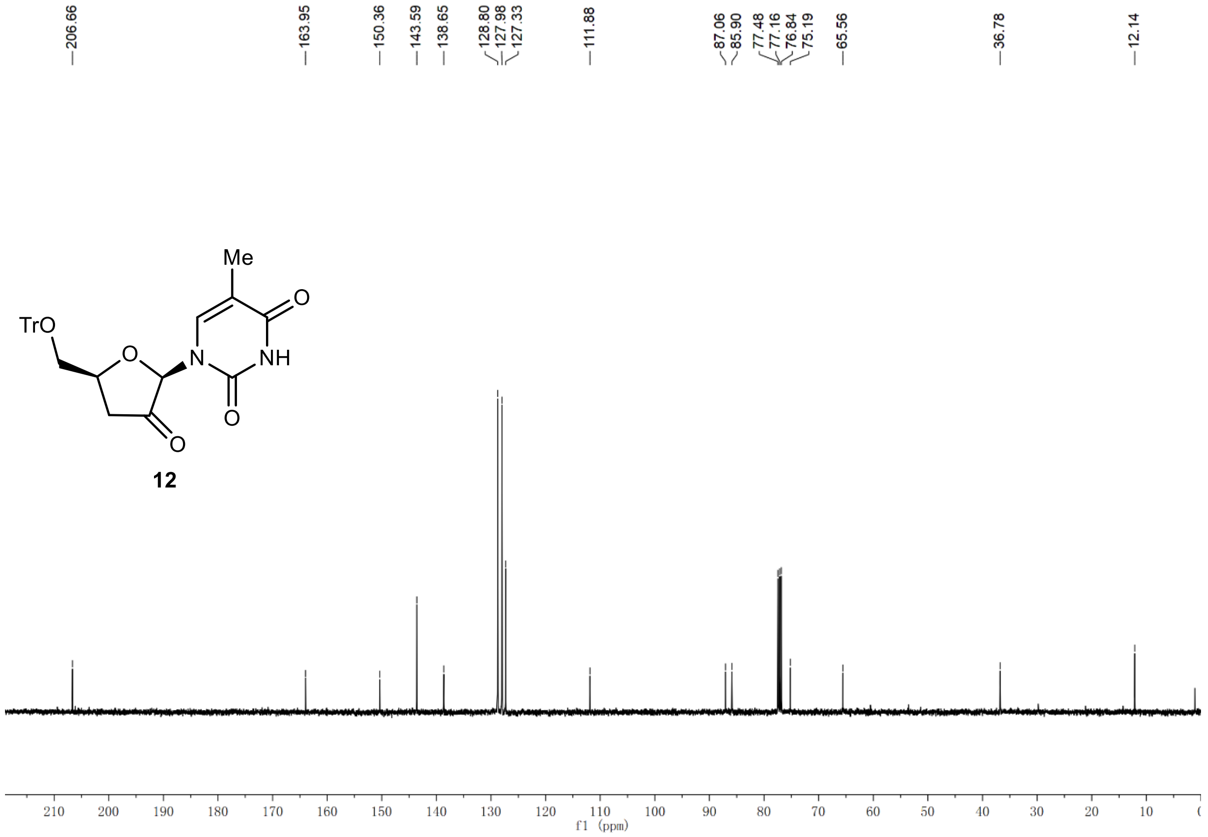

Supplementary Figure 205. ^{13}C NMR (100M, $CDCl_3$) of compound **12.**

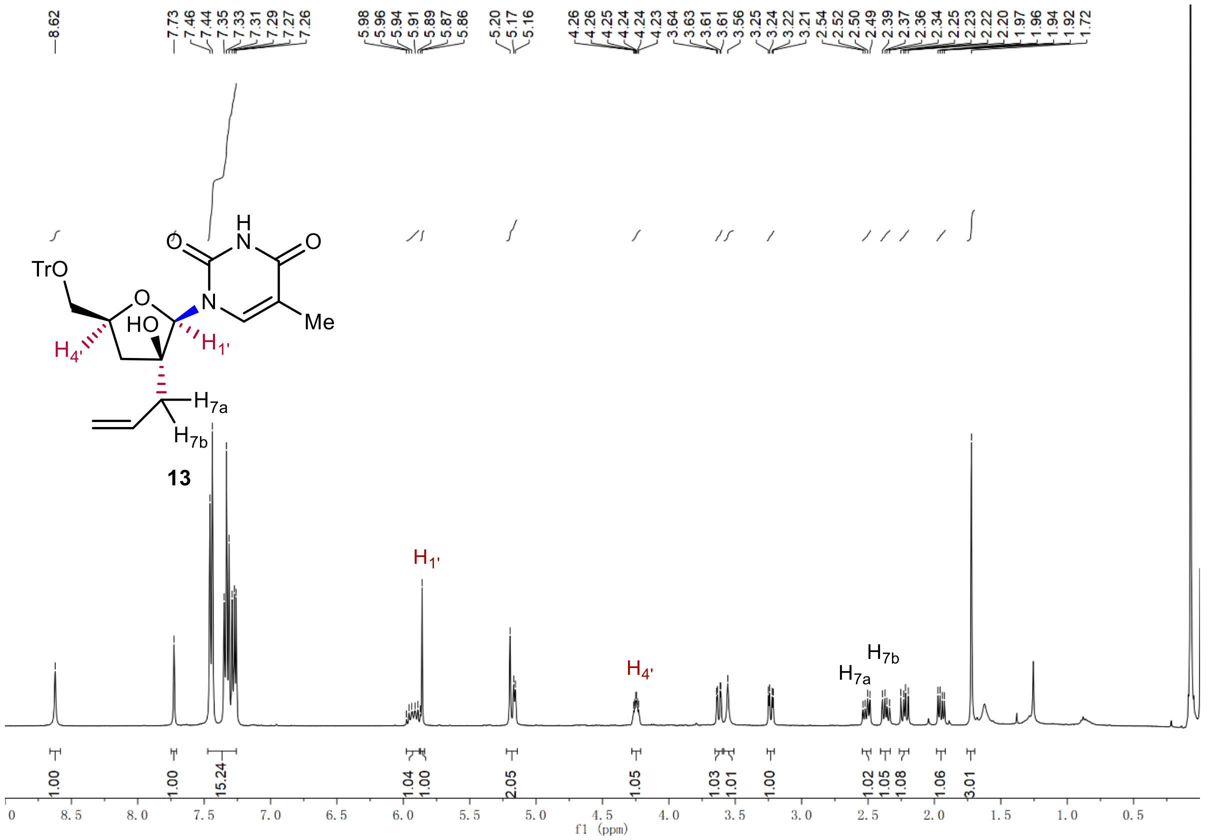

Supplementary Figure 206. ^{1}H NMR (400M, $CDCl_3$) of compound **13.**

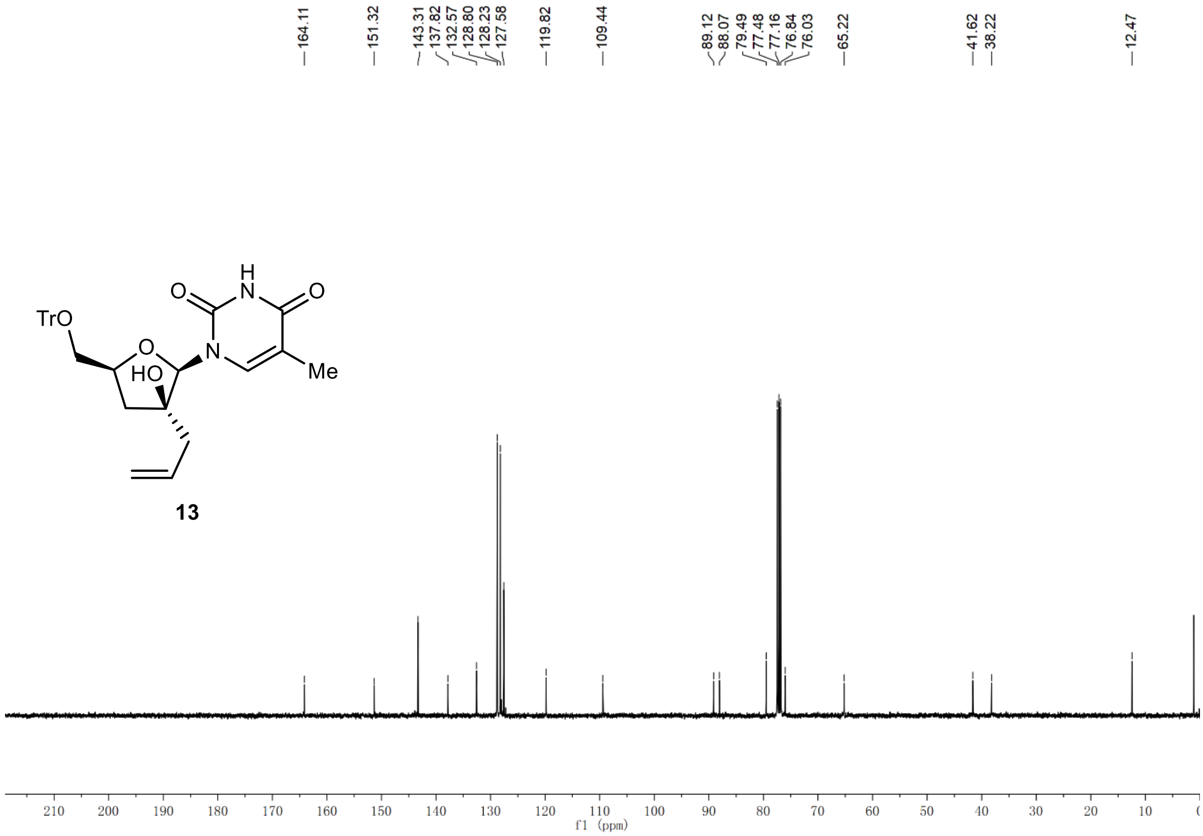

Supplementary Figure 207. ^{13}C NMR (100M, $CDCl_3$) of compound **13.**

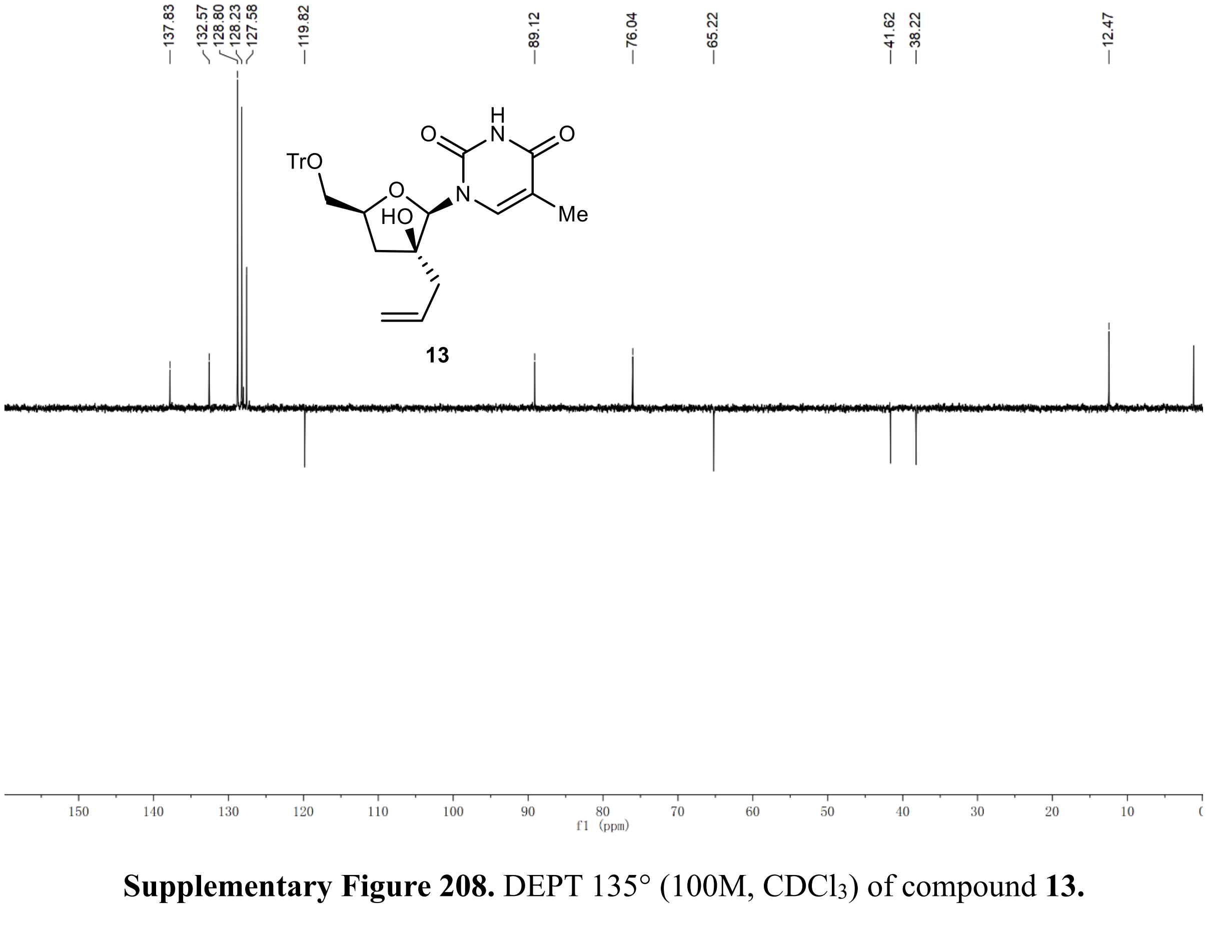

Supplementary Figure 208. DEPT 135° (100M, $CDCl_3$) of compound **13.**

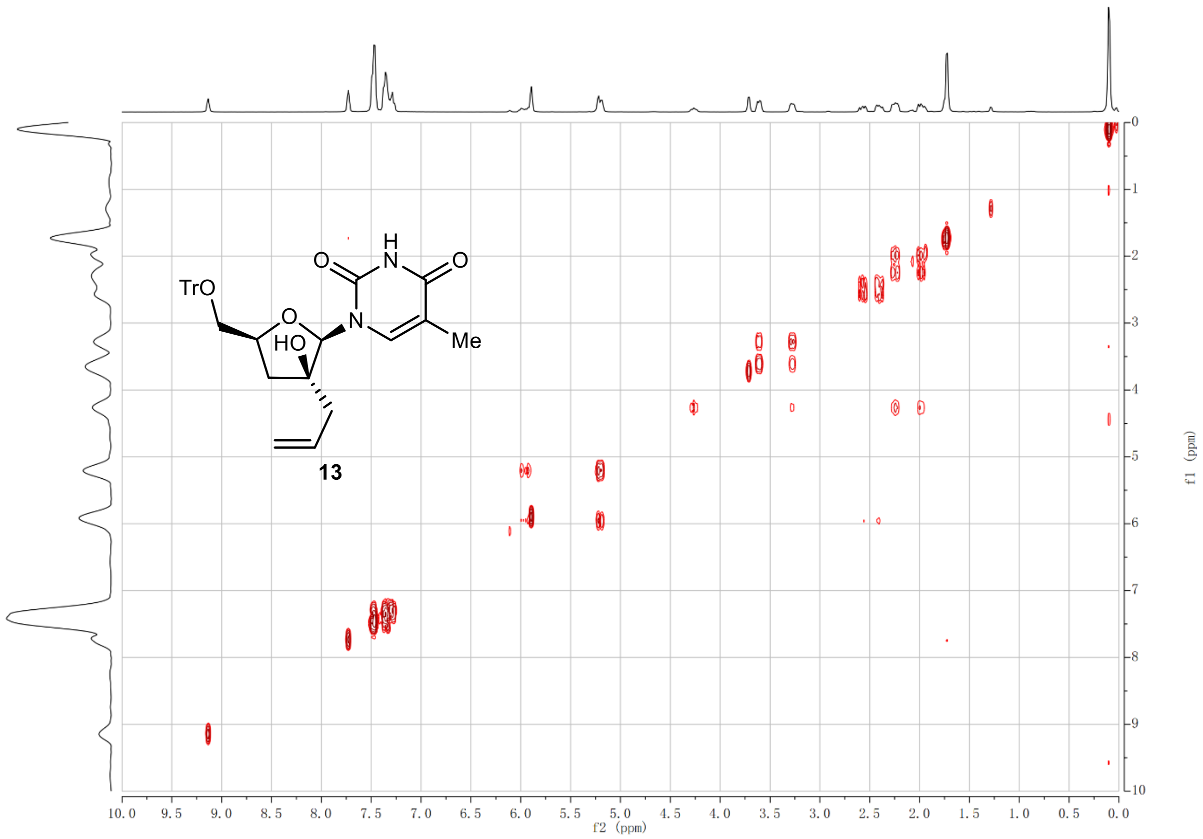

Supplementary Figure 209. ^{1}H-^{1}H COSY (400M, $CDCl_3$) of compound **13.**

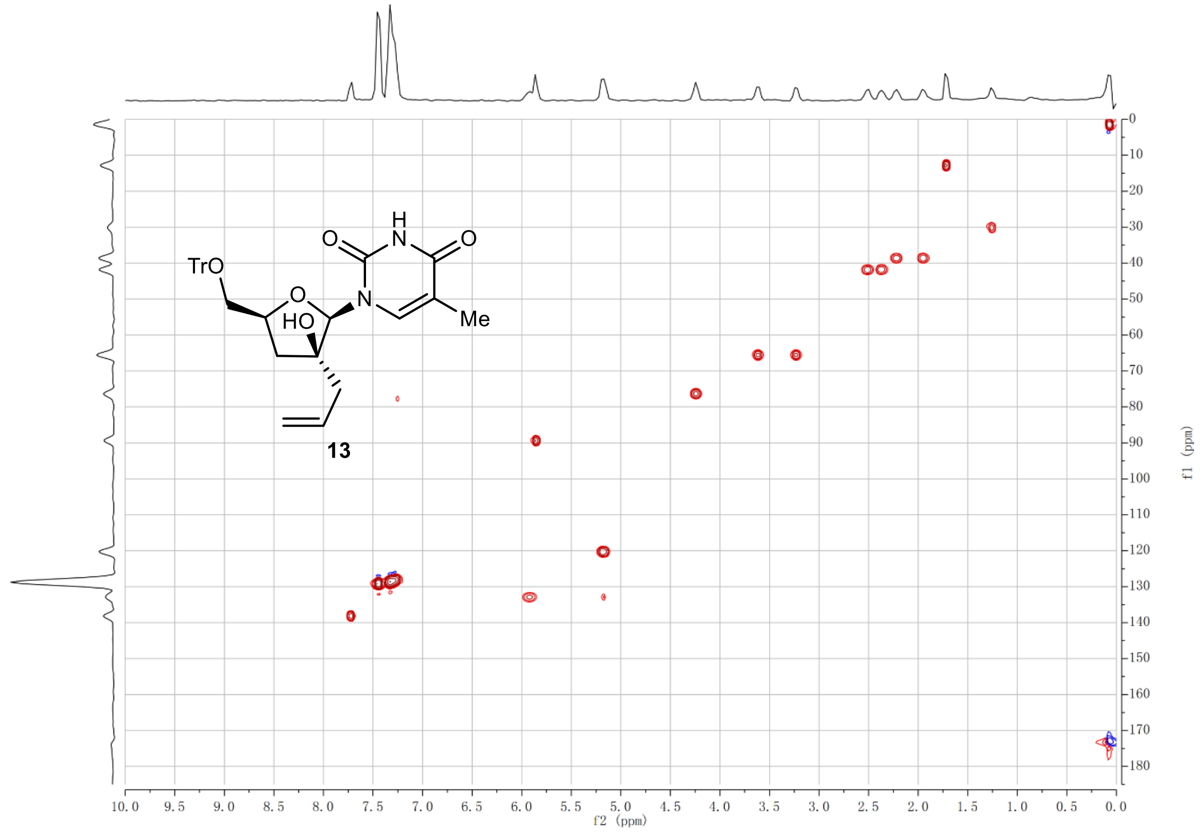

Supplementary Figure 210. ^{1}H-^{13}C HSQC (400M, $CDCl_3$) of compound **13.**

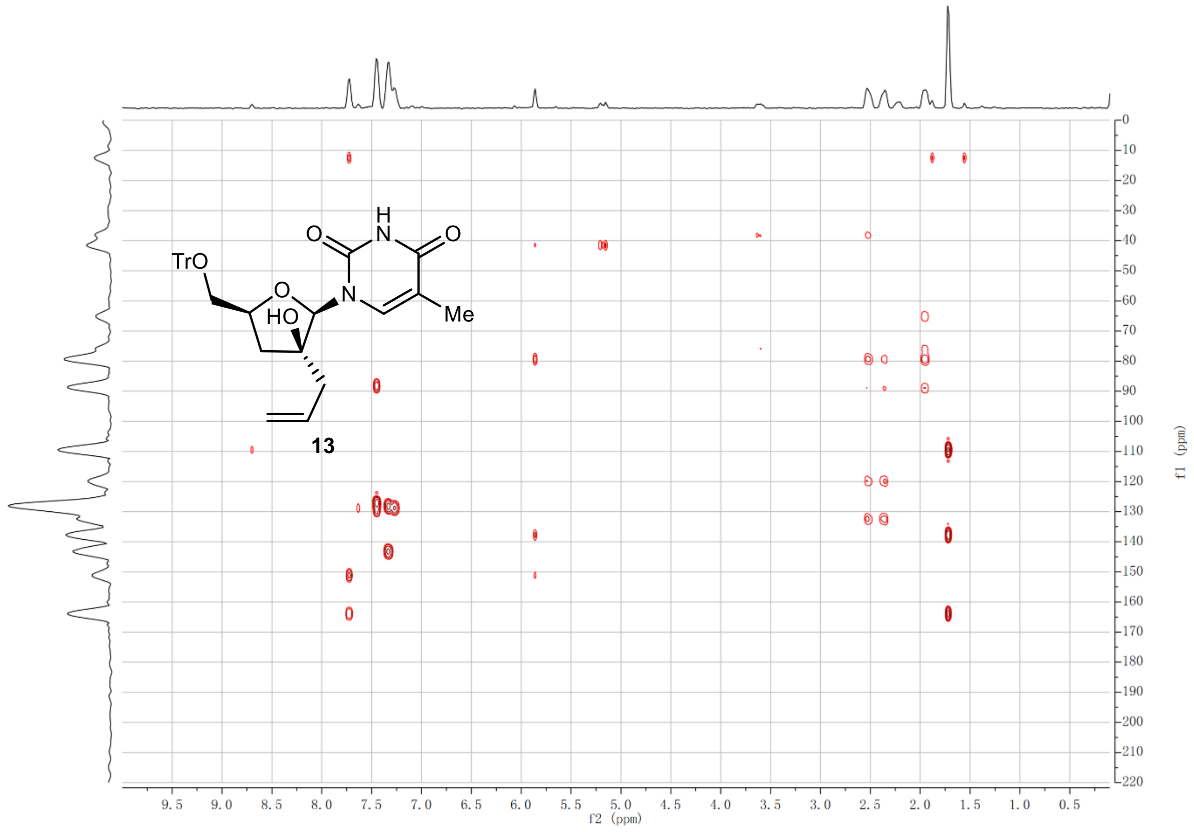

Supplementary Figure 211. ^{1}H-^{13}C HMBC (400M, $CDCl_3$) of compound **13.**

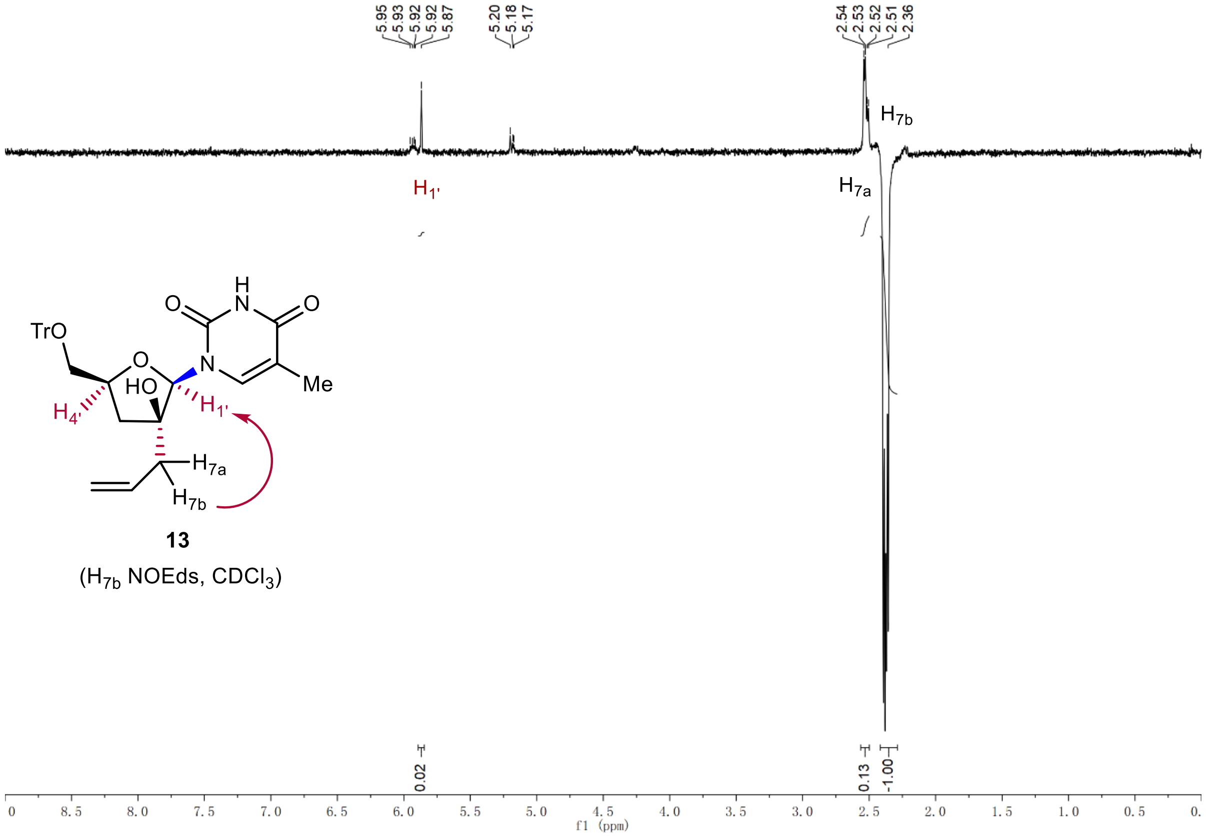

Supplementary Figure 212. H_{7b} NOEds (600M, $CDCl_3$) of compound **13.**

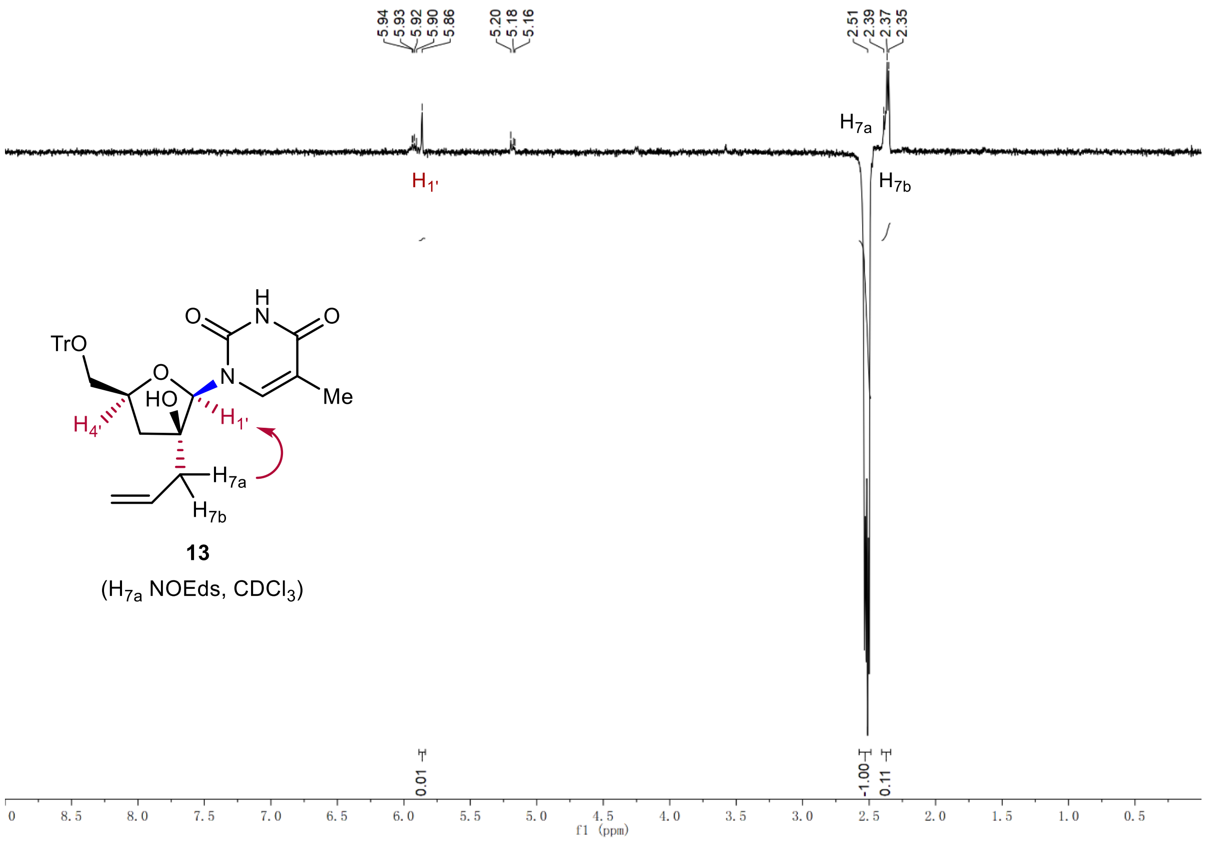

Supplementary Figure 213. H_{7a} NOEds (600M, $CDCl_3$) of compound **13.**

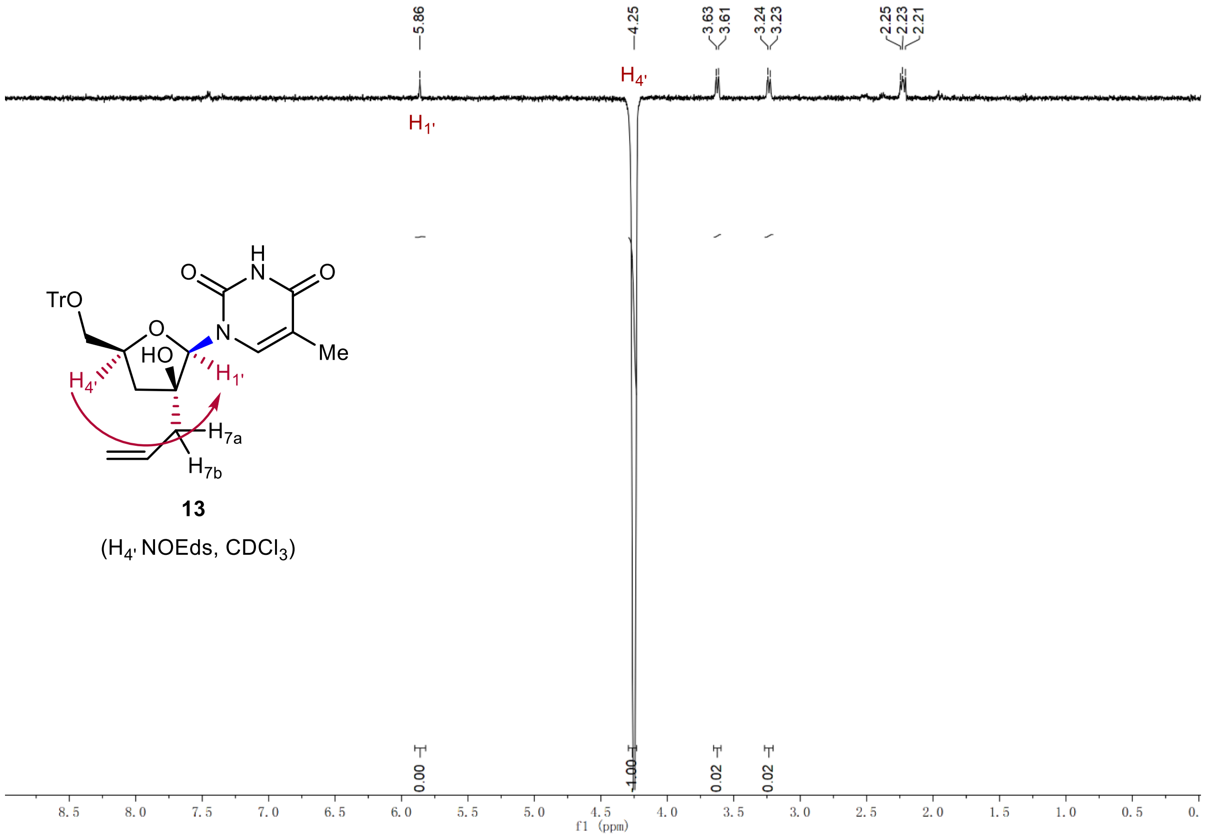

Supplementary Figure 214. $H_{4'}$ NOEds (600M, $CDCl_3$) of compound **13.**

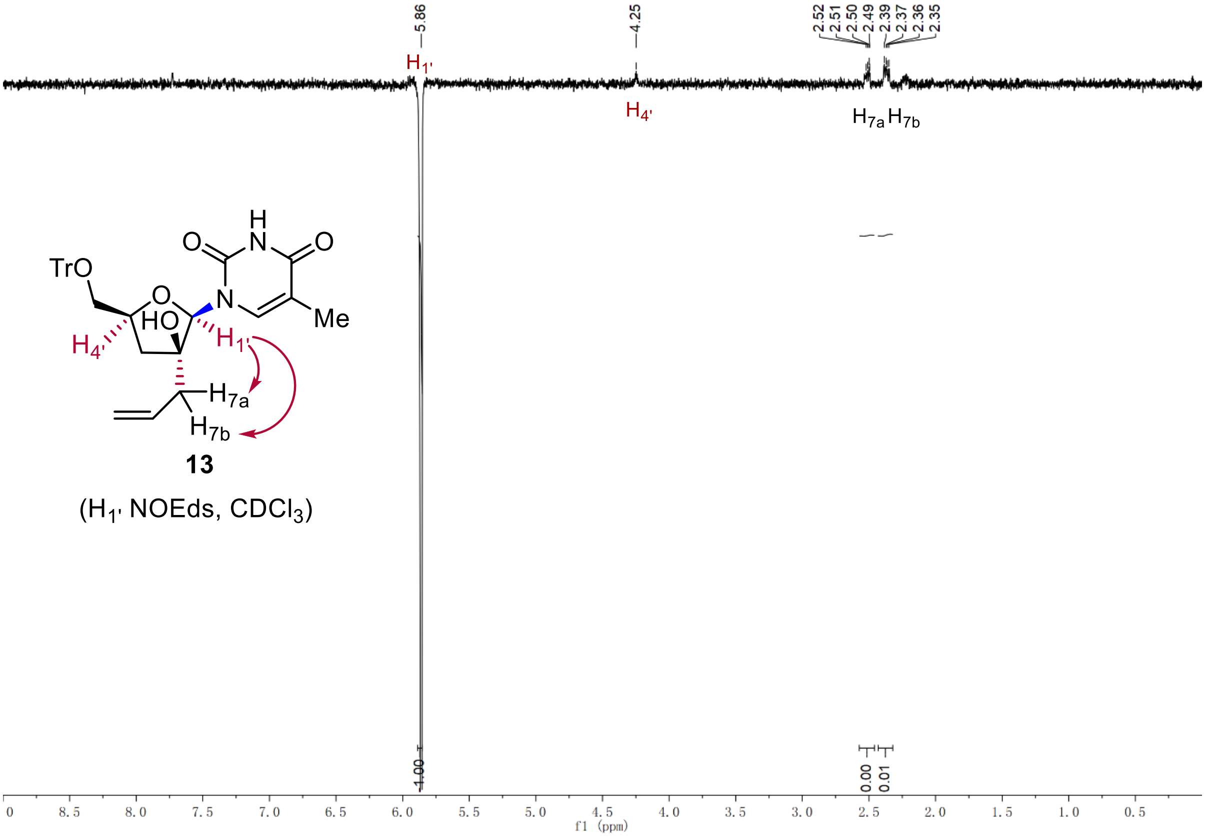

Supplementary Figure 215. $H_{1'}$ NOEds (600M, $CDCl_3$) of compound **13.**

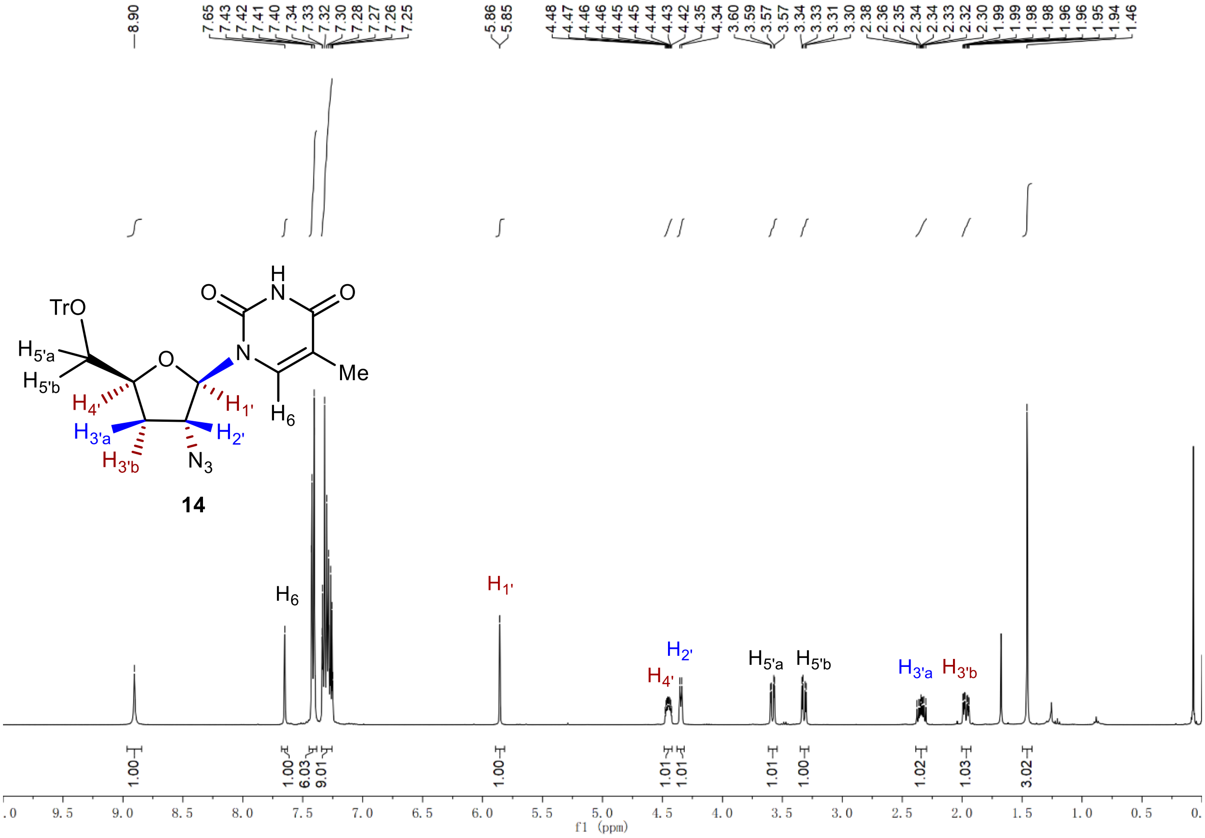

Supplementary Figure 216. ^1H NMR (400M, $CDCl_3$) of compound **14.**

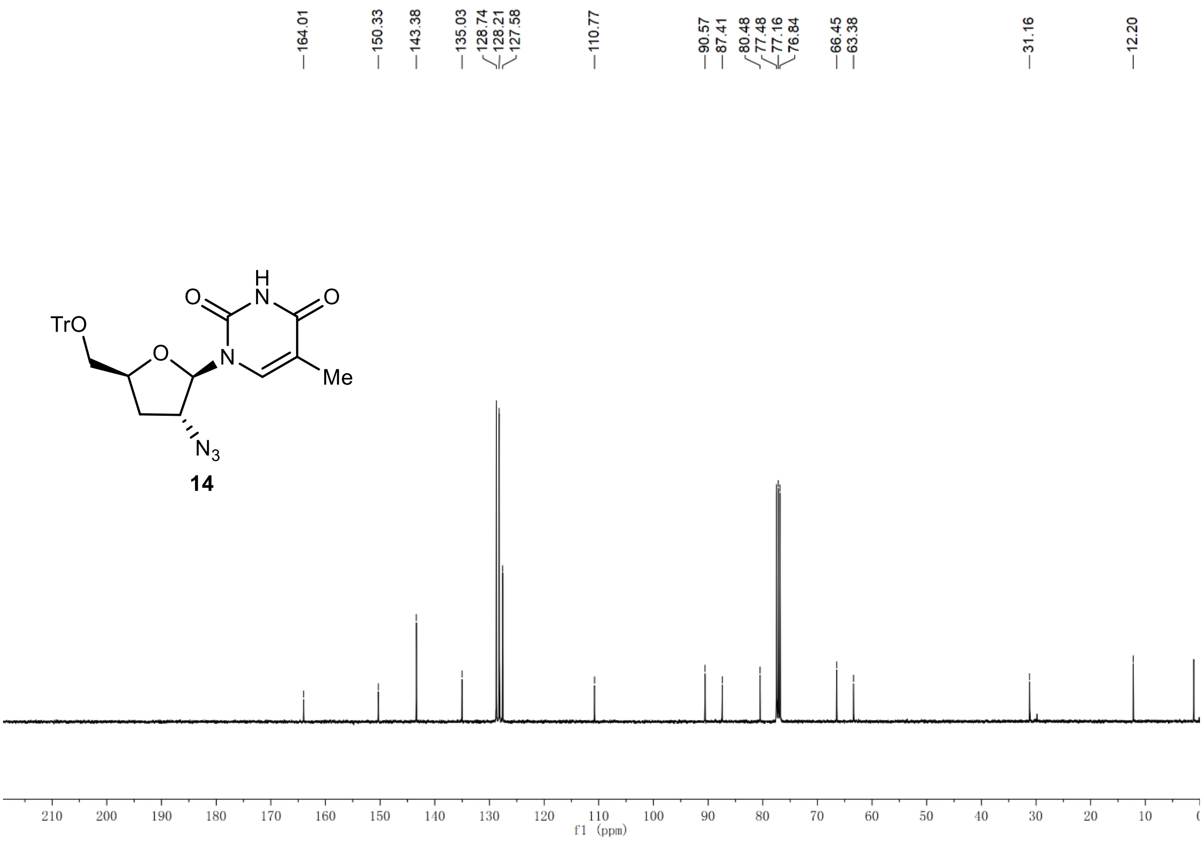

Supplementary Figure 217. ^{13}C NMR (100M, $CDCl_3$) of compound **14.**

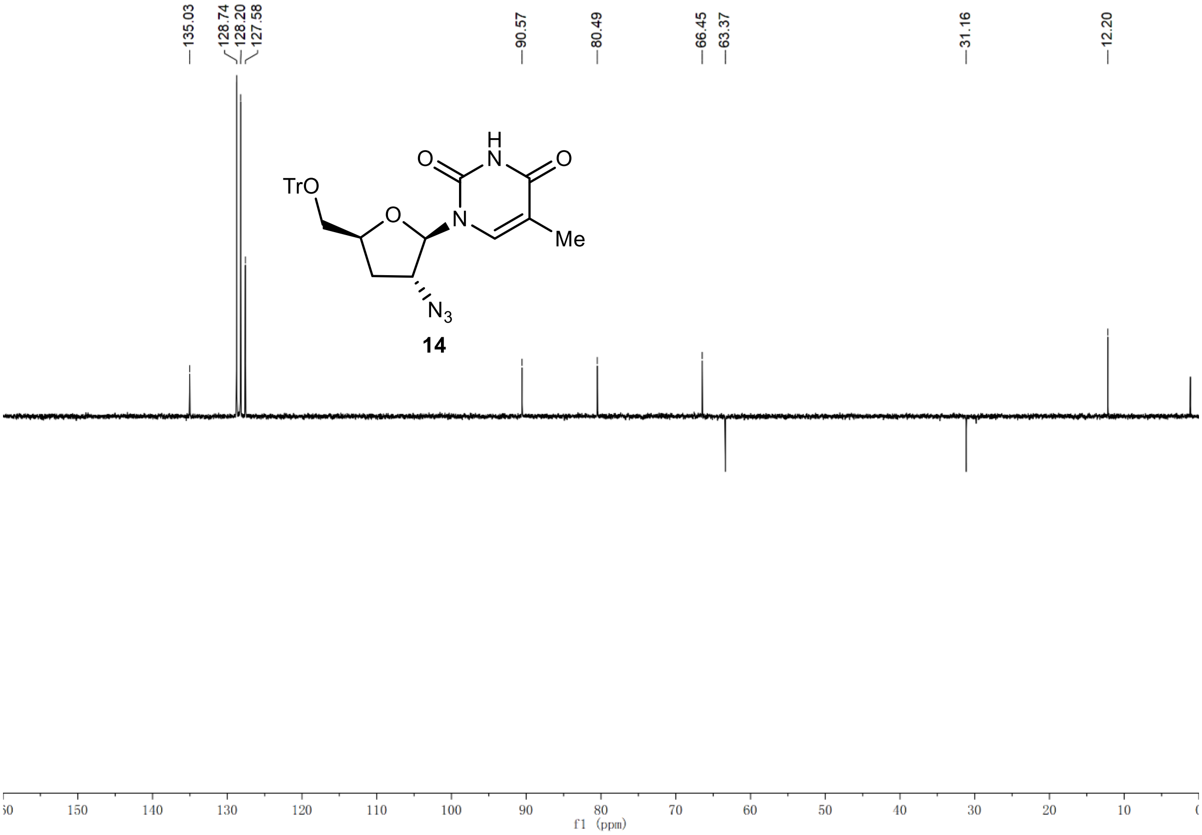

Supplementary Figure 218. DEPT 135° (100M, $CDCl_3$) of compound **14.**

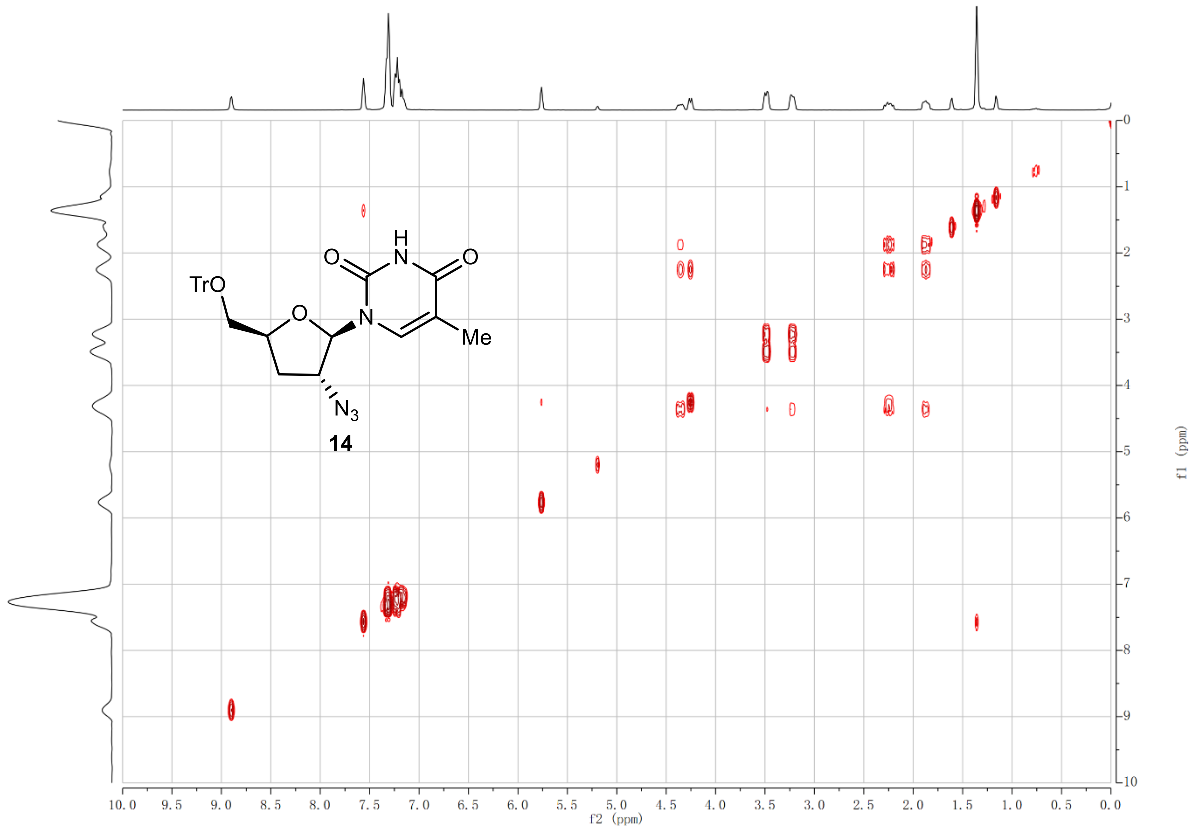

Supplementary Figure 219. ^{1}H-^{1}H COSY (400M, $CDCl_3$) of compound **14.**

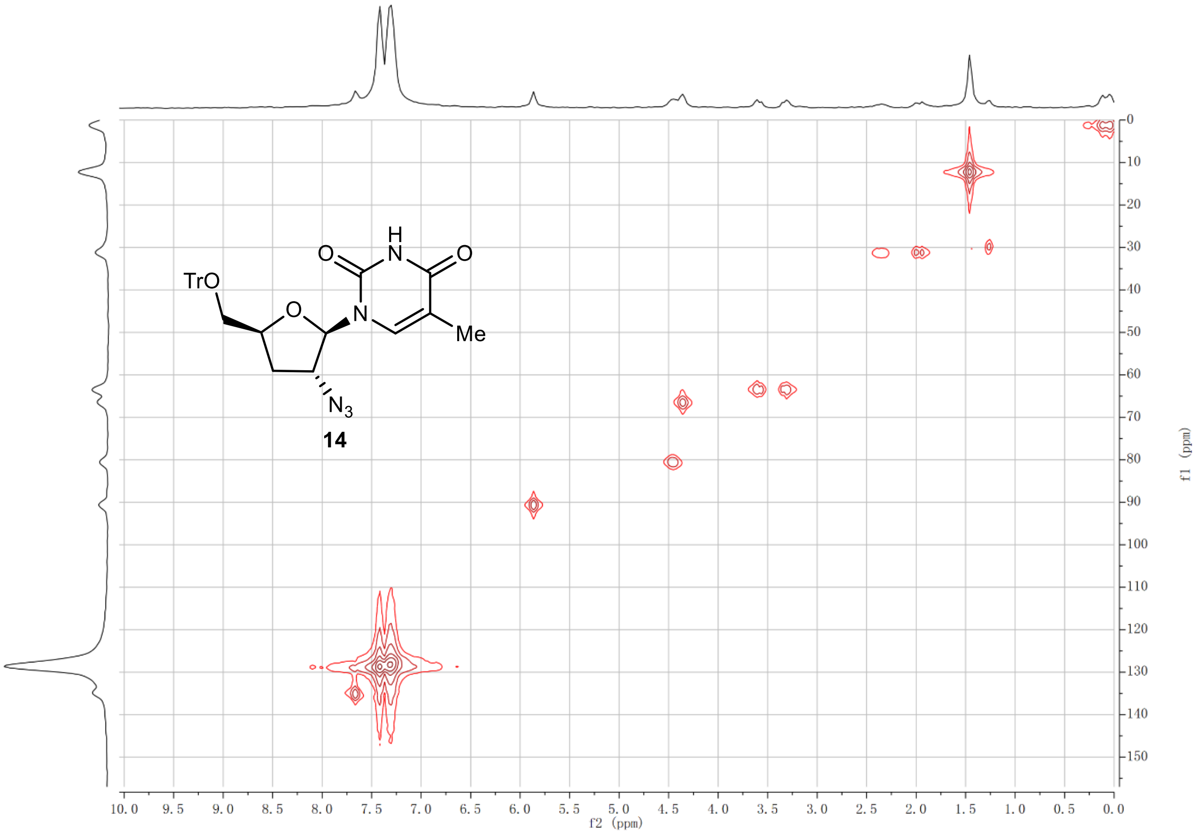

Supplementary Figure 220. ^{1}H-^{13}C HMQC (400M, $CDCl_3$) of compound **14.**

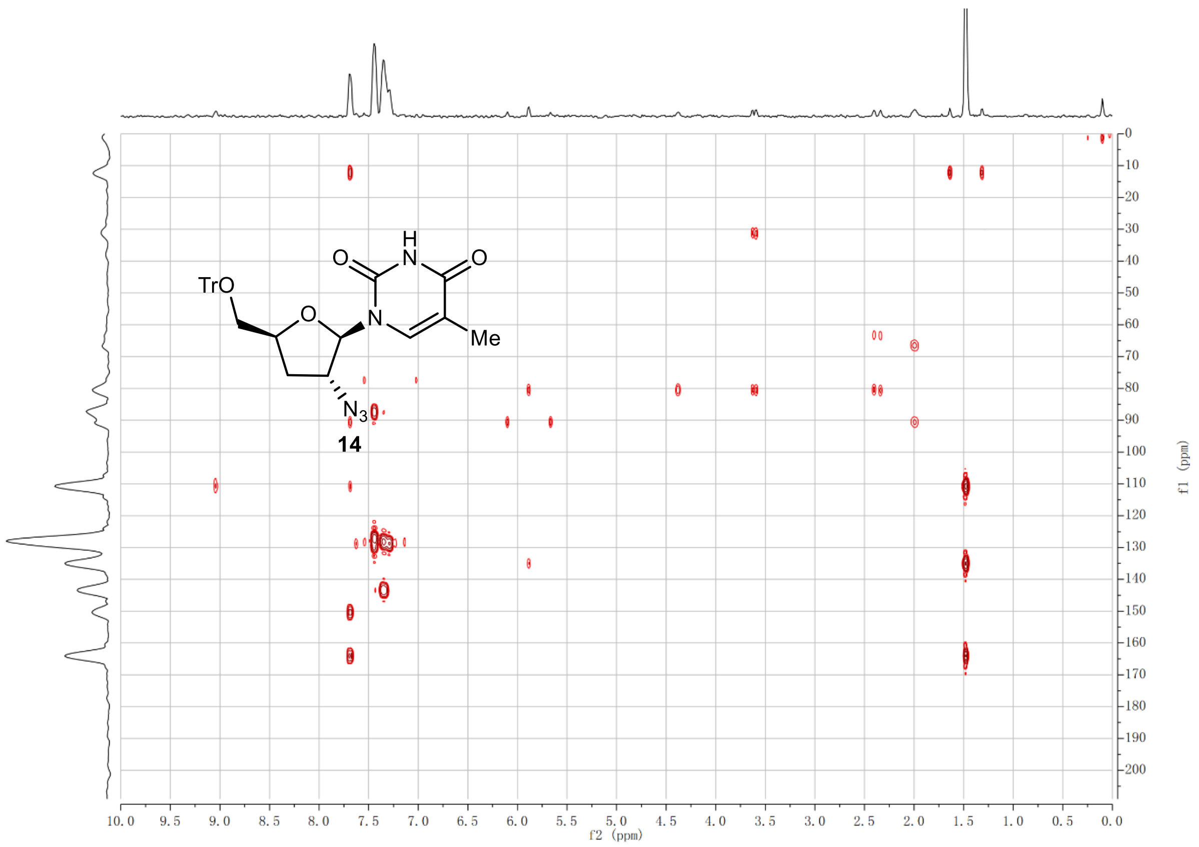

Supplementary Figure 221. ^{1}H-^{13}C HMBC (400M, $CDCl_3$) of compound **14.**

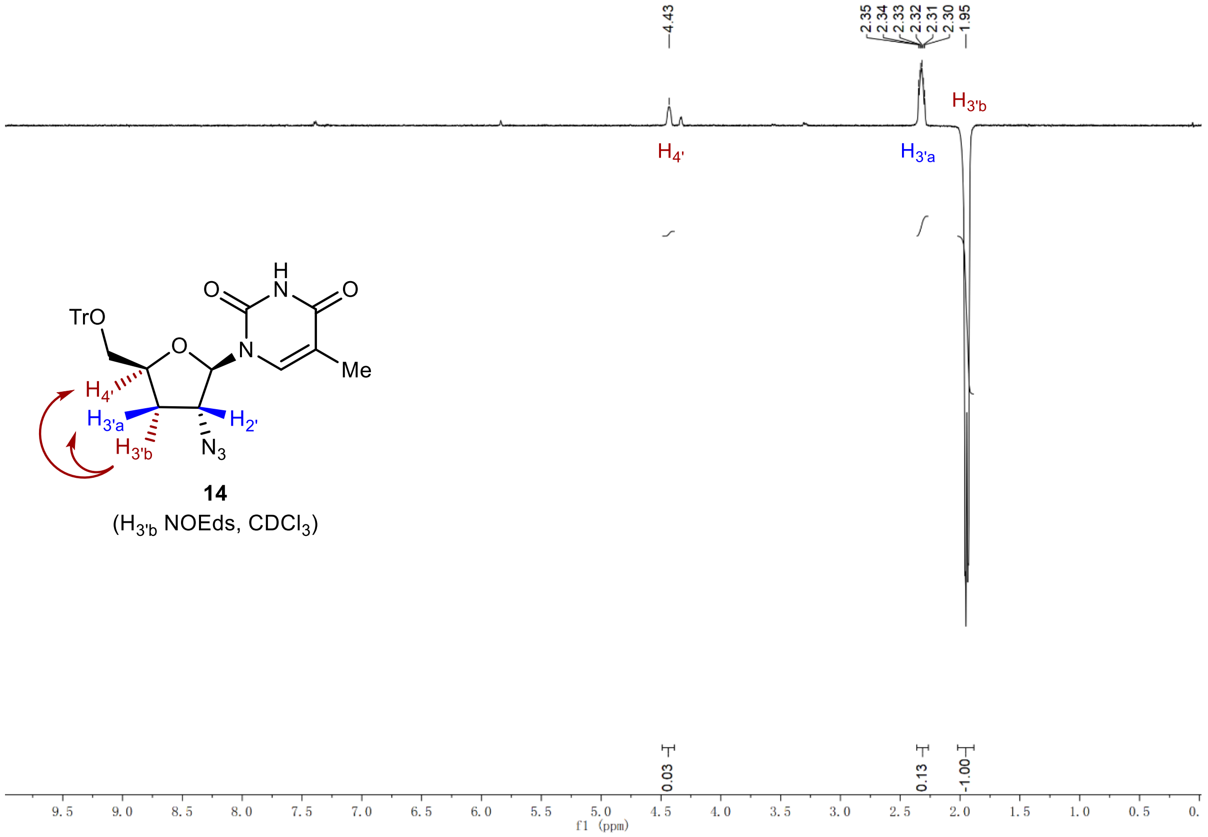

Supplementary Figure 222. $H_{3'b}$ NOEds (600M, $CDCl_3$) of compound **14.**

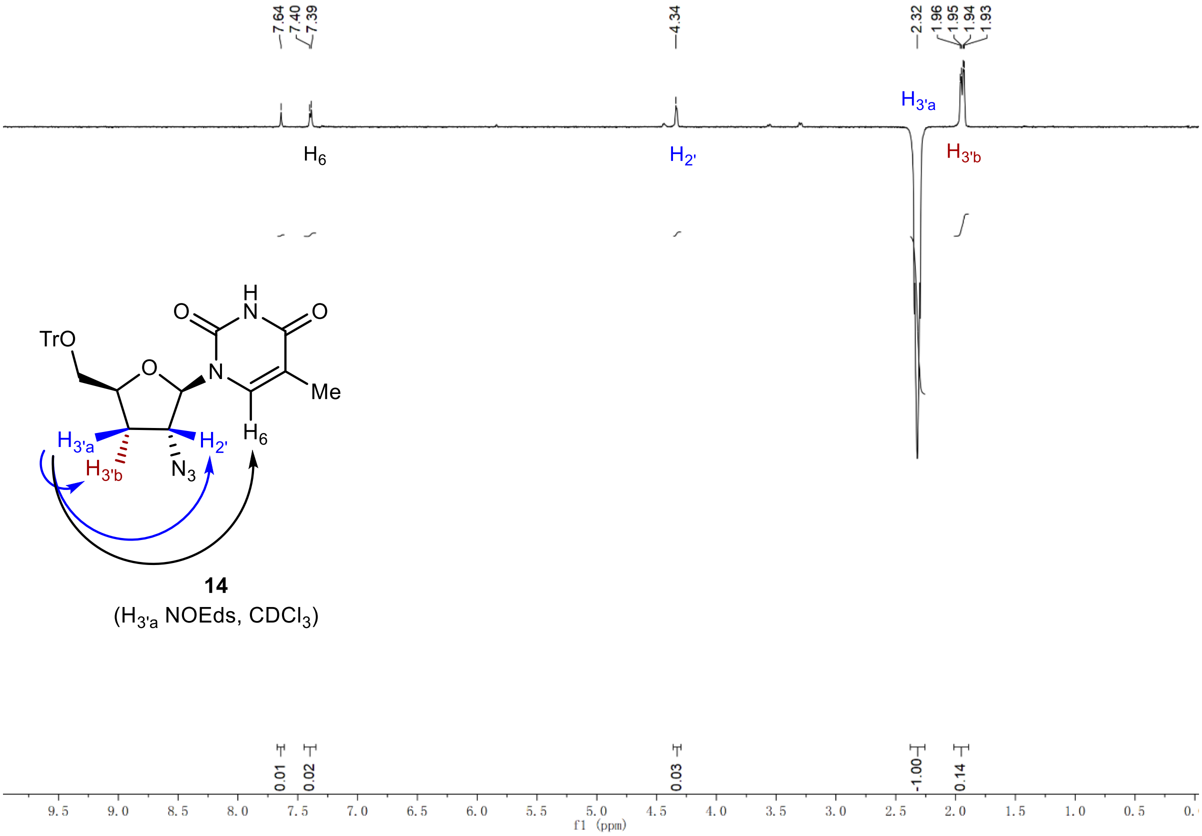

Supplementary Figure 223. $H_{3'a}$ NOEds (600M, $CDCl_3$) of compound **14.**

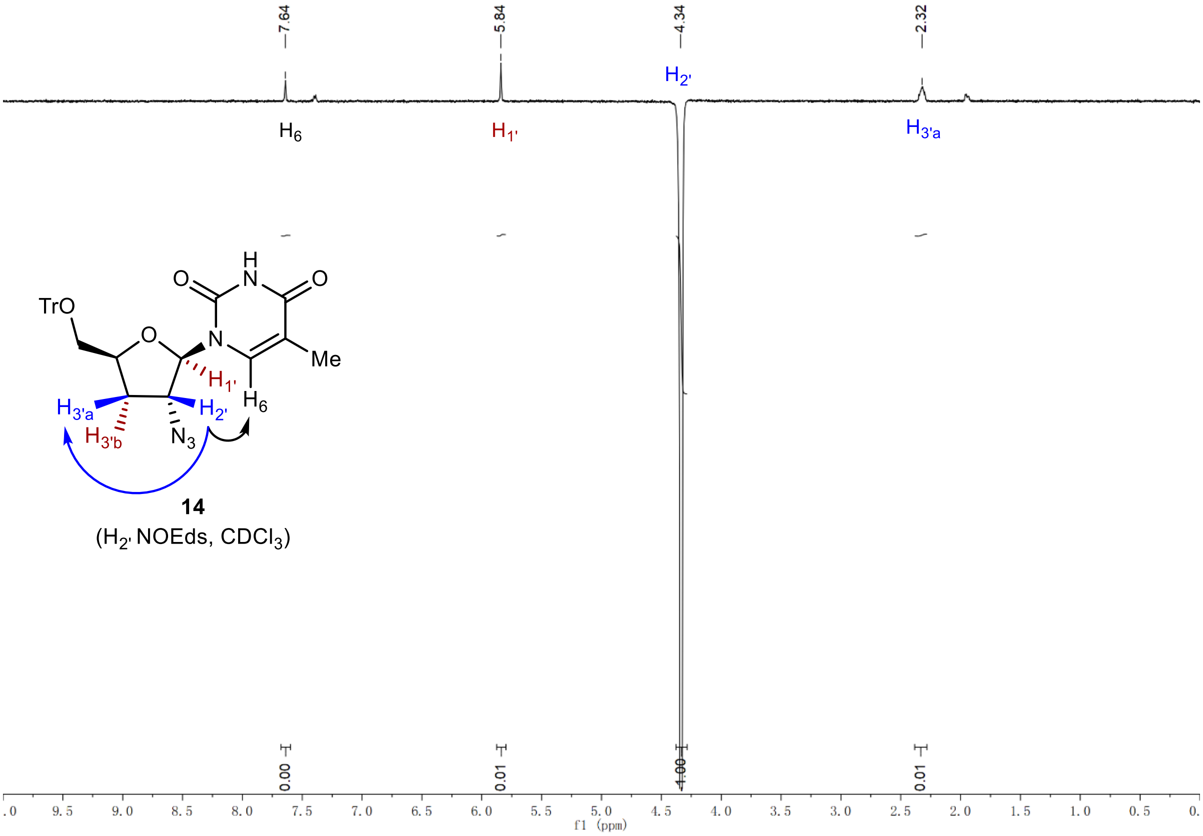

Supplementary Figure 224. $H_{2'}$ NOEds (600M, $CDCl_3$) of compound **14.**

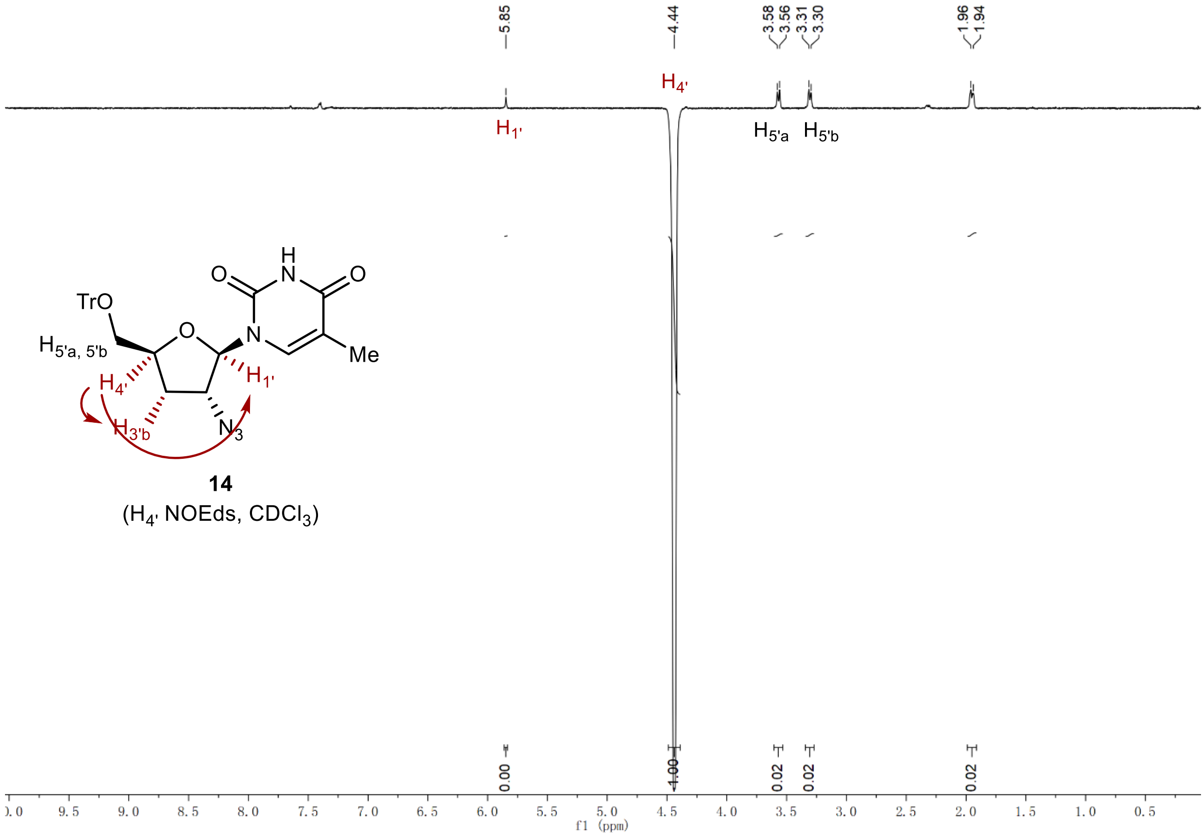

Supplementary Figure 225. $H_{4'}$ NOEds (600M, $CDCl_3$) of compound **14.**

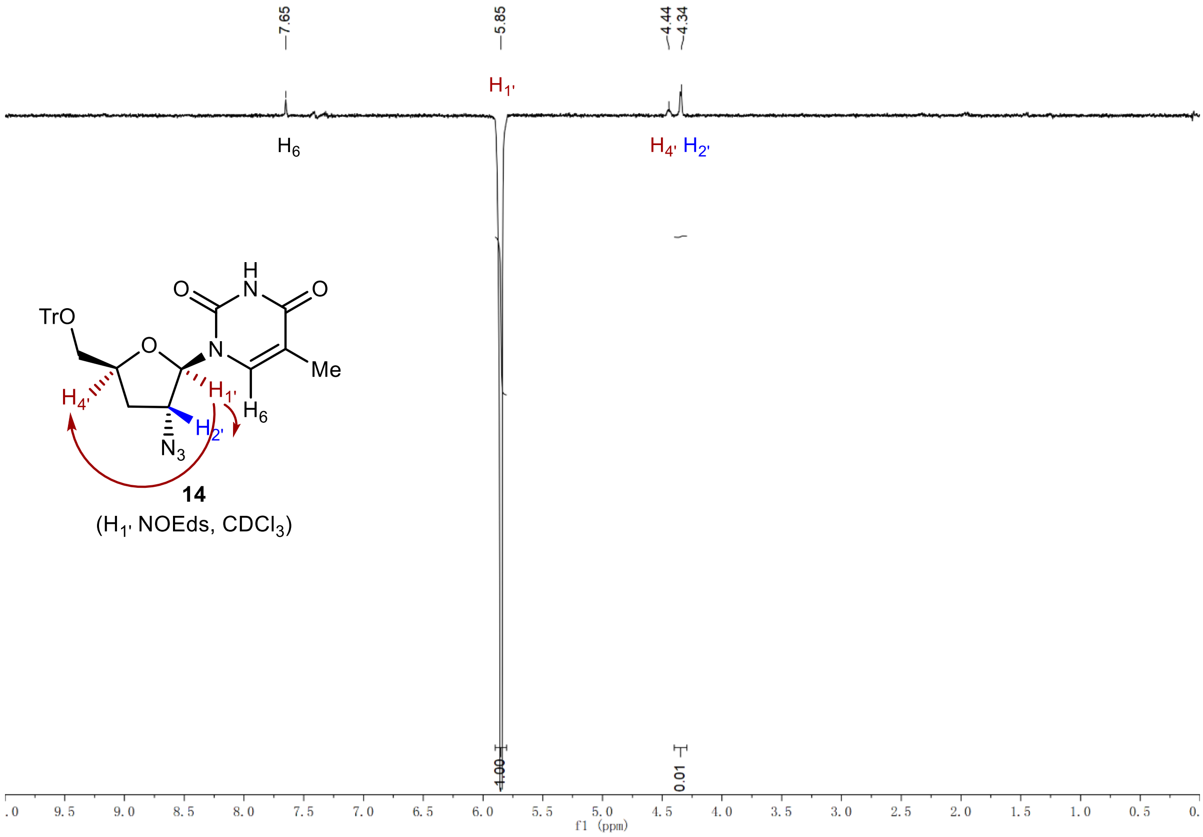

Supplementary Figure 226. $H_{1'}$ NOEds (600M, $CDCl_3$) of compound **14.**

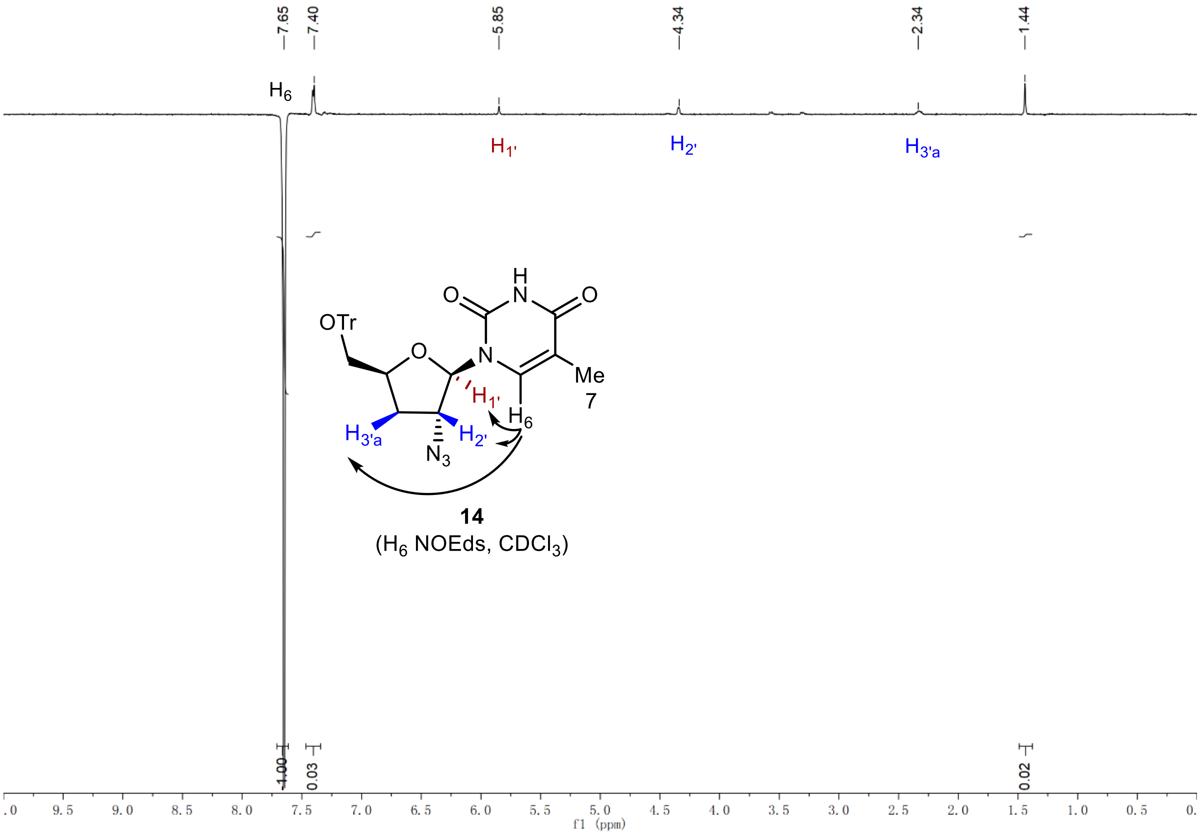

Supplementary Figure 227. H_6 NOEds (600M, $CDCl_3$) of compound **14.**

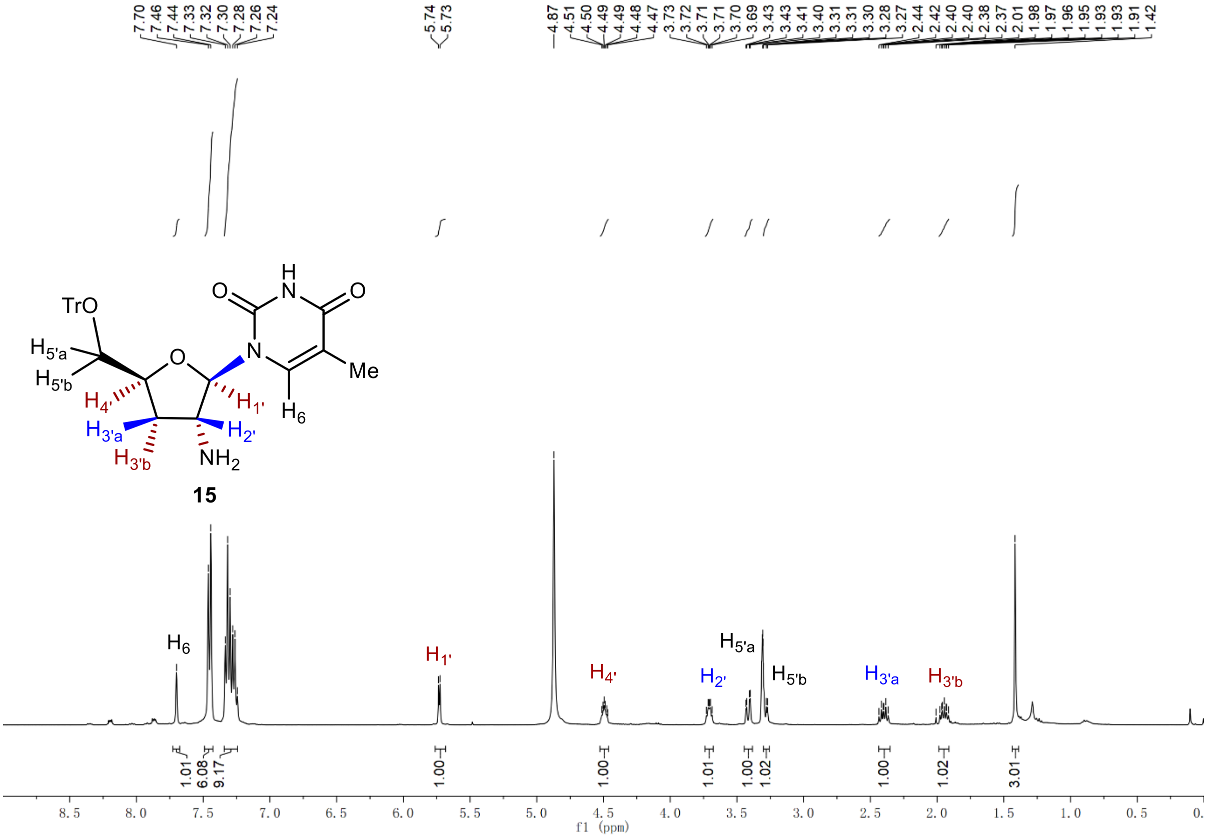

Supplementary Figure 228. ^{1}H NMR (400M, CD_3OD) of compound **15.**

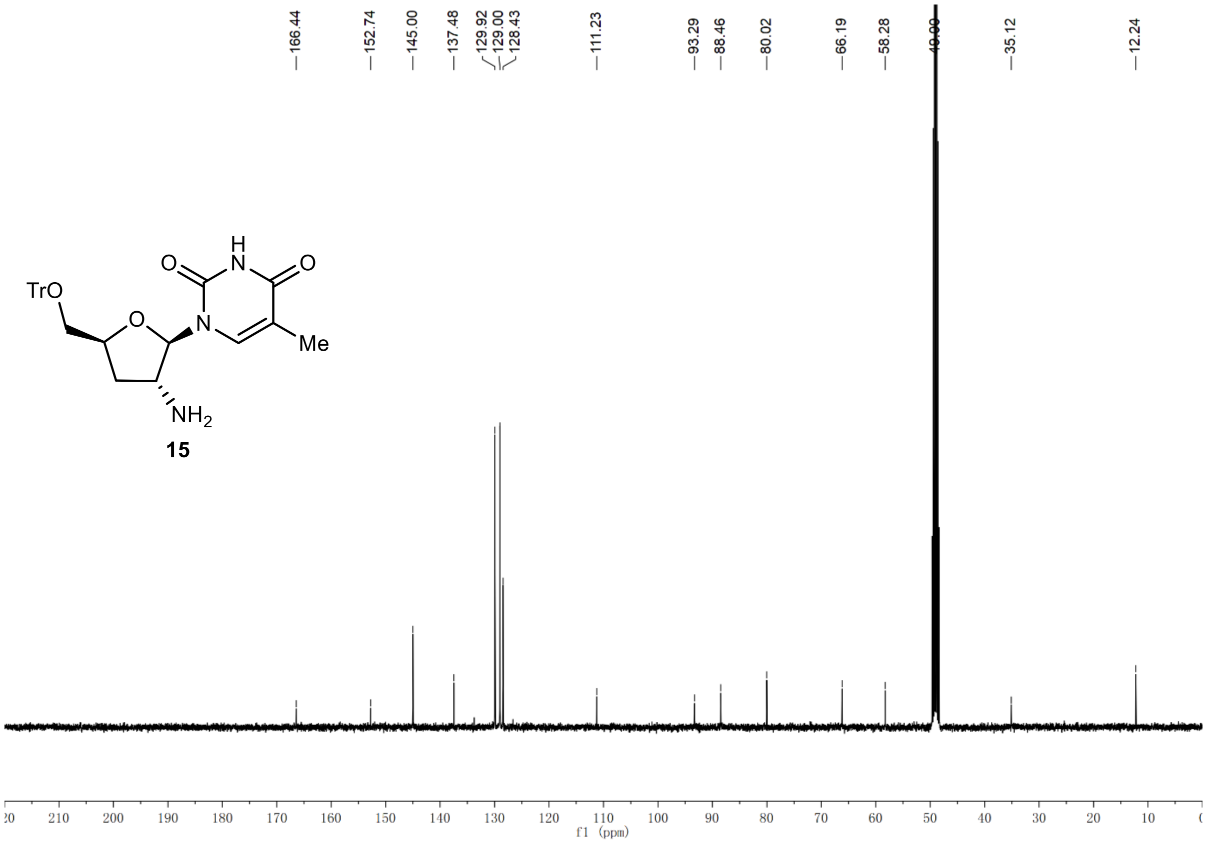

Supplementary Figure 229. ^{13}C NMR (100M, CD_3OD) of compound **15.**

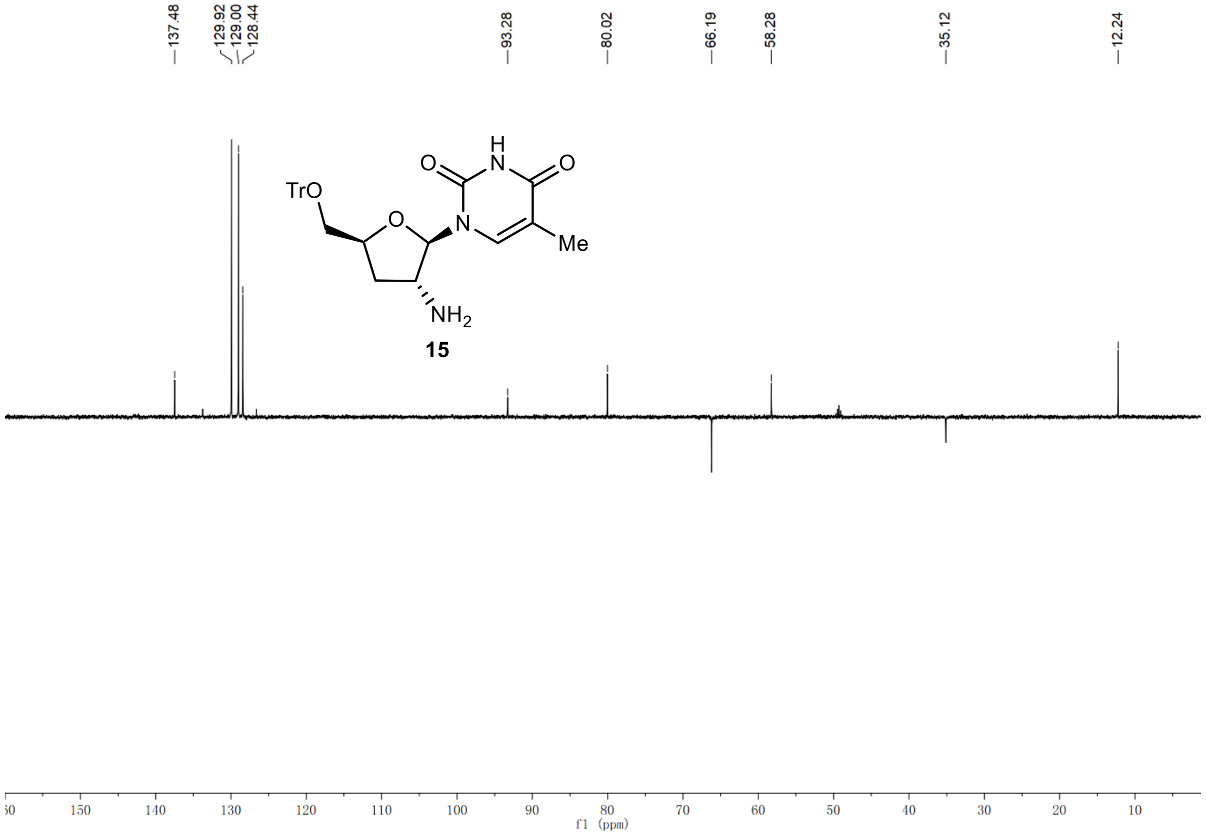

Supplementary Figure 230. DEPT 135° (100 M, CD_3OD) of compound **15.**

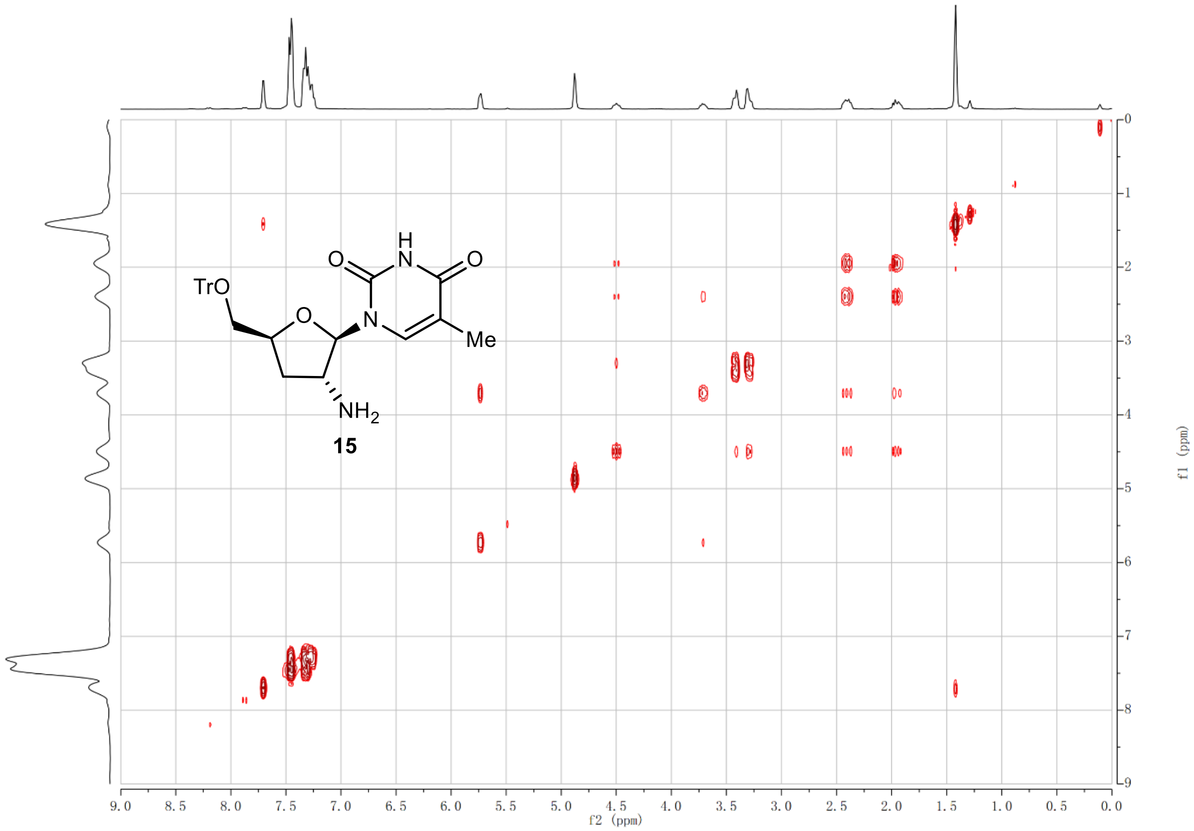

Supplementary Figure 231. ^{1}H-^{1}H COSY (400 M, CD$_3$OD) of compound **15.**

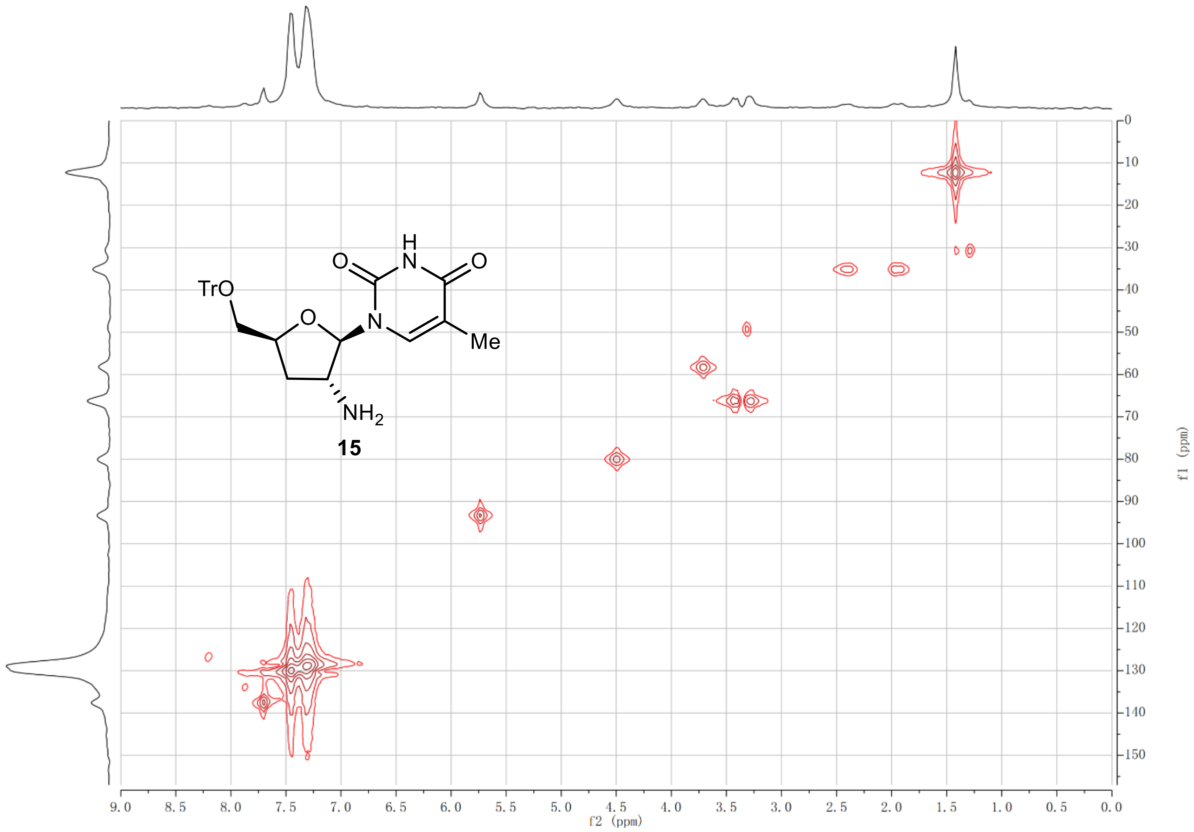

Supplementary Figure 232. ^{1}H-^{13}C HMQC (400M, CD$_3$OD) of compound **15.**

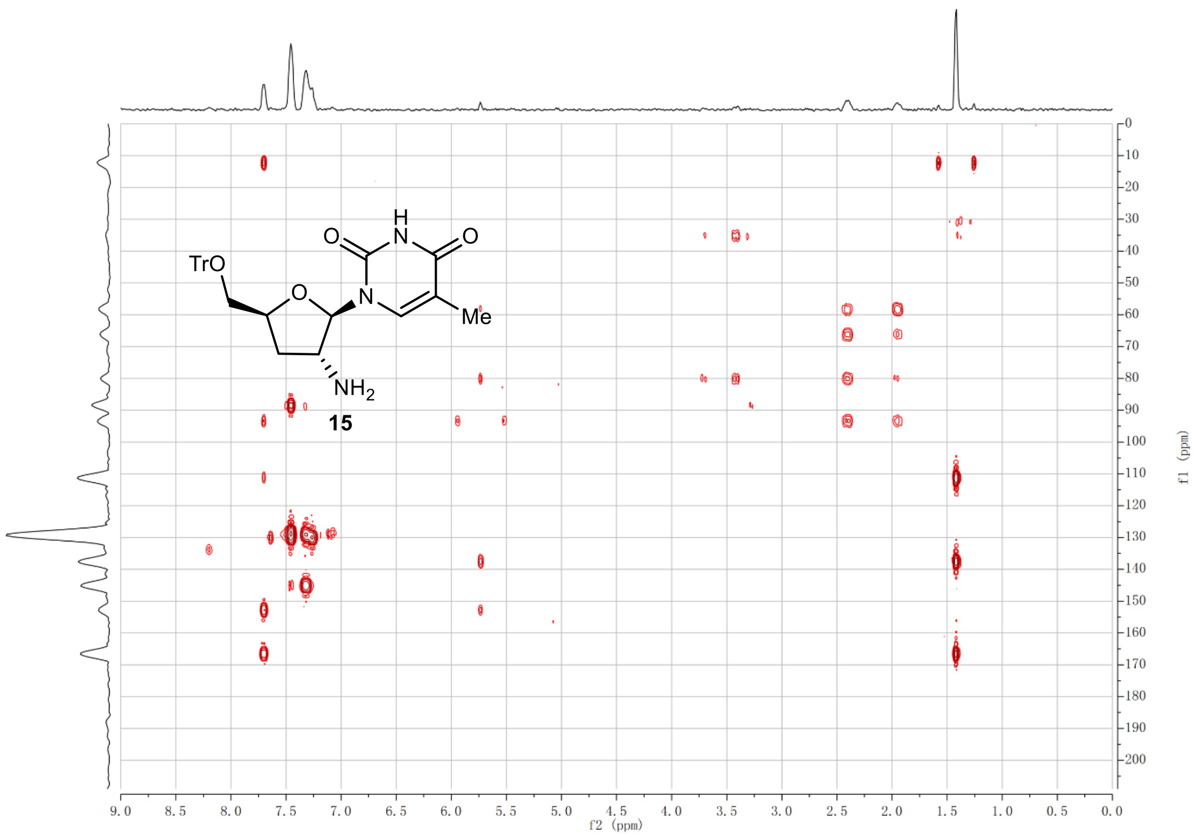

Supplementary Figure 233. ^{1}H-^{13}C HMBC (400M, CD3OD) of compound **15.**

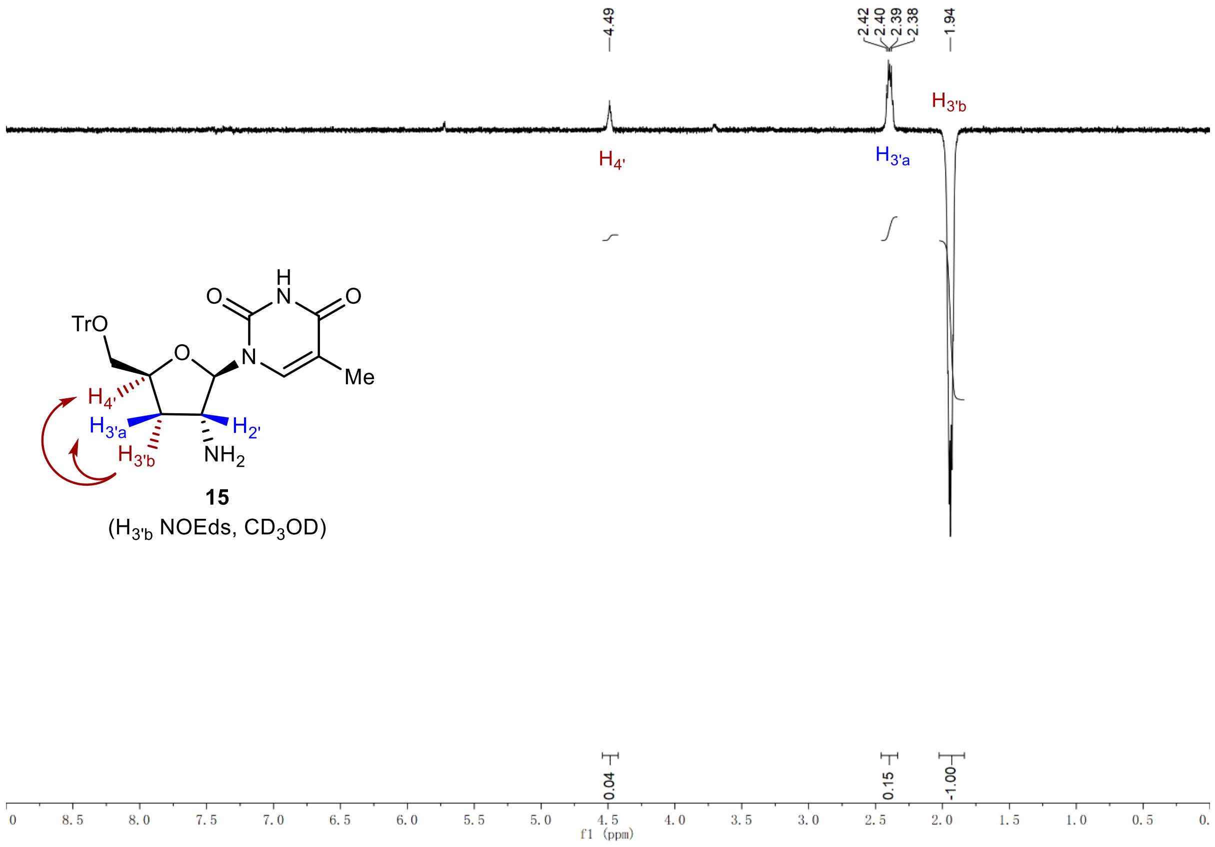

Supplementary Figure 234. H3'b NOEds (600M, CD3OD) of compound **15.**

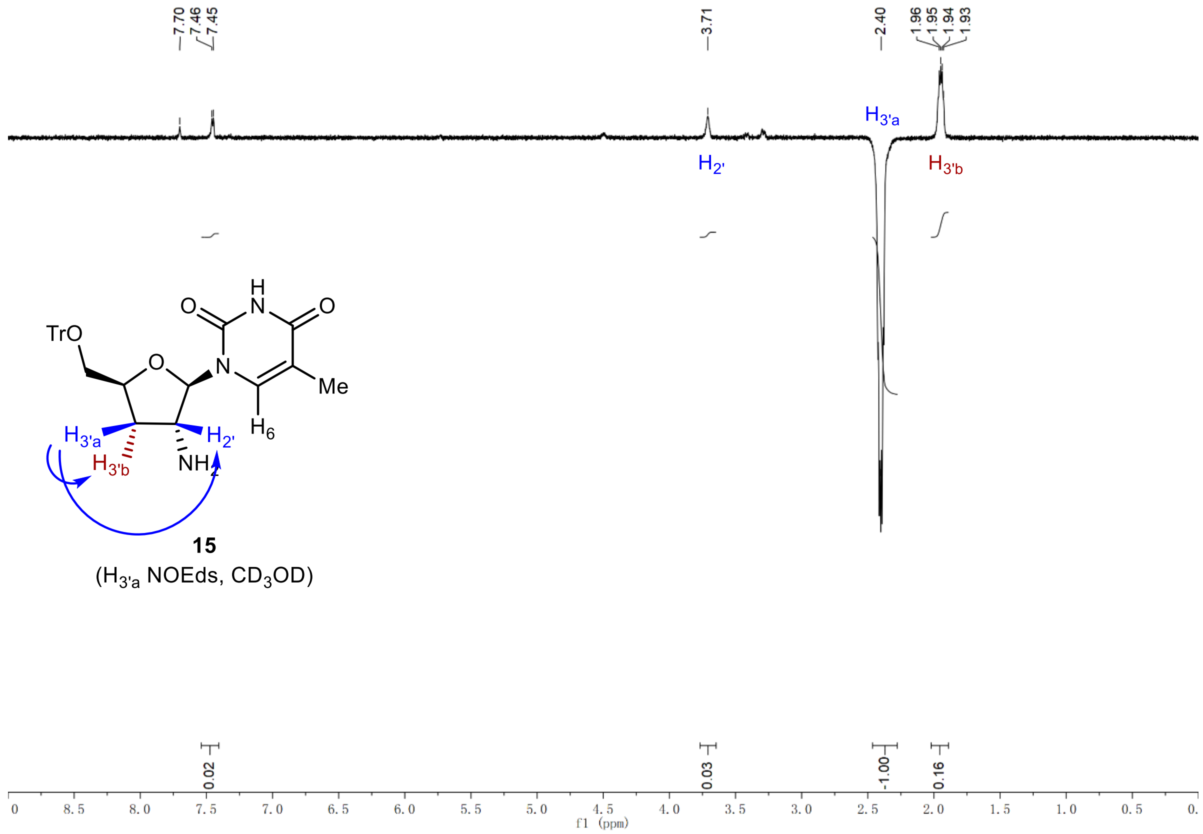

Supplementary Figure 235. $H_{3'a}$ NOEds (600M, CD_3OD) of compound **15.**

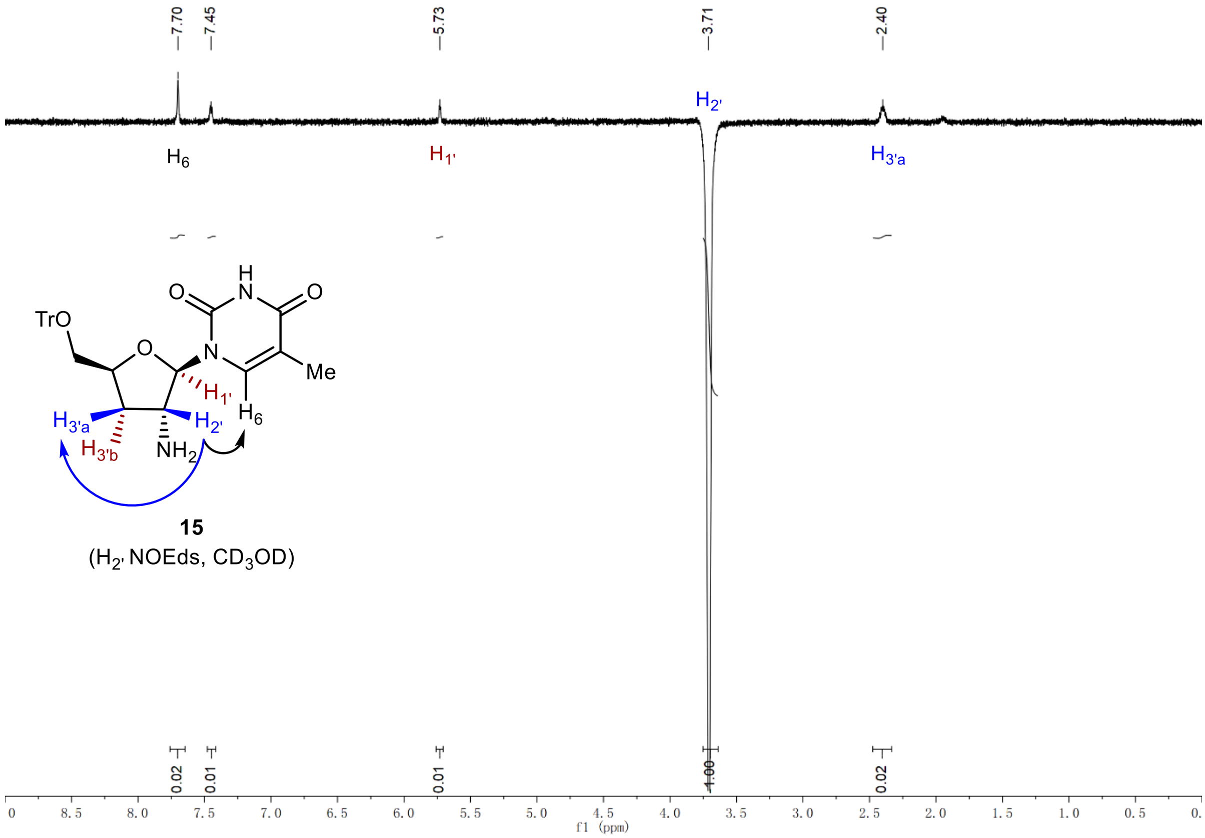

Supplementary Figure 236. $H_{2'}$ NOEds (600M, CD_3OD) of compound **15.**

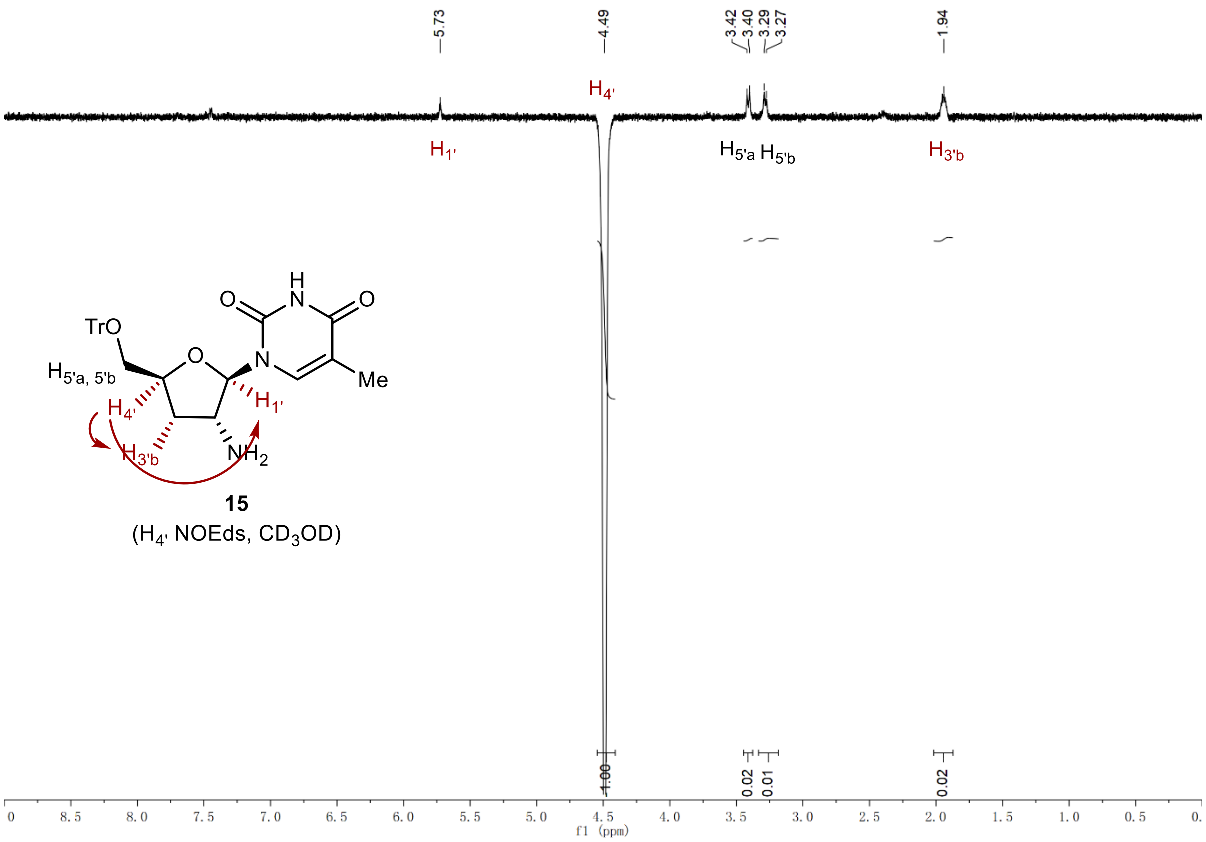

Supplementary Figure 237. $H_{4'}$ NOEds (600M, CD_3OD) of compound **15.**

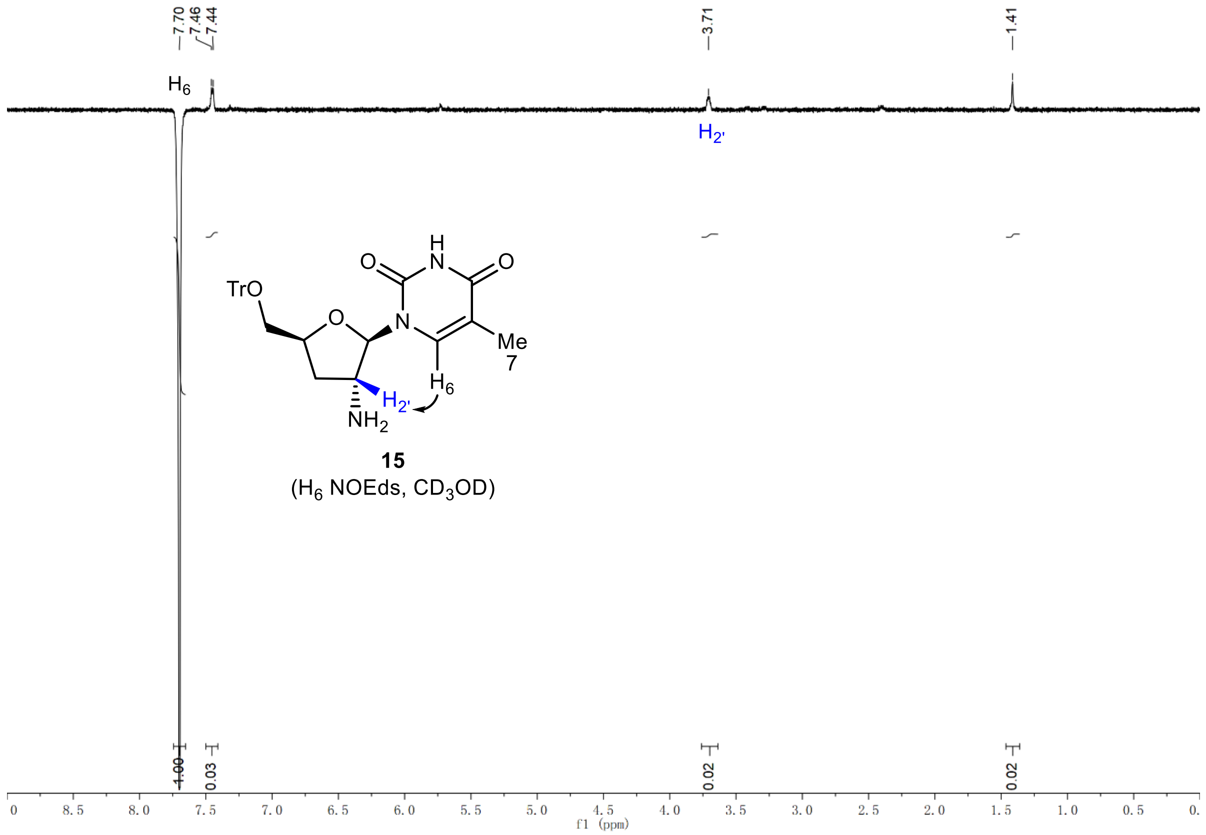

Supplementary Figure 238. H_6 NOEds (600M, CD_3OD) of compound **15.**

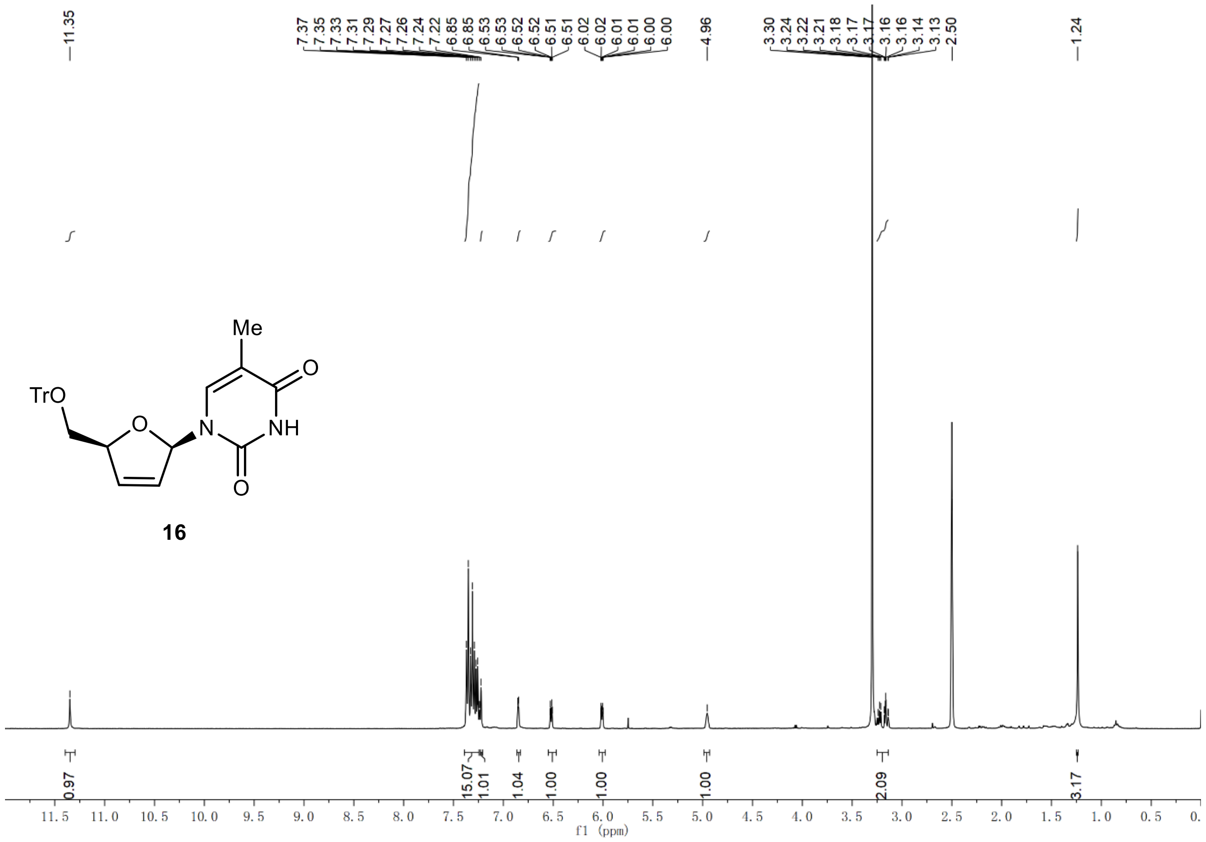

Supplementary Figure 239. ^{1}H NMR (400M, $(CD_3)_2SO$) of compound **16.**

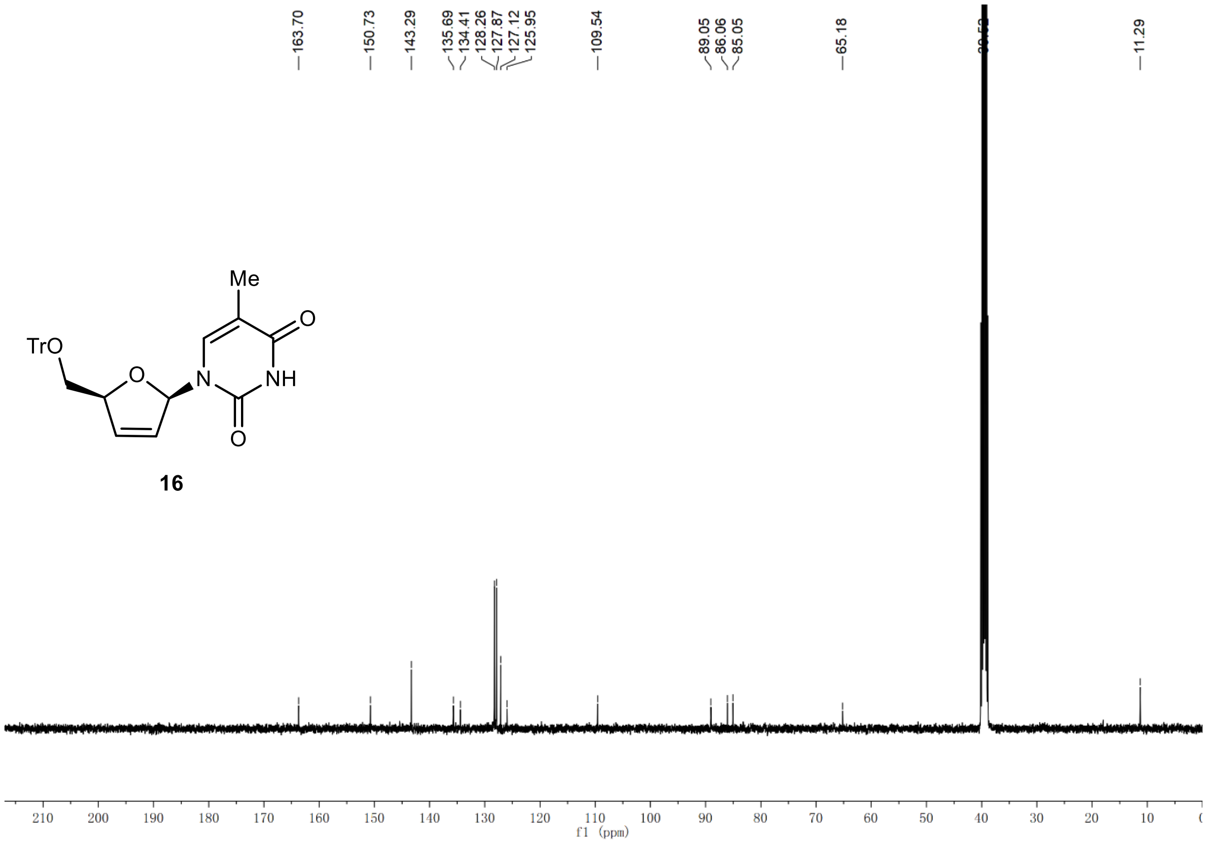

Supplementary Figure 240. ^{13}C NMR (100M, $(CD_3)_2SO$) of compound **16.**

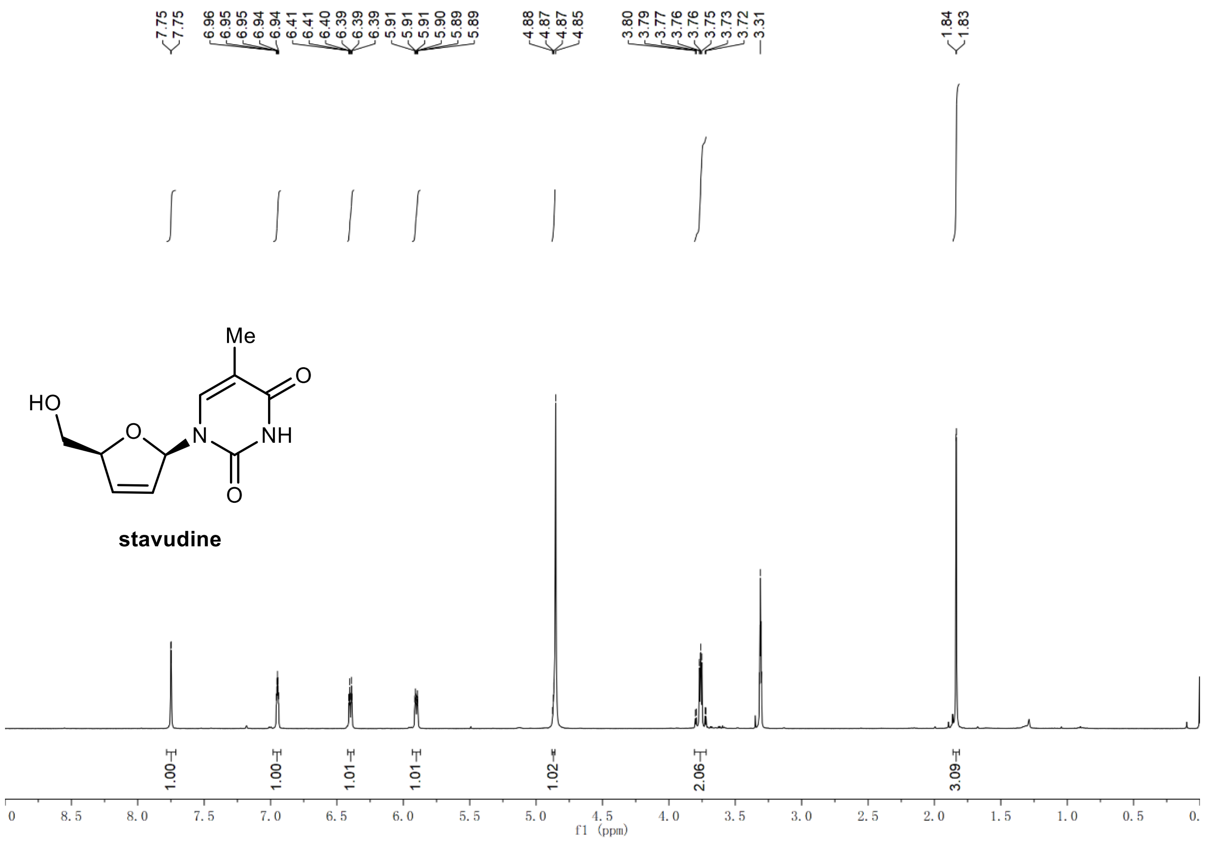

Supplementary Figure 241. ^{1}H NMR (400M, CD_3OD) of stavudine.

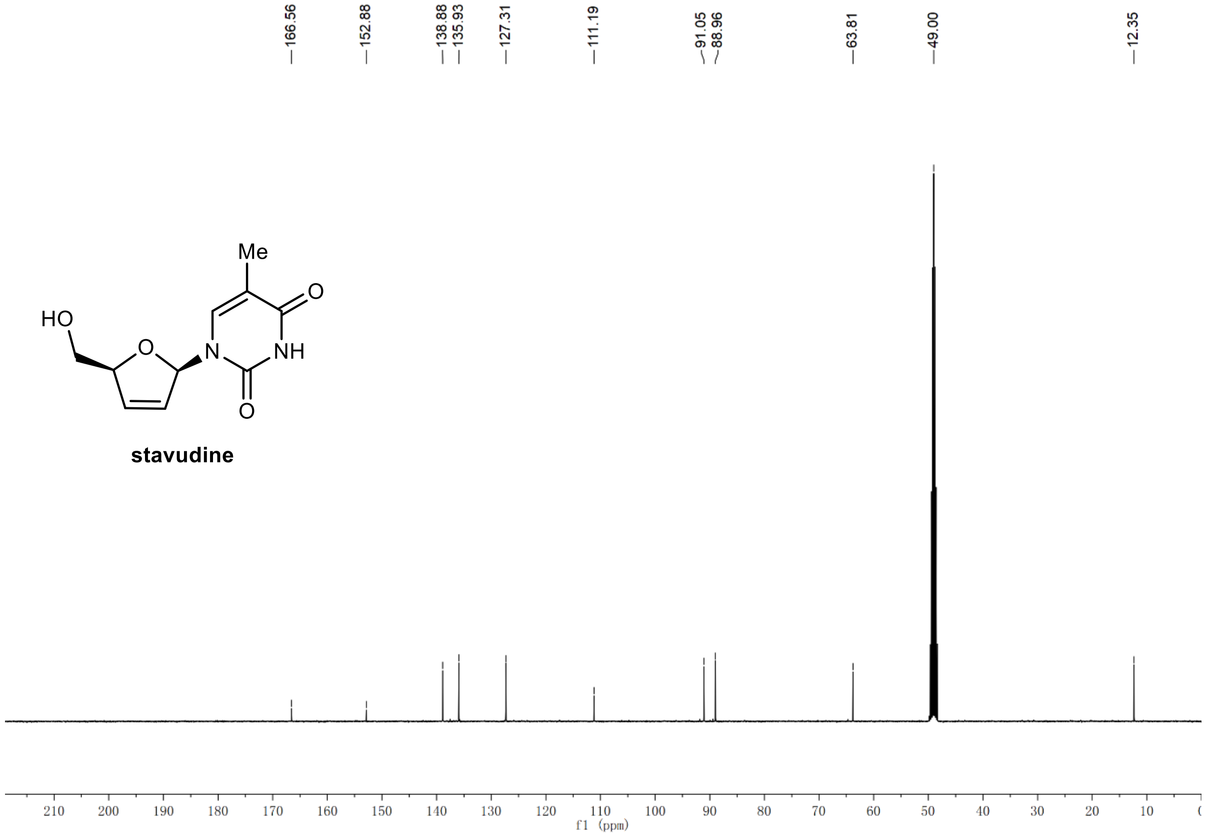

Supplementary Figure 242. ^{13}C NMR (100M, CD_3OD) of stavudine.

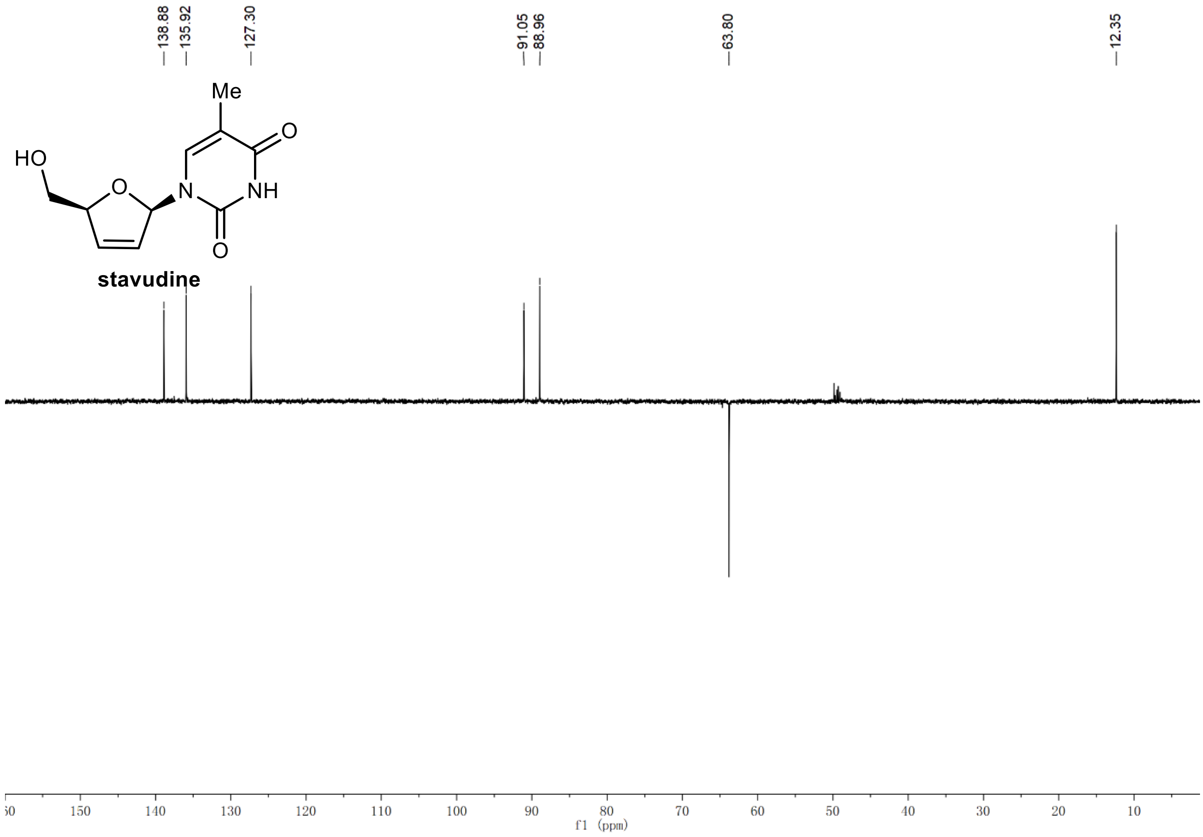

Supplementary Figure 243. DEPT 135° (100 M, CD_3OD) of stavudine.

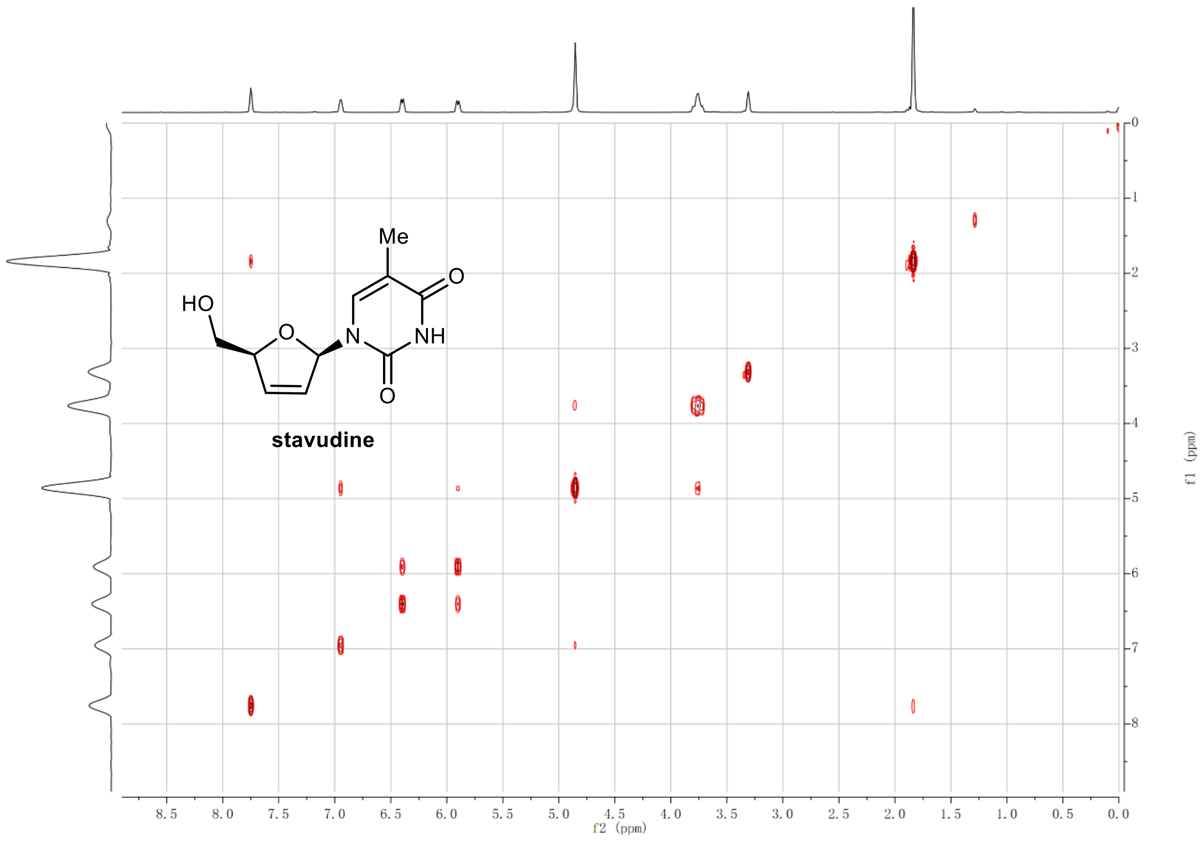

Supplementary Figure 244. ^1H-^1H COSY (400 M, CD_3OD) of stavudine.

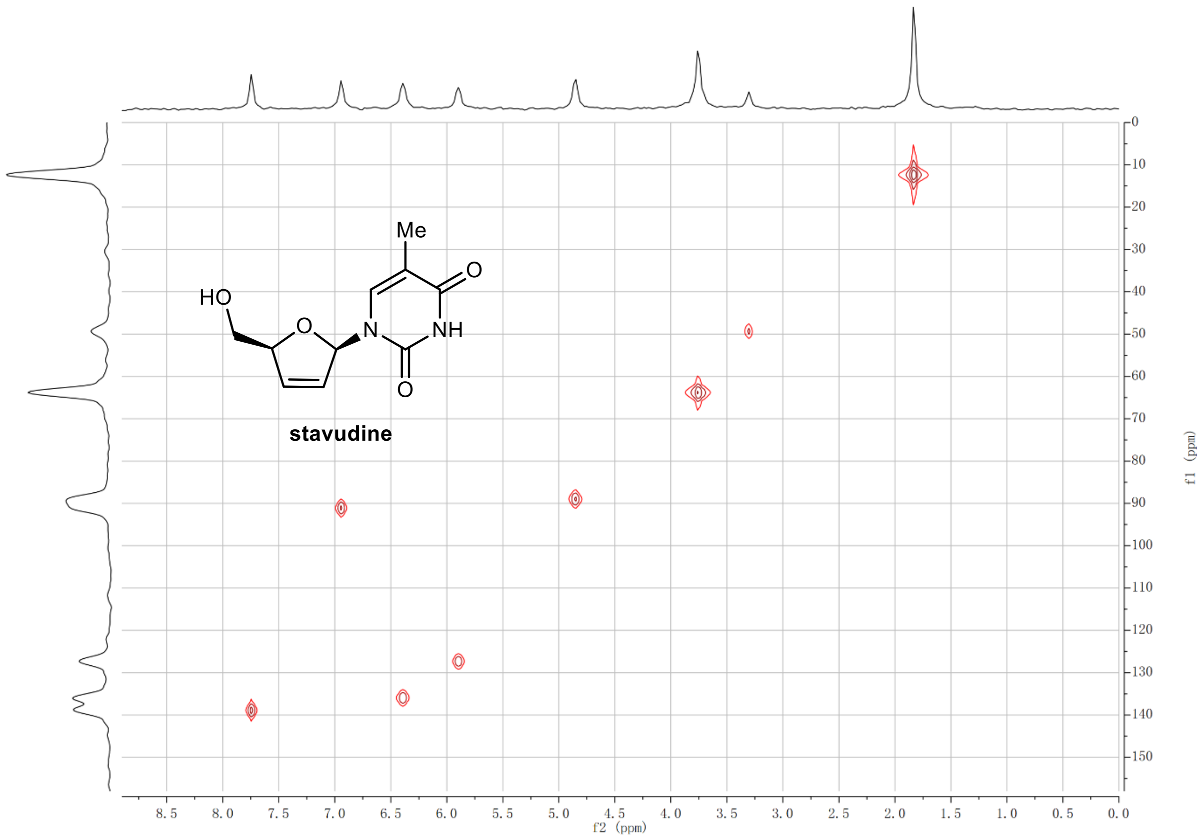

Supplementary Figure 245. ^{1}H-^{13}C HMQC (400M, CD_3OD) of stavudine.

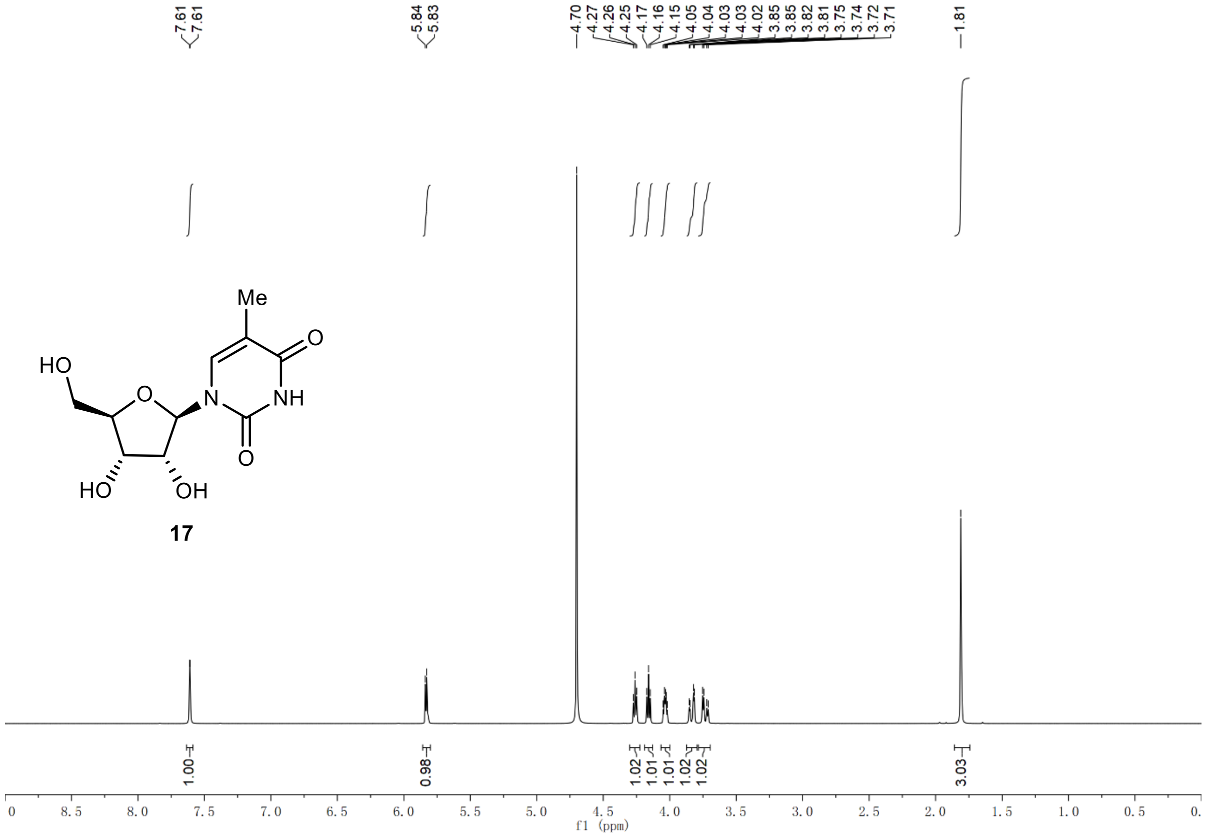

Supplementary Figure 246. ^{1}H NMR (400M, D_2O) of compound **17.**

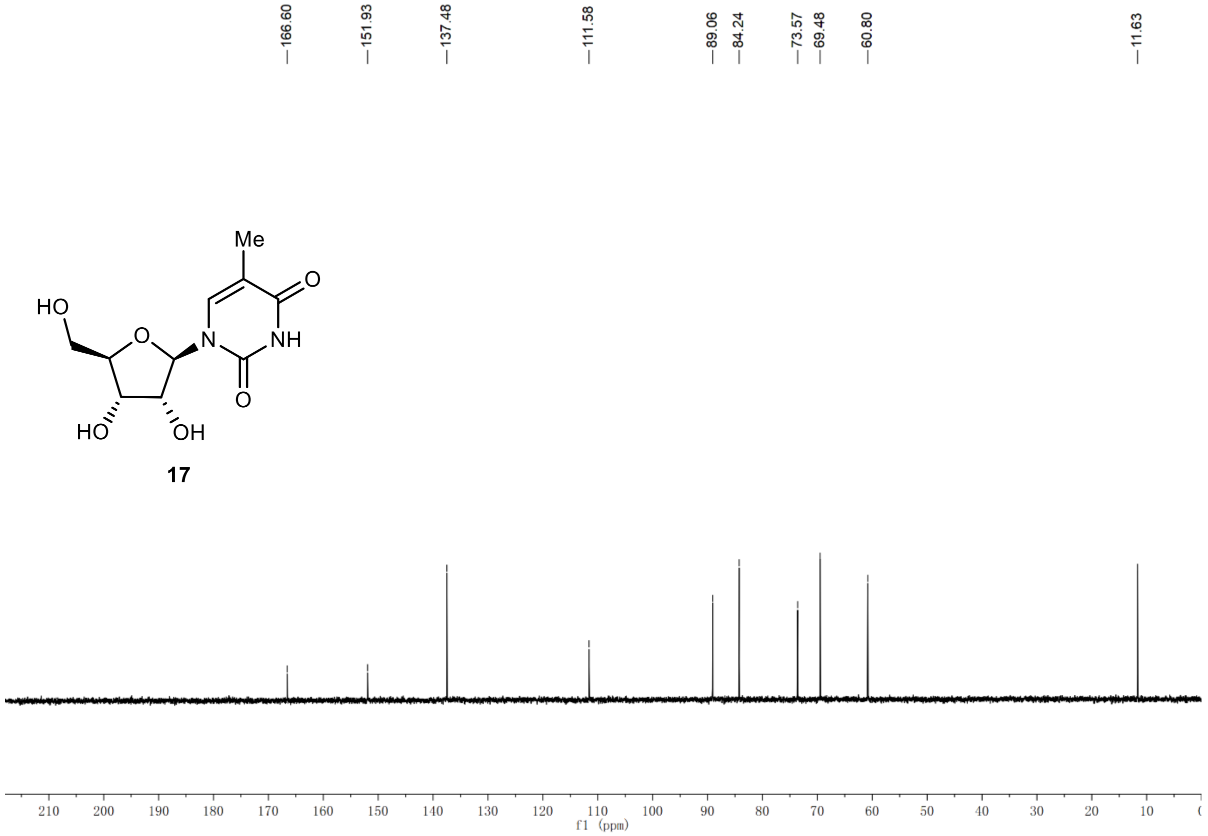

Supplementary Figure 247. ^{13}C NMR (100M, D_2O) of compound **17.**

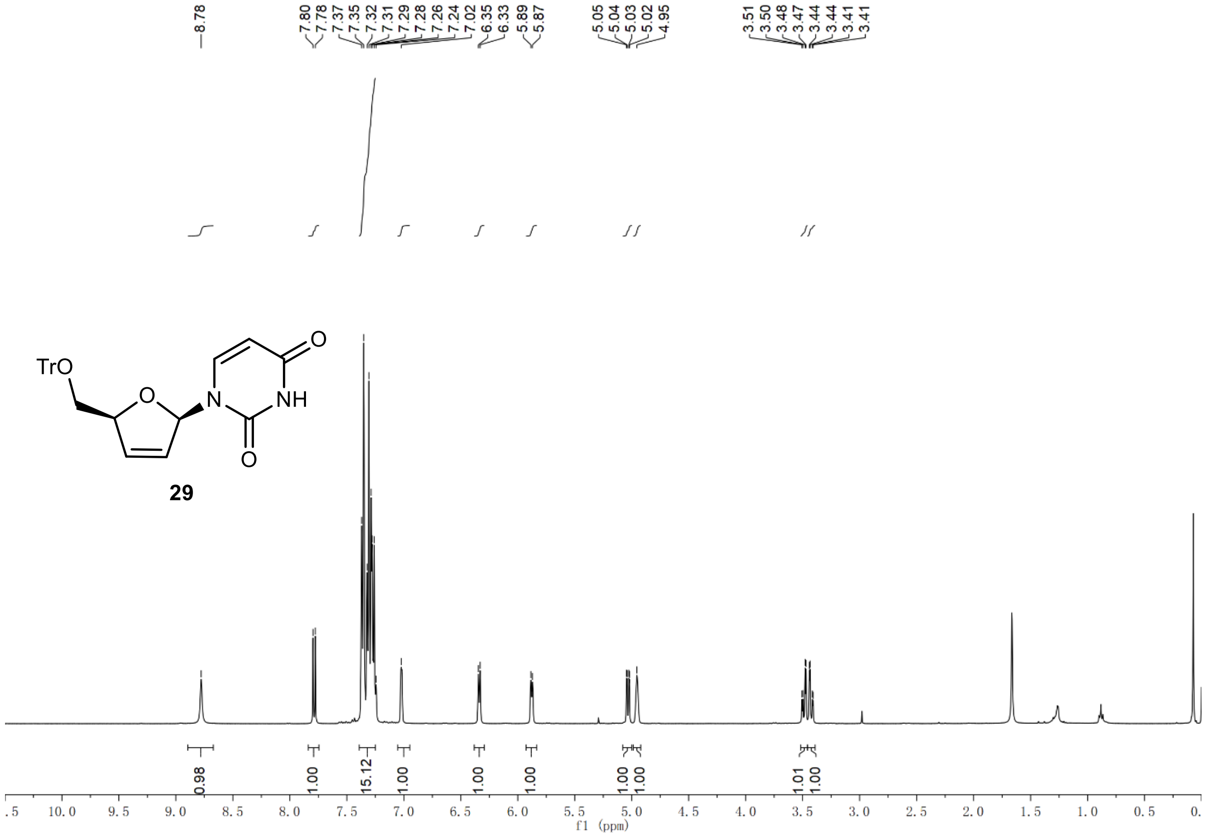

Supplementary Figure 248. ^{1}H NMR (400M, $CDCl_3$) of compound **29.**

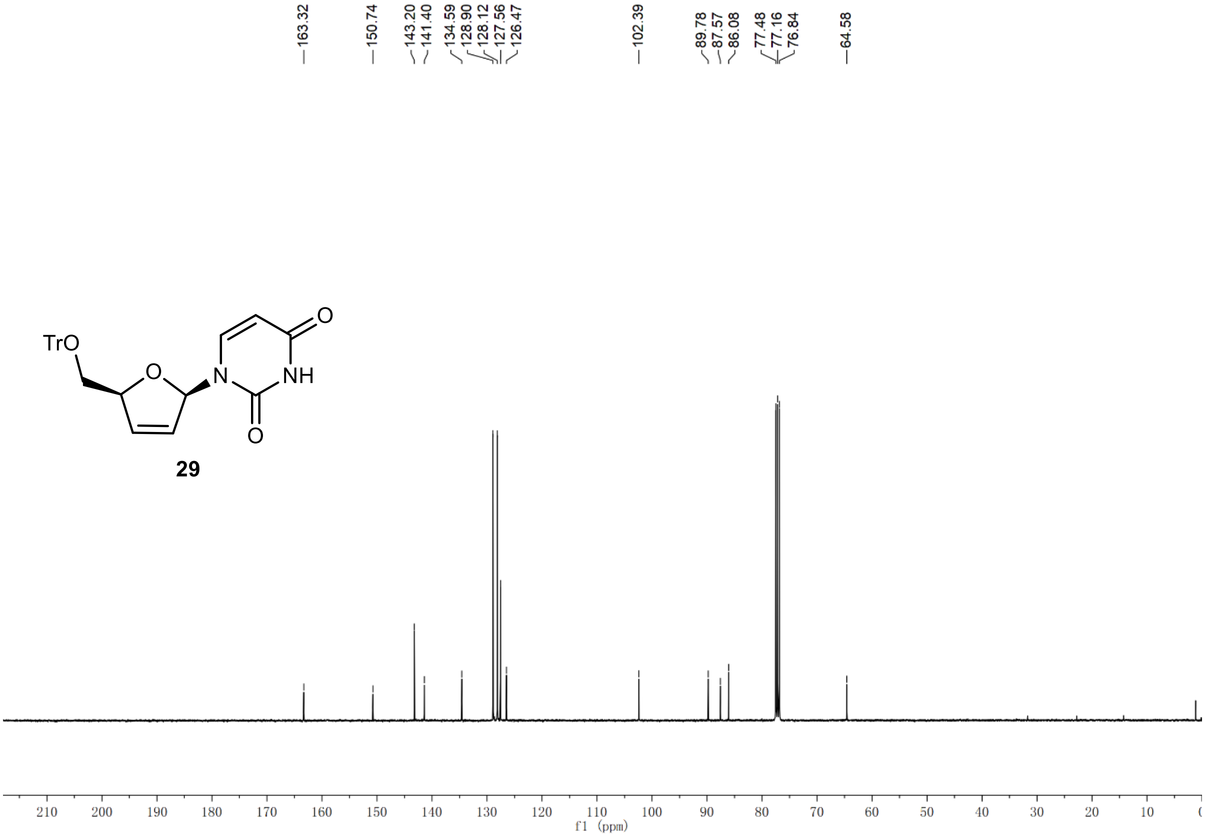

Supplementary Figure 249. ^{13}C NMR (100M, $CDCl_3$) of compound **29.**

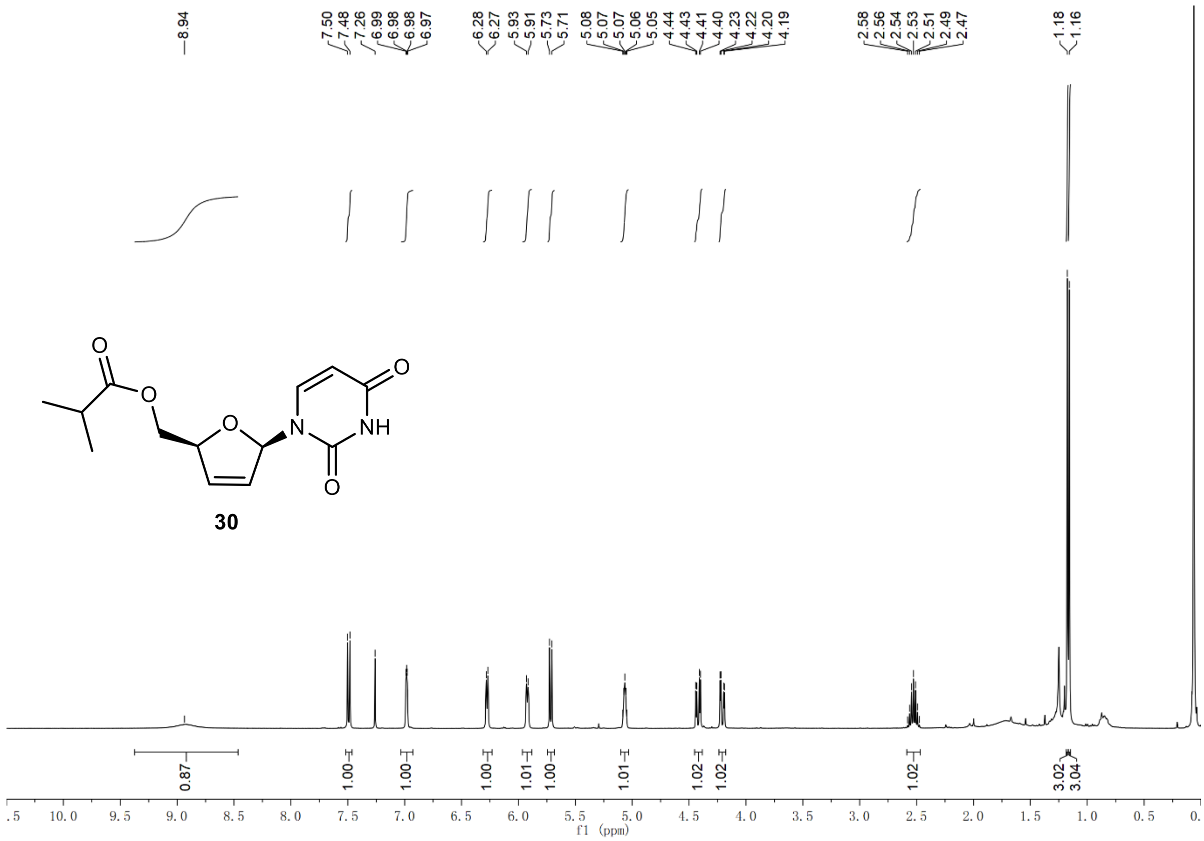

Supplementary Figure 250. ^{1}H NMR (400M, $CDCl_3$) of compound **30.**

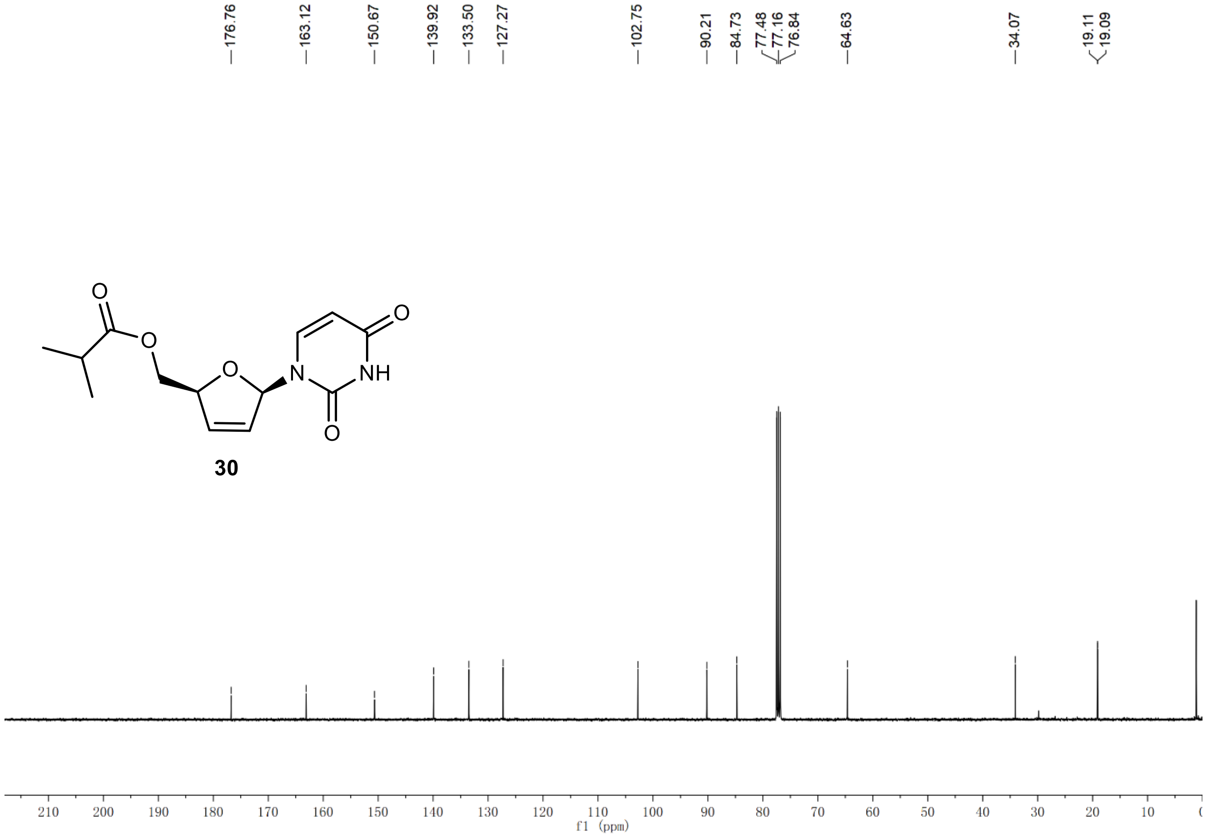

Supplementary Figure 251. ^{13}C NMR (100M, $CDCl_3$) of compound **30.**

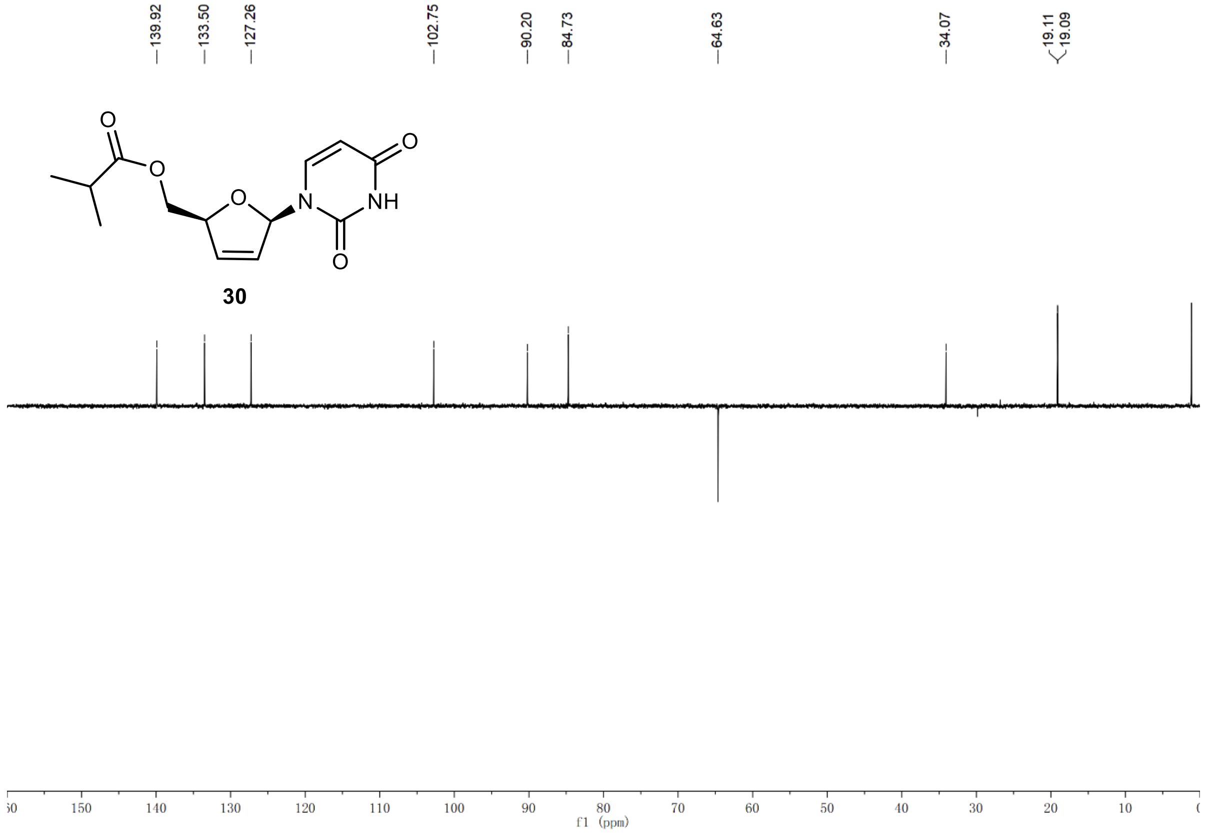

Supplementary Figure 252. DEPT 135° (100M, $CDCl_3$) of compound **30.**

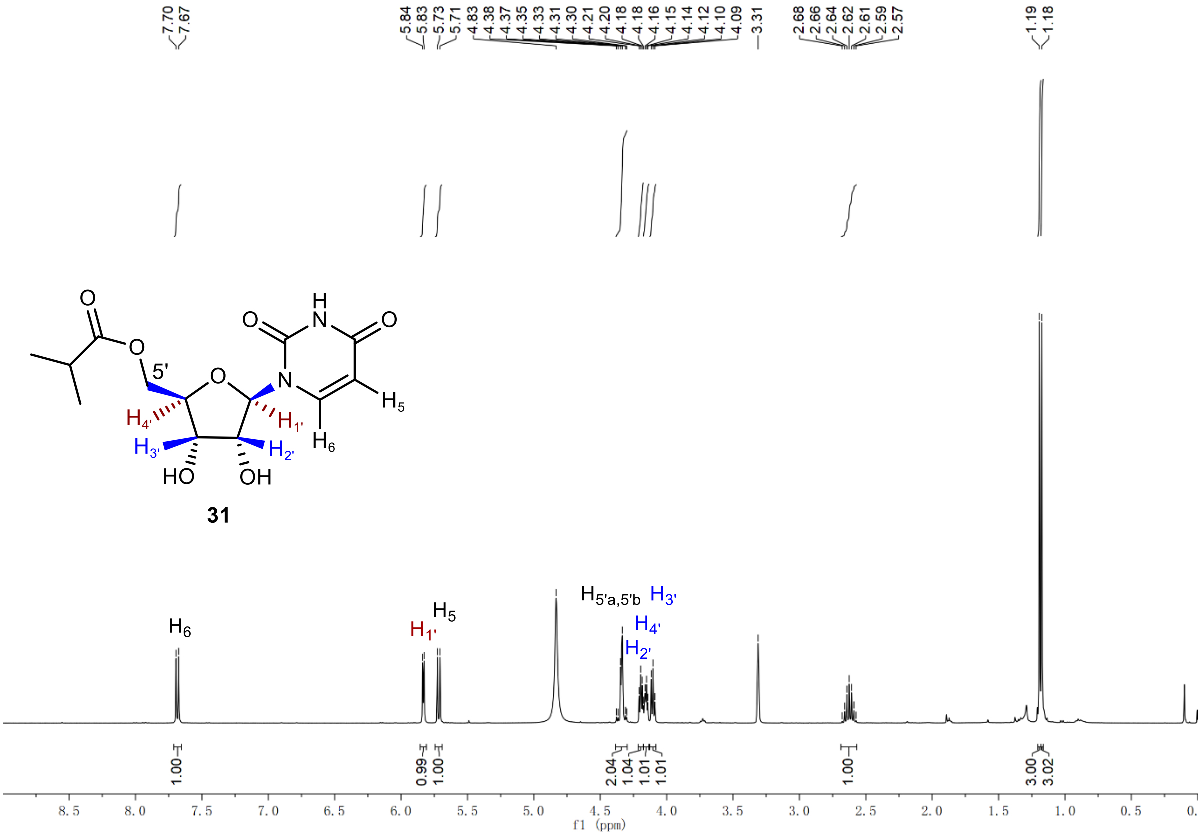

Supplementary Figure 253. ^{1}H NMR (400M, CD_3OD) of compound **31.**

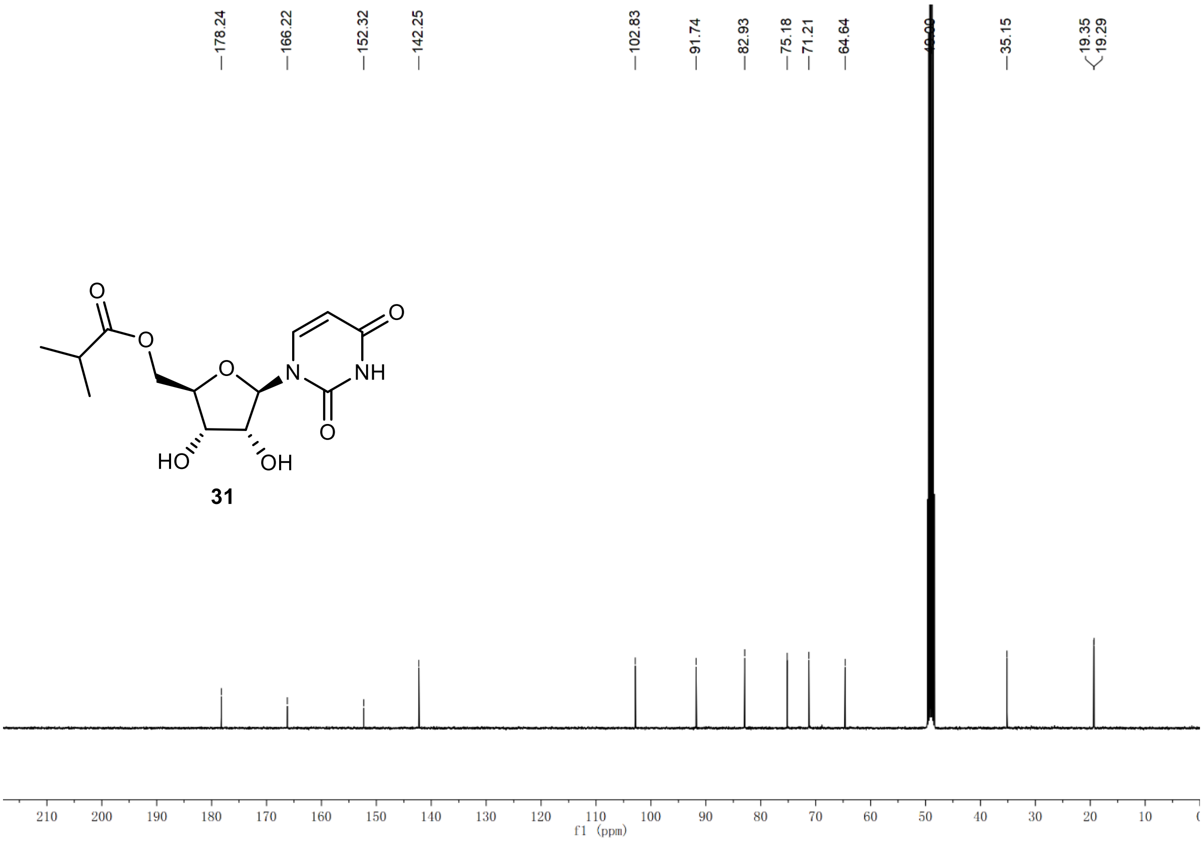

Supplementary Figure 254. ^{13}C NMR (100M, CD_3OD) of compound **31.**

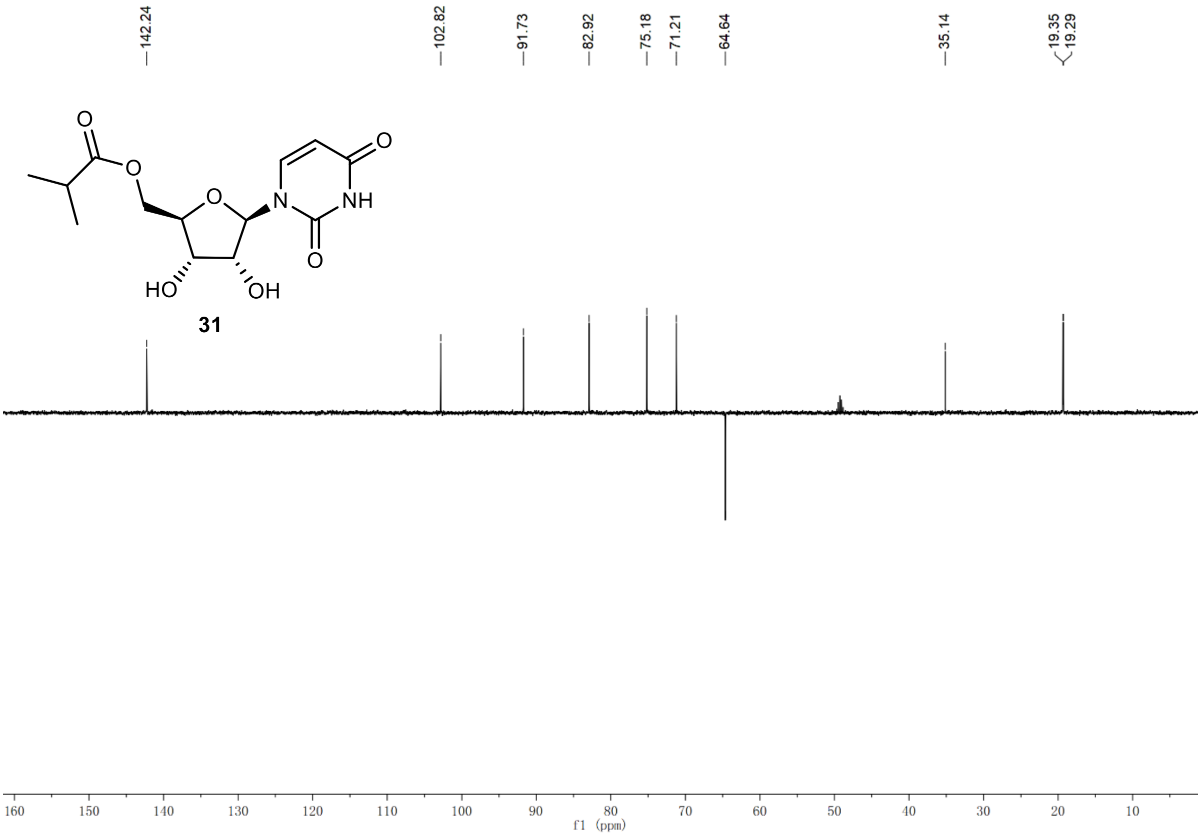

Supplementary Figure 255. DEPT 135° (100 M, CD_3OD) of compound **31.**

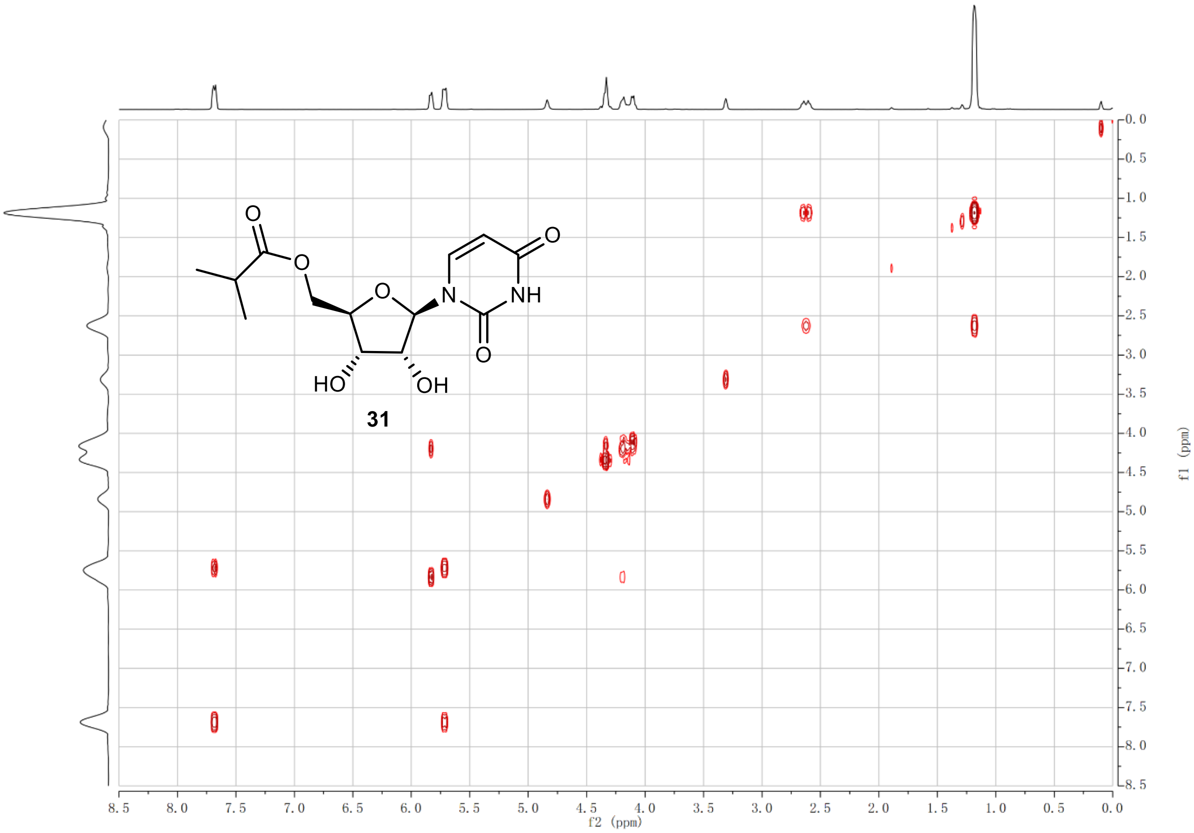

Supplementary Figure 256. ^1H-^1H COSY (400M, CD_3OD) of compound **31.**

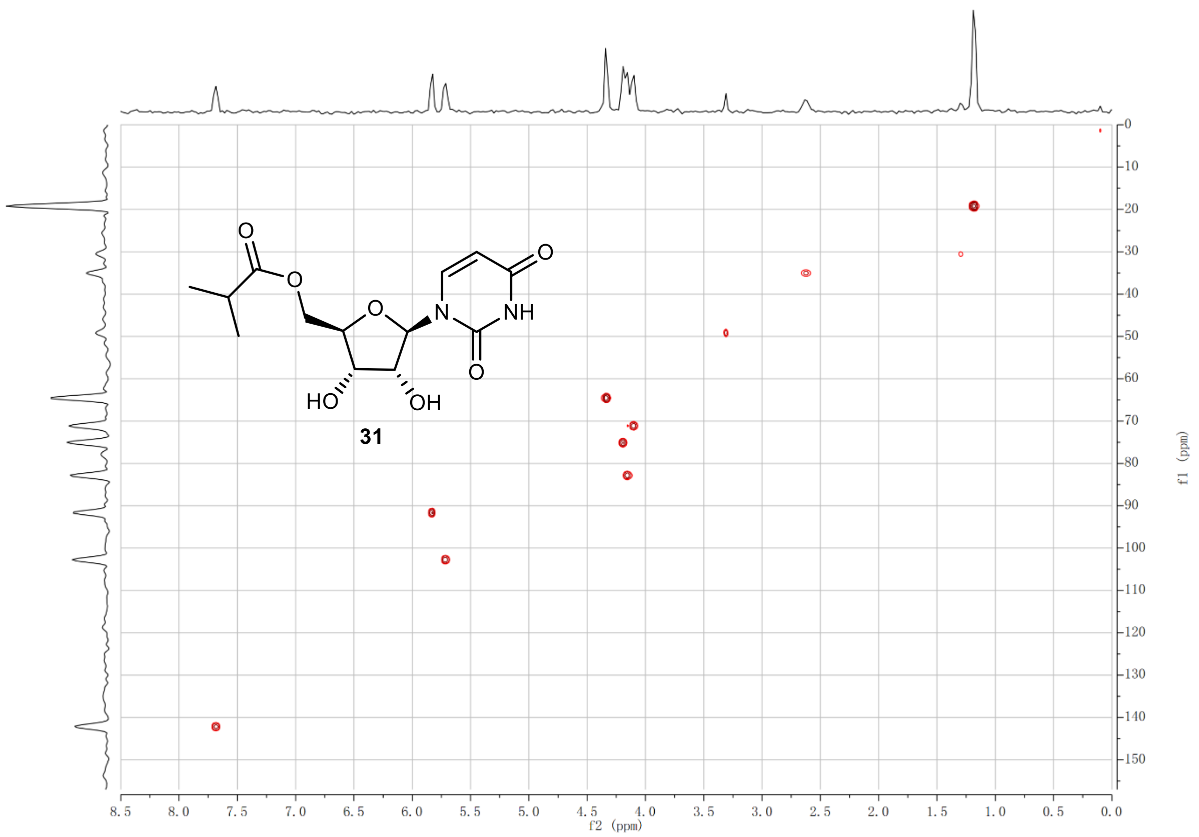

Supplementary Figure 257. ^{1}H-^{13}C HMQC (400M, CD_3OD) of compound **31.**

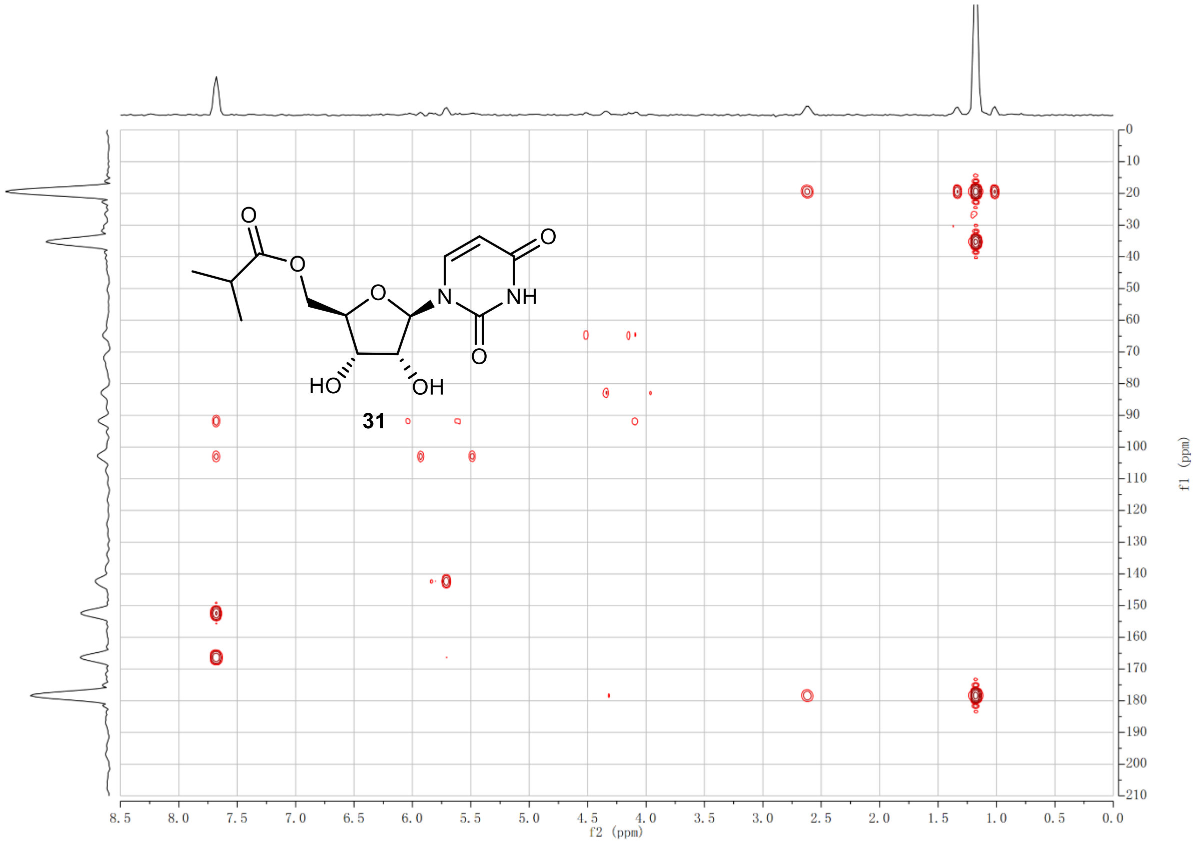

Supplementary Figure 258. ^{1}H-^{13}C HMBC (400M, CD_3OD) of compound **31.**

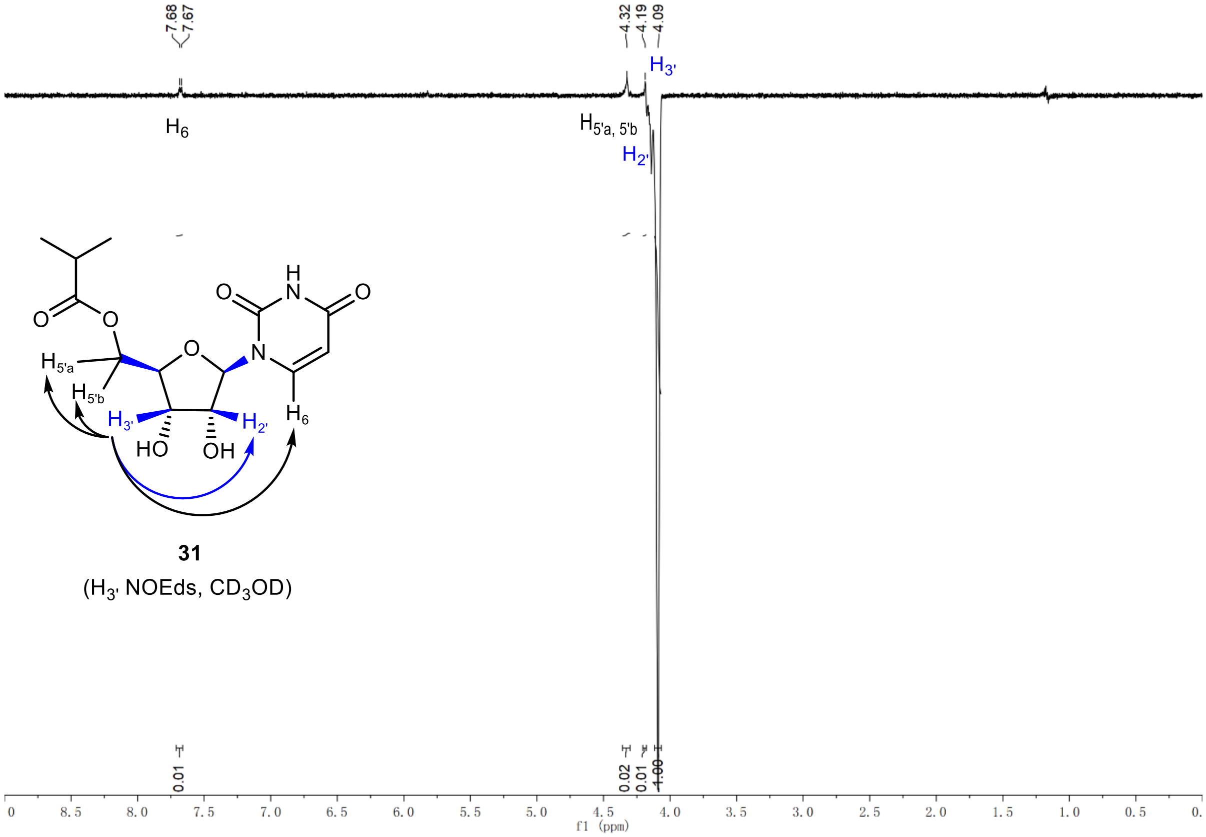

Supplementary Figure 259. $H_{3'}$ NOEds (600M, CD_3OD) of compound **31.**

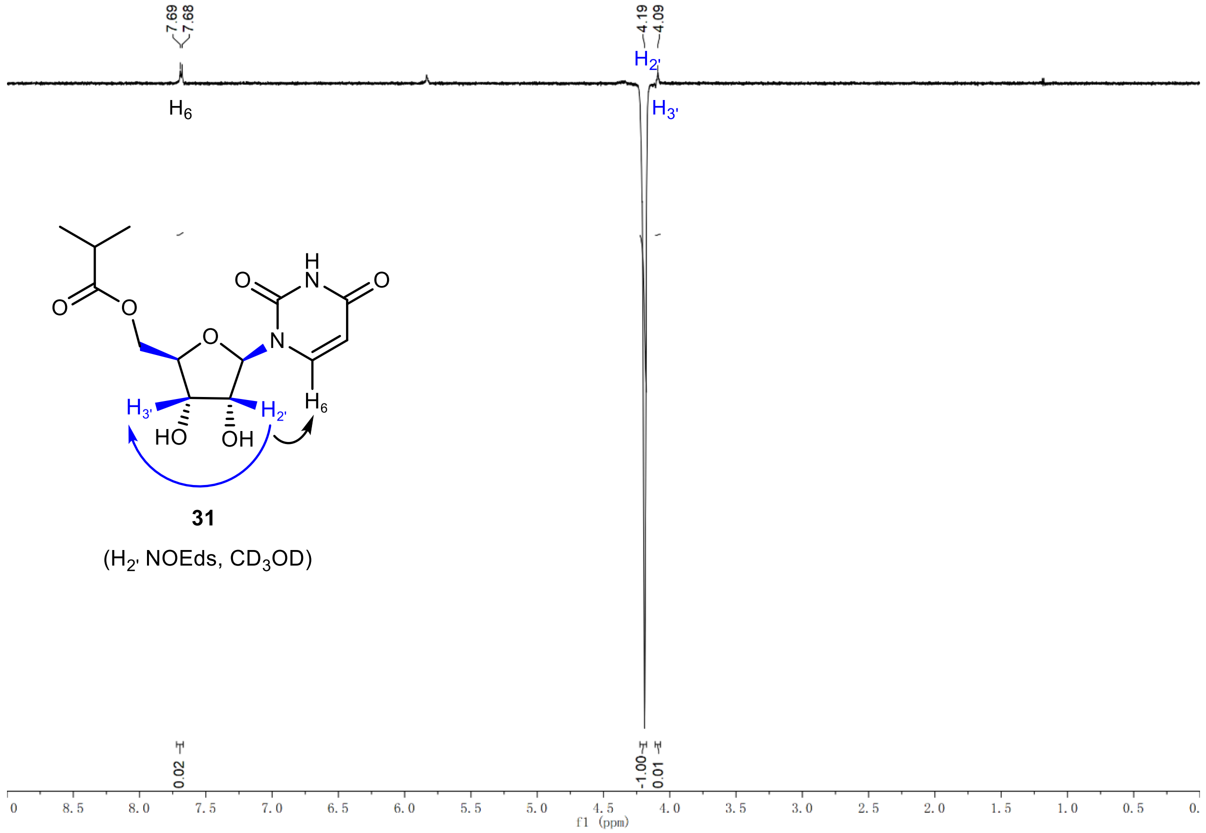

Supplementary Figure 260. $H_{2'}$ NOEds (600M, CD_3OD) of compound **31.**

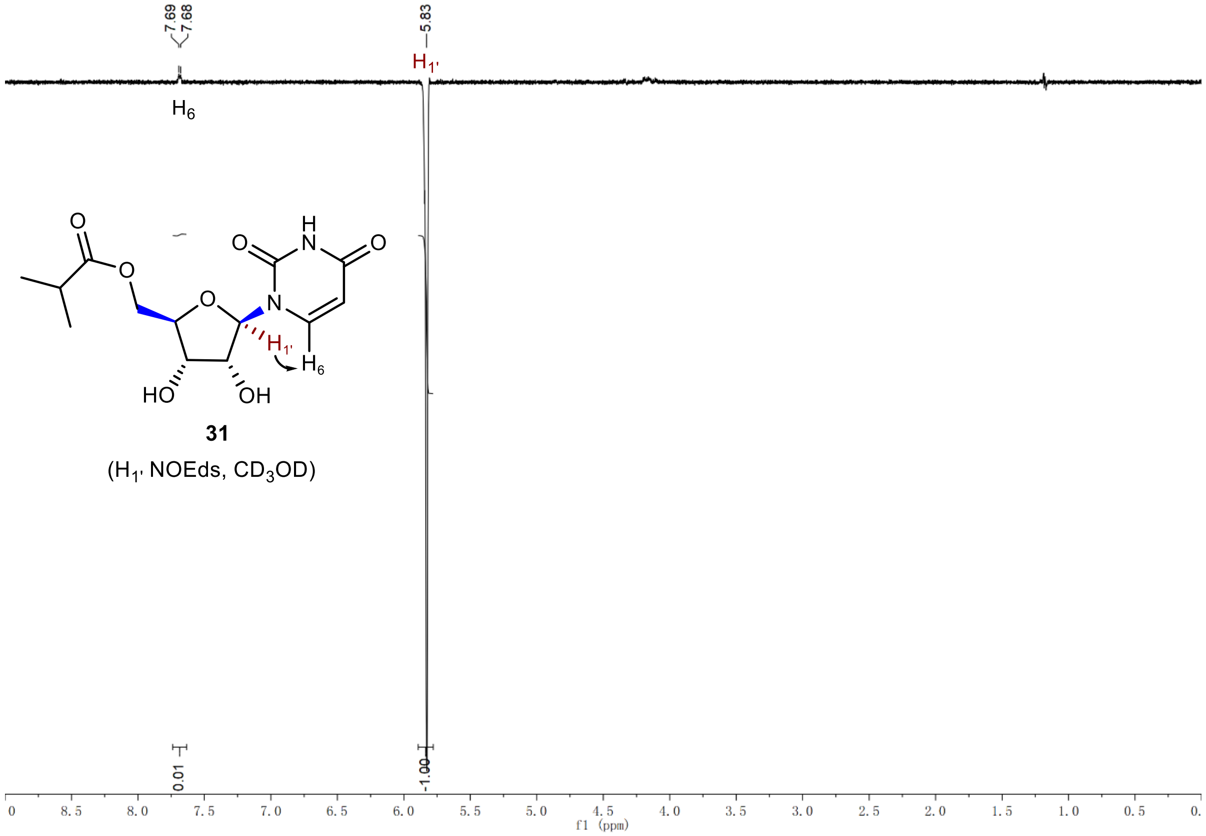

Supplementary Figure 261. $H_{1'}$ NOEds (600M, CD_3OD) of compound **31.**

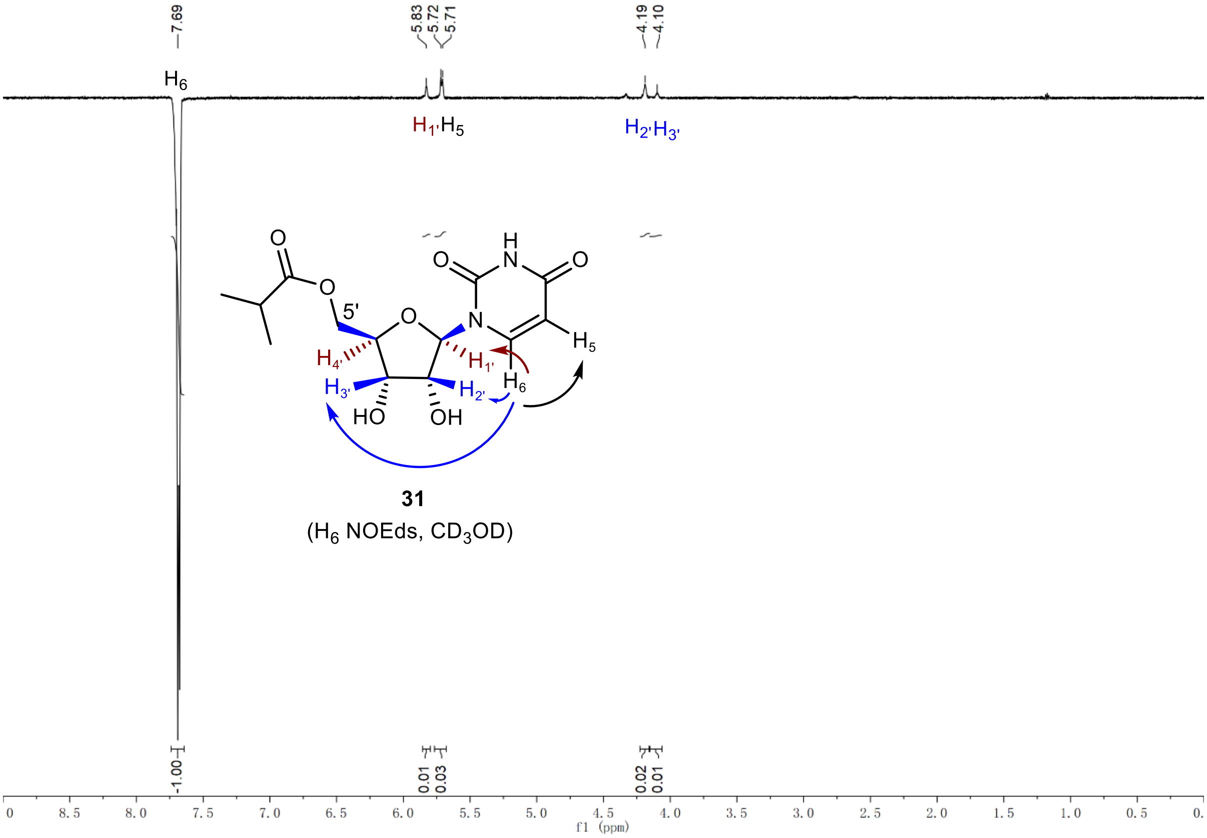

Supplementary Figure 262. H_6 NOEds (600M, CD_3OD) of compound **31.**

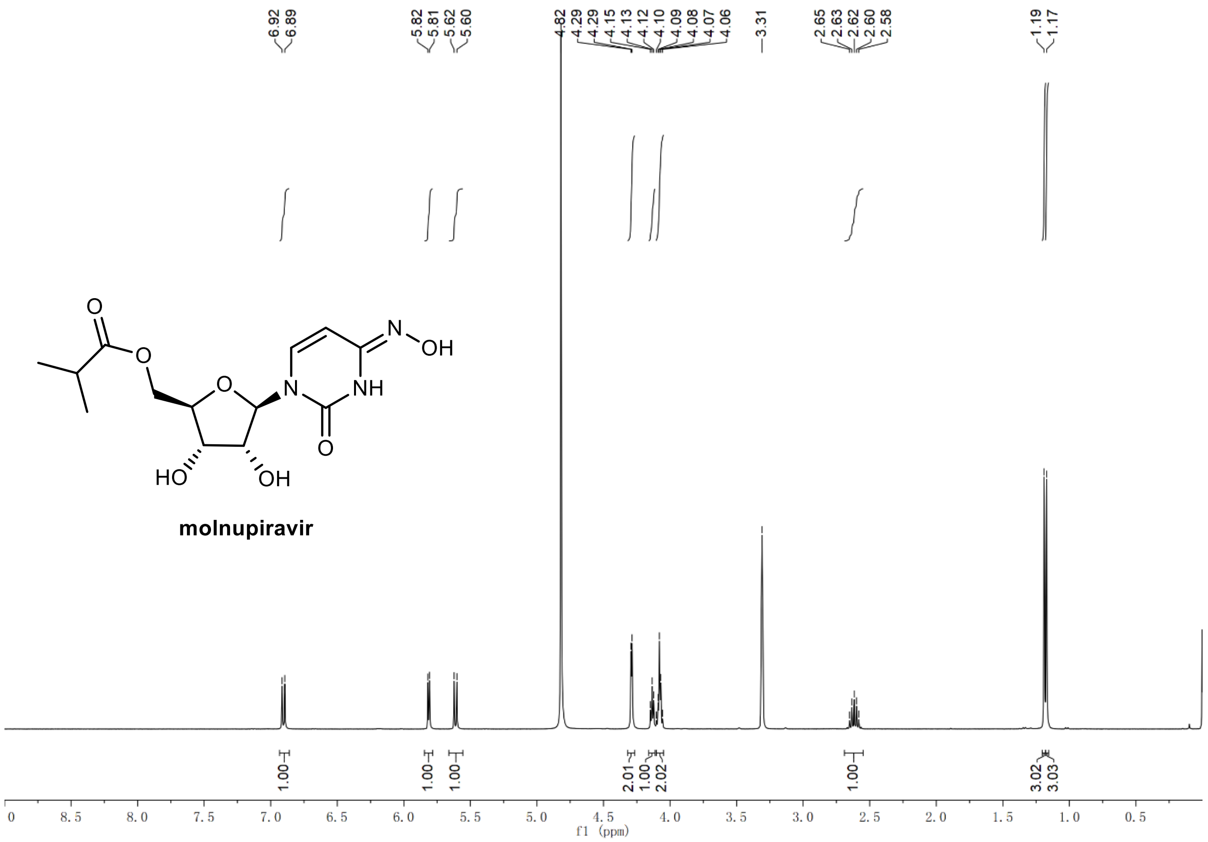

Supplementary Figure 263. ^{1}H NMR (400M, CD_3OD) of molnupiravir.

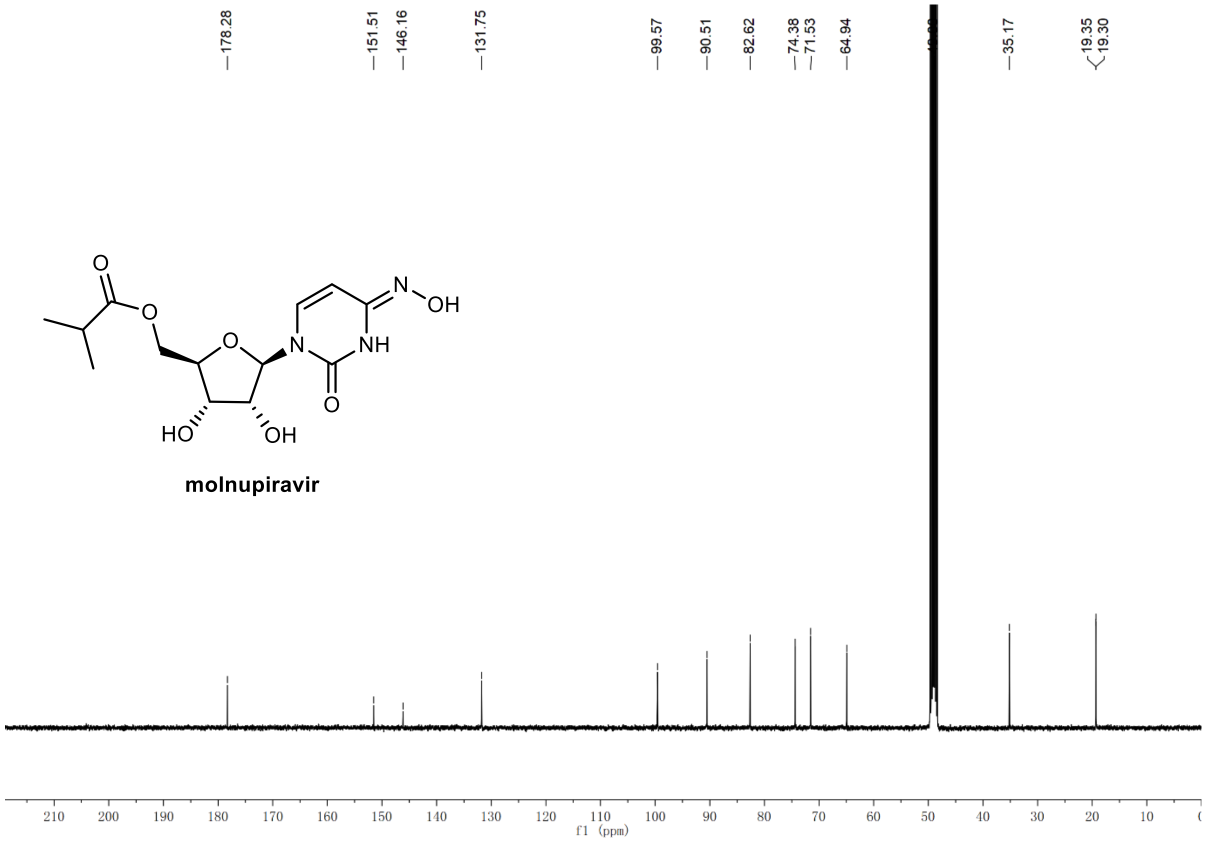

Supplementary Figure 264. ^{13}C NMR (100M, CD_3OD) of molnupiravir.

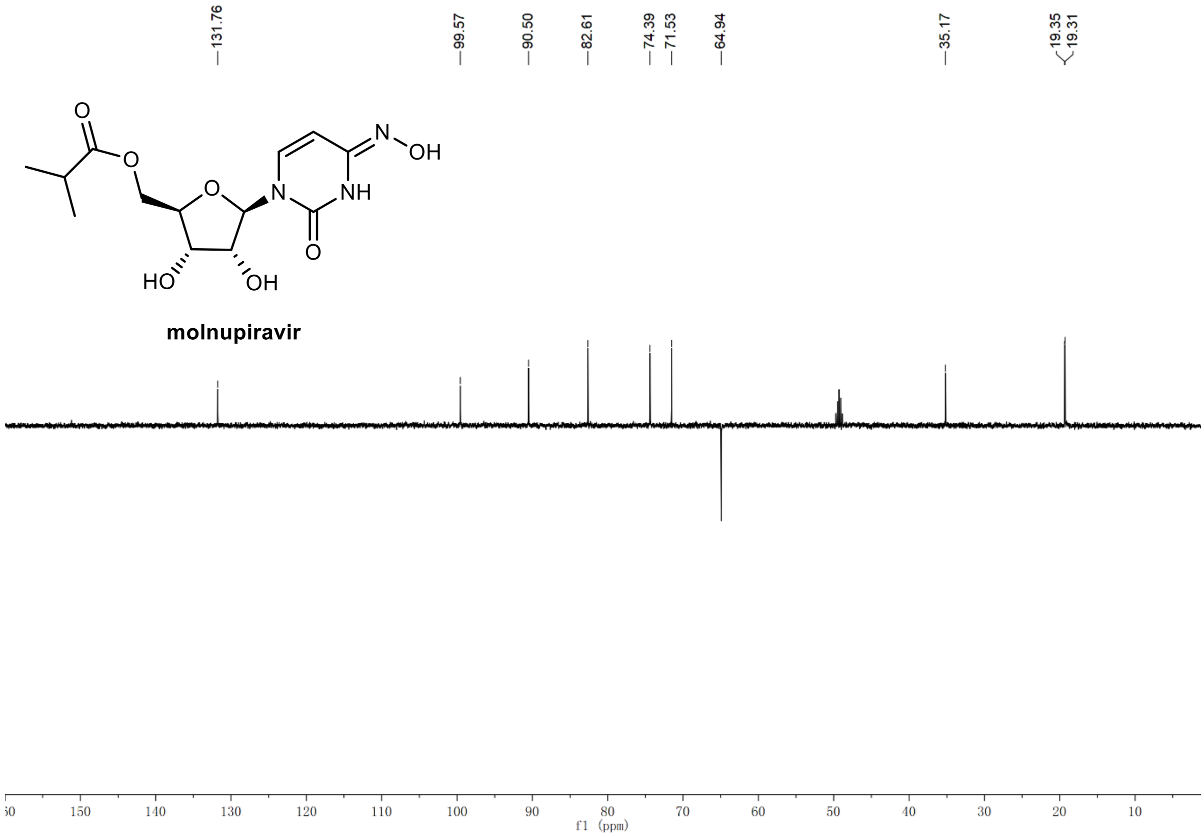

Supplementary Figure 265. DEPT 135° (100M, CD_3OD) of molnupiravir.

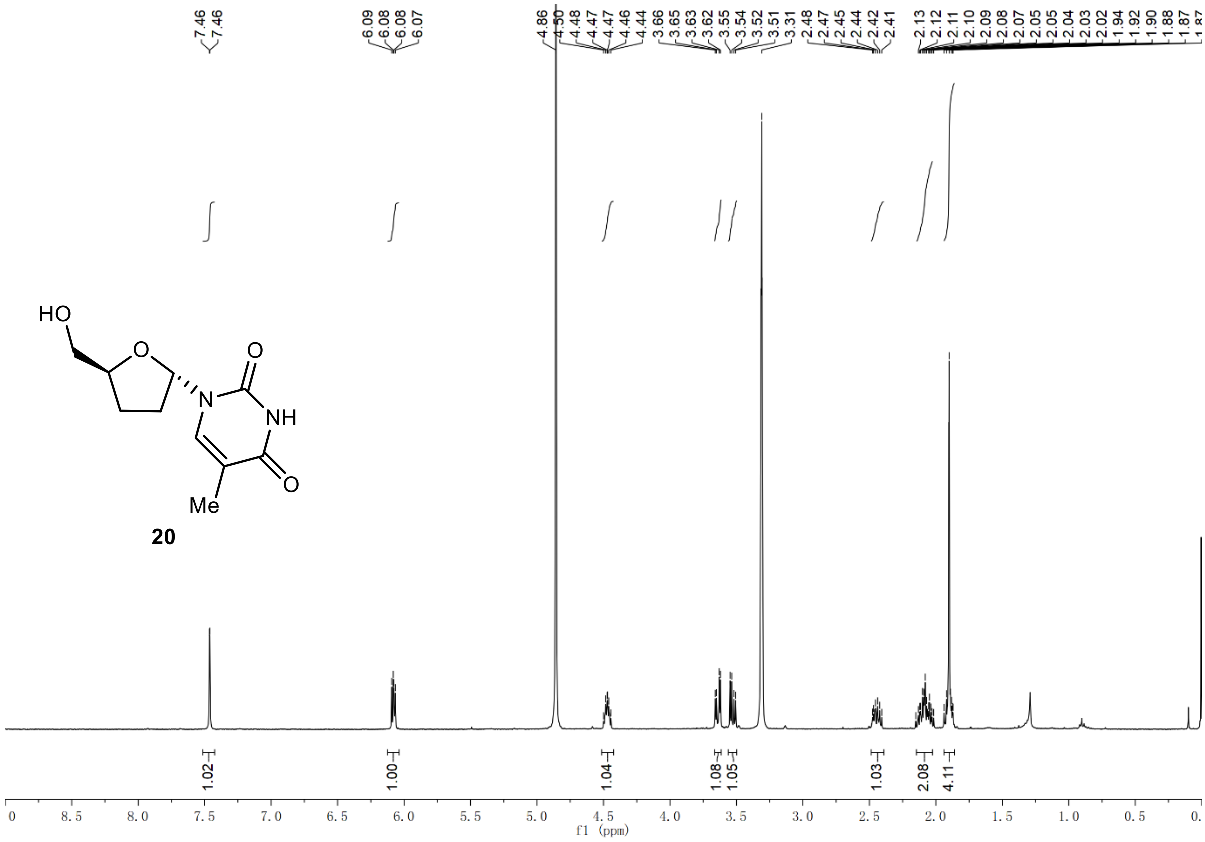

Supplementary Figure 266. ^1H NMR (400M, CD_3OD) of compound **20.**

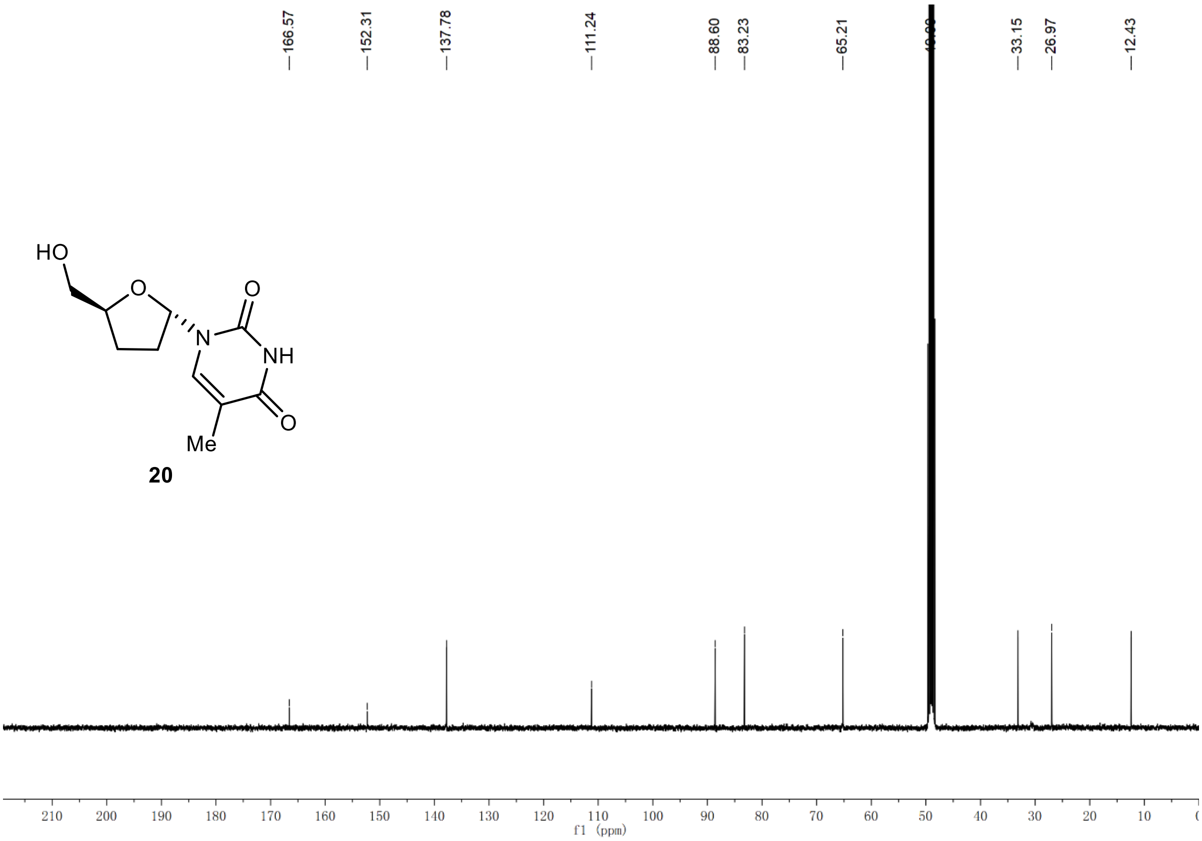

Supplementary Figure 267. ^{13}C NMR (100M, CD_3OD) of compound **20.**

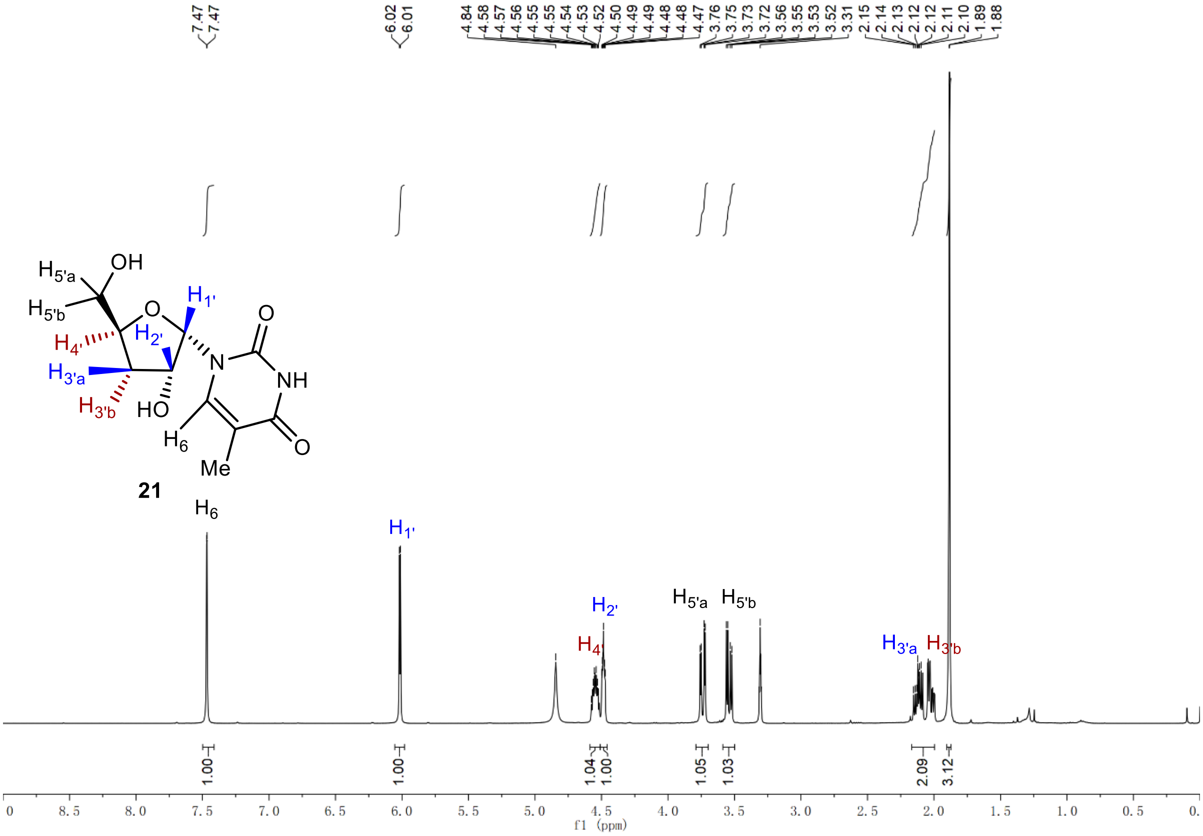

Supplementary Figure 268. ^{1}H NMR (400M, CD_3OD) of compound **21.**

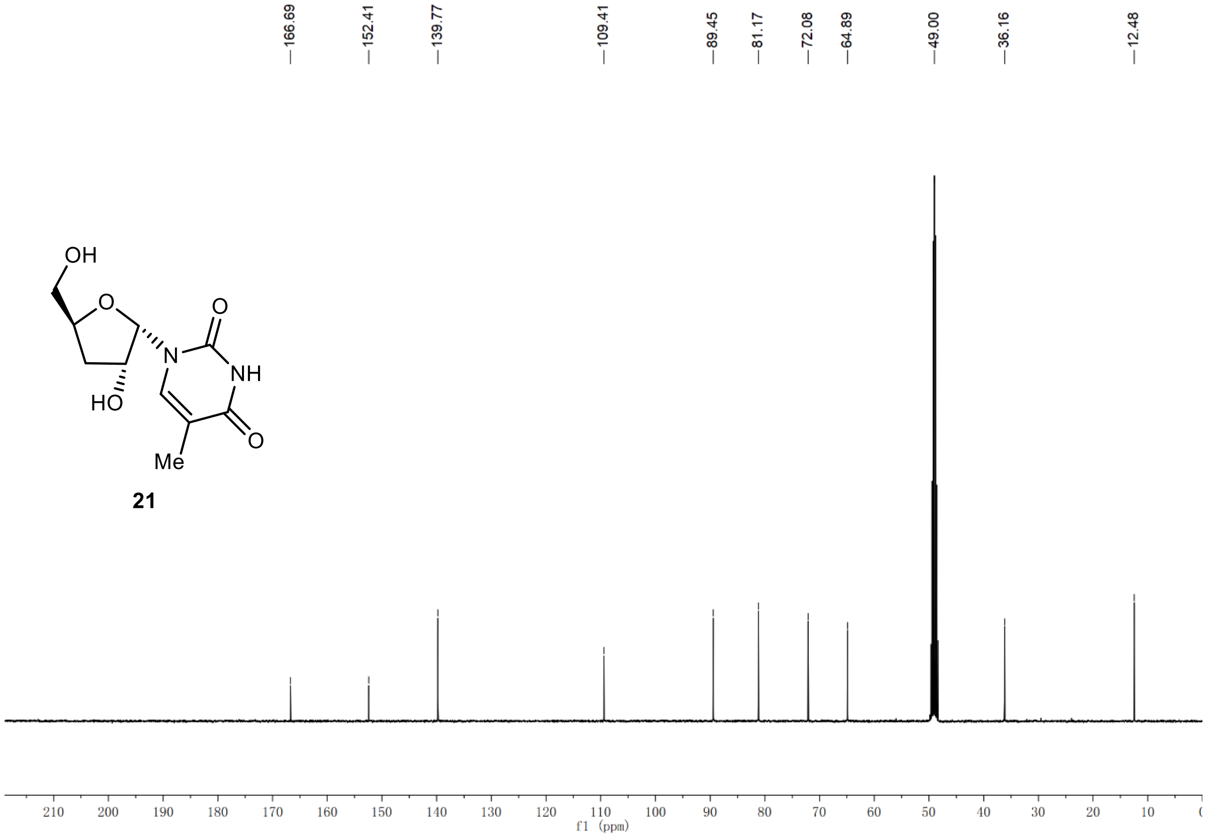

Supplementary Figure 269. ^{13}C NMR (100M, CD_3OD) of compound **21.**

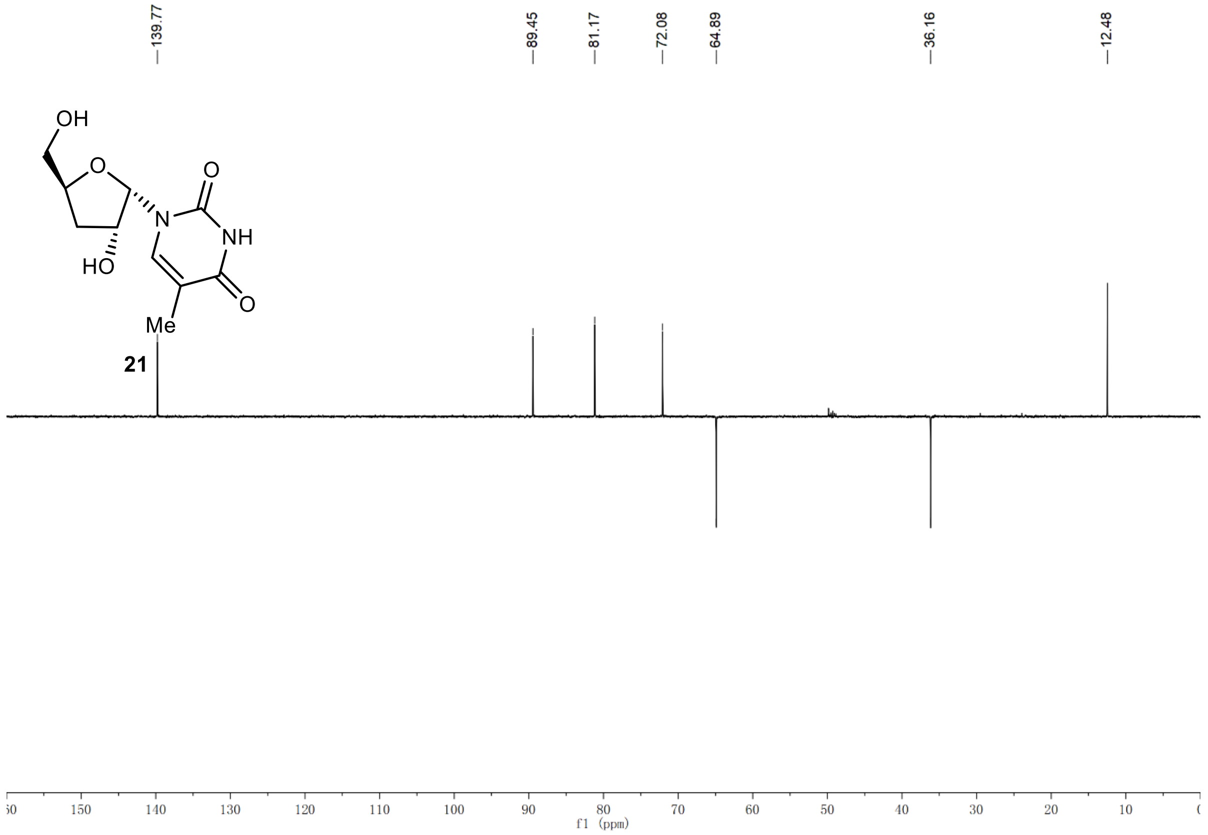

Supplementary Figure 270. DEPT 135° (100 M, CD_3OD) of compound **21.**

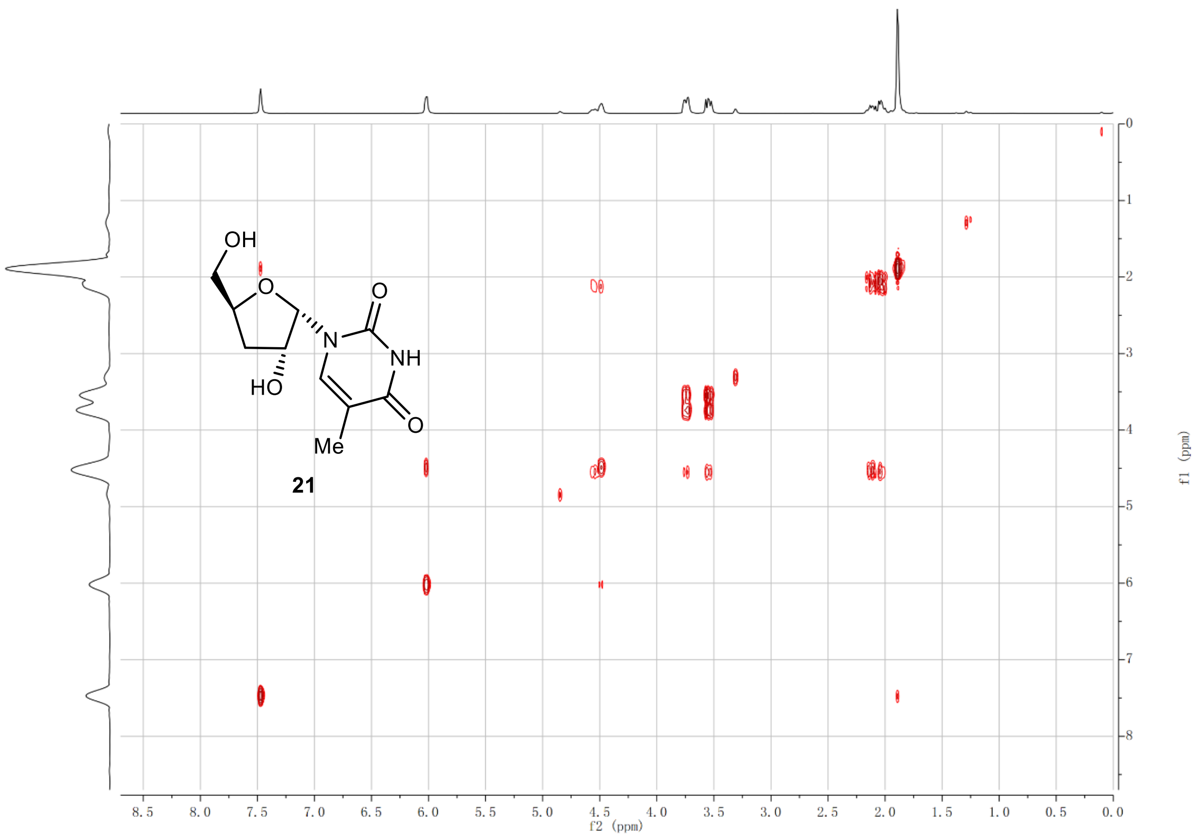

Supplementary Figure 271. ^{1}H-^{1}H COSY (400M, CD_3OD) of compound **21.**

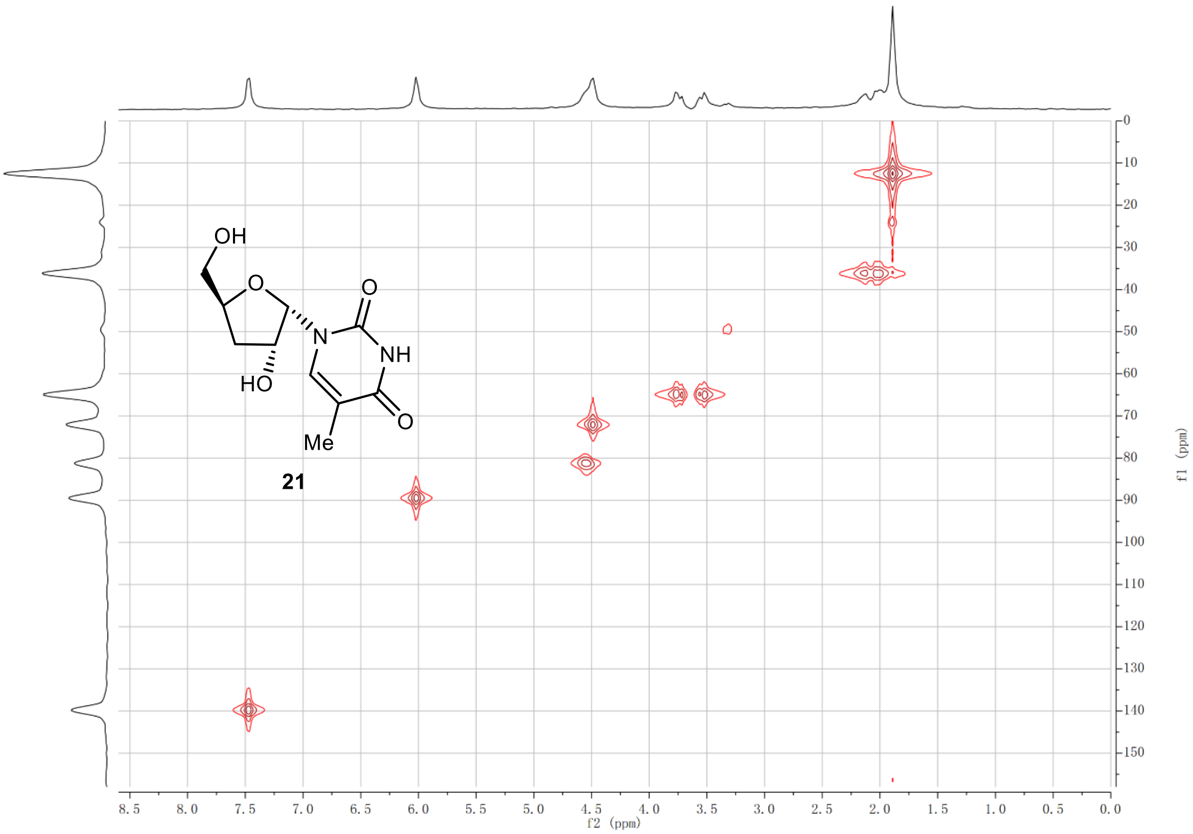

Supplementary Figure 272. ^{1}H-^{13}C HMQC (400M, CD_3OD) of compound **21.**

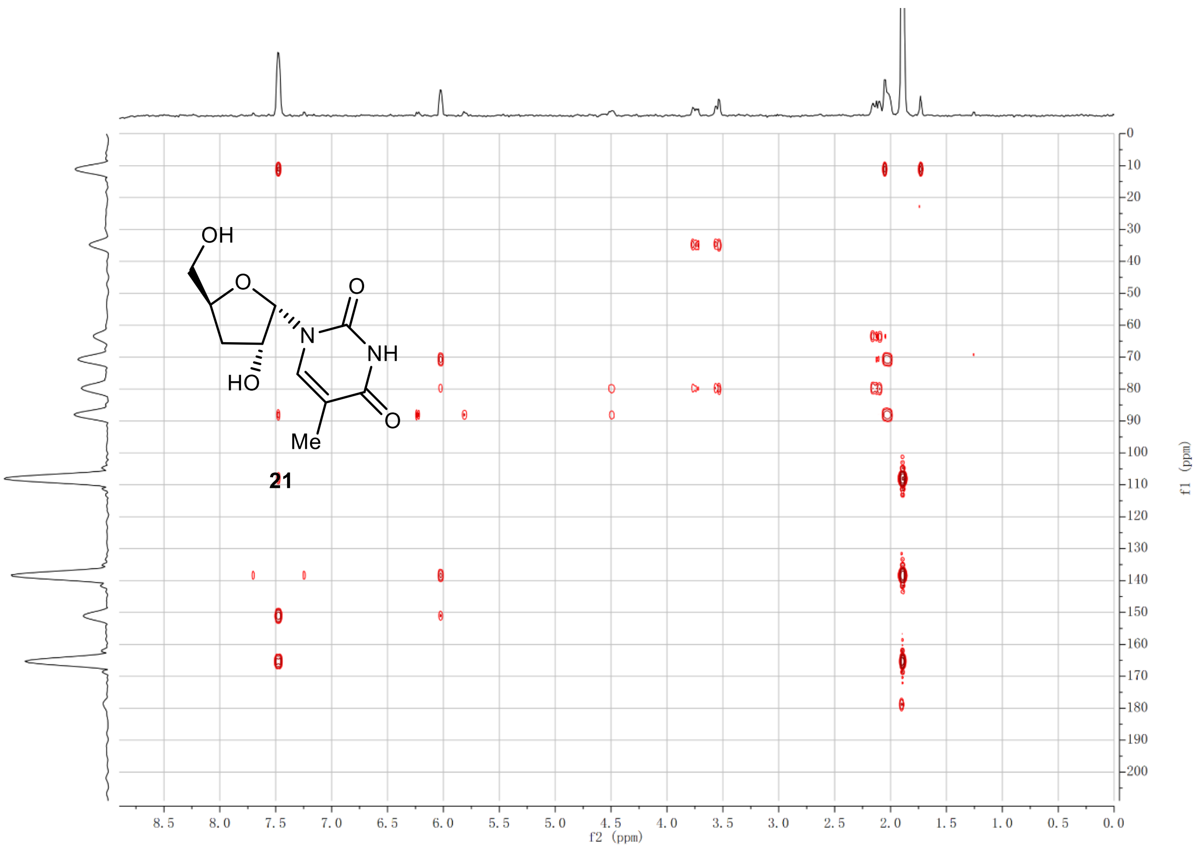

Supplementary Figure 273. ^{1}H-^{13}C HMBC (400M, CD_3OD) of compound **21.**

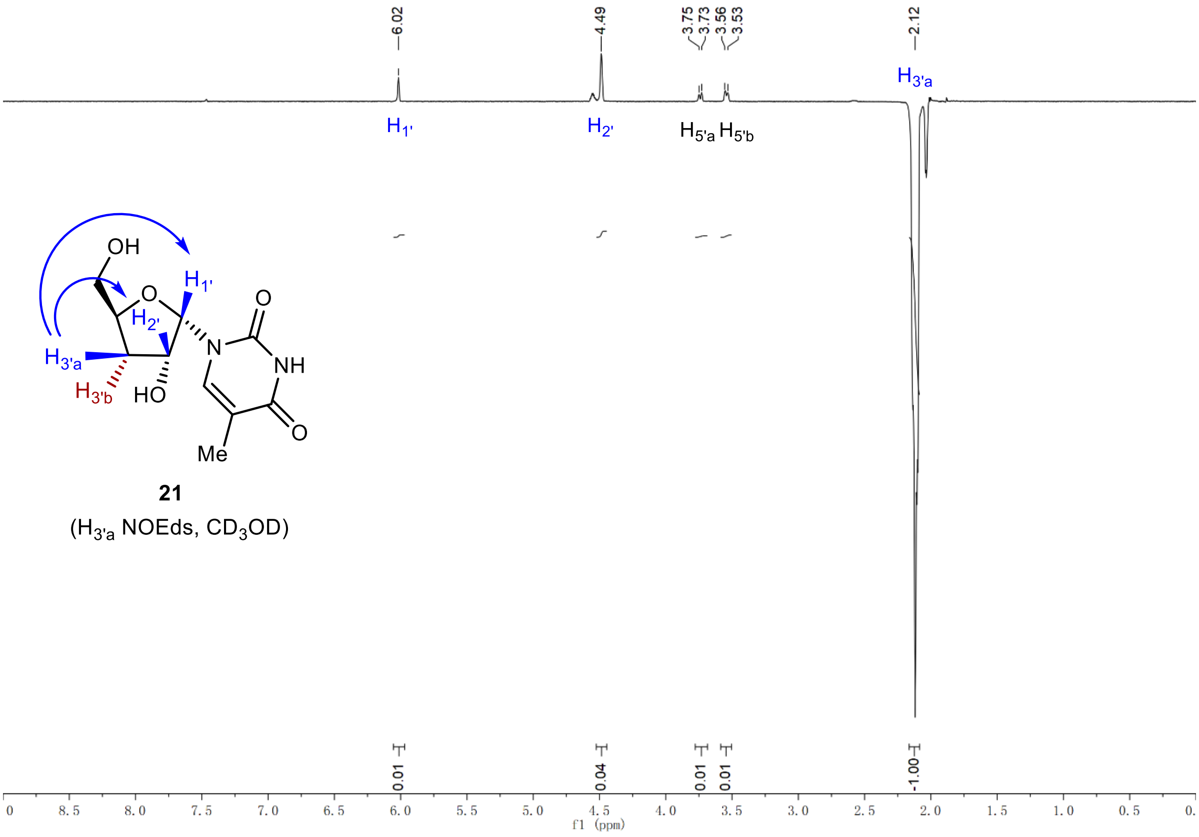

Supplementary Figure 274. $H_{3'a}$ NOEds (600M, CD_3OD) of compound **21.**

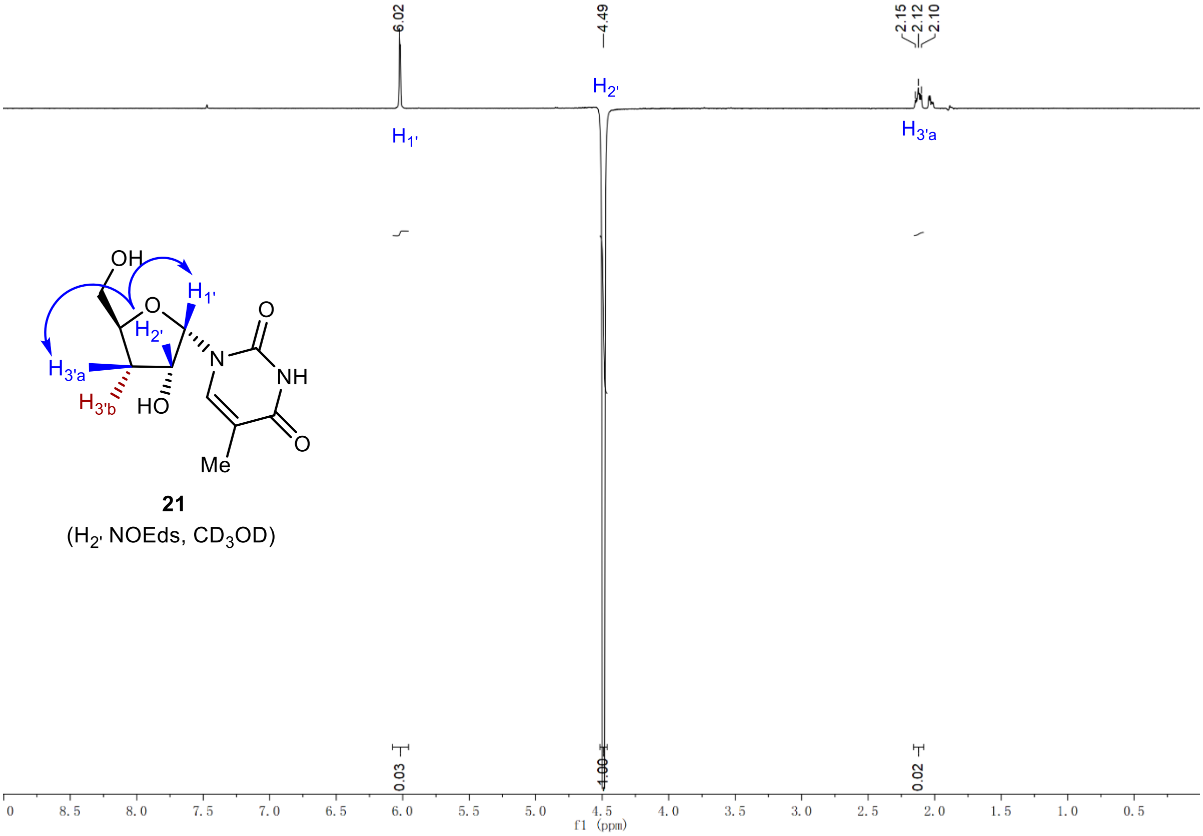

Supplementary Figure 275. $H_{2'}$ NOEds (600M, CD_3OD) of compound **21.**

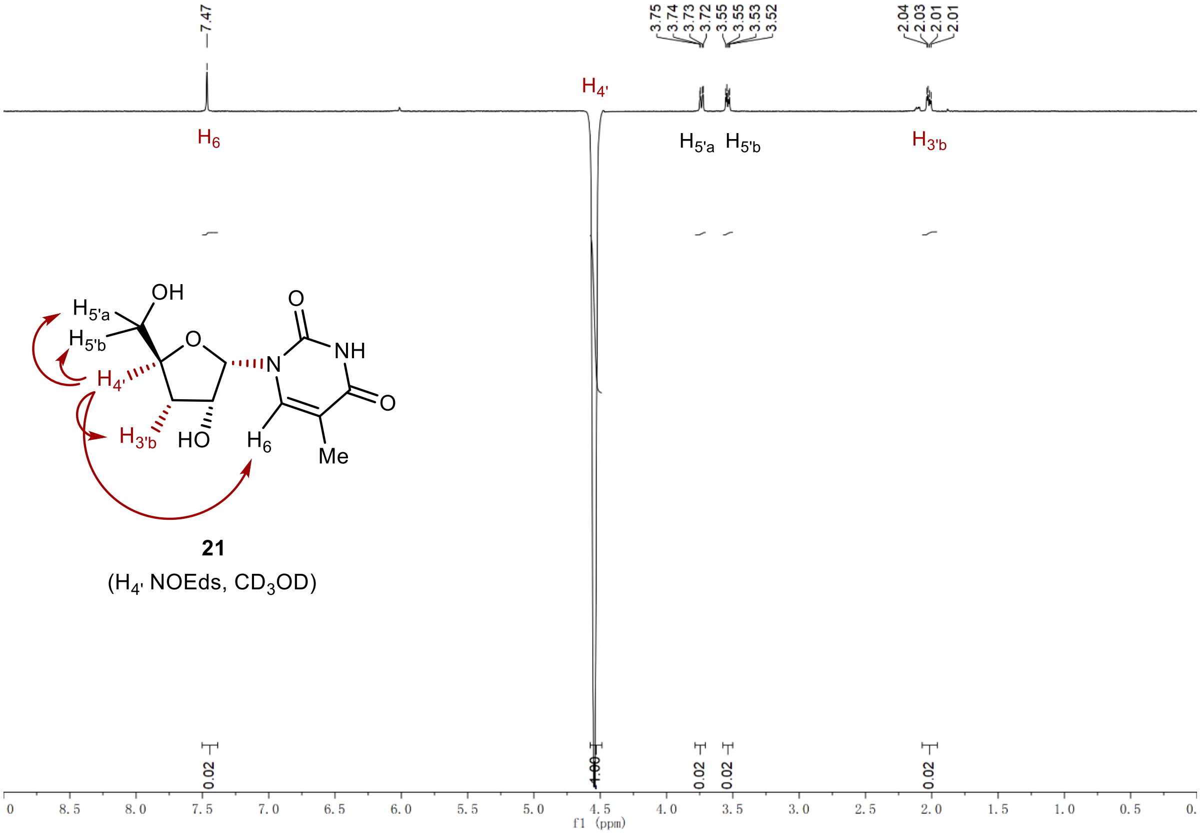

Supplementary Figure 276. $H_{4'}$ NOEds (600M, CD_3OD) of compound **21.**

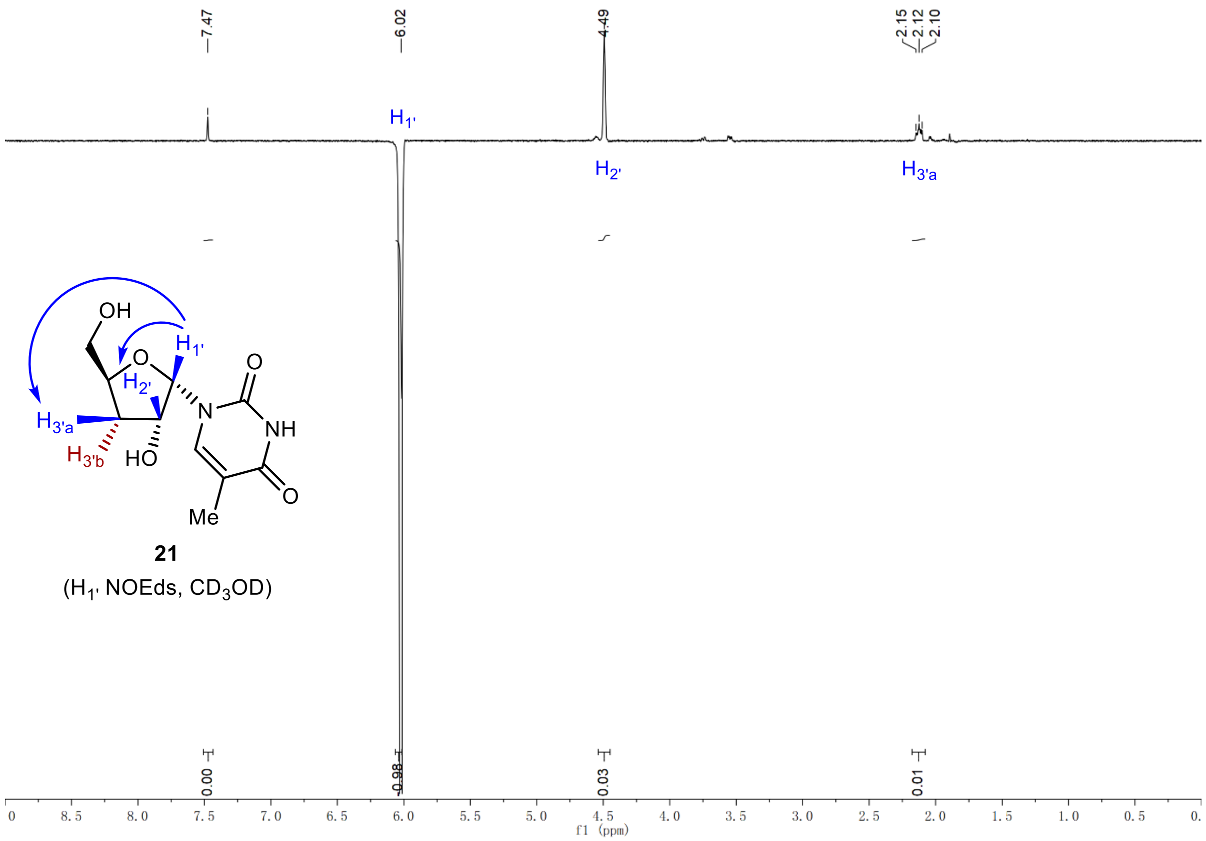

Supplementary Figure 277. $H_{1'}$ NOEds (600M, CD_3OD) of compound **21.**

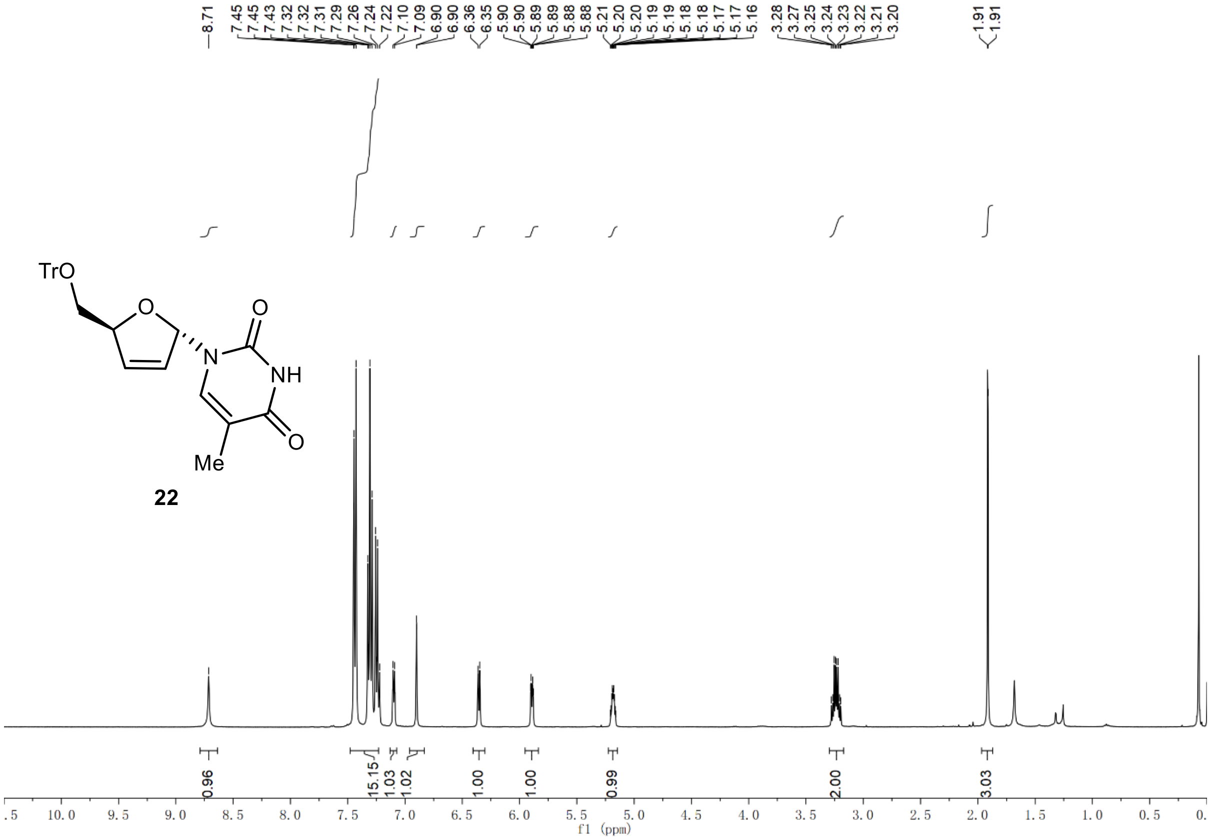

Supplementary Figure 278. ^{1}H NMR (400M, $CDCl_3$) of compound **22.**

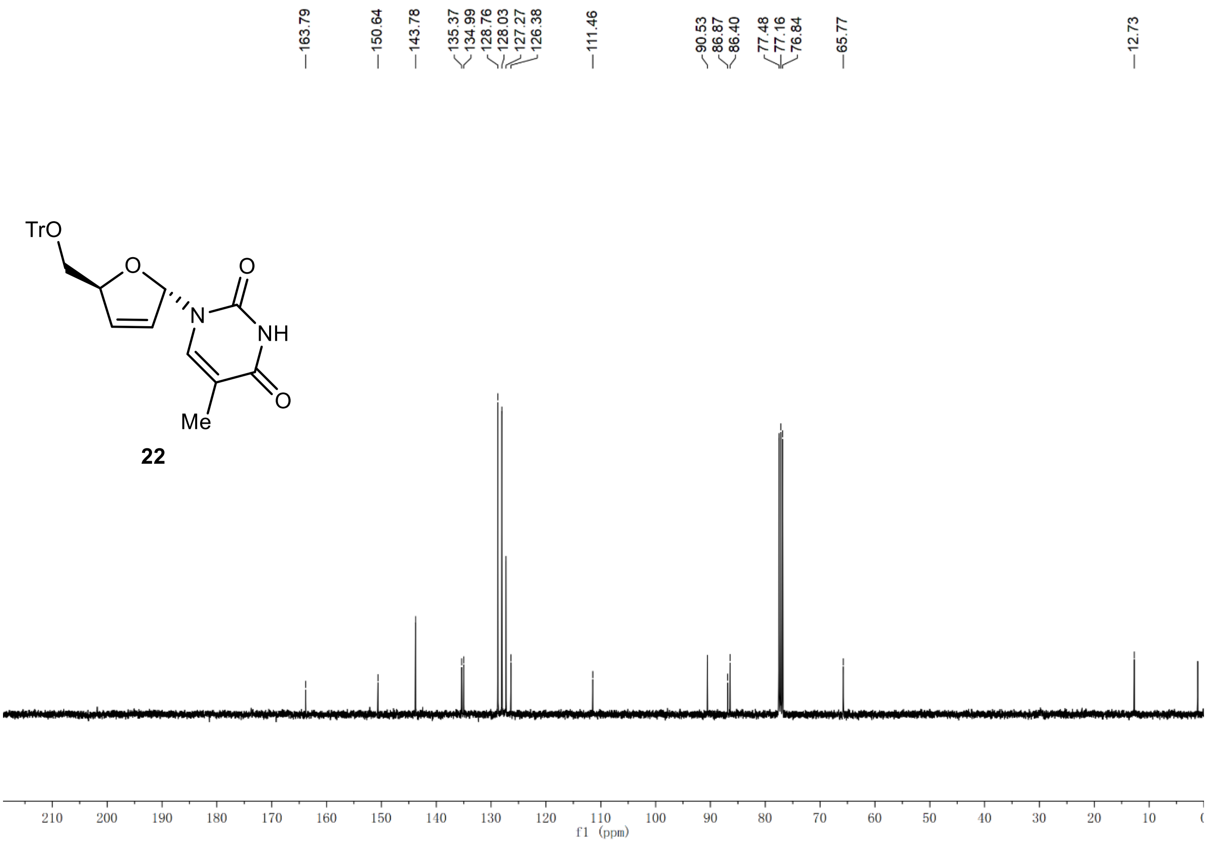

Supplementary Figure 279. ^{13}C NMR (100M, $CDCl_3$) of compound **22.**

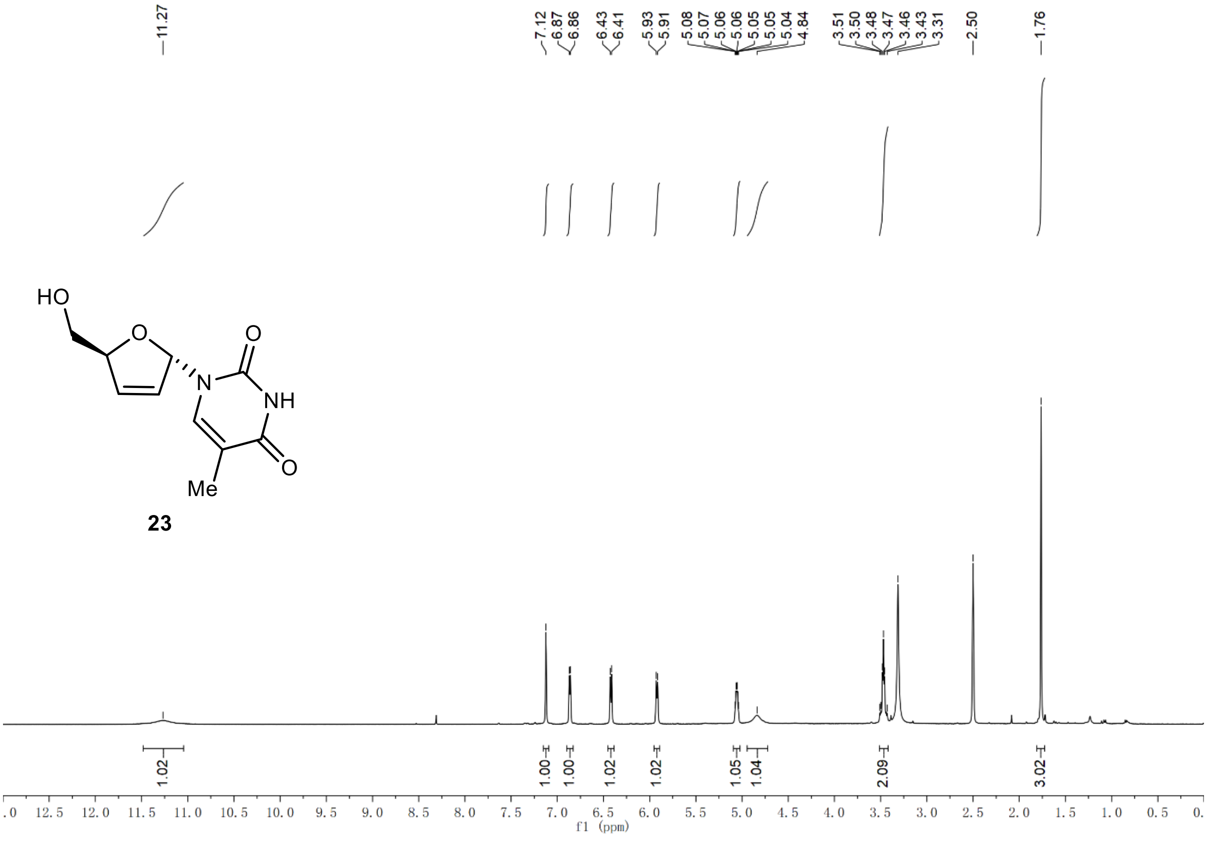

Supplementary Figure 280. ^{1}H NMR (400M, $(CD_3)_2SO$) of compound **23.**

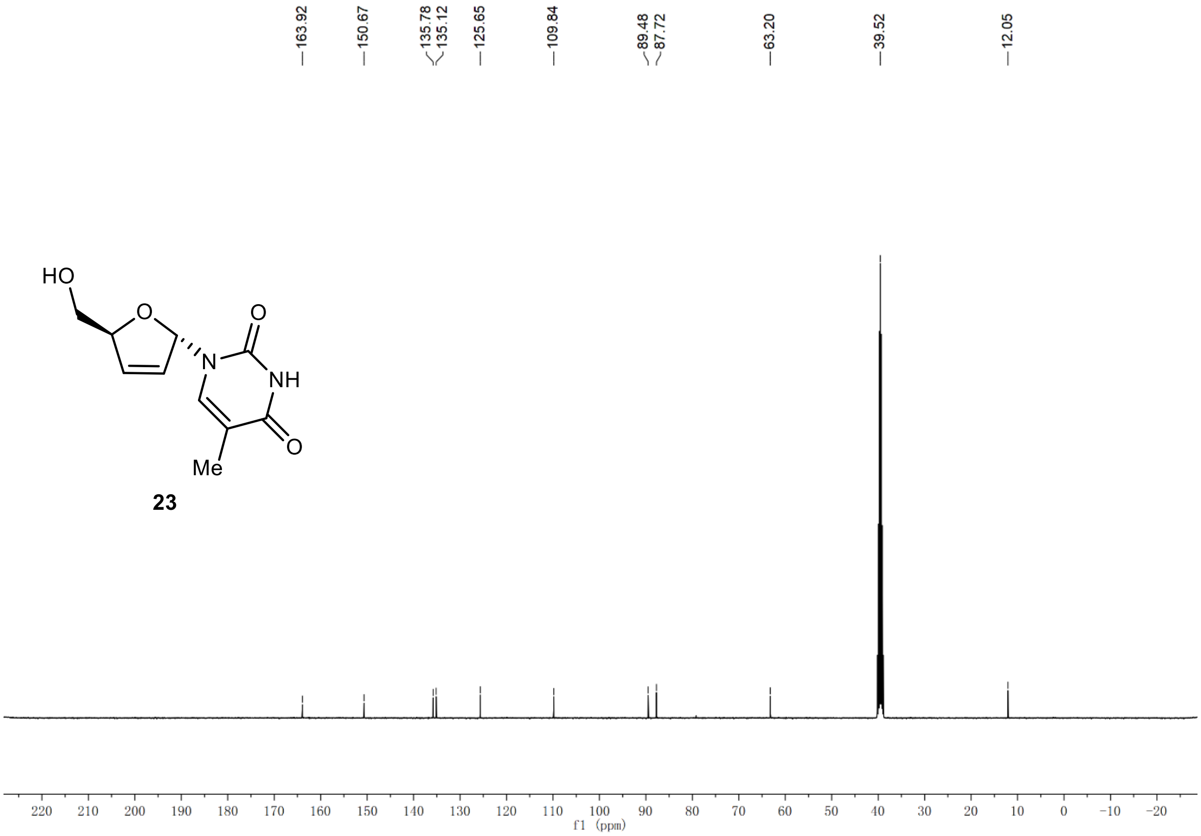

Supplementary Figure 281. ^{13}C NMR (100M, $(CD_3)_2SO$) of compound **23.**

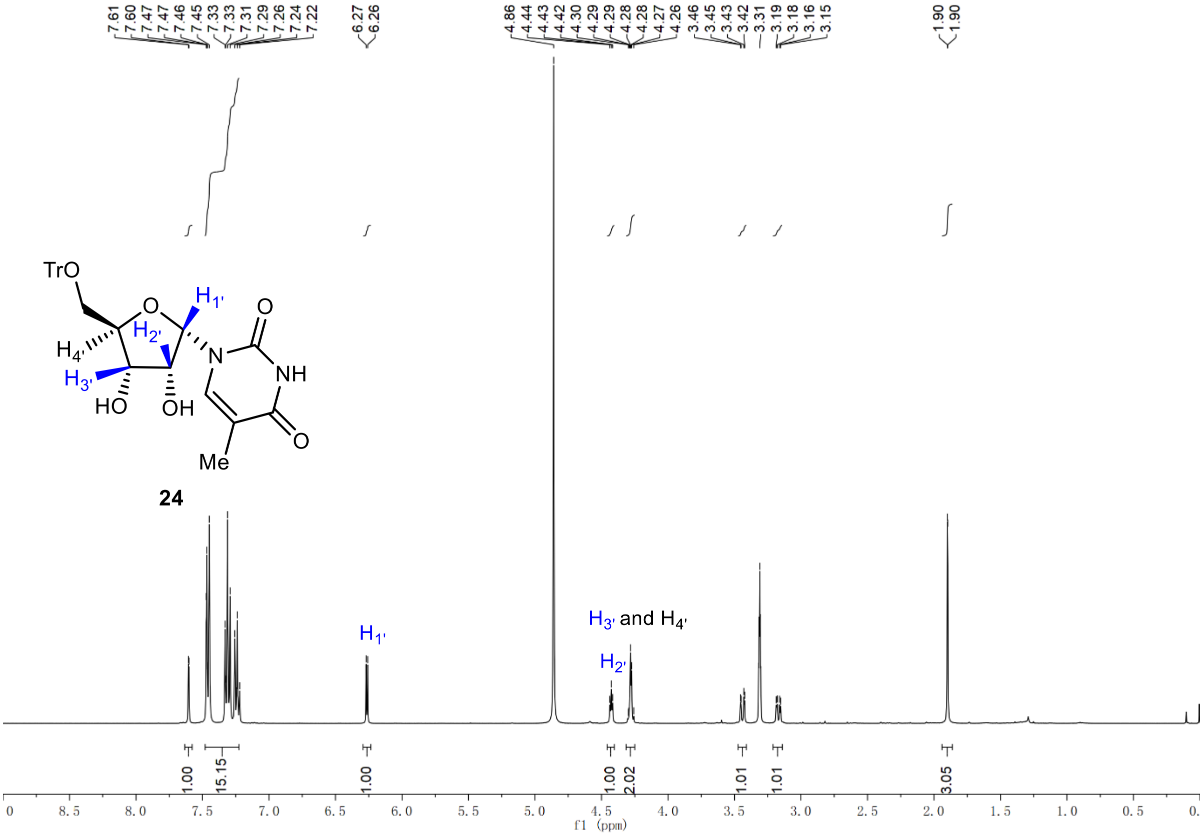

Supplementary Figure 282. ^{1}H NMR (400M, CD_3OD) of compound **24.**

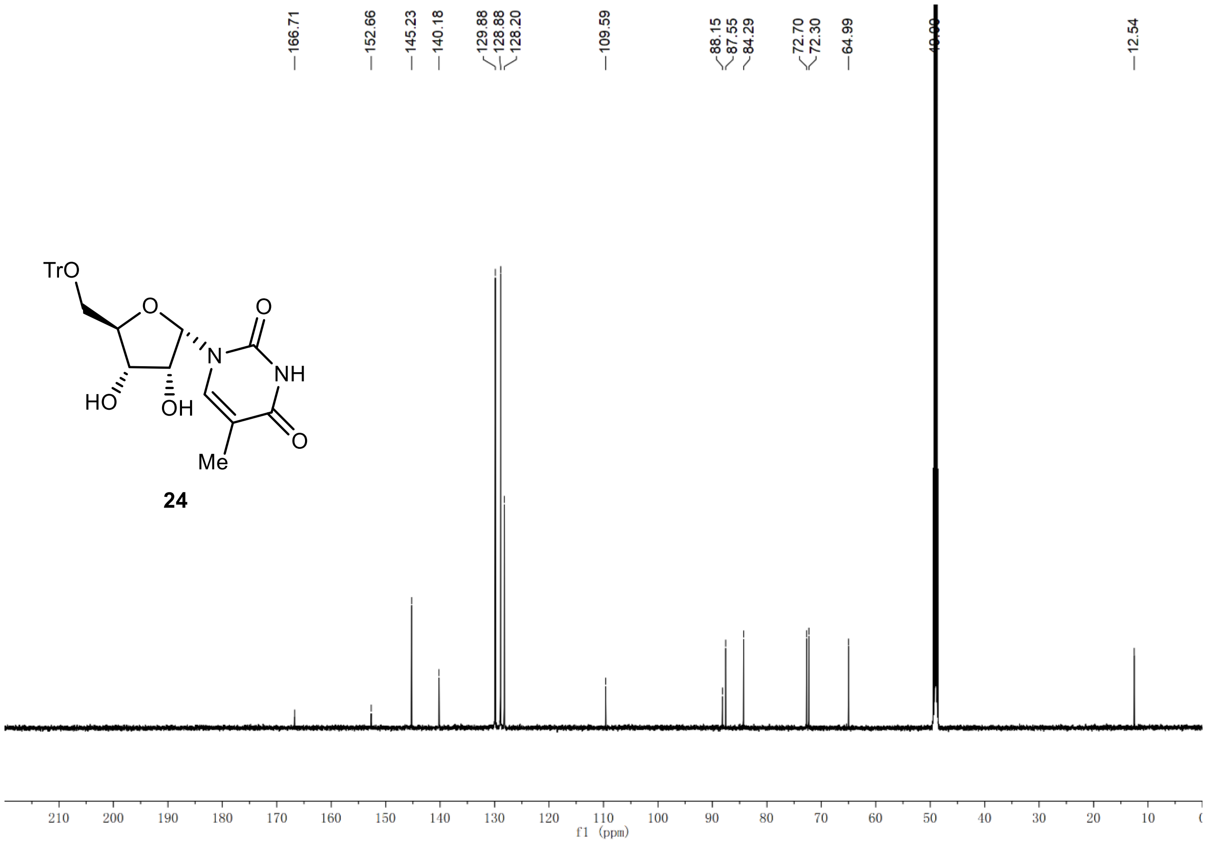

Supplementary Figure 283. ^{13}C NMR (100M, CD_3OD) of compound **24.**

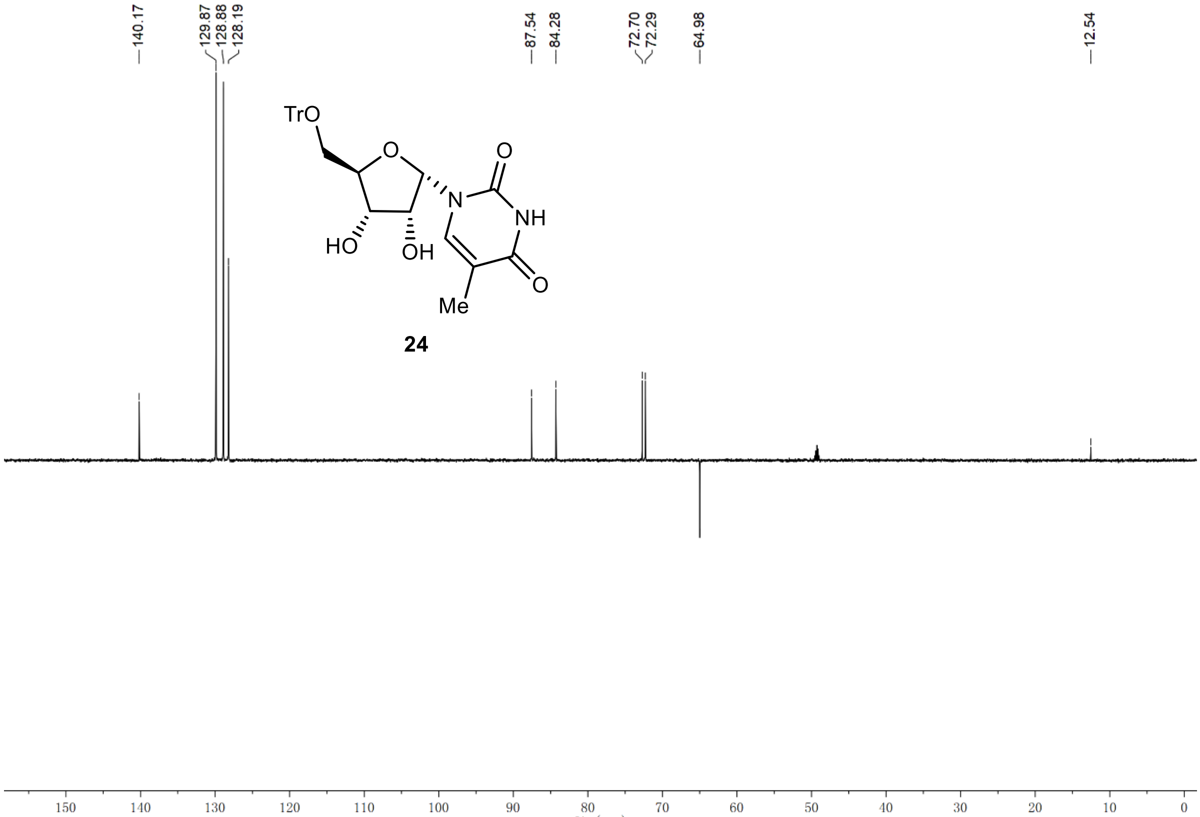

Supplementary Figure 284. DEPT 135° (100 M, CD_3OD) of compound **24.**

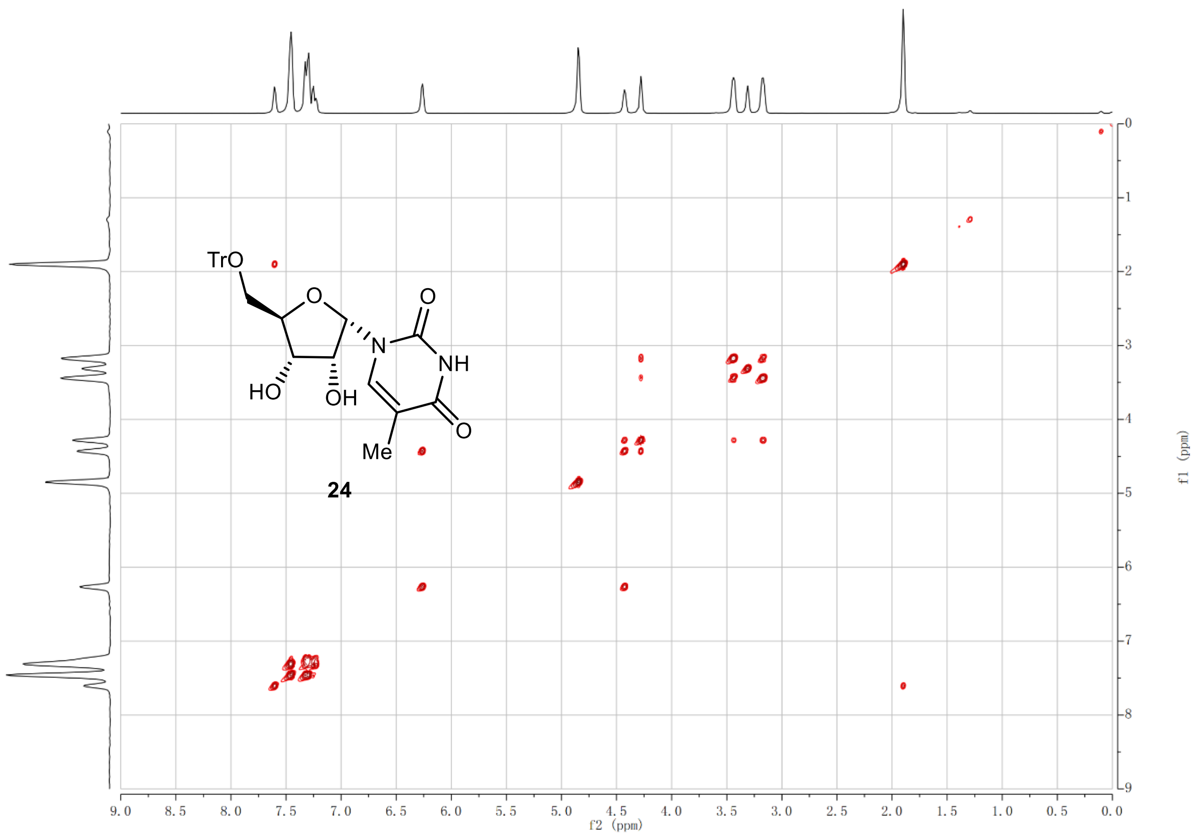

Supplementary Figure 285. ^{1}H-^{1}H COSY (400M, CD_3OD) of compound **24.**

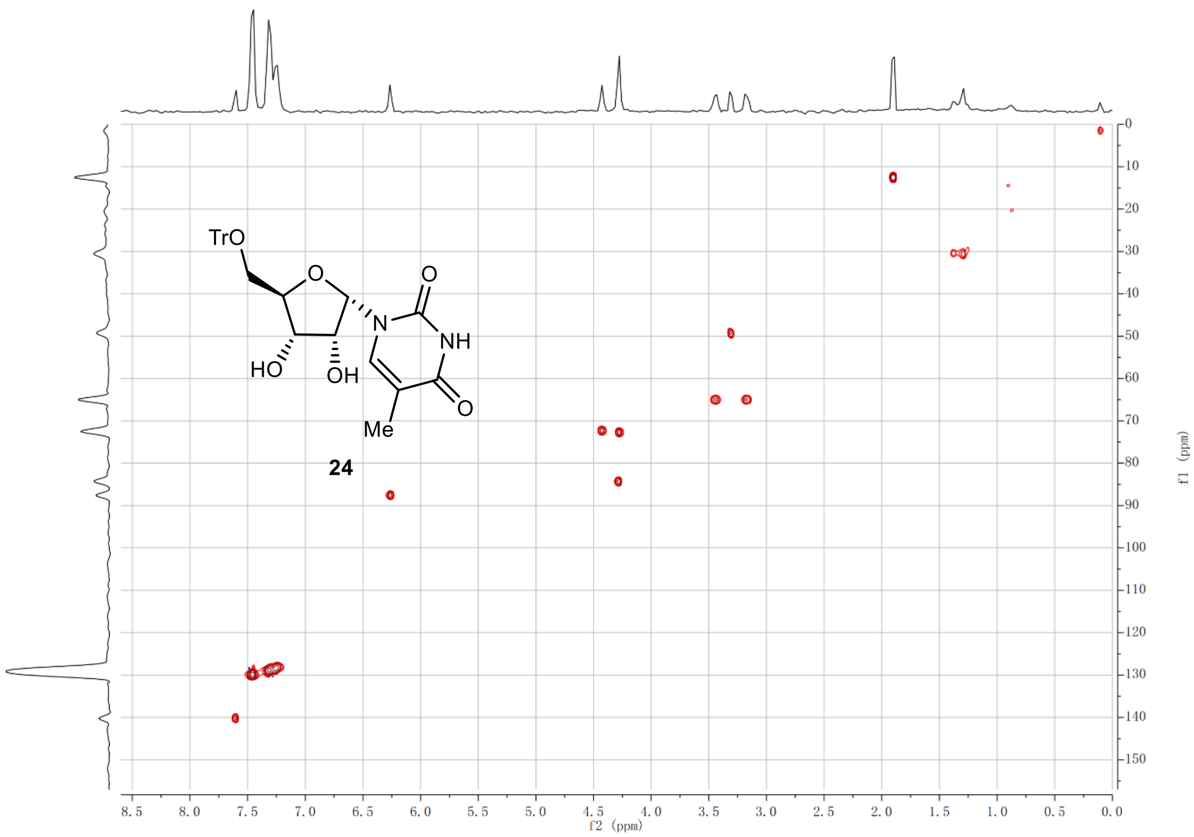

Supplementary Figure 286. ^{1}H-^{13}C HMQC (400M, CD_3OD) of compound **24.**

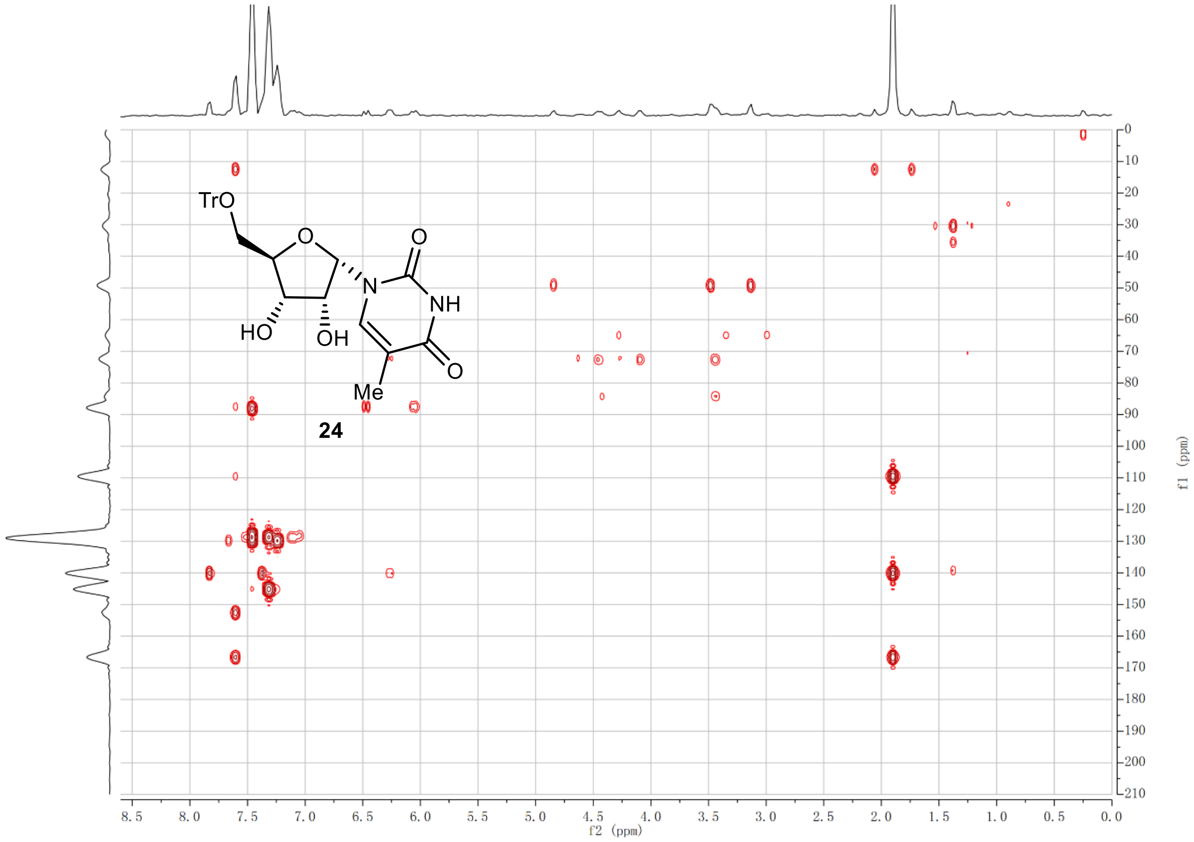

Supplementary Figure 287. ^{1}H-^{13}C HMBC (400M, CD_3OD) of compound **24.**

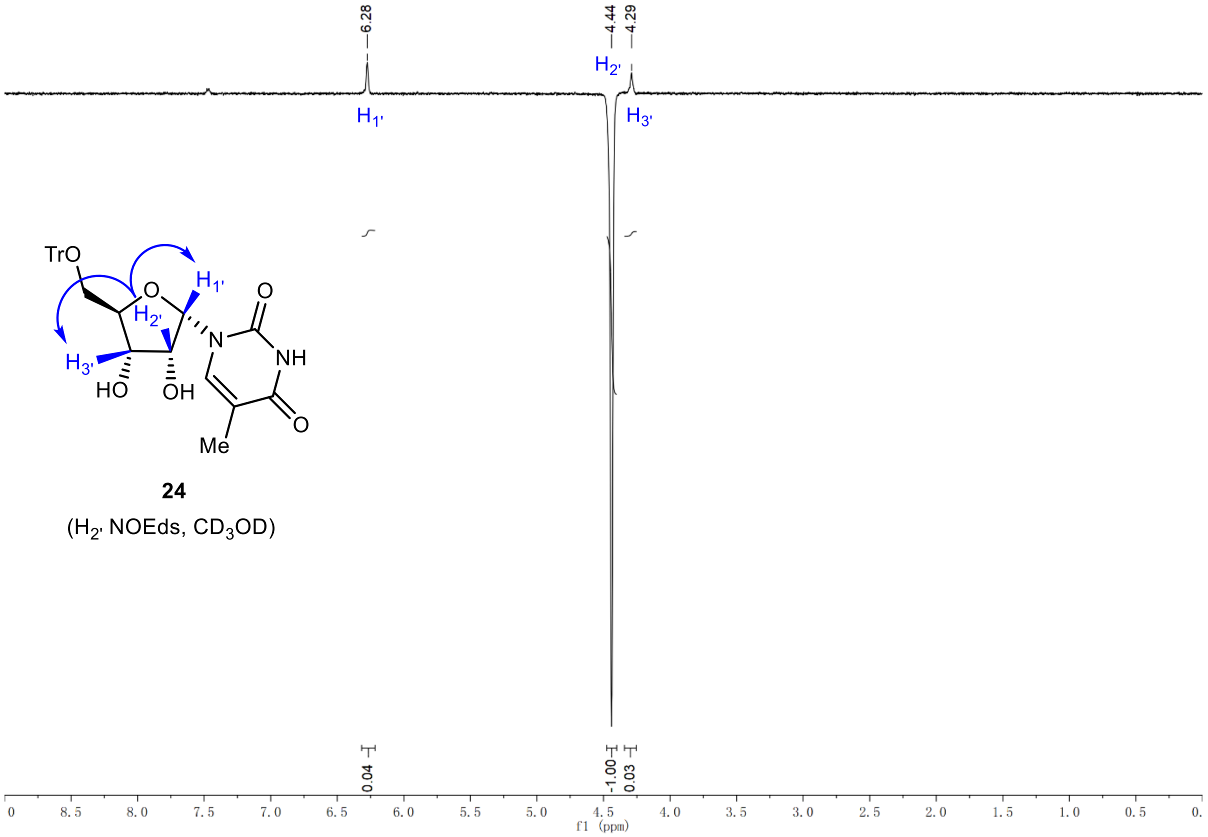

Supplementary Figure 288. $H_{2'}$ NOEds (600M, CD_3OD) of compound **24.**

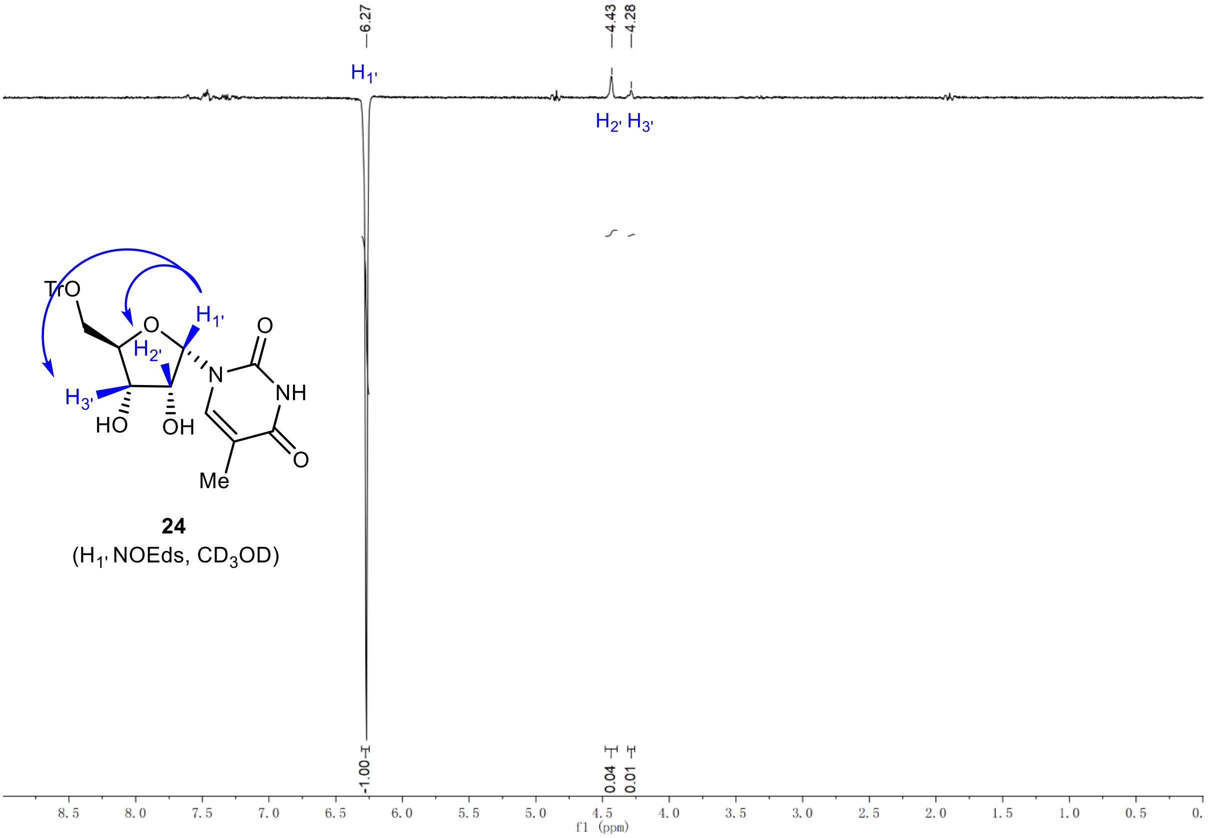

Supplementary Figure 289. H$_{1'}$ NOEds (600M, CD_3OD) of compound **24.**

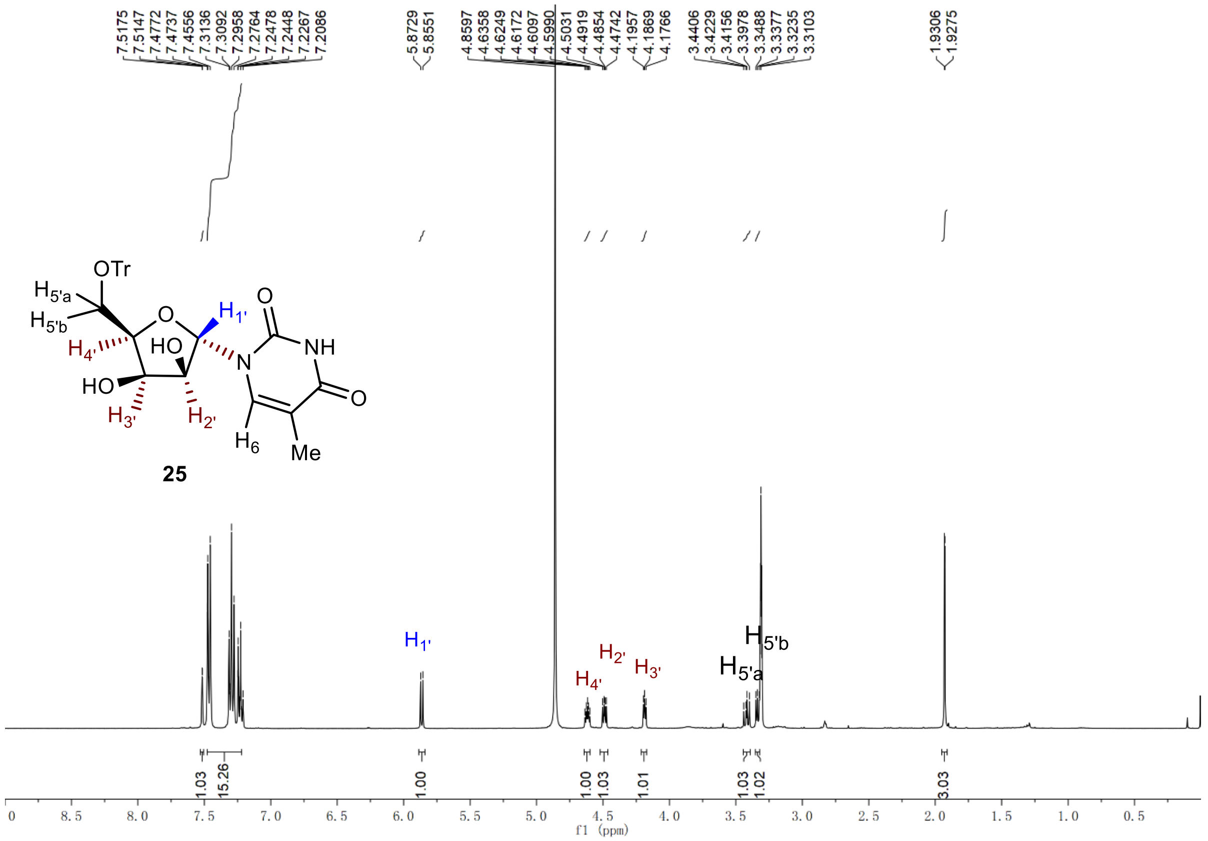

Supplementary Figure 290. ^{1}H NMR (400M, CD_3OD) of compound **25.**

H6

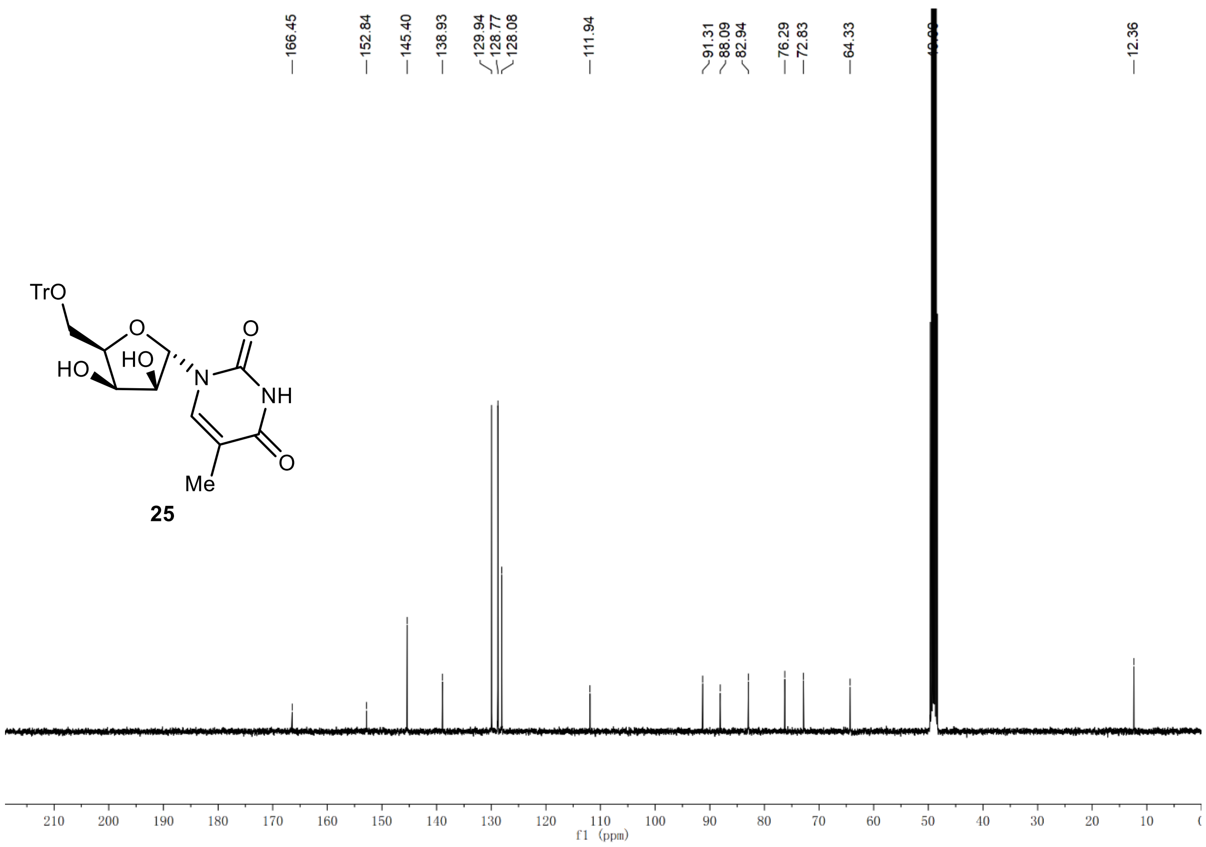

Supplementary Figure 291. ^{13}C NMR (100M, CD_3OD) of compound **25.**

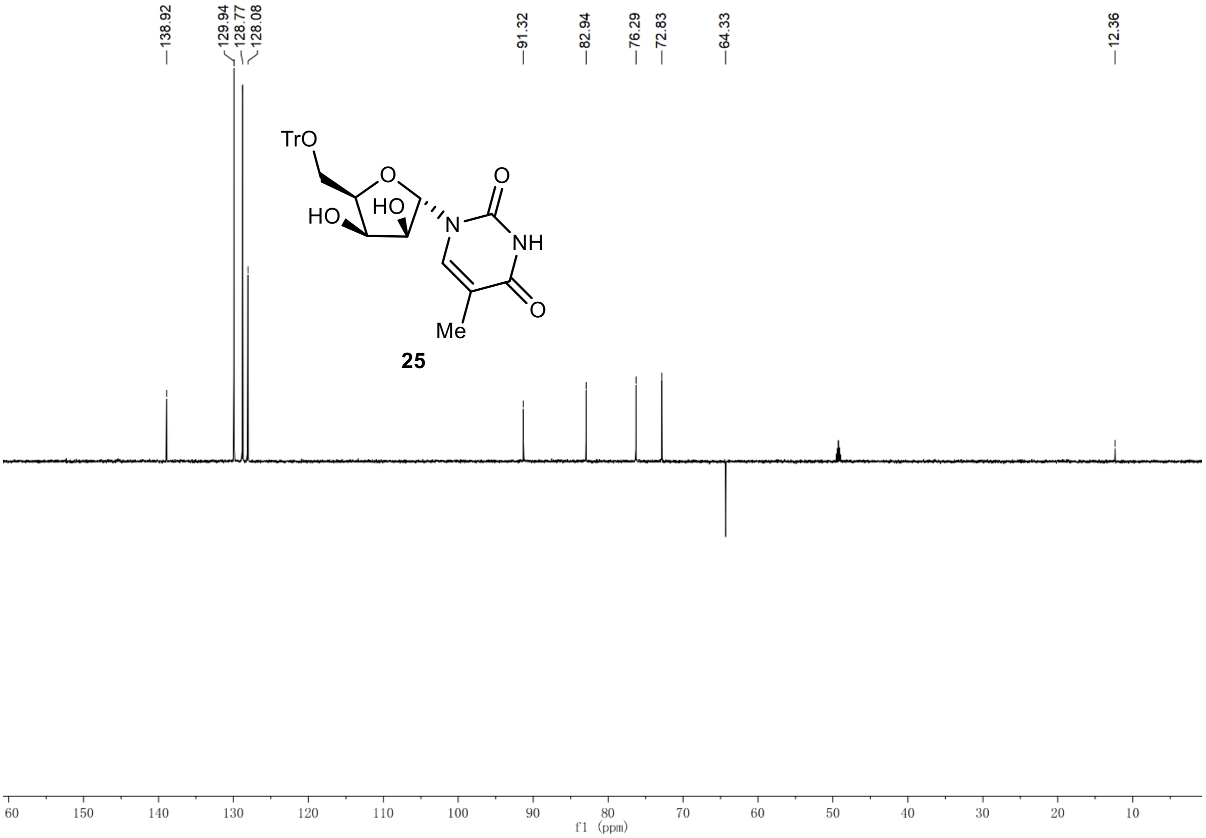

Supplementary Figure 292. DEPT 135° (100 M, CD_3OD) of compound **25.**

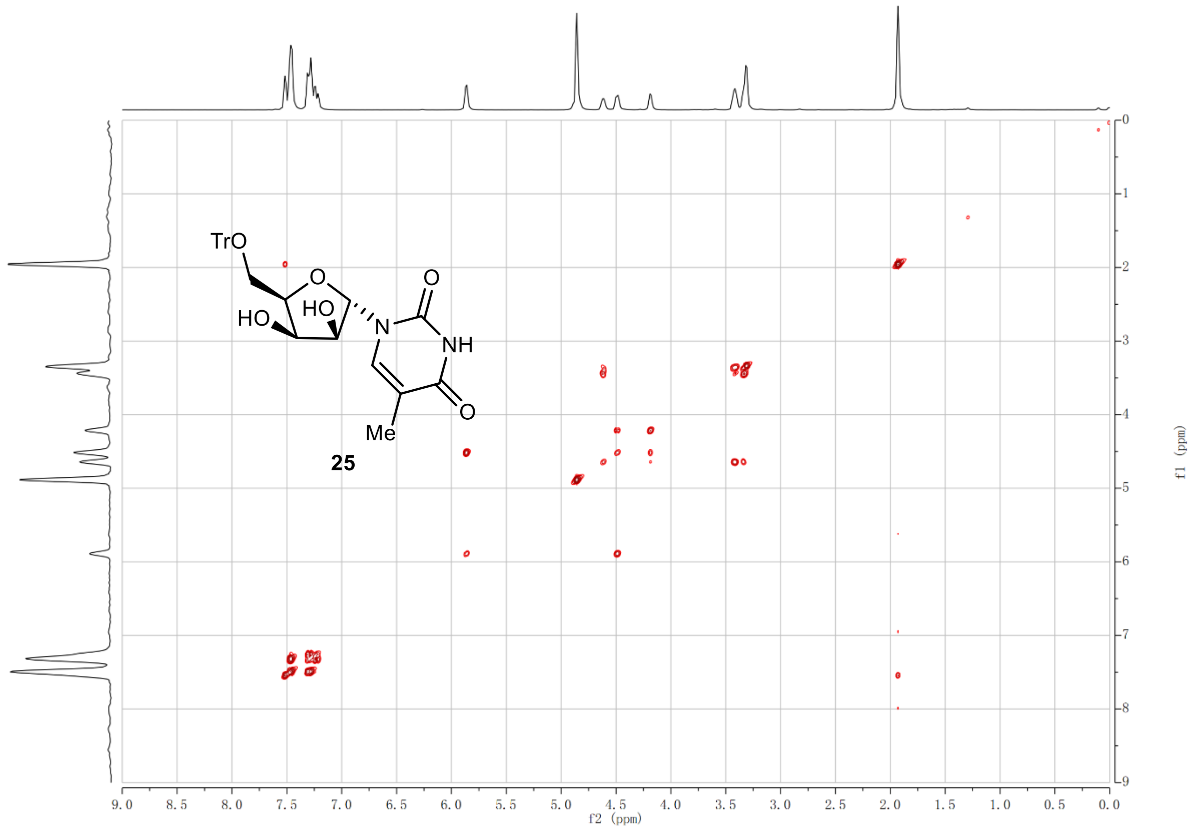

Supplementary Figure 293. ^{1}H-^{1}H COSY (400M, CD_3OD) of compound **25.**

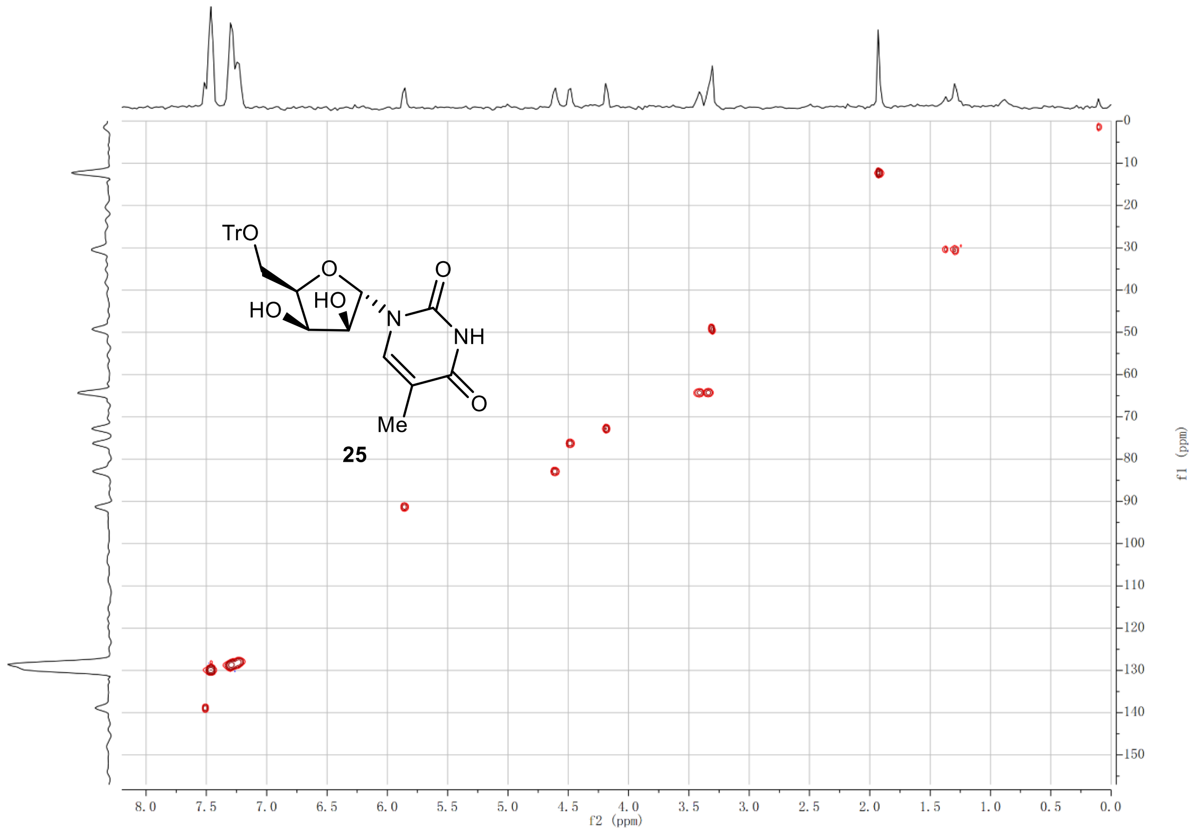

Supplementary Figure 294. ^{1}H-^{13}C HMQC (400M, CD_3OD) of compound **25.**

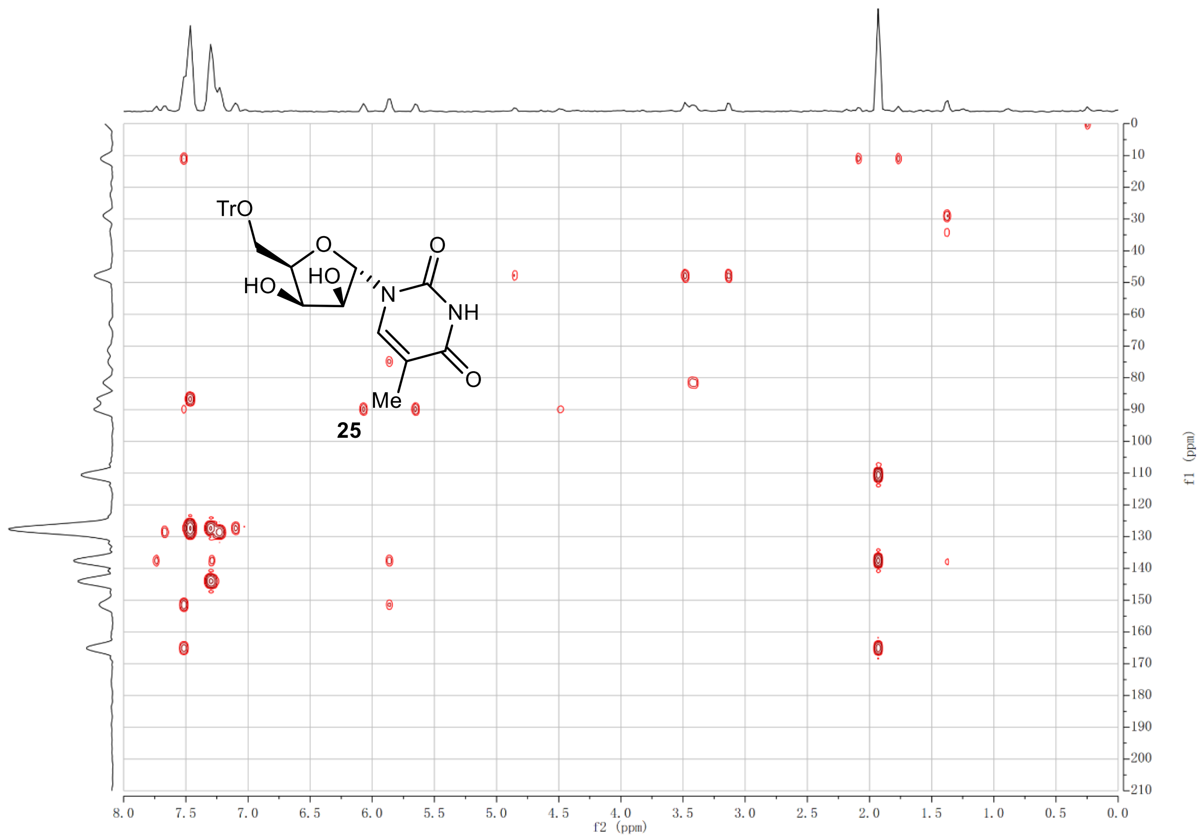

Supplementary Figure 295. ^{1}H-^{13}C HMBC (400M, CD_3OD) of compound **25.**

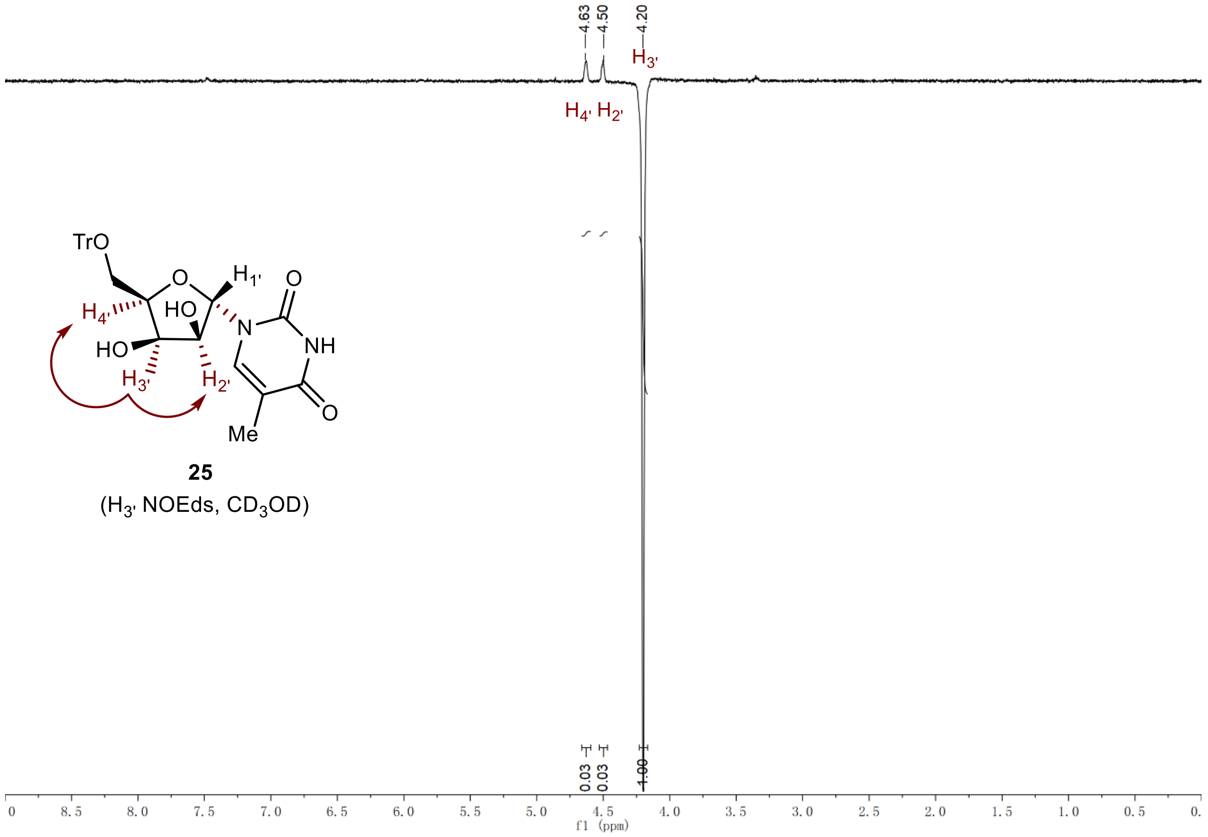

Supplementary Figure 296. $H_{3'}$ NOEds (600M, CD_3OD) of compound **25.**

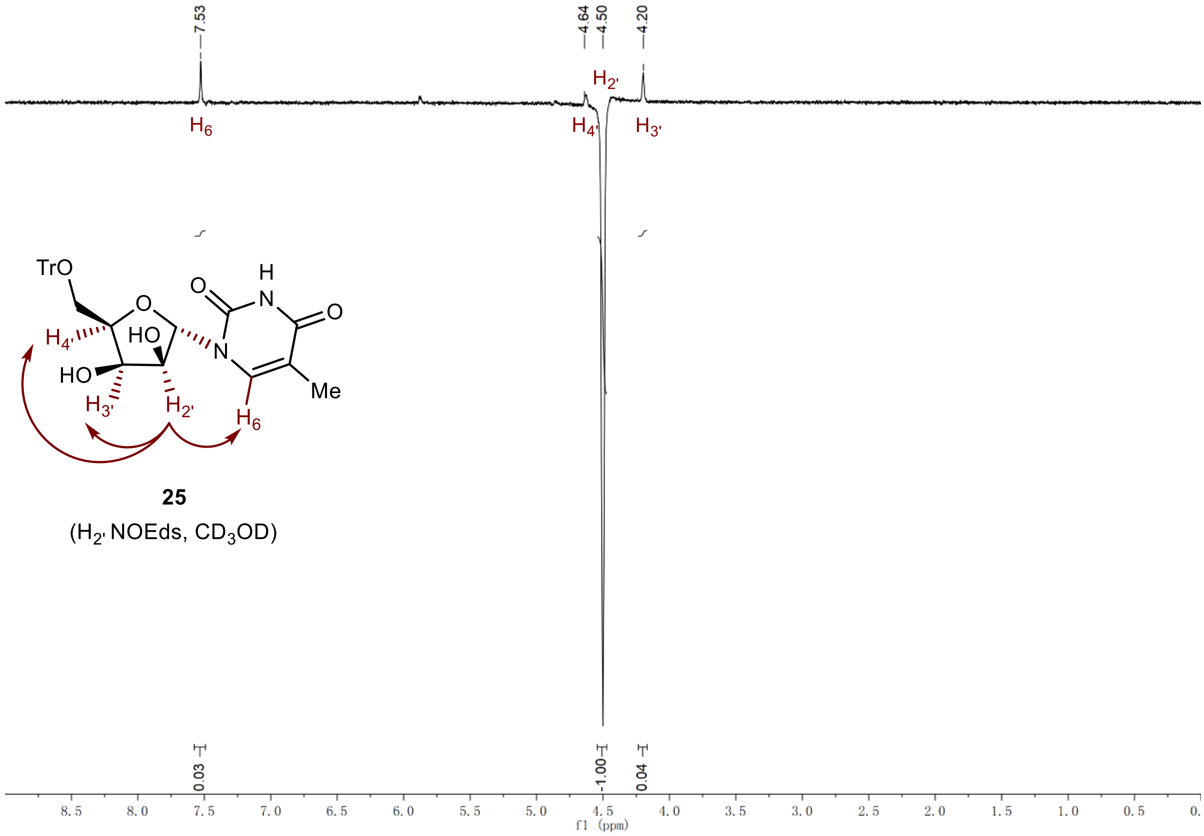

Supplementary Figure 297. $H_{2'}$ NOEds (600M, CD_3OD) of compound **25.**

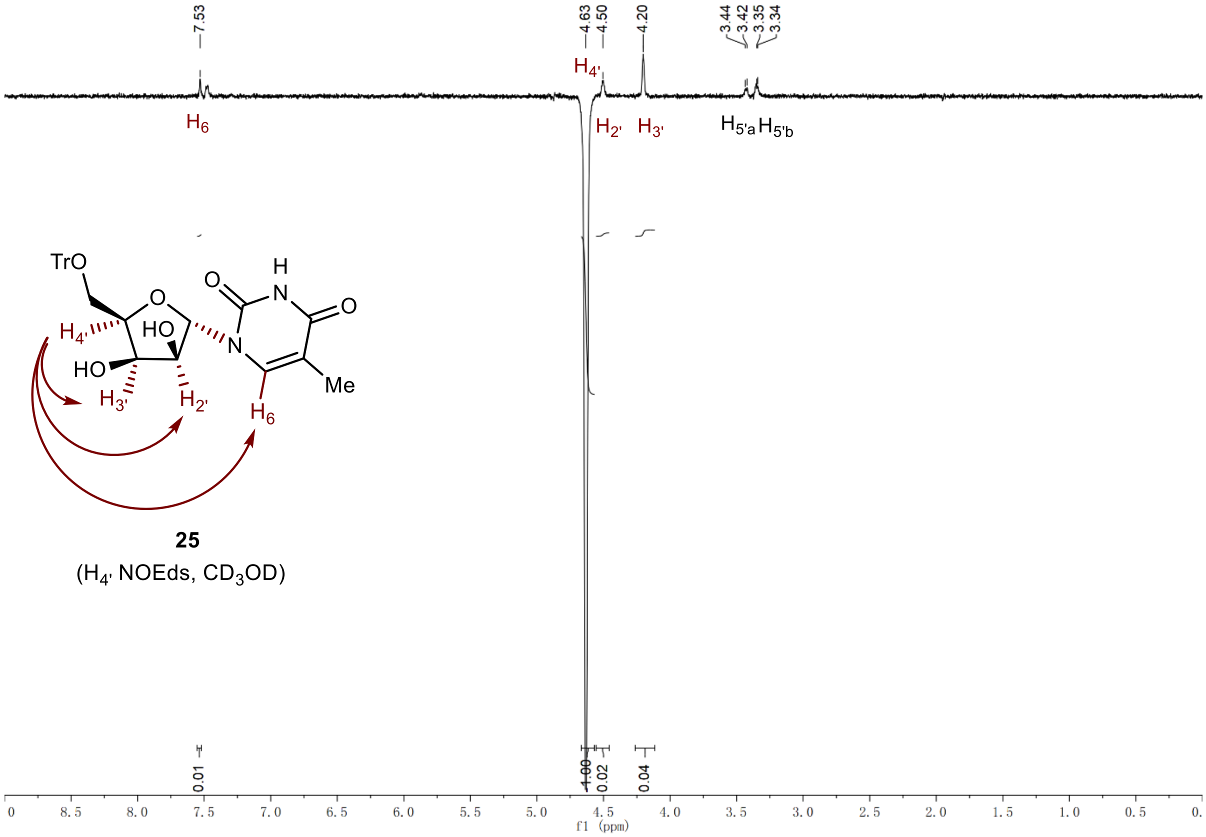

Supplementary Figure 298. $H_{4'}$ NOEds (600M, CD_3OD) of compound **25.**

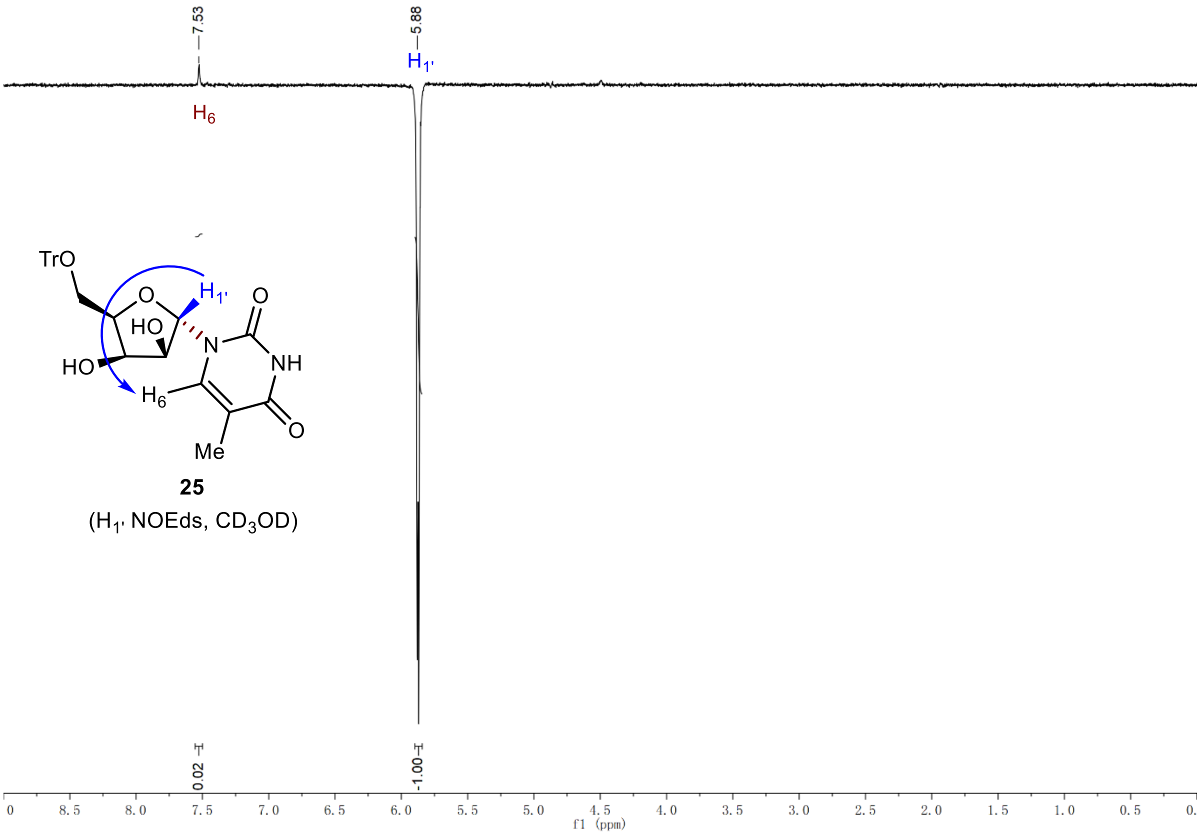

Supplementary Figure 299. $H_{1'}$ NOEds (600M, CD_3OD) of compound **25.**

4 Supplementary Tables 15-21

Supplementary Table 15. Comparison of ^{1}H NMR data of β-D-ddT (**9**) with literature.

H	Lit.[3] data (270 MHz, CD_3OD-$CDCl_3$) δ/ppm	Our data (400 MHz, CD_3OD) δ/ppm	Error Δ δ/ppm
H_6	7.76 (s, 1H)	7.92 (s, 1H)	–0.16
$H_{1'}$	6.05 (m, 1H)	6.05 (dd, *J* = 7.0, 3.6 Hz, 1H)	-
$H_{4'}$	4.10 (m, 1H)	4.15–4.10 (m, 1H)	-
$H_{5'a}$	3.77 (m, 2H)	3.88 (dd, *J* = 12.2, 3.0 Hz, 1H)	-
$H_{5'b}$		3.68 (dd, *J* = 12.2, 3.8 Hz, 1H)	-
$H_{2'}$, $H_{3'}$	2.07 (m, 4H)	2.44–1.94 (m, 4H)	-
H_7	1.90 (s, 3H)	1.88 (s, 3H)	0.02

Supplementary Table 16. Comparison of ^{13}C NMR data of β-D-ddT (**9**) with literature.

C	Lit.[1] data (50 MHz, $(CD_3)_2SO$) δ/ppm	Our data (100 MHz, $(CD_3)_2SO$) δ/ppm	Error Δ δ/ppm
C_4	163.7	163.8	–0.1
C_2	150.4	150.4	0
C_6	136.7	136.3	0.4
C_5	109.0	108.7	0.3
$C_{1'}$	84.8	84.7	0.1
$C_{4'}$	81.4	81.2	0.2
$C_{5'}$	62.8	62.1	0.7
$C_{2'}$	31.8	31.5	0.3
$C_{3'}$	25.0	24.9	0.1
C_7	12.1	12.2	–0.1

Supplementary Table 17. Comparison of ^{1}H NMR data of stavudine with literature.

H	Lit.[4] data (300 MHz, CD_3OD) δ/ppm	Our data (400 MHz, CD_3OD) δ/ppm	Error Δ δ/ppm
H_6	7.75 (d, *J* = 1.1 Hz, 1H)	7.75 (d, *J* = 1.2 Hz, 1H)	0
$H_{1'}$	6.96–6.94 (m, 1H)	6.96–6.94 (m, 1H)	-
$H_{3'}$	6.41 (dt, *J* = 5.8, 1.7 Hz, 1H)	6.40 (dt, *J* = 6.0, 1.8 Hz, 1H)	0.01
$H_{2'}$	5.90 (dq, *J* = 2.4, 1.2 Hz, 1H)	5.90 (dd, *J* = 6.0, 1.8 Hz, 1H)	0
$H_{4'}$	4.87–4.84 (m, 1H)	4.87–4.85 (m, 1H)	-
$H_{5'}$	3.83–3.74 (m, 2H)	3.81–3.72 (m, 2H)	-
H_7	1.84 (d, *J* = 1.1 Hz, 3H)	1.84 (d, *J* = 1.2 Hz, 3H)	0

Supplementary Table 18. Comparison of ^{1}H NMR data of 5-methyluridine (**17**) with literature.

H	Lit.[5] data (400 MHz, D_2O) δ/ppm	Our data (400 MHz, D_2O) δ/ppm	Error Δ δ/ppm
H_6	7.61 (d, *J* = 1.0 Hz 1H)	7.61 (d, *J* = 1.6 Hz 1H)	0
$H_{1'}$	5.81 (d, *J* = 4.5 Hz 1H)	5.83 (d, *J* = 4.6 Hz 1H)	–0.02
$H_{2'}$	4.25 (t, *J* = 5.1 Hz 1H)	4.26 (t, *J* = 5.2 Hz 1H)	–0.01
$H_{3'}$	4.15 (t, *J* = 5.2 Hz 1H)	4.16 (t, *J* = 5.4 Hz 1H)	–0.01
$H_{4'}$	4.09–4.00 (m, 4H)	4.07–4.00 (m, 4H)	-
$H_{5'a}$	3.84 (dd, *J* = 12.5, 3.0 Hz 1H)	3.83 (dd, *J* = 12.8, 3.0 Hz 1H)	0.01
$H_{5'b}$	3.73 (dd, *J* = 12.9, 4.2 Hz 1H)	3.73 (dd, *J* = 12.8, 4.2 Hz 1H)	0
H_7	1.80 (s, 3H)	1.81 (s, 3H)	–0.01

Supplementary Table 19. Comparison of ^{13}C NMR data of 5-methyluridine (**17**) with literature.

C	Lit.[5] data (100 MHz, D_2O) δ/ppm	Our data (100 MHz, D_2O) δ/ppm	Error Δ δ/ppm
C_4	166.6	166.6	0
C_2	151.9	151.9	0
C_6	137.5	137.5	0
C_5	111.6	111.6	0
$C_{1'}$	89.2	89.1	0.1
$C_{4'}$	84.3	84.2	0.1
$C_{2'}$	73.7	73.6	0.1
$C_{3'}$	69.5	69.5	0
$C_{5'}$	60.8	60.8	0
C_7	11.7	11.6	0.1

Supplementary Table 20. Comparison of ^{1}H NMR data of molnupiravir with literature.

H	Lit.[7] data (400 MHz, CD_3OD) δ/ppm	Our data (400 MHz, CD_3OD) δ/ppm	Error Δ δ/ppm
H_6	6.89 (d, J = 8.2 Hz 1H)	6.90 (d, J = 8.2 Hz 1H)	–0.01
$H_{1'}$	5.80 (d, J = 4.8 Hz 1H)	5.81 (d, J = 4.8 Hz 1H)	–0.01
H_5	5.61 (d, J = 8.2 Hz 1H)	5.61 (d, J = 8.2 Hz 1H)	0
$H_{5'}$	4.28 (d, J = 3.6 Hz 2H)	4.29 (d, J = 3.6 Hz 2H)	–0.01
$H_{2'}$	4.13 (t, J = 4.8 Hz 1H)	4.13 (t, J = 4.8 Hz 1H)	0
$H_{3',4'}$	4.10–4.04 (m, 2H)	4.10–4.06 (m, 2H)	-
$H_{7'}$	2.60 (hept, J = 7.0 Hz 1H)	2.62 (hept, J = 7.0 Hz 1H)	–0.02
$H_{8'}$	1.18 (s, 3H)	1.19 (s, 3H)	–0.01
$H_{9'}$	1.16 (s, 3H)	1.17 (s, 3H)	–0.01

Supplementary Table 21. Comparison of ^{13}C NMR data of molnupiravir with literature.

C	Lit.[7] data (100 MHz, CD_3OD) δ/ppm	Our data (100 MHz, CD_3OD) δ/ppm	Error Δ δ/ppm
$C_{6'}$	178.3	178.3	0
C_2	151.6	151.5	0.1
C_4	146.2	146.2	0
C_6	131.8	131.8	0
C_5	99.6	99.6	0
$C_{1'}$	90.6	90.5	0.1
$C_{4'}$	82.6	82.6	0
$C_{2'}$	74.4	74.4	0
$C_{3'}$	71.6	71.5	0.1
$C_{5'}$	65.0	64.9	0.1
$C_{7'}$	35.2	35.2	0
$C_{8'}$	19.4	19.4	0
$C_{9'}$	19.3	19.3	0

5 Supplementary Figs 300-322

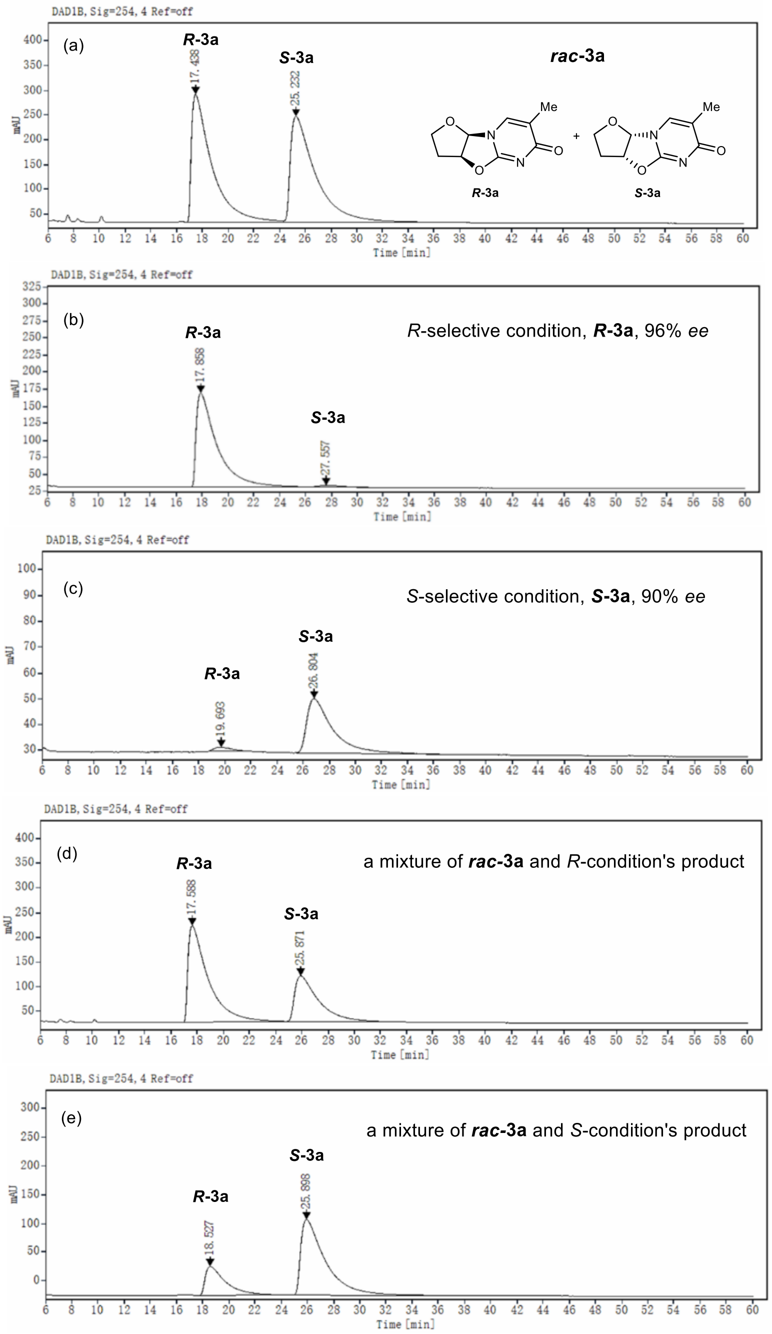

Supplementary Figure 300. HPLC Spectrum of ***R*-3a** (*R*-selective) and ***S*-3a** (*S*-selective).

Condition	Compound	RetTime [min]	Type	Width [min]	Area [mAU * s]	Height [mAU]	Area%
R-selective	***R*-3a**	17.858	BM m	1.48	15337.54	138.23	98.14
	***S*-3a**	27.557	MM m	1.47	289.98	2.31	1.86
S-selective	***R*-3a**	19.693	MM m	1.12	148.22	1.56	4.81
	***S*-3a**	26.804	MM m	1.64	2930.72	21.06	95.19

Illustrate: Due to the difference of retention time of ***R*-3a-i** and ***S*-3a-i** in the Supplementary Figs 300-308 (a), (b), (c), we added two HPLC spectrums (d) and (e).

Chiral HPLC conditions: CHIRALCEL OD-H (4.6 x 250 mm), 70:30 *n*-hexane (0.3% CH_3COOH)/*i*-PrOH, 1.0 mL/min, UV detector at 254 nm**,** injection volume: 15 μL, T (column) = 20 °C.

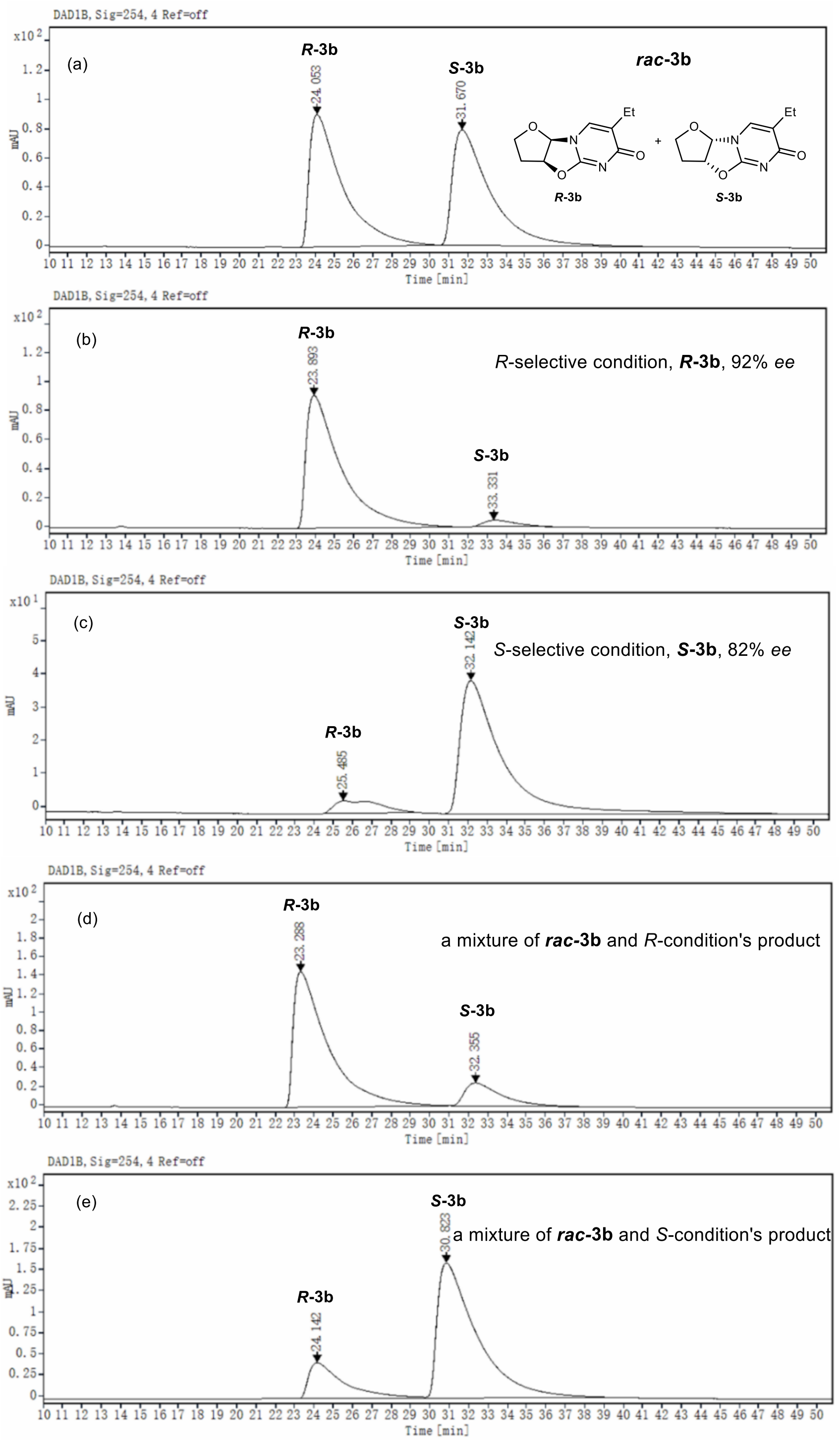

Supplementary Figure 301. HPLC Spectrum of ***R*-3b** (*R*-selective) and ***S*-3b** (*S*-selective).

Condition	Compound	RetTime [min]	Type	Width [min]	Area [mAU * s]	Height [mAU]	Area%
R-selective	***R*-3b**	23.893	BM m	1.69	11803.11	91.71	96.00
	***S*-3b**	33.331	MM m	1.37	491.76	4.24	4.00
S-selective	***R*-3b**	25.485	BM m	1.98	630.21	3.73	9.20
	***S*-3b**	32.142	BM m	1.83	6220.17	40.36	90.80

Chiral HPLC conditions: CHIRALCEL OD-H (4.6 x 250 mm), 75:25 *n*-hexane (0.3% CH_3COOH)/*i*-PrOH, 0.85 mL/min, UV detector at 254 nm**,** injection volume: 15 μL, T (column) = 20 ℃.

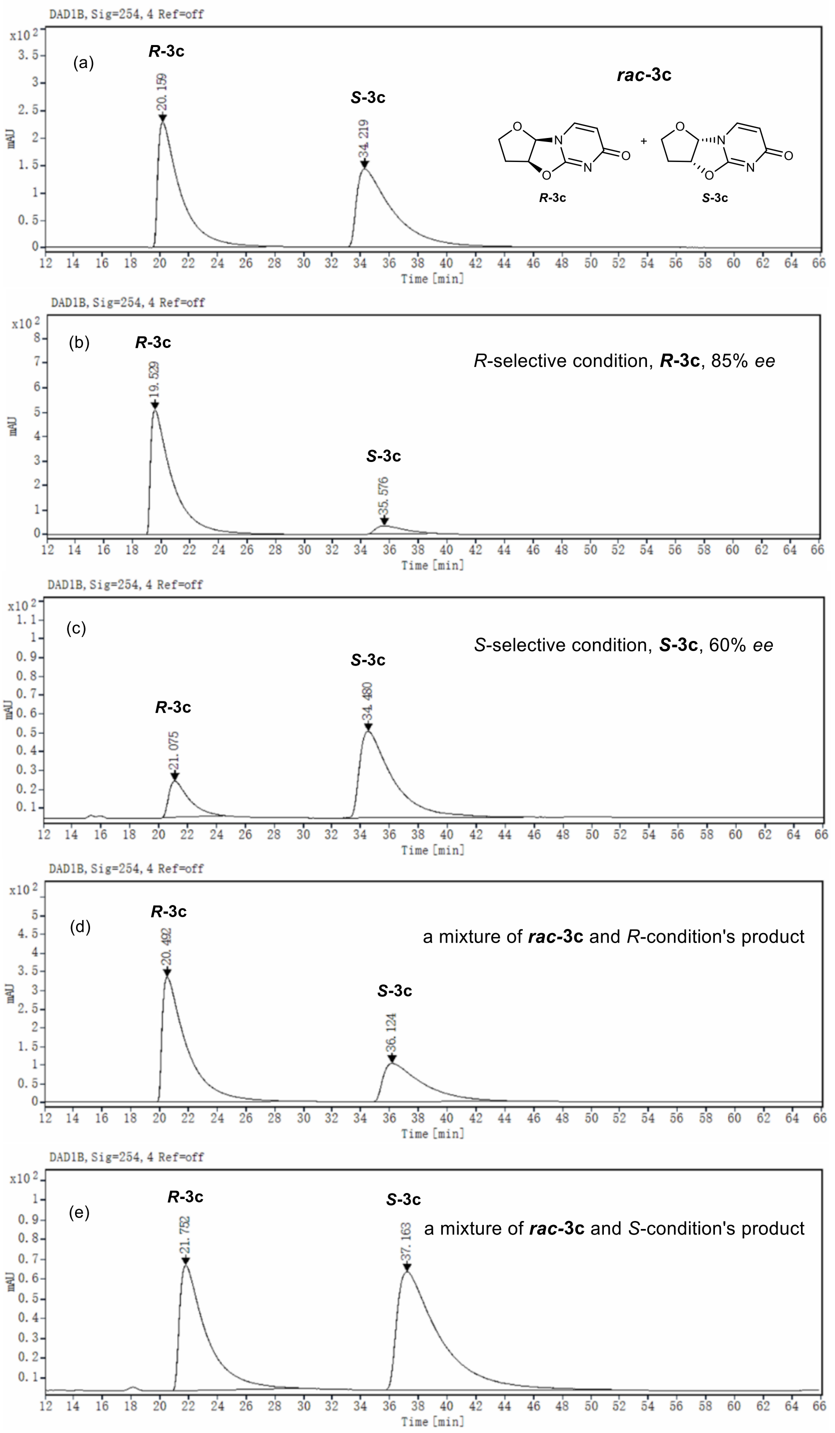

Supplementary Figure 302. HPLC Spectrum of ***R*-3c** (*R*-selective) and ***S*-3c** (*S*-selective).

Condition	Compound	RetTime [min]	Type	Width [min]	Area [mAU * s]	Height [mAU]	Area%
R-selective	***R*-3c**	19.529	BM m	1.44	53790.07	509.35	92.44
	***S*-3c**	35.576	MM m	1.59	4400.17	32.76	7.56
S-selective	***R*-3c**	21.075	MM m	1.19	1869.07	19.15	20.27
	***S*-3c**	34.480	MM m	1.88	7353.12	46.04	79.73

Chiral HPLC conditions: CHIRALCEL OD-H (4.6 x 250 mm), 70:30 *n*-hexane (0.3% CH_3COOH)/*i*-PrOH, 0.95 mL/min, UV detector at 254 nm**,** injection volume: 15 μL, T (column) = 20 °C.

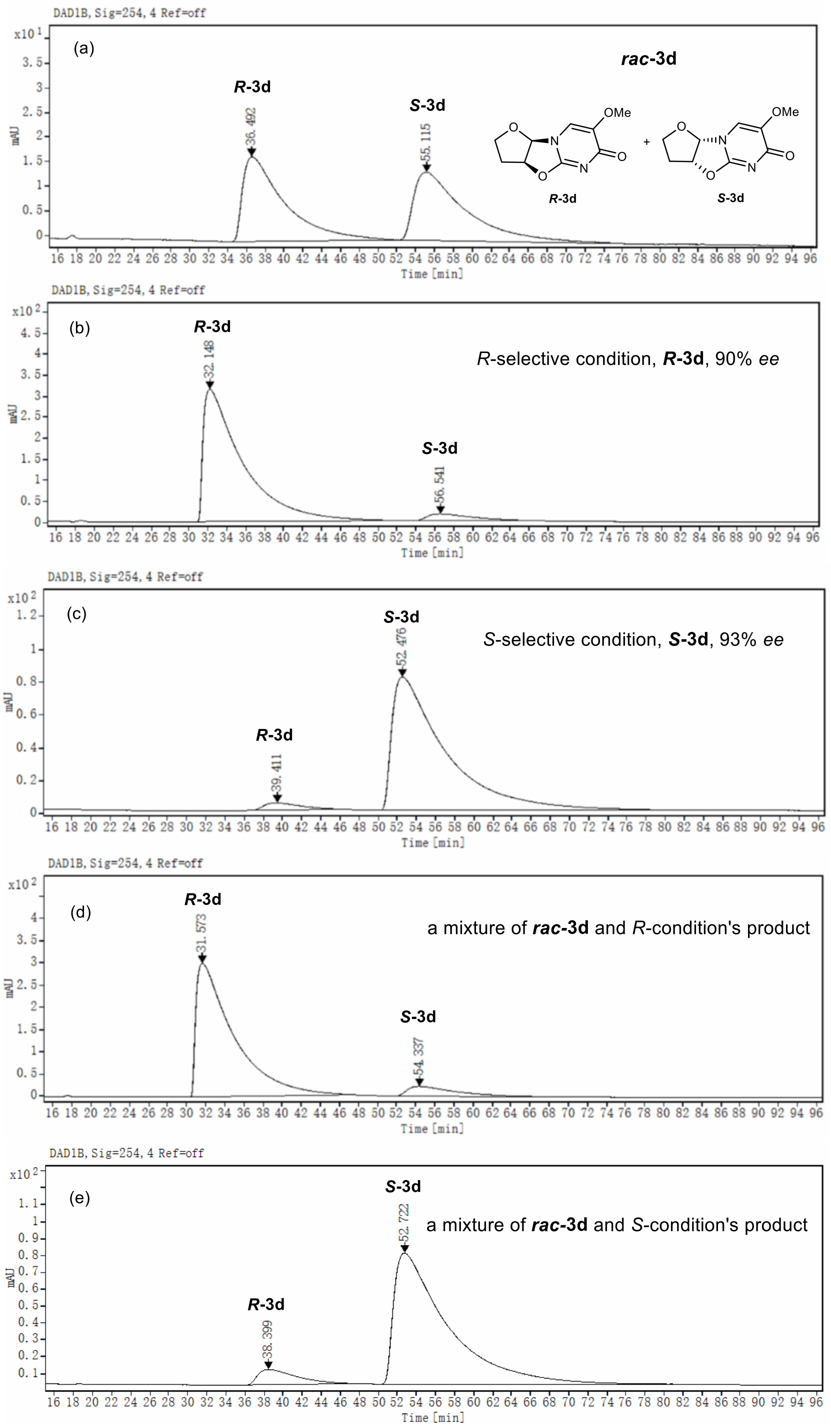

Supplementary Figure 303. HPLC Spectrum of ***R*-3d** (*R*-selective) and ***S*-3d** (*S*-selective).

Condition	Compound	RetTime [min]	Type	Width [min]	Area [mAU * s]	Height [mAU]	Area%
R-selective	***R*-3d**	32.148	BM m	3.70	91209.00	315.54	94.92
	***S*-3d**	56.541	MM m	3.73	4879.83	15.31	5.08
S-selective	***R*-3d**	39.411	MM m	3.12	1131.75	4.25	3.47
	***S*-3d**	52.476	MM m	4.55	31524.98	80.97	96.53

Chiral HPLC conditions: CHIRALCEL OD-H (4.6 x 250 mm), 70:30 *n*-hexane (0.3% CH_3COOH)/*i*-PrOH, 0.95 mL/min, UV detector at 254 nm**,** injection volume: 15 μL, T (column) = 20 °C.

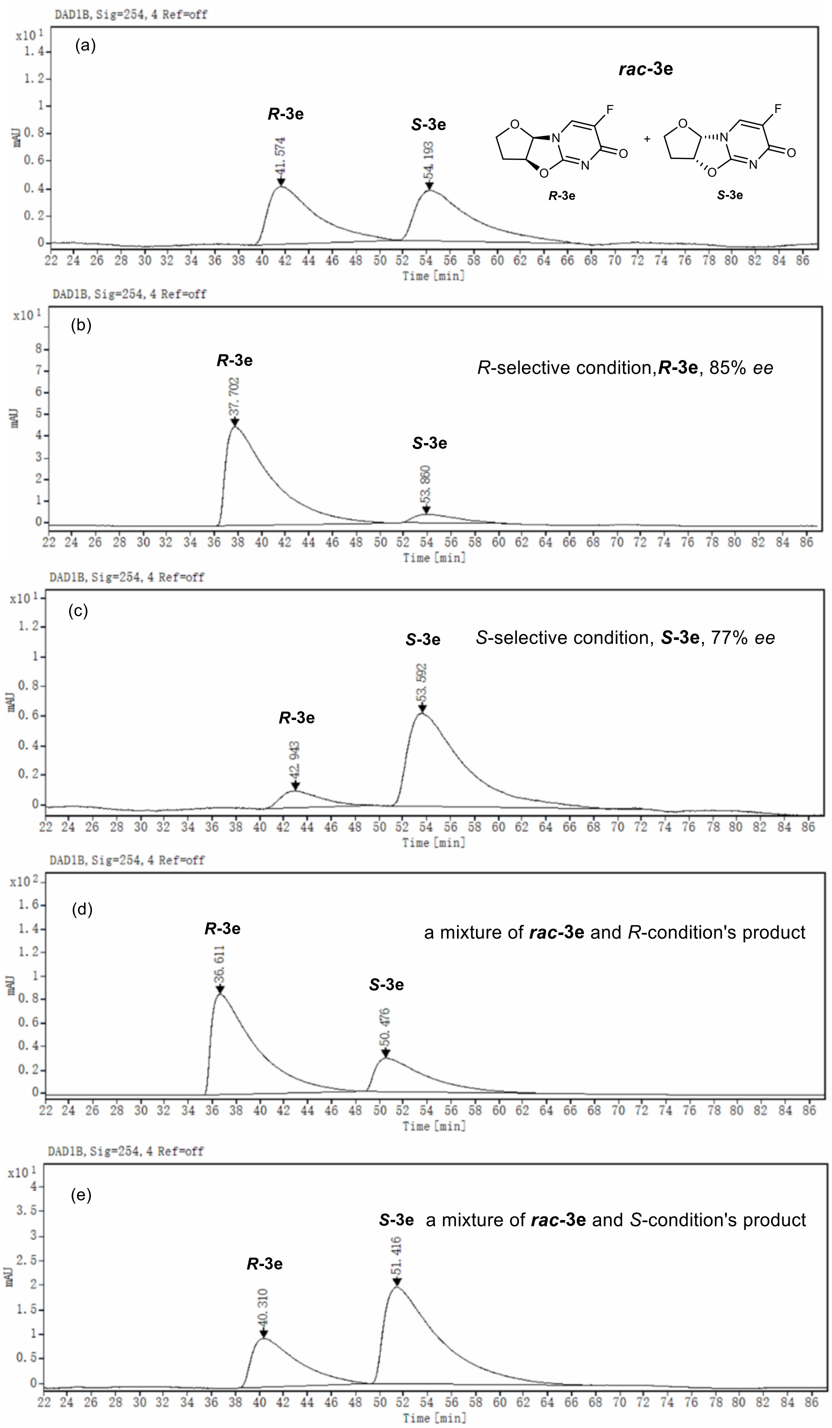

Supplementary Figure 304. HPLC Spectrum of ***R*-3e** (*R*-selective) and ***S*-3e** (*S*-selective).

Condition	Compound	RetTime [min]	Type	Width [min]	Area [mAU * s]	Height [mAU]	Area%
R-selective	***R*-3e**	37.702	BM m	3.17	12305.14	45.40	92.52
	***S*-3e**	53.860	MM m	3.03	994.80	3.84	7.48
S-selective	***R*-3e**	42.943	MM m	2.74	270.27	1.15	11.66
	***S*-3e**	53.592	MM m	3.79	2048.26	6.32	88.34

Chiral HPLC conditions: CHIRALCEL OD-H (4.6 x 250 mm), 75:25 *n*-hexane (0.3% CH_3COOH)/*i*-PrOH, 1.0 mL/min, UV detector at 254 nm**,** injection volume: 15 μL, T (column) = 20 °C.

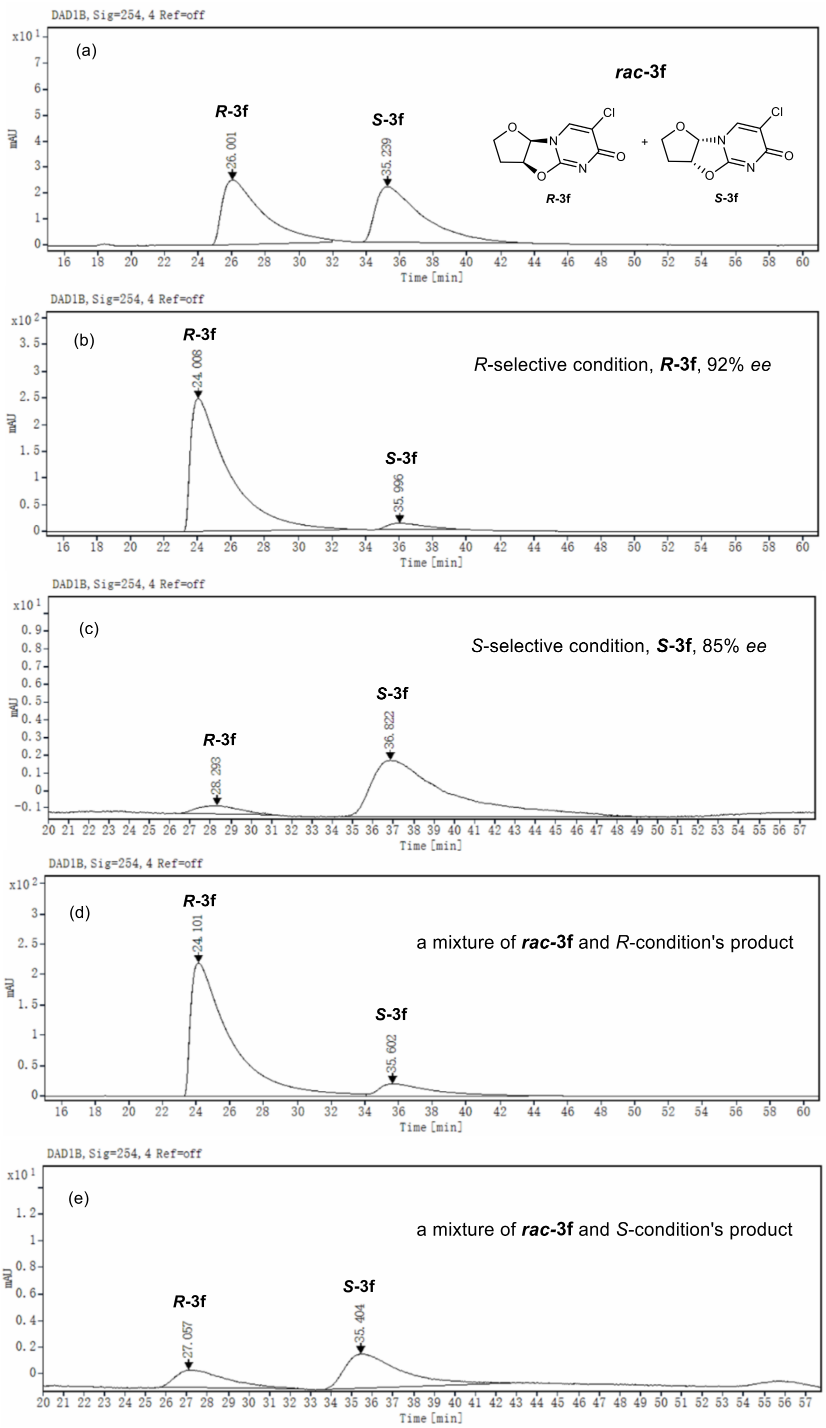

Supplementary Figure 305. HPLC Spectrum of ***R*-3f** (*R*-selective) and ***S*-3f** (*S*-selective).

Condition	Compound	RetTime [min]	Type	Width [min]	Area [mAU * s]	Height [mAU]	Area%
R-selective	***R*-3f**	24.008	BM m	2.07	39135.43	248.91	95.92
	***S*-3f**	35.996	MM m	1.77	1664.06	11.03	4.08
S-selective	***R*-3f**	28.293	MM m	1.68	67.83	0.47	7.50
	***S*-3f**	36.822	MM m	3.08	836.54	3.18	92.50

Chiral HPLC conditions: CHIRALCEL OD-H (4.6 x 250 mm), 70:30 *n*-hexane (0.3% CH_3COOH)/*i*-PrOH, 0.95 mL/min, UV detector at 254 nm**,** injection volume: 15 μL, T (column) = 20 °C.

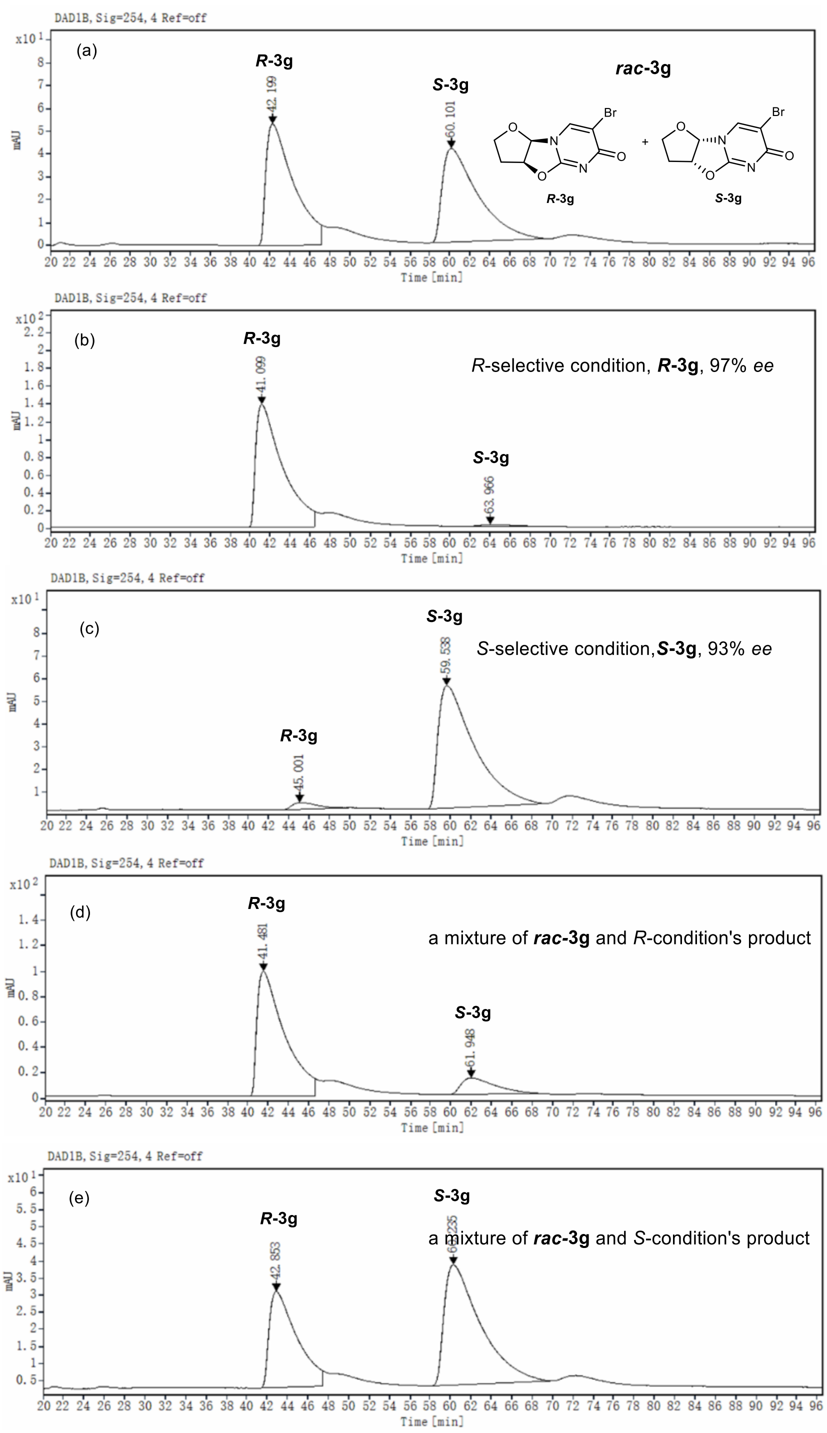

Supplementary Figure 306. HPLC Spectrum of ***R*-3g** (*R*-selective) and ***S*-3g** (*S*-selective).

Condition	Compound	RetTime [min]	Type	Width [min]	Area [mAU * s]	Height [mAU]	Area%
R-selective	***R*-3g**	41.099	BM m	2.40	25726.30	138.26	98.55
	***S*-3g**	63.966	MM m	2.38	379.71	1.87	1.45
S-selective	***R*-3g**	45.001	MM m	2.00	503.29	2.96	3.61
	***S*-3g**	59.538	BM m	2.92	13423.98	53.83	96.39

Chiral HPLC conditions: CHIRALCEL OD-H (4.6 x 250 mm), 75:25 *n*-hexane (0.3% CH_3COOH)/*i*-PrOH, 0.80 mL/min, UV detector at 254 nm**,** injection volume: 15 μL, T (column) = 20 ℃.

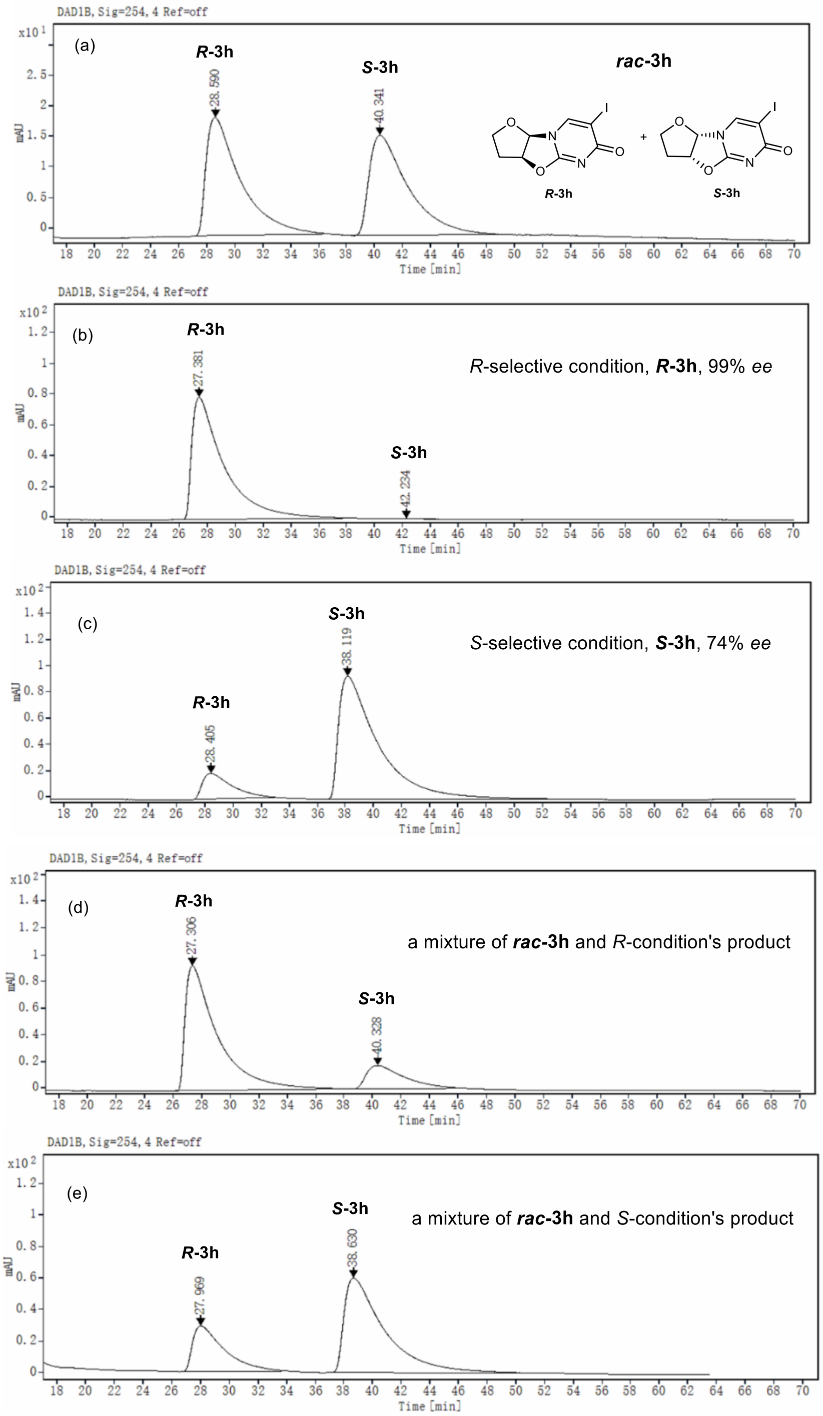

Supplementary Figure 307. HPLC Spectrum of ***R*-3h** (*R*-selective) and ***S*-3h** (*S*-selective).

Condition	Compound	RetTime [min]	Type	Width [min]	Area [mAU * s]	Height [mAU]	Area%
R-selective	***R*-3h**	27.381	BM m	2.12	12761.23	79.39	99.86
	***S*-3h**	42.234	MM m	1.29	18.33	0.17	0.14
S-selective	***R*-3h**	28.405	MM m	1.69	2790.53	19.41	13.16
	***S*-3h**	38.119	BM m	2.33	18411.02	93.45	86.84

Chiral HPLC conditions: CHIRALCEL OD-H (4.6 x 250 mm), 70:30 *n*-hexane (0.3% CH_3COOH)/*i*-PrOH, 0.95 mL/min, UV detector at 254 nm**,** injection volume: 15 μL, T (column) = 20 °C.

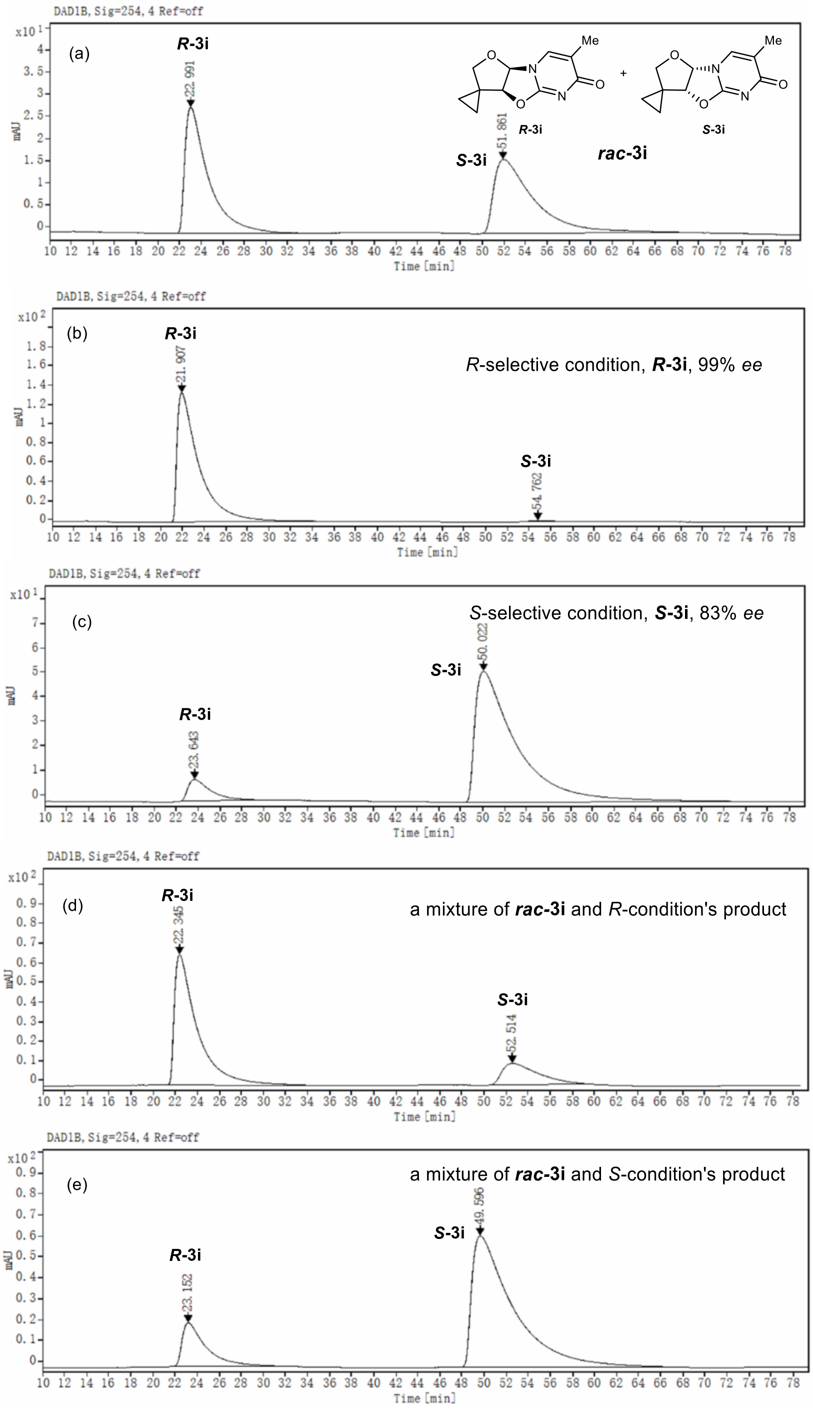

Supplementary Figure 308. HPLC Spectrum of ***R*-3i** (*R*-selective) and ***S*-3i** (*S*-selective).

Condition	Compound	RetTime [min]	Type	Width [min]	Area [mAU * s]	Height [mAU]	Area%
R-selective	***R*-3i**	21.907	BM m	1.86	18751.42	133.92	99.52
	***S*-3i**	54.762	MM m	1.32	91.07	0.81	0.48
S-selective	***R*-3i**	23.643	MM m	1.70	1260.92	8.75	8.13
	***S*-3i**	50.022	MM m	3.11	14239.67	53.72	91.87

Chiral HPLC conditions: CHIRALCEL OD-H (4.6 x 250 mm), 70:30 *n*-hexane (0.3% CH_3COOH)/*i*-PrOH, 0.80 mL/min, UV detector at 254 nm**,** injection volume: 15 μL, T (column) = 20 °C.

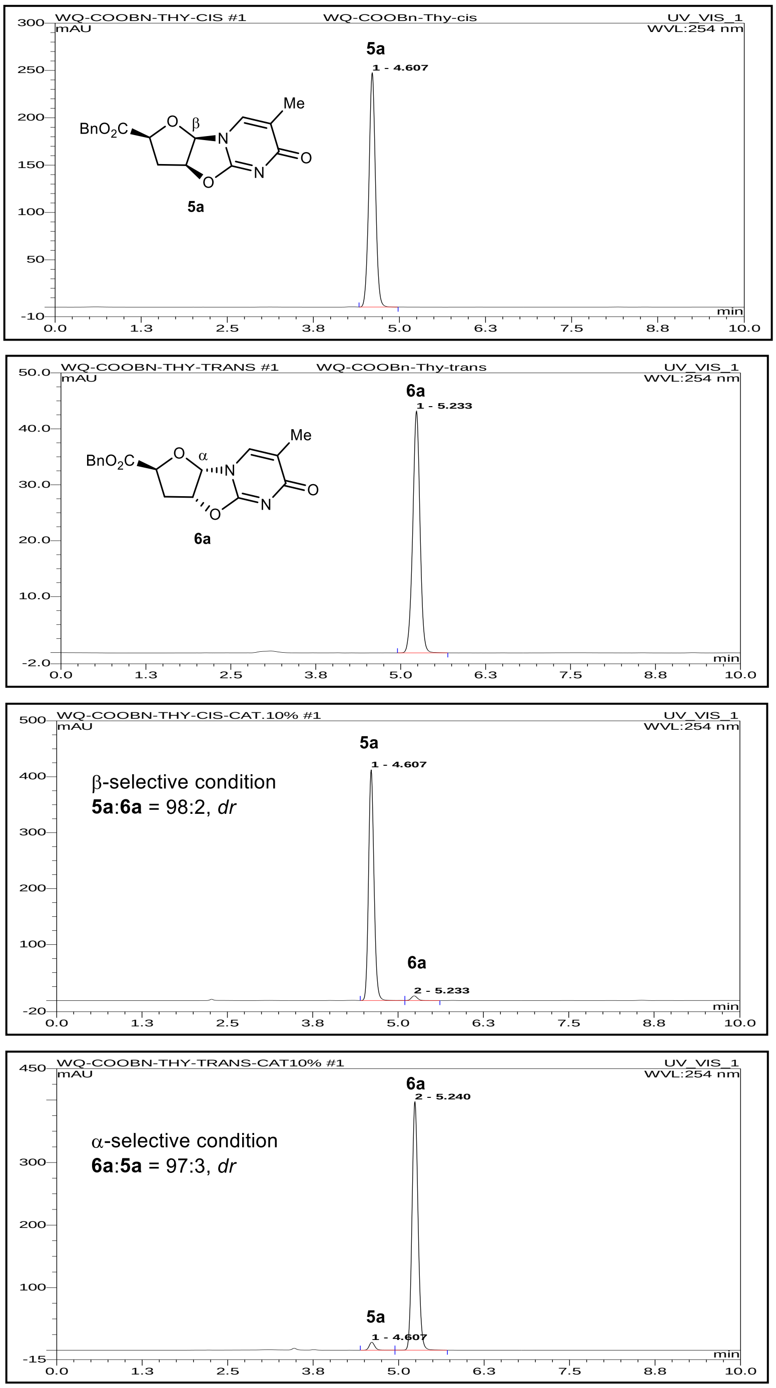

Supplementary Figure 309. HPLC Spectrum of **5a** (β-selective) and **6a** (α-selective).

Condition	Compound	Ret.Time [min]	Height [mAU]	Area [mAU*min]	Rel.Area %	Type
β-selective	**5a**	4.61	412.326	38.135	97.78	BMB
	**6a**	5.23	8.350	0.865	2.22	BMB
α-selective	**5a**	4.61	12.729	1.180	2.80	BMB
	**6a**	5.23	397.333	40.976	97.20	BMB

HPLC conditions: Agilent 5HC C18 (250 mm x 4.6 μm), 50:50 CH_3CN/H_2O, 0.8 mL/min, UV detector at 254 nm**,** injection volume: 20 μL, T (column) = 20 °C.

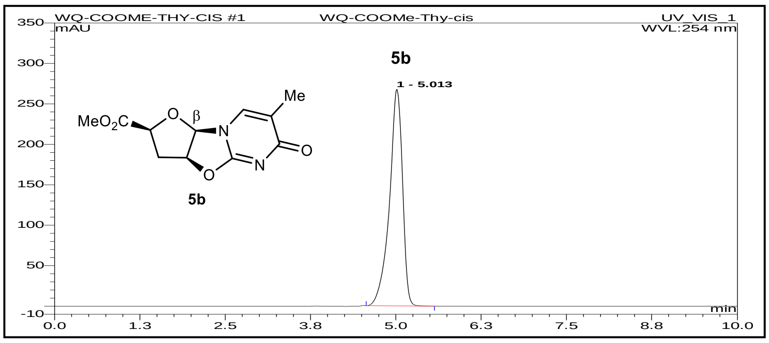

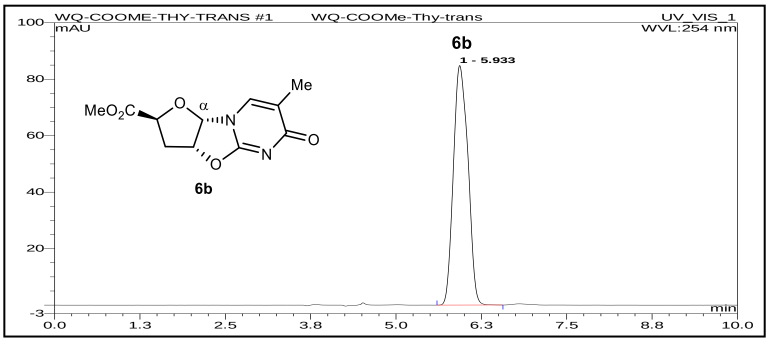

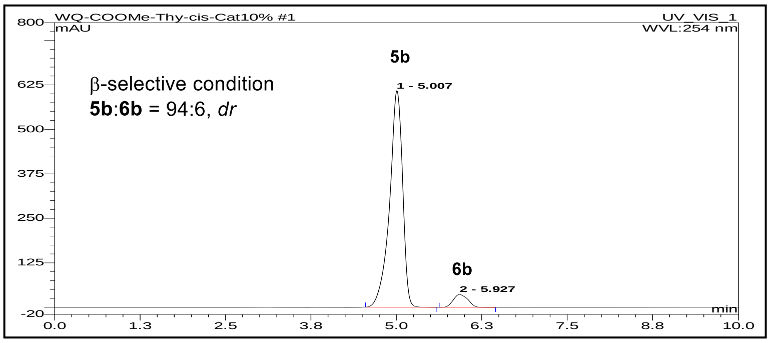

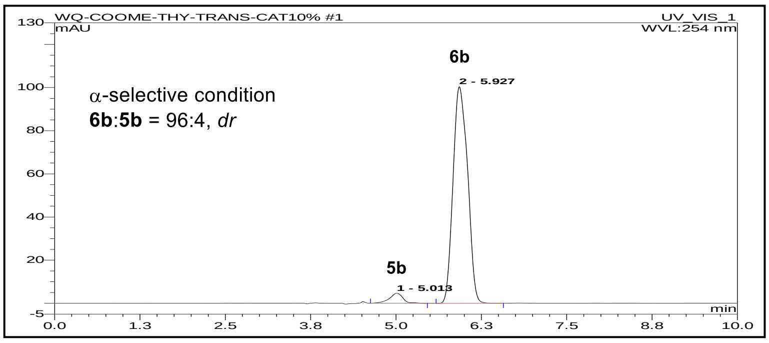

Supplementary Figure 310. HPLC Spectrum of **5b** (β-selective) and **6b** (α-selective).

Condition	Compound	Ret.Time [min]	Height [mAU]	Area [mAU*min]	Rel.Area %	Type
β-selective	**5b**	5.01	608.300	134.624	93.77	BMB
	**6b**	5.93	36.277	8.943	6.23	BMB
α-selective	**5b**	5.01	4.659	1.057	4.08	BMB
	**6b**	5.93	100.438	24.867	95.92	BMB

HPLC conditions: Agilent 5HC C18 (250 mm x 4.6 μm), 30:70 MeOH/H_2O, 0.8 mL/min, UV detector at 254 nm**,** injection volume: 20 μL, T (column) = 20 ℃.

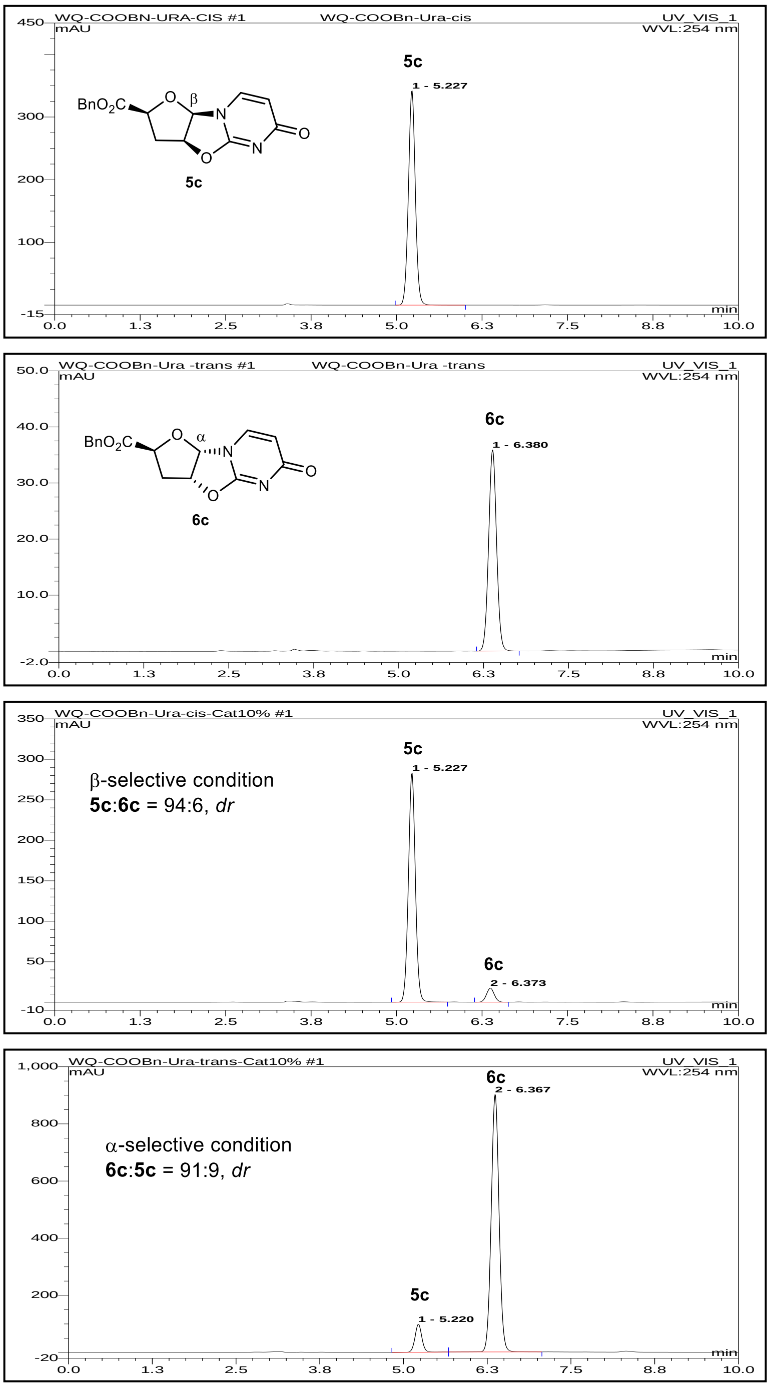

Supplementary Figure 311. HPLC Spectrum of **5c** (β-selective) and **6c** (α-selective).

Condition	Compound	Ret.Time [min]	Height [mAU]	Area [mAU*min]	Rel.Area %	Type
β-selective	**5c**	5.23	282.538	33.510	93.51	BMB
	**6c**	6.37	17.262	2.325	6.49	BMB
α-selective	**5c**	5.22	98.102	11.662	8.69	BMB
	**6c**	6.37	900.710	122.515	91.31	BMB

HPLC conditions: Agilent 5HC C18 (250 mm x 4.6 μm), 40:60 MeOH/H_2O, 0.8 mL/min, UV detector at 254 nm, injection volume: 20 μL, T (column) = 20 °C.

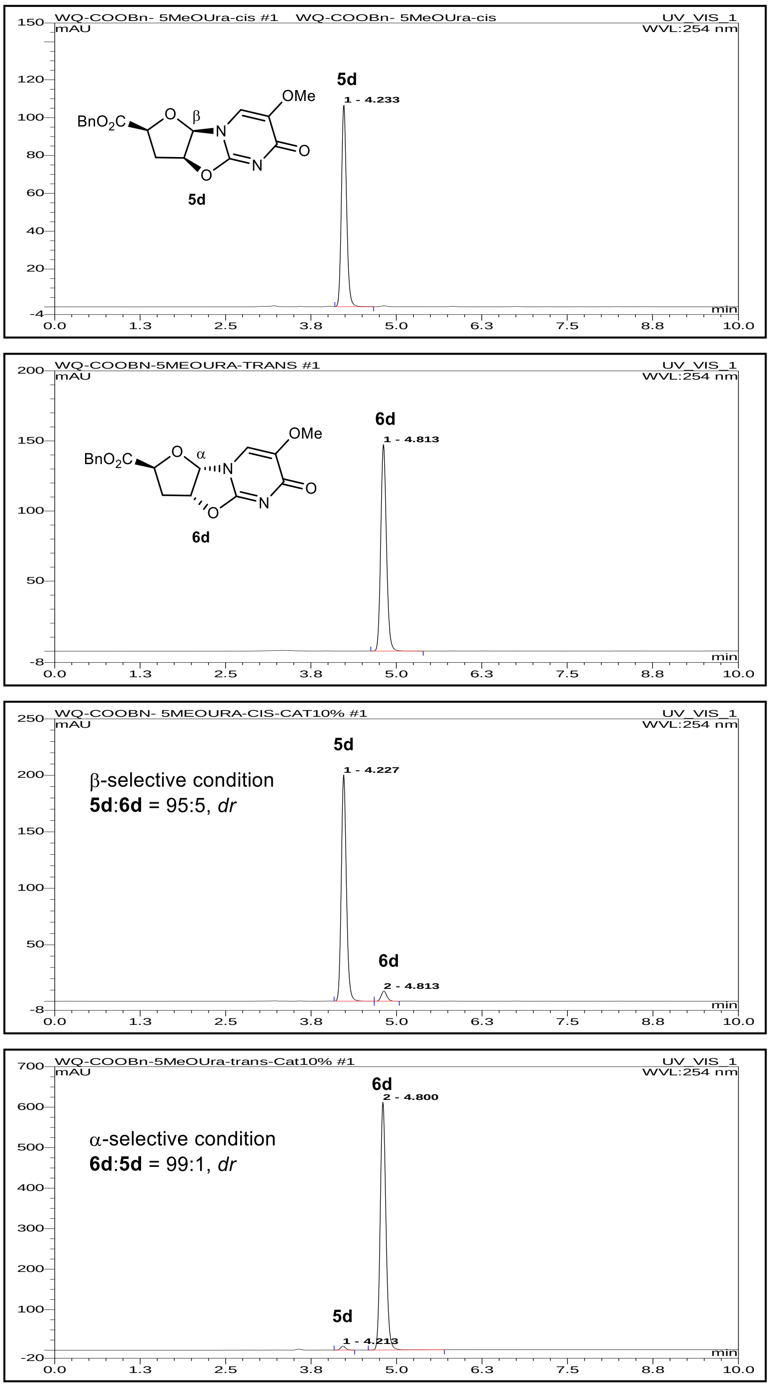

Supplementary Figure 312. HPLC Spectrum of **5d** (β-selective) and **6d** (α-selective).

Condition	Compound	Ret.Time [min]	Height [mAU]	Area [mAU*min]	Rel.Area %	Type
β-selective	**5d**	4.23	200.209	17.352	95.31	BMB
	**6d**	4.81	8.906	0.855	4.69	BMB
α-selective	**5d**	4.21	9.628	0.821	1.37	BMB
	**6d**	4.80	611.891	59.173	98.63	BMB

HPLC conditions: Agilent 5HC C18 (250 mm x 4.6 μm), 50:50 CH_3CN/H_2O, 0.8 mL/min, UV detector at 254 nm**,** injection volume: 20 μL, T (column) = 20 °C.

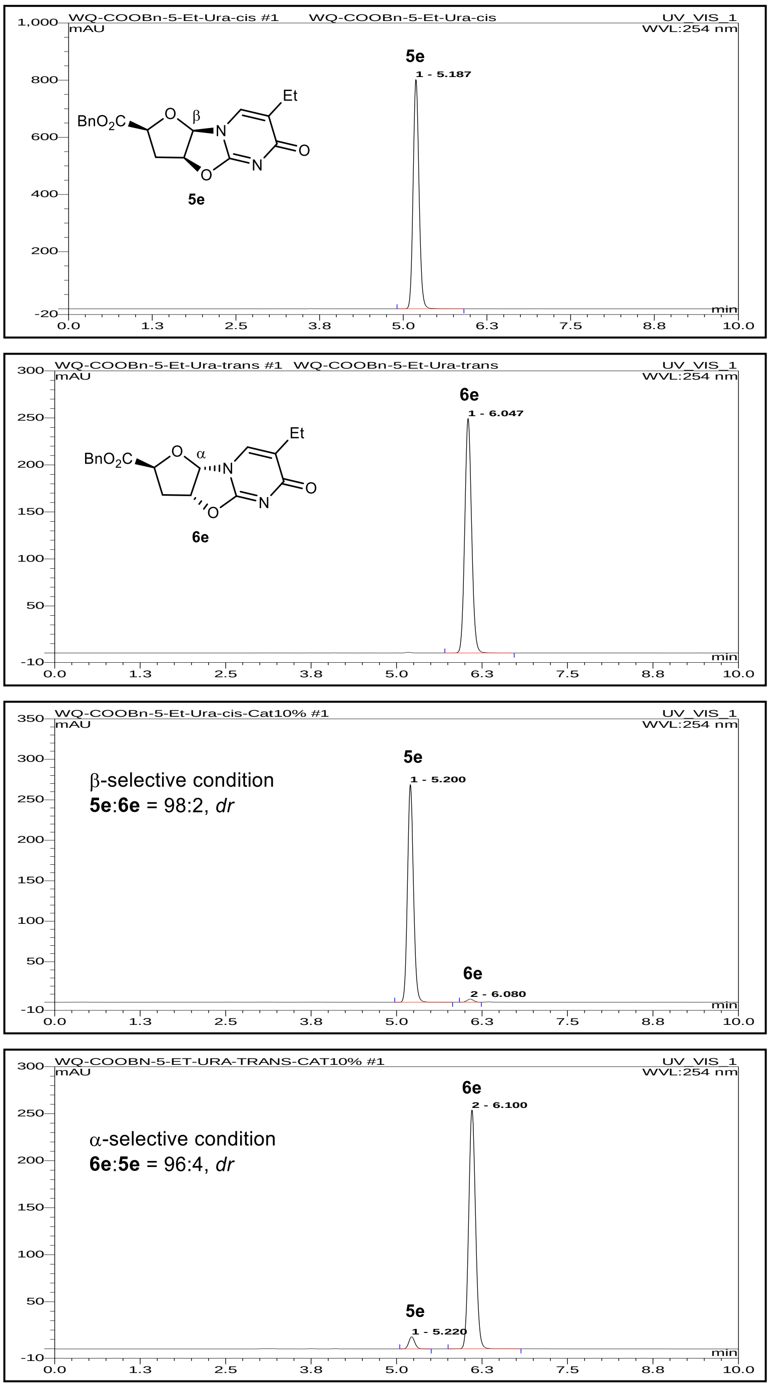

Supplementary Figure 313. HPLC Spectrum of **5e** (β-selective) and **6e** (α-selective).

Condition	Compound	Ret.Time [min]	Height [mAU]	Area [mAU*min]	Rel.Area %	Type
β-selective	**5e**	5.20	268.736	27.246	98.46	BMB
	**6e**	6.08	3.765	0.427	1.54	BMB
α-selective	**5e**	5.22	12.967	1.324	4.24	BMB
	**6e**	6.10	253.911	29.902	95.76	BMB

HPLC conditions: Agilent 5HC C18 (250 mm x 4.6 μm), 50:50 CH_3CN/H_2O, 0.8 mL/min, UV detector at 254 nm**,** injection volume: 20 μL, T (column) = 20 °C.

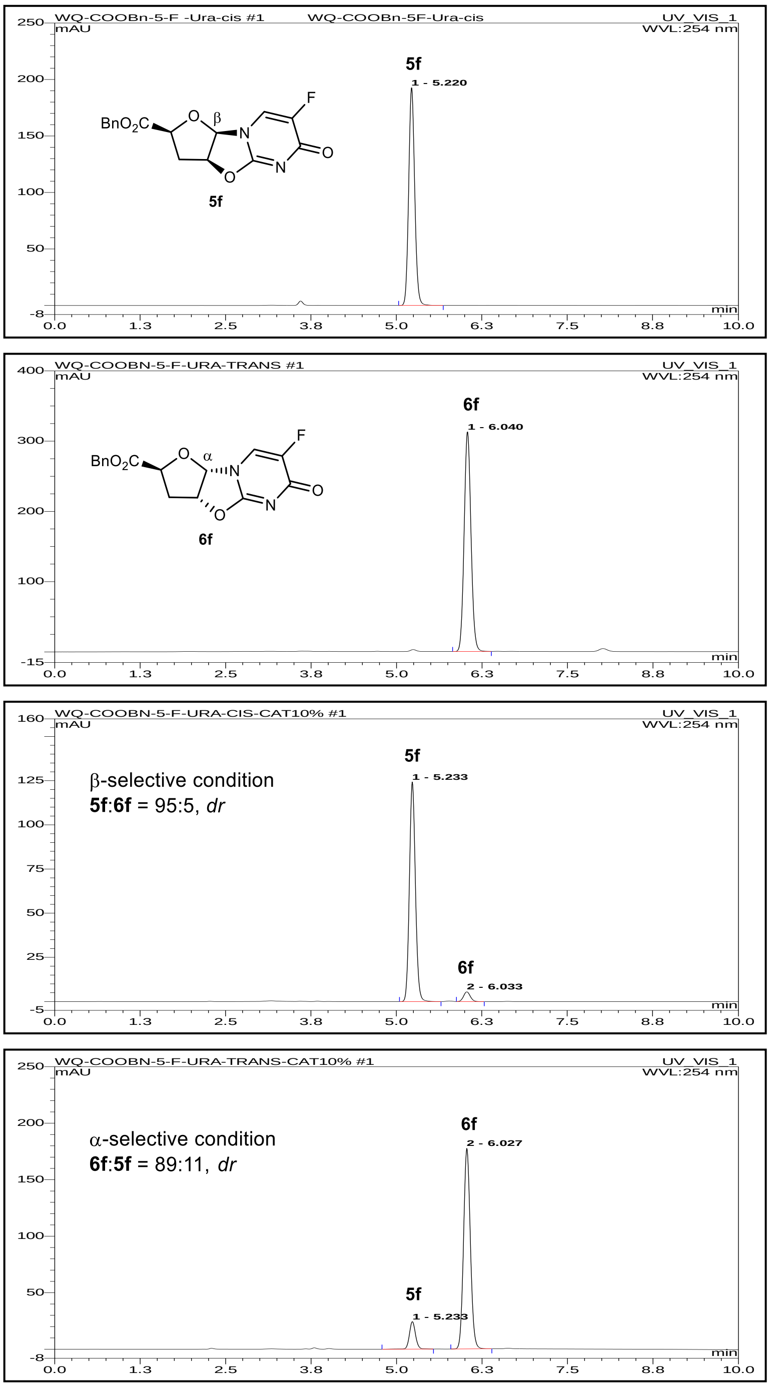

Supplementary Figure 314. HPLC Spectrum of **5f** (β-selective) and **6f** (α-selective).

Condition	Compound	Ret.Time [min]	Height [mAU]	Area [mAU*min]	Rel.Area %	Type
β-selective	**5f**	5.23	124.323	12.693	95.29	BMB
	**6f**	6.03	5.496	0.627	4.71	BMB
α-selective	**5f**	5.23	24.358	2.528	10.98	BMB
	**6f**	6.03	177.492	20.502	89.02	BMB

HPLC conditions: Agilent 5HC C18 (250 mm x 4.6 μm), 50:50 CH_3CN/H_2O, 0.8 mL/min, UV detector at 254 nm**,** injection volume: 20 μL, T (column) = 20 ℃.

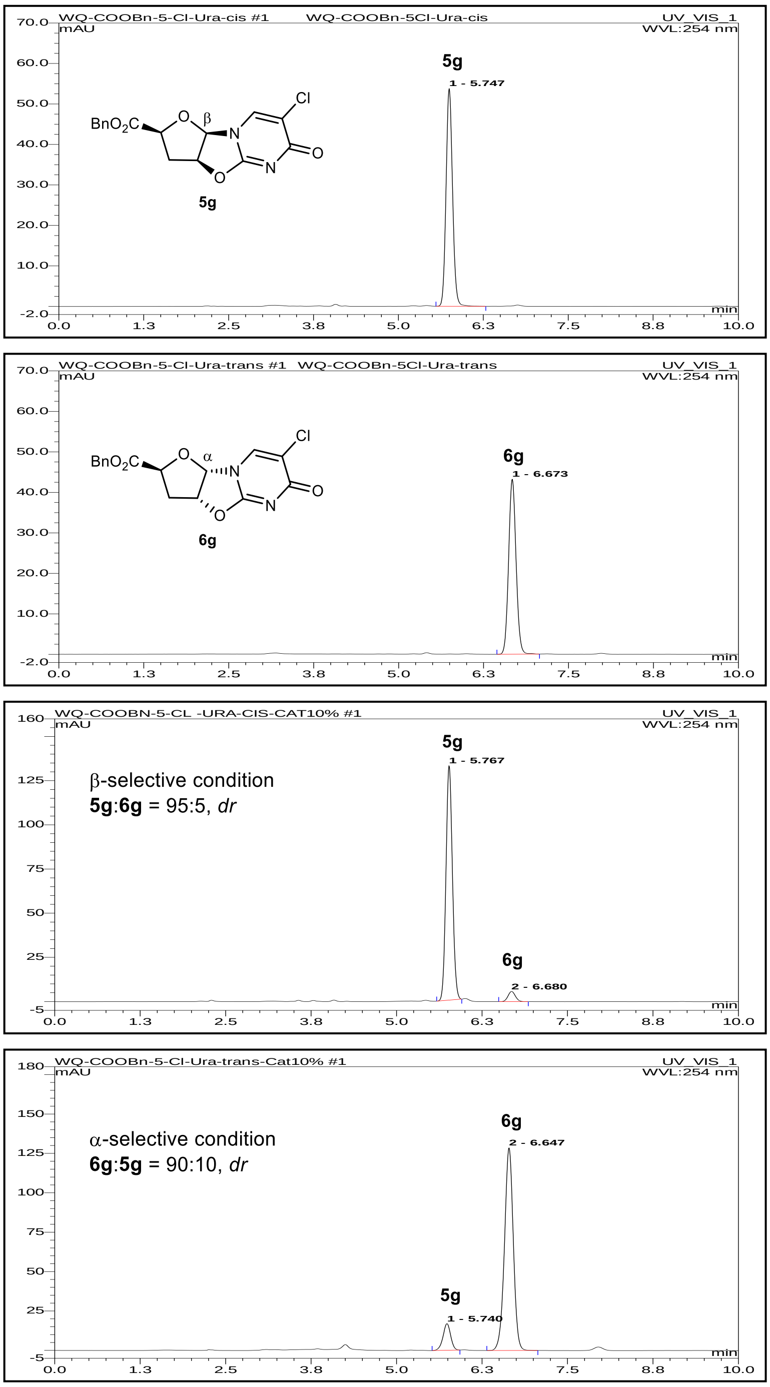

Supplementary Figure 315. HPLC Spectrum of **5g** (β-selective) and **6g** (α-selective).

Condition	Compound	Ret.Time [min]	Height [mAU]	Area [mAU*min]	Rel.Area %	Type
β-selective	**5g**	5.77	132.685	14.441	95.24	BMB
	**6g**	6.68	5.760	0.722	4.76	BMB
α-selective	**5g**	5.74	16.891	2.188	10.22	BMB
	**6g**	6.65	128.601	19.220	89.78	BMB

HPLC conditions: Agilent 5HC C18 (250 mm x 4.6 μm), 50:50 CH_3CN/H_2O, 0.8 mL/min, UV detector at 254 nm**,** injection volume: 20 μL, T (column) = 20 °C.

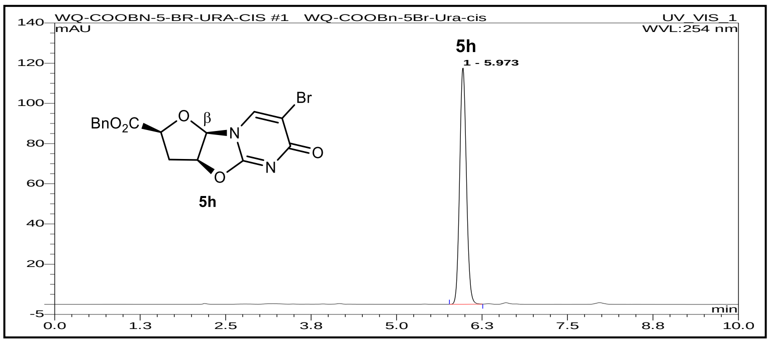

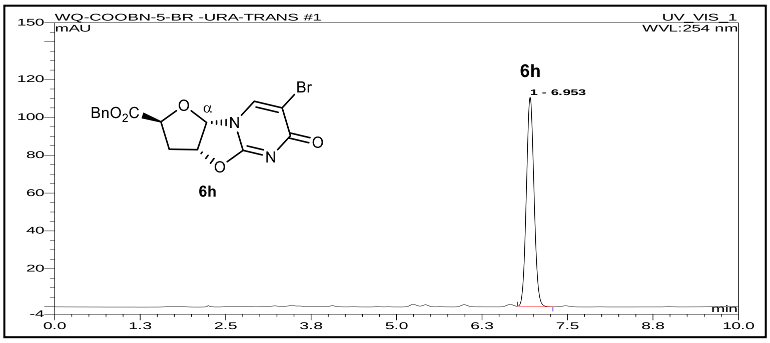

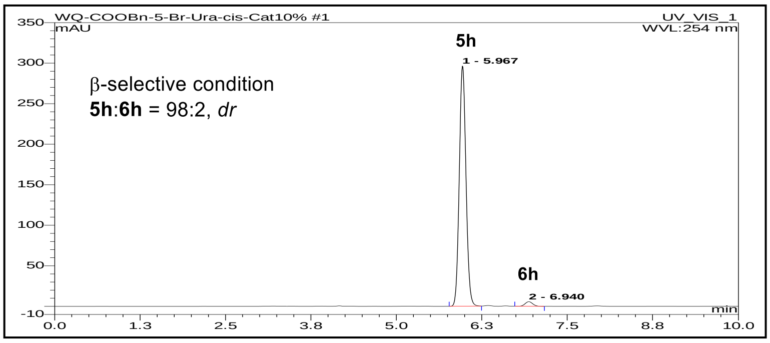

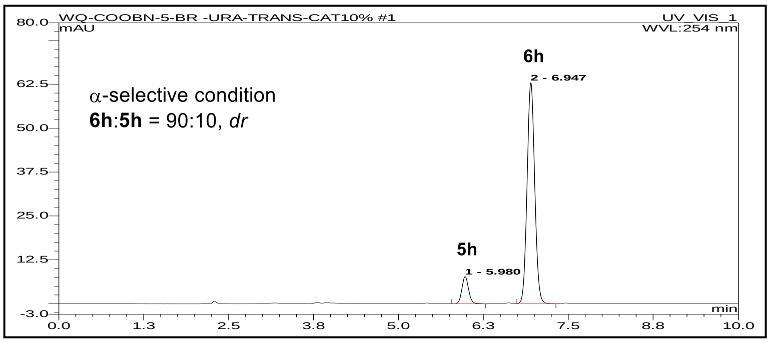

Supplementary Figure 316. HPLC Spectrum of **5h** (β-selective) and **6h** (α-selective).

Condition	Compound	Ret.Time [min]	Height [mAU]	Area [mAU*min]	Rel.Area %	Type
β-selective	**5h**	5.97	296.247	33.677	97.87	BMB
	**6h**	6.94	5.697	0.731	2.13	BMB
α-selective	**5h**	5.98	7.714	0.884	9.75	BMB
	**6h**	6.95	62.873	8.187	90.25	BMB

HPLC conditions: Agilent 5HC C18 (250 mm x 4.6 μm), 50:50 CH_3CN/H_2O, 0.8 mL/min, UV detector at 254 nm**,** injection volume: 20 μL, T (column) = 20 ℃.

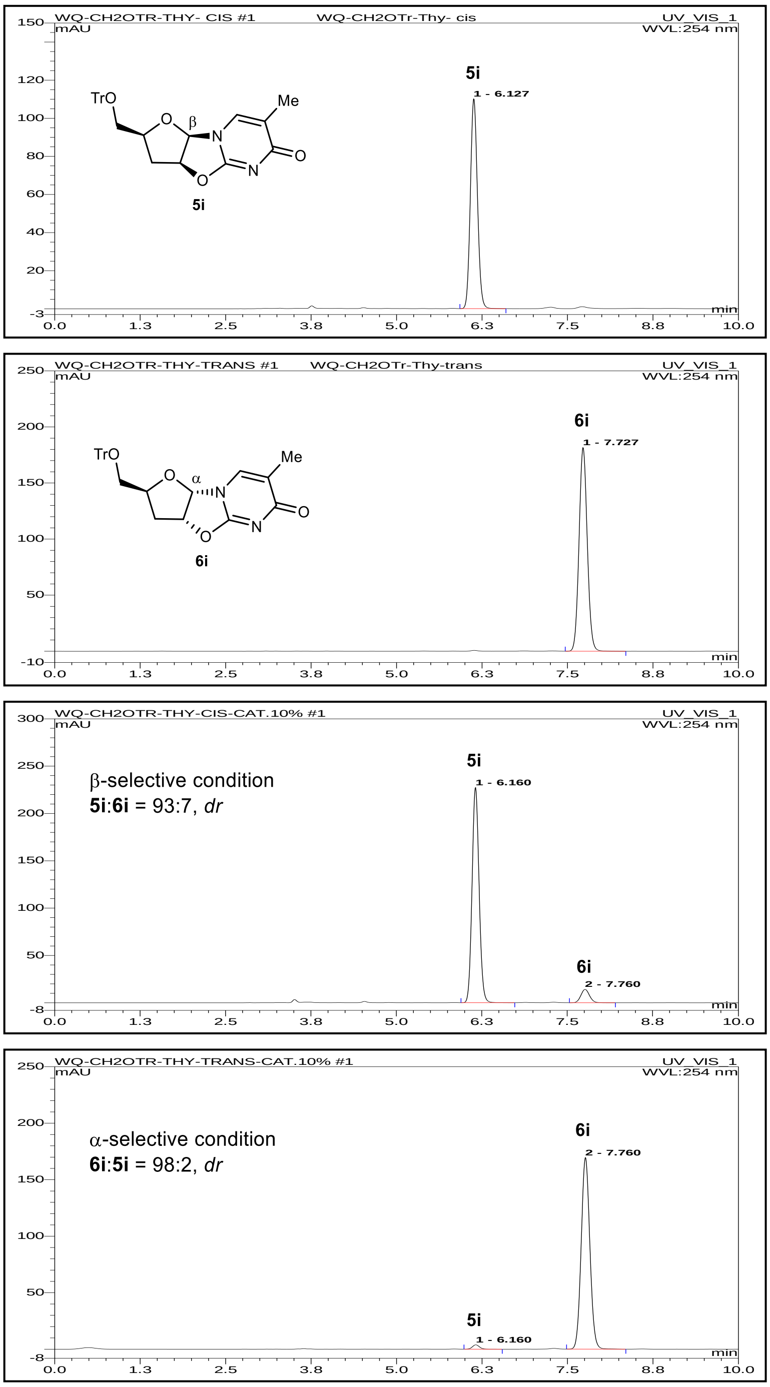

Supplementary Figure 317. HPLC Spectrum of **5i** (β-selective) and **6i** (α-selective).

Condition	Compound	Ret.Time [min]	Height [mAU]	Area [mAU*min]	Rel.Area %	Type
β-selective	**5i**	6.16	227.433	27.169	92.87	BMB
	**6i**	7.76	14.114	2.085	7.13	BMB
α-selective	**5i**	6.16	3.810	0.455	1.79	BMB
	**6i**	7.76	169.484	24.956	98.21	BMB

HPLC conditions: Agilent 5HC C18 (250 mm x 4.6 μm), 70:30 CH_3CN/H_2O, 0.8 mL/min, UV detector at 254 nm**,** injection volume: 20 μL, T (column) = 20 °C.

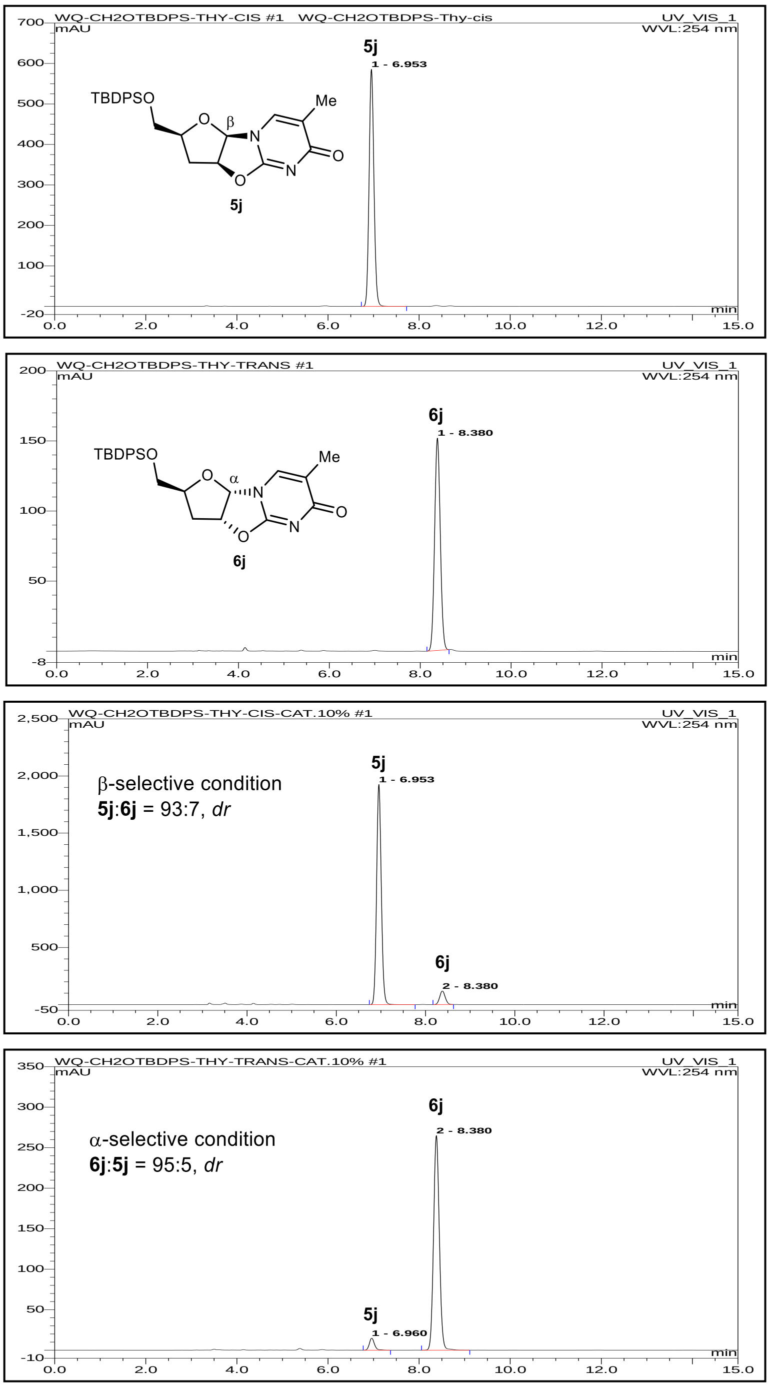

Supplementary Figure 318. HPLC Spectrum of **5j** (β-selective) and **6j** (α-selective).

Condition	Compound	Ret.Time [min]	Height [mAU]	Area [mAU*min]	Rel.Area %	Type
β-selective	**5j**	6.95	1925.310	234.360	93.20	BMB
	**6j**	8.38	118.407	17.105	6.80	BMB
α-selective	**5j**	6.96	14.779	1.918	4.69	BMB
	**6j**	8.38	264.896	38.957	95.31	BMB

HPLC conditions: Agilent 5HC C18 (250 mm x 4.6 μm), 80:20 CH_3CN/H_2O, 0.8 mL/min, UV detector at 254 nm**,** injection volume: 20 μL, T (column) = 20 °C.

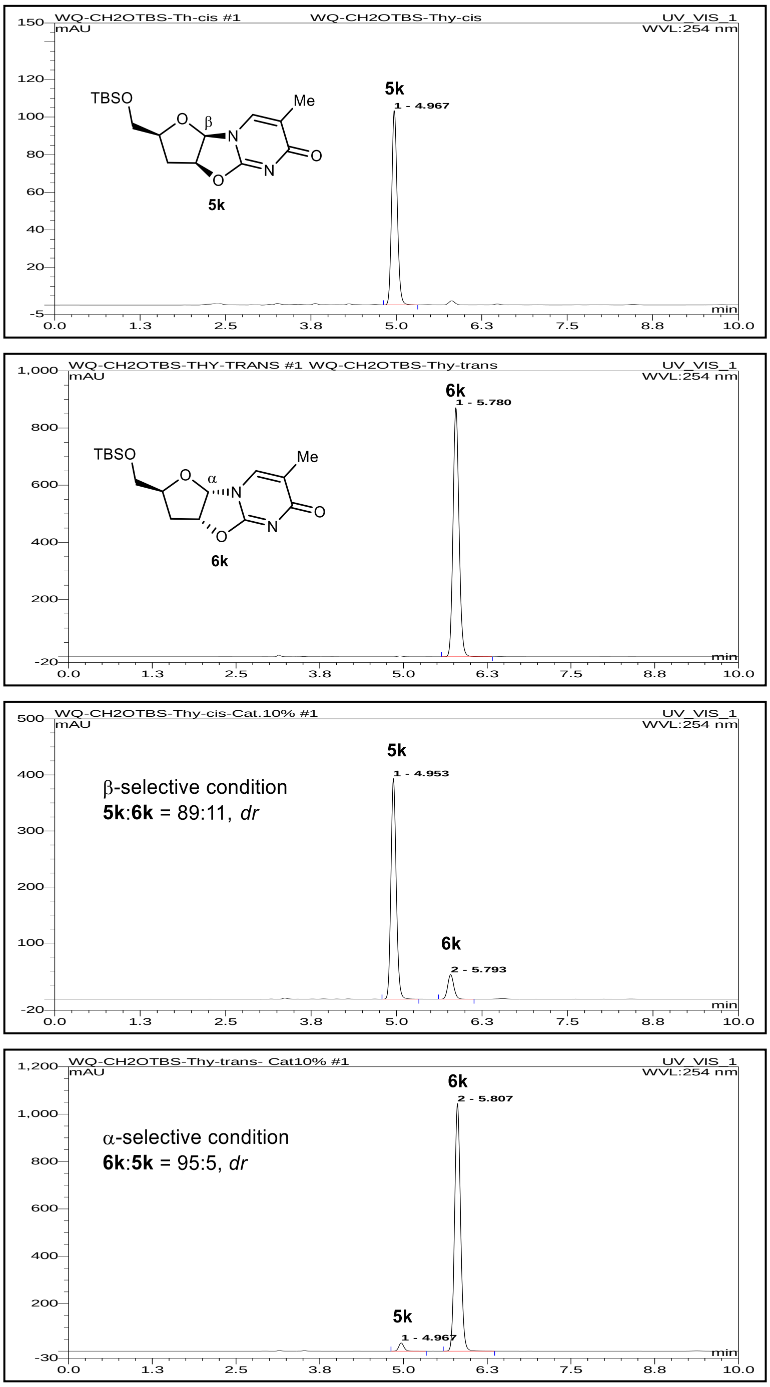

Supplementary Figure 319. HPLC Spectrum of **5k** (β-selective) and **6k** (α-selective).

Condition	Compound	Ret.Time [min]	Height [mAU]	Area [mAU*min]	Rel.Area %	Type
β-selective	**5k**	4.95	393.285	35.276	88.68	BMB
	**6k**	5.79	43.706	4.504	11.32	BMB
α-selective	**5k**	4.97	34.645	3.102	2.80	BMB
	**6k**	5.81	1044.549	107.578	97.20	BMB

HPLC conditions: Agilent 5HC C18 (250 mm x 4.6 μm), 75:25 CH_3CN/H_2O, 0.8 mL/min, UV detector at 254 nm**,** injection volume: 20 μL, T (column) = 20 ℃.

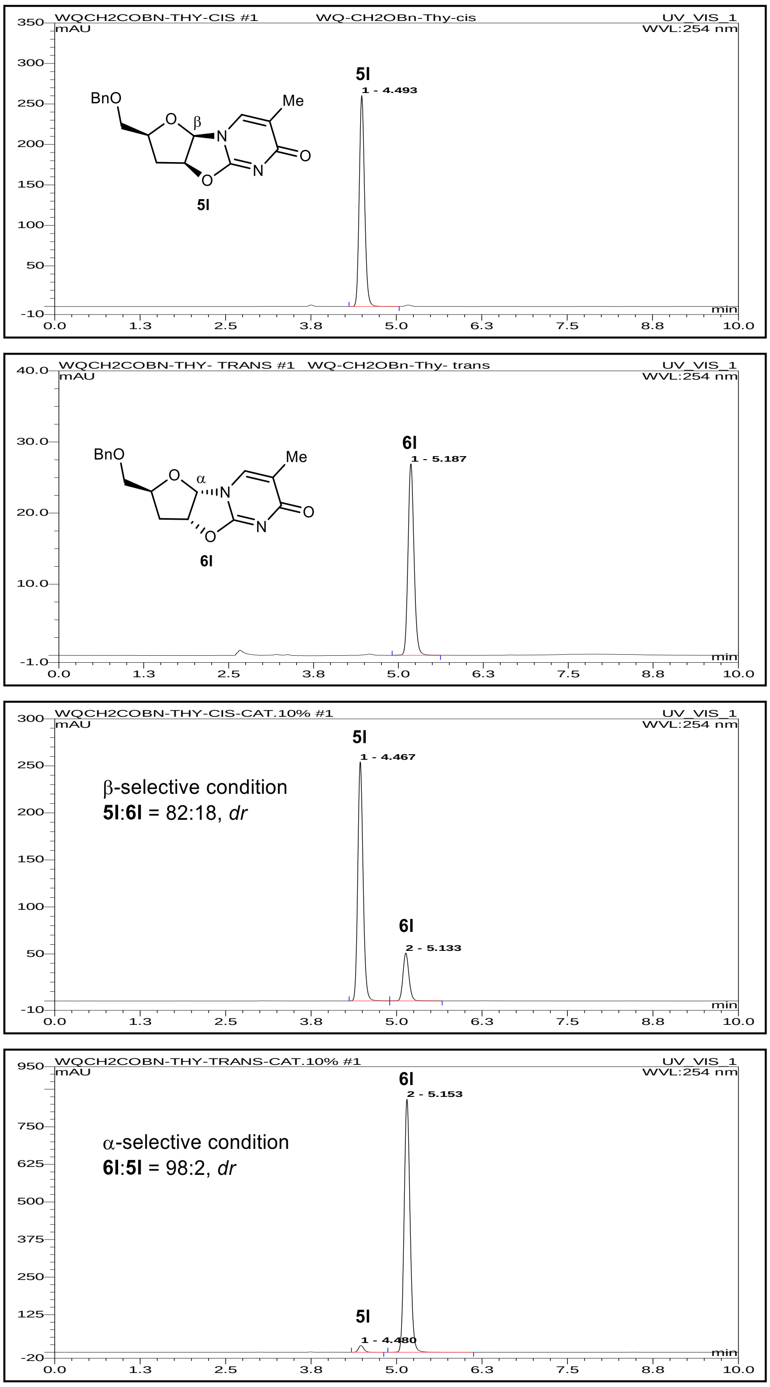

Supplementary Figure 320. HPLC Spectrum of **5l** (β-selective) and **6l** (α-selective).

Condition	Compound	Ret.Time [min]	Height [mAU]	Area [mAU*min]	Rel.Area %	Type
β-selective	**5l**	4.47	254.15	22.824	81.61	BMB
	**6l**	5.13	50.936	5.144	18.39	BMB
α-selective	**5l**	4.48	22.649	2.034	2.33	BMB
	**6l**	5.15	842.180	85.086	97.67	BMB

HPLC conditions: Agilent 5HC C18 (250 mm x 4.6 μm), 50:50 CH_3CN/H_2O, 0.8 mL/min, UV detector at 254 nm**,** injection volume: 20 μL, T (column) = 20 ℃.

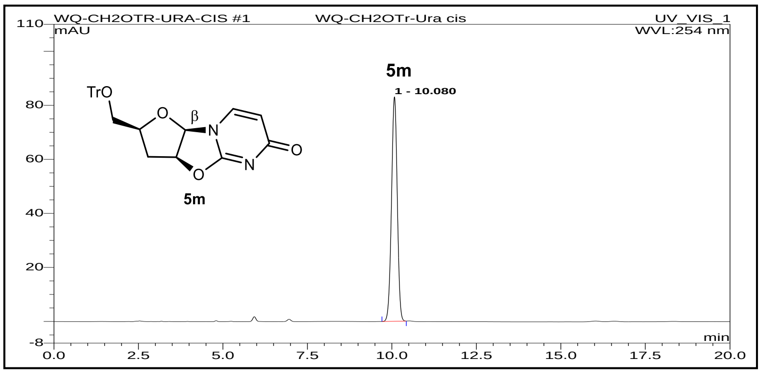

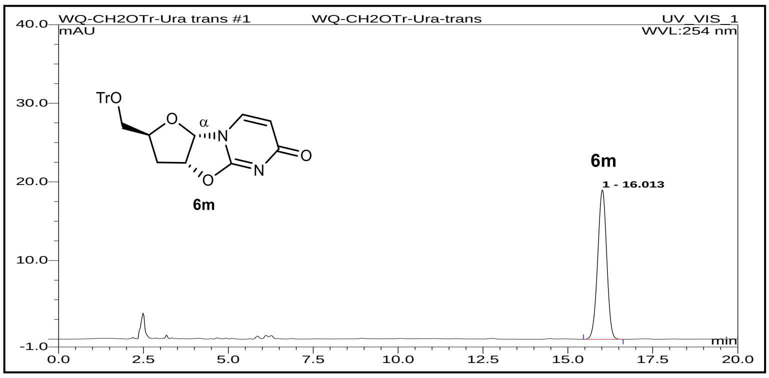

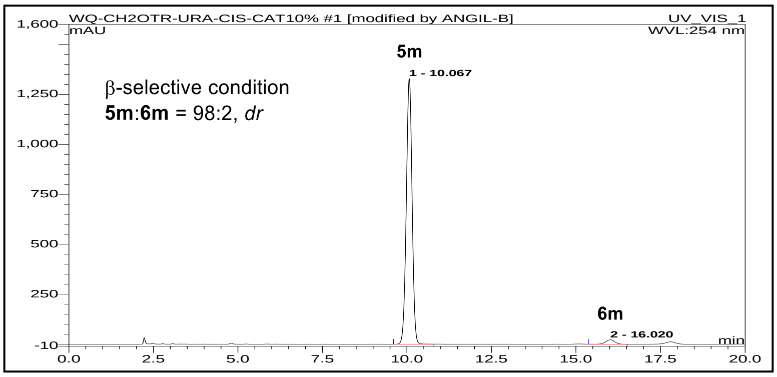

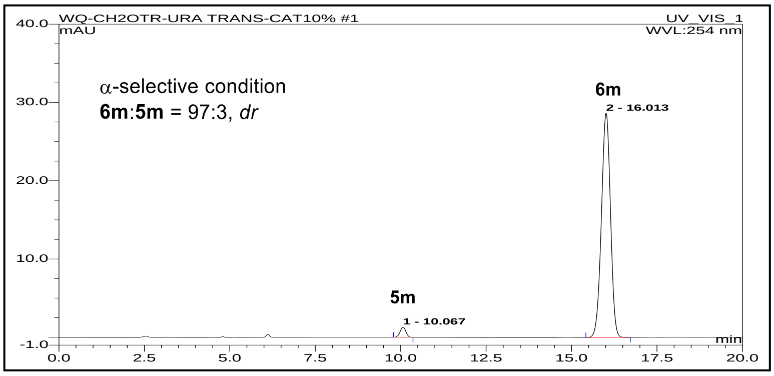

Supplementary Figure 321. HPLC Spectrum of **5m** (β-selective) and **6m** (α-selective).

Condition	Compound	Ret.Time [min]	Height [mAU]	Area [mAU*min]	Rel.Area %	Type
β-selective	**5m**	10.07	1328.413	15798.530	97.53	BMB
	**6m**	16.02	21.696	400.680	2.47	BMB
α-selective	**5m**	10.07	1.292	15.230	2.79	BMB
	**6m**	16.01	28.657	530.190	97.21	BMB

HPLC conditions: Agilent 5HC C18 (250 mm x 4.6 μm), 50:50 CH_3CN/H_2O, 1.0 mL/min, UV detector at 254 nm**,** injection volume: 20 μL, T (column) = 20 °C.

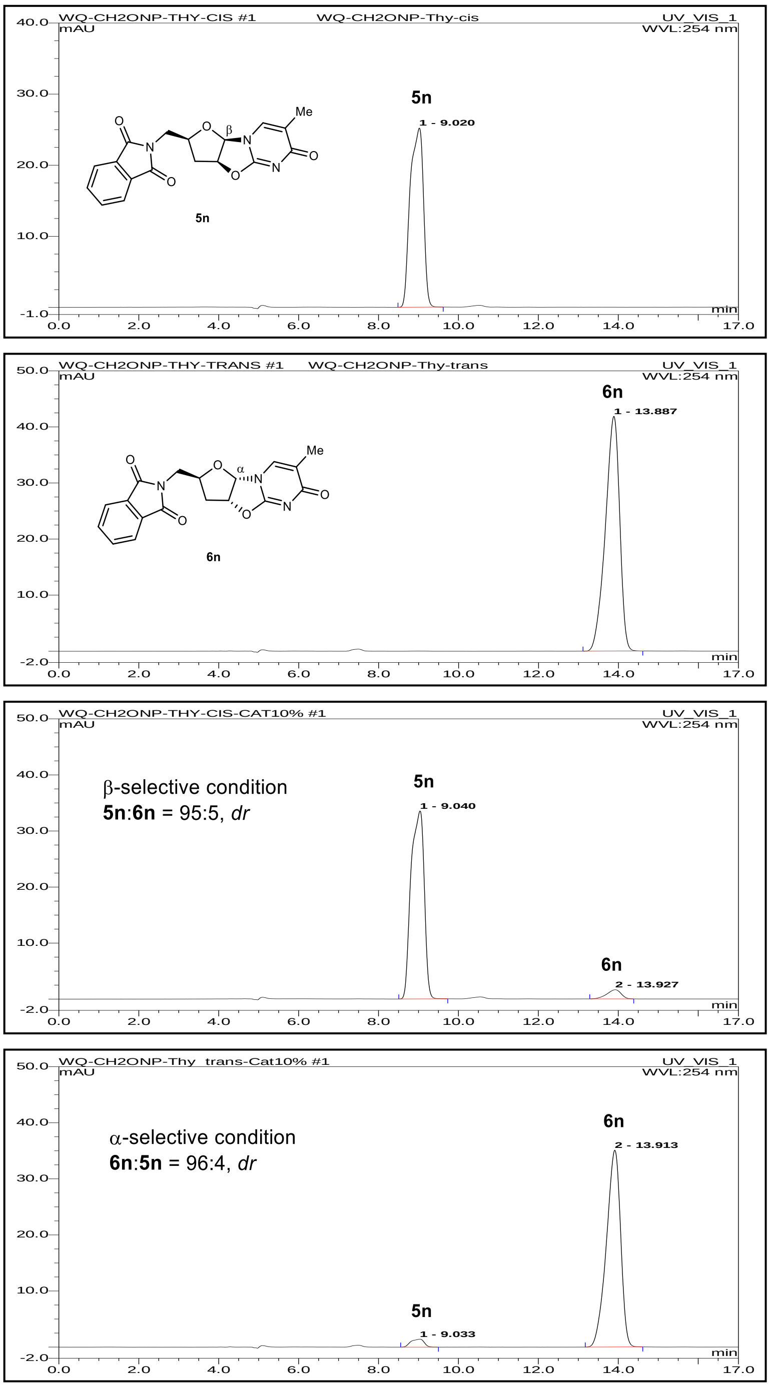

Supplementary Figure 322. HPLC Spectrum of **5n** (β-selective) and **6n** (α-selective).

Condition	Compound	Ret.Time [min]	Height [mAU]	Area [mAU*min]	Rel.Area %	Type
β-selective	**5n**	9.04	33.529	12.842	95.01	BMB
	**6n**	13.93	1.637	0.675	4.99	BMB
α-selective	**5n**	9.03	1.417	0.545	3.62	BMB
	**6n**	13.91	35.100	14.510	96.38	BMB

HPLC conditions: Agilent 5HC C18 (250 mm x 4.6 μm), 25:75 CH_3CN/H_2O, 0.8 mL/min, UV detector at 254 nm**,** injection volume: 20 μL, T (column) = 20 ℃.

6 Supplementary References

1. Albert, M., Souza, D. D., Feiertag, P. & Honig, H. A new concept for the preparation of β-L- and β-D-2′, 3′-dideoxynucleoside analogues. *Org. Lett.* **4**, 3251−3254 (2002).
2. Horton, D., Chen, K., No, Z. & Lee, H. C. Synthesis of 3′-C-substituted thymidine derivatives by free-radical techniques: scope and limitations. *Carbohydr. Res.* **342**, 259−267 (2007).
3. Sekine, M. & Nakanishi, T. Facile synthesis of 3'-O-methylthymidine and 3'-deoxythymidine and related deoxygenated thymidine derivative: a new method for selective deoxygenation of secondary hydroxy groups. *J. Org. Chem.* **55**, 924−928 (1990).
4. Becouarn, S., Czernecki, S. & Valé ry, J. M. Efficient transformation of thymidine into 2′, 3′-didehydro-2′, 3′-dideoxy-thymidine (d4T) involving opening of a 2, 3′-anhydro derivative by phenylselenol. *Nucleosides & Nucleotides.* **14(6)**, 1227−1232 (1995).
5. Thiesen, L. J. H., Cabral, N., Silva, M. J. & Bezerra, G. Larger laboratory scale synthesis of 5-methyluridine and formal synthesis of its L-enantiomer. *Arkivoc*. 249−264 (2017).
6. Bouazza, A. H., Zerrouki, R. & Krausz, P. New acyclonucleosides: synthesis and anti-HIV activity. *Nucleosides & Nucleotides*. **24(8)**, 1249−1263 (2005).
7. Hu, T. & Shen, J. "One-pot" synthesis of molnupiravir from cytidine. *Org. Process Res. Dev.* **26(2)**, 358−364 (2022).
8. Zhao, Y. & Truhlar, D.G. The M06 suite of density functionals for main group thermochemistry, thermochemical kinetics, noncovalent interactions, excited states, and transition elements. *Theor. Chem. Acc.* **120**, 215−241 (2008).
9. Krishnan, R. B. J. S., Binkley, J. S., Seeger, R. & Pople, J. A. Self-consistent molecular orbital methods. A basis set for correlated wave functions. *J. Chem. Phys.* **72**, 650 (1980).
10. Hay, P. J. & Wadt, W. R. *Ab initio* effective core potentials for molecular calculations. Potentials for the transition metal atoms Sc to Hg. *J. Chem. Phys.* **82(1)**, 270−283 (1985).
11. Fukui, K. Formulation of the reaction coordinate. *J. Phys. Chem.* **74**, 4161−4163 (1970).
12. Fukui, K. The path of chemical reactions - the IRC approach. *Acc. Chem. Res.* **14**, 363−368 (1981).
13. Feller, D. The role of databases in support of computational chemistry calculations. *J. Comput. Chem.* **17**, 1571−1586 (1996).
14. Mayer, I. & Salvador, P. Overlap populations, bond orders and valences for 'fuzzy' atoms. *Chem. Phys. Lett.* **383**, 368−375 (2004).
15. Lu, T. & Chen, Q. Interaction region indicator: a simple real space function clearly revealing both chemical bonds and weak interactions. *Chemistry–Methods.* **1**, 231−239 (2021).
16. Lu, T. & Chen, F. Multiwfn: A multifunctional wavefunction analyzer. *J. Comput. Chem.* **33**, 580−592 (2012).

17. Fox, D. J. et al. Gaussian 16, Revision A.03, Gaussian: Wallingford, CT, USA, (2016).
