# Supplementary material for: Additive-controlled asymmetric iodocyclization enables enantioselective access to both α- and β-nucleosides
Source: Nat Commun. 2023 Jan 10;14:138. doi: 10.1038/s41467-022-35610-w (PMC9831021; doi:10.1038/s41467-022-35610-w)
Supplement: Supplementary file 3 — Description of Additional Supplementary Files [file 41467_2022_35610_MOESM3_ESM.docx]

**Description of Additional Supplementary Files**

**File Name: Supplementary Data 1
Description:** Cartesian coordinates together with the electronic energies for all the complexes calcd. in this study.
