# Supplementary material for: Additive-controlled asymmetric iodocyclization enables enantioselective access to both α- and β-nucleosides
Source: Nat Commun. 2023 Jan 10;14:138. doi: 10.1038/s41467-022-35610-w (PMC9831021; doi:10.1038/s41467-022-35610-w)
Supplement: Supplementary file 4 — Supplementary Data 1 [file 41467_2022_35610_MOESM4_ESM.pdf]

Supplementary Date 1

Cartesian coordinates together with the electronic energies for all the complexes calcd. in this study.

NaI

E = -173.667062

Na	0.00000000	0.00000000	-2.25933300
I	0.00000000	0.00000000	0.46891800

NIS

E = -371.230723

I	-1.49220600	-0.00000100	-0.00001800
C	1.29801400	-1.17839600	-0.00014400
C	1.29794000	1.17835000	0.00038300
C	2.76428300	-0.76591300	-0.00000600
C	2.76424700	0.76592300	-0.00011700
H	3.23950200	-1.20311600	0.88039400
H	3.23968000	-1.20335600	-0.88016600
H	3.23973700	1.20324100	0.88006700
H	3.23932600	1.20323800	-0.88051400
N	0.54400400	0.00001700	0.00053800
O	0.84834000	-2.29172100	-0.00013400
O	0.84837700	2.29174200	-0.00027700

NHS

E = -360.525879

C	1.17171900	-0.21460200	-0.00022000
C	-1.17167400	-0.21462200	0.00040100
C	0.76633000	1.25558200	0.00005500
C	-0.76633300	1.25557200	-0.00007300

H	1.20366200	1.73201000	0.87982200
H	1.20392300	1.73238100	-0.87935400
H	-1.20377300	1.73232100	0.87945500
H	-1.20377200	1.73204300	-0.87973900
N	-0.00001800	-0.96077700	0.00030600
O	2.28601100	-0.67018700	-0.00012200
O	-2.28602000	-0.67015600	-0.00029200
H	-0.00010100	-1.97215900	0.00001200

PPh_3S

E = -1434.168021

P	-0.00178100	0.00629100	0.84416400
S	0.00236300	0.00925000	2.80782900
C	0.21586200	-1.65325300	0.10722000
C	-0.43002200	-2.03059900	-1.07346400
C	1.08295200	-2.54565800	0.74285300
C	-0.19767500	-3.29095700	-1.61920000
H	-1.11703200	-1.34649000	-1.56325100
C	1.31238800	-3.80199100	0.19215500
H	1.55604500	-2.25470100	1.67637900
C	0.67408100	-4.17459000	-0.98899700
H	-0.70428200	-3.58342500	-2.53315200
H	1.98504500	-4.49313600	0.68926500
H	0.85021200	-5.15717400	-1.41497300
C	-1.54898100	0.64459100	0.10722700
C	-2.75218500	0.37456700	0.76532100
C	-1.55793100	1.35644500	-1.09543900
C	-3.95535200	0.80448100	0.21667800
H	-2.73135300	-0.15463400	1.71365700
C	-2.76613400	1.78580100	-1.63927200
H	-0.62560200	1.58409700	-1.60388200
C	-3.96357000	1.50846200	-0.98586800
H	-4.88728300	0.59488000	0.73144100

H	-2.76903700	2.34354200	-2.57009400
H	-4.90340200	1.84737000	-1.40971200
C	1.32746200	1.01681400	0.09879700
C	1.90572600	0.67915500	-1.12854300
C	1.74432200	2.16759400	0.77228200
C	2.88650700	1.49790500	-1.68260800
H	1.59629400	-0.22355000	-1.64759000
C	2.72486700	2.98128200	0.21410400
H	1.30888900	2.40346500	1.73904600
C	3.29446100	2.64849800	-1.01299500
H	3.33740600	1.23146900	-2.63297200
H	3.04950500	3.87256700	0.74095400
H	4.06284700	3.28209800	-1.44434300

1a

E = -685.097258

O	-3.26463900	-0.74475200	-0.91083800
N	0.30036800	-0.23652100	0.89369500
O	0.90702200	-2.29061000	0.04870500
O	1.48135200	1.95369900	-0.87595000
C	-2.91116200	1.75536300	0.45726300
H	-2.54841100	2.61877900	1.01861600
H	-3.81885400	1.37813100	0.93616300
H	-3.19756200	2.07910000	-0.54714300
C	-1.87022700	0.68140000	0.38826500
C	-0.65358300	0.76181400	0.95973400
H	-0.32973200	1.64224700	1.50477500
C	1.56674900	-0.03265500	1.52689500
C	2.70600300	0.07440000	0.85521900
C	2.90413600	0.04709700	-0.63579900
H	3.85179800	0.54777200	-0.85479100
C	-2.20699800	-0.53617700	-0.35038500
N	-1.18060700	-1.48535600	-0.38209500

C	0.08011300	-1.41432900	0.18396700
C	1.80981600	0.71195400	-1.47004300
H	-1.37128000	-2.33719600	-0.89541000
H	0.77783600	2.35645700	-1.39598300
H	3.60357200	0.18571600	1.45818700
H	1.52847500	0.01976600	2.61072100
H	3.00380000	-0.99617600	-0.96068800
H	2.19850200	0.85001300	-2.48871600
H	0.92456300	0.06863000	-1.55295500

C1

E = -2581.173829

P	-0.05839300	-0.98468600	0.22444400
O	0.57485800	-1.88942500	1.18439300
O	0.86119500	-0.03462600	-0.69038300
C	1.45785100	1.04507300	-0.04281900
O	-1.00630400	0.08364500	0.97452900
C	-1.54471100	1.11783600	0.22285700
C	-0.74215400	2.18242000	-0.11931900
C	0.69685800	2.15554600	0.25946100
C	-1.32734600	3.24144600	-0.89115100
C	-2.91950300	1.00881000	-0.11913500
C	-2.72420600	3.20139500	-1.16424000
C	-3.49211300	2.07832600	-0.75885300
C	1.33128100	3.22933300	0.96831500
C	2.84789500	0.93746700	0.21935200
C	3.46266000	2.01588900	0.80383600
C	2.73185500	3.16181900	1.21098400
H	-4.54975500	2.05216200	-1.00536600
C	-3.63575800	-0.27528800	0.14939600
C	-0.56629800	4.31635100	-1.42440200
C	-3.32157900	4.26116600	-1.89616000
C	3.37304600	4.23151500	1.88962500

C	0.61095700	4.34605500	1.47211900
C	3.58883700	-0.30987400	-0.13689400
H	4.53357600	1.98324800	0.98406200
C	-1.17035800	5.31702900	-2.14211100
C	-2.56618600	5.30094200	-2.36988000
C	2.65609700	5.30842800	2.33934500
C	1.25663200	5.35603300	2.13829100
C	-4.00238100	-1.10789900	-0.93621200
C	-3.86856100	-0.69650700	1.47118300
C	-4.47898000	-1.93401400	1.68975000
C	-4.60044700	-2.33864300	-0.66124100
C	-4.84840200	-2.77099900	0.64311100
H	-4.88534200	-2.97999600	-1.49171600
H	-4.66597500	-2.25811300	2.71108400
C	3.86025700	-0.60010500	-1.48463300
C	4.00907300	-1.18905800	0.88211500
C	4.59695700	-1.74568500	-1.79016200
C	5.05804000	-2.60923300	-0.80215300
C	4.74841900	-2.31545600	0.52526200
H	4.81840000	-1.97270800	-2.83126700
H	5.08214600	-2.99213400	1.30827400
O	-0.93642800	-1.61580700	-0.94521600
H	-1.69851000	-2.11881100	-0.61382200
C	5.85668000	-3.84835800	-1.15829500
C	5.10543600	-5.12416600	-0.76160900
C	7.25110700	-3.81721800	-0.52352500
H	5.98117400	-3.85350700	-2.24858300
H	4.11596400	-5.15741700	-1.22452500
H	5.66428000	-6.01373700	-1.06808800
H	4.96830200	-5.17035700	0.32357800
H	7.79709600	-2.91636200	-0.81553700
H	7.17983400	-3.82750000	0.56896100
H	7.83449100	-4.69126300	-0.82861300
C	3.68808000	-0.94645400	2.35068300

C	4.90779600	-0.37235300	3.08322100
C	3.19161900	-2.21195600	3.05856700
H	2.87756900	-0.21245300	2.40225200
H	5.24517200	0.56772500	2.63823400
H	4.67353300	-0.18823600	4.13623500
H	5.74307800	-1.07980600	3.03945200
H	2.35046600	-2.64902900	2.51886300
H	3.98695600	-2.95843100	3.15465300
H	2.86176900	-1.95887900	4.07086500
C	-5.49266800	-4.11811600	0.90604100
C	-4.61086800	-5.26284700	0.39438300
C	-6.89635000	-4.19492900	0.29568800
H	-5.58932000	-4.22515900	1.99350400
H	-3.61433700	-5.22383800	0.84210100
H	-5.05912800	-6.23169000	0.63320600
H	-4.49521700	-5.20724600	-0.69312500
H	-7.53274300	-3.38771100	0.66723200
H	-6.85113100	-4.11299800	-0.79519800
H	-7.37011300	-5.15043700	0.53912300
C	-3.78745600	-0.70601600	-2.39147000
C	-5.10293800	-0.18857300	-2.99104300
C	-3.23891800	-1.83976600	-3.26616600
H	-3.05262200	0.10350500	-2.41589100
H	-5.51629700	0.64114100	-2.41240300
H	-4.95088700	0.15050300	-4.02007600
H	-5.85196200	-0.98773500	-3.00230300
H	-2.30704500	-2.24639200	-2.86767400
H	-3.95908800	-2.65624400	-3.37636500
H	-3.03118700	-1.45616700	-4.26909600
C	-3.51312600	0.16262200	2.67444200
C	-2.62267400	-0.58568300	3.67240200
C	-4.79019200	0.67763600	3.35083500
H	-2.95518700	1.03508500	2.32381500
H	-1.72177500	-0.96865400	3.18725400

H	-2.31938800	0.08734400	4.47975000
H	-3.15577900	-1.42601700	4.12866000
H	-5.40558200	1.24645700	2.64801100
H	-5.39286900	-0.15415700	3.73017200
H	-4.54070800	1.32630200	4.19575200
H	4.44454800	4.16825800	2.05680800
H	3.15411000	6.11780800	2.86270700
H	0.68747400	6.19675800	2.52112700
H	-0.46363800	4.38759300	1.33379800
H	0.50573600	4.33485500	-1.26490300
H	-0.57008700	6.12625700	-2.54472600
H	-3.03079900	6.10329200	-2.93321100
H	-4.39052200	4.21981900	-2.08558600
C	3.39029300	0.29363600	-2.62197400
C	2.46751300	-0.46598300	-3.58200500
C	4.58196100	0.90629100	-3.36715100
H	2.81279300	1.12080700	-2.19835100
H	1.61189100	-0.88502400	-3.04682600
H	2.09460600	0.20577200	-4.36138300
H	3.00098000	-1.28512200	-4.07500200
H	5.22189100	1.47229400	-2.68433400
H	5.19420600	0.12823000	-3.83435800
H	4.23611100	1.58077800	-4.15639600

Int-SI

E = -3637.544568

O	5.90156900	3.47187000	-0.32963100
N	3.30044100	1.51447900	2.03579200
O	1.88924100	3.31338500	1.79049400
O	-0.12252100	0.20717700	4.26724700
C	6.88274100	1.02808400	0.85121800
H	7.01754800	0.09348800	1.39932700
H	7.64999000	1.73895400	1.16976100

H	7.05573500	0.83121100	-0.21256400
C	5.51572300	1.59341800	1.08535900
C	4.55162400	0.98575900	1.81013000
H	4.71131700	0.02088700	2.27972900
C	2.26457600	0.73067100	2.62906300
H	1.65145600	1.24883700	3.35725200
C	2.07803200	-0.54175300	2.27735100
H	2.66470800	-0.94537100	1.44981100
C	1.08987900	-1.47425500	2.91746400
H	0.26521600	-1.66722500	2.21303300
H	1.56571300	-2.45593400	3.04705100
C	5.19492800	2.87358600	0.46860300
N	3.96665700	3.40231200	0.85792700
C	2.96195200	2.78682200	1.58651000
C	0.54675200	-1.02966100	4.27394700
H	-0.10573000	-1.82661800	4.66046300
H	1.38700100	-0.93240800	4.97332000
I	4.85887400	-1.63241800	-0.74392800
H	3.72737500	4.31692900	0.49255400
H	-0.56405300	0.33713500	3.41122200
C	-4.26600600	-0.01943100	-0.22151200
C	-5.58407500	0.20318900	0.30114500
C	-5.95999500	1.52456400	0.67255500
C	-5.02053600	2.58571300	0.56679800
C	-3.72802900	2.36627100	0.16562200
C	-3.38849200	1.04048500	-0.22000200
H	-5.33485200	3.59127600	0.83193200
C	-3.81188800	-1.33714300	-0.74134100
C	-2.64261600	-1.89886200	-0.27466500
C	-4.52501400	-2.03561900	-1.77358300
C	-2.15492100	-3.16702100	-0.68662400
C	-4.08148600	-3.32921900	-2.16698200
C	-2.90719700	-3.87590600	-1.58748200
H	-2.59083600	-4.87291700	-1.88116000

O	-2.08509800	0.82277700	-0.66635000
O	-1.91146700	-1.25763900	0.71507100
P	-1.12901400	0.11696300	0.41683500
O	0.11357600	-0.29693400	-0.45614500
O	-0.87454600	0.85093000	1.66418500
C	-7.26786500	1.75972300	1.17094500
C	-6.51945400	-0.84784200	0.49539200
C	-5.64512800	-1.47451800	-2.44511800
C	-4.80062200	-4.04128800	-3.16246600
C	-7.77124700	-0.59033200	0.99419600
C	-8.15767400	0.72907600	1.32506100
C	-6.30891500	-2.18036200	-3.41598700
C	-5.89418500	-3.48498600	-3.77161800
C	-0.87567700	-3.68207400	-0.11182800
C	-0.87400900	-4.24759200	1.17347600
C	0.32156100	-3.56405800	-0.84606400
C	0.33130000	-4.71878100	1.69568200
C	1.50452300	-4.03981100	-0.27723900
C	1.52778700	-4.62451900	0.99041500
H	0.34273000	-5.16340200	2.68958900
H	2.42875800	-3.95243000	-0.84362700
C	-2.66708700	3.41495300	0.08289800
C	-2.28751100	3.89138100	-1.18910900
C	-1.96539100	3.80580300	1.23661900
C	-1.19240200	4.74362500	-1.28497200
C	-0.86192000	4.64952200	1.08558700
C	-0.44810200	5.10208500	-0.16012700
H	-0.88034300	5.10088500	-2.26272100
H	-0.26486400	4.90306500	1.95536900
H	-7.54247200	2.77530100	1.44152400
H	-9.15274400	0.91874200	1.71347300
H	-8.46935300	-1.40759100	1.14251400
H	-6.22911500	-1.86436200	0.25394200
H	-5.96706700	-0.47147000	-2.18988700

H	-7.15748000	-1.73055900	-3.92081900
H	-6.43382300	-4.03385900	-4.53616500
H	-4.45373900	-5.03245000	-3.44079500
C	5.64599300	0.66723900	-2.56656100
C	3.58249600	1.00293800	-1.50036600
C	5.16624000	2.03120300	-3.02573000
C	3.77480000	2.20290800	-2.41033100
H	5.87160300	2.76876200	-2.63501200
H	5.18441000	2.06462900	-4.11603000
N	4.66378000	0.16091900	-1.68739200
O	2.65564800	0.78711200	-0.74617100
O	6.66005800	0.09133700	-2.84591000
H	3.67666200	3.11525300	-1.82001100
H	2.96426100	2.19060100	-3.14434100
C	-2.13699300	-4.35526300	2.01292800
C	-2.02592500	-3.51011800	3.28654700
C	-2.45752000	-5.81698900	2.34544100
H	-2.97349500	-3.96219200	1.42740200
H	-1.85552600	-2.45713000	3.04368500
H	-2.94476100	-3.58185200	3.87620800
H	-1.19764800	-3.85837200	3.91381500
H	-2.55061900	-6.41575900	1.43509700
H	-1.67163500	-6.26323900	2.96326400
H	-3.39722300	-5.88472100	2.90130900
C	2.79711500	-5.20512500	1.58561900
C	4.02289500	-4.30790200	1.39659200
C	3.06172000	-6.60194100	1.00996700
H	2.62823500	-5.30853100	2.66519500
H	3.82243600	-3.28603900	1.73654900
H	4.87655600	-4.69944900	1.95658800
H	4.31482700	-4.28358500	0.33814300
H	2.21130800	-7.26462900	1.18896900
H	3.21682600	-6.53998700	-0.07237300
H	3.95324600	-7.05016200	1.45922200

C	0.32844200	-2.97442000	-2.24976800
C	0.15336600	-4.08396900	-3.29629700
C	1.59097300	-2.17101600	-2.56849400
H	-0.52299900	-2.28995500	-2.32606900
H	-0.78232400	-4.62961300	-3.15824500
H	0.15703300	-3.66293500	-4.30641300
H	0.97670500	-4.80253900	-3.22258100
H	1.81756400	-1.45622600	-1.77498400
H	2.45998400	-2.82363200	-2.70438500
H	1.44934500	-1.62139500	-3.50414000
C	-2.35766700	3.32789600	2.62644600
C	-3.40009200	4.27278300	3.24112800
C	-1.16085600	3.19655700	3.57543100
H	-2.80778000	2.33566700	2.51984800
H	-4.31098300	4.32880900	2.64005700
H	-3.67336900	3.93700700	4.24633900
H	-2.98844000	5.28469000	3.32043600
H	-0.31040200	2.72647500	3.07834800
H	-0.84327800	4.17731400	3.94753000
H	-1.43438500	2.58853600	4.44215200
C	0.84147200	5.88354000	-0.30646700
C	0.61486700	7.28786600	-0.87147700
C	1.82180400	5.08757800	-1.17723000
H	1.27939500	5.97106800	0.69523200
H	-0.06146500	7.86218100	-0.23344700
H	1.55990000	7.83401300	-0.95357800
H	0.17004000	7.23585400	-1.87085300
H	1.90071600	4.06362300	-0.79753300
H	1.47072700	5.03263700	-2.21351100
H	2.81409400	5.55787200	-1.19540000
C	-3.07256000	3.50046200	-2.43263500
C	-2.23033000	3.47746900	-3.70917500
C	-4.28097100	4.43115900	-2.60105800
H	-3.45437300	2.48424800	-2.28635100

H	-1.32407800	2.88062500	-3.57273400
H	-2.80947700	3.04306800	-4.52882200
H	-1.93645300	4.48551200	-4.01882100
H	-4.93189100	4.39278500	-1.72311800
H	-3.94388800	5.46536300	-2.72713000
H	-4.87031600	4.15132900	-3.47996400
H	0.92768800	0.22823700	-0.31759200

Int-I

E = -3811.297218

O	-5.20722200	-3.64776300	2.34375000
N	-2.52456300	-0.71833300	2.99145600
O	-1.24411400	-1.71723400	1.37995600
O	0.95552100	1.93311700	2.86290300
C	-6.02464900	-1.59611500	4.19166900
H	-6.12032100	-0.71374700	4.82679400
H	-6.15349100	-2.49220500	4.80460000
H	-6.83641400	-1.59595700	3.45910700
C	-4.69904800	-1.62221700	3.49756800
C	-3.74637300	-0.68222400	3.64579700
H	-3.86991900	0.17457500	4.29849800
C	-1.64468700	0.41685300	2.98567300
H	-0.59687700	0.20439700	3.17097000
C	-2.11476700	1.63260500	2.72430500
H	-3.16727800	1.72779800	2.46215700
C	-1.28943600	2.88591000	2.71534600
H	-1.06151500	3.14330600	1.66937800
H	-1.88866500	3.71476000	3.11401100
C	-4.43178700	-2.75241800	2.60624400
N	-3.13998400	-2.74560600	2.06176300
C	-2.24884000	-1.72517200	2.11206800
C	0.02780400	2.79944300	3.47819400
H	0.45177500	3.80749600	3.58633200

H	-0.14769200	2.40098400	4.48332900
I	-3.64526500	2.98614000	-0.47141400
H	-2.90805500	-3.47952800	1.39839100
H	1.24233500	2.33806800	2.02942900
C	4.28528900	-1.42388600	-0.03153700
C	5.31064700	-2.29868500	0.45670100
C	5.00261700	-3.67553200	0.64466300
C	3.68543200	-4.14805700	0.40090000
C	2.66763000	-3.30136500	0.03808300
C	3.01710600	-1.94011500	-0.17210100
H	3.47571000	-5.20492200	0.54320200
C	4.51604200	-0.00331700	-0.41175900
C	3.68298100	0.98753400	0.06459800
C	5.50999900	0.36692700	-1.38188800
C	3.74910100	2.34260900	-0.35044700
C	5.66898800	1.74030100	-1.72040800
C	4.77356300	2.70242100	-1.18743800
H	4.86943100	3.74003400	-1.49715400
O	1.99240000	-1.08632000	-0.55973800
O	2.72302400	0.69218200	1.03126100
P	1.41849300	-0.13851500	0.60922100
O	0.26028900	0.62762500	0.09828900
O	1.17471100	-1.04215600	1.86545300
C	6.01650400	-4.55512600	1.10710100
C	6.61238900	-1.84096700	0.79099100
C	6.32175500	-0.58603500	-2.05619400
C	6.67208000	2.12286600	-2.64908500
C	7.56547100	-2.71348000	1.25185600
C	7.27105500	-4.08894600	1.40106900
C	7.27188000	-0.18730900	-2.96160300
C	7.46515900	1.18309300	-3.25188400
C	2.68853000	3.31041400	0.06618500
C	2.83141800	4.04448200	1.25849100
C	1.54835700	3.47179800	-0.74754300

C	1.84594800	4.97475600	1.59177900
C	0.58081600	4.40400700	-0.35968100
C	0.71546800	5.17093100	0.79652100
H	1.95452200	5.56425300	2.50068000
H	-0.29860500	4.52390700	-0.98453000
C	1.24676700	-3.73787700	-0.11731800
C	0.58358700	-3.54569100	-1.35424300
C	0.55820000	-4.32354900	0.96364100
C	-0.75779200	-3.90950000	-1.46940400
C	-0.76036800	-4.74916800	0.76713600
C	-1.44386000	-4.53386200	-0.42409200
H	-1.27015400	-3.73080200	-2.41302400
H	-1.27852900	-5.24941900	1.58494300
H	5.77070000	-5.60545200	1.23561200
H	8.03676700	-4.76846400	1.76002400
H	8.55292600	-2.34485600	1.50921100
H	6.84274900	-0.78630700	0.68470900
H	6.17758300	-1.64139000	-1.85919800
H	7.87551000	-0.93341000	-3.46768300
H	8.22499700	1.48283300	-3.96576500
H	6.78298300	3.17766100	-2.88392200
Na	-1.62215900	-0.41476600	-0.61843300
C	-6.05679200	1.50557900	-1.55100000
C	-4.67885400	0.16340900	-0.22109800
C	-6.60736900	0.09754300	-1.67086800
C	-5.81163600	-0.73755400	-0.66437400
H	-7.68589900	0.10761400	-1.51025300
H	-6.40080800	-0.22386000	-2.69620800
N	-4.93615400	1.42930300	-0.68829000
O	-3.71940100	-0.14683900	0.46860000
O	-6.44254800	2.51558200	-2.06464700
H	-6.38922800	-1.02049600	0.22169600
H	-5.37431100	-1.63638300	-1.10080900
C	4.04576100	3.86425800	2.15509900

C	3.67512300	3.76393700	3.63738900
C	5.05156200	4.99476000	1.90682700
H	4.52716400	2.92085800	1.87732300
H	2.94386000	2.96696300	3.79634200
H	4.56789500	3.54396700	4.22982100
H	3.25994700	4.70457300	4.01468600
H	5.36360900	5.02120700	0.85866000
H	4.60403200	5.96445000	2.14982400
H	5.94212700	4.86415300	2.52868600
C	-0.29067800	6.23664700	1.19646700
C	-1.68813900	6.02876900	0.61321100
C	0.24308000	7.62296500	0.80765400
H	-0.37583200	6.20275000	2.29202000
H	-2.07857600	5.03914300	0.87483600
H	-2.38065300	6.77537900	1.01023900
H	-1.68711000	6.13023900	-0.47701100
H	1.22223400	7.80880500	1.25650200
H	0.35392200	7.68874300	-0.27943900
H	-0.44289000	8.41098200	1.13221800
C	1.36946600	2.69170700	-2.04440800
C	2.05538700	3.42126300	-3.20921300
C	-0.09722900	2.43327900	-2.40181400
H	1.85532500	1.71629100	-1.91782600
H	3.12952800	3.53569900	-3.04869200
H	1.90558900	2.86622800	-4.13975000
H	1.61968800	4.41824900	-3.33396500
H	-0.66027700	2.09016400	-1.53240100
H	-0.58354700	3.33341900	-2.79501000
H	-0.17335600	1.66565600	-3.17698500
C	1.17290900	-4.52727600	2.34318300
C	1.41829700	-6.01861500	2.60904900
C	0.30846700	-3.93261900	3.46314300
H	2.13390000	-4.00797500	2.37495800
H	2.03773700	-6.47071600	1.82943500

H	1.91571100	-6.16295700	3.57278000
H	0.47058400	-6.56694000	2.63273600
H	0.20043900	-2.85148700	3.35058600
H	-0.69118900	-4.37853800	3.48805200
H	0.77763900	-4.11847200	4.43376500
C	-2.87970100	-4.99772200	-0.59537500
C	-2.94423500	-6.22571600	-1.51171900
C	-3.79026300	-3.88359800	-1.12384300
H	-3.25337200	-5.30660800	0.39324300
H	-2.32804900	-7.04002600	-1.12184900
H	-3.97392800	-6.58181500	-1.60816400
H	-2.57728400	-5.96963200	-2.51070900
H	-3.69691400	-2.96495100	-0.53215400
H	-3.54146000	-3.61149900	-2.15388500
H	-4.83612700	-4.20497000	-1.09397500
C	1.30446600	-3.06053500	-2.60579500
C	0.63010500	-1.84796300	-3.25252400
C	1.41683100	-4.21788100	-3.60919100
H	2.32441600	-2.77498900	-2.33732600
H	0.55732500	-1.01135700	-2.55029300
H	1.21561400	-1.50973800	-4.11344000
H	-0.37893700	-2.07460100	-3.60720100
H	1.91993900	-5.08035300	-3.16222100
H	0.42572400	-4.53933100	-3.94435500
H	1.98457500	-3.90313500	-4.48970700
I	-3.07309500	-0.49429600	-3.19507600
H	0.22856000	-1.37480900	1.80788000

Int-I’

E = -3811.248365

O	3.67235900	6.16005700	-1.09540000
N	2.86593600	2.44585300	0.22696700
O	1.16631100	3.36514400	1.45421400

O	1.10938800	-0.67111000	2.62174600
C	5.13869100	4.03063300	-2.37511700
H	5.64132500	3.09531700	-2.62693700
H	5.88636900	4.77256700	-2.08418100
H	4.62896200	4.41136900	-3.26473100
C	4.14835700	3.82852200	-1.27344300
C	3.81300700	2.62708900	-0.77477200
H	4.27048800	1.71340500	-1.13371800
C	2.51400900	1.15506200	0.72583700
H	2.37328400	1.11841600	1.80177500
C	2.31719800	0.09389100	-0.06734200
H	2.34081500	0.23874700	-1.14819400
C	2.01946000	-1.29841900	0.42489600
H	1.04765600	-1.61036800	0.01609500
H	2.73440500	-1.99581300	-0.02859400
C	3.47830800	5.01577600	-0.74615000
N	2.54480100	4.73717400	0.26793900
C	2.13346100	3.51118600	0.69626700
C	2.03292100	-1.50209400	1.93541100
H	1.81375000	-2.55934000	2.13784400
H	3.02648900	-1.28431300	2.34612400
I	5.48232700	-0.42169000	0.63555300
H	1.98258500	5.52225400	0.57530100
H	0.41891700	-0.42371500	1.99585400
C	-4.75331200	-0.91293400	0.23264900
C	-5.91164600	-0.93585300	1.07855800
C	-6.35139800	0.27854300	1.67730300
C	-5.58597800	1.46026700	1.51285700
C	-4.41623400	1.47311400	0.79290000
C	-4.05377700	0.27192100	0.12572800
H	-5.91856400	2.37360900	1.99875500
C	-4.23756500	-2.13884800	-0.43760800
C	-2.93108900	-2.51671000	-0.21908300
C	-5.02647200	-2.95158500	-1.31513400

C	-2.32597400	-3.69031100	-0.73151100
C	-4.46091000	-4.15530400	-1.82297000
C	-3.12506700	-4.50653600	-1.49430100
H	-2.71794000	-5.43761300	-1.87812000
O	-2.93508600	0.32981900	-0.70415800
O	-2.12432900	-1.68298400	0.56654900
P	-1.58735700	-0.41400100	-0.25840700
O	-0.62178700	-0.61628100	-1.32951000
O	-1.01506300	0.47327600	0.96683900
C	-7.51739400	0.27710300	2.48787600
C	-6.62555800	-2.12955600	1.37285800
C	-6.33580800	-2.58661700	-1.72698900
C	-5.23881000	-4.97788900	-2.67991700
C	-7.73919400	-2.10122800	2.17254500
C	-8.20265400	-0.88420500	2.72599100
C	-7.05528900	-3.39604400	-2.56839300
C	-6.50811300	-4.61160500	-3.04237600
C	-0.89756900	-4.02223800	-0.43540100
C	-0.52770100	-4.41214700	0.86517400
C	0.06574000	-3.99134000	-1.46714900
C	0.77643200	-4.85875500	1.08870900
C	1.35686400	-4.44302100	-1.18849800
C	1.72814700	-4.90664500	0.07388800
H	1.05785200	-5.19314600	2.08563700
H	2.09541600	-4.43276100	-1.98650500
C	-3.51525300	2.66985300	0.78624600
C	-3.42067200	3.49132900	-0.35584400
C	-2.74910700	2.96128500	1.93961500
C	-2.56977200	4.60256700	-0.32240900
C	-1.93169200	4.09442100	1.93119900
C	-1.82475600	4.92686100	0.81358600
H	-2.45961800	5.20387900	-1.21814700
H	-1.31498000	4.31571700	2.79857500
H	-7.84424300	1.21582000	2.92593900

H	-9.08968600	-0.87855300	3.35033200
H	-8.26764400	-3.02374500	2.38857200
H	-6.27689700	-3.07068900	0.96414700
H	-6.75867100	-1.65104100	-1.37750700
H	-8.05087000	-3.09822500	-2.88001300
H	-7.09144900	-5.24315600	-3.70386800
H	-4.79804700	-5.89747800	-3.05377600
Na	-0.50365600	2.61339500	0.09750400
C	8.48891200	-0.43238500	0.21184500
C	7.70536700	-2.39767400	1.23230400
C	9.72393000	-1.29494600	0.43977400
C	9.21173700	-2.58031800	1.09657700
H	10.20865600	-1.45824900	-0.52470100
H	10.42010200	-0.73254100	1.06527000
N	7.39262700	-1.14466100	0.70730900
O	6.91369500	-3.17428700	1.69991200
O	8.43723600	0.65569400	-0.29496900
H	9.39275500	-3.47729000	0.50078600
H	9.62447300	-2.75912600	2.09143100
C	-1.49718300	-4.39929200	2.03639500
C	-0.95945300	-3.54358100	3.18925600
C	-1.81166900	-5.82534200	2.50337700
H	-2.43963200	-3.95254300	1.70801900
H	-0.68015300	-2.54086100	2.85184200
H	-1.71341300	-3.44937600	3.97688500
H	-0.07029700	-3.99797900	3.63879700
H	-2.23403600	-6.41942400	1.68794600
H	-0.90533600	-6.32971300	2.85313500
H	-2.52968500	-5.81078100	3.32894100
C	3.12341200	-5.43933800	0.34430000
C	4.17157600	-4.32138400	0.34381600
C	3.50745900	-6.54132000	-0.64880900
H	3.10903300	-5.88229300	1.34809700
H	3.96246000	-3.57067600	1.11214800

H	5.17043400	-4.71650000	0.54622700
H	4.19748300	-3.81532800	-0.62917700
H	2.76161200	-7.34036400	-0.66342700
H	3.59359100	-6.14188100	-1.66437700
H	4.47554600	-6.97415000	-0.38090000
C	-0.24841800	-3.48187900	-2.86713700
C	-0.40999100	-4.64714300	-3.85201000
C	0.81910700	-2.50698100	-3.37929200
H	-1.19176400	-2.92900700	-2.82197800
H	-1.21154900	-5.32839400	-3.55390200
H	-0.63585300	-4.27172400	-4.85444200
H	0.51661300	-5.22860700	-3.90838000
H	0.98477200	-1.69885900	-2.66514600
H	1.76801100	-3.01644700	-3.57654700
H	0.48708500	-2.05776500	-4.31925900
C	-2.75709500	2.07521000	3.18282900
C	-3.72463300	2.62165100	4.24197400
C	-1.36365200	1.90667100	3.81656600
H	-3.13865100	1.08225900	2.89620000
H	-4.75045000	2.66245700	3.87122900
H	-3.70619600	1.99340100	5.13694900
H	-3.42480900	3.63503900	4.52708100
H	-0.56465100	1.71067300	3.09340300
H	-1.07291400	2.81030200	4.35932100
H	-1.37946100	1.08627000	4.53913400
C	-0.89983300	6.12873300	0.87693300
C	-1.62445300	7.30765600	1.54094900
C	-0.32759400	6.53631100	-0.48277800
H	-0.07012500	5.83025800	1.53362100
H	-1.99007800	7.04225400	2.53698000
H	-0.95431800	8.16688800	1.63537000
H	-2.48350900	7.61021500	0.93327000
H	0.09237900	5.68482000	-1.03050600
H	-1.09961000	6.98752300	-1.11479000

H	0.45632100	7.28978900	-0.35365800
C	-4.27053300	3.20797200	-1.58435300
C	-3.64356100	3.69329600	-2.89218700
C	-5.66379300	3.82427900	-1.38868500
H	-4.38843400	2.12208700	-1.66281000
H	-2.61062200	3.34897800	-2.99998300
H	-4.22717600	3.31453800	-3.73546500
H	-3.65298800	4.78632300	-2.95991700
H	-6.15706900	3.43312900	-0.49403800
H	-5.58275600	4.91148100	-1.28638700
H	-6.29916200	3.60997400	-2.25285100
I	0.48664700	2.95275300	-2.55987900
H	-1.63757500	0.55479400	1.71141100

Int-II

E = -3811.311484

O	-4.95208400	-2.98814500	5.18655000
N	-4.59077300	-0.90458300	1.76455400
O	-6.66946200	-1.69186400	1.17737700
O	-1.28010000	0.64066200	1.06761100
C	-2.41906100	-1.69636900	4.78073500
H	-1.56103900	-1.13197300	4.40987500
H	-2.11990200	-2.73342000	4.95860300
H	-2.72573400	-1.28630100	5.74686900
C	-3.55556600	-1.63785400	3.80892500
C	-3.50881700	-0.97130500	2.64339100
H	-2.61741100	-0.44533700	2.30406400
C	-4.45759700	-0.27191800	0.50601400
H	-5.40971600	-0.34485800	-0.00886800
C	-3.91562300	1.15916000	0.60663400
H	-3.50923700	1.29653300	1.60632600
C	-2.89948400	1.68210500	-0.40984100
H	-2.84840300	1.00026700	-1.26193900

H	-3.18000700	2.66390400	-0.79369300
C	-4.78322300	-2.35889100	4.16401200
N	-5.79170600	-2.28951000	3.19491100
C	-5.76537200	-1.64302200	1.98143500
C	-1.53488900	1.78708000	0.28255100
H	-0.73965200	1.97500100	-0.45525600
H	-1.54298700	2.65500800	0.95363600
I	-5.71430200	2.44322200	0.69743100
H	-6.64104200	-2.80577300	3.39020900
H	-0.67148800	0.05311900	0.57120800
C	4.27407800	-0.84308700	0.93995000
C	4.85148500	-1.54358600	2.05130000
C	4.66739800	-2.95167400	2.15116900
C	3.83152200	-3.61597800	1.21874000
C	3.18039800	-2.93334000	0.22201300
C	3.45834400	-1.54620900	0.07680600
H	3.66060100	-4.68324600	1.33575600
C	4.46241400	0.61857200	0.73367300
C	3.35357400	1.42682300	0.58142400
C	5.75820600	1.23016300	0.66777600
C	3.41763000	2.84047500	0.45913900
C	5.84541900	2.64817900	0.59368600
C	4.65846900	3.42273600	0.51953100
H	4.74009700	4.50550300	0.48434100
O	2.91053300	-0.89849300	-1.00949000
O	2.09301500	0.85347500	0.59885700
P	1.63505500	0.09519300	-0.76122400
O	1.54015500	0.98857400	-1.95262500
O	0.43670400	-0.74811800	-0.38574300
C	5.26620400	-3.66271200	3.22469900
C	5.57892800	-0.88748900	3.08177300
C	6.96340200	0.47749900	0.64395700
C	7.12265000	3.26679300	0.55996500
C	6.13184900	-1.59935200	4.11543300

C	5.99006600	-3.00542600	4.18350200
C	8.18286700	1.10364200	0.59354900
C	8.26820300	2.51532600	0.56494200
C	2.17067600	3.64711200	0.28713800
C	1.33933500	3.89302300	1.39533900
C	1.84891500	4.17834200	-0.97653100
C	0.23540500	4.72945600	1.23188300
C	0.71667400	4.98753600	-1.09433200
C	-0.09014300	5.29338300	-0.00107800
H	-0.39441900	4.95331600	2.09236700
H	0.46359200	5.38787900	-2.07004300
C	2.13837700	-3.59846700	-0.61758900
C	2.32287900	-3.77267600	-2.00410900
C	0.94132600	-4.04359500	-0.00206700
C	1.32402400	-4.41743800	-2.74724800
C	-0.02256000	-4.68755400	-0.78691000
C	0.15039200	-4.89292600	-2.16105900
H	1.46809700	-4.55688500	-3.81626900
H	-0.93982200	-5.02844100	-0.31135900
H	5.12279500	-4.73842900	3.27636000
H	6.43947500	-3.55484800	5.00392600
H	6.67828200	-1.07716400	4.89390600
H	5.68588500	0.19041800	3.04891300
H	6.90968400	-0.60554000	0.65572600
H	9.09101100	0.51022600	0.56903300
H	9.23970400	2.99713900	0.53166500
H	7.16974200	4.35122200	0.51415500
Na	-0.09799200	-2.21362200	-1.87989800
C	-1.70372000	2.15315600	-3.31431500
C	-1.40961400	-0.09402900	-3.72252500
C	-2.94553000	1.72941700	-4.09005600
C	-2.73150200	0.24349200	-4.38863800
H	-3.82090800	1.92396800	-3.46494500
H	-3.03300100	2.35307200	-4.98164700

N	-0.88461400	1.03320900	-3.16986700
O	-0.90184700	-1.20928600	-3.67912100
O	-1.45836800	3.24655400	-2.87026100
H	-3.50141000	-0.40820300	-3.96836200
H	-2.64943700	0.01645900	-5.45423400
C	1.63605300	3.31469300	2.76991300
C	0.43202700	2.55998000	3.34269900
C	2.11185600	4.41619000	3.72417500
H	2.45150100	2.59212200	2.66960600
H	-0.40944700	3.23718200	3.52779300
H	0.09720000	1.77452600	2.65867700
H	0.69759400	2.10110300	4.30066900
H	3.00044600	4.91818900	3.33006300
H	1.33174000	5.17237800	3.86143900
H	2.35651700	3.99949600	4.70614700
C	-1.31933100	6.17778200	-0.12107200
C	-2.58124600	5.31752100	-0.26956300
C	-1.23724100	7.18232900	-1.27090000
H	-1.40050300	6.74085300	0.81819900
H	-2.70210800	4.63775700	0.58057500
H	-3.47847200	5.94191000	-0.33068900
H	-2.50681200	4.72174800	-1.18588900
H	-0.30651300	7.75547200	-1.23902300
H	-1.29343200	6.67282800	-2.23830900
H	-2.07638900	7.88180900	-1.22045000
C	2.71553500	3.92774300	-2.20303100
C	3.69868900	5.08669300	-2.41872600
C	1.89318800	3.71494600	-3.47741300
H	3.28383100	3.00908400	-2.02881500
H	4.37176900	5.21768800	-1.56836400
H	4.30832300	4.91142900	-3.31087700
H	3.15005900	6.02432300	-2.56004800
H	1.09759600	2.99041700	-3.30801400
H	1.44033000	4.64847800	-3.82783000

H	2.54321800	3.34485800	-4.27587200
C	0.64633700	-3.84052600	1.47914200
C	0.70179100	-5.18094500	2.22336800
C	-0.70087700	-3.14890700	1.71891500
H	1.41700400	-3.18672700	1.89287700
H	1.66789400	-5.67678100	2.09423500
H	0.53433200	-5.02895400	3.29338300
H	-0.07368600	-5.86072400	1.85422700
H	-0.73628800	-2.17936200	1.21523600
H	-1.54627000	-3.75188500	1.36977200
H	-0.83951000	-2.98455500	2.79183200
C	-0.92399800	-5.56406700	-2.99528700
C	-2.19868500	-4.71085200	-3.03448800
C	-1.22820700	-6.97743100	-2.48863000
H	-0.53685100	-5.64272300	-4.01845800
H	-1.99885000	-3.70554100	-3.42564100
H	-2.95537700	-5.17276300	-3.67520500
H	-2.63040400	-4.61688300	-2.03123400
H	-0.32403600	-7.59079900	-2.46161600
H	-1.64823000	-6.94811100	-1.47819300
H	-1.95927500	-7.46668700	-3.13810900
C	3.57626700	-3.30755600	-2.72723900
C	3.24274200	-2.31602000	-3.84836600
C	4.36098900	-4.51094900	-3.26471800
H	4.21527000	-2.79117700	-2.00712400
H	2.70671100	-1.44581400	-3.45923800
H	4.16360400	-1.95927800	-4.31824700
H	2.63413700	-2.78604000	-4.62830100
H	4.61777100	-5.20534100	-2.45985400
H	3.77989300	-5.05959500	-4.01315200
H	5.28752300	-4.17650700	-3.73961800
H	0.04654700	1.05516400	-2.68525500
I	-3.30596500	-1.68924300	-0.88135900

TS-I

E = -3811.295848

O	1.90082000	2.13853500	5.74198900
N	3.52781900	0.56870300	2.40935200
O	4.64398800	2.57590200	2.14678400
O	1.64618300	-0.37115700	0.59831400
C	1.35590200	-0.61017600	5.29843400
H	1.36760400	-1.66033300	5.00740800
H	0.32522600	-0.24437800	5.26789400
H	1.70350600	-0.50873100	6.32913700
C	2.21287700	0.20938300	4.38929700
C	2.75854200	-0.25399800	3.26054600
H	2.57595200	-1.25464500	2.89268100
C	3.87154100	0.21995300	1.18077900
H	4.21517000	1.04919600	0.55970000
C	4.02293400	-1.14565700	0.64697000
H	3.68623400	-1.94290900	1.30957100
C	3.40096300	-1.24068400	-0.73816100
H	3.68025900	-0.36501200	-1.33172400
H	3.72642700	-2.13384200	-1.27394400
C	2.39256900	1.62912900	4.76358500
N	3.18935000	2.37597300	3.89193700
C	3.84992700	1.94298300	2.77722600
C	1.89345500	-1.26435300	-0.47631200
H	1.35127400	-0.97202600	-1.38058700
H	1.57531700	-2.27575400	-0.19338300
I	6.21276800	-1.31524500	0.61786700
H	3.35863700	3.34314600	4.14670500
H	0.91761600	0.24532100	0.32578100
C	-4.19493200	-0.49793900	0.68878400
C	-4.98049000	-0.33506100	1.87789600
C	-5.20036100	0.97801200	2.38199900
C	-4.56192300	2.08203300	1.76231900

C	-3.72579500	1.92215000	0.68576200
C	-3.59815100	0.61655300	0.13680000
H	-4.70337900	3.07385800	2.18424500
C	-3.91264900	-1.83589900	0.10206200
C	-2.59801800	-2.21952800	-0.07520700
C	-4.94401800	-2.75383800	-0.28103900
C	-2.21225500	-3.52689800	-0.47282900
C	-4.58028800	-4.06391500	-0.69943300
C	-3.20942100	-4.42903000	-0.74732100
H	-2.94612000	-5.44960900	-1.01214600
O	-2.87069300	0.47506400	-1.02393500
O	-1.58751300	-1.31919300	0.20207300
P	-1.36713300	-0.15598300	-0.92969400
O	-1.02232600	-0.72497000	-2.26354100
O	-0.46150500	0.86333100	-0.27917500
C	-6.00322700	1.15846400	3.53909700
C	-5.52593100	-1.43135800	2.60079500
C	-6.31935000	-2.39710900	-0.28903200
C	-5.59682700	-4.98430200	-1.06589100
C	-6.28552400	-1.22588500	3.72439500
C	-6.54221100	0.08338900	4.19465100
C	-7.27775700	-3.30437400	-0.66280000
C	-6.91690600	-4.61752400	-1.04627900
C	-0.77422100	-3.93887100	-0.49203600
C	-0.12128000	-4.20344900	0.72841900
C	-0.09831900	-4.13835700	-1.70687300
C	1.15556400	-4.75889300	0.70374200
C	1.19377600	-4.68059000	-1.68174300
C	1.82676000	-5.02923200	-0.49118000
H	1.63996600	-5.00681800	1.64735600
H	1.69929400	-4.84403700	-2.62810800
C	-2.91396600	3.06132600	0.16090800
C	-3.16392600	3.59376900	-1.12204100
C	-1.88889900	3.60826700	0.96349900

C	-2.40494900	4.68041900	-1.56063000
C	-1.16374000	4.70885400	0.48281200
C	-1.40414700	5.26408000	-0.77606800
H	-2.59792000	5.09065300	-2.54971900
H	-0.37710200	5.12304600	1.10535800
H	-6.16780200	2.16959300	3.90105600
H	-7.15077800	0.23010500	5.08076000
H	-6.68930000	-2.07768200	4.26192200
H	-5.32943000	-2.44088600	2.25890700
H	-6.60290700	-1.38924900	-0.00537800
H	-8.32238900	-3.01096000	-0.67073400
H	-7.68619300	-5.32597400	-1.33512500
H	-5.30265600	-5.98349800	-1.37485000
Na	-0.26555100	2.83339900	-1.21754000
C	2.25776900	0.34709700	-3.86031700
C	0.70268800	2.04010000	-3.81392500
C	2.97113300	1.55722100	-4.44567600
C	1.89680800	2.64436100	-4.52657900
H	3.77163200	1.83104800	-3.75308000
H	3.41672600	1.28555600	-5.40419000
N	0.94824700	0.71451700	-3.58449400
O	-0.31629100	2.62642900	-3.47442000
O	2.73088600	-0.74411400	-3.63895400
H	2.17107000	3.57008500	-4.01730700
H	1.59891000	2.88262200	-5.55169900
C	-0.74820300	-3.87336800	2.07222800
C	0.07808500	-2.79475200	2.78462700
C	-0.91869900	-5.11689800	2.94992300
H	-1.74356200	-3.45584100	1.90067200
H	1.06104900	-3.19766800	3.06788500
H	0.22875400	-1.92340200	2.13718800
H	-0.42912100	-2.47240600	3.69982300
H	-1.53432500	-5.86725300	2.44655000
H	0.04805700	-5.57549800	3.18208000

H	-1.40071900	-4.85493600	3.89670800
C	3.19634100	-5.69122700	-0.43303700
C	4.21554300	-4.80225700	0.29198100
C	3.74312400	-6.09671400	-1.80001500
H	3.07448800	-6.60463800	0.16542400
H	3.85822900	-4.49503300	1.28011400
H	5.16727100	-5.32523100	0.42522900
H	4.41436000	-3.90150900	-0.29751600
H	3.04955400	-6.75208700	-2.33333000
H	3.92744300	-5.21567800	-2.42435700
H	4.69271400	-6.62590300	-1.68442600
C	-0.73647500	-3.79192000	-3.04288100
C	-1.28533500	-5.04771100	-3.73171900
C	0.23895300	-3.06276700	-3.97232800
H	-1.56384400	-3.10509300	-2.84522200
H	-2.05537200	-5.53765400	-3.12996400
H	-1.72415400	-4.78975400	-4.70042300
H	-0.48133800	-5.77159400	-3.90545300
H	0.76855000	-2.26293000	-3.45156800
H	0.98660000	-3.74470100	-4.39263000
H	-0.31035800	-2.62241900	-4.80980400
C	-1.52600600	3.04978200	2.33434600
C	-1.97196900	4.01466000	3.44069500
C	-0.02902700	2.74141500	2.47263800
H	-2.05881800	2.10490600	2.46788400
H	-3.04563600	4.21581700	3.39154600
H	-1.74417600	3.59977300	4.42707500
H	-1.44938200	4.97282500	3.34860800
H	0.29490400	2.01961800	1.71825400
H	0.58965900	3.63958900	2.37157300
H	0.15148400	2.32316400	3.46973000
C	-0.59889000	6.42334400	-1.34349100
C	0.23340300	5.95869800	-2.54982100
C	0.30910300	7.09778200	-0.31734400

H	-1.32793600	7.16379600	-1.70024800
H	-0.37596500	5.44294800	-3.29710500
H	0.71936500	6.81501600	-3.02745800
H	1.02011500	5.26959900	-2.21786000
H	-0.24752500	7.43329800	0.56273600
H	1.09993300	6.41183200	0.00657900
H	0.79353600	7.96896200	-0.76570900
C	-4.25426800	3.04778100	-2.02970100
C	-3.68982600	2.58619100	-3.37733400
C	-5.36295000	4.09118800	-2.21619600
H	-4.70113900	2.17687600	-1.54312000
H	-2.91561600	1.82808300	-3.24004300
H	-4.49024100	2.16004900	-3.98960700
H	-3.25004200	3.42105100	-3.93279800
H	-5.78138100	4.39904100	-1.25350800
H	-4.98005200	4.98483200	-2.71987300
H	-6.17136600	3.68142700	-2.82861200
H	0.24460400	0.09879500	-3.13215600
I	2.69154300	2.96049700	-0.82925400

P-*R*

E = -3811.346803

O	1.32978400	-1.26585000	5.21718300
N	-1.33065700	-1.77010400	2.24282900
O	-1.69009700	-3.89347800	3.04227700
O	-1.81449000	-1.31065600	-0.02094200
C	0.87559200	1.03373000	3.53829200
H	0.58885500	1.72280400	2.73947500
H	1.96108100	0.89211400	3.49897600
H	0.65393500	1.49414700	4.50559000
C	0.16983800	-0.27773100	3.38994100
C	-0.72079900	-0.54359400	2.41690200
H	-0.98012400	0.20584300	1.67704200

C	-2.28373300	-1.97276000	1.16195200
H	-2.32837800	-3.04718000	0.97418600
C	-3.64430900	-1.31575900	1.43366900
H	-3.50849900	-0.48662000	2.13132000
C	-4.08604500	-0.80918800	0.07317400
H	-4.45612300	-1.62635300	-0.55285000
H	-4.83858500	-0.02258800	0.14062400
C	0.50110800	-1.34969700	4.32945500
N	-0.21325100	-2.53085500	4.12710400
C	-1.11987700	-2.83172100	3.12561300
C	-2.76175100	-0.30577800	-0.47480800
H	-2.72517800	-0.27977700	-1.56481200
H	-2.48932300	0.67369900	-0.05985800
I	-5.06372100	-2.62013700	2.40403400
H	-0.01455900	-3.29499100	4.76148600
H	-0.35291900	-1.03020800	-0.21137800
C	2.40331400	2.79452300	0.75846900
C	3.06601100	3.13620400	1.98421000
C	4.15820900	2.33710900	2.41959300
C	4.52062500	1.18462600	1.67891000
C	3.81113600	0.77001600	0.57783900
C	2.75369000	1.61424700	0.13903600
H	5.35475400	0.58234900	2.02739700
C	1.34652100	3.66979700	0.18171500
C	0.10202100	3.15782100	-0.12571900
C	1.59528900	5.05691900	-0.08757700
C	-0.98109100	3.94437800	-0.60452500
C	0.51797000	5.87375600	-0.52712100
C	-0.75428400	5.29176000	-0.75030500
H	-1.57315400	5.92886000	-1.07406300
O	2.10154100	1.25016300	-1.03539200
O	-0.10899900	1.78688200	0.07638500
P	0.56496800	0.81062500	-1.02073100
O	-0.04229200	0.88437000	-2.36163400

O	0.57860800	-0.59743400	-0.32475700
C	4.82881600	2.66635500	3.62725800
C	2.64421400	4.21262900	2.81018400
C	2.88257400	5.64718700	0.03798200
C	0.74027800	7.25492300	-0.77120100
C	3.29745200	4.49230000	3.98351800
C	4.41119200	3.72216100	4.39319300
C	3.07019500	6.98045500	-0.22163000
C	1.98679600	7.80032700	-0.61748700
C	-2.31868700	3.35979100	-0.93922700
C	-3.31782500	3.27128400	0.04724500
C	-2.59649800	2.97399500	-2.26829800
C	-4.56616100	2.73829300	-0.29668900
C	-3.85787500	2.46111800	-2.56011600
C	-4.85659400	2.32216600	-1.59501600
H	-5.33331000	2.67300000	0.46793000
H	-4.08301800	2.17846200	-3.58494100
C	4.11266000	-0.52915800	-0.09632600
C	4.47595200	-0.56567100	-1.46594600
C	4.05415300	-1.73347800	0.64560100
C	4.73541000	-1.80009900	-2.06294700
C	4.35116800	-2.94276600	-0.00239600
C	4.67978700	-3.00534800	-1.35536300
H	5.00008500	-1.82924000	-3.11779800
H	4.29790600	-3.86079800	0.57236100
H	5.66346600	2.04663400	3.94171000
H	4.91858100	3.95886000	5.32212800
H	2.95166800	5.30891100	4.60851000
H	1.78507500	4.80374800	2.51376100
H	3.72392800	5.02950500	0.32987600
H	4.06182600	7.41058400	-0.12854000
H	2.15135700	8.85469100	-0.81236300
H	-0.09780200	7.86419700	-1.09706000
Na	2.04301800	-2.20153900	-1.23483200

C	-2.03931900	-2.33038100	-3.40669800
C	0.26554400	-2.40742900	-3.56040100
C	-1.70322100	-3.76682200	-3.76978700
C	-0.20173600	-3.75287200	-4.07682700
H	-1.91035400	-4.37688100	-2.88443400
H	-2.33975000	-4.10340200	-4.58867200
N	-0.83686600	-1.63903000	-3.30332300
O	1.41869100	-2.03039100	-3.39796600
O	-3.12740400	-1.83997300	-3.21362200
H	0.35857500	-4.54119700	-3.57225800
H	0.01150700	-3.79164400	-5.14981400
C	-3.06085700	3.73993700	1.47372200
C	-2.49015500	2.59705700	2.32054300
C	-4.29806900	4.33538500	2.15051000
H	-2.30117900	4.52788800	1.42819300
H	-3.20768800	1.76796800	2.36125400
H	-1.55763800	2.22288900	1.89256800
H	-2.29065100	2.92210800	3.34634300
H	-4.77885300	5.08674500	1.51853300
H	-5.03883600	3.56582900	2.38886600
H	-4.01168100	4.80907800	3.09342800
C	-6.21776400	1.79227300	-2.02677800
C	-7.27001800	1.81356600	-0.92001300
C	-6.11685900	0.38555300	-2.63591800
H	-6.56187800	2.46664100	-2.82341900
H	-7.40913000	2.81657300	-0.50710800
H	-8.23139500	1.47205400	-1.31210000
H	-6.99583800	1.14123000	-0.09862600
H	-5.33161100	0.31281600	-3.39163400
H	-5.89589300	-0.35733300	-1.86531100
H	-7.06691200	0.10701800	-3.10086300
C	-1.60737800	3.19336700	-3.40314100
C	-1.81461300	4.58323900	-4.02328700
C	-1.70951600	2.12664600	-4.49983400

H	-0.59779600	3.14726600	-2.98798100
H	-1.64432800	5.38283100	-3.29846800
H	-1.12537700	4.73162400	-4.86027000
H	-2.83753200	4.68072000	-4.40207400
H	-1.81721100	1.12488200	-4.07911000
H	-2.56314400	2.31456300	-5.15998600
H	-0.80747900	2.14339300	-5.11697900
C	3.68869000	-1.81004900	2.12356400
C	4.92614400	-2.15455700	2.96427200
C	2.57327100	-2.82280700	2.39803800
H	3.31776900	-0.83419500	2.44825800
H	5.75679100	-1.46628300	2.78118600
H	4.67425500	-2.12083300	4.02785200
H	5.27887100	-3.16454300	2.72996900
H	1.64119800	-2.54055400	1.89668900
H	2.82302800	-3.83072700	2.05563500
H	2.39529800	-2.86895300	3.47517400
C	4.91023200	-4.31419000	-2.09082200
C	3.69794600	-4.62931500	-2.98280000
C	5.20132400	-5.49353400	-1.16483700
H	5.78130800	-4.16036700	-2.74175400
H	3.46174700	-3.80098200	-3.65735800
H	3.88975700	-5.52403300	-3.58294100
H	2.81502300	-4.82315600	-2.36010500
H	6.02786100	-5.28035000	-0.48068800
H	4.31605400	-5.75135400	-0.57383400
H	5.46586200	-6.37356400	-1.75636000
C	4.67403500	0.68668000	-2.30784500
C	3.82390400	0.66557500	-3.58325900
C	6.16254900	0.85839200	-2.64071500
H	4.37587600	1.55732700	-1.71970400
H	2.76519700	0.51380300	-3.35984800
H	3.93234900	1.61350500	-4.11839100
H	4.14322500	-0.13397900	-4.25933100

H	6.76796500	0.90129200	-1.73082900
H	6.52685900	0.02525000	-3.25006400
H	6.32035400	1.78231100	-3.20460000
H	-0.74188100	-0.66062400	-3.01343800
I	0.20933100	-4.31382300	-0.41311300

Int-III

E = -5071.735195

O	3.62483800	5.52339500	2.51311200
N	2.02981900	3.16061300	-0.34241400
O	2.04796500	1.46090200	1.19402900
O	-1.05377500	1.31216800	-2.63683000
C	3.06432500	6.80122900	0.00452400
H	4.09611700	7.05583100	0.26058000
H	2.42034300	7.43675700	0.61973600
H	2.88926700	7.03825000	-1.04717600
C	2.80469600	5.35343500	0.28398900
C	2.28056900	4.48933500	-0.60900900
H	1.99939500	4.80972600	-1.60642700
C	1.58110200	2.25433400	-1.33566100
C	1.72083900	2.45168600	-2.64784000
C	1.14468700	1.50169600	-3.66030800
H	1.62896300	1.64986900	-4.63133400
H	1.32486200	0.46674200	-3.35151500
C	3.12388500	4.85894600	1.62591300
N	2.79471000	3.51963800	1.83029600
C	2.26454100	2.62288900	0.92429000
C	-0.37274400	1.66260100	-3.82108000
H	-0.62947000	2.68859300	-4.11442900
H	-0.73135200	0.97644200	-4.59093900
I	1.82563200	-2.30170400	-1.41724600
H	2.97764000	3.14312900	2.75642600
C	-4.65496800	-1.07227100	1.24721500

C	-5.80595900	-1.92936100	1.27557100
C	-5.61583300	-3.33386800	1.39125100
C	-4.30166500	-3.86306100	1.37954700
C	-3.19620500	-3.06589100	1.20818600
C	-3.40790400	-1.66141900	1.16678300
H	-4.16879400	-4.94007100	1.44565300
C	-4.78109900	0.40945400	1.27779800
C	-4.11967900	1.17252200	0.33848200
C	-5.53684500	1.09171900	2.29067100
C	-4.17612400	2.58982300	0.29067500
C	-5.69287000	2.50293600	2.19737600
C	-5.01227100	3.21779100	1.17939300
H	-5.10616200	4.29996900	1.14613700
O	-2.27370100	-0.86905700	1.12260400
O	-3.37423400	0.53346400	-0.63927700
P	-1.91441500	0.00659900	-0.17551000
O	-0.93938800	1.06226000	0.14169600
O	-1.57119300	-1.05365700	-1.28269100
C	-6.74700600	-4.19002300	1.45961400
C	-7.13536900	-1.43766200	1.16467200
C	-6.11330000	0.42458000	3.40585600
C	-6.47119400	3.18673300	3.16853200
C	-8.20802300	-2.29079200	1.21509800
C	-8.01675700	-3.68295100	1.38032300
C	-6.84460600	1.11512500	4.33838800
C	-7.04266200	2.51018000	4.21325900
C	-3.28250200	3.37658500	-0.61190000
C	-3.50439300	3.42298000	-2.00105900
C	-2.23340400	4.11975100	-0.03658800
C	-2.72845400	4.28468300	-2.77596500
C	-1.46672600	4.95177400	-0.85979600
C	-1.72636600	5.08486100	-2.22221000
H	-2.93931800	4.36831000	-3.84150700
H	-0.68064600	5.54470400	-0.40012700

C	-1.84916100	-3.67293300	0.98924500
C	-0.80231400	-3.49744400	1.91010200
C	-1.65253300	-4.48183400	-0.15327700
C	0.40497100	-4.17748200	1.70800600
C	-0.43337500	-5.13481300	-0.30953500
C	0.60236900	-5.02137700	0.61979000
H	1.20514200	-4.03896600	2.43065600
H	-0.26934700	-5.75672400	-1.18930500
H	-6.57986300	-5.25892500	1.55912900
H	-8.87535300	-4.34470700	1.42603000
H	-9.21375300	-1.89462400	1.12031600
H	-7.29575500	-0.37446800	1.02700400
H	-5.96012000	-0.64209600	3.52108500
H	-7.26946300	0.58644500	5.18536900
H	-7.62970400	3.04150600	4.95503700
H	-6.58668100	4.26279800	3.07293500
S	3.26561200	-1.66072100	1.26578600
P	4.71992900	-0.31434900	1.20011700
C	6.37463700	-1.09146300	1.28223900
C	7.49000400	-0.48783100	0.69314700
C	6.52050800	-2.29271300	1.98044100
C	8.74509600	-1.07743800	0.81687200
H	7.37874900	0.43055800	0.12306600
C	7.77712000	-2.87710800	2.10001700
H	5.64401500	-2.77177300	2.40748800
C	8.88928700	-2.26957800	1.52145700
H	9.60762500	-0.60954000	0.35375400
H	7.88587000	-3.81241700	2.63894900
H	9.86758900	-2.73050600	1.61182500
C	4.68213300	0.82292000	2.62835400
C	3.61178200	0.76144800	3.52314100
C	5.71836800	1.73897800	2.83903900
C	3.57086000	1.63258900	4.61029400
H	2.80593000	0.05738300	3.34049500

C	5.65593700	2.62944200	3.90689000
H	6.57306800	1.76442600	2.16791000
C	4.58091200	2.57697700	4.79256100
H	2.73758500	1.58405600	5.30354900
H	6.44424300	3.36028500	4.04917100
H	4.53168300	3.27276600	5.62345800
C	4.80771500	0.67551500	-0.33195400
C	4.80025600	-0.03419800	-1.53858000
C	4.96362800	2.06293100	-0.34870300
C	4.92232300	0.64012100	-2.74725300
H	4.69498300	-1.11517400	-1.52882700
C	5.09296100	2.73455100	-1.56345900
H	4.97353200	2.62780400	0.57833100
C	5.06770500	2.02635400	-2.76082200
H	4.90300100	0.08224100	-3.67763400
H	5.20163000	3.81490400	-1.56549400
H	5.16278500	2.55311000	-3.70535300
H	1.11733300	1.36978400	-0.90925800
H	-0.87659900	1.96296000	-1.93869300
C	-4.61800800	2.62836200	-2.66331500
C	-4.08353000	1.72051600	-3.77492600
C	-5.71244600	3.56809500	-3.18351700
H	-5.07273200	1.98306600	-1.90622200
H	-3.33045900	1.03606800	-3.37966500
H	-4.90200500	1.14288200	-4.21564500
H	-3.62191000	2.30898600	-4.57648600
H	-6.10941800	4.19217700	-2.37746800
H	-5.32099700	4.23177800	-3.96166200
H	-6.53818700	2.99424900	-3.61493700
C	-1.93917900	4.10911200	1.45830300
C	-2.36032500	5.43920000	2.09704000
C	-0.46943500	3.81419200	1.75990000
H	-2.52269600	3.30794700	1.91843200
H	-3.41661000	5.66023200	1.92001800

H	-2.19071500	5.41542400	3.17768400
H	-1.77414000	6.26644100	1.68188100
H	-0.19749200	2.83787200	1.35493200
H	0.18763700	4.58624700	1.34160600
H	-0.30609100	3.80181000	2.84269800
C	-1.01122700	6.10445800	-3.09894200
C	-0.12549900	5.42702900	-4.15018900
C	-0.20406500	7.13064800	-2.30551500
H	-1.79691700	6.65141300	-3.63775000
H	-0.71155000	4.78639000	-4.81461000
H	0.38964700	6.17166300	-4.76496400
H	0.62680400	4.79769100	-3.66212300
H	-0.82104500	7.63963300	-1.56012700
H	0.62987500	6.65583500	-1.77695700
H	0.21545400	7.88374900	-2.97816000
C	-2.71548300	-4.67279300	-1.22864300
C	-3.19384300	-6.12808500	-1.28407000
C	-2.20842300	-4.21878300	-2.59937800
H	-3.57531900	-4.04339400	-0.99074100
H	-3.58623600	-6.45649500	-0.31739400
H	-3.98245800	-6.24790100	-2.03354900
H	-2.36916100	-6.79818200	-1.54903300
H	-1.91420400	-3.16664600	-2.56061800
H	-1.34774200	-4.82122700	-2.91270500
H	-2.99502800	-4.33750000	-3.35292600
C	-0.95227300	-2.64413100	3.16049300
C	0.09035600	-1.52108400	3.20888200
C	-0.87888800	-3.51597300	4.42059600
H	-1.94151100	-2.17937800	3.14770000
H	0.05680500	-0.89627900	2.31189700
H	-0.08882100	-0.88182000	4.08025300
H	1.10260400	-1.93103700	3.29441800
H	-1.64061300	-4.30082200	4.40033000
H	0.09987900	-3.99867700	4.50684000

H	-1.03375300	-2.90697400	5.31669600
C	1.87025100	-5.82734900	0.39288800
C	1.56511800	-7.32559800	0.53964000
C	3.02947500	-5.44018300	1.30706600
H	2.17654500	-5.64344800	-0.64545600
H	0.76647000	-7.64242200	-0.13630900
H	2.45532400	-7.92285400	0.31989200
H	1.24641000	-7.54800900	1.56373600
H	3.28471200	-4.38083000	1.21523500
H	2.79138300	-5.64763500	2.35676400
H	3.91618400	-6.02636700	1.04850200
H	2.24638200	3.32851000	-3.01920300
H	-1.12490700	-0.64524800	-2.05967900
C	0.54008600	-4.56855100	-4.93355400
C	1.26664000	-4.36581800	-3.60881700
C	0.09849300	-2.39655100	-3.96504700
C	-0.25035300	-3.27881500	-5.15355700
N	0.98831300	-3.07231200	-3.16034600
O	-0.33995900	-1.28344100	-3.75724600
O	1.96750300	-5.16444000	-3.04225200
H	0.01469100	-2.74645200	-6.06958800
H	-1.33300400	-3.42395800	-5.15417400
H	-0.08517700	-5.46006300	-4.85418800
H	1.28966300	-4.75922800	-5.70486500

Int-IV

E = -4711.183119

O	1.14033800	1.09728300	4.49277000
N	1.29638900	-0.37515900	0.73061000
O	-0.21562500	-1.97837400	1.44029800
O	0.90696500	1.98846500	-1.00552300
C	2.72752400	2.57574100	2.64352500
H	3.43880700	2.85219500	1.86280600

H	3.24934800	2.46631400	3.59576700
H	1.99965100	3.38531900	2.75689000
C	2.01149100	1.31273300	2.29140700
C	2.06588300	0.76102900	1.07777300
H	2.63420400	1.19680600	0.27284400
C	1.15511000	-0.83147300	-0.49022500
C	1.93503500	-0.37362800	-1.65942500
C	1.11182900	0.27513100	-2.77553300
H	1.78461500	0.38173100	-3.63116400
H	0.28682900	-0.37970000	-3.05269900
C	1.19250000	0.69505500	3.35423700
N	0.48302400	-0.44150600	2.96443100
C	0.42402400	-1.01055900	1.72934600
C	0.55680800	1.65876500	-2.33403400
H	0.96513800	2.43771800	-2.99109200
H	-0.52840300	1.65449300	-2.45433900
I	2.83218900	-2.26420100	-2.34381500
H	-0.15337300	-0.84160200	3.64633600
C	-5.18949900	-0.59951400	-0.04772800
C	-6.34437700	-1.21079600	-0.64168300
C	-6.34284900	-2.61415800	-0.87064800
C	-5.17130600	-3.36290300	-0.59316400
C	-4.01797300	-2.76430800	-0.15409900
C	-4.05653200	-1.37095500	0.13879100
H	-5.18074300	-4.43587400	-0.76594200
C	-5.16679800	0.83913100	0.32332200
C	-4.12936800	1.62858800	-0.12523900
C	-6.16114100	1.44185900	1.16558200
C	-4.02306100	3.01849800	0.15912400
C	-6.11814000	2.84735800	1.38663400
C	-5.04402300	3.60960800	0.85688600
H	-5.00988900	4.67702700	1.05713200
O	-2.92934000	-0.80758500	0.69685500
O	-3.15983800	1.07687100	-0.92939600

P	-1.98643700	0.17894800	-0.21718700
O	-1.15922200	0.98994800	0.73926100
O	-1.29610100	-0.54782300	-1.32954400
C	-7.49417400	-3.23654700	-1.42053300
C	-7.48916500	-0.46800200	-1.04168500
C	-7.17610600	0.69014500	1.81735200
C	-7.11832100	3.45903500	2.18710100
C	-8.58044700	-1.09404500	-1.58808800
C	-8.59402000	-2.49724100	-1.76714600
C	-8.11897000	1.30766400	2.59954800
C	-8.10292300	2.71070700	2.77681900
C	-2.76647600	3.72539200	-0.22043100
C	-2.44037300	3.93729300	-1.57377500
C	-1.85206300	4.07061100	0.79150100
C	-1.20056000	4.49035300	-1.88718100
C	-0.62819800	4.63607700	0.42745600
C	-0.27289900	4.83211900	-0.90268800
H	-0.93399200	4.63610800	-2.93369600
H	0.07802000	4.88276600	1.21349000
C	-2.74714500	-3.53521500	0.00593600
C	-2.38864600	-4.06128100	1.25708100
C	-1.91026000	-3.71805700	-1.11364900
C	-1.18694300	-4.76004800	1.37790200
C	-0.70363400	-4.39633800	-0.93665800
C	-0.32104100	-4.91326600	0.29986100
H	-0.89856600	-5.14537100	2.35390800
H	-0.03345400	-4.52548700	-1.78317800
H	-7.47524500	-4.31178000	-1.57609300
H	-9.46714600	-2.97956400	-2.19412000
H	-9.43985600	-0.50566700	-1.89274800
H	-7.48816600	0.60923300	-0.92100200
H	-7.19324300	-0.38710700	1.69680700
H	-8.88112400	0.71278900	3.09232400
H	-8.85907100	3.18683400	3.39229500

H	-7.07448100	4.53461800	2.33504900
S	6.80865200	-2.22564100	-0.89680800
P	6.38137100	-0.49426600	-0.08616800
C	7.83092600	0.46288300	0.46923900
C	8.18104100	1.68800500	-0.09936700
C	8.63598900	-0.09712700	1.46888700
C	9.32407100	2.35542200	0.33852300
H	7.57177800	2.12163100	-0.88573000
C	9.77071000	0.57461500	1.90481900
H	8.37282300	-1.06146000	1.89578600
C	10.11465300	1.80313700	1.34021600
H	9.59498500	3.30635300	-0.10803000
H	10.39082500	0.13898800	2.68112200
H	11.00335400	2.32519100	1.67961200
C	5.33924700	-0.63336300	1.41999700
C	4.41775900	-1.68418900	1.50541600
C	5.48631900	0.24610000	2.49860100
C	3.65631300	-1.84994800	2.65996900
H	4.33323700	-2.39130800	0.68260500
C	4.73133100	0.06615100	3.65615000
H	6.21339400	1.05209900	2.45121100
C	3.81761500	-0.98113500	3.73847400
H	2.95388800	-2.67474400	2.72595200
H	4.86608600	0.73719500	4.49861200
H	3.23353300	-1.11944000	4.64337400
C	5.46039600	0.63792300	-1.19236000
C	5.33967700	0.32690800	-2.54612900
C	4.86657800	1.80360400	-0.69136900
C	4.61198200	1.16579100	-3.39078300
H	5.79815300	-0.58365000	-2.92091000
C	4.13021100	2.63328900	-1.53221300
H	4.97488600	2.05417100	0.36194800
C	4.00192900	2.31029900	-2.88448200
H	4.51498000	0.91477700	-4.44232300

H	3.64760400	3.52001000	-1.13728000
H	3.42583900	2.95721900	-3.53914200
H	0.43496400	-1.64000900	-0.59870300
H	0.11859000	1.91487500	-0.42958400
C	-3.41448100	3.61129000	-2.69609400
C	-2.83008400	2.63343000	-3.72075000
C	-3.88114600	4.90457300	-3.37686400
H	-4.29495600	3.13432400	-2.25497000
H	-2.58899300	1.67710000	-3.24984100
H	-3.55591200	2.44733600	-4.51845300
H	-1.92494100	3.03886900	-4.18720500
H	-4.32315800	5.59262700	-2.65086300
H	-3.04010300	5.41704600	-3.85605500
H	-4.62722200	4.68734700	-4.14722200
C	-2.12927900	3.81422200	2.26614400
C	-2.59993600	5.09597000	2.96491300
C	-0.91412600	3.23311900	2.99514000
H	-2.92504700	3.06783800	2.33244000
H	-3.50869700	5.49813400	2.51009400
H	-2.80085700	4.90636900	4.02392100
H	-1.82549300	5.86827700	2.89856000
H	-0.50035000	2.41210800	2.40537500
H	-0.14324900	3.99343400	3.16567500
H	-1.20541300	2.84957000	3.97741100
C	1.08728000	5.36593000	-1.31477400
C	2.09900100	5.37668700	-0.17122200
C	0.96134000	6.76367600	-1.93318200
H	1.46720100	4.67889500	-2.08673500
H	2.16839800	4.38412700	0.28666200
H	3.08768200	5.67262400	-0.53672600
H	1.81245500	6.09319600	0.60575200
H	0.26798200	6.76532300	-2.77808700
H	0.58051400	7.46935700	-1.18787300
H	1.93241000	7.12684200	-2.28424300

C	-2.31389800	-3.23661700	-2.49987500
C	-3.15574700	-4.30043500	-3.21936900
C	-1.11557000	-2.87111600	-3.37630400
H	-2.91369800	-2.32989200	-2.37484800
H	-4.08086900	-4.52238500	-2.68457000
H	-3.41968800	-3.96018300	-4.22576800
H	-2.58499900	-5.23078600	-3.31256300
H	-0.41173100	-2.25234600	-2.81963100
H	-0.58725500	-3.76004000	-3.73865100
H	-1.45202900	-2.30852900	-4.25226200
C	-3.24163400	-3.83021700	2.49431500
C	-2.58375100	-2.79170100	3.41462900
C	-3.52809100	-5.13038500	3.25205700
H	-4.20357600	-3.41998300	2.17112200
H	-2.45049100	-1.84571200	2.88242200
H	-3.20105600	-2.62029600	4.30229700
H	-1.60030400	-3.15097800	3.74309200
H	-3.97776200	-5.87937700	2.59462200
H	-2.61344200	-5.55895900	3.67400900
H	-4.21561900	-4.94196000	4.08201900
C	1.04933600	-5.53761800	0.47248300
C	1.00276900	-6.90361400	1.16075800
C	1.95335000	-4.56788500	1.24295400
H	1.47803400	-5.67316700	-0.52869800
H	0.34910400	-7.59299600	0.62066500
H	2.00326100	-7.34325000	1.21298700
H	0.62595100	-6.81594200	2.18474500
H	1.99452000	-3.59710700	0.73477700
H	1.54568800	-4.40238900	2.24761100
H	2.97340100	-4.95571800	1.33952900
H	2.79066100	0.24317600	-1.39291800

Int-IV’

E = -4711.165683

O	4.37179300	6.04753500	3.36877400
N	1.51612000	4.05056200	1.33304100
O	0.36661100	6.06972700	1.24546300
O	0.13639900	2.71574100	-2.24798700
C	4.86860600	3.23141300	2.94290300
H	4.76518000	2.16224500	2.75196500
H	4.97465400	3.40068700	4.01753900
H	5.78144500	3.59762900	2.46492900
C	3.67146400	3.96917500	2.43213000
C	2.69931700	3.37613400	1.72933500
H	2.69473300	2.30588900	1.50905800
C	0.60728500	3.34468000	0.63459900
C	-0.81450100	3.65223200	0.55781800
C	-1.55629400	3.52794000	-0.75737600
H	-2.62588700	3.46432100	-0.54249800
H	-1.37793100	4.48168900	-1.27337800
C	3.55545900	5.39599300	2.76253900
N	2.36592800	5.99912600	2.31325200
C	1.32828300	5.44596400	1.62005300
C	-1.12048600	2.40646000	-1.68949600
H	-1.09735000	1.44394900	-1.15890800
H	-1.87251400	2.32919800	-2.48585800
I	-1.12011400	1.88375500	1.92009900
H	2.26759000	6.98960500	2.50695100
C	2.97182200	-3.16741100	0.37499700
C	3.60622200	-4.15841600	1.19504700
C	3.02774800	-4.48818800	2.45344100
C	1.87034000	-3.79800900	2.89989100
C	1.31561000	-2.77929600	2.16791200
C	1.88955600	-2.48890800	0.90093500
H	1.43115200	-4.07327600	3.85502300
C	3.44384500	-2.80918700	-0.99091800
C	3.64116500	-1.47792200	-1.29553900

C	3.60887400	-3.77234900	-2.04251800
C	3.98935800	-1.00754200	-2.59571500
C	4.08347700	-3.33261200	-3.31176700
C	4.26728700	-1.94526700	-3.55547200
H	4.57501300	-1.62515900	-4.54792200
O	1.28987900	-1.48903700	0.16940700
O	3.53080200	-0.53900000	-0.30504700
P	2.06040300	-0.04176200	0.22497800
O	1.36719800	0.83740800	-0.77277000
O	2.27787200	0.44500600	1.61801600
C	3.63773700	-5.48388800	3.26065900
C	4.81740800	-4.80105000	0.82131700
C	3.29102200	-5.14861500	-1.88565600
C	4.29255800	-4.28305400	-4.34504800
C	5.39186900	-5.74800800	1.63080900
C	4.79065100	-6.10642200	2.85999200
C	3.48815400	-6.04071100	-2.90954400
C	4.01019100	-5.60990200	-4.15091200
C	3.87462800	0.45884800	-2.85399900
C	4.70189500	1.37254700	-2.17622700
C	2.81628200	0.92701300	-3.66458300
C	4.43958800	2.73652700	-2.29018800
C	2.60320800	2.30437900	-3.75225000
C	3.37733600	3.22281400	-3.04650600
H	5.07615300	3.44594800	-1.76311600
H	1.77808000	2.66952400	-4.34878900
C	0.13538900	-2.00264700	2.65571400
C	-1.10481400	-2.12919200	2.00420700
C	0.25686200	-1.15574000	3.77826600
C	-2.22511700	-1.48080600	2.53045800
C	-0.89205700	-0.53912300	4.27617600
C	-2.14945900	-0.70348400	3.68441200
H	-3.17933800	-1.58516400	2.01941500
H	-0.81245400	0.09683300	5.15667400

H	3.17593000	-5.73057000	4.21280800
H	5.25250400	-6.86134500	3.48775900
H	6.32023300	-6.22177700	1.32860500
H	5.29143100	-4.52490300	-0.11412500
H	2.88077600	-5.49021300	-0.94215900
H	3.23512800	-7.08632800	-2.76674500
H	4.16881400	-6.32691500	-4.94971400
H	4.66751700	-3.93007300	-5.30193900
S	-4.24411100	1.35353900	-0.29057200
P	-5.44596800	-0.01313800	-1.04221500
C	-4.66040300	-1.02831100	-2.34073600
C	-3.44521600	-0.60932600	-2.88234100
C	-5.28571800	-2.17725300	-2.83797900
C	-2.86003300	-1.32872600	-3.92101800
H	-2.95508300	0.26908100	-2.47323000
C	-4.68920600	-2.90312300	-3.86296200
H	-6.22904100	-2.51539100	-2.41770000
C	-3.47830000	-2.47650400	-4.40736900
H	-1.91588500	-0.99317300	-4.33693000
H	-5.16982300	-3.80053600	-4.23819500
H	-3.01664100	-3.04313100	-5.20951700
C	-6.93997200	0.68483100	-1.83366700
C	-6.92306600	2.01289400	-2.26012900
C	-8.07023900	-0.10680900	-2.06511800
C	-8.02698900	2.54482000	-2.92137400
H	-6.04715200	2.62242200	-2.05826300
C	-9.17119500	0.42946700	-2.72417000
H	-8.10157700	-1.13419900	-1.71310500
C	-9.14879400	1.75467200	-3.15483100
H	-8.01107500	3.57878800	-3.24965300
H	-10.04835400	-0.18541500	-2.89667900
H	-10.00964800	2.17178100	-3.66730200
C	-6.09796400	-1.17034400	0.20474700
C	-7.13597000	-0.73190500	1.03529900

C	-5.51843100	-2.42289300	0.42011900
C	-7.60189500	-1.54856800	2.05911300
H	-7.57609900	0.24909200	0.87781800
C	-5.98696400	-3.23610000	1.45144500
H	-4.70595100	-2.76680100	-0.21447900
C	-7.02854000	-2.80241100	2.26683400
H	-8.40808600	-1.20484000	2.69863300
H	-5.53667700	-4.21043500	1.61246100
H	-7.39125500	-3.43907000	3.06722100
H	0.76764600	2.01638200	-1.99334200
C	5.88961600	0.92882300	-1.33679300
C	5.83546500	1.50700500	0.08126200
C	7.20242900	1.30714800	-2.03402800
H	5.86237500	-0.16112100	-1.24899100
H	4.90301700	1.22047800	0.57420800
H	6.67121500	1.12960100	0.67957200
H	5.90745500	2.60062700	0.06787300
H	7.25607200	0.86661900	-3.03350000
H	7.28547100	2.39389400	-2.14153100
H	8.06270600	0.95751000	-1.45488000
C	1.86364200	-0.02773900	-4.38779900
C	1.05596800	0.64625600	-5.49992300
C	0.88644300	-0.72429600	-3.42703100
H	2.46758800	-0.80901000	-4.85899600
H	1.69381000	1.19390700	-6.19957600
H	0.50475500	-0.11320900	-6.06283000
H	0.32313100	1.34542900	-5.08207300
H	1.38265200	-1.23453500	-2.60011000
H	0.20179200	0.00920800	-2.98653000
H	0.30013600	-1.46829900	-3.97741900
C	3.06844900	4.71114200	-3.02424200
C	2.50476000	5.09694800	-1.64701100
C	2.09340700	5.15485000	-4.11303800
H	4.01768200	5.24611000	-3.16431200

H	3.18931500	4.80354300	-0.84252300
H	2.32939700	6.17605200	-1.58046100
H	1.54967800	4.57684400	-1.50723000
H	2.44073800	4.86484100	-5.10868700
H	1.10941200	4.70519400	-3.94343100
H	1.97601500	6.24204800	-4.09434800
C	1.59166700	-0.88299400	4.45758800
C	1.69568400	-1.63847500	5.78838600
C	1.82427600	0.61603000	4.68131500
H	2.38271200	-1.22924100	3.78733600
H	1.59169400	-2.71818900	5.65271800
H	2.66194000	-1.44637500	6.26500900
H	0.90802300	-1.31227900	6.47682100
H	1.72108500	1.15452400	3.73760500
H	1.12800500	1.03136100	5.41808900
H	2.83769000	0.77698000	5.06358400
C	-1.29670200	-2.95639200	0.74221500
C	-1.78732100	-2.07285600	-0.41069800
C	-2.24968400	-4.12852700	1.00567100
H	-0.33655100	-3.38373000	0.44490400
H	-1.03959000	-1.31180000	-0.65611300
H	-1.97827600	-2.67426300	-1.30715400
H	-2.71504200	-1.55215400	-0.14704100
H	-1.84552400	-4.79460900	1.77360700
H	-3.22178800	-3.76942200	1.35730800
H	-2.40735600	-4.71007500	0.09141300
C	-3.37493400	-0.08327400	4.33532500
C	-3.75595900	-0.89566100	5.58139200
C	-4.57349200	0.03751600	3.39542500
H	-3.09291900	0.92619800	4.66723200
H	-2.92359200	-0.96052500	6.28733700
H	-4.61107500	-0.44335800	6.09317700
H	-4.03013500	-1.91496200	5.28954600
H	-4.32536200	0.56899800	2.47073900

H	-4.95466400	-0.95269400	3.11939600
H	-5.38891500	0.57200400	3.89214100
H	-1.12166100	4.51255700	1.13817600
H	0.99177700	2.45425000	0.11554700

TS-II

E = -4711.182479

O	1.11413400	0.95584900	4.55163200
N	1.22385100	-0.44565200	0.75762700
O	-0.18025300	-2.12899900	1.48498600
O	0.87996700	2.06692200	-0.94157000
C	2.67777800	2.47484200	2.70533900
H	3.24711600	2.85200000	1.85324700
H	3.35306500	2.29915000	3.54560000
H	1.95938200	3.23875400	3.01483700
C	1.95102500	1.22033000	2.34214500
C	1.96249800	0.70698500	1.11035200
H	2.49417000	1.18261900	0.30404900
C	1.04255800	-0.87974400	-0.48033900
C	1.81678000	-0.35254200	-1.64031300
C	1.04748700	0.38262500	-2.74067400
H	1.77214000	0.55434900	-3.54124000
H	0.24883500	-0.24874900	-3.12677400
C	1.17424200	0.56467200	3.40883300
N	0.51878900	-0.60452400	3.01834400
C	0.44066000	-1.14375900	1.77008100
C	0.45350000	1.72926800	-2.24708100
H	0.77140400	2.53093900	-2.92784800
H	-0.63445800	1.67127300	-2.28737700
I	2.78159400	-2.16164500	-2.44407800
H	-0.08406700	-1.04189300	3.70708800
C	-5.11757700	-0.61909800	-0.00360800
C	-6.27715800	-1.20944800	-0.61136500

C	-6.29343300	-2.61029700	-0.85251200
C	-5.12897600	-3.37309800	-0.58662600
C	-3.96873100	-2.79192800	-0.14276600
C	-3.99354100	-1.40431100	0.17699100
H	-5.14418300	-4.44200500	-0.78376500
C	-5.09012200	0.81500600	0.38092200
C	-4.05166200	1.60884900	-0.05567000
C	-6.09628000	1.41300200	1.21298700
C	-3.96591300	3.00318000	0.21175600
C	-6.06811000	2.81936300	1.42624800
C	-5.00079800	3.58858100	0.89376000
H	-4.98534600	4.65960200	1.07469000
O	-2.86494500	-0.86979900	0.76199000
O	-3.05343400	1.05186600	-0.82972100
P	-1.90801800	0.19184100	-0.03715300
O	-1.17662000	1.00272500	0.98478400
O	-1.09189500	-0.47494900	-1.11765300
C	-7.44996000	-3.21419900	-1.41200300
C	-7.41036600	-0.44892000	-1.01157800
C	-7.10857100	0.65467300	1.86093900
C	-7.07937800	3.42642900	2.21622600
C	-8.50706700	-1.05674000	-1.56766000
C	-8.53877700	-2.45823700	-1.75705100
C	-8.06181200	1.26743700	2.63413900
C	-8.06016500	2.67157100	2.80383300
C	-2.73085200	3.73567400	-0.18843100
C	-2.43766400	3.95392100	-1.54806800
C	-1.81365400	4.12079600	0.80699200
C	-1.22109200	4.54314200	-1.88620400
C	-0.61531600	4.72329500	0.41753900
C	-0.28647700	4.91807600	-0.92030800
H	-0.97931200	4.68832500	-2.93846900
H	0.09310700	5.00254200	1.18980700
C	-2.69934900	-3.57380500	-0.04238200

C	-2.33554500	-4.20624200	1.15612700
C	-1.87474100	-3.66610400	-1.18176400
C	-1.13256600	-4.91143700	1.20850100
C	-0.66870600	-4.35944000	-1.07391300
C	-0.27596800	-4.97515600	0.11383000
H	-0.83617600	-5.37748400	2.14568300
H	-0.00767200	-4.42315100	-1.93500000
H	-7.44339700	-4.28821700	-1.57646300
H	-9.41631800	-2.92573600	-2.19131800
H	-9.35690400	-0.45462300	-1.87223000
H	-7.39710800	0.62728600	-0.88408700
H	-7.11474800	-0.42327200	1.74570500
H	-8.82125200	0.66810200	3.12559600
H	-8.82468200	3.14355400	3.41207500
H	-7.04682700	4.50314700	2.35831700
S	6.77311400	-2.14913800	-1.01451300
P	6.33442300	-0.46979300	-0.10597000
C	7.78088300	0.46869200	0.48934500
C	8.09576200	1.74376600	0.01849000
C	8.61557400	-0.15438000	1.42531100
C	9.23382400	2.39667900	0.49021900
H	7.46285400	2.22799000	-0.71806400
C	9.74517900	0.50266200	1.89542500
H	8.37933800	-1.15610700	1.77477300
C	10.05423700	1.78051900	1.42864700
H	9.47742300	3.38677200	0.11937400
H	10.38844700	0.01757900	2.62200200
H	10.93894500	2.29144800	1.79443100
C	5.30791800	-0.69851300	1.39848100
C	4.38503700	-1.75084100	1.42749700
C	5.45880900	0.12094400	2.52235400
C	3.62423300	-1.97626000	2.57180600
H	4.29708600	-2.41033100	0.56634500
C	4.70276400	-0.11779500	3.66856400

H	6.18659600	0.92767900	2.51652100
C	3.78611400	-1.16495500	3.69414500
H	2.91692800	-2.79937500	2.59364200
H	4.83701000	0.50824600	4.54515400
H	3.19868000	-1.34766300	4.58899200
C	5.38767400	0.71068600	-1.13590300
C	5.25824500	0.47939700	-2.50460700
C	4.78061600	1.83376100	-0.55932700
C	4.51029200	1.35683600	-3.29016700
H	5.72549100	-0.40018900	-2.93789000
C	4.02661200	2.70265100	-1.34247500
H	4.89011300	2.01841000	0.50732700
C	3.88890700	2.45952500	-2.71018800
H	4.40540000	1.16765300	-4.35387900
H	3.53357800	3.55685100	-0.89185800
H	3.29491300	3.13474200	-3.31901500
H	0.48608000	-1.80785700	-0.55630500
H	0.11866100	2.04840800	-0.33273400
C	-3.42554300	3.60127300	-2.65082300
C	-2.84811200	2.61857700	-3.67510400
C	-3.91959800	4.88057700	-3.33886000
H	-4.29349100	3.11994600	-2.18986300
H	-2.60786900	1.66246900	-3.20347100
H	-3.57773400	2.43270200	-4.46931900
H	-1.94268500	3.01881900	-4.14529700
H	-4.35365700	5.57364700	-2.61291300
H	-3.09458800	5.39540200	-3.84256800
H	-4.67912700	4.64499800	-4.09038300
C	-2.06897000	3.88129500	2.28855900
C	-2.60598900	5.15154000	2.96099900
C	-0.82035000	3.39107700	3.02723100
H	-2.81998100	3.09159600	2.37439900
H	-3.53782700	5.49450400	2.50502400
H	-2.78981300	4.97476900	4.02526800

H	-1.87447700	5.96244600	2.87233900
H	-0.35917800	2.57664700	2.46492400
H	-0.09492800	4.20063100	3.16903000
H	-1.08691300	3.02072700	4.02127400
C	1.06030200	5.46708000	-1.35889000
C	2.04977500	5.63217200	-0.20744400
C	0.90013100	6.79323600	-2.11226200
H	1.48053700	4.72234200	-2.05324800
H	2.15331900	4.69957300	0.35673900
H	3.03304400	5.92547500	-0.58809000
H	1.71909900	6.41136600	0.48746700
H	0.22768200	6.69319100	-2.96773000
H	0.48084200	7.55354700	-1.44561500
H	1.86668500	7.15395200	-2.47748200
C	-2.29972200	-3.07736800	-2.51988300
C	-3.17531100	-4.07141700	-3.29703200
C	-1.11517100	-2.66829400	-3.39387700
H	-2.88419600	-2.17337900	-2.31721600
H	-4.09228000	-4.31936700	-2.75994100
H	-3.45454100	-3.65276000	-4.26910300
H	-2.62070000	-4.99964700	-3.47172100
H	-0.39021200	-2.10247200	-2.80951300
H	-0.60754000	-3.53671800	-3.82775800
H	-1.45953200	-2.04094700	-4.22178400
C	-3.18518200	-4.07697400	2.40995700
C	-2.56409900	-3.05429000	3.37226500
C	-3.40642800	-5.42103600	3.11004100
H	-4.16747200	-3.69575200	2.11274500
H	-2.46766700	-2.08046500	2.88432500
H	-3.18200700	-2.94727200	4.26957600
H	-1.56504700	-3.38873900	3.67769100
H	-3.81465600	-6.16488200	2.42046500
H	-2.47329400	-5.81899800	3.52114900
H	-4.10553900	-5.30200400	3.94304300

C	1.09661600	-5.60916900	0.22069100
C	1.06423200	-7.01654400	0.82039100
C	2.01376200	-4.68829300	1.03428100
H	1.50652400	-5.67932300	-0.79495100
H	0.39833600	-7.67074300	0.25210700
H	2.06560700	-7.45709900	0.82101700
H	0.71204000	-6.99607900	1.85658600
H	2.04439900	-3.68719800	0.58796000
H	1.62495700	-4.58803500	2.05472800
H	3.03598300	-5.07833900	1.08637500
H	2.66378100	0.25156500	-1.31989700

Int-V

E = -4711.216345

O	1.77280300	0.14265600	4.80052200
N	1.03558800	-0.49432900	0.87916200
O	-0.07876100	-2.38959200	1.51375100
O	0.61845300	2.44267900	-0.90853100
C	2.66592100	2.20228100	2.99821000
H	2.75920100	2.90007000	2.16107400
H	3.66455400	1.97879400	3.38811800
H	2.10494700	2.68960300	3.80000700
C	1.97583900	0.94592500	2.56749900
C	1.65274000	0.68060900	1.29010000
H	1.83296700	1.40745800	0.51043700
C	0.55470000	-0.72428200	-0.47010000
C	1.47782700	-0.08518100	-1.53135900
C	0.86519300	0.77767100	-2.63758900
H	1.71329200	1.12203200	-3.23579400
H	0.22217500	0.19116200	-3.29675900
C	1.57676200	0.00366300	3.60880900
N	0.91369600	-1.12615500	3.12398000
C	0.57590700	-1.41430500	1.82589900

C	0.06781100	1.98966700	-2.13364200
H	0.11419800	2.78415000	-2.89471100
H	-0.98249800	1.72436300	-1.99585000
I	2.68886300	-1.68672800	-2.42808300
H	0.54747400	-1.77098900	3.81388000
C	-4.99187500	-0.81734600	0.11347900
C	-6.16397000	-1.34159200	-0.53565300
C	-6.18529800	-2.70910600	-0.92381100
C	-5.01524200	-3.49457500	-0.76906300
C	-3.84848000	-2.96414100	-0.28457500
C	-3.87389700	-1.62217300	0.18692900
H	-5.02946900	-4.53199800	-1.09314100
C	-4.96933500	0.57789500	0.62377400
C	-3.97174500	1.43648400	0.22124200
C	-5.97202800	1.09017800	1.51548700
C	-3.95745400	2.82537400	0.50830500
C	-5.98124500	2.47977900	1.81849800
C	-4.98383200	3.32586600	1.26730800
H	-5.02565400	4.39211600	1.47191400
O	-2.72032400	-1.18332400	0.81656000
O	-2.90018900	0.93222000	-0.52162000
P	-1.86909600	0.12053300	0.42445000
O	-1.36220000	0.81736700	1.61407800
O	-0.80450000	-0.26113300	-0.69090200
C	-7.35141600	-3.25197100	-1.52379000
C	-7.30284000	-0.54462400	-0.83510600
C	-6.93719600	0.25486100	2.13870600
C	-6.98011500	2.99792600	2.68367800
C	-8.40942000	-1.09217500	-1.43330700
C	-8.44597800	-2.46551200	-1.76782300
C	-7.87824400	0.78207300	2.98600100
C	-7.91180500	2.17067100	3.25328200
C	-2.88301100	3.69454400	-0.05359100
C	-2.87620400	3.98129700	-1.43098900

C	-1.89370200	4.23356100	0.79568400
C	-1.87396300	4.81209900	-1.93938300
C	-0.93251400	5.08361700	0.24322000
C	-0.89854900	5.37618200	-1.12244900
H	-1.85457500	5.03018000	-3.00552200
H	-0.17528000	5.51109800	0.89625700
C	-2.55816800	-3.71263400	-0.32293000
C	-2.13779300	-4.49935000	0.75829700
C	-1.75546200	-3.57649800	-1.47241900
C	-0.89182900	-5.12188600	0.68707400
C	-0.50847300	-4.20088200	-1.48932600
C	-0.05212900	-4.95692300	-0.41004400
H	-0.54517300	-5.70089200	1.54019800
H	0.14096300	-4.08563300	-2.35350200
H	-7.34780100	-4.30240100	-1.80079700
H	-9.33134000	-2.88596300	-2.23286500
H	-9.26349100	-0.46183100	-1.65821700
H	-7.28988600	0.51258200	-0.59792300
H	-6.91377200	-0.81202400	1.94740000
H	-8.60053600	0.12650000	3.46096900
H	-8.66666900	2.57368600	3.92012200
H	-6.97890200	4.06325800	2.89588900
S	6.77072300	-1.90769200	-1.40016200
P	6.36123000	-0.37487400	-0.24501300
C	7.84458200	0.52480400	0.33312400
C	7.92034000	1.91889000	0.35752800
C	8.92328200	-0.23837500	0.79247000
C	9.06575100	2.54404900	0.84758400
H	7.09420300	2.51966500	-0.00938800
C	10.06112400	0.39033100	1.28382800
H	8.86847700	-1.32236200	0.74460200
C	10.13277300	1.78240100	1.31308200
H	9.12288300	3.62760100	0.85995200
H	10.89533700	-0.20564000	1.63901300

H	11.02360300	2.27194800	1.69330900
C	5.48269400	-0.81526700	1.29505900
C	4.57743100	-1.88075700	1.26757300
C	5.71140100	-0.12340100	2.48880200
C	3.91342900	-2.25208700	2.43248000
H	4.42267300	-2.42933300	0.34165700
C	5.03852400	-0.49770800	3.64982500
H	6.42369400	0.69678000	2.51703200
C	4.14778400	-1.56681300	3.62335000
H	3.21681200	-3.08400400	2.41237400
H	5.21096800	0.04065000	4.57623600
H	3.62098400	-1.84828400	4.52942800
C	5.31671000	0.90053100	-1.03682100
C	5.31535700	0.99590400	-2.42882000
C	4.54319000	1.78395000	-0.27486000
C	4.55054900	1.97491900	-3.05713400
H	5.89194700	0.28021200	-3.00777000
C	3.78179100	2.76437900	-0.90742400
H	4.52264300	1.69616200	0.80948900
C	3.78557700	2.85773300	-2.29864300
H	4.54429200	2.04035000	-4.14042500
H	3.16222900	3.43782200	-0.32278300
H	3.18463000	3.61647100	-2.79041100
H	0.47951000	-1.80625000	-0.58354500
H	-0.01338000	3.04742000	-0.49206800
C	-3.94066800	3.45263400	-2.38252900
C	-3.35025800	2.52390900	-3.45026100
C	-4.70833700	4.61298100	-3.02881100
H	-4.66336800	2.87029700	-1.80382300
H	-2.92180300	1.62668900	-2.99619400
H	-4.13045200	2.21095700	-4.15055000
H	-2.56750700	3.03032300	-4.02572700
H	-5.13325600	5.27406400	-2.26860700
H	-4.05417000	5.21275300	-3.66949700

H	-5.52381900	4.23009000	-3.64916200
C	-1.84055400	3.91990500	2.28299400
C	-2.39339500	5.09052200	3.10523200
C	-0.42207300	3.56799400	2.74123800
H	-2.45867500	3.03639700	2.46175300
H	-3.43021100	5.31842900	2.84365100
H	-2.35364900	4.85814000	4.17344400
H	-1.79906000	5.99438300	2.93185700
H	0.00821600	2.79621500	2.09937200
H	0.23486900	4.44536500	2.74720400
H	-0.44996300	3.17062200	3.76079000
C	0.19024200	6.25823800	-1.69928100
C	1.57571500	5.67905200	-1.39122000
C	0.06935500	7.69971800	-1.19517300
H	0.05951200	6.26390500	-2.78872600
H	1.61151600	4.61648300	-1.64821900
H	2.35681600	6.21313400	-1.94088700
H	1.80308800	5.76681100	-0.32261700
H	-0.90607300	8.12492800	-1.44478700
H	0.18426000	7.73648700	-0.10691300
H	0.84651500	8.33044700	-1.63686700
C	-2.25118700	-2.81384800	-2.69419600
C	-3.08615700	-3.72959900	-3.60043600
C	-1.12006100	-2.19223500	-3.51032700
H	-2.89408900	-1.99504800	-2.34773900
H	-3.96135200	-4.12735500	-3.08339800
H	-3.43062400	-3.18395400	-4.48456200
H	-2.47569600	-4.57484500	-3.93494200
H	-0.42048700	-1.66604300	-2.86036800
H	-0.55409600	-2.94631300	-4.06653500
H	-1.52544700	-1.48162400	-4.23755100
C	-2.97300900	-4.62681000	2.02111200
C	-2.36772400	-3.78611000	3.15291000
C	-3.14679400	-6.08595000	2.45386200

H	-3.97031400	-4.22763700	1.80737100
H	-2.28436800	-2.73788100	2.85611000
H	-2.98356900	-3.86004900	4.05501400
H	-1.35923600	-4.14286000	3.38963600
H	-3.55821500	-6.69440300	1.64370600
H	-2.19302400	-6.52929900	2.75644900
H	-3.82368800	-6.14824800	3.31109800
C	1.36332500	-5.49794800	-0.40430400
C	1.43030900	-6.98967000	-0.06897800
C	2.21319900	-4.67545200	0.57181700
H	1.77477000	-5.35394500	-1.41153600
H	0.81538000	-7.57705700	-0.75575000
H	2.46106900	-7.35046800	-0.13191600
H	1.07526600	-7.18093600	0.94873500
H	2.17735100	-3.61146400	0.31245400
H	1.82037100	-4.78143400	1.58988000
H	3.25856500	-5.00299000	0.56334300
H	2.25964100	0.49629400	-1.05142500

TS-III

E = -4711.165043

O	-1.45674300	2.55449900	-2.73143700
N	-1.58677900	0.28612800	0.59925300
O	-1.39882600	-1.53666500	-0.80996600
O	-0.24357200	-2.23391000	2.81961400
C	-1.48935200	3.98619200	-0.21249900
H	-2.23391600	4.33131300	0.50920700
H	-1.68516400	4.44966400	-1.17921600
H	-0.49470800	4.29160900	0.12728800
C	-1.53366200	2.49738800	-0.34812000
C	-1.55995400	1.69222300	0.71436200
H	-1.53789000	2.07831100	1.72368100
C	-1.81155500	-0.53314800	1.58928300

C	-1.96944700	-0.07287300	2.98971600
C	-0.58882700	0.01085400	3.66907900
H	0.07187300	0.57233100	2.99431400
H	-0.68902300	0.56634100	4.60670800
C	-1.50741300	1.91040900	-1.71043800
N	-1.63387500	0.52070300	-1.75984200
C	-1.48395100	-0.34634700	-0.72345100
C	0.01582600	-1.37714000	3.90062500
H	1.09209900	-1.26291700	4.08157400
H	-0.43605900	-1.82938400	4.78913000
I	-3.35123600	-1.39746400	4.03238000
H	-1.52684000	0.09514400	-2.67647200
C	4.86029800	-1.21167100	-0.85991400
C	6.02273900	-2.03690300	-0.66203300
C	5.87174700	-3.45167800	-0.65284000
C	4.57042100	-4.01382000	-0.69316200
C	3.44874400	-3.22646800	-0.71041700
C	3.62040800	-1.81915600	-0.85231300
H	4.46171000	-5.09352600	-0.61680500
C	4.97132700	0.26194600	-1.01017900
C	4.18248900	1.10091400	-0.24252700
C	5.89965300	0.85801600	-1.92983500
C	4.36714700	2.51231900	-0.20934700
C	6.13310500	2.25923300	-1.86892900
C	5.37567700	3.04965100	-0.96994700
H	5.55403700	4.12213900	-0.93443600
O	2.49040800	-1.07612000	-1.07538600
O	3.25289900	0.58328000	0.61921600
P	1.85553900	-0.09166000	0.07387300
O	0.96977100	0.86512100	-0.65335100
O	1.32659900	-0.84210000	1.25920300
C	7.01584900	-4.28070500	-0.51479000
C	7.32411400	-1.50682600	-0.44406700
C	6.58594200	0.10804500	-2.92400300

C	7.08217200	2.85180900	-2.74360600
C	8.40949900	-2.33246000	-0.29229200
C	8.26228400	-3.73751300	-0.34782600
C	7.48101100	0.71076700	-3.77057900
C	7.74839200	2.09663300	-3.67189400
C	3.45631300	3.39346900	0.57857200
C	3.37581000	3.29784200	1.98131900
C	2.68900200	4.36200000	-0.10692600
C	2.55359800	4.19096000	2.67171700
C	1.89035600	5.23747400	0.63195200
C	1.81605500	5.17652900	2.02367200
H	2.50707000	4.13287600	3.75756500
H	1.31475300	5.99434000	0.09951100
C	2.08791800	-3.77373800	-0.44706200
C	1.07558200	-3.71523500	-1.42362500
C	1.80999300	-4.28817000	0.83692000
C	-0.19973100	-4.17386200	-1.10070900
C	0.50607800	-4.69937600	1.11951600
C	-0.51319100	-4.64509100	0.17318900
H	-0.97805400	-4.13075000	-1.86056500
H	0.26980400	-5.05627400	2.11979800
H	6.87554700	-5.35817800	-0.52429200
H	9.13055200	-4.37848400	-0.23551200
H	9.39027900	-1.90126800	-0.11945700
H	7.45494700	-0.43296600	-0.38443100
H	6.38348600	-0.95298900	-3.01555800
H	7.98489500	0.11945600	-4.52826600
H	8.46566000	2.55838100	-4.34248900
H	7.25332500	3.92240800	-2.67082200
S	-4.89038200	-0.49736900	0.77845900
P	-5.60305700	0.25963900	-0.90509300
C	-7.42211500	0.15290500	-1.00517900
C	-8.16431700	1.09344800	-1.72625400
C	-8.06632400	-0.92240700	-0.38913500

C	-9.54453000	0.94974800	-1.83658600
H	-7.67091900	1.94524500	-2.18634200
C	-9.44585800	-1.06024600	-0.50372400
H	-7.48322500	-1.63315000	0.18929400
C	-10.18426800	-0.12690200	-1.22782400
H	-10.11962600	1.68350300	-2.39161000
H	-9.94505600	-1.89433300	-0.02200600
H	-11.26077900	-0.23477000	-1.31206000
C	-4.99562000	-0.57432000	-2.40931400
C	-4.08737100	-1.62372800	-2.28314700
C	-5.44167500	-0.18495100	-3.67846300
C	-3.59127900	-2.25642600	-3.42157500
H	-3.76655000	-1.94142600	-1.29680300
C	-4.94362200	-0.81774100	-4.81156900
H	-6.17109400	0.61424500	-3.78280400
C	-4.01197700	-1.84897100	-4.68396800
H	-2.87883400	-3.06910600	-3.31371600
H	-5.28253200	-0.50854400	-5.79460600
H	-3.62232700	-2.33838500	-5.57058500
C	-5.25210100	2.03654200	-1.10665900
C	-5.74621000	2.89892900	-0.11841400
C	-4.50413500	2.55144500	-2.16489000
C	-5.50536900	4.26398800	-0.20377200
H	-6.31409700	2.49356100	0.71502200
C	-4.25847300	3.92286900	-2.24245900
H	-4.09441600	1.89467200	-2.92532000
C	-4.76388000	4.77764400	-1.26965400
H	-5.89442900	4.92914800	0.56013100
H	-3.65283000	4.30645100	-3.05686000
H	-4.57169800	5.84411900	-1.33192900
H	-1.82369500	-1.58746600	1.33665700
H	0.39805100	-2.00102500	2.11151800
C	4.19402400	2.30464500	2.79400600
C	3.29640200	1.35555000	3.59884600

C	5.17967700	3.03933000	3.71206000
H	4.78428200	1.69507200	2.10656400
H	2.63710000	0.78816800	2.93638900
H	3.91057400	0.64310400	4.15907000
H	2.68865000	1.91012600	4.32352900
H	5.82913600	3.70558100	3.13731100
H	4.65367400	3.64364000	4.45836400
H	5.80878600	2.32109800	4.24643500
C	2.66119300	4.47117900	-1.62712100
C	3.11792900	5.85146500	-2.10932000
C	1.27398300	4.11905500	-2.16840300
H	3.34868800	3.73305400	-2.03992400
H	4.12403900	6.08854200	-1.75004100
H	3.12608100	5.88784900	-3.20285600
H	2.44522700	6.64009100	-1.75578400
H	0.96037300	3.14475000	-1.78242100
H	0.53374400	4.87488100	-1.88530900
H	1.28784800	4.06566600	-3.26127900
C	0.99508900	6.17656800	2.81643400
C	-0.49431800	6.10951500	2.46640500
C	1.52965500	7.60040000	2.62523300
H	1.10204300	5.91479800	3.87662300
H	-0.88783400	5.09909700	2.61451700
H	-1.07104900	6.79931500	3.09020400
H	-0.66137400	6.38672700	1.41959900
H	2.58667700	7.66072400	2.89591300
H	1.43342400	7.91095300	1.57967400
H	0.97134500	8.31222800	3.24106100
C	2.85821500	-4.41438300	1.93800500
C	3.00004700	-5.87121700	2.39405700
C	2.56405800	-3.49107600	3.12341700
H	3.82418400	-4.10535600	1.53850100
H	3.23297300	-6.52922200	1.55122600
H	3.80085200	-5.96212400	3.13446000

H	2.07642300	-6.23511400	2.85536600
H	2.52083000	-2.45086600	2.78429200
H	1.60749700	-3.73992100	3.59319300
H	3.35562100	-3.58369400	3.87439300
C	1.35328700	-3.23213400	-2.84026900
C	0.47092000	-2.04767400	-3.25290200
C	1.20181300	-4.39156900	-3.83349900
H	2.39226300	-2.89276400	-2.88645700
H	0.63060300	-1.19852000	-2.58298100
H	0.71888400	-1.73833800	-4.27378600
H	-0.59027000	-2.32215100	-3.22953100
H	1.85130000	-5.22831900	-3.56192400
H	0.17019000	-4.76029400	-3.84809600
H	1.45850300	-4.06844900	-4.84724500
C	-1.89598100	-5.16606000	0.51887200
C	-2.07891700	-6.58036600	-0.04695200
C	-3.02806900	-4.25338100	0.04391700
H	-1.95177600	-5.22478900	1.61312700
H	-1.29687100	-7.25146100	0.31785900
H	-3.05286000	-6.99400200	0.23343100
H	-2.01868300	-6.55982700	-1.14064000
H	-2.93220200	-3.24185000	0.44854700
H	-3.04122100	-4.17750900	-1.04926900
H	-3.99861200	-4.64978700	0.35716000
H	-2.48283700	0.88676400	3.01932400

P-*S*

E = -4711.234576

O	2.27994500	3.25586800	2.42483800
N	1.44527700	0.31436700	-0.18646800
O	2.42027000	-1.14648900	1.26216900
O	0.51275100	-0.41119400	-2.22065600
C	0.75428900	4.02380100	0.03490700

H	1.36905200	4.48532300	-0.74646600
H	0.80933400	4.65461500	0.92474300
H	-0.28269000	3.99290300	-0.31423200
C	1.23333200	2.64190300	0.36972100
C	1.03336500	1.60028000	-0.45913600
H	0.48222700	1.71871600	-1.38213800
C	1.41047100	-0.76645100	-1.19619100
C	2.78063500	-0.83643700	-1.87573200
C	2.66129800	0.28426700	-2.90097600
H	2.82502600	1.24225200	-2.39241700
H	3.37923300	0.20579300	-3.71738900
C	1.93784300	2.40277300	1.62332800
N	2.24215000	1.05580600	1.86191500
C	2.06106400	-0.00922200	1.00974000
C	1.21097100	0.14641200	-3.35830800
H	0.73966700	1.09717900	-3.61927800
H	1.10538900	-0.55764400	-4.18846100
I	3.07418100	-2.74703100	-2.88725500
H	2.73464400	0.84442100	2.72560100
C	-4.95699900	-1.67990700	0.53662400
C	-5.83711800	-2.78968100	0.31833600
C	-5.28274400	-4.09524600	0.21462200
C	-3.87672900	-4.26474100	0.26635000
C	-3.01593900	-3.19687600	0.35248200
C	-3.59640900	-1.91062200	0.49109900
H	-3.46607100	-5.26866200	0.20829300
C	-5.45143700	-0.30254000	0.79809700
C	-4.94960600	0.74848200	0.06267300
C	-6.37106200	-0.00219200	1.85933300
C	-5.28506000	2.10771300	0.28866500
C	-6.81154600	1.34124400	2.02648200
C	-6.25303500	2.36955100	1.22489000
H	-6.55671000	3.39781000	1.40189800
O	-2.71861900	-0.84700800	0.63989200

O	-4.07403000	0.48277300	-0.98341000
P	-2.54514300	0.16712200	-0.60162500
O	-1.67306300	1.29255700	-0.23832100
O	-2.13541300	-0.68995800	-1.86015100
C	-6.14654900	-5.20675900	0.02635100
C	-7.24237300	-2.63730800	0.16991000
C	-6.83773600	-0.98169300	2.77781300
C	-7.75008900	1.64695600	3.04716900
C	-8.04914700	-3.72828400	-0.02887100
C	-7.50003600	-5.03131000	-0.08857000
C	-7.73191500	-0.65200200	3.76435400
C	-8.20903100	0.67321000	3.89397500
C	-4.51571300	3.19702100	-0.38161200
C	-4.68318600	3.46461900	-1.74903200
C	-3.59807400	3.94495800	0.38759600
C	-3.97385000	4.52400600	-2.31836800
C	-2.91905300	4.99766600	-0.22534500
C	-3.10072300	5.31050600	-1.57337000
H	-4.11363500	4.75026900	-3.37373400
H	-2.22588400	5.58989800	0.36969000
C	-1.53907600	-3.41931800	0.29211800
C	-0.71886400	-3.18049400	1.41452900
C	-0.98492000	-3.99193300	-0.87221100
C	0.60846900	-3.61106200	1.38487500
C	0.33980000	-4.43295800	-0.83814200
C	1.14647000	-4.28338900	0.28799600
H	1.23770800	-3.42478300	2.25138600
H	0.76187300	-4.90920200	-1.72038600
H	-5.70637500	-6.19783400	-0.04001400
H	-8.15206600	-5.88510900	-0.24003000
H	-9.11888600	-3.59096700	-0.14781500
H	-7.67170200	-1.64216100	0.20529400
H	-6.47164100	-1.99861800	2.69967300
H	-8.07072000	-1.41480800	4.45763300

H	-8.92134100	0.91721800	4.67508000
H	-8.08191700	2.67613600	3.15148700
S	6.22223700	-1.32012200	-0.56123800
P	6.37088900	0.35708500	0.45920500
C	8.08410400	0.96144900	0.65038200
C	8.38131400	2.32682600	0.68369800
C	9.10621300	0.02063500	0.80063800
C	9.69486500	2.74586400	0.88054300
H	7.59357200	3.06231900	0.54525300
C	10.41581500	0.44549800	0.99681700
H	8.86897800	-1.03795000	0.74368600
C	10.71027700	1.80689300	1.03894800
H	9.92411900	3.80620500	0.90196700
H	11.20827600	-0.28678900	1.11057100
H	11.73362300	2.13599700	1.18796600
C	5.72221100	0.25341600	2.16259800
C	4.97039100	-0.86079700	2.52768900
C	5.93919400	1.28582900	3.08630900
C	4.39648400	-0.92501000	3.79796000
H	4.81058600	-1.65905000	1.81099300
C	5.35900200	1.22219200	4.34698900
H	6.55563000	2.14011500	2.81878800
C	4.58145100	0.11670100	4.70156800
H	3.79214100	-1.78598300	4.06522100
H	5.51223400	2.03051500	5.05366900
H	4.12668500	0.07065300	5.68594500
C	5.49669200	1.75253700	-0.33810800
C	5.64073700	1.89456200	-1.72454200
C	4.75665400	2.69387000	0.37932200
C	5.05951900	2.97672700	-2.37704100
H	6.19901200	1.14729600	-2.28253600
C	4.17057200	3.77363200	-0.28052800
H	4.61329600	2.59966900	1.45036000
C	4.32377900	3.91852100	-1.65551400

H	5.17808700	3.08419700	-3.45073700
H	3.58635300	4.48343500	0.29759800
H	3.86950500	4.76162800	-2.16718200
H	1.08362700	-1.68690100	-0.70529700
H	-1.16970800	-0.61884300	-2.04840000
C	-5.63047200	2.65605100	-2.62016700
C	-4.89127700	1.99809000	-3.79029600
C	-6.79145800	3.52526600	-3.11758100
H	-6.05840700	1.85491700	-2.00997500
H	-4.08379700	1.35729900	-3.42778000
H	-5.58074500	1.38364400	-4.37719400
H	-4.46366000	2.75131300	-4.46020700
H	-7.33517100	3.96961500	-2.27918000
H	-6.42693500	4.34009600	-3.75159700
H	-7.49348300	2.92832800	-3.70755600
C	-3.33393100	3.65161300	1.85980100
C	-3.99475200	4.71338300	2.74816600
C	-1.83950300	3.54256700	2.18156400
H	-3.78115900	2.68292000	2.10010600
H	-5.07394600	4.76857100	2.57987500
H	-3.82273200	4.49150500	3.80573100
H	-3.57430600	5.70263200	2.53688800
H	-1.35840200	2.80685600	1.53192500
H	-1.33143000	4.50578300	2.06682300
H	-1.70869500	3.23087100	3.22255000
C	-2.38909600	6.49138700	-2.20613400
C	-0.86625900	6.37990500	-2.08559200
C	-2.88344200	7.81134900	-1.60294100
H	-2.64404300	6.49006000	-3.27341500
H	-0.50492100	5.43819400	-2.50851700
H	-0.37711400	7.20722100	-2.60897600
H	-0.55355000	6.41818800	-1.03651100
H	-3.96660500	7.91067800	-1.71037200
H	-2.64641500	7.85716400	-0.53490800

H	-2.40555400	8.66602600	-2.09155800
C	-1.75930600	-4.11669200	-2.17872300
C	-2.11352900	-5.57271100	-2.50424600
C	-0.97009700	-3.50811400	-3.34629000
H	-2.68748300	-3.54623500	-2.07980800
H	-2.75303700	-6.01833200	-1.73846000
H	-2.63886700	-5.63423000	-3.46231300
H	-1.20533900	-6.18032800	-2.57717700
H	-0.59801700	-2.50983700	-3.09968400
H	-0.10309700	-4.12365900	-3.60692700
H	-1.60653800	-3.43512600	-4.23315400
C	-1.22357600	-2.49500300	2.67566300
C	-0.49841400	-1.16028300	2.89442700
C	-1.08146600	-3.40322100	3.90290800
H	-2.28848800	-2.28022200	2.55768500
H	-0.62013900	-0.50119900	2.02829200
H	-0.90374000	-0.64559700	3.77126800
H	0.57295600	-1.32348900	3.05522100
H	-1.60309700	-4.35242800	3.75198500
H	-0.03050900	-3.62602300	4.11228000
H	-1.50175500	-2.91514500	4.78744700
C	2.53072500	-4.90223400	0.33238900
C	2.48899300	-6.18716800	1.17109600
C	3.61006300	-3.95278400	0.85382700
H	2.79415400	-5.17669600	-0.69684500
H	1.74444500	-6.88838800	0.78408800
H	3.46564300	-6.68078900	1.17215300
H	2.22397900	-5.95434900	2.20812200
H	3.65531800	-3.03314000	0.26619800
H	3.41518500	-3.66846500	1.89425200
H	4.59101000	-4.43639500	0.81360600
H	3.62286600	-0.76730000	-1.18567000
